# A Multi-Label Classifier for Predicting the Subcellular Localization of Gram-Negative Bacterial Proteins with Both Single and Multiple Sites

**DOI:** 10.1371/journal.pone.0020592

**Published:** 2011-06-17

**Authors:** Xuan Xiao, Zhi-Cheng Wu, Kuo-Chen Chou

**Affiliations:** 1 Computer Department, Jing-De-Zhen Ceramic Institute, Jing-De-Zhen, China; 2 Gordon Life Science Institute, San Diego, California, United States of America; King's College London, United Kingdom

## Abstract

Prediction of protein subcellular localization is a challenging problem, particularly when the system concerned contains both singleplex and multiplex proteins. In this paper, by introducing the “multi-label scale” and hybridizing the information of gene ontology with the sequential evolution information, a novel predictor called **iLoc-Gneg** is developed for predicting the subcellular localization of Gram-positive bacterial proteins with both single-location and multiple-location sites. For facilitating comparison, the same stringent benchmark dataset used to estimate the accuracy of **Gneg-mPLoc** was adopted to demonstrate the power of **iLoc-Gneg**. The dataset contains 1,392 Gram-negative bacterial proteins classified into the following eight locations: (1) cytoplasm, (2) extracellular, (3) fimbrium, (4) flagellum, (5) inner membrane, (6) nucleoid, (7) outer membrane, and (8) periplasm. Of the 1,392 proteins, 1,328 are each with only one subcellular location and the other 64 are each with two subcellular locations, but none of the proteins included has 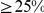 pairwise sequence identity to any other in a same subset (subcellular location). It was observed that the overall success rate by jackknife test on such a stringent benchmark dataset by **iLoc-Gneg** was over 91%, which is about 6% higher than that by **Gneg-mPLoc**. As a user-friendly web-server, **iLoc-Gneg** is freely accessible to the public at http://icpr.jci.edu.cn/bioinfo/iLoc-Gneg. Meanwhile, a step-by-step guide is provided on how to use the web-server to get the desired results. Furthermore, for the user's convenience, the **iLoc-Gneg** web-server also has the function to accept the batch job submission, which is not available in the existing version of **Gneg-mPLoc** web-server. It is anticipated that **iLoc-Gneg** may become a useful high throughput tool for Molecular Cell Biology, Proteomics, System Biology, and Drug Development.

## Introduction

Bacteria can be divided into two groups: Gram-positive and Gram-negative. Gram-positive bacteria are those that are stained dark blue or violet by Gram staining; while Gram-negative bacteria cannot retain the stain, instead taking up the counter-stain and appearing red or pink.

It has special meaning for both basic research and drug design to study bacteria because (1) they are the workhorses for the fields of molecular biology, biochemistry, and genetics due to their ability to quickly grow and being relatively easier to be manipulated, and (2) they are both harmful and useful. With the explosion of protein sequences generated in the post-genomic era, we are challenged to develop computational methods for timely and accurately identifying the subcellular locations of newly discovered bacterial proteins based on their sequence information alone because this kind of knowledge will be very useful for selecting proper bacterial proteins for a special target, or screening and prioritizing candidates in drug design.

Actually, numerous predictors were developed for identifying subcellular localization of proteins in various organisms (see [1], [2] as well as the long list of references cited in the two review papers). However, those that are specialized for dealing with Gram-negative proteins are only a few. They are called “**PSORT**” [1], [3], [4], “**PSORT-B**” [5], and **PSORTb v.2.0** [6]. All these methods have played important roles in stimulating the development of this area. To improve the prediction coverage scope and the quality of benchmark datasets, the predictor called **Gneg-PLoc** [7] was developed. Compared with the previous methods, **Gneg-PLoc** extended the coverage scope from five to eight subcellular location sites. Also, the benchmark datasets used to train and test the predictor have been significantly refined. For instance, the benchmark datasets used in **PSORT-B** [5] contain many proteins with pairwise sequence identity higher than 90%, while in the benchmark datasets of **Gneg-PLoc** [7] none of the proteins included has 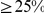 pairwise sequence identity to any other in a same subcellular location; i.e., the latter is much more stringent and rigorous than the former in excluding the homology bias and redundancy. Also, **Gneg-PLoc** was able to yield higher success rates.

However, all the aforementioned predictors cannot be used to deal with multiplex proteins that may simultaneously exist at, or move between, two or more different subcellular locations. Proteins with multiple locations or dynamic feature of this kind are particularly interesting because they may have some very special biological functions intriguing to investigators in both basic research and drug discovery [8], [9]. Particularly, as pointed out by Millar et al. [10], recent evidences have indicated that an increasing number of proteins have multiple locations in the cell.

To make **Gneg-PLoc** [7] be able to deal with multiplex Gram-negative proteins as well, a predictor called **Gneg-mPLoc** [11] was developed recently, where the character “m” in front of “PLoc” stands for “multiple”, meaning that it can be also used to deal with Gram-negative bacterial proteins with multiple locations.

However, **Gneg-mPLoc** has the following shortcomings. **(1)** In predicting the number of subcellular location sites for a query Gram-negative protein, an optimal threshold factor 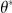 (see Eq.48 of [2]) was adopted without providing its statistical implication and detailed learning process. It would be more instructive if we could find a more intuitive approach to determine this with a more natural manner. **(2)** In formulating the protein samples, only the integer numbers 0 and 1 were used to reflect the GO (gene ontology) information [12], [13]. Such an over-simplified formulation might cause some useful information lost so as to limit the prediction quality. **(3)** Although a web-server for **Gneg-mPLoc** has been established at http://www.csbio.sjtu.edu.cn/bioinf/Gneg-multi/, only one query protein sequence at a time is allowed when using the web-server to conduct prediction. For the convenience of users in handling many query Gram-negative protein sequences, such a rigid limit should be improved.

The present study was dedicated to develop a new and more powerful predictor, called **iLoc-Gneg**, for predicting Gram-negative bacterial protein subcellular localization by addressing the above three problems.

To establish a really useful statistical predictor for protein system, we usually need to consider the following procedures [14]: (1) select or construct a valid benchmark dataset to train and test the predictor; (2) formulate the protein samples with an effective mathematical expression that can truly reflect their intrinsic correlation with the attribute to be predicted; (3) introduce or develop a powerful algorithm (or engine) to operate the prediction; (4) properly perform cross-validation tests to objectively evaluate the anticipated accuracy of the predictor; (5) establish a user-friendly web-server [15] for the predictor that is accessible to the public. Below, let us describe how to realize these steps one by one.

## Materials and Methods

Here, we choose to use the same dataset 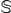 in establishing **Gneg-mPLoc** [11] as the benchmark dataset for the current study. The reasons doing so are as follows. **(1)** The dataset was constructed specialized for Gram-negative bacterial proteins and it can cover 8 subcellular location sites; compared with the other datasets such as the one in **PSORTb v.2.0** [6] that only covered 5 subcellular locations, the coverage scope of the dataset 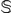 from [11] is much wider. **(2)** None of proteins included in 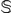 has 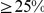 pairwise sequence identity to any other in a same subcellular location; compared with most of the other benchmark datasets in this area, the dataset 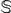 is much more rigorous in excluding homology bias and redundancy. **(3)** It contains both singleplex and multiplex proteins and hence can be used to train and test a predictor developed aimed at being able to deal with proteins with both single and multiple location sites. **(4)** Using the dataset 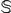 will also make it easier to compare the new predictor with the existing one because the tested results by **Gneg-mPLoc** on 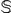 have been well documented and reported [11].

**Figure 1:**
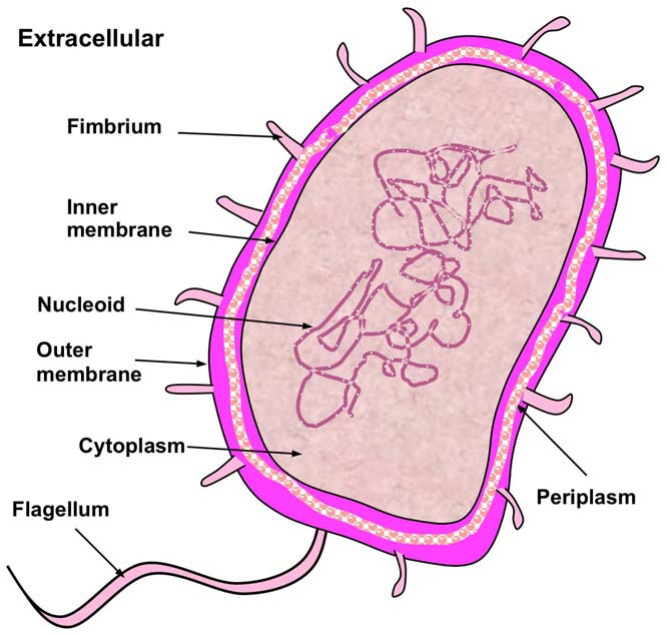
Illustration to show the 8 subcellular locations of Gram-negative bacterial proteins. The 8 locations are: (1) cytoplasm, (2) extracellular, (3) fimbrium, (4) flagellum, (5) inner membrane, (6) nucleoid, (7) outer membrane, and (8) periplasm. Note that in prokaryotic life forms, the nucleoid region is the part of the cell that contains the DNA molecule; unlike the true nucleus of eukaryotes, it is not delimited by a membrane.

The dataset 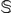 contains 1,392 Gram-negative bacterial protein sequences, of which 1,328 belong to one subcellular location, 64 to two locations, and none to three or more locations. The dataset covers 8 subcellular locations (**Fig. 1**), as can be formulated by
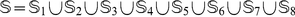
(1)where 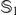 represents the subset for the subcellular location of cell inner membrane, 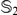 for cell outer membrane, 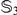 for cytoplasm, 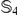 for extracellular, and so forth (**Table 1**); while 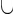 represents the symbol for “union” in the set theory. To avoid homology bias and redundancy, none of the proteins in 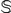 has 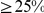 pairwise sequence identity to any other in a same subset. For convenience, hereafter let us just use the subscripts of **Eq.1** as the codes of the 8 location sites; i.e., “1” for “cell membrane”, “2” for “cell wall”, “3” for “chloroplast”, and so forth (**Table 2**).

**Table 1:** Breakdown of the Gram-negative bacterial protein benchmark dataset 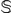 taken from [11].

Subset	Subcellular location	Number of proteins
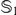	Cell inner membrane	557
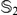	Cell outer membrane	124
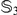	Cytoplasm	410
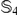	Extracellular	133
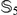	Fimbrium	32
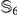	Flagellum	12
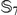	Nucleoid	8
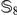	Periplasm	180
Total number of locative proteins 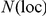		1,456^a^
Total number of different proteins 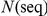		1,392^b^

None of proteins included here has 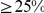 sequence identity to any other in a same subcellular location.

a See Eqs.36–38 of [2] for the definition about the number of locative proteins, and its relation with the number of different proteins.

b Of the 1,392 different proteins, 1,328 have one subcellular location, 64 have two locations, and none have three or more locations.

**Table 2:** A comparison of the jackknife success rates by **Gnec-mPLoc** [11] and the current **iLoc-Gneg** on the benchmark dataset 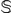 (cf. Supporting Information S1) that covers 8 location sites of Gram-negative bacterial proteins in which none of the proteins included has 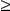
25% pairwise sequence identity to any other in a same location.

Code	Subcellular location	Success rate by jackknife test	
		Gneg-mPLoc^a^	iLoc-Gneg^b^
1	Cell inner membrane	525/557 = 94.3%	539/557 = 96.8%
2	Cell outer membrane	105/124 = 84.7%	103/124 = 83.1%
3	Cytoplasm	357/410 = 87.1%	367/410 = 89.5%
4	Extracellular	79/133 = 59.4%	115/133 = 86.5%
5	Fimbrium	28/32 = 87.5%	30/32 = 93.8%
6	Flagellum	0/12 = 0.0%	12/12 = 100%
7	Nucleoid	0/8 = 0.0%	4/8 = 50%
8	Periplasm	154/180 = 85.6%	161/180 = 89.4%
Overall^c^		1248/1456 = **85.7%**	1331/1456 = **91.4%**

a The predictor from [11].

b The predictor proposed in this paper.

c Note that instead of 1,392 (the number of total different Gram-positive bacterial proteins), here we use 1,456 (the number of total different locative proteins) for the denominator. This is because some of the Gram-negative bacterial proteins in 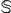 may have more than one location site. See footnotes a and b of Table 1 for further explanation.

For readers' convenience, the corresponding accession numbers and protein sequences in 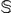 are given in Supporting Information S1.

Note that because some proteins may occur in two or more locations, the 1,392 Gram-negative proteins actually correspond to 1,456 locative proteins. The concept of “locative proteins” was introduced for studying proteins with multiple subcellular location sites, as elaborated in [2].

To develop a powerful method for statistically predicting protein subcellular localization according to the sequence information, one of the most important things is to formulate the protein sequences with an effective mathematical expression that can truly reflect the intrinsic correlation with their subcellular localization [14]. However, it is by no means an easy job to realize this because this kind of correlation is usually deeply “buried” or hidden in piles of complicated sequences.

The most straightforward method to formulate the sample of a query protein 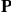 was just using its entire amino acid sequence, as can be generally written by
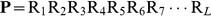
(2)where 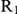 represents the 1^st^ residue of the protein 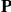, 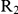 the 2^nd^ residue, …, 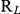 the 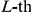 residue, and they each belong to one of the 20 native amino acids. In order to identify its subcellular location(s), the sequence-similarity-search-based tools, such as BLAST [16], [17], was utilized to search protein database for those proteins that have high sequence similarity to the query protein 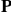. Subsequently, the subcellular location annotations of the proteins thus found were used to deduce the subcellular location(s) for 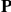. Unfortunately, although it was quite intuitive and able to contain the entire information of a protein sequence, this kind of straightforward sequential model failed to work when the query protein 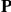 did not have significant sequence similarity to any location-known proteins.

Thus, various non-sequential or discrete models to formulate protein samples were proposed in hopes to establish some sort of correlation or cluster manner by which the prediction quality could be improved.

Among the discrete models for a protein sample, the simplest one is its amino acid (AA) composition or AAC [18]. According to the AAC-discrete model, the protein 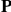 of **Eq.2** can be formulated by [19], [20]

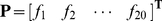
(3)where 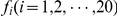 are the normalized occurrence frequencies of the 20 native amino acids in protein 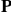, and 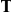 the transposing operator. Many methods for predicting protein subcellular localization were based on the AAC-discrete model (see, e.g., [19], [21], [22], [23], [24]). However, as we can see from **Eq.3**, if using the ACC model to represent the protein 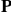, all its sequence-order effects would be lost, and hence the prediction quality might be limited.

To avoid completely lose the sequence-order information, the pseudo amino acid composition (PseAAC) was proposed to represent the sample of a protein, as formulated by [25]

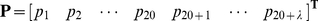
(4)where the first 20 elements are associated with the 20 elements in **Eq.3** or the 20 amino acid components of the protein 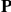, while the additional 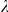 factors are used to incorporate some sequence-order information via a series of rank-different correlation factors along a protein chain. For a brief introduction about PseAAC, please see a Wikipedia article at http://en.wikipedia.org/wiki/Pseudo_amino_acid_composition.

According to [14], the PseAAC for a protein 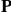 can be generally formulated as
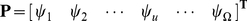
(5)where the subscript 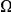 is an integer, and its value as well as the components 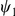, 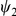, … will depend on how to extract the desired information from the amino acid sequence of 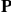 (cf. **Eq.2**). As a general form, **Eq.5** can cover various different modes of PseAAC. For example, when its elements are given by
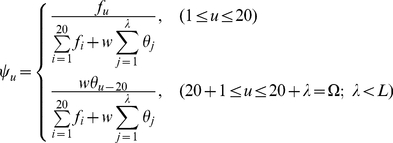
(6)we immediately obtain the formulation of PseAAC as originally introduced in [25], where the meanings for 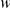, 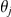, and 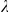 were clearly elaborated and hence there is no need to repeat here.

Below, let us use the general form of PseAAC (**Eq.5**) to find the formulations to reflect the core and essential features of protein samples that are closely correlated with their subcellular localization.

### 1. GO (Gene Ontology) Formulation

GO database [12] was established according to the molecular function, biological process, and cellular component. Accordingly, protein samples defined in a GO database space would be clustered in a way better reflecting their subcellular locations [2], [26]. However, in order to incorporate more information, instead of only using 0 and 1 elements as done in [11], here let us use a different approach as described below.

#### Step 1

Compression and reorganization of the existing GO numbers. The GO database (version 74.0 released 30 July 2009) contains many GO numbers. However, these numbers do not increase successively and orderly. For easier handling, some reorganization and compression procedure was taken to renumber them. For example, after such a procedure, the original GO numbers GO:0000001, GO:0000002, GO:0000003, GO:0000009, GO:00000011, GO:0000012, GO:0000015, …, GO:0090204 would become GO_compress: 00001, GO_compress: 00002, GO_compress: 00003, GO_compress: 00004, GO_compress: 00005, GO_compress: 00006, GO_compress: 00007, ……, GO_compress: 11118, respectively. The GO database obtained thru such a treatment is called GO_compress database, which contains 11,118 numbers increasing successively from 1 to the last one.

#### Step 2

Using **Eq.5** with 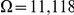, the protein 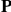 can be formulated as
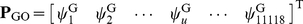
(7)where 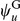 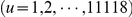 are defined via the following steps.

#### Step 3

Use BLAST [27] to search the homologous proteins of the protein 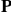 from the Swiss-Prot database (version 55.3), with the expect value 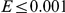 for the BLAST parameter.

#### Step 4

Those proteins which have 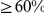 pairwise sequence identity with the protein 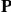 are collected into a set, 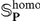, called the “homology set” of 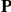. All the elements in 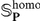 can be deemed as the “representative proteins” of 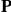, sharing some similar attributes such as structural conformations and biological functions [28], [29], [30]. Because they were retrieved from the Swiss-Prot database, these representative proteins must each have their own accession numbers.

#### Step 5

Search each of these accession numbers collected in Step 4 against the GO database at http://www.ebi.ac.uk/GOA/ to find the corresponding GO numbers [31].

#### Step 6

Based on the results obtained in Step 5, the elements in **Eq.7** can be written as
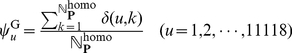
(8)where 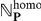 is the number of representative proteins in 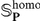, and
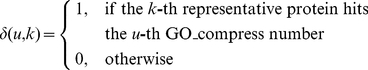
(9)


As we can see from **Eq.7**, the GO formulation derived from the above steps consists of 11,118 real numbers rather than only the elements 0 and 1 as in the GO formulation adopted in [11].

Note that the GO formulation of **Eq.6** may become a naught vector or meaningless under any of the following situations: **(1)** the protein 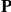 does not have significant homology to any protein in the Swiss-Prot database, i.e., 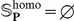 meaning the homology set 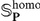 is an empty one; **(2)** its representative proteins do not contain any useful GO information for statistical prediction based on a given training dataset.

Under such a circumstance, let us consider using the sequential evolution formulation to represent the protein 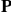, as described below.

### 2. SeqEvo (Sequential Evolution) Formulation

Biology is a natural science with historic dimension. All biological species have developed continuously starting out from a very limited number of ancestral species. It is true for protein sequence as well [30]. Their evolution involves changes of single residues, insertions and deletions of several residues [32], gene doubling, and gene fusion. With these changes accumulated for a long period of time, many similarities between initial and resultant amino acid sequences are gradually eliminated, but the corresponding proteins may still share many common attributes, such as having basically the same biological function and residing in a same subcellular location.

To incorporate the sequential evolution information into the PseAAC of **Eq.4**, here let us use the information of the PSSM (Position-Specific Scoring Matrix) [27], as described below.

#### Step 1

According to [27], the sequential evolution information of protein 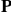 can be expressed by a 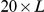 matrix as given by
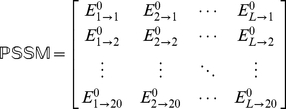
(10)where 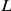 is the length of 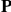 (counted in the total number of its constituent amino acids as shown in **Eq.1**), 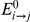 represents the score of the amino acid residue in the 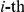 position of the protein sequence being changed to amino acid type 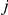 during the evolutionary process. Here, the numerical codes 1, 2, …, 20 are used to denote the 20 native amino acid types according to the alphabetical order of their single character codes. The 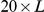 scores in **Eq.10** were generated by using PSI-BLAST [27] to search the UniProtKB/Swiss-Prot database (Release 2010_04 of 23-Mar-2010) through three iterations with 0.001 as the 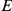-value cutoff for multiple sequence alignment against the sequence of the protein 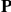. However, according to the formulation of **Eq.10**, proteins with different lengths will correspond to column-different matrices causing difficulty for developing a predictor able to uniformly cover proteins of any length. To make the descriptor become a size-uniform matrix, let us consider the following steps.

#### Step 2

Use the elements in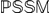 of Eq.10 to define a new matrix 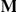 as formulated by
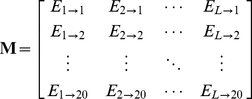
(11)with
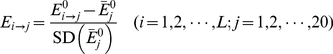
(12)where
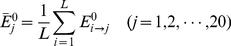
(13)is the mean for 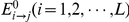 and
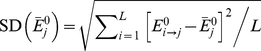
(14)is the corresponding standard deviation.

#### Step 3

Introduce a new matrix generated by multiplying 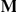 with its own transpose matrix 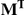; i.e.,
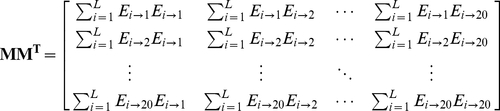
(15)which contains 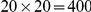 elements. Since 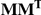 is a symmetric matrix, we only need the information of its 210 elements, of which 20 are the diagonal elements and 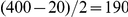 are the lower triangular elements, to formulate the protein 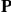; i.e., the general PseAAC form of **Eq.5** can now be formulated as
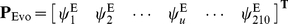
(16)where the components 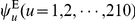 are respectively taken from the 210 diagonal and lower triangular elements of **Eq.15** by following a given order, say from left to right and from the 1^st^ row to the last as illustrated by following equation
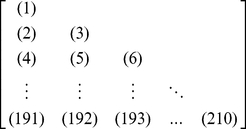
(17)where the numbers in parentheses indicate the order of elements taken from **Eq.15** for **Eq.16**.

### 3. The Self-consistency Formulation Principle

Regardless of using which formulation to represent protein samples, the following self-consistency principle must be observed during the course of prediction: if the query protein 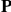 was defined in the form of 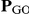 (see **Eq.7**), then all the protein samples used to train the prediction engine should also be expressed in the GO formulation; if the query protein was defined in the form of 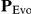 (see **Eq.16**), then all the training data should be expressed in the SeqEvo formulation as well.

Below, let us consider the algorithm or operation engine for conducting the prediction.

### 4. Multi-Label KNN (K-Nearest Neighbor) Classifier

In this study, let us introduce a novel classifier, called the multi-label KNN or abbreviated as ML-KNN classifier, to predict the subcellular localization for the systems that contain both single-location and multiple-location proteins.

Suppose the 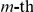 subset 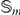 of 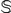 (**Eq.1**) contains 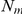 Gram-negative proteins, and 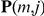 is the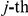 one in that subset. Thus, we have
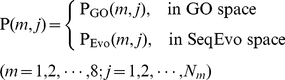
(18)where 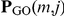 and 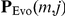 have the same forms as 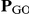
(**Eq.7**), and 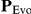
(**Eq.16**), respectively; the only difference is that the corresponding constituent elements are derived from the amino acid sequence of 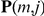 instead of 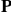.

In sequence analysis, there are many different scales to define the distance between two proteins, such as Euclidean distance, Hamming distance [33], and Mahalanobis distance [18], [34], [35]. In [11], the distance between 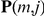 and 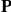 was defined by 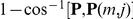. However, we have observed that when the GO descriptor was formulated with real numbers, better outcomes would be resulted by using the Euclidean metric; i.e., the distance between 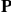 and 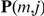 should be defined here by
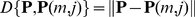
(19)where 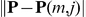 represents the module of the vector difference between 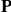 and 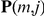 in the Euclidean space. According to **Eq.19**, when 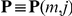 we have 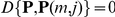, indicating the distance between these two protein sequences is zero and hence they have perfect or 100% similarity.

Suppose 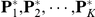 are the *K* nearest neighbor proteins to the protein 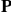 that forms a set denoted by 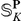, which is a subset of 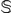; i.e.,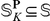. Based on the *K* nearest neighbor proteins in 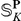, let us define an accumulation-layer (AL) scale, given by
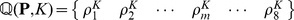
(20)where
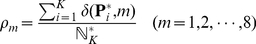
(21)where
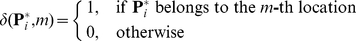
(22)and
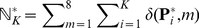
(23)Note that 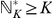 because a protein may belong to one or more subcellular location sites in the current system.

Now, for a query protein 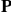, its subcellular location(s) will be predicted according to the following steps.

#### Step 1

The number of how many different subcellular locations it belongs to will be determined by its nearest neighbor protein in 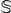. For example, suppose 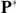 is the nearest protein to 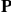 in 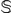. If 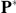 has only one subcellular location, then 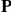 will also have only one location; if 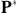 has two subcellular locations, then 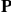 will also have two locations; and so forth. In general, if 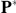 belongs to 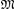 different location sites, then 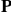 will be predicted to have the same number, 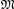, of subcellular locations as well, as can be formulated by
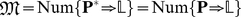
(24)where 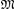 is an integer 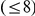, 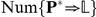 represents the number of different subcellular locations to which 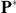 belongs, and 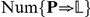 the number of different subcellular locations to which 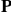 belongs.

#### Step 2

However, the concrete location site(s) to which 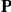 belongs will not be determined by the location site(s) of 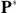, but by the element(s) in **Eq.20** that has (have) the highest score(s), as can be expressed by 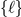, the subscript(s) of **Eq.1**. For example, if 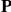 is found belonging to only one location 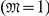 in Step 1, and the highest score in **Eq.20** is 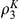, then 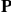 will be predicted as 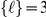 meaning that it belongs to 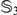 or resides at “cytoplasm” (cf. **Table 1**). If 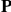 is found belonging to two locations 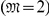, and the first two highest scores in **Eq.20** are 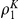 and 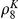, then 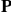 will be predicted as 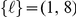 meaning that it belongs to 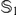 and 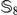 or resides simultaneously at “cell inner membrane” and “periplasm”. And so forth. In other words, the concrete predicted subcellular location(s) can be formulated as
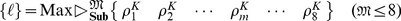
(25)where the operator “
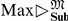
” means identifying the 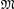 highest scores for the elements in the brackets right after it, followed by taking their 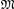 subscripts.

**Figure 2:**
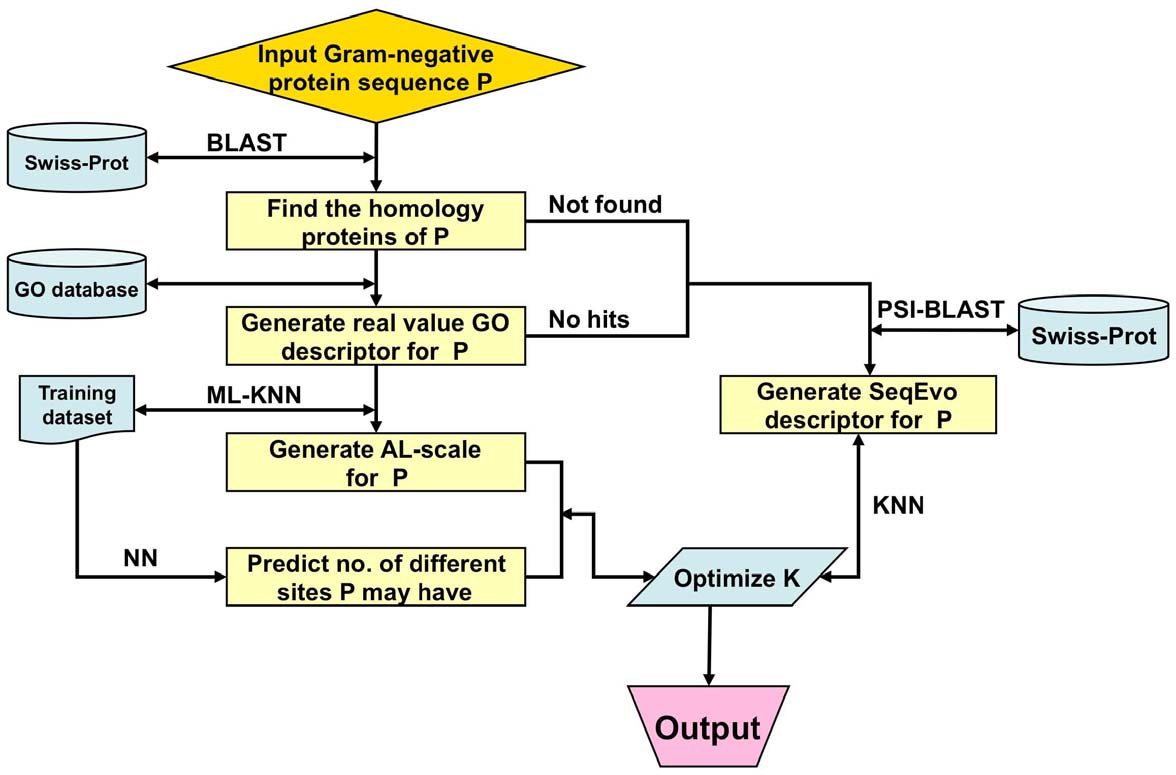
A flowchart to show the prediction process of iLoc-Gneg.

The entire classifier thus established is called **iLoc-Gneg**, which can be used to predict the subcellular localization of both singleplex and multiplex Gram-negative bacterial proteins. To provide an intuitive picture, a flowchart is provided in **Fig. 2** to illustrate the prediction process of **iLoc-Gneg**.

### 5. Protocol Guide

For user's convenience, a web-server for **iLoc-Gneg** was established. Below, let us give a step-by-step guide on how to use it to get the desired results.

#### Step 1

**Figure 3:**
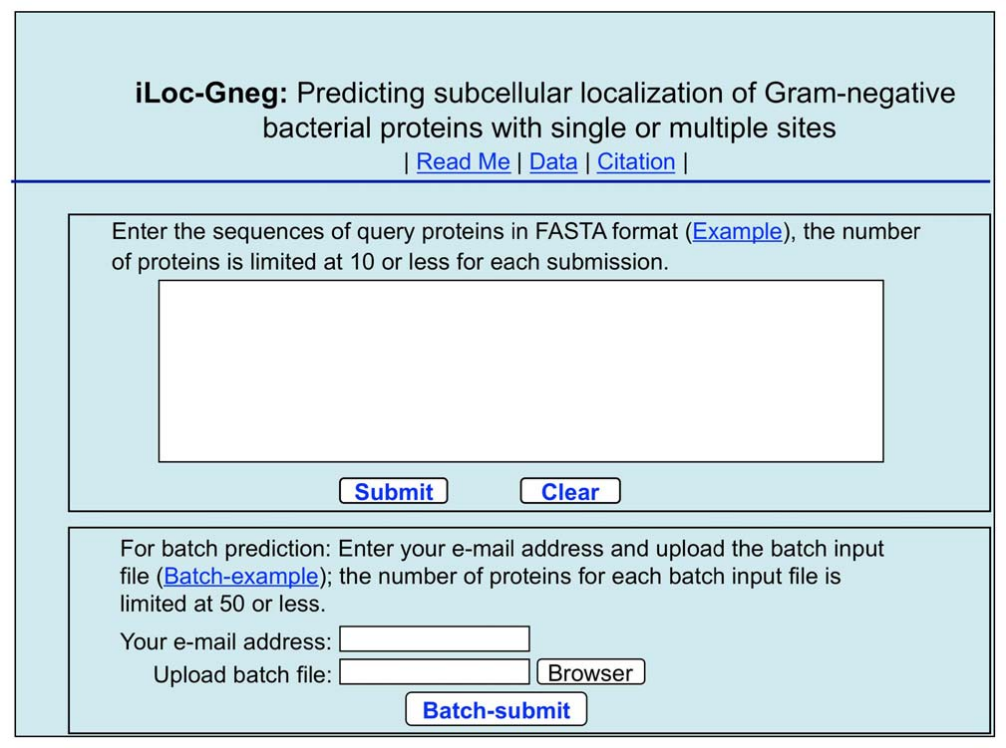
A semi-screenshot to show the top page of the iLoc-Gneg web-server. Its website address is at http://icpr.jci.edu.cn/bioinfo/iLoc-Gneg.

Open the web server at site http://icpr.jci.edu.cn/bioinfo/iLoc-Gneg and you will see the top page of the predictor on your computer screen, as shown in **Fig. 3**. Click on the Read Me button to see a brief introduction about **iLoc-Gneg** predictor and the caveat when using it.

#### Step 2

Either type or copy and paste the query protein sequence into the input box at the center of **Fig. 3**. The input sequence should be in the FASTA format. A sequence in FASTA format consists of a single initial line beginning with a greater-than symbol (“>”) in the first column, followed by lines of sequence data. The words right after the “>” symbol in the single initial line are optional and only used for the purpose of identification and description. All lines should be no longer than 120 characters and usually do not exceed 80 characters. The sequence ends if another line starting with a “>” appears; this indicates the start of another sequence. Example sequences in FASTA format can be seen by clicking on the Example button right above the input box. For more information about FASTA format, visit http://en.wikipedia.org/wiki/Fasta_format. Different with **Gneg-mPLoc** [11], where only one query protein sequence at a time is allowed for each submission, now the maximum number of query proteins for each submission can be 10.

#### Step 3

**Figure 4:**
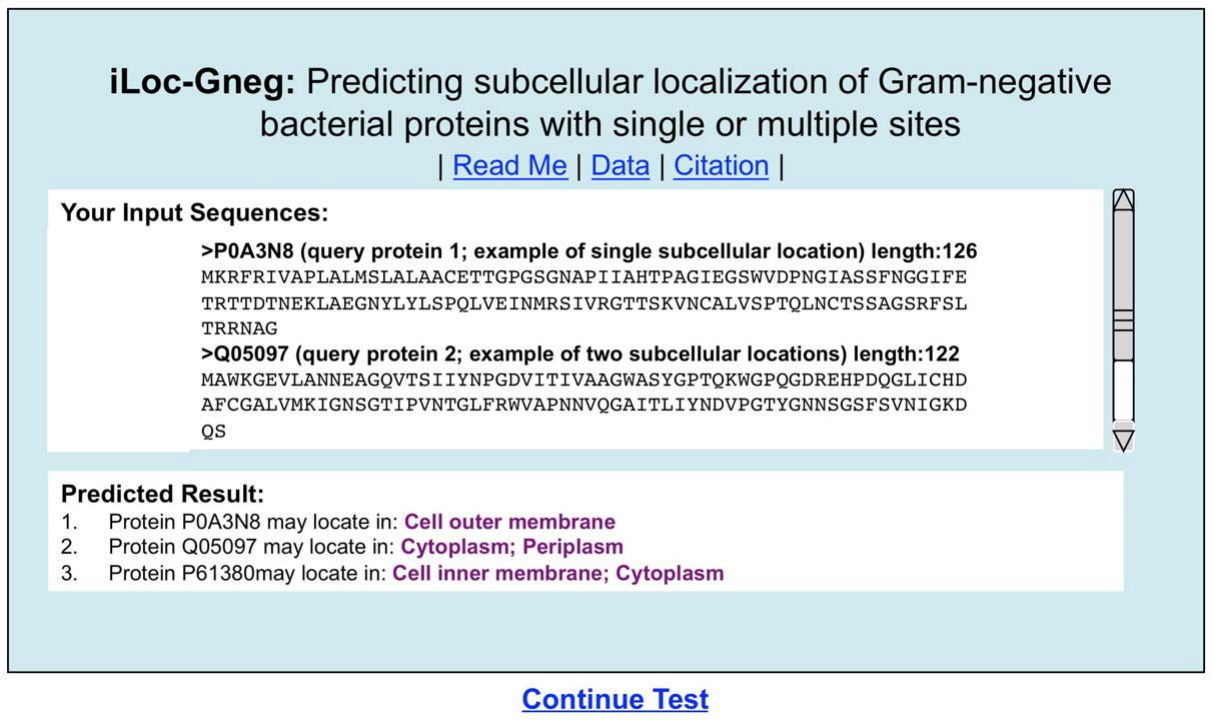
A semi-screenshot to show the output of iLoc-Gneg. The input was taken from the three protein sequences listed in the Example window of the **iLoc-Gneg** web-server (cf. Fig. 3).

Click on the Submit button to see the predicted result. For example, if you use the three query protein sequences in the Example window as the input, after clicking the Submit button, you will see **Fig. 4** shown on your screen, indicating that the predicted result for the 1^st^ query protein is “**Cell outer membrane**”, that for the 2^nd^ one is “**Cytoplasm; Periplasm**”, and that for the 3^rd^ one is “**Cell inner membrane; Cytoplasm**”. In other words, the 1^st^ query protein (P0A3N8) is a single-location one residing at “cell outer membrane” only, the 2^nd^ one (Q05097) can simultaneously reside in two different sites (“cytoplasm” and “periplasm”), and the 3^rd^ one (P61380) can also simultaneously reside in two different sites (“cell inner membrane” and “cytoplasm”). All these results are exactly the same as observed by experiments as shown in the Supporting Information S1. It takes about 10 seconds for the above computation before the predicted results appear on your computer screen; the more number of query proteins and longer of each sequence, the more time it is usually needed.

#### Step 4

As shown on the lower panel of **Fig. 3**, you may also choose the batch prediction by entering your e-mail address and your desired batch input file (in FASTA format) via the “Browse” button. To see the sample of batch input file, click on the button Batch-example. The maximum number of the query proteins for each batch input file is 50. After clicking the button Batch-submit, you will see “Your batch job is under computation; once the results are available, you will be notified by e-mail.” Note that if you submit a batch input file from an Apple computer, although it looks like in the FASTA format, your input might change to non-FASTA format in the server end and cause errors. Under such a circumstance, the safest way is to submit your input file with a pdf format.

#### Step 5

Click on the Citation button to find the relevant papers that document the detailed development and algorithm of **iLoc-Gneg**.

#### Step 6

Click on the Data button to download the benchmark datasets used to train and test the **iLoc-Gneg** predictor.

#### Caveat

To obtain the predicted result with the expected success rate, the entire sequence of the query protein rather than its fragment should be used as an input. A sequence with less than 50 amino acid residues is generally deemed as a fragment. Also, if the query Gram-negative protein is known not one of the 8 locations as shown in **Fig. 1**, stop the prediction because the result thus obtained will not make any sense.

## Results and Discussion

In statistical prediction, it would be meaningless to simply report a success rate of a predictor without specifying what method and benchmark dataset were used to test its accuracy [14]. As is well known, the following three methods are often used to examine the quality of a predictor: independent dataset test, subsampling test, and jackknife test [36]. Owing to that subsampling test and jackknife test can be performed with one benchmark dataset and that independent dataset test can be treated as a special case of subsampling test, one benchmark dataset would suffice to serve all the three kinds of cross-validation. However, as demonstrated by Eq.1 of [37] and elucidated in [2], among the three cross-validation methods, the jackknife test is deemed the least arbitrary that can always yield a unique result for a given benchmark dataset and hence has been widely recognized and increasingly used to examine the power of various predictors (see, e.g., [38], [39], [40], [41], [42], [43], [44], [45], [46], [47], [48], [49], [50], [51], [52], [53], [54], [55], [56]). Accordingly, in this study, the jackknife test will be adopted to evaluate the power of **iLoc-Gneg** as well.

However, even if using the jackknife test to examine the accuracy, a same predictor may still yield obviously different success rates when tested by different benchmark datasets. This is because the more stringent of a benchmark dataset in excluding homologous sequences, the more difficult for a predictor to achieve a high success rate. Also, the more number of subsets (subcellular locations) a benchmark dataset covers, the more difficult to achieve a high overall success rate, as elaborated in a recent review [14].

As mentioned in the Materials section, the benchmark dataset used in this study is 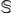 (cf. Supporting Information S1), which is the same benchmark dataset constructed in [11] for **Gneg-mPLoc**.

Actually, for such a dataset containing both single-location and multiple-location Gram-negative proteins distributed among 8 subcellular location sites, so far only one existing predictor, i.e., **Gneg-mPLoc** [11], had the capacity to deal with it. Therefore, to demonstrate the power of the current predictor, it would suffice to just compare **iLoc-Gneg** with **Gneg-mPLoc** [11].

Listed in **Table 2** are the results obtained with **Gneg-mPLoc** [11] and **iLoc-Gneg** on the aforementioned benchmark dataset 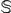 by the jackknife test. As we can see from **Table 2**, for such a stringent and complicated benchmark dataset, the overall success rate achieved by **iLoc-Gneg** is over 91.4%, which is about 6% higher than that by **Gneg-mPLoc** [11].

Note that during the course of the jackknife test by **Gneg-mPLoc** and **iLoc-Gneg**, the false positives (over-predictions) and false negatives (under-predictions) were also taken into account to reduce the scores in calculating the overall success rate. As for the detailed process of how to count the over-predictions and under-predictions for a system containing both single-location and multiple-location proteins, see Eqs.43–48 and Fig. 4 in a comprehensive review [2].

To provide a more intuitive and easier-to-understand measurement, let us introduce a new scale, the so-called “absolute true” success rate, to reflect the accuracy of a predictor, as defined by
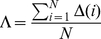
(26)where 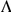 represents the absolute true rate, 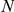 the number of total proteins investigated, and
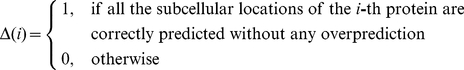
(27)According to the above definition, for a protein belonging to, say, two subcellular locations, if only one of the two is correctly predicted, or the predicted result contains a location not belonging to the two, the prediction score will be counted as 0. In other words, when and only when all the subcellular locations of a query protein are exactly predicted without any underprediction or overprediction, can the prediction be scored with 1. Therefore, the absolute true scale is much more strict and harsh than the scale used previously [2], [11] in measuring the success rate. However, even if using such a stringent criterion on the same benchmark dataset by the jackknife test, the overall absolute true success rate achieved by **iLoc-Gneg** was 1252/1392 = 89.9%.

Why can **iLoc-Gneg** enhance the success rate so remarkably? One of the key reasons is that the GO formulation for protein samples in **iLoc-Gneg** contains more information than that in **Gneg-mPLoc** [11], as elaborated as follows. For example, for the protein with the access number “P0A8U0” as denoted by 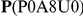, according to Steps 3 and 4 in the Section of “GO (Gene Ontology) Formulation”, we found 47 proteins that were homologous to it; i.e., 
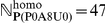
. Each of the 47 homologous proteins hit GO:0005886 (or GO_compress:00277) and GO:0016020 (or GO_compress:00830), and hence the two GO numbers were hit by a total of 47 times. Only one of the 47 proteins hit GO:0005737 (or GO_compress: 00269). Substituting these data into Eqs.8–9, we have
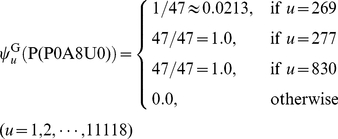
(28)In contrast, if the same protein was represented according to the formulation in **Gneg-mPLoc** [11], it would be
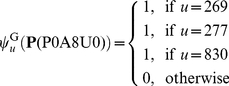
(29)It can be seen by a comparison of Eq.28 with Eq.29 that although the elements in the 269^th^, 277^th^, and 830^th^ components are all not zero in both formulations, the differences of their weights are completely ignored in Eq.29 as formulated in **Gneg-mPLoc** [11]. That is also why, when the sequence of 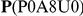 was inputted into **iLoc-Gneg** and **Gneg-mPLoc** [11] as a query protein for prediction, the former could accurately predict its both location sites (“cell inner membrane” and “cytoplasm”), while the latter could predict only one site (“cell inner membrane”) but miss the site of “cytoplasm”.

### Conclusions

Prediction of protein subcellular localization is a challenging problem, particularly when the system concerned contains both singleplex and multiplex proteins. The reasons why **iLoc-Gneg** can achieve higher success rates than **Gneg-mPLoc** are as follows. **(1)** The GO formulation used to represent protein samples in **iLoc-Gneg** is formed by the probabilities of hits (cf. Eqs.8–9) and hence contains more information than that in **Gneg-mPLoc** [11] where only the number “0” or “1” was used regardless how many hits were found to the corresponding component in the GO formulation. **(2)** The accumulation-layer scale has been introduced in **iLoc-Gneg** that is more natural and effective for dealing with proteins having both single and multiple subcellular locations.

## Supporting Information

Supporting Information S1
**This benchmark dataset** 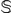 **includes 1,456 locative protein sequences (1,392 different proteins), classified into 8 Gram-negative subcellular locations.** Among the 1,392 different proteins, 1,328 belong to one location; and 64 to two locations. Both the accession numbers and sequences are given. None of the proteins has ≥25% sequence identity to any other in the same subset (subcellular location). See the text of the paper for further explanation.(PDF)
