# Supplementary material for: A Multi-Label Classifier for Predicting the Subcellular Localization of Gram-Negative Bacterial Proteins with Both Single and Multiple Sites
Source: PLoS One. 2011 Jun 17;6(6):e20592. doi: 10.1371/journal.pone.0020592 (PMC3117797; doi:10.1371/journal.pone.0020592)
Supplement: Supporting Information S1 — This benchmark dataset includes 1,456 locative protein sequences (1,392 different proteins), classified into 8 Gram-negative subcellular locations. Among the 1,392 different proteins, 1,328 belong to one location; and 64 to two locations. Both the accession numbers and sequences are given. None of the proteins has ≥25% sequence identity to any other in the same subset (subcellular location). See the text of the paper for further explanation. (PDF) [file pone.0020592.s001.pdf]

Online Supporting Information S1. This benchmark dataset $\mathbb{S}$ includes 1,456 locative protein sequences (1,392 different proteins), classified into 8 Gram-negative subcellular locations. Among the 1,392 different proteins, 1,328 belong to one location; and 64 to two locations. Both the accession numbers and sequences are given. None of the proteins has ≥25% sequence identity to any other in the same subset (subcellular location). See the text of the paper for further explanation.

---

(1) $\mathbb{S}_1$: 557 cell inner membrane proteins

>P76264
MNITATVLLAFGMSMDAFAASIGKGATLHKPKFSEALRTGLIFGAVETLTPLIGWGMGML
ASRFVLEWNHWIAFVLLIFLGGRMIIEGFRGADDEDEEPRRRHGFWLLVTTAIATSLDAM
AVGVGLAFLQVNIIATALAIGCATLIMSTLGMMVGRFIGSIIGKKAEILGGLVLIGIGVQ
ILWTHFHG
>P17201
MGRLNRFRLGKDGRREQASLSRRGFLVTSLGAGVMFGFARPSSANQIFPLDRSLPGDGAF
EPTIWCSIAPDGEITVNIIRAEMGQHIGTALARIIADEMEADWSKVRINYVDTDPKWGLM
VTGGSWSVWMTWDVFRQAGAATRTAMVEEGARLLGTTPDKCTVASSIVSAGGKQISFGDI
VAKGHPSHAFTPEEMAKLPLKPASERRLIGNAELKALDIPAKTNGTAIYGIDAKVEGMLY
GRPKMPPTRYGSKVRSVDDTEAKKIKGYVRYLLIDDPSQVVQGWVVVLAESYSAAIRATD
ALKVEWTPGETIHTSERDIQDRGRELINNKAGGVYIFNDDGVDQAFGSAHTVMDQEYTCA
SVLHYQLEPTNALAFEKDGVYEIHAGNQWQSLILPTLAKSLQVPESKVILRSYLLGGGFG
RRLNGDYMIPAALASKALGGKPVKLILTRSDDMQFDSFRSPSVQRVRMAFDASDRITAMD
YQAAAGWPTGVMAEAFMEKGVDGKPYDQFAIAGGDHWYEVGAFRVRALRNDLAEKTFRPG
WLRSVSPGWTSWGVECFLDEVAHRQKKDPAQFRLELLTGQGRNKGQAPDSVGGALRQAAV
VRRLMEKVNWGKTSLPKDTAMGLATTAGQERGMPTWDRCVAQVHVDRSTGVVTCQKLTIL
VDAGTVVDPDGAKAQTEGAALWGLSMVLFENTEIVNGMPVDRNLNTYTPLRIADTPEMDI
EFLPSTEKPMGLGEPGTTVVGPAIGNAIFNAVGVRLRHMPVRPADVLRGLQNG
>P0ABB4
MATGKIVQVIGAVVDVEFPQDAVPRVYDALEVQNGNERLVLEVQQQLGGGIVRTIAMGSS
DGLRRGLDVKDLEHPIEVPVGKATLGRIMNVLGEPVDMKGEIGEEERWAIHRAAPSYEEL
SNSQELLETGIKVIDLMCPFAKGGKVGLFGGAGVGKTVNMMELIRNIAIEHSGYSVFAGV
GERTREGNDFYHEMTDSNVIDKVSLVYGQMNEPPGNRLRVALTGLTMAEKFRDEGRDVLL
FVDNIYRYTLAGTEVSALLGRMPSAVGYQPTLAEEMGVLQERITSTKTGSITSVQAVYVP
ADDLTDPSPATTFAHLDATVVLSRQIASLGIYPAVDPLDSTSRQLDPLVVGQEHYDTARG
VQSILQRYQELKDIIAILGMDELSEEDKLVVARARKIQRFLSQPFFVAEVFTGSPGKYVS
LKDTIRGFKGIMEGEYDHLPEQAFYMVGSIEEAVEKAKKL
>P76169
MIKTTLLFFATALCEIIGCFLPWLWLKRNASIWLLLPAGISLALFVWLLTLHPAASGRVY
AAYGGVYVCTALMWLRVVDGVKLTLYDWTGALIALCGMLIIVAGWGRT
>P08550
MVWIDYAIIAVIAFSSLVSLIRGFVREALSLVTWGCAFFVASHYYTYLSVWFTGFEDELV
RNGIAIAVLFIATLIVGAIVNFVIGQLVEKTGLSGTDRVLGVCFGALRGVLIVAAILFFL
DSFTGVSKSEDWSKSQLIPQFSFIIRCFFDYLQSSSSFLPRA

>P08848
MFTSAHANRSPLTSASVRRPSHSVVEHSATGLISEIVYREDQPMMTQLLLLPLLQQLGQQ
SRWQLWLTPQQKLSKEWVQSSGLPLSKVMQINQMSPCNTLESMIRALRTGNYNVVIGWLT
DELTEQEHERLALAAEEGHPMGFIMRPVRNTSQPGRQLSGLKIHSNLYH
>P0AE14
MTLFTTLLVLIFERLFKLGEHWQLDHRLEAFFRRVKHFSLGRTLGMTIIAMGVTFLLLRA
LQGVLFNVPTLLVWLLIGLLCIGAGKVRLHYHAYLTAASRNDSHARATMAGELTMIHGVP
AGCDEREYLRELQNALLWINFRFYLAPLFWLIVGGTWGPVTLMGYAFLRAWQYWLARYQT
PHHRLQSGIDAVLHVLDWVPVRLAGVVYALIGHGEKALPAWFASLGDFHTSQYQVLTRLA
QFSLAREPHVDKVETPKAAVSMAKKTSFVVVVVIALLTIYGALV
>Q9RPF3
MKYSLPTTATAPFCPSAVSHSVAVPADASPLRKLALFVGPGLLVSVGYMDPGNWATAIEA
GSRFGYALLFVVVLASFSGMLLQSLCSRLGIATGRDLAQLSRERYRPGVARGQWLLAELS
IVATDLAEVLGAALAFHLLLGVSITTGVVLTAFDTLIVLALQGANFRRLEAIVLGLIATI
GACFFVELVLIGPYWPDVAAGLRPSWDTLSSQEPLYLAIGILGATVMPHNLYLHSSVVQT
RVSGDDAASKRSAIRFSRLDTIGSLSLALLVNAAILILAAAAFHGSGHTEVVEIQDAYHL
LDPLVGGALASFLFGFALLAAGQSSTFTGTIAGQVVMEGFLRAKIPCWQRRLITRGLALV
PALIGVLWLGEAAVGKLLVLSQVVLSLQLPFALWPLIRFSSDRGLMGEFVNPRWVSALAW
SLFGLISAANLTLLYFWFG
>P04124
MADLKPSLTGLTEEEAKEFHGIFVTSTVLYLATAVIVHYLVWTARPWIAPIPKGWVNLEG
VQSALSYLV
>P32129
MANLLNKFIMTRILAAITLLLSIVLTILVTIFCSVPIIIAGIVKLLLPVPVIWRKVSRFC
DFMMYCWCEGLAVLLHLNPHLQWEVHGLEGLSKKNWYLLICNHRSWADIVVLCVLFRKHI
PMNKYFLKQQLAWVPFLGLACWSLDMPFMKRYSRAYLLRHPERRGKDVETTRRSCEKFRL
HPTTIVNFVEGSRFTQEKHQQTHSTFQNLLPPKAAGIAMALNVLGKQFDKLLNVTLCYPD
NNRQPFFDMLSGKLTRIVVHVDLQPIADELHGDYINDKSFKRHFQQWLNSLWQEKDRLLT
SLMSSQRQNK
>P56580
MTHIRIEKGTGGWGGPLELKATPGKKIVYITAGTRPAIVDKLAQLTGWQAIDGFKEGEPA
EAEIGVAVIDCGGTLRCGIYPKRRIPTINIHSTGKSGPLAQYIVEDIYVSGVKEENITVV
GDATPQPSSVGRDYDTSKKITEQSDGLLAKVGMGMGSTVAVLFQSGRDTIDTVLKTILPF
MAFVSALIGIIMASGLGDWIAHGLAPLASHPLGLVMLALICSFPLLSPFLGPGAVIAQVI
GVLIGVQIGLGNIPPHLALPALFAINAQAACDFIPVGLSLAEARQDTVRVGVPSVLVSRF
LTGAPTVLIAWFVSGFIYQ
>P02942
MLKRIKIVTSLLLVLAVFGLLQLTSGGLFFNALKNDKENFTVLQTIRQQQSTLNGSWVAL
LQTRNTLNRAGIRYMMDQNNIGSGSTVAELMESASISLKQAEKNWADYEALPRDPRQSTA
AAAEIKRNYDIYHNALAELIQLLGAGKINEFFDQPTQGYQDGFEKQYVAYMEQNDRLHDI
AVSDNNASYSQAMWILVGVMIVVLAVIFAVWFGIKASLVAPMNRLIDSIRHIAGGDLVKP
IEVDGSNEMGQLAESLRHMQGELMRTVGDVRNGANAIYSGASEIATGNNDLSSRTEQQAA
SLEETAASMEQLTATVKQNAENARQASHLALSASETAQRGGKVVDNVVQTMRDISTSSQK
IADIISVIDGIAFQTNILALNAAVEAARAGEQGRGFAVVAGEVRNLAQRSAQAAREIKSL

IEDSVGKVDVGSTLVESAGETMAEIVSAVTRVTDIMGEIASASDEQSRGIDQVGLAVAEM
DRVTQQNAALVEESAAAAAALEEQASRLTEAVAVFRIQQQQRETSAVVKTVTPAAPRKMA
VADSEENWETF
>Q46909
MNTSPVRMDDLPLNRFHCRIAALTFGAHLTDGYVLGVIGYAIIQLTPAMQLTPFMAGMIG
GSALLGLFLGSLVLGWISDHIGRQKIFTFSFLLITLASFLQFFATTPEHLIGLRILIGIG
LGGDYSVGHTLLAEFSPRRHRGILLGAFSVVWTVGYVLASIAGHHFISENPEAWRWLLAS
AALPALLITLLRWGTPESPRWLLRQGRFAEAHAIVHRYFGPHVLLGDEVVTATHKHIKTL
FSSRYWRRTAFNSVFFVCLVIPWFVIYTWLPTIAQTIGLEDALTASLMLNALLIVGALLG
LVLTHLLAHRKFLLGSFLLLAATLVVMACLPSGSSLTLLLFVLFSTTISAVSNLVGILPA
ESFPTDIRSLGVGFATAMSRLGAAVSTGLLPWVLAQWGMQVTLLLLATVLLVGFVVTWLW
APETKALPLVAAGNVGGANEHSVSV
>P31064
MSWQQFKHAWLIKFWAPIPAVIAAGILSTYYFGITGTFWAVTGEFTRWGGQLLQLFGVHA
EEWGYFKIIHLEGSPLTRIDGMMILGMFGGCFAAALWANNVKLRMPRSRIRIMQAIIGGI
IAGFGARLAMGCNLAAFFTGIPQFSLHAWFFAIATAIGSWFGARFTLLPIFRIPVKMQKV
SAASPLTQKPDQARRRFRLGMLVFFGMLGWALLTAMNQPKLGLAMLFGVGFGLLIERAQI
CFTSAFRDMWITGRTHMAKAIIIGMAVSAIGIFSYVQLGVEPKIMWAGPNAVIGGLLFGF
GIVLAGGCETGWMYRAVEGQVHYWWVGLGNVIGSTILAYYWDDFAPALATDWDKINLLKT
FGPMGGLLVTYLLLFAALMLIIGWEKRFFRRAAPQTAKEIA
>P0AC41
MKLPVREFDAVVIGAGGAGMRAALQISQSGQTCALLSKVFPTRSHTVSAQGGITVALGNT
HEDNWEWHMYDTVKGSDYIGDQDAIEYMCKTGPEAILELEHMGLPFSRLDDGRIYQRPFG
GQSKNFGGEQAARTAAAADRTGHALLHTLYQQNLKNHTTIFSEWYALDLVKNQDGAVVGC
TALCIETGEVVYFKARATVLATGGAGRIYQSTTNAHINTGDGVGMAIRAGVPVQDMEMWQ
FHPTGIAGAGVLVTEGCRGEGGYLLNKHGERFMERYAPNAKDLAGRDVVARSIMIEIREG
RGCDGPWGPHAKLKLDHLGKEVLESRLPGILELSRTFAHVDPVKEPIPVIPTCHYMMGGI
PTKVTGQALTVNEKGEDVVVPGLFAVGEIACVSVHGANRLGGNSLLDLVVFGRAAGLHLQ
ESIAEQGALRDASESDVEASLDRLNRWNNNRNGEDPVAIRKALQECMQHNFSVFREGDAM
AKGLEQLKVIRERLKNARLDDTSSEFNTQRVECLELDNLMETAYATAVSANFRTESRGAH
SRFDFPDRDDENWLCHSLYLPESESMTRRSVNMEPKLRPAFPPKIRTY
>P0AB12
MRTVLNILNFVLGGFATTLGWLLATLVSIVLIFTLPLTRSCWEITKLSLVPYGNEAIHVD
ELNPAGKNVLLNTGGTVLNIFWLIFFGWWLCLMHIATGIAQCISIIGIPVGIANFKIAAI
ALWPVGRRVVSVETAQAAREANARRRFE
>P38055
MEQYDQIGARLDRLPLARFHYRIFGIISFSLLLTGFLSYSGNVVLAKLVSNGWSNNFLNA
AFTSALMFGYFIGSLTGGFIGDYFGRRRAFRINLLIVGIAATGAAFVPDMYWLIFFRFLM
GTGMGALIMVGYASFTEFIPATVRGKWSARLSFVGNWSPMLSAAIGVVVIAFFSWRIMFL
LGGIGILLAWFLSGKYFIESPRWLAGKGQIAGAECQLREVEQQIEREKSIRLPPLTSYQS
NSKVKVIKGTFWLLFKGEMLRRTLVAITVLIAMNISLYTITVWIPTIFVNSGIDVDKSIL
MTAVIMIGAPVGIFIAALIIDHFPRRLFGSTLLIIIAVLGYIYSIQTTEWAILIYGLVMI
FFLYMYVCFASAVYIPELWPTHLRLRGSGFVNAVGRIVAVFTPYGVAALLTHYGSITVFM
VLGVMLLLCALVLSIFGIETRKVSLEEISEVN

>P39196
MSESVHTNTSLWSKGMKAVIVAQFLSAFGDNALLFATLALLKAQFYPEWSQPILQMVFVG
AYILFAPFVGQVADSFAKGRVMMFANGLKLLGAASICFGINPFLGYTLVGVGAAAYSPAK
YGILGELTTGSKLVKANGLMEASTIAAILLGSVAGGVLADWHVLVALAACALAYGGAVVA
NIYIPKLAAARPGQSWNLINMTRSFLNACTSLWRNGETRFSLVGTSLFWGAGVTLRFLLV
LWVPVALGITDNATPTYLNAMVAIGIVVGAGAAAKLVTLETVSRCMPAGILIGVVVLIFS
LQHELLPAYALLMLIGVMGGFFVVPLNALLQERGKKSVGAGNAIAVQNLGENSAMLLMLG
IYSLAVMIGIPVVPIGIGFGALFALAITALWIWQRRH
>P61380
MSWIEPIISHFCQDLGVPTSSPLSPLIQLEMAQSGTLQLEQHGATLTLWLARSLAWHRCE
DAMVKALTLTAAQKSGALPLRAGWLGESQLVLFVSLDERSLTLPLLHQAFEQLLRLQQEV
LAP
>P0AFN6
MMHLYWVALKSIWAKEIHRFMRIWVQTLVPPVITMTLYFIIFGNLIGSRIGDMHGFSYMQ
FIVPGLIMMSVITNAYANVASSFFGAKFQRNIEELLVAPVPTHVIIAGYVGGGVARGLFV
GILVTAISLFFVPFQVHSWVFVALTLVLTAVLFSLAGLLNGVFAKTFDDISLVPTFVLTP
LTYLGGVFYSLTLLPPFWQGLSHLNPIVYMISGFRYGFLGINDVPLVTTFGVLVVFIVAF
YLICWSLIQRGRGLRS
>P32705
MKRVLTALAATLPFAANAADAISGAVERQPTNWQAIIMFLIFVVFTLGITYWASKRVRSR
SDYYTAGGNITGFQNGLAIAGDYMSAASFLGISALVFTSGYDGLIYSLGFLVGWPIILFL
IAERLRNLGRYTFADVASYRLKQGPIRILSACGSLVVVALYLIAQMVGAGKLIELLFGLN
YHIAVVLVGVLMMMYVLFGGMLATTWVQIIKAVLLLFGASFMAFMVMKHVGFSFNNLFSE
AMAVHPKGVDIMKPGGLVKDPISALSLGLGLMFGTAGLPHILMRFFTVSDAREARKSVFY
ATGFMGYFYILTFIIGFGAIMLVGANPEYKDAAGHLIGGNNMAAVHLANAVGGNLFLGFI
SAVAFATILAVVAGLTLAGASAVSHDLYANVFKKGATEREELRVSKITVLILGVIAIILG
VLFENQNIAFMVGLAFAIAASCNFPIILLSMYWSKLTTRGAMMGGWLGLITAVVLMILGP
TIWVQILGHEKAIFPYEYPALFSITVAFLGIWFFSATDNSAEGARERELFRAQFIRSQTG
FGVEQGRAH
>P0AE74
MSLAKDNIWKLLAPLVVMGVMFLIPVPDGMPPQAWHYFAVFVAMIVGMILEPIPATAISF
IAVTICVIGSNYLLFDAKELADPAFNAQKQALKWGLAGFSSTTVWLVFGAFIFALGYEVS
GLGRRIALFLVKFMGKRTLTLGYAIVIIDILLAPFTPSNTARTGGTVFPVIKNLPPLFKS
FPNDPSARRIGGYLMWMMVISTSLSSSMFVTGAAPNVLGLEFVSKIAGIQISWLQWFLCF
LPVGVILLIIAPWLSYVLYKPEITHSEEVATWAGDELKTMGALTRREWTLIGLVLLSLGL
WVFGSEVINATAVGLLAVSLMLALHVVPWKDITRYNSAWNTLVNLATLVVMANGLTRSGF
IDWFAGTMSTHLEGFSPNATVIVLVLVFYFAHYLFASLSAHTATMLPVILAVGKGIPGVP
MEQLCILLVLSIGIMGCLTPYATGPGVIIYGCGYVKSKDYWRLGAIFGVIYISMLLLVGW
PILAMWN
>P45394
MLLATALLIVGLLLVVYSADRLVFAASILCRTFGIPPLIIGMTVVSIGTSLPEVIVSLAA
SLHEQRDLAVGTALGSNIINILLILGLAALVRPFTVHSDVLRRELPLMLLVSVVAGSVLY
DGQLSRSDGIFLLFLAVLWLLFIVKLARQAERQGTDSLTREQLAELPRDGGLPVAFLWLG
IALIIMPVATRMVVDNATVLANYFAISELTMGLTAIAIGTSLPELATAIAGVRKGENDIA

VGNIIGANIFNIVIVLGLPALITPGEIDPLAYSRDYSVMLLVSIIFALLCWRRSPQPGRG
VGVLLTGGFIVWLAMLYWLSPILVE
>P33226
MRKLWNALRRPSARWSVLALVAIGIVIGIALIVLPHVGIKVTSTTEFCVSCHSMQPVYEE
YKQSVHFQNASGVRAECHDCHIPPDIPGMVKRKLEASNDIYQTFIAHSIDTPEKFEAKRA
ELAEREWARMKENNSATCRSCHNYDAMDHAKQHPEAARQMKVAAKDNQSCIDCHKGIAHQ
LPDMSSGFRKQFDELRASANDSGDTLYSIDIKPIYAAKGDKEASGSLLPASEVKVLKRDG
DWLQIEITGWTESAGRQRVLTQFPGKRIFVASIRGDVQQQVKTLEKTTVADTNTEWSKLQ
ATAWMKKGDMVNDIKPIWAYADSLYNGTCNQCHGAPEIAHFDANGWIGTLNGMIGFTSLD
KREERTLLKYLQMNASDTAGKAHGDKKEEK
>Q06910
MKPPMDLDSLLTQTPAKDNAALERVLAAARGELALRRPVRRWRTQAVGLMAASAGLGLLA
AVVLLAVGAVTGPLLLARAPLLAMLVGTSAVCAWGALSPKGRWMRRLGVGLAVVSAAALV
LARGAPHSPPSFPGWVCTVSHLAIGVVPLVVALFALRGAFFQPLRAVVAGLSVGSTGALL
GELACEQDWRHVLSHHLLAWVVITVVLVVISKSLKPRSYAP
>P32135
MLTKKKWALFSLLTLCGGTIYKLPSLKDAFYIPMQEYFHLTNGQIGNAMSVNSFVTTVGF
FLSIYFADKLPRRYTMSFSLIATGLLGVYLTTMPGYWGILFVWALFGVTCDMMNWPVLLK
SVSRLGNSEQQGRLFGFFETGRGIVDTVVAFSALAVFTWFGSGLLGFKAGIWFYSLIVIA
VGIIIFFVLNDKEEAPSVEVKKEDGASKNTSMTSVLKDKTIWLIAFNVFFVYAVYCGLTF
FIPFLKNIYLLPVALVGAYGIINQYCLKMIGGPIGGMISDKILKSPSKYLCYTFIISTAA
LVLLIMLPHESMPVYLGMACTLGFGAIVFTQRAVFFAPIGEAKIAENKTGAAMALGSFIG
YAPAMFCFSLYGYILDLNPGIIGYKIVFGIMACFAFSGAVVSVMLVKRISQRKKEMLAAE
A
>P27125
MSNAITMGIFWHLIGAASAACFYAPFKKVKKWSWETMWSVGGIVSWIILPWAISALLLPN
FWAYYSSFSLSTRLPVFLFGAMWGIGNINYGLTMRYLGMSMGIGIAIGITLIVGTLMTPI
INGNFDVLISTEGGRMTLLGVLVALIGVGIVTRAGQLKERKMGIKAEEFNLKKGLVLAVM
CGIFSAGMSFAMNAAKPMHEAAAALGVDPLYVALPSYVVIMGGGAIINLGFCFIRLAKVK
DLSLKADFSLAKSLIIHNVLLSTLGGLMWYLQFFFYAWGHARIPAQYDYISWMLHMSFYV
LCGGIVGLVLKEWNNAGRRPVTVLSLGCVVIIVAANIVGIGMAN
>P36646
MASKQLWRWHGITGDGNAQDGMLWAESRTLLLMALQQQMVTPLSLKRIAINSAQWRGDKS
AEVIHQLATLLKAGLTLSEGLALLAEQHPSKQWQALLQSLAHDLEQGIAFSNALLPWSEV
FPPLYQAMIRTGELTGKLDECCFELARQQKAQRQLTDKVKSALRYPIIILAMAIMVVVAM
LHFVLPEFAAIYKTFNTPLPALTQGIMTLADFSGEWSWLLVLFGFLLAIANKLLMRRPTW
LIVRQKLLLRIPIMGSLMRGQKLTQIFTILALTQSAGITFLQGVESVRETMRCPYWVQLL
TQIQHDISNGQPIWLALKNTGEFSPLCLQLVRTGEASGSLDLMLDNLAHHHRENTMALAD
NLAALLEPALLIITGGIIGTLVVAMYLPIFHLGDAMSGMG
>P0ADR0
MSEALSLFSLFASSFLSATLLPGNSEVVLVAMLLSGISHPWVLVLTATMGNSLGGLTNVI
LGRFFPLRKTSRWQEKATGWLKRYGAVTLLLSWMPVVGDLLCLLAGWMRISWGPVIFFLC
LGKALRYVAVAAATVQGMMWWH
>P42590

MSDTKRNTIGKFGLLSLTFAAVYSFNNVINNNIELGLASAPMFFLATIFYFIPFCLIIAE
FVSLNKNSEAGVYAWVKSSLGGRWAFITAYTYWFVNLFFFTSLLPRVIAYASYAFLGYEY
IMTPVATTIISMVLFAFSTWVSTNGAKMLGPITSVTSTLMLLLTLSYILLAGTALVGGVQ
PADAITVDAMIPNFNWAFLGVTTWIFMAAGGAESVAVYVNDVKGGSKSFVKVIILAGIFI
GVLYSVSSVLINVFVSSKELKFTGGSVQVFHGMAAYFGLPEALMNRFVGLVSFTAMFGSL
LMWTATPVKIFFSEIPEGIFGKKTVELNENGVPARAAWIQFLIVIPLMIIPMLGSNTVQD
LMNTIINMTAAASMLPPLFIMLAYLNLRAKLDHLPRDFRMGSRRTGIIVVSMLIAIFAVG
FVASTFPTGANILTIIFYNVGGIVIFLGFAWWKYSKYIKGLTAEERHIEATPASNVD
>P0AEK4
MGFLSGKRILVTGVASKLSIAYGIAQAMHREGAELAFTYQNDKLKGRVEEFAAQLGSDIV
LQCDVAEDASIDTMFAELGKVWPKFDGFVHSIGFAPGDQLDGDYVNAVTREGFKIAHDIS
SYSFVAMAKACRSMLNPGSALLTLSYLGAERAIPNYNVMGLAKASLEANVRYMANAMGPE
GVRVNAISAGPIRTLAASGIKDFRKMLAHCEAVTPIRRTVTIEDVGNSAAFLCSDLSAGI
SGEVVHVDGGFSIAAMNELELK
>P76103
MRLFSIPPPTLLAGFLAVLIGYASSAAIIWQAAIVAGATTAQISGWMTALGLAMGVSTLT
LTLWYRVPVLTAWSTPGAALLVTGLQGLTLNEAIGVFIVTNALIVLCGITGLFARLMRII
PHSLAAAMLAGILLRFGLQAFASLDGQFTLCGSMLLVWLATKAVAPRYAVIAAMIIGIVI
VIAQGDVVTTDVVFKPVLPTYITPDFSFAHSLSVALPLFLVTMASQNAPGIAAMKAAGYS
APVSPLIVFTGLLALVFSPFGVYSVGIAAITAAICQSPEAHPDKDQRWLAAAVAGIFYLL
AGLFGSAITGMMAALPVSWIQMLAGLALLSTIGGSLYQALHNERERDAAVVAFLVTASGL
TLVGIGSAFWGLIAGGVCYVVLNLIADRNRY
>P31436
MQKTATTPSKILDLTAAAFLLVAFLTGIAGALQTPTLSIFLADELKARPIMVGFFFTGSA
IMGILVSQFLARHSDKQGDRKLLILLCCLFGVLACTLFAWNRNYFILLSTGVLLSSFAST
ANPQMFALAREHADRTGRETVMFSTFLRAQISLAWVIGPPLAYELAMGFSFKVMYLTAAI
AFVVCGLIVWLFLPSIQRNIPVVTQPVEILPSTHRKRDTRLLFVVCSMMWAANNLYMINM
PLFIIDELHLTDKLTGEMIGIAAGLEIPMMLIAGYYMKRIGKRLLMLIAIVSGMCFYASV
LMATTPAVELELQILNAIFLGILCGIGMLYFQDLMPEKIGSATTLYANTSRVGWIIAGSV
DGIMVEIWSYHALFWLAIGMLGIAMICLLFIKDI
>P0AAE5
MEKKLGLSALTALVLSSMLGAGVFSLPQNMAAVASPAALLIGWGITGAGILLLAFAMLIL
TRIRPELDGGIFTYAREGFGELIGFCSAWGYWLCAVIANVSYLVIVFSALSFFTDTPELR
LFGDGNTWQSIVGASALLWIVHFLILRGVQTAASINLVATLAKLLPLGLFVVLAMMMFKL
DTFKLDFTGLALGVPVWEQVKNTMLITLWVFIGVEGAVVVSARARNKRDVGKATLLAVLS
ALGVYLLVTLLSLGVVARPELAEIRNPSMAGLMVEMMGPWGEIIIAAGLIVSVCGAYLSW
TIMAAEVPFLAATHKAFPRIFARQNAQAAPSASLWLTNICVQICLVLIWLTGSDYNTLLT
IASEMILVPYFLVGAFLLKIATRPLHKAVGVGACIYGLWLLYASGPMHLLLSVVLYAPGL
LVFLYARKTHTHDNVLNRQEMVLIGMLLIASVPATWMLVG
>P32166
MTEQQISRTQAWLESLRPKTLPLAFAAIIVGTALAWWQGHFDPLVALLALITAGLLQILS
NLANDYGDAVKGSDKPDRIGPLRGMQKGVITQQEMKRALIITVVLICLSGLALVAVACHT
LADFVGFLILGGLSIIAAITYTVGNRPYGYIGLGDISVLVFFGWLSVMGSWYLQAHTLIP
ALILPATACGLLATAVLNINNLRDINSDRENGKNTLVVRLGEVNARRYHACLLMGSLVCL

ALFNLFSLHSLWGWLFLLAAPLLVKQARYVMREMDPVAMRPMLERTVKGALLTNLLFVLG
IFLSQWAA
>P71238
MSTSIRICSYLLLPLIYLLVNVKIAQLGESFPITIVTFLPVLLLLFLERISVKKLMIALG
IGAGLTAFNYLFGQSLDASKYVTSTMLFVYIVIIIGMVWSIRFKTISPHNHRKILRFFYL
VVGLVVALAAVEMAQIILTGGSSIMESISKYLIYSNSYVLNFIKFGGKRTTALYFEPAFF
ALALISIWLSIKQFGIKTPKTDAMILAGIILSGSFSGVMTFILFYLLEWAFQYLNKEAIK
KKLPLALISLAVFLVGVVIAFPYISTRLGDLGTEGSSSYYRIVGPLVMVGYSLTHIDGVV
RFGSLYEYVASFGIFNGADVGKTIDNGLYLLIIYFSWFAVFLSLWYMGKVIKMMINAFGD
NRNFRVQLYLFTPVSLFFTGSIFSPEYAFLIVCPFILRKALNITR
>P28303
MPPGVAVCFSSLFIRLVCMAFLTSSDKALWHLALPMIFSNITVPLLGLVDTAVIGHLDSP
VYLGGVAVGATATSFLFMLLLFLRMSTTGLTAQAYGAKNPQALARTLVQPLLLALGAGAL
IALLRTPIIDLALHIVGGSEAVLEQARRFLEIRWLSAPASLANLVLLGWLLGVQYARAPV
ILLVVGNILNIVLDVWLVMGLHMNVQGAALATVIAEYATLLIGLLMVRKILKLRGISGEM
LKTAWRGNFRRLLALNRDIMLRSLLLQLCFGAITVLGARLGSDIIAVNAVLMTLLTFTAY
ALDGFAYAVEAHSGQAYGARDGSQLLDVWRAACRQSGIVALLFSVVYLLAGEHIIALLTS
LTQIQQLADRYLIWQVILPVVGVWCYLLDGMFIGATRATEMRNSMAVAAAGFALTLLTLP
WLGNHALWLALTVFLALRGLSLAAIWRRHWRNGTWFAAT
>P0AAR5
MQRIILIIIGWLAVVLGTLGVVLPVLPTTPFILLAAWCFARSSPRFHAWLLYRSWFGSYL
RFWQKHHAMPRGVKPRAILLILLTFAISLWFVQMPWVRIMLLVILACLLFYMWRIPVIDE
KQEKH
>P33915
MSRLSPVNQARWARFRHNRRGYWSLWIFLVLFGLSLCSELIANDKPLLVRYDGSWYFPLL
KNYSESDFGGPLASQADYQDPWLKQRLENNGWVLWAPIRFGATSINFATNKPFPSPPSRQ
NWLGTDANGGDVLARILYGTRISVLFGLMLTLCSSVMGVLAGALQGYYGGKVDLWGQRFI
EVWSGMPTLFLIIILLSSVVQPNFWWLLAITVLFGWMSLVGVVRAEFLRTRNFDYIRAAQA
LGVSDRSIILRHMLPNAMVATLTFLPFILCSSITTLTSLDFLGFGLPLGSPSLGELLLQG
KNNLQAPWLGITAFLSVAILLSLLIFIGEAVRDAFDPNKAV
>P0AAG5
MRSFSQLWPTLKRLLAYGSPWRKPLGIAVLMMWVAAAAEVSGPLLISYFIDNMVAKNNLP
LKVVAGLAAAYVGLQLFAAGLHYAQSLLFNRAAVGVVQQLRTDVMDAALRQPLSEFDTQP
VGQVISRVTNDTEVIRDLYVTVVATVLRSAALVGAMLVAMFSLDWRMALVAIMIFPVVLV
VMVIYQRYSTPIVRRVRAYLADINDGFNEIINGMSVIQQFRQQARFGERMGEASRSHYMA
RMQTLRLDGFLLRPLLSLFSSLILCGLLMLFGFSASGTIEVGVLYAFISYLGRLNEPLIE
LTTQQAMLQQAVVAGERVFELMDGPRQQYGNDDRPLQSGTIEVDNVSFAYRDDNLVLKNI
NLSVPSRNFVALVGHTGSGKSTLASLLMGYYPLTEGEIRLDGRPLSSLSHSALRQGVAMV
QQDPVVLADTFLANVTLGRDISEERVWQALETVQLAELARSMSDGIYTPLGEQGNNLSVG
QKQLLALARVLVETPQILILDEATASIDSGTEQAIQHALAAVREHTTLVVIAHRLSTIVD
ADTILVLHRGQAVEQGTHQQLLAAQGRYWQMYQLQLAGEELAASVREEESLSA
>P64515
MVSALYAVLSALLLMKFSFDVVRLRMQYRVAYGDGGFSELQSAIRIHGNAVEYIPIAIVL
MLFMEMNGAETWMVHICGIVLLAGRLMHYYGFHHRLFRWRRSGMSATWCALLLMVLANLW

YMPWELVFSLR
>P56427
MAKKNKPTECPAGEKWAVPYADFLSLLLALFIALYAISAVNKSKVEALKTEFIKIFNYAP
KPEAMQPVVVIPPDSGKEEEQMASESSKPASQNTETKATIARKGEGSVLEQIDQGSILKL
PSNLLFENATSDAINQDMMLYIERIAKIIQKLPKRVHINVRGFTDDTPLVKTRFKSHYEL
AANRAYRVMKVLIQYGVNPNQLSFSSYGSTNPIAPNDSLENRMKNNRVEIFFSTDANDLS
KIHSILDNEFNPHKQQE
>P37621
MPEPVAEPALNGLRLNLRIVSIVMFNFASYLTIGLPLAVLPGYVHDVMGFSAFWAGLVIS
LQYFATLLSRPHAGRYADSLGPKKIVVFGLCGCFLSGLGYLTAGLTASLPVISLLLLCLG
RVILGIGQSFAGTGSTLWGVGVVGSLHIGRVISWNGIVTYGAMAMGAPLGVVFYHWGGLQ
ALALIIMGVALVAILLAIPRPTVKASKGKPLPFRAVLGRVWLYGMALALASAGFGVIATF
ITLFYDAKGWDGAAFALTLFSCAFVGTRLLFPNGINRIGGLNVAMICFSVEIIGLLLVGV
ATMPWMAKIGVLLAGAGFSLVFPALGVVAVKAVPQQNQGAALATYTVFMDLSLGVTGPLA
GLVMSWAGVPVIYLAAAGLVAIALLLTWRLKKRPPEHVPEAASSS
>P76630
MESPTPQPAPGSATFMEGCKDSLPIVISYIPVAFAFGLNATRLGFSPLESVFFSCIIYAG
ASQFVITAMLAAGSSLWIAALTVMAMDVRHVLYGPSLRSRIIQRLQKSKTALWAFGLTDE
VFAAATAKLVRNNRRWSENWMIGIAFSSWSSWVFGTVIGAFSGSGLLQGYPAVEAALGFM
LPALFMSFLLASFQRKQSLCVTAALVGALAGVTLFSIPVAILAGIVCGCLTALIQAFWQG
APDEL
>P76470
MSTALLDAVVKKNRVRLIPFMLALYVLAFLDRSNIGFAKQTYQIDTGLSNEAYALGAGIF
FVVYAFLGVPANLLMRKLGARTWIGTTTLLWGFLSAAMAWADTEAKFLIVRTLLRAAEAG
FFPGMIYLTSQWFPQRNRASIMGLFYMGAPLALTLGSPLSGALLEMHGFMGHPGWFWMFV
IEGLLAVGAGVFTFFWLDDTPEQARFLSKQEKTLLINQLASEEQQKVTSRLSDALRNGRV
WQLAIIYLTIQVAVYGLIFFLPTQVAALLGTKVGFTASVVTAIPWVAALFGTWLIPRYSD
KTGERRNVAALTLLAAGIGIGLSGLLSPVMAIVALCVAAIGFIAVQPVFWTMPTQLLSGT
ALAAGIGFVNLFGAVGGFIAPILRVKAETLFASDAAGLLTLAAVAVIGSLIIFTLRVNRT
VAQTDVAHH
>P0A712
MQIKRSIEKIPGGMMLVPLFLGALCHTFSPGAGKYFGSFTNGMITGTVPILAVWFFCMGA
SIKLSATGTVLRKSGTLVVTKIAVAWVVAAIASRIIPEHGVEVGFFAGLSTLALVAAMDM
TNGGLYASIMQQYGTKEEAGAFVLMSLESGPLMTMIILGTAGIASFEPHVFVGAVLPFLV
GFALGNLDPELREFFSKAVQTLIPFFAFALGNTIDLTVIAQTGLLGILLGVAVIIVTGIP
LIIADKLIGGGDGTAGIAASSSAGAAVATPVLIAEMVPAFKPMAPAATSLVATAVIVTSI
LVPILTSIWSRKVKARAAKIEILGTVK
>P37669
MQPKIYWIDNLRGIACLMVVMIHTTTWYVTNAHSVSPVTWDIANVLNSASRVSVPLFFMI
SGYLFFGERSAQPRHFLRIGLCLIFYSAIALLYIALFTSINMELALKNLLQKPVFYHLWF
FFAIAVIYLVSPLIQVKNVGGKMLLVLMAVIGIIANPNTVPQKIDGFEWLPINLYINGDT
FYYILYGMLGRAIGMMDTQHKALSWVSAALFATGVFIISRGTLYELQWRGNFADTWYLYC
GPMVFICAIALLTLVKNTLDTRTIRGLGLISRHSLGIYGFHALIIHALRTRGIELKNWPI
LDIIWIFCATLAASLLLSMLVQRIDRNRLVS

>P69853
MTHFSQQDNFSVAARVLGALFYYAPESAEAAPLVAVLTSDGWETQWPLPEASLAPLVTAF
QTQCEETHAQAWQRLFVGPWALPSPPWGSVWLDRESVLFGDSTLALRQWMREKGIQFEMK
QNEPEDHFGSLLLMAAWLAENGRQTECEELLAWHLFPWSTRFLDVFIEKAEHPFYRALGE
LARLTLAQWQSQLLIPVAVKPLFR
>P72292
MRGQRRWAHPFTLIRRLFGNAVFSSLTRRIVFFNLVALVVLVGGIMYLNQFREGLIDARV
ESLLTQGEIIAGAISASASVDTNSITIDPEKLLELQAGESITPLPSDEDLEFPIIQERVA
PVLRRLISPTRTRARLFDADADLLLDSRHLYSGGQVLRFDLPPVDPESPSLADEFGTWFN
RLLQPGDLPLYKEPPGGNGSIYPEVMNALTGVRGAVVRVTEKGELIVSVAVPVQRFRAVL
GVLLLSTQAGDIDKIVHAERLAIIRVFGVAALVNVILSLLLSSTIANPLRRLSAAAIRVR
RGGAKEREEIPDFSSRQDEIGNLSVALREMTTALYDRIAAIENFAADVSHELKNPLTSLR
SAVETLPLARNEESKKRLMDVIQHDVRRLDRLISDISDASRLDAELARADAKKVDLEKLL
GDLVEISRQIRGSKKPVLLDFVVDRKDNPRASFIVSGYELRIGQIITNLIENARSFVPEQ
NGRIVVRLTRSRLRCIVYVEDNGPGIQAEDIDRIFERFYTDRPEGEDFGQNSGLGLSISR
QIAEAHGGTLRAENIAGKDGRISGARFVLSLPAGPHP
>P27243
MLTSFKLHSLKPYTLKSSMILEIITYILCFFSMIIAFVDNTFSIKIYNITAIVCLLSLIL
RGRQENYNIKNLILPLSIFLIGLLDLIWYSAFKVDNSPFRATYHSYLNTAKIFIFGSFIV
FLTLTSQLKSKKESVLYTLYSLSFLIAGYAMYINSIHENDRISFGVGTATGAAYSTMLIG
IVSGVAILYTKKNHPFLFLLNSCAVLYVLALTQTRATLLLFPIICVAALIAYYNKSPKKF
TSSIVLLIAILASIVIIFNKPIQNRYNEALNDLNSYTNANSVTSLGARLAMYEIGLNIFI
KSPFSFRSAESRAESMNLLVAEHNRLRGALEFSNVHLHNEIIEAGSLKGLMGIFSTLFLY
FSLFYIAYKKRALGLLILTLGIVGIGLSDVIIWARSIPIIIISAIVLLLVINNRNNTIN
>P0AEJ0
MQQQKPLEGAQLVIMTIALSLATFMQVLDSTIANVAIPTIAGNLGSSLSQGTWVITSFGV
ANAISIPLTGWLAKRVGEVKLFLWSTIAFAIASWACGVSSSLNMLIFFRVIQGIVAGPLI
PLSQSLLLNNYPPAKRSIALALWSMTVIVAPICGPILGGYISDNYHWGWIFFINVPIGVA
VVLMTLQTLRGRETRTERRRIDAVGLALLVIGIGSLQIMLDRGKELDWFSSQEIIILTVV
AVVAICFLIVWELTDDNPIVDLSLFKSRNFTIGCLCISLAYMLYFGAIVLLPQLLQEVYG
YTATWAGLASAPVGIIPVILSPIIGRFAHKLDMRRLVTFSFIMYAVCFYWRAYTFEPGMD
FGASAWPQFIQGFAVACFFMPLTTITLSGLPPERLAAASSLSNFTRTLAGSIGTSITTTM
WTNRESMHHAQLTESVNPFNPNAQAMYSQLEGLGMTQQQASGWIAQQITNQGLIISANEI
FWMSAGIFLVLLGLVWFAKPPFGAGGGGGGAH
>P04140
MSEPKTGRGALFTGGLAAILASACCLGPLVLIALGFSGAWIGNLAVLEPYRPIFIGVALV
ALFFAWRRIYRQAAACKPGEVCAIPQVRATYKLIFWIVAALVLVALGFPYVMPFFY
>P0AFS7
MVNVRQPRDVAQILLSVLFLAIMIVACLWIVQPFILGFAWAGTVVIATWPVLLRLQKIMF
GRRSLAVLVMTLLLVMVFIIPIALLVNSIVDGSGPLIKAISSGDMTLPDLAWLNTIPVIG
AKLYAGWHNLLDMGGTAIMAKVRPYIGTTTTWFVGQAAHIGRFMVHCALMLLFSALLYWR
GEQVAQGIRHFATRLAGVRGDAAVLLAAQAIRAVALGVVVTALVQAVLGGIGLAVSGVPY
ATLLTVLMILSCLVQLGPLPVLIPAIIWLYWTGDTTWGTVLLVWSGVVGTLDNVIRPMLI
RMGADLPLILILSGVIGGLIAFGMIGLFIGPVLLAVSWRLFAAWVEEVPPPTDQPEEILE

ELGEIEKPNK
>P0AG90
MLNRYPLWKYVMLIVVIVIGLLYALPNLFGEDPAVQITGARGVAASEQTLIQVQKTLQEE
KITAKSVALEEGAILARFDSTDTQLRAREALMGVMGDKYVVALNLAPATPRWLAAIHAEP
MKLGLDLRGGVHFLMEVDMDTALGKLQEQNIDSLRSDLREKGIPYTTVRKENNYGLSITF
RDAKARDEAIAYLSKRHPDLVISSQGSNQLRAVMSDARLSEAREYAVQQNINILRNRVNQ
LGVAEPVVQRQGADRIVVELPGIQDTARAKEILGATATLEFRLVNTNVDQAAAASGRVPG
DSEVKQTREGQPVVLYKRVILTGDHITDSTSSQDEYNQPQVNISLDSAGGNIMSNFTKDN
IGKPMATLFVEYKDSGKKDANGRAVLVKQEEVINIANIQSRLGNSFRITGINNPNEARQL
SLLLRAGALIAPIQIVEERTIGPTLGMQNIEQGLEACLAGLLVSILFMIIFYKKFGLIAT
SALIANLILIVGIMSLLPGATLSMPGIAGIVLTLAVAVDANVLINERIKEELSNGRTVQQ
AIDEGYRGAFSSIFDANITTLIKVIILYAVGTGAIKGFAITTGIGVATSMFTAIVGTRAI
VNLLYGGKRVKKLSI
>P46139
MMDNDNSLNKRPTFKRALRNISMTSIFITMMLIWLLLSVTSVLTLKQYAQKNLALTAATM
TYSLEAAVVFADGPAATETLAALGQQGQFSTAEVRDKQQNILASWHYTRKDPGDTFSNFI
SHWLFPAPIIQPIRHNGETIGEVRLTARDSSISHFIWFSLAVLTGCILLASGIAITLTRH
LHNGLVEALKNITDVVHDVRSNRNFSRRVSEERIAEFHRFALDFNSLLDEMEEWQLRLQA
KNAQLLRTALHDPLTGLANRAAFRSGINTLMNNSDARKTSALLFLDGDNFKYINDTWGHA
TGDRVLIEIAKRLAEFGGLRHKAYRLGGDEFAMVLYDVQSESEVQQICSALTQIFNLPFD
LHNGHQTTMTLSIGYAMTIEHASAEKLQELADHNMYQAKHQRAEKLVR
>P33021
MDVMRSVLGMVVLLTIAFLLSVNKKKISLRTVGAALVLQVVIGGIMLWLPPGRWVAEKVA
FGVHKVMAYSDAGSAFIFGSLVGPKMDTLFDGAGFIFGFRVLPAIIFVTALVSILYYIGV
MGILIRILGGIFQKALNISKIESFVAVTTIFLGQNEIPAIVKPFIDRLNRNELFTAICSG
MASIAGSTMIGYAALGVPVEYLLAASLMAIPGGILFARLLSPATESSQVSFNNLSFTETP
PKSIIEAAATGAMTGLKIAAGVATVVMAFVAIIALINGIIGGVGGWFGFEHASLESILGY
LLAPLAWVMGVDWSDANLAGSLIGQKLAINEFVAYLNFSPYLQTAGTLDAKTVAIISFAL
CGFANFGSIGVVVGAFSAVAPHRAPEIAQLGLRALAAATLSNLMSATIAGFFIGLA
>P64439
MKHKQRWAGAICCFVLFIVVCLFLATHMKGAFRAAGHPEIGLLFFILPGAVASFFSQRRE
VLKPLFGAMLAAPCSMLIMRLFFSPTRSFWQELAWLLSAVFWCALGALCFLFISSLFKPQ
HRKNQ
>P05701
MMNEHSIDTDNRKANNALYLFIIIGLIPLLCIFVVYYKTPDALLLRKIATSTENLPSITS
SYNPLMTKVMDIYCKTAPFLALILYILTFKIRKLINNTDRNTVLRSCLLSPLVYAAIVYL
FCFRNFELTTAGRPVRLMATNDATLLLFYIGLYSIIFFTTYITLFTPVTAFKLLKKRQ
>P0A2R8
MLSAFQLEKNRLTRLEVEESQSLIDAVWVDLVEPDDDERLRVQSELGQSLATRPELEDIE
ASARFFEDEDGLHIHSFFFFEDAEDHAGNSTVAFTIRDGRLFTLRERELPAFRLYRMRAR
SQAMVDGNAYELLLDLFETKIEQLADEIENIYSDLEKLSRVIMEGHQGDEYDEALSTLAE
LEDIGWKVRLCLMDTQRALNFLVRKARLPGGQLEQAREILRDIESLLPHNESLFQKVNFL
MQAAMGFINIEQNRIIKIFSVVSVVFLPPTLVASSYGMNFEFMPELKWSFGYPGAIIFMI
LAGLAPYLYFKRKNWL

>P46125
MLLAGSSLLTLLDDIATLLDDISVMGKLAAKKTAGVLGDDLSLNAQQVSGVRANRELPVV
WGVAKGSLINKVILVPLALIISAFIPWAITPLLMIGGAFLCFEGVEKVLHMLEARKHKED
PAQSQQRLEKLAAQDPLKFEKDKIKGAIRTDFILSAEIVAITLGIVAEAPLLNQVLVLSG
IALVVTVGVYGLVGVIVKIDDLGYWLAEKSSALMQALGKGLLIIAPWLMKALSIVGTLAM
FLVGGGIVVHGIAPLHHAIEHFAGQQSAVVAMILPTVLNLILGFIIGGIVVLGVKAVEKM
RGQAH
>P22729
MKPMHIAMALLSAAMFFVLAGVFMGVQLELDGTKLVVDTASDVRWQWVFIGTAVVFFFQL
LRPAFQKGLKSVSGPKFILPAIDGSTVKQKLFLVALLVLAVAWPFMVSRGTVDIATLTMI
YIILGLGLNVVVGLSGLLVLGYGGFYAIGAYTFALLNHYYGLGFWTCLPIAGLMAAAAGF
LLGFPVLRLRGDYLAIVTLGFGEIVRILLLNNTEITGGPNGISQIPKPTLFGLEFSRTAR
EGGWDTFSNFFGLKYDPSDRVIFLYLVALLLVVLSLFVINRLLRMPLGRAWEALREDEIA
CRSLGLSPRRIKLTAFTISAAFAGFAGTLFAARQGFVSPESFTFAESAFVLAIVVLGGMG
SQFAVILAAILLVVSRELMRDFNEYSMLMLGGLMVLMMIWRPQGLLPMTRPQLKLKNGAA
KGEQA
>P69831
MFSEVMRYILDLGPTVMLPIVIIIFSKILGMKAGDCFKAGLHIGIGFVGIGLVIGLMLDS
IGPAAKAMAENFDLNLHVVDVGWPGSSPMTWASQIALVAIPIAILVNVAMLLTRMTRVVN
VDIWNIWHMTFTGALLHLATGSWMIGMAGVVIHAAFVYKLGDWFARDTRNFFELEGIAIP
HGTSAYMGPIAVLVDAIIEKIPGVNRIKFSADDIQRKFGPFGEPVTVGFVMGLIIGILAG
YDVKGVLQLAVKTAAVMLLMPRVIKPIMDGLTPIAKQARSRLQAKFGGQEFLIGLDPALL
LGHTAVVSASLIFIPLTILIAVCVPGNQVLPFGDLATIGFFVAMAVAVHRGNLFRTLISG
VIIMSITLWIATQTIGLHTQLAANAGALKAGGMVASMDQGGSPITWLLIQVFSPQNIPGF
IIIGAIYLTGIFMTWRRARGFIKQEKVVLAE
>P23830
MLSKFKRNKHQQHLAQLPKISQSVDDVDFFYAPADFRETLLEKIASAKQRICIVALYLEQ
DDGGKGILNALYEAKRQRPELDVRVLVDWHRAQRGRIGAAASNTNADWYCRMAQENPGVD
VPVYGVPINTREALGVLHFKGFIIDDSVLYSGASLNDVYLHQHDKYRYDRYHLIRNRKMS
DIMFEWVTQNIMNGRGVNRLDDVNRPKSPEIKNDIRLFRQELRDAAYHFQGDADNDQLSV
TPLVGLGKSSLLNKTIFHLMPCAEQKLTICTPYFNLPAILVRNIIQLLREGKKVEIIVGD
KTANDFYIPEDEPFKIIGALPYLYEINLRRFLSRLQYYVNTDQLVVRLWKDDDNTYHLKG
MWVDDKWMLITGNNLNPRAWRLDLENAILIHDPQLELAPQREKELELIREHTTIVKHYRD
LQSIADYPVKVRKLIRRLRRIRIDRLISRIL
>P63235
MATSVQTGKAKQLTLLGFFAITASMVMAVYEYPTFATSGFSLVFFLLLGGILWFIPVGLC
AAEMATVDGWEEGGVFAWVSNTLGPRWGFAAISFGYLQIAIGFIPMLYFVLGALSYILKW
PALNEDPITKTIAALIIILWALALTQFGGTKYTARIAKVGFFAGILLPAFILIALAAIYLH
SGAPVAIEMDSKTFFPDFSKVGTLVVFVAFILSYMGVEASATHVNEMSNPGRDYPLAMLL
LMVAAICLSSVGGLSIAMVIPGNEINLSAGVMQTFTVLMSHVAPEIEWTVRVISALLLLG
VLAEIASWIVGPSRGMYVTAQKNLLPAAFAKMNKNGVPVTLVISQLVITSIALIILTNTG
GGNNMSFLIALALTVVIYLCAYFMLFIGYIVLVLKHPDLKRTFNIPGGKGVKLVVAIVGL
LTSIMAFIVSFLPPDNIQGDSTDMYVELLVVSFLVVLALPFILYAVHDRKGKANTGVTLE
PINSQNAPKGHFFLHPRARSPHYIVMNDKKH

>P30845
MLKRLLKRPSLNLLAWLLLAAFYISICLNIAFFKQVLQALPLDSLHNVLVFLSMPVVAFS
VINIVLTLSSFLWLNRPLACLFILVGAAAQYFIMTYGIVIDRSMIANIIDTTPAESYALM
TPQMLLTLGFSGVLAALIACWIKIKPATSRLRSVLFRGANILVSVLLILLVAALFYKDYA
SLFRNNKELVKSLSPSNSIVASWSWYSHQRLANLPLVRIGEDAHRNPLMQNEKRKNLTIL
IVGETSRAENFSLNGYPRETNPRLAKDNVVYFPNTASCGTATAVSVPCMFSDMPREHYKE
ELAQHQEGVLDIIQRAGINVLWNDNDGGCKGACDRVPHQNVTALNLPDQCINGECYDEVL
FHGLEEYINNLQGDGVIVLHTIGSHGPTYYNRYPPQFRKFTPTCDTNEIQTCTKEQLVNT
YDNTLVYVDYIVDKAINLLKEHQDKFTTSLVYLSDHGESLGENGIYLHGLPYAIAPDSQK
QVPMLLWLSEDYQKRYQVDQNCLQKQAQTQHYSQDNLFSTLLGLTGVETKYYQAADDILQ
TCRRVSE
>P39338
MAQVINEMDVPSHSFVFHGTGERYFLICVVNVLLTIITLGIYLPWALMKCKRYLYANMEV
NGQRFSYGITGGNVFVSCLFFVFFYFAILMTVSADMPLVGCVLTLLLLVLLIFMAAKGLR
HQALMTSLNGVRFSFNCSMKGFWWVTFFLPILMAIGMGTVFFISTKMLPANSSSSVIISM
VLMAIVGIVSIGIFNGTLYSLVMSFLWSNTSFGIHRFKVKLDTTYCIKYAILAFLALLPF
LAVAGYIIFDQILNAYDSSVYANDDIENLQQFMEMQRKMIIAQLIYYFGIAVSTSYLTVS
LRNHFMSNLSLNDGRIRFRLTLTYHGMLYRMCALVVISGITGGLAYPLLKIWMIDWQAKN
TYLLGDLDDLPLINKEEQPDKGFLASISRGVMPSLPFL
>P76226
MLDRHLHPRIKPLLHQCVRVLDKPGITPDGLTLVGFAIGVLALPFLALGWYLAALVVILL
NRLLDGLDGALARRRELTDAGGFLDISLDFLFYALVPFGFILAAPEQNALAGGWLLFAFI
GTGSSFLAFAALAAKHQIDNPGYAHKSFYYLGGLTEGTETILLFVLGCLFPAWFAWFAWI
FGALCWMTTFTRVWSGYLTLKSLQRQ
>P29824
MMTTLRRRLPDIVQYSVLSLAAFLSIFPFIWMVIGTTNTTSQIIRGKVTFGTALFDNIAS
FFAQVDVPLVFWNSVKIALVGTALTLLVSSLAGYGFEMFRSKLRERVYTVILLTLMVPFA
ALMIPLFMLMGQAGLLNTHIAIMLPMIASAFIIFYFRQASKAFPTELRDAAKVDGLKEWQ
IFFYIYVPVMRSTYAAAFVIVFMLNWNNYLWPLIVLQSNDTKTITLVVSSLASAYSPEYG
TVMIGTILATLPTLLVFFAMQRQFVQGMLGSVK
>P27303
MSANAETQTPQQPVKKSGKRKRLLLLLTLLFIIIAVAIGIYWFLVLRHFEETDDAYVAGN
QIQIMSQVSGSVTKVWADNTDFVKEGDVLVTLDPTDARQAFEKAKTALASSVRQTHQLMI
NSKQLQANIEVQKIALAKAQSDYNRRVPLGNANLIGREELQHARDAVTSAQAQLDVAIQQ
YNANQAMILGTKLEDQPAVQQAATEVRNAWLALERTRIISPMTGYVSRRAVQPGAQISPT
TPLMAVVPATNMWVDANFKETQIANMRIGQPVTITTDIYGDDVKYTGKVVGLDMGTGSAF
SLLPAQNATGNWIKVVQRLPVRIELDQKQLEQYPLRIGLSTLVSVNTTNRDGQVLANKVR
STPVAVSTAREISLAPVNKLIDDIVKANAG
>P27650
MKYSKITTTLAALALPSIAHAHVGLHADGTLAGLNHPFSGLDHILAMVAVGFWASTLGGK
AVWIVPSAFVIVMAGGGVLGIEGIALPMVETAIALTVAMLGLLVAFEVKIPTPVAAIVVG
ICALFHGHVHGIELPTMSNATGYVAGFLAATVILHVLGIGLASLRFGKAGQVVARVAGGA
VALAGAALLVG
>P11551

MGNTSIQTQSYRAVDKDAGQSRSYIIPFALLCSLFFLWAVANNLNDILLPQFQQAFTLTN
FQAGLIQSAFYFGYFIIPIPAGILMKKLSYKAGIITGLFLYALGAALFWPAAEIMNYTLF
LVGLFIIAAGLGCLETAANPFVTVLGPESSGHFRLNLAQTFNSFGAIIAVVFGQSLILSN
VPHQSQDVLDKMSPEQLSAYKHSLVLSVQTPYMIIVAIVLLVALLIMLTKFPALQSDNHS
DAKQGSFSASLSRLARIRHWRWAVLAQFCYVGAQTACWSYLIRYAVEEIPGMTAGFAANY
LTGTMVCFFIGRFTGTWLISRFAPHKVLAAYALIAMALCLISAFAGGHVGLIALTLCSAF
MSIQYPTIFSLGIKNLGQDTKYGSSFIVMTIIGGGIVTPVMGFVSDAAGNIPTAELIPAL
CFAVIFIFARFRSQTATN
>P17443
MSGQGKRLMVMAGGTGGHVFPGLAVAHHLMAQGWQVRWLGTADRMEADLVPKHGIEIDFI
RISGLRGKGIKALIAAPLRIFNAWRQARAIMKAYKPDVVLGMGGYVSGPGGLAAWSLGIP
VVLHEQNGIAGLTNKWLAKIATKVMQAFPGAFPNAEVVGNPVRTDVLALPLPQQRLAGRE
GPVRVLVVGGSQGARILNQTMPQVAAKLGDSVTIWHQSGKGSQQSVEQAYAEAGQPQHKV
TEFIDDMAAAYAWADVVVCRSGALTVSEIAAAGLPALFVPFQHKDRQQYWNALPLEKAGA
AKIIEQPQLSVDAVANTLAGWSRETLLTMAERARAASIPDATERVANEVSRVARA
>P76339
MKRLSITVRLTLLFILLLSVAGAGIVWTLYNGLASELKWRDDTTLINRTAQIKQLLIDGV
NPDTLPVYFNRMMDVSQDILIIHGDSINKIVNRTNVSDGMLNNIPASETISAAGIYRSII
NDTEIDALRINIDEVSPSLTVTVAKLASARHNMLEQYKINSIIICIVAIVLCSVLSPLLI
RTGLREIKKLSGVTEALNYNDSREPVEVSALPRELKPLGQALNKMHHALVKDFERLSQFA
DDLAHELRTPINALLGQNQVTLSQTRSIAEYQKTIAGNIEELENISRLTENILFLARADK
NNVLVKLDSLSLNKEVENLLDYLEYLSDEKEICFKVECNQQIFADKILLQRMLSNLIVNA
IRYSPEKSRIHITSFLDTNSYLNIDIASPGTKINEPEKLFRRFWRGDNSRHSVGQGLGLS
LVKAIAELHGGSATYHYLNKHNVFRITLPQRN
>P75892
MAMFGFPHWQLKSTSTESGVVAPDERLPFAQTAVMGVQHAVAMFGATVLMPILMGLDPNL
SILMSGIGTLLFFFITGGRVPSYLGSSAAFVGVVIAATGFNGQGINPNISIALGGIIACG
LVYTVIGLVVMKIGTRWIERLMPPVVTGAVVMAIGLNLAPIAVKSVSASAFDSWMAVMTV
LCIGLVAVFTRGMIQRLLILVGLIVACLLYGVMTNVLGLGKAVDFTLVSHAAWFGLPHFS
TPAFNGQAMMLIAPVAVILVAENLGHLKAVAGMTGRNMDPYMGRAFVGDGLATMLSGSVG
GSGVTTYAENIGVMAVTKVYSTLVFVAAAVIAMLLGFSPKFGALIHTIPAAVIGGASIVV
FGLIAVAGARIWVQNRVDLSQNGNLIMVAVTLVLGAGDFALTLGGFTLGGIGTATFGAIL
LNALLSRKLVDVPPPEVVHQEP
>P07014
MRLEFSIYRYNPDVDDAPRMQDYTLEADEGRDMMLLDALIQLKEKDPSLSFRRSCREGVC
GSDGLNMNGKNGLACITPISALNQPGKKIVIRPLPGLPVIRDLVVDMGQFYAQYEKIKPY
LLNNGQNPPAREHLQMPEQREKLDGLYECILCACCSTSCPSFWWNPDKFIGPAGLLAAYR
FLIDSRDTETDSRLDGLSDAFSVFRCHSIMNCVSVCPKGLNPTRAIGHIKSMLLQRNA
>P31069
MSHWATFKQTATNLWVTLRHDILALAVFLNGLLIFKTIYGMSVNLLDIFHIKAFSELDLS
LLANAPLFMLGVFLVLNSIGLLFRAKLAWAISIILLLIALIYTLHFYPWLKFSIGFCIFT
LVFLLILRKDFSHSSAAAGTIFAFISFTTLLFYSTYGALYLSEGFNPRIESLMTAFYFSI
ETMSTVGYGDIVPVSESARLFTISVIISGITVFATSMTSIFGPLIRGGFNKLVKGNNHTM
HRKDHFIVCGHSILAINTILQLNQRGQNVTVISNLPEDDIKQLEQRLGDNADVIPGDSND

SSVLKKAGIDRCRAILALSDNDADNAFVVLSAKDMSSDVKTVLAVSDSKNLNKIKMVHPD
IILSPQLFGSEILARVLNGEEINNDMLVSMLLNSGHGIFSDNDELETKADSKESAQK
>P64590
MDWYLKVLKNYVGFRGRARRKEYWMFILVNIIFTFVLGLLDKMLGWQRAGGEGILTTIYG
ILVFLPWWAVQFRRLHDTDRSAWWALLFLIPFIGWLIIIVFNCQAGTPGENRFGPDPKLE
P
>P27254
MINEATLAESIRRLRQGERATLAQAMTLVESRHPRHQALSTQLLDAIMPYCGNTLRLGVT
GTPGAGKSTFLEAFGMLLIREGLKVAVIAVDPSSPVTGGSILGDKTRMNDLARAEAAFIR
PVPSSGHLGGASQRARELMLLCEAAGYDVVIVETVGVGQSETEVARMVDCFISLQIAGGG
DDLQGIKKGLMEVADLIVINKDDGDNHTNVAIARHMYESALHILRRKYDEWQPRVLTCSA
LEKRGIDEIWHAIIDFKTALTASGRLQQVRQQQSVEWLRKQTEEEVLNHLFANEDFDRYY
RQTLLAVKNNTLSPRTGLRQLSEFIQTQYFD
>P32137
MSHITTEDPATLRLPFKEKLSYGIGDLASNILLDIGTLYLLKFYTDVLGLPGTYGGIIFL
ISKFFTAFTDMGTGIMLDSRRKIGPKGKFRPFILYASFPVTLLAIANFVGTPFDVTGKTV
MATILFMLYGLFFSMMNCSYGAMVPAITKNPNERASLAAWRQGGATLGLLLCTVGFVPVM
NLIEGNQQLGYIFAATLFSLFGLLFMWICYSGVKERYVETQPANPAQKPGLLQSFRAIAG
NRPLFILCIANLCTLGAFNVKLAIQVYYTQYVLNDPILLSYMGFFSMGCIFIGVFLMPAS
VRRFGKKKVYIGGLLIWVLGDLLNYFFGGGSVSFVAFSCLAFFGSAFVNSLNWALVSDTV
EYGEWRTGVRSEGTVYTGFTFFRKVSQALAGFFPGWMLTQIGYVPNVAQADHTIEGLRQL
IFIYPSALAVVTIVAMGCFYSLNEKMYVRIVEEIEARKRTA
>P38392
MLDVFTPLLKLFANEPLERLMYTIIIFGLTLWLIPKEFTVAFNAYTEIPWLFQIIVFAFS
FVVAISFSRLRAHIQKHYSLLPEQRVLLRLSEKEIAVFKDFLKTGNLIITSPCRNPVMKK
LERKGIIQHQSDSANCSYYLVTEKYSHFMKLFWNSRSRRFNR
>P0ADM0
MKISRLGEAPDYRFSLANERTFLAWIRTALGFLAAGVGLDQLAPDFATPVIRELLALLLC
LFSGGLAMYGYLRWLRNEKAMRLKEDLPYTNSLLIISLILMVVAVIVMGLVLYAG
>P0A924
MRSIARRTAVGAALLLVMPVAVWISGWRWQPGEQSWLLKAAFWVTETVTQPWGVITHLIL
FGWFLWCLRFRIKAAFVLFAILAAAILVGQGVKSWIKDKVQEPRPFVIWLEKTHHIPVDE
FYTLKRAERGNLVKEQLAEEKNIPQYLRSHWQKETGFAFPSGHTMFAASWALLAVGLLWP
RRRTLTIAILLVWATGVMGSRLLLGMHWPRDLVVATLISWALVAVATWLAQRICGPLTPP
AEENREIAQREQES
>P0AAI3
MAKNLILWLVIAVVLMSVFQSFGPSESNGRKVDYSTFLQEVNNDQVREARINGREINVTK
KDSNRYTTYIPVQDPKLLDNLLTKNVKVVGEPPEEPSLLASIFISWFPMLLLIGVWIFFM
RQMQGGGGKGAMSFGKSKARMLTEDQIKTTFADVAGCDEAKEEVAELVEYLREPSRFQKL
GGKIPKGVLMVGPPGTGKTLLAKAIAGEAKVPFFTISGSDFVEMFVGVGASRVRDMFEQA
KKAAPCIIFIDEIDAVGRQRGAGLGGGHDEREQTLNQMLVEMDGFEGNEGIIVIAATNRP
DVLDPALLRPGRFDRQVVVGLPDVRGREQILKVHMRRVPLAPDIDAAIIARGTPGFSGAD
LANLVNEAALFAARGNKRVVSMVEFEKAKDKIMMGAERRSMVMTEAQKESTAYHEAGHAI
IGRLVPEHDPVHKVTIIPRGRALGVTFFLPEGDAISASRQKLESQISTLYGGRLAEEIIY

GPEHVSTGASNDIKVATNLARNMVTQWGFSEKLGPLLYAEEEGEVFLGRSVAKAKHMSDE
TARIIDQEVKALIERNYNRARQLLTDNMDILHAMKDALMKYETIDAPQIDDLMARRDVRP
PAGWEEPGASNNSGDNGSPKAPRPVDEPRTPNPGNTMSEQLGDK
>P0AEB0
MAEVTQLKRYDARPINWGKWFLIGIGMLVSAFILLVPMIYIFVQAFSKGLMPVLQNLADP
DMLHAIWLTVMIALIAVPVNLVFGILLAWLVTRFNFPGRQLLLTLLDIPFAVSPVVAGLV
YLLFYGSNGPLGGWLDEHNLQIMFSWPGMVLVTIFVTCPFVVRELVPVMLSQGSQEDEAA
ILLGASGWQMFRRVTLPNIRWALLYGVVLTNARAIGEFGAVSVVSGSIRGETLSLPLQIE
LLEQDYNTVGSFTAAALLTLMAIITLFLKSMLQWRLENQEKRAQQEEHHEH
>P0AER8
MFHLDTLATLVAATLTLLLGRKLVHSVSFLKKYTIPEPVAGGLLVALALLVLKKSMGWEV
NFDMSLRDPLMLAFFATIGLNANIASLRAGGRVVGIFLIVVVGLLVMQNAIGIGMASLLG
LDPLMGLLAGSITLSGGHGTGAAWSKLFIERYGFTNATEVAMACATFGLVLGGLIGGPVA
RYLVKHSTTPNGIPDDQEVPTAFEKPDVGRMITSLVLIETIALIAICLTVGKIVAQLLAG
TAFELPTFVCVLFVGVILSNGLSIMGFYRVFERAVSVLGNVSLSLFLAMALMGLKLWELA
SLALPMLAILVVQTIFMALYAIFVTWRMMGKNYDAAVLAAGHCGFGLGATPTAIANMQAI
TERFGPSHMAFLVVPMVGAFFIDIVNALVIKLYLMLPIFAG
>P39401
MSELLSFALFLASVLIYAWKAGRNTWWFAATLTVLGLFVVLNITLFASDYFTGDGINDAV
LYTLTNSLTGAGVSKYILPGIGIVLGLTAVFGALGWILRRRRHHPHHFGYSLLALLLALG
SVDASPAFRQITELVKSQSRDGDPDFAAYYKEPSKTIPDPKLNLVYIYGESLERTYFDNE
AFPDLTPELGALKNEGLDFSHTQQLPGTDYTIAGMVASQCGIPLFAPFEGNASASVSSFF
PQNICLGDILKNSGYQNYFVQGANLRFAGKDVFLKSHGFDHLYGSEELKSVVADPHYRND
WGFYDDTVLDEAWKKFEELSRSGQRFSLFTLTVDTHHPDGFISRTCNRKKYDFDGKPNQS
FSAVSCSQENIATFINKIKASPWFKDTVIVVSSDHLAMNNTAWKYLNKQDRNNLFFVIRG
DKPQQETLAVKRNTMDNGATVLDILGGDNYLGLGRSSLSGQSMSEIFLNIKEKTLAWKPD
IIRLWKFPKEMKEFTIDQQKNMIAFSGSHFRLPLLLRVSDKRVEPLPESEYSAPLRFQLA
DFAPRDNFVWVDRCYKMAQLWAPELALSTDWCVSQGQLGGQQIVQHVDKTTWQGKTAFKD
TVIDMARYKGNVDTLKIVDNDIRYKADSFIFNVAGAPEEVKQFSGISRPESWGRWSNAQL
GDEVKIEYKHPLPKKFDLVITAKAYGNNASRPIPVRVGNEEQTLVLGNEVTTTTLHFDNP
TDADTLVIVPPEPVSTNEGNILGHSPRKLGIGMVEIKVVEREG
>P0ADL1
MSEFIAENRGADAITRPNWSAVFSVAFCVACLIIVEFLPVSLLTPMAQDLGISEGVAGQS
VTVTAFVAMFASLFITQTIQATDRRYVVILFAVLLTLSCLLVSFANSFSLLLIGRACLGL
ALGGFWAMSASLTMRLVPPRTVPKALSVIFGAVSIALVIAAPLGSFLGELIGWRNVFNAA
AVMGVLCIFWIIKSLPSLPGEPSHQKQNTFRLLQRPGVMAGMIAIFMSFAGQFAFFTYIR
PVYMNLAGFGVDGLTLVLLSFGIASFIGTSLSSFILKRSVKLALAGAPLILAVSALVLTL
WGSDKIVATGVAIIWGLTFALVPVGWSTWITRSLADQAEKAGSIQVAVIQLANTCGAAIG
GYALDNIGLTSPLMLSGTLMLLTALLVTAKVKMKKS
>P0AGA2
MAKQPGLDFQSAKGGLGELKRRLLFVIGALIVFRIGSFIPIPGIDAAVLAKLLEQQRGTI
IEMFNMFSGGALSRASIFALGIMPYISASIIIQLLTVVHPTLAEIKKEGESGRRKISQYT
RYGTLVLAIFQSIGIATGLPNMPGMQGLVINPGFAFYFTAVVSLVTGTMFLMWLGEQITE
RGIGNGISIIIFAGIVAGLPPAIAHTIEQARQGDLHFLVLLLVAVLVFAVTFFVVFVERG

QRRIVVNYAKRQQGRRVYAAQSTHLPLKVNMAGVIPAIFASSIILFPATIASWFGGGTGW
NWLTTISLYLQPGQPLYVLLYASAIIFFCFFYTALVFNPRETADNLKKSGAFVPGIRPGE
QTAKYIDKVMTRLTLVGALYITFICLIPEFMRDAMKVPFYFGGTSLLIVVVVIMDFMAQV
QTLMMSSQYESALKKANLKGYGR
>P29913
MLNDQDRIFTNLYGMGDRSLAGAKKRGHWDGTAAIIQRGRDKIIDEMKASGLRGRGGAGF
PTGMKWSFMPKESDGRPSYLVINADESEPATCKDREIMRHDPHTLIEGALIASFAMGAHA
AYIYIRGEFIREREALQAAIDECYDAGLLGRNAAGSGWDFDLYLHHGAGAYICGEETALL
ESLEGKKGMPRMKPPFPAGAGLYGCPTTVNNVESIAVVPTILRRGAEWFASFGRPNNAGV
KLFGLTGHVNTPCVVEEAMSIPMRELIEKHGGGIRGGWKNLKAVIPGGASCPVLTAEQCE
NAIMDYDGMRDVRSSFGTACMIVMDQSTDVVKAIWRLSKFFKHESCGQCTPCREGTGWMM
RVMERLVRGDAEVEEIDMLFDVTKQVEGHTICALGDAAAWPIQGLIRNFREEIEDRIKAK
RTGRMGAMAAE
>P02916
MDVIKKKHWWQSDALKWSVLGLLGLLVGYLVVLMYAQGEYLFAITTLILSSAGLYIFANR
KAYAWRYVYPGMAGMGLFVLFPLVCTIAIAFTNYSSTNQLTFERAQEVLLDRSWQAGKTY
NFGLYPAGDEWQLALSDGETGKNYLSDAFKFGGEQKLQLKETTAQPEGERANLRVITQNR
QALSDITAILPDGNKVMMSSLRQFSGTQPLYTLDGDGTLTNNQSGVKYRPNNQIGFYQSI
TADGNWGDEKLSPGYTVTTGWKNFTRVFTDEGIQKPFLAIFVWTVVFSLITVFLTVAVGM
VLACLVQWEALRGKAVYRVLLILPYAVPSFISILIFKGLFNQSFGEINMMLSALFGVKPA
WFSDPTTARTMLIIVNTWLGYPYMMILCMGLLKAIPDDLYEASAMDGAGPFQNFFKITLP
LLIKPLTPLMIASFAFNFNNFVLIQLLTNGGPDRLGTTTPAGYTDLLVNYTYRIAFEGGG
GQDFGLAAAIATLIFLLVGALAIVNLKATRMKFD
>P0A8Q0
MTTKRKPYVRPMTSTWWKKLPFYRFYMLREGTAVPAVWFSIELIFGLFALKNGPEAWAGF
VDFLQNPVIVIINLITLAAALLHTKTWFELAPKAANIIVKDEKMGPEPIIKSLWAVTVVA
TIVILFVALYW
>P76552
MGINEIIMYIMMFFMLIAAVDRILSQFGGSARFLGKFGKSIEGSGGQFEEGFMAMGALGL
AMVGMTALAPVLAHVLGPVIIPVYEMLGANPSMFAGTLLACDMGGFFLAKELAGGDVAAW
LYSGLILGSMMGPTIVFSIPVALGIIEPSDRRYLALGVLAGIVTIPIGCIAGGLVAMYSG
VQINGQPVEFTFALILMNMIPVIIVAILVALGLKFIPEKMINGFQIFAKFLVALITLGLA
AAVVKFLLGWELIPGLDPIFMAPGDKPGEVMRAIEVIGSISCVLLGAYPMVLLLTRWFEK
PLMSVGKVLNMNNIAAAGMVATLANNIPMFGMMKQMDTRGKVINCAFAVSAAFALGDHLG
FAAANMNAMIFPMIVGKLIGGVTAIGVAMMLVPKEDATATKTEAEAQS
>P06611
MSIVMQLQDVAESTRLGPLSGEVRAGEILHLVGPNGAGKSTLLARMAGMTSGKGSIQFAG
QPLEAWSATKLALHRAYLSQQQTPPFATPVWHYLTLHQHDKTRTELLNDVAGALALDDKL
GRSTNQLSGGEWQRVRLAAVVLQITPQANPAGQLLLLDEPMNSLDVAQQSALDKILSALC
QQGLAIVMSSHDLNHTLRHAHRAWLLKGGKMLASGRREEVLTPPNLAQAYGMNFRRLDIE
GHRMLISTI
>P42601
MNTVGTPLLWGGFAVVVAIMLAIDLLLQGRRGAHAMTMKQAAAWSLVWVTLSLLFNAAFW
WYLVQTEGRAVADPQALAFLTGYLIEKSLAVDNVFVWLMLFSYFSVPAALQRRVLVYGVL

GAIVLRTIMIFTGSWLISQFDWILYIFGAFLLFTGVKMALAHEDESGIGDKPLVRWLRGH
LRMTDTIDNEHFFVRKNGLLYATPLMLVLILVELSDVIFAVDSIPAIFAVTTDPFIVLTS
NLFAILGLRAMYFLLAGVAERFSMLKYGLAVILVFIGIKMLIVDFYHIPIAVSLGVVFGI
LVMTFIINAWVNYRHDKQRGG
>P45800
MSTIVIFLAALLACSLLAGWLIKVRSRRRQLPWTNAFADAQTRKLTPEERSAVENYLESL
TQVLQVPGPTGASAAPISLALNAESNNVMMLTHAITRYGISTDDPNKWRYYLDSVEVHLP
PFWEQYINDENTVELIHTDSLPLVISLNGHTLQEYMQETRSYALQPVPSTQASIRGEESE
QIELLNIRKETHEEYALSRPRGLREALLIVASFLMFFFCLITPDVFVPWLAGGALLLLGA
GLWGLFAPPAKSSLREIHCLRGTPRRWGLFGENDQEQINNISLGIIDLVYPAHWQPYIAQ
DLGQQTDIDIYLDRHVVRQGRYLSLHDEVKNFPLQHWLRSTIIAAGSLLVLFMLLFWIPL
DMPLKFTLSWMKGAQTIEATSVKQLADAGVRVGDTLRISGTGMCNIRTSGTWSAKTNSPF
LPFDCSQIIWNDARSLPLPESELVNKATALTEAVNRQLHPKPEDESRVSASLRSAIQKSG
MVLLDDFGDIVLKTADLCSAKDDCVRLKNALVNLGNSKDWDALVKRANAGKLDGVNVLLR
PVSAESLDNLVATSTAPFITHETARAAQSLNSPAPGGFLIVSDEGSDFVDQPWPSASLYD
YPPQEQWNAFQKLAQMLMHTPFNAEGIVTKIFTDANGTQHIGLHPIPDRSGLWRYLSTTL
LLLTMLGSAIYNGVQAWRRYQRHRTRMMEIQAYYESCLNPQLITPSESLIE
>P55734
MALTTISPHDAQELIARGAKLIDIRDADEYLREHIPEADLAPLSVLEQSGLPAKLRHEQI
IFHCQAGKRTSNNADKLAAIAAPAEIFLLEDGIDGWKKAGLPVAVNKSQPLPLMRQVQIA
AGGLILIGVVLGYTVNSGFFLLSGFVGAGLLFAGISGFCGMARLLDKMPWNQRA
>P75925
MSFTNTPERYGVISAAFHWLSAIIVYGMFALGLWMVTLSYYDGWYHKAPELHKSIGILLM
MGLVIRVLWRVISPPPGPLPSYSPMTRLAARAGHLALYLLLFAIGISGYLISTADGKPIS
VFGWFDVPATLADAGAQADFAGALHFWLAWSVVVLSVMHGFMALKHHFIDKDDTLKRMLG
KSSSDYGV
>P21865
MNNEPLRPDPDRLLEQTAAPHRGKLKVFFGACAGVGKTWAMLAEAQRLRAQGLDIVVGVV
ETHGRKDTAAMLEGLAVLPLKRQAYRGRHISEFDLDAALARRPALILMDELAHSNAPGSR
HPKRWQDIEELLEAGIDVFTTVNVQHLESLNDVVSGVTGIQVRETVPDPFFDAADDVVLV
DLPPDDLRQRLKEGKVYIAGQAERAIEHFFRKGNLIALRELALRRTADRVDEQMRAWRGH
PGEEKVWHTRDAILLCIGHNTGSEKLVRAAARLASRLGSVWHAVYVETPALHRLPEKKRR
AILSALRLAQELGAETATLSDPAEEKAVVRYAREHNLGKIILGRPASRRWWRRETFADRL
ARIAPDLDQVLVALDEPPARTINNAPDNRSFKDKWRVQIQGCVVAAALCAVITLIAMQWL
MAFDAANLVMLYLLGVVVVALFYGRWPSVVATVINVVSFDLFFIAPRGTLAVSDVQYLLT
FAVMLTVGLVIGNLTAGVRYQARVARYREQRTRHLYEMSKALAVGRSPQDIAATSEQFIA
STFHARSQVLLPDDNGKLQPLTHPQGMTPWDDAIAQWSFDKGLPAGAGTDTLPGVPYQIL
PLKSGEKTYGLVVVEPGNLRQLMIPEQQRLLETFTLLVANALERLTLTASEEQARMASER
EQIRNALLAALSHDLRTPLTVLFGQAEILTLDLASEGSPHARQASEIRQHVLNTTRLVNN
LLDMARIQSGGFNLKKEWLTLEEVVGSALQMLEPGLSSPINLSLPEPLTLIHVDGPLFER
VLINLLENAVKYAGAQAEIGIDAHVEGENLQLDVWDNGPGLPPGQEQTIFDKFARGNKES
AVPGVGLGLAICRAIVDVHGGTITAFNRPEGGACFRVTLPQQTAPELEEFHEDM
>P07000
MFQQQKDWETRENAFAAFTMGPLTDFWRQRDEAEFTGVDDIPVRFVRFRAQHHDRVVVIC

PGRIESYVKYAELAYDLFHLGFDVLIIDHRGQGRSGRLLADPHLGHVNRFNDYVDDLAAF
WQQEVQPGPWRKRYILAHSMGGAISTLFLQRHPGVCDAIALTAPMFGIVIRMPSFMARQI
LNWAEAHPRFRDGYAIGTGRWRALPFAINVLTHSRQRYRRNLRFYADDPTIRVGGPTYHW
VRESILAGEQVLAGAGDDATPTLLLQAEEERVVDNRMHDRFCELRTAAGHPVEGGRPLVI
KGAYHEILFEKDAMRSVALHAIVDFFNRHNSPSGNRSTEV
>P69681
MKIATIKTGLASLAMLPGLVMAAPAVADKADNAFMMICTALVLFMTIPGIALFYGGLIRG
KNVLSMLTQVTVTFALVCILWVVYGYSLAFGEGNNFFGNINWLMLKNIELTAVMGSIYQY
IHVAFQGSFACITVGLIVGALAERIRFSAVLIFVVVWLTLSYIPIAHMVWGGGLLASHGA
LDFAGGTVVHINAAIAGLVGAYLIGKRVGFGKEAFKPHNLPMVFTGTAILYIGWFGFNAG
SAGTANEIAALAFVNTVVATAAAILGWIFGEWALRGKPSLLGACSGAIAGLVGVTPACGY
IGVGGALIIGVVAGLAGLWGVTMLKRLLRVDDPCDVFGVHGVCGIVGCIMTGIFAASSLG
GVGFAEGVTMGHQLLVQLESIAITIVWSGVVAFIGYKLADLTVGLRVPEEQEREGLDVNS
HGENAYNA
>P0AEN4
MISRVTEALSKVKGSMGSHERHALPGVIGDDLLRFGKLPLCLFICIILTAVTVVTTAHHT
RLLTAQREQLVLERDALDIEWRNLILEENALGDHSRVERIATEKLQMQHVDPSQENIVVQ
K
>P0AEK7
MSKSKMIVRTKFIDRACHWTVVICFFLVALSGISFFFPTLQWLTQTFGTPQMGRILHPFF
GIAIFVALMFMFVRFVHHNIPDKKDIPWLLNIVEVLKGNEHKVADVGKYNAGQKMMFWSI
MSMIFVLLVTGVIIWRPYFAQYFPMQVVRYSLLIHAAAGIILIHAILIHMYMAFWVKGSI
KGMIEGKVSRRWAKKHHPRWYREIEKAEAKKESEEGI
>Q03478
MQKQEHVVFKRAKAHGHDEPHGGAWKVAFADFMIALMALFLVLWVMQVVDKEERKAIVAH
LHSSSVFDKSYGNPFDTSQSISPIDLAQDSSVPSKHNSNHVVSSYFQGDGDGPEINSLVP
GTFDTQEQLAALAKVIEEMTAQINAQGNVNVTVTPQGLRIVLQDDYKQHMFSRGGAELTP
FFEDLLLALAPLFEQVTNPLIISGHTDAIPFKKRFGRQSNWALSASRADVARKTLVEGGM
PDDRVMQVTGMSDRALLNPDEPDSSENRRIELFILTTPAAKVLETLFGNQDDSELQKAKQ
KAEFNQPVIRQEVIRYSADAEKQEAKIQAL
>P75788
MSLPFLRTLQGDRFFQLLILVGIGLSFFVPFAPKSWPAAIDWHTIITLSGLMLLTKGVEL
SGYFDVLGRKMVRRFATERRLAMFMVLAAALLSTFLTNDVALFIVVPLTITLKRLCEIPV
NRLIIFEALAVNAGSLLTPIGNPQNILIWGRSGLSFAGFIAQMAPLAGAMMLTLLLLCWC
CFPGKAMQYHTGVQTPEWKPRLVWSCLGLYIVFLTALEFKQELWGLVIVAAGFALLARRV
VLSVDWTLLLVFMAMFIDVHLLTQLPALQGVLGNVSHLSEPGLWLTAIGLSQVISNVPST
ILLLNYVPPSLLLVWAVNVGGFGLLPGSLANLIALRMANDRRIWWRFHLYSIPMLLWAAL
VGYVLLVILPAN
>P75961
MKKFIILLSLLILLPLTAASKPLIPIMKTLFTDVTGTVPDAEEIAHKAELFRQQTGIAPF
IVVLPDINNEASLRQNGKAMLAHASSSLSDVKGSVLLLFTTREPRLIMITNGQVESGLDD
KHLGLLIENHTLAYLNADLWYQGINNALAVLQAQILKQSTPPLTYYPHPGQQHENAPPGS
TNTLGFIAWAATFILFSRIFYYTTRFIYALKFAVAMTIANMGYQALCLYIDNSFAITRIS
PLWAGLIGVCTFIAALLLTSKR

>O33953
MRVENNNVSGQNLDPEQIDLIDLLVQLWRGKMTIIISVIVAIVLAIGYLVVAKEKWTSTA
IVTQPDVGQIAGYNNAINVIYGSAAPKVSEIQSILIGRFSTTFSALAETLDNQEEPEKLT
IEPTVKNQSLPLAVSYVGQSPEAAQKQLAQYIQQVDDQVNDELEKDLKDNIALRMKNLQD
SLKTQEVVAQEQKELRIRQIQEALQYANQAQVTKPQIQQTQDVTQDTMFLLGSDALESMV
KHEASRPLVFSSTYYQTRQNLLDIESLKVDDLDIHAYRYVMKPTLPIRRDSPKKAITLIL
AVLLGGMVGAGIVLGRNALRNYNAK
>P15928
MSATASTATQPKPLEWLNRLRANPRIPLIVAGSAAVAIVVAMVLWAKTPDYRTLFSNLSD
QDGGAIVAQLTQMNIPYRFANGSGAIEVPADKVHELRLRLAQQGLPKGGAVGFELLDQEK
FGISQFSEQVNYQRALEGELARTIETLGPVKSARVHLAMPKPSLFVREQKSPSASVTVTL
EPGRALDEGQISAVVHLVSSAVAGLPPGNVTLVDQSGHLLTQSNTSGRDLNDAQLKFAND
VESRIQRRIEAILSPIVGNGNVHAQVTAQLDFANKEQTEEHYSPNGDASKATLRSRQLNI
SEQVGAGYPGGVPGALSNQPAPPNEAPIATPPTNQQNAQNTPQTSTSTNSNSAGPRSTQR
NETSNYEVDRTIRHTKMNVGDIERLSVAVVVNYKTLADGKPLPLTADQMKQIEDLTREAM
GFSDKRGDTLNVVNSPFSAVDNTGGELPFWQQQSFIDQLLAAGRWLLVLVVAWILWRKAV
RPQLTRRVEEAKAAQEQAQVRQETEEAVEVRLSKDEQLQQRRANQRLGAEVMSQRIREMS
DNDPRVVALVIRQWMSNDHE
>Q9ZHG0
MTLLSFGFSPVFFSVMAFCIISRSKFYPQRTRNKVIVLILLTFFICFLYPLTKVYLVGSY
GIFDKFYLFCFISTLIAIAINVVILTINGAKNERN
>P77308
MTFWSILRQRCWGLVLVVAGVCVITFIISHLIPGDPARLLAGDRASDAIVENIRQQLGLD
QPLYVQFYRYVSDLFHGDLGTSIRTGRPVLEELRIFFPATLELAFGALLLALLIGIPLGI
LSAVWRNRWLDHLVRIMAITGISTPAFWLGLGVIVLFYGHLQILPGGGRLDDWLDPPTHV
TGFYLLDALLEGNGEVFFNALQHLILPALTLAFVHLGIVARQIRSAMLEQLSEDYIRTAR
ASGLPGWYIVLCYALPNALIPSITVLGLALGDLLYGAVLTETVFAWPGMGAWVVTSIQAL
DFPAVMGFAVVVSFAYVLVNLVVDLLYLWIDPRIGRGGGE
>P26266
MSSLNIKQGSDAHFPDYPLASPSNNEIDLLNLISVLWRAKKTVMAVVFAFACAGLLISFI
LPQKWTSAAVVTPPEPVQWQELEKSFTKLRVLDLDIKIDRTEAFNLFIKKFQSVSLLEEY
LRSSPYVMDQLKEAKIDELDLHRAIVALSEKMKAVDDNASKKKDEPSLYTSWTLSFTAPT
SEEAQTVLSGYIDYISTLVVKESLENVRNKLEIKTQFEKEKLAQDRIKTKNQLDANIQRL
NYSLDIANAAGIKKPVYSNGQAVKDDPDFSISLGADGIERKLEIEKAVTDVAELNGELRN
RQYLVEQLTKAHVNDVNFTPFKYQLSPSLPVKKDGPGKAIIVILSALIGGMVACGGVLLR
YAMASRKQDAMMADHLV
>P33361
MTYFRINPVLALLLLLTAIAAALPFISYAPNRLVSGEGRHLWQLWPQTIWMLVGVGCAWL
TACFIPGKKGSICALILAQFVFVLLVWGAGKAATQLAQNGSALARTSLGSGFWLAAALAL
LACSDAIRRISTHPLWRWLLHMQIAIIPLWLLYSGTLNDLSLMKEYANRQDVFDDALAQH
LTLLFGAVLPALVIGVPLGIWCYFSTARQGAIFSLLNVIQTVPSVALFGLLIAPLAALVT
AFPWLGTLGIAGTGMTPALIALVLYALLPLVRGVVVGLNQIPRDVLESARAMGMSGAQRF
LHVQLPLALPVFLRSLRVVMVQTVGMAVIAALIGAGGFGALVFQGLLSSAIDLVLLGVIP
VIVLAVLTDALFDLLIALLKVKRND

>P0ABT8
MSAAGKSNPLAISGLVVLTLIWSYSWIFMKQVTSYIGAFDFTALRCIFGALVLFIVLLLR
GRGMRPTPFKYTLAIALLQTCGMVGLAQWALVSGGAGKVAILSYTMPFWVVIFAALFLGE
RLRRGQYFAILIAAFGLFLVLQPWQLDFSSMKSAMLAILSGVSWGASAIVAKRLYARHPR
VDLLSLTSWQMLYAALVMSVVALLVPQREIDWQPTVFWALAYSAILATALAWSLWLFVLK
NLPASIASLSTLAVPVCGVLFSWWLLGENPGAVEGSGIVLIVLALALVSRKKKEAVSVKR
I
>P76460
MIGRISRFMTRFVSRWLPDPLIFAMLLTLLTFVIALWLTPQTPISMVKMWGDGFWNLLAF
GMQMALIIVTGHALASSAPVKSLLRTAASAAKTPVQGVMLVTFFGSVACVINWGFGLVVG
AMFAREVARRVPGSDYPLLIACAYIGFLTWGGGFSGSMPLLAATPGNPVEHIAGLIPVGD
TLFSGFNIFITVALIVVMPFITRMMMPKPSDVVSIDPKLLMEEADFQKQLPKDAPPSERL
EESRILTLIIGALGIAYLAMYFSEHGFNITINTVNLMFMIAGLLLHKTPMAYMRAISAAA
RSTAGILVQFPFYAGIQLMMEHSGLGGLITEFFINVANKDTFPVMTFFSSALINFAVPSG
GGHWVIQGPFVIPAAQALGADLGKSVMAIAYGEQWMNMAQPFWALPALAIAGLGVRDIMG
YCITALLFSGVIFVIGLTLF
>P77377
MSLREKTISGAKWSAIATVIIIGLGLVQMTVLARIIDNHQFGLLTVSLVIIALADTLSDF
GIANSIIQRKEISHLELTTLYWLNVGLGIVVCVAVFLLSDLIGDVLNNPDLAPLIKTLSL
AFVVIPHGQQFRALMQKELEFNKIGMIETSAVLAGFTCTVVSAHFWPLAMTAILGYLVNS
AVRTLLFGYFGRKIYRPGLHFSLASVAPNLRFGAWLTADSIINYLNTNLSTLVLARILGA
GVAGGYNLAYNVAVVPPMKLNPIITRVLFPAFAKIQDDTEKLRVNFYKLLSVVGIINFPA
LLGLMVVSNNFVPLVFGEKWNSIIPVLQLLCVVGLLRSVGNPIGSLLMAKARVDISFKFN
VFKTFLFIPAIVIGGQMAGAIGVTLGFLLVQIINTILSYFVMIKPVLGSSYRQYILSLWL
PFYLSLPTLVVSYALGIVLKGQLALGMLLAVQIATGVLAFVVMIVLSRHPLVVEVKRQFC
RSEKMKMLLRAG
>P07654
MAMVEMQTTAALAESRRKMQARRRLKNRIALTLSMATMAFGLFWLIWILMSTITRGIDGM
SLALFTEMTPPPNTEGGGLANALAGSGLLILWATVFGTPLGIMAGIYLAEYGRKSWLAEV
IRFINDILLSAPSIVVGLFVYTIVVAQMEHFSGWAGVIALALLQVPIVIRTTENMLKLVP
YSLREAAYALGTPKWKMISAITLKASVSGIMTGILLAIARIAGETAPLLFTALSNQFWST
DMMQPIANLPVTIFKFAMSPFAEWQQLAWAGVLIITLCVLLLNILARVVFAKNKHG
>P0AAC6
MDRIVSSSHDRTSLLSTHKVLRNTYFLLSLTLAFSAITATASTVLMLPSPGLILTLVGMY
GLMFLTYKTANKPTGIISAFAFTGFLGYILGPILNTYLSAGMGDVIAMALGGTALVFFCC
SAYVLTTRKDMSFLGGMLMAGIVVVLIGMVANIFLQLPALHLAISAVFILISSGAILFET
SNIIHGGETNYIRATVSLYVSLYNIFVSLLSILGFASRD
>P29915
MADLRKIKIDDTIIEVDPNMTLIQACEMAGIEVPRFCYHERLSIAGNCRMCLVEVVGGPP
KPAASCAMQVKDLRPGPEGAPSEIRTNSPMVKKAREGVMEFLLINHPLDCPICDQGGECD
LQDQAMAYGVDFSRYREPKRATEDLNLGPLVETHMTRCISCTRCVRFTTEVAGITQMGQT
GRGEDSEITSYLNQTLESNMQGNIIDLCPVGALVSKPYAFTARPWELTKTESIDVMDALG
SSIRIDTKGREVMRILPRNHDGVNEEWISDKTRFVWDGLRRQRLDRPYIRENGRLRPASW
PEALEAAARAMKGKKIAGLIGDLVPAEAAFSLKQLVEGLGGKVECRVDGARLPAGNRSAY

VGTARIEDIDDAEMIQLIGTNPRDEAPVLNARIRKAWSKGAKVGLVGEPVDLTYDYAHVG
TDRAALESLSSREISDETKARPSIVIVGQGAIARRDGEAVLAHAMKLAENSNSGLLILHT
AAGRVGAMDVGAVTEGGLLAAIDGAEVVYNLGADEVDIDQGPFVIYQGSHGDRGAHRDII
LPGACYTEESGLFVNTEGRPQLAMRANFAPGEGKENWAILRALSAELGATQPWDSLAGLR
RKLVEAVPHLAQIDQVPQNEWQPLGRFDLGQASFRYAIRDFYLTNPIARSSPLMGELSAM
AAARKAPAPLAAE
>P0A8U0
MDDLTAQALKDFTARYCDAWHEEHKSWPLSEELYGVPSPCIISTTEDAVYWQPQPFTGEQ
NVNAVERAFDIVIQPTIHTFYTTQFAGDMHAQFGDIKLTLLQTWSEDDFRRVQENLIGHL
VTQKRLKLPPTLFIATLEEELEVISVCNLSGEVCKETLGTRKRTHLASNLAEFLNQLKPL
L
>P69805
MSEMVDTTQTTTEKKLTQSDIRGVFLRSNLFQGSWNFERMQALGFCFSMVPAIRRLYPEN
NEARKQAIRRHLEFFNTQPFVAAPILGVTLALEEQRANGAEIDDGAINGIKVGLMGPLAG
VGDPIFWGTVRPVFAALGAGIAMSGSLLGPLLFFILFNLVRLATRYYGVAYGYSKGIDIV
KDMGGGFLQKLTEGASILGLFVMGALVNKWTHVNIPLVVSRITDQTGKEHVTTVQTILDQ
LMPGLVPLLLTFACMWLLRKKVNPLWIIVGFFVIGIAGYACGLLGL
>P0ACB7
MLKVLLLFVLLIAGIVVGPMIAGHQGYVLIQTDNYNIETSVTGLAIILILAMVVLFAIEW
LLRRIFRTGAHTRGWFVGRKRRRARKQTEQALLKLAEGDYQQVEKLMAKNADHAEQPVVN
YLLAAEAAQQRGDEARANQHLERAAELAGNDTIPVEITRVRLQLARNENHAARHGVDKLL
EVTPRHPEVLRLAEQAYIRTGAWSSLLDIIPSMAKAHVGDEEHRAMLEQQAWIGLMDQAR
ADNGSEGLRNWWKNQSRKTRHQVALQVAMAEHLIECDDHDTAQQIIIDGLKRQYDDRLLL
PIPRLKTNNPEQLEKVLRQQIKNVGDRPLLWSTLGQSLMKHGEWQEASLAFRAALKQRPD
AYDYAWLADALDRLHKPEEAAAMRRDGLMLTLQNNPPQ
>P0AFR2
MNKIFSSHVMPFRALIDACWKEKYTAARFTRDLIAGITVGIIAIPLAMALAIGSGVAPQY
GLYTAAVAGIVIALTGGSRFSVSGPTAAFVVILYPVSQQFGLAGLLVATLLSGIFLILMG
LARFGRLIEYIPVSVTLGFTSGIGITIGTMQIKDFLGLQMAHVPEHYLQKVGALFMALPT
INVGDAAIGIVTLGILVFWPRLGIRLPGHLPALLAGCAVMGIVNLLGGHVATIGSQFHYV
LADGSQGNGIPQLLPQLVLPWDLPNSEFTLTWDSIRTLLPAAFSMAMLGAIESLLCAVVL
DGMTGTKHKANSELVGQGLGNIIAPFFGGITATAAIARSAANVRAGATSPISAVIHSILV
ILALLVLAPLLSWLPLSAMAALLLMVAWNMSEAHKVVDLLRHAPKDDIIVMLLCMSLTVL
FDMVIAISVGIVLASLLFMRRIARMTRLAPVVVDVPDDVLVLRVIGPLFFAAAEGLFTDL
ESRLEGKRIVILKWDAVPVLDAGGLDAFQRFVKRLPEGCELRVCNVEFQPLRTMARAGIQ
PIPGRLAFFPNRRAAMADL
>P0AEW1
MTGSMIVNNLAGLMMLTSLFVISVKSYRLSCGFYACQSLVLVSIFATLSCLFAAEQLLIW
SASAFITKVLLVPLIMTYAARNIPQNIPEKALFGPAMMALLAALIVLLCAFVVQPVKLPM
ATGLKPALAVALGHFLLGLLCIVSQRNILRQIFGYCLMENGSHLVLALLAWRAPELVEIG
IATDAIFAVIVMVLLARKIWRTHGTLDVNNLTALKG
>P0AAP7
MADFTLSKSLFSGKYRNASSTPGNIAYALFVLFCFWAGAQLLNLLVHAPGVYERLMQVQE
TGRPRVEIGLGVGTIFGLIPFLVGCLIFAVVALWLHWRHRRQ

>P32678
MHSTEVQAKPLFSWKALGWALLYFWFFSTLLQAIIYISGYSGTNGIRDSLLFSSLWLIPV
FLFPKRIKIIAAVIGVVLWAASLAALCYYVIYGQEFSQSVLFVMFETNTNEASEYLSQYF
SLKIVLIALAYTAVAVLLWTRLRPVYIPKPWRYVVSFALLYGLILHPIAMNTFIKNKPFE
KTLDNLASRMEPAAPWQFLTGYYQYRQQLNSLTKLLNENNALPPLANFKDESGNEPRTLV
LVIGESTQRGRMSLYGYPRETTPELDALHKTDPNLTVFNNVVTSRPYTIEILQQALTFAN
EKNPDLYLTQPSLMNMMKQAGYKTFWITNQQTMTARNTMLTVFSRQTDKQYYMNQQRTQS
AREYDTNVLKPFQEVLNDPAPKKLIIVHLLGTHIKYKYRYPENQGKFDGNTDHVPPGLNA
EELESYNDYDNANLYNDHVVASLIKDFKAANPNGFLVYFSDHGEEVYDTPPHKTQGRNED
NPTRHMYTIPFLLWTSEKWQATHPRDFSQDVDRKYSLAELIHTWSDLAGLSYDGYDPTRS
VVNPQFKETTRWIGNPYKKNALIDYDTLPYGDQVGNQ
>P13033
MRFDTVIMGGGLAGLLCGLQLQKHGLRCAIVTRGQSALHFSSGSLDLLSHLPDGQPVTDI
HSGLESLRQQAPAHPYSLLEPQRVLDLACQAQALIAESGAQLQGSVELAHQRVTPLGTLR
STWLSSPEVPVWPLPAKKICVVGISGLMDFQAHLAAASLRELGLAVETAEIELPELDVLR
NNATEFRAVNIARFLDNEENWPLLLDALIPVANTCEMILMPACFGLADDKLWRWLNEKLP
CSLMLLPTLPPSVLGIRLQNQLQRQFVRQGGVWMPGDEVKKVTCKNGVVNEIWTRNHADI
PLRPRFAVLASGSFFSGGLVAERNGIREPILGLDVLQTATRGEWYKGDFFAPQPWQQFGV
TTDETLRPSQAGQTIENLFAIGSVLGGFDPIAQGCGGGVCAVSALHAAQQIAQRAGGQQ
>P23136
MAEAEHTASTPGGESSRRDFLIYGTTAVGAVGVALAVWPFIDFMNPAADTLALASTEVDV
SAIAEGQAITVTWRGKPVFVRHRTQKEIVVARAVDPASLRDPQTDEARVQQAQWLVMVGV
CTHLGCIPLGQKAGDPKGDFDGWFCPCHGSHYDSAGRIRKGPAPLNLPVPPYAFTDDTTV
LIG
>P0A996
MNDTSFENCIKCTVCTTACPVSRVNPGYPGPKQAGPDGERLRLKDGALYDEALKYCINCK
RCEVACPSDVKIGDIIQRARAKYDTTRPSLRNFVLSHTDLMGSVSTPFAPIVNTATSLKP
VRQLLDAALKIDHRRTLPKYSFGTFRRWYRSVAAQQAQYKDQVAFFHGCFVNYNHPQLGK
DLIKVLNAMGTGVQLLSKEKCCGVPLIANGFTDKARKQAITNVESIREAVGVKGIPVIAT
SSTCTFALRDEYPEVLNVDNKGLRDHIELATRWLWRKLDEGKTLPLKPLPLKVVYHTPCH
MEKMGWTLYTLELLRNIPGLELTVLDSQCCGIAGTYGFKKENYPTSQAIGAPLFRQIEES
GADLVVTDCETCKWQIEMSTSLRCEHPITLLAQALA
>P75958
MAMPLSLLIGLRFSRGRRRGGMVSLISVISTIGIALGVAVLIVGLSAMNGFERELNNRIL
AVVPHGEIEAVDQPWTNWQEALDHVQKVPGIAAAAPYINFTGLVESGANLRAIQVKGVNP
QQEQRLSALPSFVQGDAWRNFKAGEQQIIIGKGVADALKVKQGDWVSIMIPNSNPEHKLM
QPKRVRLHVAGILQLSGQLDHSFAMIPLADAQQYLDMGSSVSGIALKMTDVFNANKLVRD
AGEVTNSYVYIKSWIGTYGYMYRDIQMIRAIMYLAMVLVIGVACFNIVSTLVMAVKDKSG
DIAVLRTLGAKDGLIRAIFVWYGLLAGLFGSLCGVIIGVVVSLQLTPIIEWIEKLIGHQF
LSSDIYFIDFLPSELHWLDVFYVLVTALLLSLLASWYPARRASNIDPARVLSGQ
>P0A7C8
MTSLSRPRVEFISTILQTVLNLGLLCLGLILVVFLGKETVHLADVLFAPEQTSKYELVEG
LVVYFLYFEFIALIVKYFQSGFHFPLRYFVYIGITAIVRLIIVDHKSPLDVLIYSAAILL
LVITLWLCNSKRLKRE

>P33790
MNEVYVIAGGEWLRNNLNAIAAFMGTWTWDSIEKIALTLSVLAVAVMWVQRHNVMDLLGW
VAVFVLISLLVNVRTSVQIIDNSDLVKVHRVDNVPVGLAMPLSLTTRIGHAMVASYEMIF
TQPDSVTYSKTGMLFGANLIVKSTDFLSRNPEIINLFQDYVQNCVLGDIYLNHKYTLEDL
MASADPYTLIFSRPSPLRGVYDNNNNFITCKDASVTLKDRLNLDTKTGGKTWHYYVQQIF
GGRPDPDLLFRQLVSDSYSYFYGSSQSASQIMRQNVTMNALKEGITSNAARNGDTASLVS
LATTSSMEKQRLAHVSIGHVTMRNLPMVQTILTGIAIGIFPLLILAAVFNKLTLSVLKGY
VFALMWLQTWPLLYAILNSAMTFYAKQNGAPVVLSELSQIQLKYSNLASTAGYLSAMIPP
LSWMMVKGLGAGFSSVYSHFASSSISPTASAAGSVVDGNYSYGNMQTENVNGFSWSTNST
TSFGQMMYQTGSGATATQTRDGNMVMDASGAMSRLPVGINATRQIAAAQQEMAREASNRA
ESALHGFSSSIASAWNTLSQFGSNRGSSDSVTGGADSTMSAQDSMMASRMRSAVESYAKA
HNISNEQATRELASRSTNASLGLYGDAYAKGHLGISVLGNGGGVGLQAGAKASIDGSDLD
SHEASSGSRASHDARHDIDARATQDFKEASDYFTSRKVSESGSHTDNNADSRVDQLSAAL
NSAKQSYDQYTTNMTRSHEYAEMASRTESMSGQMSEDLSQQFAQYVMKNAPQDVEAILTN
TSSPEIAERRRAMAWSFVQEQVQPGVDNTWRESRRDIGKGMESVPSGGGSQDIIADHQGH
QAIIEQRTQDSNIRNDVKHQVDNMVTEYRGNIGDTQNSIRGEENIVKGQYSELQNHHKTE
ALTQNNKYNEEKLAQERIPGADSPKELLEKAKSYQHKE
>P32709
MTQTSAFHFESLVWDWPIAIYLFLIGISAGLVTLAVLLRRFYPQAGGADSTLLRTTLIVG
PGAVILGLLILVFHLTRPWTFWKLMFHYSFTSVMSMGVMLFQLYMVVLVLWLAKIFEHDL
LALQQRWLPKLGIVQKVLSLLTPVHRGLETLMLVLAVLLGAYTGFLLSALKSYPFLNNPI
LPVLFLFSGISSGAAVALIAMAIRQRSNPHSTEAQFVHRMEIPVVWGEIFLLVAFFVGLA
LGDDGKVRALVAALGGGFWTWWFWLGVAGLGLIVPMLLKPWVNRSSGIPAVLAACGASLV
GVLMLRFFILYAGQLTVA
>P29923
MIGLTHYLVVGAILFVTGIFGIFVNRKNVIVILMSIELMLLAVNINFVAFSTHLGDLAGQ
VFTMFVLTVAAAEAAIGLAILVVFFRNRGTIAVEDVNVMKG
>P37619
MNVFSQTQRYKALFWLSLFHLLVITSSNYLVQLPVSILGFHTTWGAFSFPFIFLATDLTV
RIFGAPLARRIIFAVMIPALLISYVISSLFYMGSWQGFGALAHFNLFVARIATASFMAYA
LGQILDVHVFNRLRQSRRWWLAPTASTLFGNVSDTLAFFFIAFWRSPDAFMAEHWMEIAL
VDYCFKVLISIVFFLPMYGVLLNMLLKRLADKSEINALQAS
>P69425
MFDIGFSELLLVFIIGLVVLGPQRLPVAVKTVAGWIRALRSLATTVQNELTQELKLQEFQ
DSLKKVEKASLTNLTPELKASMDELRQAAESMKRSYVANDPEKASDEAHTIHNPVVKDNE
AAHEGVTPAAAQTQASSPEQKPETTPEPVVKPAADAEPKTAAPSPSSSDKP
>Q59601
MSSNKASFFTRLRRLCRLTVWLFKTGKNLRGIDGGCPKSRNRAVIALGKGALAALDIGLE
VGRPAPEHPNGVLVAANHVSWLDIFAMSAVYPSSFIAKQEIKSWPVLGKMGQNAGTVFIN
RNSRRDIEPINRAVCETLQRGQNVSFFPEARTSSGLGLLPFKAALFQSAIDAGAKVLAVA
LRYYDETGKRTARPSYADVGLPTCLWRIVSMKKLTIKVDFVCVADAAESEDRYALKDKIE
ESIRAVVADDADIAV
>P13738
MKHLHRFFSSDASGGIILIIAAILAMIMANSGATSGWYHDFLETPVQLRVGSLEINKNML

LWINDALMAVFFLLVGLEVKRELMQGSLASLRQAAFPVIAAIGGMIVPALLYLAFNYADP
ITREGWAIPAATDIAFALGVLALLGSRVPLALKIFLMALAIIDDLGAIIIIALFYTNDLS
MASLGVAAVAIAVLAVLNLCGARRTGVYILVGVVLWTAVLKSGVHATLAGVIVGFFIPLK
EKHGRSPAKRLEHVLHPWVAYLILPLFAFANAGVSLQGVTLDGLTSILPLGIIAGLLIGK
PLGISLFCWLALRLKLAHLPEGTTYQQIMVVGILCGIGFTMSIFIASLAFGSVDPELINW
AKLGILVGSISSAVIGYSWLRVRLRPSV
>P0AEH1
MLSFLWDLASFIVALGVLITVHEFGHFWVARRCGVRVERFSIGFGKALWRRTDKLGTEYV
IALIPLGGYVKMLDERAEPVVPELRHHAFNNKSVGQRAAIIAAGPVANFIFAIFAYWLVF
IIGVPGVRPVVGEIAANSIAAEAQIAPGTELKAVDGIETPDWDAVRLQLVDKIGDESTTI
TVAPFGSDQRRDVKLDLRHWAFEPDKEDPVSSLGIRPRGPQIEPVLENVQPNSAASKAGL
QAGDRIVKVDGQPLTQWVTFVMLVRDNPGKSLALEIERQGSPLSLTLIPESKPGNGKAIG
FVGIEPKVIPLPDEYKVVRQYGPFNAIVEATDKTWQLMKLTVSMLGKLITGDVKLNNLSG
PISIAKGAGMTAELGVVYYLPFLALISVNLGIINLFPLPVLDGGHLLFLAIEKIKGGPVS
ERVQDFCYRIGSILLVLLMGLALFNDFSRL
>Q07282
MNRTVMMALVIIFLDAMGIGIIMPVLPALLREFVGKANVAENYGVLLALYAMMQVIFAPL
LGRWSDRIGRRPVLLLSLLGATLDYALMATASVVWVLYLGRLIAGITGATGAVAASTIAD
VTPEESRTHWFGMMGACFGGGMIAGPVIGGFAGQLSVQAPFMFAAAINGLAFLVSLFILH
ETHNANQVSDELKNETINETTSSIREMISPLSGLLVVFFIIQLIGQIPATLWVLFGEERF
AWDGVMVGVSLAVFGLTHALFQGLAAGFIAKHLGERKAIAVGILADGCGLFLLAVITQSW
MVWPVLLLLACGGITLPALQGIISVRVGQVAQGQLQGVLTSLTHLTAVIGPLVFAFLYSA
TRETWNGWVWIIGCGLYVVALIILRFFHPGRVIHPINKSDVQQRI
>P0A6J3
MVSSFTSAPRSGFYYFAQGWKLVSQPGIRRFVILPLLVNILLMGGAFWWLFTQLDVWIPT
LMSYVPDWLQWLSYLLWPLAVISVLLVFGYFFSTIANWIAAPFNGLLAEQLEARLTGATP
PDTGIFGIMKDVPRIMKREWQKFAWYLPRAIVLLILYFIPGIGQTVAPVLWFLFSAWMLA
IQYCDYPFDNHKVPFKEMRTALRTRKITNMQFGALTSLFTMIPLLNLFIMPVAVCGATAM
WVDCYRDKHAMWR
>P27844
MDAKQTRQGVLLALAAYFIWGIAPAYFKLIYYVPADEILTHRVIWSFFFMVVLMSICRQW
SYLKTLIQTPQKIFMLAVSAVLIGGNWLLFIWAVNNHHMLEASLGYFINPLVNIVLGMIF
LGERFRRMQWLAVILAICGVLVQLWTFGSLPIIALGLAFSFAFYGLVRKKIAVEAQTGML
IETMWLLPVAAIYLFAIADSSTSHMGQNPMSLNLLLIAAGIVTTVPLLCFTAAATRLRLS
TLGFFQYIGPTLMFLLAVTFYGEKPGADKMVTFAFIWVALAIFVMDAIYTQRRTSK
>P0AC23
MKADNPFDLLLPAAMAKVAEEAGVYKATKHPLKTFYLAITAGVFISIAFVFYITATTGTG
TMPFGMAKLVGGICFSLGLILCVVCGADLFTSTVLIVVAKASGRITWGQLAKNWLNVYFG
NLVGALLFVLLMWLSGEYMTANGQWGLNVLQTADHKVHHTFIEAVCLGILANLMVCLAVW
MSYSGRSLMDKAFIMVLPVAMFVASGFEHSIANMFMIPMGIVIRDFASPEFWTAVGSAPE
NFSHLTVMNFITDNLIPVTIGNIIGGGLLVGLTYWVIYLRENDHH
>P0AGK1
MEWSLTQNKLLAFHRLMRTDKPIGALLLLWPTLWALWVATPGVPQLWILAVFVAGVWLMR
AAGCVVNDYADRKFDGHVKRTANRPLPSGAVTEKEARALFVVLVLISFLLVLTLNTMTIL

LSIAALALAWVYPFMKRYTHLPQVVLGAAFGWSIPMAFAAVSESVPLSCWLMFLANILWA
VAYDTQYAMVDRDDDVKIGIKSTAILFGQYDKLIIGILQIGVLALMAIIGELNGLGWGYY
WSILVAGALFVYQQKLIANREREACFKAFMNNNYVGLVLFLGLAMSYWHF
>P75810
MTVNSSRNALKRRTWALFMFFFLPGLLMASWATRTPAIRDILSVSIAEMGGVLFGLSIGS
MSGILCSAWLVKRFGTRNVILVTMSCALIGMMILSLALWLTSPLLFAVGLGVFGASFGSA
EVAINVEGAAVEREMNKTVLPMMHGFYSLGTLAGAGVGMALTAFGVPATVHILLAALVGI
APIYIAIQAIPDGTGKNAADGTQHGEKGVPFYRDIQLLLIGVVVLAMAFAEGSANDWLPL
LMVDGHGFSPTSGSLIYAGFTLGMTVGRFTGGWFIDRYSRVAVVRASALMGALGIGLIIF
VDSAWVAGVSVVLWGLGASLGFPLTISAASDTGPDAPTRVSVVATTGYLAFLVGPPLLGY
LGEHYGLRSAMLVVLALVILAAIVAKAVAKPDTKTQTAMENS
>P0AF98
MIIIRYLVRETLKSQLAILFILLLIFFCQKLVRILGAAVDGDIPANLVLSLLGLGVPEMA
QLILPLSLFLGLLMTLGKLYTESEITVMHACGLSKAVLVKAAMILAVFTAIVAAVNVMWA
GPWSSRHQDEVLAEAKANPGMAALAQGQFQQATNGSSVLFIESVDGSDFKDVFLAQIRPK
GNARPSVVVADSGHLTQLRDGSQVVTLNQGTRFEGTALLRDFRITDFQDYQAIIGHQAVA
LDPNDTDQMDMRTLWNTDTDRARAELNWRITLVFTVFMMALMVVPLSVVNPRQGRVLSML
PAMLLYLLFFLIQTSLKSNGGKGKLDPTLWMWTVNLIYLALAIVLNLWDTVPVRRLRASF
SRKGAV
>P31462
MSRFLICSFALVLLYPAGIDMYLVGLPRIAADLNASEAQLHIAFSVYLAGMAAAMLFAGK
VADRSGRKPVAIPGAALFIIASVFCSLAETSTLFLAGRFLQGLGAGCCYVVAFAILRDTL
DDRRRAKVLSLLNGITCIIPVLAPVLGHLIMLKFPWQSLFWAMAMMGIAVLMLSLFILKE
TRPAAPAASDKPRENSESLLNRFFLSRVVITTLSVSVILTFVNTSPVLLMEIMGFERGEY
ATIMALTAGVSMTVSFSTPFALGIFKPRTLMITSQVLFLAAGITLAVSPSHAVSLFGITL
ICAGFSVGFGVAMSQALGPFSLRAGVASSTLGIAQVCGSSLWIWLAAVVGIGAWNMLIGI
LIACSIVSLLLIMFVAPGRPVAAHEEIHHHA
>P0A6H8
MTTVYTLVSWLAILGYWLLIAGVTLRILMKRRAVPSAMAWLLIIYILPLVGIIAYLAVGE
LHLGKRRAERARAMWPSTAKWLNDLKACKHIFAEENSSVAAPLFKLCERRQGIAGVKGNQ
LQLMTESDDVMQALIRDIQLARHNIEMVFYIWQPGGMADQVAESLMAAARRGIHCRLMLD
SAGSVAFFRSPWPELMRNAGIEVVEALKVNLMRVFLRRMDLRQHRKMIMIDNYIAYTGSM
NMVDPRYFKQDAGVGQWIDLMARMEGPIATAMGIIYSCDWEIETGKRILPPPPDVNIMPF
EQASGHTIHTIASGPGFPEDLIHQALLTAAYSAREYLIMTTPYFVPSDDLLHAICTAAQR
GVDVSIILPRKNDSMLVGWASRAFFTELLAAGVKIYQFEGGLLHTKSVLVDGELSLVGTV
NLDMRSLWLNFEITLAIDDKGFGADLAAVQDDYISRSRLLDARLWLKRPLWQRVAERLFY
FFSPLL
>P07001
MRIGIPRERLTNETRVAATPKTVEQLLKLGFTVAVESGAGQLASFDDKAFVQAGAEIVEG
NSVWQSEIILKVNAPLDDEIALLNPGTTLVSFIWPAQNPELMQKLAERNVTVMAMDSVPR
ISRAQSLDALSSMANIAGYRAIVEAAHEFGRFFTGQITAAGKVPPAKVMVIGAGVAGLAA
IGAANSLGAIVRAFDTRPEVKEQVQSMGAEFLELDFKEEAGSGDGYAKVMSDAFIKAEME
LFAAQAKEVDIIVTTALIPGKPAPKLITREMVDSMKAGSVIVDLAAQNGGNCEYTVPGEI
FTTENGVKVIGYTDLPGRLPTQSSQLYGTNLVNLLKLLCKEKDGNITVDFDDVVIRGVTV

IRAGEITWPAPPIQVSAQPQAAQKAAPEVKTEEKCTCSPWRKYALMALAIILFGWMASVA
PKEFLGHFTVFALACVVGYYVVWNVSHALHTPLMSVTNAISGIIVVGALLQIGQGGWVSF
LSFIAVLIASINIFGGFTVTQRMLKMFRKN
>P69212
MYIYWILLGLAIATEITGTLSMKWASVSEGNGGFILMLVMISLSYIFLSFAVKKIALGVA
YALWEGIGILFITLFSVLLFDESLSLMKIAGLTTLVAGIVLIKSGTRKARKPELEVNHGA
V
>P04846
MKLTTHHLRTGAALLLAGILLAGCDQSSSDAKHIKVGVINGAEQDVAEVAKKVAKEKYGL
DVELVGFSGSLLPNDATNHGELDANVFQHRPFLEQDNQAHGYKLVAVGNTFVFPMAGYSK
KIKTVAQIKEGATVAIPNDPTNLGRALLLLQKEKLITLKEGKGLLPTALDITDNPRHLQI
MELEGAQLPRVLDDPKVDVAIISTTYIQQTGLSPVHDSVFIEDKNSPYVNILVAREDNKN
AENVKEFLQSYQSPEVAKAAETIFNGGAVPGW
>Q05605
MTRTQPSASPTPSRAWRAIAALMFSLVLAPVAMADEPTANASTPAAAAAPATPAAAPAPA
ADGSAPVADAPAAAPVDAPVAVDPGVEALVEDTTLGMAHDLSPWGMYKNADIVVKIVMIG
LAIASIITWTIWIAKGFELMGAKRRLRGEIAQLKKSASLKEASEVSNKEGTLAHTLVHDA
LEEMRLSANTREKEGIKERVAFRLERLVAASGRNMSSGTGVLATIGSTAPFVGLFGTVWG
IMNSFIGIAKTQTTNLAVVAPGIAEALLATALGLVAAIPAVVIYNVFARSIAGYKAQVSD
ASAQVLLLVSRDLDHQGSERAAPHMVKVG
>P0AFA5
MDWLLDVFATWLYGLKVIAITLAVIMFISGLDDFFIDVVYWVRRIKRKLSVYRRYPRMSY
RELYKPDEKPLAIMVPAWNETGVIGNMAELAATTLDYENYHIFVGTYPNDPDTQRDVDEV
CARFPNVHKVVCARPGPTSKADCLNNVLDAITQFERSANFAFAGFILHDAEDVISPMELR
LFNYLVERKDLIQIPVYPFEREWTHFTSMTYIDEFSELHGKDVPVREALAGQVPSAGVGT
CFSRRAVTALLADGDGIAFDVQSLTEDYDIGFRLKEKGMTEIFVRFPVVDEAKEREQRKF
LQHARTSNMICVREYFPDTFSTAVRQKSRWIIGIVFQGFKTHKWTSSLTLNYFLWRDRKG
AISNFVSFLAMLVMIQLLLLLAYESLWPDAWHFLSIFSGSAWLMTLLWLNFGLMVNRIVQ
RVIFVTGYYGLTQGLLSVLRLFWGNLINFMANWRALKQVLQHGDPRRVAWDKTTHDFPSV
TGDTRSLRPLGQILLENQVITEEQLDTALRNRVEGLRLGGSMLMQGLISAEQLAQALAEQ
NGVAWESIDAWQIPSSLIAEMPASVALHYAVLPLRLENDELIVGSEDGIDPVSLAALTRK
VGRKVRYVIVLRGQIVTGLRHWYARRRGHDPRAMLYNAVQHQWLTEQQAGEIWRQYVPHQ
FLFAEILTTLGHINRSAINVLLLRHERSSLPLGKFLVTEGVISQETLDRVLTIQRELQVS
MQSLLLKAGLNTEQVAQLESENEGE
>P0AE34
MNEFFPLASAAGMTVGLAVCALIVGLALAMFFAVWESAKWRPVAWAGSALVTILRGLPEI
LVVLFIYFGSSQLLLTLSDGFTINLGFVQIPVQMDIENFDVSPFLCGVIALSLLYAAYAS
QTLRGALKAVPVGQWESGQALGLSKSAIFFRLVMPQMWRHALPGLGNQWLVLLKDTALVS
LISVNDLMLQTKSIATRTQEPFTWYIVAAAIYLVITLLSQYILKRIDLRATRFERRPS
>P77307
MNSHNITNESLALALMLVVVAILISHKEKLALEKDILWSVGRAIIQLIIVGYVLKYIFSV
DDASLTLLMVLFICFNAAWNAQKRSKYIAKAFISSFIAITVGAGITLAVLILSGSIEFIP
MQVIPIAGMIAGNAMVAVGLCYNNLGQRVISEQQQIQEKLSLGATPKQASAILIRDSIRA
ALIPTVDSAKTVGLVSLPGMMSGLIFAGIDPVKAIKYQIMVTFMLLSTASLSTIIACYLT

YRKFYNSRHQLVVTQLKKK

>P56579

MIETITHGAEWFIGLFQKGGEVFTGMVTGILPLLISLLVIMNALINFIGQHRIERFAQRC
AGNPVSRYLLLPCIGTFVFCNPMTLSLGRFMPEKYKPSYYAAASYSCHSMNGLFPHINPG
ELFVYLGIASGLTTLNLPLGPLAVSYLLVGLVTNFFRGWVTDLTTAIFEKKMGIQLEQKV
HLAGATS

>Q56348

MIDSAKETDRPKHRKRDEVIAFLILAVVIWPILSVAIVGGYGFLVWMSQIIFGPPGPMH

>P0AFQ2

MSNPILSWRRVRALCVKETRQIVRDPSSWLIAVVIPLLLLFIFGYGINLDSSKLRVGILL
EQRSEAALDFTHTMTGSPYIDATISDNRQELIAKMQAGKIRGLVVIPVDFAEQMERANAT
APIQVITDGSEPNTANFVQGYVEGIWQIWQMQRAEDNGQTFEPLIDVQTRYWFNPAAISQ
HFIIPGAVTIIMTVIGAILTSLVVAREWERGTMEALLSTEITRTELLLCKLIPYYFLGML
AMLLCMLVSVFILGVPYRGSLLILFFISSLFLLSTLGMGLLISTITRNQFNAAQVALNAA
FLPSIMLSGFIFQIDSMPAVIRAVTYIIPARYFVSTLQSLFLAGNIPVVLVVNVLFLIAS
AVMFIGLTWLKTKRRLD

>P29920

MAEFWASPYGFALSMLLQGLAVIAFVMGSLIFMVYGDRKIWAAVQMRRGPNVVGPWGLLQ
TFADALKYIVKEIVIPAGADKFVYFLAPFLSMMLALFAFVVIPFDEGWVMANINVGILFI
FAASSLEVYGVIMGGWASNSKYPFLASLRSAAQMISYEVSLGLIIIGIIISTGSMNLTAI
VEAHGGDYGLLNWYWLPHLPMVVLFFVSALAECNRPPFDLVEAESELVAGFMTEYSSTPY
LLFMAGEYIAMYLMCALLSLLFFGGWLSPVPFIADGWWWMVIKMWFWFYMFAMVKAIVPR
YRYDQLMRIGWKVFLPLSLGWVVLVAILARYEILGGFWARFAVGG

>P0AFV0

MDLLIVLTYVALAWAVFKIFRIPVNQWTLATAALGGVFLVSGLILLMNYNHPYTFTAQKA
VIAIPITPQVTGIVTEVTDKNNQLIQKGEVLFKLDPVRYQARVDRLQADLMTATHNIKTL
RAQLTEAQANTTQVSAERDRLFKNYQRYLKGSQAAVNPFSERDIDDARQNFLAQDALVKG
SVAEQAQIQSQLDSMVNGEQSQIVSLRAQLTEAKYNLEQTVIRAPSNGYVTQVLIRPGTY
AAALPLRPVMVFIPEQKRQIVAQFRQNSLLRLKPGDDAEVVFNALPGQVFHGKLTSILPV
VPGGSYQAQGVLQSLTVVPGTDGVLGTIELDPNDDIDALPDGIYAQVAVYSDHFSHVSVM
RKVLLRMTSWMHYLYLDH

>P0ACG4

MKQHKAMIVALIVICITAVVAALVTRKDLCEVHIRTGQTEVAVFTAYESE

>P46889

MSQEYIEDKEVTLTKLSSGRRLLEALLILIVLFAVWLMAALLSFNPSDPSWSQTAWHEPI
HNLGGMPGAWLADTLFFIFGVMAYTIPVIIVGGCWFAWRHQSSDEYIDYFAVSLRIIGVL
ALILTSCGLAAINADDIWYFASGGVIGSLLSTTLQPLLHSSGGTIALLCVWAAGLTLFTG
WSWVTIAEKLGGWILNILTFASNRTRRDDTWVDEDEYEDDEEYEDENHGKQHESRRARIL
RGALARRKRLAEKFINPMGRQTDAALFSGKRMDDDEEITYTARGVAADPDDVLFSGNRAT
QPEYDEYDPLLNGAPITEPVAVAAAATTATQSWAAPVEPVTQTPPVASVDVPPAQPTVAW
QPVPGPQTGEPVIAPAPEGYPQQSQYAQPAVQYNEPLQQPVQPQQPYYAPAAEQPAQQPY
YAPAPEQPVAGNAWQAEEQQSTFAPQSTYQTEQTYQQPAAQEPLYQQPQPVEQQPVVEPE
PVVEETKPARPPLYYFEEVEEKRAREREQLAAWYQPIPEPVKEPEPIKSSLKAPSVAAVP
PVEAAAAVSPLASGVKKATLATGAAATVAAPVFSLANSGGPRPQVKEGIGPQLPRPKRIR

VPTRRELASYGIKLPSQRAAEEKAREAQRNQYDSGDQYNDDEIDAMQQDELARQFAQTQQ
QRYGEQYQHDVPVNAEDADAAAEAELARQFAQTQQQRYSGEQPAGANPFSLDDFEFSPMK
ALLDDGPHEPLFTPIVEPVQQPQQPVAPQQQYQQPQQPVPPQPQYQQPQQPVAPQPQYQQ
PQQPVAPQQQYQQPQQPVAPQQQYQQPQQPVAPQPQDTLLHPLLMRNGDSRPLHKPTTPL
PSLDLLTPPPSEVEPVDTFALEQMARLVEARLADFRIKADVVNYSPGPVITRFELNLAPG
VKAARISNLSRDLARSLSTVAVRVVEVIPGKPYVGLELPNKKRQTVYLREVLDNAKFRDN
PSPLTVVLGKDIAGEPVVADLAKMPHLLVAGTTGSGKSVGVNAMILSMLYKAQPEDVRFI
MIDPKMLELSVYEGIPHLLTEVVTDMKDAANALRWCVNEMERRYKLMSALGVRNLAGYNE
KIAEADRMMRPIPDPYWKPGDSMDAQHPVLKKEPYIVVLVDEFADLMMTVGKKVEELIAR
LAQKARAAGIHLVLATQRPSVDVITGLIKANIPTRIAFTVSSKIDSRTILDQAGAESLLG
MGDMLYSGPNSTLPVRVHGAFVRDQEVHAVVQDWKARGRPQYVDGITSDSESEGGAGGFD
GAEELDPLFDQAVQFVTEKRKASISGVQRQFRIGYNRAARIIEQMEAQGIVSEQGHNGNR
EVLAPPPFD
>Q00185
MKNRNNAVGPQIRAKKPKASKTVPILAGLSLGAGLQTATQYFAHSFQYQAGLGWNINHVY
TPWSILQWAGKWYGQYPDDFMRAASMGMVVSTVGLLGTAVTQMVKANTGKANDYLHGSAR
WADKKDIQAAGLLPRPRTVVELVSGKHPPTSSGVYVGGWQDKDGKFHYLRHNGPEHVLTY
APTRSGKGVGLVVPTLLSWAHSAVITDLKGELWALTAGWRKKHARNKVVRFEPASAQGSA
CWNPLDEIRLGTEYEVGDVQNLATLIVDPDGKGLESHWQKTSQALLVGVILHALYKAKNE
GTPATLPSVDGMLADPNRDVGELWMEMTTYGHVDGQNHPAVGSAARDMMDRPEEESGSVL
STAKSYLALYRDPVVARNVSKSDFRIKQLMHHDDPVSLFIVTQPNDKARLRPLVRVMVNM
IVRLLADKMDFENGRPVAHYKHRLLMMLDEFPSLGKLEILQESLAFVAGYGIKCYLICQD
INQLKSRETGYGHDESITSNCHVQNAYPPNRVETAEHLSKLTGTTTIVKEQITTSGRRTS
ALLGNVSRTFQEVQRPLLTPDECLRMPGPKKSADGSIEEAGDMVVYVAGYPAIYGKQPLY
FKDPIFQARAAVPAPKVSDKLIQTATVEEGEGITI
>P39398
MEKENITIDPRSSFTPSSSADIPVPPDGLVQRSTRIKRIQTTAMLLLFFAAVINYLDRSS
LSVANLTIREELGLSATEIGALLSVFSLAYGIAQLPCGPLLDRKGPRLMLGLGMFFWSLF
QAMSGMVHNFTQFVLVRIGMGIGEAPMNPCGVKVINDWFNIKERGRPMGFFNAASTIGVA
VSPPILAAMMLVMGWRGMFITIGVLGIFLAIGWYMLYRNREHVELTAVEQAYLNAGSVNA
RRDPLSFAEWRSLFRNRTMWGMMLGFSGINYTAWLYLAWLPGYLQTAYNLDLKSTGLMAA
IPFLFGAAGMLVNGYVTDWLVKGGMAPIKSRKICIIAGMFCSAAFTLIVPQATTSMTAVL
LIGMALFCIHFAGTSCWGLIHVAVASRMTASVGSIQNFASFICASFAPIITGFIVDTTHS
FRLALIICGCVTAAGALAYIFLVRQPINDPRKD
>P0AGC0
MLAFLNQVRKPTLDLPLEVRRKMWFKPFMQSYLVVFIGYLTMYLIRKNFNIAQNDMISTY
GLSMTQLGMIGLGFSITYGVGKTLVSYYADGKNTKQFLPFMLILSAICMLGFSASMGSGS
VSLFLMIAFYALSGFFQSTGGSCSYSTITKWTPRRKRGTFLGFWNISHNLGGAGAAGVAL
FGANYLFDGHVIGMFIFPSIIALIVGFIGLRYGSDSPESYGLGKAEELFGEEISEEDKET
ESTDMTKWQIFVEYVLKNKVIWLLCFANIFLYVVRIGIDQWSTVYAFQELKLSKAVAIQG
FTLFEAGALVGTLLWGWLSDLANGRRGLVACIALALIIATLGVYQHASNEYIYLASLFAL
GFLVFGPQLLIGVAAVGFVPKKAIGAADGIKGTFAYLIGDSFAKLGLGMIADGTPVFGLT
GWAGTFAALDIAAIGCICLMAIVAVMEERKIRREKKIQQLTVA
>P26406

MDNIDNKYNPQLCKIFLAISDLIFFNLALWFSLGCVYFIFDQVQRFIPQDQLDTRVITHF
ILSVVCVGWFWIRLRHYTYRKPFWYELKEIFRTIVIFAIFDLALIAFTKWQFSRYVWVFC
WTFALILVPFFRALTKHLLNKLGIWKKKTIILGSGQNARGAYSALQSEEMMGFDVIAFFD
TDASDAEINMLPVIKDTEIIWDLNRTGDVHYILAYEYTELEKTHFWLRELSKHHCRSVTV
VPSFRGLPLYNTDMSFIFSHEVMLLRIQNNLAKRSSRFLKRTFDIVCSIMILIIASPLMI
YLWYKVTRDGGPAIYGHQRVGRHGKLFPCYKFRSMVMNSQEVLKELLANDPIARAEWEKD
FKLKNDPRITAVGRFIRKTSLDELPQLFNVLKGDMSLVGPRPIVSDELERYCDDVDYYLM
AKPGMTGLWQVSGRNDVDYDTRVYFDSWYVKNWTLWNDIAILFKTAKVVLRRDGAY
>P69423
MSVEDTQPLITHLIELRKRLLNCIIAVIVIFLCLVYFANDIYHLVSAPLIKQLPQGSTMI
ATDVASPFFTPIKLTFMVSLILSAPVILYQVWAFIAPALYKHERRLVVPLLVSSSLLFYI
GMAFAYFVVFPLAFGFLANTAPEGVQVSTDIASYLSFVMALFMAFGVSFEVPVAIVLLCW
MGITSPEDLRKKRPYVLVGAFVVGMLLTPPDVFSQTLLAIPMYCLFEIGVFFSRFYVGKG
RNREEENDAEAESEKTEE
>P0AGH1
MMRHLRNIFNLGIKELRSLLGDKAMLTLIVFSFTVSVYSSATVTPGSLNLAPIAIADMDQ
SQLSNRIVNSFYRPWFLPPEMITADEMDAGLDAGRYTFAINIPPNFQRDVLAGRQPDIQV
NVDATRMSQAFTGNGYIQNIINGEVNSFVARYRDNSEPLVSLETRMRFNPNLDPAWFGGV
MAIINNITMLAIVLTGSALIREREHGTVEHLLVMPITPFEIMMAKIWSMGLVVLVVSGLS
LVLMVKGVLGVPIEGSIPLFMLGVALSLFATTSIGIFMGTIARSMPQLGLLVILVLLPLQ
MLSGGSTPRESMPQMVQDIMLTMPTTHFVSLAQAILYRGAGFEIVWPQFLTLMAIGGAFF
TIALLRFRKTIGTMA
>P75962
MSQDSKVFFRIFLGIGLVLILISVVVFYNQFTYSKDAIHTEGVIVDTVWHSSHSHRTGKD
GSWYPVVAFRPTPDYTLIFNSSIGSDFYEDSEGDKVNVYYSPGHPEKAEINNPWVNFFKW
GFIGIMGVIFIAVGLLISMPSSKKSRRKRKSRP
>P39301
MEILYNIFTVFFNQVMTNAPLLLGIVTCLGYILLRKSVSVIIKGTIKTIIGFMLLQAGSG
ILTSTFKPVVAKMSEVYGINGAISDTYASMMATIDRMGDAYSWVGYAVLLALALNICYVL
LRRITGIRTIMLTGHIMFQQAGLIAVTLFIFGYSMWTTIICTAILVSLYWGITSNMMYKP
TQEVTDGCGFSIGHQQQFASWIAYKVAPFLGKKEESVEDLKLPGWLNIFHDNIVSTAIVM
TIFFGAILLSFGIDTVQAMAGKVHWTVYILQTGFSFAVAIFIITQGVRMFVAELSEAFNG
ISQRLIPGAVLAIDCAAIYSFAPNAVVWGFMWGTIGQLIAVGILVACGSSILIIPGFIPM
FFSNATIGVFANHFGGWRAALKICLVMGMIEIFGCVWAVKLTGMSAWMGMADWSILAPPM
MQGFFSIGIAFMAVIIVIALAYMFFAGRALRAEEDAEKQLAEQSA
>P76417
MKTTAKLSFMMFVEWFIWGAWFVPLWLWLSKSGFSAGEIGWSYACTAIAAILSPILVGSI
TDRFFSAQKVLAVLMFAGALLMYFAAQQTTFAGFFPLLLAYSLTYMPTIALTNSIAFANV
PDVERDFPRIRVMGTIGWIASGLACGFLPQILGYADISPTNIPLLITAGSSALLGVFAFF
LPDTPPKSTGKMDIKVMLGLDALILLRDKNFLVFFFCSFLFAMPLAFYYIFANGYLTEVG
MKNATGWMTLGQFSEIFFMLALPFFTKRFGIKKVLLLGLVTAAIRYGFFIYGSADEYFTY
ALLFLGILLHGVSYDFYYVTAYIYVDKKAPVHMRTAAQGLITLCCQGFGSLLGYRLGGVM
MEKMFAYQEPVNGLTFNWSGMWTFGAVMIAIIAVLFMIFFRESDNEITAIKVDDRDIALT
QGEVK

>P0AD65
MKLQNSFRDYTAESALFVRRALVAFLGILLLTGVLIANLYNLQIVRFTDYQTRSNENRIK
LVPIAPSRGIIYDRNGIPLALNRTIYQIEMMPEKVDNVQQTLDALRSVVDLTDDDIAAFR
KERARSHRFTSIPVKTNLTEVQVARFAVNQYRFPGVEVKGYKRRYYPYGSALTHVIGYVS
KINDKDVERLNNDGKLANYAATHDIGKLGIERYYEDVLHGQTGYEEVEVNNRGRVIRQLK
EVPPQAGHDIYLTLDLKLQQYIETLLAGSRAAVVVTDPRTGGVLALVSTPSYDPNLFVDG
ISSKDYSALLNDPNTPLVNRATQGVYPPASTVKPYVAVSALSAGVITRNTTLFDPGWWQL
PGSEKRYRDWKKWGHGRLNVTRSLEESADTFFYQVAYDMGIDRLSEWMGKFGYGHYTGID
LAEERSGNMPTREWKQKRFKKPWYQGDTIPVGIGQGYWTATPIQMSKALMILINDGIVKV
PHLLMSTAEDGKQVPWVQPHEPPVGDIHSGYWELAKDGMYGVANRPNGTAHKYFASAPYK
IAAKSGTAQVFGLKANETYNAHKIAERLRDHKLMTAFAPYNNPQVAVAMILENGGAGPAV
GTLMRQILDHIMLGDNNTDLPAENPAVAAAEDH
>P69797
MTIAIVIGTHGWAAEQLLKTAEMLLGEQENVGWIDFVPGENAETLIEKYNAQLAKLDTTK
GVLFLVDTWGGSPFNAASRIVVDKEHYEVIAGVNIPMLVETLMARDDDPSFDELVALAVE
TGREGVKALKAKPVEKAAPAPAAAAPKAAPTPAKPMGPNDYMVIGLARIDDRLIHGQVAT
RWTKETNVSRIIVVSDEVAADTVRKTLLTQVAPPGVTAHVVDVAKMIRVYNNPKYAGERV
MLLFTNPTDVERLVEGGVKITSVNVGGMAFRQGKTQVNNAVSVDEKDIEAFKKLNARGIE
LEVRKVSTDPKLKMMDLISKIDK
>P77031
MQHNSYRRWITLAIISFSGGVSFDLAYLRYIYQIPMAKFMGFSNTEIGLIMSTFGIAAII
LYAPSGVIADKFSHRKMITSAMIITGLLGLLMATYPPLWVMLCIQIAFAITTILMLWSVS
IKAASLLGDHSEQGKIMGWMEGLRGVGVMSLAVFTMWVFSRFAPDDSTSLKTVIIIYSVV
YILLGILCWFFVSDNNNLRSANNEEKQSFQLSDILAVLRISTTWYCSMVIFGVFTIYAIL
SYSTNYLTEMYGMSLVAASYMGIVINKIFRALCGPLGGIITTYSKVKSPTRVIQILSVLG
LLTLTALLVTNSNPQSVAMGIGLILLLGFTCYASRGLYWACPGEARTPSYIMGTTVGICS
VIGFLPDVFVYPIIGHWQDTLPAAEAYRNMWLMGMAALGMVIVFTFLLFQKIRTADSAPA
MASSK
>P0AD47
MNTLRYFDFGAARPVLLLIARIAVVLIFIIFGFPKMMGFDGTVQYMASLGAPMPMLAAII
AVVMEVPAAILIVLGFFTRPLAVLFIFYTLGTAVIGHHYWDMTGDAVGPNMINFWKNVSI
AGAFLLLAITGPGAISLDRR
>P21365
MSITAQSVYRDTGNFFRNQFMTILLVSLLCAFITVVLGHVFSPSDAQLAQLNDGVPVSGS
SGLFDLVQNMSPEQQQILLQASAASTFSGLIGNAILAGGVILIIQLVSAGQRVSALRAIG
ASAPILPKLFILIFLTTLLVQIGIMLVVVPGIIMAILLALAPVMLVQDKMGVFASMRSSM
RLTWANMRLVAPAVLSWLLAKTLLLLFASSFAALTPEIGAVLANTLSNLISAILLIYLFR
LYMLIRQ
>P25522
MSDNDTIVAQATPPGRGGVGILRISGFKAREVAETVLGKLPKPRYADYLPFKDADGSVLD
QGIALWFPGPNSFTGEDVLELQGHGGPVILDLLLKRILTIPGLRIARPGEFSERAFLNDK
LDLAQAEAIADLIDASSEQAARSALNSLQGAFSARVNHLVEALTHLRIYVEAAIDFPDEE
IDFLSDGKIEAQLNDVIADLDAVRAEARQGSLLREGMKVVIAGRPNAGKSSLLNALAGRE
AAIVTDIAGTTRDVLREHIHIDGMPLHIIDTAGLREASDEVERIGIERAWQEIEQADRVL

FMVDGTTTDAVDPAEIWPEFIARLPAKLPITVVRNKADITGETLGMSEVNGHALIRLSAR
TGEGVDVLRNHLKQSMGFDTNMEGGFLARRRHLQALEQAAEHLQQGKAQLLGAWAGELLA
EELRLAQQNLSEITGEFTSDDLLGRIFSSFCIGK
>P0C2V3
MNPHDLEWLNRIGERKDIMLAVLLLAVVFMMVLPLPPLVLDILIAVNMTISVVLLMIAIY
INSPLQFSAFPAVLLVTTLFRLALSVSTTRMILLQADAGQIVYTFGNFVVGGNFIVGIVI
FLIITIVQFLVITKGSERVAEVSARFSLDAMPGKQMSIDGDMRAGVIDVNEARERRATIE
KESQMFGSMDGAMKFVKGDAIAGLIIIFVNILGGVTIGVTQKGLAAAEALQLYSILTVGD
GMVSQVPALLIAITAGIIVTRVSSEDSSDLGSDIGKQVVAQPKAMLIGGVLLLLFGLIPG
FPTVTFLILALLVGCGGYMLSRKQSRNDEANQDLQSLLTSGSGAPAARTKAKTSGANKGR
LGEQEAFAMTVPLLIDVDSSQQEALEAIALNDELVRVRRALYLDLGVPFPGIHLRFNEGM
GEGEYLISLQEVPVARGELKAGYLLVRESVSQLELLGIPYEKGEHLLPDQETFWVSVEYE
ERLEKSQLEFFSHSQVLTWHLSHVLREYAEDFIGIQETRYLLEQMEGGYGELIKEVQRIV
PLQRMTEILQRLVGEDISIRNMRSILEAMVEWGQKEKDVVQLTEYIRSSLKRYICYKYAN
GNNILPAYLFDQEVEEKIRSGVRQTSAGSYLALDPAVTESLLEQVRKTIGDLSQIQSKPV
LIVSMDIRRYVRKLIESEYYGLPVLSYQELTQQINIQPLGRVCL
>P77354
MSSERDLVNFLGDFSMDVAKAVIAGGVATAIGSLASFACVSFGFPVILVGGAILLTGIVC
TVVLNEIDAQCHLSEKLKYAIRDGLKRQQELDKWKRENMTPFMYVLNTPPVI
>P37748
MIYLVISVFLITAFICLYLKKDIFYPAVCVNIIFALVLLGYEITSDIYAFQLNDATLIFL
LCNVLTFTLSCLLTESVLDLNIRKVNNAIYSIPSKKVHNVGLLVISFSMIYICMRLSNYQ
FGTSLLSYMNLIRDADVEDTSRNFSAYMQPIILTTFALFIWSKKFTNTKVSKTFTLLVFI
VFIFAIILNTGKQIVFMVIISYAFIVGVNRVKHYVYLITAVGVLFSLYMLFLRGLPGGMA
YYLSMYLVSPIIAFQEFYFQQVSNSASSHVFWFFERLMGLLTGGVSMSLHKEFVWVGLPT
NVYTAFSDYVYISAELSYLMMVIHGCISGVLWRLSRNYISVKIFYSYFIYTFSFIFYHES
FMTNISSWIQITLCIIVFSQFLKAQKIK
>P0AAS3
MMELMVVHPHIFWLSLGGLLLAAEMLGGNGYLLWSGVAAVITGLVVWLVPLGWEWQGVMF
AILTLLAAWLWWKWLSRRVREQKHSDSHLNQRGQQLIGRRFVLESPLVNGRGHMRVGDSS
WPVSASEDLGAGTHVEVIAIEGITLHIRAVSS
>P0AE06
MNKNRGFTPLAVVLMLSGSLALTGCDDKQAQQGGQQMPAVGVVTVKTEPLQITTELPGRT
SAYRIAEVRPQVSGIILKRNFKEGSDIEAGVSLYQIDPATYQATYDSAKGDLAKAQAAAN
IAQLTVNRYQKLLGTQYISKQEYDQALADAQQANAAVTAAKAAVETARINLAYTKVTSPI
SGRIGKSNVTEGALVQNGQATALATVQQLDPIYVDVTQSSNDFLRLKQELANGTLKQENG
KAKVSLITSDGIKFPQDGTLEFSDVTVDQTTGSITLRAIFPNPDHTLLPGMFVRARLEEG
LNPNAILVPQQGVTRTPRGDATVLVVGADDKVETRPIVASQAIGDKWLVTEGLKAGDRVV
ISGLQKVRPGVQVKAQEVTADNNQQAASGAQPEQSKS
>P0ABF8
MQFNIPTLLTLFRVILIPFFVLVFYLPVTWSPFAAALIFCVAAVTDWFDGFLARRWNQST
RFGAFLDPVADKVLVAIAMVLVTEHYHSWWVTLPAATMIAREIIISALREWMAELGKRSS
VAVSWIGKVKTTAQMVALAWLLWRPNIWVEYAGIALFFVAAVLTLWSMLQYLSAARADLL
DQ

>P29922
MMTFAFYLFAISACVAGFMVVIGRNPVHSVLWLILAFLSAAGLFVLQGAEFVAMLLVVVY
VGAVAVLFLFVVMMLDVDFAELKGELARYLPLALVIGVVLLAQLGIAFSGWTPSDQAESL
RAAPVDAAVENTLGLGLVLYDRYVLMFQLAGLVLLVAMIGAIVLTMRHRKDVKRQNVLEQ
MWRDPAKTMELKDVKPGQGL
>P22610
MPLLDYLASHPLAFVLCTILLGLLVGSFLNVVVHRLPKMMERNWKAEAREALGLEPEPKQ
ATYNLVLPNSACPRCGHEIRPWENIPLVSYLALGGKCSSCKAAIGKRYPLVELATALLSG
YVAWHFGFTWQAGAMLLLTWGLLAMSLIDADHQLLPDVLVLPLLWLGLIANHFGLFASLD
DALFGAVFGYLSLWSVFWLFKLVTGKEGMGYGDFKLLAMLGAWGGWQILPLTILLSSLVG
AILGVIMLRLRNAESGTPIPFGPYLAIAGWIALLWGDQITRTYLQFAGFK
>P33599
MVNNMTDLTAQEPAWQTRDHLDDPVIGELRNRFGPDAFTVQATRTGVPVVWIKREQLLEV
GDFLKKLPKPYVMLFDLHGMDERLRTHREGLPAADFSVFYHLISIDRNRDIMLKVALAEN
DLHVPTFTKLFPNANWYERETWDLFGITFDGHPNLRRIMMPQTWKGHPLRKDYPARATEF
SPFELTKAKQDLEMEALTFKPEEWGMKRGTENEDFMFLNLGPNHPSAHGAFRIVLQLDGE
EIVDCVPDIGYHHRGAEKMGERQSWHSYIPYTDRIEYLGGCVNEMPYVLAVEKLAGITVP
DRVNVIRVMLSELFRINSHLLYISTFIQDVGAMTPVFFAFTDRQKIYDLVEAITGFRMHP
AWFRIGGVAHDLPRGWDRLLREFLDWMPKRLASYEKAALQNTILKGRSQGVAAYGAKEAL
EWGTTGAGLRATGIDFDVRKARPYSGYENFDFEIPVGGGVSDCYTRVMLKVEELRQSLRI
LEQCLNNMPEGPFKADHPLTTPPPKERTLQHIETLITHFLQVSWGPVMPANESFQMIEAT
KGINSYYLTSDGSTMSYRTRVRTPSFAHLQQIPAAIRGSLVSDLIVYLGSIDFVMSDVDR

>P77510
MLQLNENKQFAFFQRLAFPLRIFLLILVFSIFVIAALAQYFTASFEDYLTLHVRDMAMNQ
AKIIASNDSVISAVKTRDYKRLATIANKLQRDTDFDYVVIGDRHSIRLYHPNPEKIGYPM
QFTKQGALEKGESYFITGKGSMGMAMRAKTPIFDDDGKVIGVVSIGYLVSKIDSWRAEFL
LPMAGVFVVLLGILMLLSWFLAAHIRRQMMGMEPKQIARVVRQQEALFSSVYEGLIAVDP
HGYITAINRNARKMLGLSSPGRQWLGKPIVEVVRPADFFTEQIDEKRQDVVANFNGLSVI
ANREAIRSGDDLLGAIISFRSKDEISTLNAQLTQIKQYVESLRTLRHEHLNWMSTLNGLL
QMKEYDRVLAMVQGESQAQQQLIDSLREAFADRQVAGLLFGKVQRARELGLKMIIVPGSQ
LSQLPPGLDSTEFAAIVGNLLDNAFEASLRSDEGNKIVELFLSDEGDDVVIEVADQGCGV
PESLRDKIFEQGVSTRADEPGEHGIGLYLIASYVTRCGGVITLEDNDPCGTLFSIYIPKV
KPNDSSINPIDR
>P0AG99
MYEALLVVFLIVAIGLVGLIMLQQGKGADMGASFGAGASATLFGSSGSGNFMTRMTALLA
TLFFIISLVLGNINSNKTNKGSEWENLSAPAKTEQTQPAAPAKPTSDIPN
>P38540
MKHVLTLLALASVFAVSNQALAYDGQNCKEPGNCWENKPGYPEKIAGSKYDPKHDPVELN
KQEESIKAMDARNAKRIANAKSSGNFVFDVK
>Q51575
MIPRRSSDITIKTRSDVLPFSGASSRWLQRYAPALLAVALIIAMSISLAWQAAGWLRLQR
SPVAVAASPVSHESIRSDPTRLARLFGTSAQDPNAPPPATNLDLVLKGSFVQSDPKLSSA
IIQRQGDKPHRYAVGGEISDGVKLHAVYRDRVELQRGGRLESLPFPHRSGGLLASADDIT

SENDSIEQLQSLQDENAAALRERLDALRQQMEATPIAEPAEEDSSEPTTTPTESD
>P0AA70
MRFRQLLPLFGALFALYIIWGSTYFVIRIGVESWPPLMMAGVRFLAAGILLLAFLLLRGH
KLPPLRPLLNAALIGLLLLAVGNGMVTVAEHQNVPSGIAAVVVATVPLFTLCFSRLFGIK
TRKLEWVGIAIGLAGIIMLNSGGNLSGNPWGAILILIGSISWAFGSVYGSRITLPVGMMA
GAIEMLAAGVVLMIASMIAGEKLTALPSLSGFLAVGYLALFGSIIAINAYMYLIRNVSPA
LATSYAYVNPVVAVLLGTGLGGETLSKIEWLALGVIVFAVVLVTLGKYLFPAKPVVAPVI
QDASSE
>P76111
MNQSLTLAFLIAAGIGLVVQNTLMVRITQTSSTILIAMLLNSLVGIVLFVSILWFKQGMA
GFGELVSSVRWWTLIPGLLGSFFVFASISGYQNVGAATTIAVLVASQLIGGLMLDIFRSH
GVPLRALFGPICGAILLVVGAWLVARRSF
>P0AD17
MSHVWGLFSHPDREMQVINRENETISHHYTHHVLLMAAIPVICAFIGTTQIGWNFGDGTI
LKLSWFTGLALAVLFYGVMLAGVAVMGRVIWWMARNYPQRPSLAHCMVFAGYVATPLFLS
GLVALYPLVWLCALVGTVALFYTGYLLYLGIPSFLNINKEEGLSFSSSTLAIGVLVLEVL
LALTVILWGYGYRLF
>Q8FJ36
MFVPFLIILREGLEAALIVSLIASYLTRTQRGRWIGVMWIGVLLAAALCLGLGIFINETT
GEFPQKEQELFEGIVAVIAVVILTWMVFWMRKVSRNVKVQLEQAVDSALQRGNHHGWALV
MMVFFAVAREGLESVFFLLAAFQQDVGIWPPLGAMLGLATAVVLGFLLYWGGIRLNLGAF
FKWTSLFILFVAAGLAAGAIRAFHEAGLWNHFQEIAFDMSAVLSTHSLFGTLMEGIFGYQ
EAPSVSEVAVWFIYLIPALVAFVLPPRAGATASRSM
>P76224
MATPLRYALIFLLWAMVAVIYAPLIPAALTLISPALSLTHWQALFADPQLPQALLATLVS
TTIAAVGALLIALLVIVALWPGPKWQRMCARLPWLLAIPHVAFATSALLLFADGGLLYDY
FPYFTPPMDRFGIGLGLTLAVKESAFLLWILAAVLSEKWLLQQVIVLDSLGYSRWQCLNW
LLLPSVAPALAMAMLAIVAWSLSVVDVAIILGPGNPPTLAVISWQWLTQGDIDQQTKGAL
ASLLLMLLLAAYVLLSYLLWRSWRRTIPRVDGVRKPATPLLPGNTLAIFLPLTGVLCVVL
LAILADQSTINSEALINSLTMGLVATFIALLLLLLWLEWGPQRRQLWLWLPILLPALPLV
AGQYTLALWLKLDGSWTAVVWGHLLWVMPWMLFILQPAWQRIDSRLILIAQTLGWSRAKI
FFYVKCPLMLRPVLIAFAVGFAVGIAQYMPTLWLGAGRFPTLTTEAVALSSGGSNGILAA
QALWQLLLPLIIFALTALVAKWVGYVRQGLR
>P0ADR2
MTSRFMLIFAAISGFIFVALGAFGAHVLSKTMGAVEMGWIQTGLEYQAFHTLAILGLAVA
MQRRISIWFYWSSVFLALGTVLFSGSLYCLALSHLRLWAFVTPVGGVSFLAGWALMLVGA
IRLKRKGVSHE
>P33602
MATIHVDGKEYEVNGADNLLEACLSLGLDIPYFCWHPALGSVGACRQCAVKQYQNAEDTR
GRLVMSCMTPASDGTFISIDDEEAKQFRESVVEWLMTNHPHDCPVCEEGGNCHLQDMTVM
TGHSFRRYRFTKRTHRNQDLGPFISHEMNRCIACYRCVRYYKDYADGTDLGVYGAHDNVY
FGRPEDGTLESEFSGNLVEICPTGVFTDKTHSERYNRKWDMQFAPSICQQCSIGCNISPG
ERYGELRRIENRYNGTVNHYFLCDRGRFGYGYVNLKDRPRQPVQRRGDDFITLNAEQAMQ
GAADILRQSKKVIGIGSPRASVESNFALRELVGEENFYTGIAHGEQERLQLALKVLREGG

IYTPALREIESYDAVLVLGEDVTQTGARVALAVRQAVKGKAREMAAAQKVADWQIAAILN
IGQRAKHPLFVTNVDDTRLDDIAAWTYRAPVEDQARLGFAIAHALDNSAPAVDGIEPELQ
SKIDVIVQALAGAKKPLIISGTNAGSLEVIQAAANVAKALKGRGADVGITMIARSVNSMG
LGIMGGGSLEEALTELETGRADAVVVLENDLHRHASAIRVNAALAKAPLVMVVDHQRTAI
MENAHLVLSAASFAESDGTVINNEGRAQRFFQVYDPAYYDSKTVMLESWRWLHSLHSTLL
SREVDWTQLDHVIDAVVAKIPELAGIKDAAPDATFRIRGQKLAREPHRYSGRTAMRANIS
VHEPRQPQDIDTMFTFSMEGNNQPTAHRSQVPFAWAPGWNSPQAWNKFQDEVGGKLRFGD
PGVRLFETSENGLDYFTSVPARFQPQDGKWRIAPYYHLFGSDELSQRAPVFQSRMPQPYI
KLNPADAAKLGVNAGTRVSFSYDGNTVTLPVEIAEGLTAGQVGLPMGMSGIAPVLAGAHL
EDLKEAQQ
>P29040
MRLEMIGLRTWLLATVVGWALLVCVLAVAGLGKRVELLPDDPALVQRLPALPAPAPERLG
PFEKYAEIAAHPAFAEDRLPHPFFLSGNDGSGAASTVRLTGVLLTSTFKMATLTLDPADS
VRVQLGGDAVKGYRLLALQPRSATIEGPGGTQTLELQVFNGQGGQPPTAIGGRPQAPGAV
PPLPPNVPPAPATPAPPPAEVPQQQPGGQAPPTVPPQRSDGAQEAPRPSDEQMRAIRERI
EARRRQLQQQRQGGSTPGQTQ
>P39282
MPHTIKKMSLIGLILMIFTSVFGFANSPSAYYLMGYSAIPFYIFSALLFFIPFALMMAEM
GAAYRKEEGGIYSWMNNSVGPRFAFIGTFMWFSSYIIWMVSTSAKVWVPFSTFLYGSDMT
QHWRIAGLEPTQVVGLLAVAWMILVTVVASKGINKIARITAVGGIAVMCLNLVLLLVSIT
ILLLNGGHFAQDINFLASPNPGYQSGLAMLSFVVFAIFAYGGIEAVGGLVDKTENPEKNF
AKGIVFAAIVISIGYSLAIFLWGVSTNWQQVLSNGSVNLGNITYVLMKSLGMTLGNALHL
SPEASLSLGVWFARITGLSMFLAYTGAFFTLCYSPLKAIIQGTPKALWPEPMTRLNAMGM
PSIAMWMQCGLVTVFILLVSFGGGTASAFFNKLTLMANVSMTLPYLFLALAFPFFKARQD
LDRPFVIFKTHLSAMIATVVVVLVVTFANVFTIIQPVVEAGDWDSTLWMIGGPVFFSLLA
MAIYQNYCSRVAKNPQWAVE
>P0ABA4
MSEFITVARPYAKAAFDFAVEHQSVERWQDMLAFAAEVTKNEQMAELLSGALAPETLAES
FIAVCGEQLDENGQNLIRVMAENGRLNALPDVLEQFIHLRAVSEATAEVDVISAAALSEQ
QLAKISAAMEKRLSRKVKLNCKIDKSVMAGVIIRAGDMVIDGSVRGRLERLADVLQS
>P0AAA7
MSLAKASLWTAASTLVKIGAGLLVGKLLAVSFGPAGLGLAANFRQLITVLGVLAGAGIFN
GVTKYVAQYHDNPQQLRRVVGTSSAMVLGFSTLMALVFVLAAAPISQGLFGNTDYQGLVR
LVALVQMGIAWGNLLLALMKGFRDAAGNALSLIVGSLIGVLAYYVSYRLGGYEGALLGLA
LIPALVVIPAAIMLIKRGVIPLSYLKPSWDNGLAGQLSKFTLMALITSVTLPVAYIMMRK
LLAAQYSWDEVGIWQGVSSISDAYLQFITASFSVYLLPTLSRLTEKRDITREVVKSLKFV
LPAVAAASFTVWLLRDFAIWLLLSNKFTAMRDLFAWQLVGDVLKVGAYVFGYLVIAKASL
RFYILAEVSQFTLLMVFAHWLIPAHGALGAAQAYMATYIVYFSLCCGVFLLWRRRA
>P42628
MEIASNKGVIADASTPAGRAGMSESEWREAIKFDSTDTGWVIMSIGMAIGAGIVFLPVQV
GLMGLWVFLLSSVIGYPAMYLFQRLFINTLAESPECKDYPSVISGYLGKNWGILLGALYF
VMLVIWMFVYSTAITNDSASYLHTFGVTEGLLSDSPFYGLVLICILVAISSRGEKLLFKI
STGMVLTKLLVVAALGVSMVGMWHLYNVGSLPPLGLLVKNAIITLPFTLTSILFIQTLSP
MVISYRSREKSIEVARHKALRAMNIAFGILFVTVFFYAVSFTLAMGHDEAVKAYEQNISA

LAIAAQFISGDGAAWVKVVSVILNIFAVMTAFFGVYLGFREATQGIVMNILRRKMPAEKI
NENLVQRGIMIFAILLAWSAIVLNAPVLSFTSICSPIFGMVGCLIPAWLVYKVPALHKYK
GMSLYLIIVTGLLLCVSPFLAFS
>Q09049
MISESVVDLSRLQFAMTALYHFLFVPLTLGMTFLLAIMESVYVMTGKQVYKDMVKFWGKL
FGINFALGVTTGITMEFQFGTNWAYYSHYVGDIFGAPLAIEGLTAFFLESTFIGMFFFGW
DRLSKIQHLAVTWLVALGSNLSALWILVANGWMQHPVGAEFNFETMRMELVDFGALLLNP
VAQVKFVHTVASGYVTGAVFVLAISSYYLLKKRDLGFARRSFAIASAFGMASILSVIVLG
DESGYEVGEVQKAKLAAIEAEWETHPAPASFTLIGFPNEEEQRTDFAVKIPWVLGIIATR
SLDEQVIGIKDLIADHEARIRNGMVRYGLLEELRAGNKSPEKIAAFNEVKDDLGYGLLLK
KYTPNVVDASEEQIKQAAKDTIPSVASMFWSFRAMVGAGFAMLILFVCAFWASARKNEES
KPWLLKFALYSLPLPWIATQTGWFVAEHGRQPWTIGGVLPTHLSASSLSTGDLWGSLIAL
IAFYTLLLVVEMYLMIRFARLGPSSLHTGRYHFEQLEQHAVKHASPSQADPQQPVNA
>P33650
MKKLTIGLIGNPNSGKTTLFNQLTGSRQRVGNWAGVTVERKEGQFSTTDHQVTLVDLPGT
YSLTTISSQTSLDEQIACHYILSGDADLLINVVDASNLERNLYLTLQLLELGIPCIVALN
MLDIAEKQNIRIEIDALSARLGCPVIPLVSTRGRGIEALKLAIDRYKANENVELVHYAQP
LLNEADSLAKVMPSDIPLKQRRWLGLQMLEGDIYSRAYAGEASQHLDAALARLRNEMDDP
ALHIADARYQCIAAICDVVSNTLTAEPSRFTTAVDKIVLNRFLGLPIFLFVMYLMFLLAI
NIGGALQPLFDVGSVALFVHGIQWIGYTLHFPDWLTIFLAQGLGGGINTVLPLVPQIGMM
YLFLSFLEDSGYMARAAFVMDRLMQALGLPGKSFVPLIVGFGCNVPSVMGARTLDAPRER
LMTIMMAPFMSCGARLAIFAVFAAAFFGQNGALAVFSLYMLGIVMAVLTGLMLKYTIMRG
EATPFVMELPVYHVPHVKSLIIQTWQRLKGFVLRAGKVIIIVSIFLSAFNSFSLSGKIVD
NINDSALASVSRVITPVFKPIGVHEDNWQATVGLFTGAMAKEVVVGTLNTLYTAENIQDE
EFNPAEFNLGEELFSAIDETWQSLKDTFSLSVLMNPIEASKGDGEMGTGAMGVMDQKFGS
AAAAYSYLIFVLLYVPCISVMGAIARESSRGWMGFSILWGLNIAYSLATLFYQVASYSQH
PTYSLVCILAVILFNIVVIGLLRRARSRVDIELLATRKSVSSCCAASTTGDCH
>P23889
MARSGFEVQKVTVEALFLREIRTRFGKFRLGYLWAILEPSAHLLILLGILGYVMHRTMPD
ISFPVFLLNGLIPFFIFSSISKRSIGAIEANQGLFNYRPVKPIDTIIARALLETLIYVAV
YILLMLIVWMTGEYFEITNFLQLVLTWSLLIIILSCGVGLIFMVVGKTFPEMQKVLPILLK
PLYFISCIMFPLHSIPKQYWSYLLWNPLVHVVELSREAVMPGYISEGVSLNYLAMFTLVT
LFIGLALYRTREEAMLTS
>P64585
MADTHHAQGPGKSVLGIGQRIVSIMVEMVETRLRLAVVELEEEKANLFQLLLMLGLTMLF
AAFGLMSLMVLIIWAVDPQYRLNAMIATTVVLLLLALIGGIWTLRKSRKSTLLRHTRHEL
ANDRQLLEEESREQ
>P77858
MRQTLCDGYLVIFALAQAVILLMLTPLFTGISRQIRARMHSRRGPGIWQDYRDIHKLFKR
QEVAPTSSGLMFRLMPWVLISSMLVLAMALPLFITVSPFAGGGDLITLIYLLALFRFFFA
LSGLDTGSPFAGVGASRELTLGILVEPMLILSLLVLALIAGSTHIEMISNTLAMGWNSPL
TTVLALLACGFACFIEMGKIPFDVAEAEQELQEGPLTEYSGAGLALAKWGLGLKQVVMAS
LFVALFLPFGRAQELSLACLLTSLVVTLLKVLLIFVLASIAENTLARGRFLLIHHVTWLG
FSLAALAWVFWLTGL

>P0AAE8
MSSAKKIGLFACTGVVAGNMMGSGIALLPANLASIGGIAIWGWIISIIGAMSLAYVYARL
ATKNPQQGGPIAYAGEISPAFGFQTGVLYYHANWIGNLAIGITAVSYLSTFFPVLNDPVP
AGIACIAIVWVFTFVNMLGGTWVSRLTTIGLVLVLIPVVMTAIVGWHWFDAATYAANWNT
ADTTDGHAIIKSILLCLWAFVGVESAAVSTGMVKNPKRTVPLATMLGTGLAGIVYIAATQ
VLSGMYPSSVMAASGAPFAISASTILGNWAAPLVSAFTAFACLTSLGSWMMLVGQAGVRA
ANDGNFPKVYGEVDSNGIPKKGLLLAAVKMTALMILITLMNSAGGKASDLFGELTGIAVL
LTMLPYFYSCVDLIRFEGVNIRNFVSLICSVLGCVFCFIALMGASSFELAGTFIVSLIIL
MFYARKMHERQSHSMDNHTASNAH
>P60778
MTNSNRIKLTWISFLSYALTGALVIVTGMVMGNIADYFNLPVSSMSNTFTFLNAGILISI
FLNAWLMEIVPLKTQLRFGFLLMVLAVAGLMFSHSLALFSAAMFILGVVSGITMSIGTFL
VTQMYEGRQRGSRLLFTDSFFSMAGMIFPMIAAFLLARSIEWYWVYACIGLVYVAIFILT
FGCEFPALGKHAPKTDAPVEKEKWGIGVLFLSVAALCYILGQLGFISWVPEYAKGLGMSL
NDAGTLVSNFWMSYMVGMWAFSFILRFFDLQRILTVLAGLAAILMYVFNTGTPAHMAWSI
LALGFFSSAIYTTIITLGSQQTKVPSPKLVNFVLTCGTIGTMLTFVVTGPIVEHSGPQAA
LLTANGLYAVVFVMCFLLGFVSRHRQHNTLTSH
>P75685
MEKYLHLLSRGDKIGLTLIRLSIAIVFMWIGLLKFVPYEADSITPFVANSPLMSFFYEHP
EDYKQYLTHEGEYKPEARAWQTANNTYGFSNGLGVVEVIIALLVLANPVNRWLGLLGGLM
AFTTPLVTLSFLITTPEAWVPALGDAHHGFPYLSGAGRLVLKDTLMLAGAVMIMADSARE
ILKQRSNESSSTLKTEY
>P0ABP3
MLTFIELLIGVVVIVGVARYIIKGYSATGVLFVGGLLLLIISAIMGHKVLPSSQASTGYS
ATDIVEYVKILLMSRGGDLGMMIMMLCGFAAYMTHIGANDMVVKLASKPLQYINSPYLLM
IAAYFVACLMSLAVSSATGLGVLLMATLFPVMVNVGISRGAAAAICASPAAIILAPTSGD
VVLAAQASEMSLIDFAFKTTLPISIAAIIGMAIAHFFWQRYLDKKEHISHEMLDVSEITT
TAPAFYAILPFTPIIGVLIFDGKWGPQLHIITILVICMLIASILEFLRSFNTQKVFSGLE
VAYRGMADAFANVVMLLVAAGVFAQGLSTIGFIQSLISIATSFGSASIILMLVLVILTML
AAVTTGSGNAPFYAFVEMIPKLAHSSGINPAYLTIPMLQASNLGRTLSPVSGVVVAVAGM
AKISPFEVVKRTSVPVLVGLVIVIVATELMVPGTAAAVTGK
>P0AAD8
MSTSDSIVSSQTKQSSWRKSDTTWTLGLFGTAIGAGVLFFPIRAGFGGLIPILLMLVLAY
PIAFYCHRALARLCLSGSNPSGNITETVEEHFGKTGGVVITFLYFFAICPLLWIYGVTIT
NTFMTFWENQLGFAPLNRGFVALFLLLLMAFVIWFGKDLMVKVMSYLVWPFIASLVLISL
SLIPYWNSAVIDQVDLGSLSLTGHDGILITVWLGISIMVFSFNFSPIVSSFVVSKREEYE
KDFGRDFTERKCSQIISRASMLMVAVVMFFAFSCLFTLSPANMAEAKAQNIPVLSYLANH
FASMTGTKTTFAITLEYAASIIALVAIFKSFFGHYLGTLEGLNGLVLKFGYKGDKTKVSL
GKLNTISMIFIMGSTWVVAYANPNILDLIEAMGAPIIASLLCLLPMYAIRKAPSLAKYRG
RLDNVFVTVIGLLTILNIVYKLF
>P21503
MSTYTQPVMLLLSGLLLLTLAIAVLNTLVPLWLAQEHMSTWQVGVVSSSYFTGNLVGTLL
TGYVIKRIGFNRSYYLASFIFAAGCAGLGLMIGFWSWLAWRFVAGVGCAMIWVVVESALM
CSGTSRNRGRLLAAYMMVYYVGTFLGQLLVSKVSTELMSVLPWVTGLTLAGILPLLFTRV

LNQQAENHDSTSITSMLKLRQARLGVNGCIISGIVLGSLYGLMPLYLNHKGVSNASIGFW
MAVLVSAGILGQWPIGRLADKFGRLLVLRVQVFVVILGSIAMLSQAAMAPALFILGAAGF
TLYPVAMAWACEKVEHHQLVAMNQALLLSYTVGSLLGPSFTAMLMQNFSDNLLFIMIASV
SFIYLLMLLRNAGHTPKPVAHV
>P03959
MAAQGFLLIATFLLVLMVLARPLGSGLARLINDIPLPGTTGVERVLFRALGVSDREMNWK
QYLCAILGLNMLGLAVLFFMLLGQHYLPLNPQQLPGLSWDLALNTAVSFVTNTNWQSYSG
ETTLSYFSQMAGLTVQNFLSAASGIAVIFALIRAFTRQSMSTLGNAWVDLLRITLWVLVP
VALLIALFFIQQGALQNFLPYQAVNTVEGAQQLLPMGPVASQEAIKMLGTNGGGFFNANS
SHPFENPTALTNFVQMLAIFLIPTALCFAFGEVMGDRRQGRMLLWAMSVIFVICVGVVMW
AEVQGNPHLLALGTDSSINMEGKESRFGVLVSSLFAVVTTAASCGAVIAMHDSFTALGGM
VPMWLMQIGEVVFGGVGSGLYGMMLFVLLAVFIAGLMIGRTPEYLGKKIDVREMKLTALA
ILVTPTLVLMGAALAMMTDAGRSAMLNPGPHGFSEVLYAVSSAANNNGSAFAGLSANSPF
WNCLLAFCMFVGRFGVIIPVMAIAGSLVSKKSQAASSGTLPTHGPLFVGLLIGTVLLVGA
LTFIPALALGPVAEYLS
>P63183
MSTDNKQSLPAITLAAIGVVYGDIGTSPLYTLRECLSGQFGFGVERDAVFGFLSLIFWLL
IFVVSIKYLTFVMRADNAGEGGILTLMSLAGRNTSARTTSMLVIMGLIGGSFFYGEVVIT
PAISVMSAIEGLEIVAPQLDTWIVPLSIIVLTLLFMIQKHGTAMVGKLFAPIMLTWFLIL
AGLGLRSIIANPEVLHALNPMWAVHFFLEYKTVSFIALGAVVLSITGVEALYADMGHFGK
FPIRLAWFTVVLPSLTLNYFGQGALLLKNPEAIKNPFFLLAPDWALIPLLIIAALATVIA
SQAVISGVFSLTRQAVRLGYLSPMRIIHTSEMESGQIYIPFVNWMLYVAVVIVIVSFEHS
SNLAAAYGIAVTGTMVLTSILSTTVARQNWHWNKYFVALILIAFLCVDIPLFTANLDKLL
SGGWLPLSLGTVMFIVMTTWKSERFRLLRRMHEHGNSLEAMIASLEKSPPVRVPGTAVYM
SRAINVIPFALMHNLKHNKVLHERVILLTLRTEDAPYVHNVRRVQIEQLSPTFWRVVASY
GWRETPNVEEVFHRCGLEGLSCRMMETSFFMSHESLILGKRPWYLRLRGKLYLLLQRNAL
RAPDQFEIPPNRVIELGTQVEI
>P20471
MLQNTTQSNLPREPEAKQIDYNDSIRSTYFSIDDLRACGASLAEKGTSALPGFFPFEFRA
RHRENEKEILRVYRATAADVEAGASITPAAEWLLDNHHVVEEAIQEVRRDFPRRFYRQLP
TLSVSGTVIPRTMALAWLYVAHTHSTVTRESITAMVEGFQEHETLKIGELWALPSILRFV
LIENLRRIAIRVERSRGMRRKANEVADQLIRLNDPEGCRTLLVESEALAADNTFIAQLLY
RMRDGSQSSGAVIAWIEERLERRGTDVEEALVAEQNRLSSGNATMSNIIRSLREIDDTDW
AVWFESVSKIDATLREGSDYAALDFGSRNTYRDTIEKLARRSGHSEHEVTEIAIEMVEEA
KAAAAVEAPLQEPNVGSFLVGKQRLALEKRIGYSPSIFQHLIRSVRKLDWFAIAGPNILL
TILAMIVVYAFVSPMDIPSGAKLIMLLLFALPASEGAMGLFNTVFTLFAKPSRLVGYEFL
DGIPEDARTLVVVPCLIAKRDHVDELVRNLEVHYLANPRGEIYFALLSDWADSKSEEAPA
DTDVLEYAKREIASLSARYAYDGKTRFFLLHRRRLYNEAEGVWMGWERKRGKLHELNLLL
RGDRDTSFLQGANMVPEGVQYVMTLDSDTRLMRDAVTKLVGKLYHPINRPVVNPRTQEVV
TGYSLLQPRVTPSLTTGSEASAFQRIFTINRGIDPYVFTVSDVYQDIAGEGSFTGKGLYH
VDAFEAALKSRIEENAVLSHDLLEGSYARCALVTDIELVEDFPIRYEVEMSRQHRWARGD
WQLLPYIFNPKNGLSMLGRWKMYDNLRRSLIPVAWLAASVMGWYYMEPTPALIWQLVLIF
SLFVAPTLSLISGIMPRRNDIVARAHLHTVLSDIRAANAQVALRIVFIAHNAAMMADAIV
RSLYRTFVSRKLMLEWRTAAQVQSAGHSGIGDYFRAMWTAPALALVSLALAAISDTGLPF

IGLPFALIWAASPAVAWFVSQSAETEDQLVVSEEAIEEMRKIARRTWRYFEAFVTAEQNF
LPPDNFQETPQPVLAERTSPTNIGVYLLSVMSARSFGWIGFEETITRLEQTIATIDRMPK
YRGHLFNWYRTRGLEPMEPRYVSSVDSGNLAGHLIAVSSMCREWAEAPSAHVQGNLDGIG
DVAAILKEALNELPDDRKTVRPLRRLVEERIAGFQNALAAVKRERELASIRVINLAVLAR
DMHKLTVNLDHEVRTVQSGEVATWAGSLVAACEAHIADGVFDLGAIEALRQRLLVLKERA
RDIAFSMDFSFLFRPERRLLSIGYRVNANELDEACYDLLASEARLTSLFAIAKGDLPTEH
WYKLGRPIVPIGARGALVSWSGSMFEYLMPPLVMQERQGGILNQTNNLVVQEQINHGRRL
GTPWGISEAAFNARDHELTYQYTNFGVPTLGLKRGLGQNAVIAPYASILACMYDPKSALA
NLARLREVGALGAYGYHDAVDFTPTRVPEGQKCAVVRNYYAHHHGMSVAAVANVVFNGQL
REWFHADPVIEAAELLLQEKAPRDIPVMAAKREPEALGKGQADLLRPEVRVVEDPINQDR
ETVLLSNGHYSVMLTATGAGYARWNGQSVTRWTPDPVEDRTGTFIFLRDTVTGDWWSATA
EPRRAPGEKTVTRFGDDKAEFVKTVGDLTSEVECIVATEHDAEGRRVILLNTGTEDRFIE
VTSYAEPVLAMDDADSSHPTFSKMFLRTEISRHGDVIWVSRNKRSPGDPDIEVAHLVTDN
AGSERHTQAETDRRRFLGQGRTLAEAAAFDPGATLSGTDGFTLDPIVSLRRVVRVPAGKK
VSVIFWTIAAPDREGVDRAIDRYRHPETFNHELIHAWTRSQVQMRHVGITSKEAASFQML
GRYLVYPDMHLRADAETVKTGLASQSALWPLAISGDFPIFCLRINDDGDLGIAREALRAQ
EYLRARGITADLVVVNERASSYAQDLQHTLDSMCENLRLRGLSDGPRQHIFAVRRDLMEP
ETWSTLISASRAVFHARNGTISDQIARATSLYSKSSEKKEEGAEMLLPVIREADARTAVE
LDGGDLDFWNGFGGFAEDGREYAVRLRGGEATPQPWINVISNEQFGFHVSAEGAAFSWSR
NSRDYQLTPWTNDAVVNRPGEAIFVRDMASGAVLTPYAALSRRKSALFETRHGLGYSRFL
STQDELEIEAMHTVHRTLPAKLVRLTIRNRSSAARKLRVYGYAEWVLGNNRSRTAPFVLS
EWDESAKTLVATNPYSIDYPGRCAFFASDGDIAGYTASRREFLGRAGGILAPQAVISGAE
LTGSTDVDGDACAALATDITVEAGVERQVTFFLGDADNPDQVRAVLEELRADSFGAALEA
AKAFWGDFTGVVKVETPDRAFNHMINHWLPYQALGCRIMARSAFYQASGAFGFRDQLQDT
LAFLIHRPALARAQILNAAARQFVEGDVQHWWLPGTDAGVRTMISDDVVWLAHAVAHYCA
VTGEEDILKEKVPFITGPALEEGQHDSFYKPDVADEVGDVYEHCARALDLAIHRTGANGL
PLILGGDWNDGMNRVGEAGEGTSVWLGWFLAGTLRAFLPYARARKDKPRVALWERHLEAL
KDALEQAGWDGDYYRRGYYDDDTPLGSAENGECRIDSIAQSWSTLSGEGDKERSLRAMDA
VMAELVDPEKRIVRLFTPPLETTKQDPGYIKAYPPGVRENGGQYTHAATWVVLAFAAQER
AEEAWRTFRMLNPVSHALSQVDAEHYRVEPYVVAADIYGEGALAGRGGWTWYTGSAGWLY
RAGVEGILGIRKRGDKLLIRPVLPSEWPGYSAEVRVNGTTHRISVSRDSKSGEPVVSVNN
SVTKNAHEGVLL
>P30149
MQALLEHFITQSTVYSLMAVVLVAFLESLALVGLILPGTVLMAGLGALIGSGELSFWHAW
LAGIIGCLMGDWISFWLGWRFKKPLHRWSFLKKNKALLDKTEHALHQHSMFTILVGRFVG
PTRPLVPMVAGMLDLPVAKFITPNIIGCLLWPPFYFLPGILAGAAIDIPAGMQSGEFKWL
LLATAVFLWVGGWLCWRLWRSGKATDRLSHYLSRGRLLWLTPLISAIGVVALVVLIRHPL
MPVYIDILRKVVGV
>P31446
MGIIAQNKISSLGMLFGAIALMMGIIHFSFGPFSAPPPTFESIVADKTAEIKRGLLAGIK
GEKITTVEKKEDVDVDKILNQSGIALAIAALLCAFIGGMRKENRWGIRGALVFGGGTLAF
HTLLFGIGIVCSILLIFLIFSFLTGGSLV
>P31549
MATRRQPLIPGWLIPGVSATTLVVAVALAAFLALWWNAPQDDWVAVWQDSYLWHVVRFSF

WQAFLSALLSVIPAIFLARALYRRRFPGRLALLRLCAMTLILPVLVAVFGILSVYGRQGW
LATLCQSLGLEWTFSPYGLQGILLAHVFFNLPMASRLLLQALENIPGEQRQLAAQLGMRS
WHFFRFVEWPWLRRQIPPVAALIFMLCFASFATVLSLGGGPQATTIELAIYQALSYDYDP
ARAAMLALLQMVCCLGLVLLSQRLSKAIAPGTTLLQGWRDPDDRLHSRICDTVLIVLALL
LLLPPLLAVIVDGVNRQLPEVLAQPVLWQALWTSLRIALAAGVLCVVLTMMLLWSSRELR
ARQKMLAGQVLEMSGMLILAMPGIVLATGFFLLLNNTIGLPQSADGIVIFTNALMAIPYA
LKVLENPMRDITARYSMLCQSLGIEGWSRLKVVELRALKRPLAQALAFACVLSIGDFGVV
ALFGNDDFRTLPFYLYQQIGSYRSQDGAVTALILLLLCFLLFTVIEKLPGRNVKTD
>P62723
MTNITLQKQHRTLWHFIPGLALSAVITGVALWGGSIPAVAGAGFSALTLAILLGMVLGNT
IYPHIWKSCDGGVLFAKQYLLRLGIILYGFRLTFSQIADVGISGIIIDVLTLSSTFLLAC
FLGQKVFGLDKHTSWLIGAGSSICGAAAVLATEPVVKAEASKVTVAVATVVIFGTVAIFL
YPAIYPLMSQWFSPETFGIYIGSTVHEVAQVVAAGHAISPDAENAAVISKMLRVMMLAPF
LILLAARVKQLSGANSGEKSKITIPWFAILFIVVAIFNSFHLLPQSVVNMLVTLDTFLLA
MAMAALGLTTHVSALKKAGAKPLLMALVLFAWLIVGGGAINYVIQSVIA
>P60782
MSAIAPGMILIAYLCGSISSAILVCRLCGLPDPRTSGSGNPGATNVLRIGGKGAAVAVLI
FDVLKGMLPVWGAYELGVSPFWLGLIAIAACLGHIWPVFFGFKGGKGVATAFGAIAPIGW
DLTGVMAGTWLLTVLLSGYSSLGAIVSALIAPFYVWWFKPQFTFPVSMLSCLILLRHHDN
IQRLWRRQETKIWTKFKRKREKDPE
>P76175
MFHRLLIATVVGILAAFAVAGFRHAMLLLEWLFLNNDSGSLVNAATNLSPWRRLLTPALG
GLAAGLLLMGWQKFTQQRPHAPTDYMEALQTDGQFDYAASLVKSLASLLVVTSGSAIGRE
GAMILLAALAASCFAQRFTPRQEWKLWIACGAAAGMAAAYRAPLAGSLFIAEVLFGTMML
ASLGPVIISAVVALLVSNLINHSDALLYNVQLSVTVQARDYALIISTGVLAGLCGPLLLT
LMNACHRGFVSLKLAPPWQLALGGLIVGLLSLFTPAVWGNGYSTVQSFLTAPPLLMIIAG
IFLCKLCAVLASSGSGAPGGVFTPTLFIGLAIGMLYGRSLGLWFPDGEEITLLLGLTGMA
TLLAATTHAPIMSTLMICEMTGEYQLLPGLLIACVIASVISRTLHRDSIYRQHTAQHS
>P06030
MAHVKNHDYQILPPSIWPFFGAIGAFVMLTGAVAWMKGITFFGLPVEGPWMFLIGLVGVL
YVMFGWWADVVNEGETGEHTPVVRIGLQYGFILFIMSEVMFFVAWFWAFIKNALYPMGPD
SPIKDGVWPPEGIVTFDPWHLPLINTLILLLSGVAVTWAHHAFVLEGDRKTTINGLIVAV
ILGVCFTGLQAYEYSHAAFGLADTVYAGAFYMATGFHGAHVIIGTIFLFVCLIRLLKGQM
TQKQHVGFEAAAWYWHFVDVVWLFLFVVIYIWGR
>P0AGF4
MNTQYNSSYIFSITLVATLGGLLFGYDTAVISGTVESLNTVFVAPQNLSESAANSLLGFC
VASALIGCIIGGALGGYCSNRFGRRDSLKIAAVLFFISGVGSAWPELGFTSINPDNTVPV
YLAGYVPEFVIYRIIGGIGVGLASMLSPMYIAELAPAHIRGKLVSFNQFAIIFGQLLVYC
VNYFIARSGDASWLNTDGWRYMFASECIPALLFLMLLYTVPESPRWLMSRGKQEQAEGIL
RKIMGNTLATQAVQEIKHSLDHGRKTGGRLLMFGVGVIVIGVMLSIFQQFVGINVVLYYA
PEVFKTLGASTDIALLQTIIVGVINLTFTVLAIMTVDKFGRKPLQIIGALGMAIGMFSLG
TAFYTQAPGIVALLSMLFYVAAFAMSWGPVCWVLLSEIFPNAIRGKALAIAVAAQWLANY
FVSWTFPMMDKNSWLVAHFHNGFSYWIYGCMGVLAALFMWKFVPETKGKTLEELEALWEP
ETKKTQQTATL

>P52966
MLGLGYIGRKLFGTPNDRKVKRTRPLVAKINALEPAFEKLSDAEIVAKTRELQARAQAGE
SLDALLVEAFANCREAARRALGLRAFDTQLMGGIFLHQGNIAEMKTGEGKTLVATFPAYL
NALAGKGVHIVTVNDYLARRDSEWMGKVYRHLGLTCGVVYPFQPDDEKRAAYGADITYAT
NNELGFDYLRDNMKSSVAEMYQRDHFFAIVDEVDSILIDEARTPLIISGPSQDRSDMYRT
LDAYIPFLTEEHYKLDEKQRNATFTEEGNEFLEQKLQADGLLPEGQSLYDPESTTIVHHI
GQALRAHKLFFKDQNYVVTDDEIVLIDEFTGRMMKGRRLSDGLHQAIEAKERVTIQPENV
TLASVTFQNYFRLYEKLAGMTGTAVTEAEEFGDIYKLGVVEVPTNRPVARKDEHDRVYRT
AKEKYAAVIEAIKTAHEKGQPTLVGTTSIEKSEMLSEMLKAEGLPHNVLNARQHEQEAQI
VADAGRLGAITIATNMAGRGTDIQLGGNVEMKVQEEIAANPEAAPEEIRARIEAEHAAEK
QKVIEAGGLFVLATERHESRRIDNQLRGRSGRQGDPGRSLFFLSLEDDLMRIFGSDRLEG
VLSKLGMKEGEAIIHPWVNKSLERAQAKVEGRNFDWRKQLLKFDDVMNDQRKAVFGQRRE
IMETDEISEIVADMRQQVIDDLIDDFAPPKSYVDQWDIEGMRAAFIDHAGVDLPLADWAA
EEGVDQDVLRERVTAALDAVMAQKTEAFGAETMRVIEKQILLQTIDAKWREHLVTLEHLR
SVVGFRGYAQRDPLSEYKTESFQLFESMLDSLRYEVTKRLGQIRPMSDEERAEMLRQQAA
ALAAAEGAADPAEAPAPQPAAQVALAAAPGFVESDPTTWGEPSRNDPCPCGSGEKFKHCH
GRLA
>P60632
MSKTLNIIWQYLRAFVLIYACLYAGIFIASLLPVTIPGSIIGMLILFVLLALQILPAKWV
NPGCYVLIRYMALLFVPIGVGVMQYFDLLRAQFGPVVVSCAVSTLVVFLVVSWSSQLVHG
ERKVVGQKGSEE
>P77579
MAIKKRSATVVPGASGAAAAVKNPQASKTSFWGELPQHVMSGISRMVPTLIMGGVILAFS
QLIAYSWLKIPAEIGIMDALNSGKFSGFDLSLLKFAWLSQSFGGVLFGFAIPMFAAFVAN
SIGGKLAFPAGFIGGLMSTQPTQLLNFDPSTMQWATSSPVPSTFIGALIISIVAGYLVKW
MNQKIQLPDFLLAFKTTFLLPILSAIFVMLAMYYVITPFGGWINGGIRTVLTAAGEKGAL
MYAMGIAAATAIDLGGPINKAAGFVAFSFTTDHVLPVTARSIAIVIPPIGLGLATIIDRR
LTGKRLFNAQLYPQGKTAMFLAFMGISEGAIPFALESPITAIPSYMVGAIVGSTAAVWLG
AVQWFPESAIWAWPLVTNLGVYMAGIALGAVITALMVVFLRLMMFRKGKLLIDSL
>P31224
MPNFFIDRPIFAWVIAIIIMLAGGLAILKLPVAQYPTIAPPAVTISASYPGADAKTVQDT
VTQVIEQNMNGIDNLMYMSSNSDSTGTVQITLTFESGTDADIAQVQVQNKLQLAMPLLPQ
EVQQQGVSVEKSSSSFLMVVGVINTDGTMTQEDISDYVAANMKDAISRTSGVGDVQLFGS
QYAMRIWMNPNELNKFQLTPVDVITAIKAQNAQVAAGQLGGTPPVKGQQLNASIIAQTRL
TSTEEFGKILLKVNQDGSRVLLRDVAKIELGGENYDIIAEFNGQPASGLGIKLATGANAL
DTAAAIRAELAKMEPFFPSGLKIVYPYDTTPFVKISIHEVVKTLVEAIILVFLVMYLFLQ
NFRATLIPTIAVPVVLLGTFAVLAAFGFSINTLTMFGMVLAIGLLVDDAIVVVENVERVM
AEEGLPPKEATRKSMGQIQGALVGIAMVLSAVFVPMAFFGGSTGAIYRQFSITIVSAMAL
SVLVALILTPALCATMLKPIAKGDHGEGKKGFFGWFNRMFEKSTHHYTDSVGGILRSTGR
YLVLYLIIVVGMAYLFVRLPSSFLPDEDQGVFMTMVQLPAGATQERTQKVLNEVTHYYLT
KEKNNVESVFAVNGFGFAGRGQNTGIAFVSLKDWADRPGEENKVEAITMRATRAFSQIKD
AMVFAFNLPAIVELGTATGFDFELIDQAGLGHEKLTQARNQLLAEAAKHPDMLTSVRPNG
LEDTPQFKIDIDQEKAQALGVSINDINTTLGAAWGGSYVNDFIDRGRVKKVYVMSEAKYR
MLPDDIGDWYVRAADGQMVPFSAFSSSRWEYGSPRLERYNGLPSMEILGQAAPGKSTGEA

MELMEQLASKLPTGVGYDWTGMSYQERLSGNQAPSLYAISLIVVFLCLAALYESWSIPFS
VMLVVPLGVIGALLAATFRGLTNDVYFQVGLLTTIGLSAKNAILIVEFAKDLMDKEGKGL
IEATLDAVRMRLRPILMTSLAFILGVMPLVISTGAGSGAQNAVGTGVMGGMVTATVLAIF
FVPVFFVVVRRRFSRKNEDIEHSHTVDHH
>P46133
MSMSSIPSSSQSGKLYGWVERIGNKVPHPFLLFIYLIIVLMVTTAILSAFGVSAKNPTDG
TPVVVKNLLSVEGLHWFLPNVIKNFSGFAPLGAILALVLGAGLAERVGLLPALMVKMASH
VNARYASYMVLFIAFFSHISSDAALVIMPPMGALIFLAVGRHPVAGLLAAIAGVGCGFTA
NLLIVTTDVLLSGISTEAAAAFNPQMHVSVIDNWYFMASSVVVLTIVGGLITDKIIEPRL
GQWQGNSDEKLQTLTESQRFGLRIAGVVSLLFIAAIALMVIPQNGILRDPINHTVMPSPF
IKGIVPLIILFFFVVSLAYGIATRTIRRQADLPHLMIEPMKEMAGFIVMVFPLAQFVAMF
NWSNMGKFIAVGLTDILESSGLSGIPAFVGLALLSSFLCMFIASGSAIWSILAPIFVPMF
MLLGFHPAFAQILFRIADSSVLPLAPVSPFVPLFLGFLQRYKPDAKLGTYYSLVLPYPLI
FLVVWLLMLLAWYLVGLPIGPGIYPRLS
>P0AFM6
MGIFSRFADIVNANINALLEKAEDPQKLVRLMIQEMEDTLVEVRSTSARALAEKKQLTRR
IEQASAREVEWQEKAELALLKEREDLARAALIEKQKLTDLIKSLEHEVTLVDDTLARMKK
EIGELENKLSETRARQQALMLRHQAANSSRDVRRQLDSGKLDEAMARFESFERRIDQMEA
EAESHSFGKQKSLDDQFAELKADDAISEQLAQLKAKMKQDNQ
>P0AGH5
MPYDSVYSEKRPPGTLRTAWRKFYSDASAMVGLYGCAGLAVLCIFGGWFAPYGIDQQFLG
YQLLPPSWSRYGEVSFFLGTDDLGRDVLSRLLSGAAPTVGGAFVVTLAATICGLVLGTFA
GATHGLRSAVLNHILDTLLAIPSLLLAIIVVAFAGPSLSHAMFAVWLALLPRMVRSIYSM
VHDELEKEYVIAARLDGASTLNILWFAVMPNITAGLVTEITRALSMAILDIAALGFLDLG
AQLPSPEWGAMLGDALELIYVAPWTVMLPGAAIMISVLLVNLLGDGVRRAIIAGVE
>P37327
MDNDKIDQHSDEIEVESEEKERGKKIEIDEDRLPSRAMAIHEHIRQDGEKELERDAMALL
WSAIAAGLSMGASLLAKGIFQVELEGVPGSFLLENLGYTFGFIIVIMARQQLFTENTVTA
VLPVMQKPTMSNVGLLIRLWGVVLLGNILGTGIAAWAFEYMPIFNEETRDAFVKIGMDVM
KNTPSEMFANAIISGWLIATMVWMFPAAGAAKIVVIILMTWLIALGDTTHIVVGSVEILY
LVFNGTLHWSDFIWPFALPTLAGNICGGTFIFALMSHAQIRNDMSNKRKAEARQKAERAE
NIKKNYKNPA
>P0ABN9
MLFTIQLIIILICLFYGARKGGIALGLLGGIGLVILVFVFHLQPGKPPVDVMLVIIAVVA
ASATLQASGGLDVMLQIAEKLLRRNPKYVSIVAPFVTCTLTILCGTGHVVYTILPIIYDV
AIKNNIRPERPMAASSIGAQMGIIASPVSVAVVSLVAMLGNVTFDGRHLEFLDLLAITIP
STLIGILAIGIFSWFRGKDLDKDEEFQKFISVPENREYVYGDTATLLDKKLPKSNWLAMW
IFLGAIAVVALLGADSDLRPSFGGKPLSMVLVIQMFMLLTGALIIILTKTNPASISKNEV
FRSGMIAIVAVYGIAWMAETMFGAHMSEIQGVLGEMVKEYPWAYAIVLLLVSKFVNSQAA
ALAAIVPVALAIGVDPAYIVASAPACYGYYILPTYPSDLAAIQFDRSGTTHIGRFVINHS
FILPGLIGVSVSCVFGWIFAAMYGFL
>P76425
MTEFTTLLQQGNAWFFIPSAILLGALHGLEPGHSKTMMAAFIIAIKGTIKQAVMLGLAAT
ISHTAVVWLIAFGGMVISKRFTAQSAEPWLQLISAVIIISTAFWMFWRTWRGERNWLENM

HGHDYEHHHHDHEHHHDHGHHHHHEHGEYQDAHARAHANDIKRRFDGREVTNWQILLFGL
TGGLIPCPAAITVLLICIQLKALTLGATLVVSFSIGLALTLVTVGVGAAISVQQVAKRWS
GFNTLAKRAPYFSSLLIGLVGVYMGVHGFMGIMR
>P67153
MVQKPLIKQGYSLAEEIANSVSHGIGLVFGIVGLVLLLVQAVDLNASATAITSYSLYGGS
MILLFLASTLYHAIPHQRAKMWLKKFDHCAIYLLIAGTYTPFLLVGLDSPLARGLMIVIW
SLALLGILFKLTIAHRFKILSLVTYLAMGWLSLVVIYEMAVKLAAGSVTLLAVGGVVYSL
GVIFYVCKRIPYNHAIWHGFVLGGSVCHFLAIYLYIGQA
>P39270
MTRTLKPLILNTSALTLTLILIYTGISAHDKLTWLMEVTPVIIVVQLLLATARRYPLTPL
LYTLIFLHAIILMVGGQYTYAKVPVGFEVQEWLGLSRNPYDKLGHFFQGLVPALVAREIL
VRGMYVRGRKMVAFLVCCVALAISAMYELIEWWAALAMGQGADDFLGTQGDQWDTQSDMF
CALLGALTTVIFLARFHCRQLRRFGLITG
>P0A742
MSIIKEFREFAMRGNVVDLAVGVIIGAAFGKIVSSLVADIIMPPLGLLIGGIDFKQFAVT
LRDAQGDIPAVVMHYGVFIQNVFDFLIVAFAIFMAIKLINKLNRKKEEPAAAPAPTKEEV
LLTEIRDLLKEQNNRS
>P06739
MKTWLMGFSEFLLRYKLVWSETWKIRKQLDTPVREKDENEFLPAHLELIETPVSRRPRLV
AYFIMGFLVIAFILSVLGQVEIVATANGKLTHSGRSKEIKPIENSIVKEIIVKEGESVRK
GDVLLKLTALGAEADTLKTQSSLLQARLEQTRYQILSRSIELNKLPELKLPDEPYFQNVS
EEEVLRLTSLIKEQFSTWQNQKYQKELNLDKKRAERLTVLARINRYENLSRVEKSRLDDF
SSLLHKQAIAKHAVLEQENKYVEAVNELRVYKSQLEQIESEILSAKEEYQLVTQLFKNEI
LDKLRQTTDNIGLLTLELAKNEERQQASVIRAPVSVKVQQLKVHTEGGVVTTAETLMVIV
PEDDTLEVTALVQNKDIGFINVGQNAIIKVEAFPYTRYGYLVGKVKNINLDAIEDQRLGL
VFNVIISIEENCLSTGNKNIPLSSGMAVTAEIKTGMRSVISYLLSPLEESVTESLRER
>P0ABC9
MTDLSHSREKDKINPVVFYTSAGLILLFSLTTILFRDFSALWIGRTLDWVSKTFGWYYLL
AATLYIVFVVCIACSRFGSVKLGPEQSKPEFSLLSWAAMLFAAGIGIDLMFFSVAEPVTQ
YMQPPEGAGQTIEAARQAMVWTLFHYGLTGWSMYALMGMALGYFSYRYNLPLTIRSALYP
IFGKRINGPIGHSVDIAAVIGTIFGIATTLGIGVVQLNYGLSVLFDIPDSMAAKAALIAL
SVIIATISVTSGVDKGIRVLSELNVALALGLILFVLFMGDTSFLLNALVLNVGDYVNRFM
GMTLNSFAFDRPVEWMNNWTLFFWAWWVAWSPFVGLFLARISRGRTIRQFVLGTLIIPFT
FTLLWLSVFGNSALYEIIHGGAAFAEEAMVHPERGFYSLLAQYPAFTFSASVATITGLLF
YVTSADSGALVLGNFTSQLKDINSDAPGWLRVFWSVAIGLLTLGMLMTNGISALQNTTVI
MGLPFSFVIFFVMAGLYKSLKVEDYRRESANRDTAPRPLGLQDRLSWKKRLSRLMNYPGT
RYTKQMMETVCYPAMEEVAQELRLRGAYVELKSLPPEEGQQLGHLDLLVHMGEEQNFVYQ
IWPQQYSVPGFTYRARSGKSTYYRLETFLLEGSQGNDLMDYSKEQVITDILDQYERHLNF
IHLHREAPGHSVMFPDA
>P0A9C0
MKTRDSQSSDVIIIGGGATGAGIARDCALRGLRVILVERHDIATGATGRNHGLLHSGARY
AVTDAESARECISENQILKRIARHCVEPTNGLFITLPEDDLSFQATFIRACEEAGISAEA
IDPQQARIIEPAVNPALIGAVKVPDGTVDPFRLTAANMLDAKEHGAVILTAHEVTGLIRE
GATVCGVRVRNHLTGETQALHAPVVVNAAGIWGQHIAEYADLRIRMFPAKGSLLIMDHRI

NQHVINRCRKPSDADILVPGDTISLIGTTSLRIDYNEIDDNRVTAEEVDILLREGEKLAP
VMAKTRILRAYSGVRPLVASDDDPSGRNVSRGIVLLDHAERDGLDGFITITGGKLMTYRL
MAEWATDAVCRKLGNTRPCTTADLALPGSQEPAEVTLRKVISLPAPLRGSAVYRHGDRTP
AWLSEGRLHRSLVCECEAVTAGEVQYAVENLNVNSLLDLRRRTRVGMGTCQGELCACRAA
GLLQRFNVTTSAQSIEQLSTFLNERWKGVQPIAWGDALRESEFTRWVYQGLCGLEKEQKD
AL

>P37433
MKGRLLQRLRQLSISNSLRGAFLTGALLTLIVSMVSLYSWHEQSSQVRYSLDEYFPRIHS
AFLIEGNLNLAVDQLNEFLLAPNTTVRLQLRTQIIQHLDKIERLSQGLQLAERRQLAVIL
QDSRTLLAELDNALYNMFLVREKVSELSARIDWLHDDFTTELNSLVQDFTWQQGTLLDQI
EANQGDAAQYLQRSREVQNEQQQVYTLARIENQIVDDLRDRLNELKSGNNDGMLVETHIR
YLENLKKTADENIRALDDWPSTITLRQTIDELLEIGMVKNKMPDTMRDYVAAQKALLDAS
RAREATLGRFRTLLEAQLGSSHQQMQTFNQRLEQIVRVSGGLILVATLLALLLAWGLNHY
FIRSRLVKRFTALNQAVVQIGLGRTDSTIPVYGRDELGRIARLLRHTLGQLNMQRRQLEQ
EVAERKEIEADLRAMQDELIQTAKLAVVGQTMTTLAHEINQPLNALSMYLFTAGRAIEQG
QSGQARNTLTKAEGLINRIDAIIRSLRQFTRRAELETPLYPVDLRQTFVAAWELLAMRHQ
SRQGALSLPTDTVWVSGDEVRIQQVLVNVLANALDACSHDAVIAVTWQTQGEALEVYIAD
NGPGWPVALLPSLLKPFTTSKAVGLGIGLSISVSLMAQMKGDLRLASTLTRNACVVLQFS
VTDVDDVE

>P0AER3
MSIDWNWGIFLQQAPFGNTTYLGWIWSGFQVTIALSICAWIIAFLVGSFFGILRTVPNRF
LSGLGTLYVELFRNVPLIVQFFTWYLVIPELLPEKIGMWFKAELDPNIQFFLSSMLCLGL
FTAARVCEQVRAAIQSLPRGQKNAALAMGLTLPQAYRYVLLPNAYRVIVPPMTSEMMNLV
KNSAIASTIGLVDMAAQAGKLLDYSAHAWESFTAITLAYVLINAFIMLVMTLVERKVRLP
GNMGGK

>P28785
MENTSVNKEPSIIVGGFVLGGAMIGAGMFSLPTIMSGAWFINSLFILFIVCFFMFHSGIY
ILECISKYGAGTNYFDISKELLPKWACYIANASLIFVLYILIYAYISAAGSIIYEASLLY
GINFNLRAIFFIFTIALGATIWWGGACASRLTSIFLFIKIVLFILAFSGLFFKAKGDLLF
SATFAGKSQLYLYPFIFIIIPYAITSFGYHGNVCSLYKLYNQNERKVVKSCIIGCLLALV
IYLLWMIGTMGNLPREQFITIIQKGGNLDAFIDSLYTVLNSKYIEGFLLWFSISAVFCSF
LGVAIGLFDYILASLKFKDNKTGRLKSGVLCFTPPLLLCLFFPNGFLIAIAYAGTAACVW
AIICPAVMALKARQKFPNSGFKVWGGKKLIYAVIAFGVVGIICQSWRNLIYCLFIVK

>P0AC44
MVSNASALGRNGVHDFILVRATAIVLTLYIIYMVGFFATSGELTYEVWIGFFASAFTKVF
TLLALFSILIHAWIGMWQVLTDYVKPLALRLMLQLVIVVALVVYVIYGFVVVWGV

>P76180
MTTTTPQRIGGWLLGPLAWLLVALLSTTLALLLYTAALSSPQTFQTLGGQALTTQILWGV
SFITAIALWYYTLWLTIAFFKRRRCVPKHYIIWLLISVLLAVKAFAFSPVEDGIAVRQLL
FTLLATALIVPYFKRSSRVKATFVNP

>P0AF54
MNGTIYQRIEDNAHFRELVEKRQRFATILSIIMLAVYIGFILLIAFAPGWLGTPLNPNTS
VTRGIPIGVGVIVISFVLTGIYIWRANGEFDRLNNEVLHEVQAS

>P36672

MMSKINQTDIDRLIELVGGRGNIATVSHCITRLRFVLNQPANARPKEIEQLPMVKGCFTN
AGQFQVVIGTNVGDYYQALIASTGQAQVDKEQVKKAARHNMKWHEQLISHFAVIFFPLLP
ALISGGLILGFRNVIGDLPMSNGQTLAQMYPSLQTIYDFLWLIGEAIFFYLPVGICWSAV
KKMGGTPILGIVLGVTLVSPQLMNAYLLGQQLPEVWDFGMFSIAKVGYQAQVIPALLAGL
ALGVIETRLKRIVPDYLYLVVVPVCSLILAVFLAHALIGPFGRMIGDGVAFAVRHLMTGS
FAPIGAALFGFLYAPLVITGVHQTTLAIDLQMIQSMGGTPVWPLIALSNIAQGSAVIGII
ISSRKHNEREISVPAAISAWLGVTEPAMYGINLKYRFPMLCAMIGSGLAGLLCGLNGVMA
NGIGVGGLPGILSIQPSYWQVFALAMAIAIIIPIVLTSFIYQRKYRLGTLDIV
>P77306
MDDIVNSVPSWMFTAIIAVCILFIIGIIFARLYRRASAEQAFVRTGLGGQKVVMSGGAIV
MPIFHEIIPINMNTLKLEVSRSTIDSLITKDRMRVDVVVAFFVRVKPSVEGIATAAQTLG
QRTLSPEDLRMLVEDKFVDALRATAAQMTMHELQDTRENFVQGVQNTVAEDLSKNGLELE
SVSLTNFNQTSKEHFNPNNAFDAEGLTKLTQETERRRRERNEVEQDVEVAVREKNRDALS
RKLEIEQQEAFMTLEQEQQVKTRTAEQNARIAAFEAERRREAEQTRILAERQIQETEIDR
EQAVRSRKVEAEREVRIKEIEQQQVTEIANQTKSIAIAAKSEQQSQAEARANLALAEAVS
AQQNVETTRQTAEADRAKQVALIAAAQDAETKAVELTVRAKAEKEAAEMQAAAIVELAEA
TRKKGLAEAEAQRALNDAINVLSDEQTSLKFKLALLQALPAVIEKSVEPMKSIDGIKIIQ
VDGLNRGGAAGDANTGNVGGGNLAEQALSAALSYRTQAPLIDSLLNEIGVSGGSLAALTS
PLTSTTPVEEKAE
>P15070
MSDMNNPADDNNGAMDDLWAEALSEQKSTSSKSAAETVFQQFGGGDVSGTLQDIDLIMDI
PVKLTVELGRTRMTIKELLRLTQGSVVALDGLAGEPLDILINGYLIAQGEVVVVADKYGV
RITDIITPSERMRRLSR
>P69432
MNNLIITTRQSPVRLLVDYVATTILWTLFALFIFLFAMDLLTGYYWQSEARSRLQFYFLL
AVANAVVLIVWALYNKLRFQKQQHHAAYQYTPQEYAESLAIPDELYQQLQKSHRMSVHFT
SQGQIKMVVSEKALVRA
>P23894
MMRIALFLLTNLAVMVVFGLVLSLTGIQSSSVQGLMIMALLFGFGGSFVSLLMSKWMALR
SVGGEVIEQPRNERERWLVNTVATQARQAGIAMPQVAIYHAPDINAFATGARRDASLVAV
STGLLQNMSPDEAEAVIAHEISHIANGDMVTMTLIQGVVNTFVIFISRILAQLAAGFMGG
NRDEGEESNGNPLIYFAVATVLELVFGILASIITMWFSRHREFHADAGSAKLVGREKMIA
ALQRLKTSYEPQEATSMMALCINGKSKSLSELFMTHPPLDKRIEALRTGEYLK
>Q05490
MAQADRPARGGLAARPMRGASFALAGLVACAACAAVVLWLRPAAPSPAPAGAVAGGPAAG
VPAAASGAAEAAMPLPAALPGALAGSHAPRLPLAAGGRLARTRAVREFFDYCLTAQGELT
PAALDALVRREIAAQLDGSPAQAEALGVWRRYRAYFDALAQLPGDGAVLGDKLDPAAMQL
ALDQRAALADRTLGEWAEPFFGDEQRRQRHDLERIRIANDTTLSPEQKAARLAALDAQLT
PDERAQQAALHAQQDAVTKIADLQKAGATPDQMRAQIAQTLGPEAAARAAQMQQDDEAWQ
TRYQAYAAERDRIAAQGLAPQDRDARIAQLRQQTFTAPGEAIRAASLDRGAGG
>P0A6A0
MTPGEVRRLYFIIRTFLSYGLDELIPKMRITLPLRLWRYSLFWMPNRHKDKLLGERLRLA
LQELGPVWIKFGQMLSTRRDLFPPHIADQLALLQDKVAPFDGKLAKQQIEAAMGGLPVEA
WFDDFEIKPLASASIAQVHTARLKSNGKEVVIKVIRPDILPVIKADLKLIYRLARWVPRL

LPDGRRLRPTEVVREYEKTLIDELNLLRESANAIQLRRNFEDSPMLYIPEVYPDYCSEGM
MVMERIYGIPVSDVAALEKNGTNMKLLAERGVQVFFTQVFRDSFFHADMHPGNIFVSYEH
PENPKYIGIDCGIVGSLNKEDKRYLAENFIAFFNRDYRKVAELHVDSGWVPPDTNVEEFE
FAIRTVCEPIFEKPLAEISFGHVLLNLFNTARRFNMEVQPQLVLLQKTLLYVEGVGRQLY
PQLDLWKTAKPFLESWIKDQVGIPALVRAFKEKAPFWVEKMPELPELVYDSLRQGKYLQH
SVDKIARELQSNHVRQGQSRYFLGIGATLVLSGTFLLVSRPEWGLMPGWLMAGGLIAWFV
GWRKTR
>P0A6J5
MRVVILGSGVVGVASAWYLNQAGHEVTVIDREPGAALETSAANAGQISPGYAAPWAAPGV
PLKAIKWMFQRHAPLAVRLDGTQFQLKWMWQMLRNCDTSHYMENKGRMVRLAEYSRDCLK
ALRAETNIQYEGRQGGTLQLFRTEQQYENATRDIAVLEDAGVPYQLLESSRLAEVEPALA
EVAHKLTGGLQLPNDETGDCQLFTQNLARMAEQAGVKFRFNTPVDQLLCDGEQIYGVKCG
DEVIKADAYVMAFGSYSTAMLKGIVDIPVYPLKGYSLTIPIAQEDGAPVSTILDETYKIA
ITRFDNRIRVGGMAEIVGFNTELLQPRRETLEMVVRDLYPRGGHVEQATFWTGLRPMTPD
GTPVVGRTRFKNLWLNTGHGTLGWTMACGSGQLLSDLLSGRTPAIPYEDLSVARYSRGFT
PSRPGHLHGAHS
>P76242
MTCSTSLSGKNRIVLIAGILMIATTLRVTFTGAAPLLDTIRSAYSLTTAQTGLLTTLPLL
AFALISPLAAPVARRFGMERSLFAALLLICAGIAIRSLPSPYLLFGGTAVIGGGIALGNV
LLPGLIKRDFPHSVARLTGAYSLTMGAAAALGSAMVVPLALNGFGWQGALLMLMCFPLLA
LFLWLPQWRSQQHANLSTSRALHTRGIWRSPLAWQVTLFLGINSLVYYVIIGWLPAILIS
HGYSEAQAGSLHGLLQLATAAPGLLIPLFLHHVKDQRGIAAFVALMCAVGAVGLCFMPAH
AITWTLLFGFGSGATMILGLTFIGLRASSAHQAAALSGMAQSVGYLLAACGPPLMGKIHD
ANGNWSVPLMGVAILSLLMAIFGLCAGRDKEIR
>P0AD30
METFNSLFMVSPLLLGVLFFVAMLAGFIDSIAGGGGLLTIPALMAAGMSPANALATNKLQ
ACGGSISATIYFIRRKVVSLSDQKLNIAMTFVGSMSGALLVQYVQADVLRQILPILVICI
GLYFLLMPKLGEEDRQRRMYGLPFALIAGGCVGFYDGFFGPAAGSFYALAFVTLCGFNLA
KATAHAKLLNATSNIGGLLLFILGGKVIWATGFVMLVGQFLGARMGSRLVLSKGQKLIRP
MIVIVSAVMSAKLLYDSHGQEILHWLGMN
>P33695
MPNETLHIDIGVCTYRRPELAETLRSLAAMNVPERARLRVIVADNDAEPSARALVEGLRP
EMPFDILYVHCPHSNISIARNCCLDNSTGDFLAFLDDDETVSGDWLTRLLETARTTGAAA
VLGPVRAHYGPTAPRWMRSGDFHSTLPVWAKGEIRTGYTCNALLRRDAASLLGRRFKLSL
GKSGGEDTDFFTGMHCAGGTIAFSPEAWVHEPVPENRASLAWLAKRRFRSGQTHGRLLAE
KAHGLRQAWNIALAGAKSGFCATAAVLCFPSAARRNRFALRAVLHAGVISGLLGLKEIEQ
YGAREVTSA
>Q59659
MADVNRGNRPLSPHLQVYRLPLAAITSIMTRITGHALVAGIVLITWWLVAAVTSPGAFAC
ADWVVRSWLGFIILTGSMWALWYHLLAGLRHLFYDAGYGLEIEQAHKSSQALIAGSVVLA
VLTLIVFFVF
>P0ABG4
MRLSLPRLKMPRLPGFSILVWISTALKGWVMGSREKDTDSLIMYDRTLLWLTFGLAAIGF
IMVTSASMPIGQRLTNDPFFFAKRDGVYLILAFILAIITLRLPMEFWQRYSATMLLGSII

LLMIVLVVGSSVKGASRWIDLGLLRIQPAELTKLSLFCYIANYLVRKGDEVRNNLRGFLK
PMGVILVLAVLLLAQPDLGTVVVLFVTTLAMLFLAGAKLWQFIAIIGMGISAVVLLILAE
PYRIRRVTAFWNPWEDPFGSGYQLTQSLMAFGRGELWGQGLGNSVQKLEYLPEAHTDFIF
AIIGEELGYVGVVLALLMVFFVAFRAMSIGRKALEIDHRFSGFLACSIGIWFSFQALVNV
GAAAGMLPTKGLTLPLISYGGSSLLIMSTAIMMLLRIDYETRLEKAQAFVRGSR
>P09391
MLMITSFANPRVAQAFVDYMATQGVILTIQQHNQSDVWLADESQAERVRAELARFLENPA
DPRYLAASWQAGHTGSGLHYRRYPFFAALRERAGPVTWVMMIACVVVFIAMQILGDQEVM
LWLAWPFDPTLKFEFWRYFTHALMHFSLMHILFNLLWWWYLGGAVEKRLGSGKLIVITLI
SALLSGYVQQKFSGPWFGGLSGVVYALMGYVWLRGERDPQSGIYLQRGLIIFALIWIVAG
WFDLFGMSMANGAHIAGLAVGLAMAFVDSLNARKRK
>P0ABB8
MFKEIFTRLIRHLPSRLVHRDPLPGAQQTVNTVVPPSLSAHCLKMAVMPEEELWKTFDTH
PEGLNQAEVESAREQHGENKLPAQQPSPWWVHLWVCYRNPFNILLTILGAISYATEDLFA
AGVIALMVAISTLLNFIQEARSTKAADALKAMVSNTATVLRVINDKGENGWLEIPIDQLV
PGDIIKLAAGDMIPADLRILQARDLFVAQASLTGESLPVEKAATTRQPEHSNPLECDTLC
FMGTTVVSGTAQAMVIATGANTWFGQLAGRVSEQESEPNAFQQGISRVSMLLIRFMLVMA
PVVLLINGYTKGDWWEAALFALSVAVGLTPEMLPMIVTSTLARGAVKLSKQKVIVKHLDA
IQNFGAMDILCTDKTGTLTQDKIVLENHTDISGKTSERVLHSAWLNSHYQTGLKNLLDTA
VLEGTDEESARSLASRWQKIDEIPFDFERRRMSVVVAENTEHHQLVCKGALQEILNVCSQ
VRHNGEIVPLDDIMLRKIKRVTDTLNRQGLRVVAVATKYLPAREGDYQRADESDLILEGY
IAFLDPPKETTAPALKALKASGITVKILTGDSELVAAKVCHEVGLDAGEVVIGSDIETLS
DDELANLAQRTTLFARLTPMHKERIVTLLKREGHVVGFMGDGINDAPALRAADIGISVDG
AVDIAREAADIILLEKSLMVLEEGVIEGRRTFANMLKYIKMTASSNFGNVFSVLVASAFL
PFLPMLPLHLLIQNLLYDVSQVAIPFDNVDDEQIQKPQRWNPADLGRFMIFFGPISSIFD
ILTFCLMWWVFHANTPETQTLFQSGWFVVGLLSQTLIVHMIRTRRVPFIQSCASWPLMIM
TVIVMIVGIALPFSPLASYLQLQALPLSYFPWLVAILAGYMTLTQLVKGFYSRRYGWQ
>P06974
MGDSILSQAEIDALLNGDSEVKDEPTASVSGESDIRPYDPNTQRRVVRERLQALEIINER
FARHFRMGLFNLLRRSPDITVGAIRIQPYHEFARNLPVPTNLNLIHLKPLRGTGLVVFSP
SLVFIAVDNLFGGDGRFPTKVEGREFTHTEQRVINRMLKLALEGYSDAWKAINPLEVEYV
RSEMQVKFTNITTSPNDIVVNTPFHVEIGNLTGEFNICLPFSMIEPLRELLVNPPLENSR
NEDQNWRDNLVRQVQHSQLELVANFADISLRLSQILKLNPGDVLPIEKPDRIIAHVDGVP
VLTSQYGTLNGQYALRIEHLINPILNSLNEEQPK
>P39314
MPVMISGVLYALLAGLMWGLIFVGPLIVPEYPAMLQSMGRYLALGLIALPIAWLGRVRLR
QLARRDWLTALMLTMMGNLIYYFCLASAIQRTGAPVSTMIIGTLPVVIPVFANLLYSQRD
GKLAWGKLAPALICIGIGLACVNIAELNHGLPDFDWARYTSGIVLALVSVVCWAWYALRN
ARWLRENPDKHPMMWATAQALVTLPVSLIGYLVACYWLNTQTPDFSLPFGPRPLVFISLM
VAIAVLCSWVGALCWNVASQLLPTVILGPLIVFETLAGLLYTFLLRQQMPPLMTLSGIAL
LVIGVVIAVRAKPEKPLTESVSES
>P41036
MSTTTQNIPWYRHLNRAQWRAFSAAWLGYLLDGFDFVLIALVLTEVQGEFGLTTVQAASL
ISAAFISRWFGGLMLGAMGDRYGRRLAMVTSIVLFSAGTLACGFAPGYITMFIARLVIGM

GMAGEYGSSATYVIESWPKHLRNKASGFLISGFSVGAVVAAQVYSLVVPVWGWRALFFIG
ILPIIFALWLRKNIPEAEDWKEKHAGKAPVRTMVDILYRGEHRIANIVMTLAAATALWFC
FAGNLQNAAIVAVLGLLCAAIFISFMVQSAGKRWPTGVMLMVVVLFAFLYSWPIQALLPT
YLKTDLAYNPHTVANVLFFSGFGAAVGCCVGGFLGDWLGTRKAYVCSLLASQLLIIPVFA
IGGANVWVLGLLLFFQQMLGQGIAGILPKLIGGYFDTDQRAAGLGFTYNVGALGGALAPI
IGALIAQRLDLGTALASLSFSLTFVVILLIGLDMPSRVQRWLRPEALRTHDAIDGKPFSG
AVPFGSAKNDLVKTKS
>P0ABB0
MQLNSTEISELIKQRIAQFNVVSEAHNEGTIVSVSDGVIRIHGLADCMQGEMISLPGNRY
AIALNLERDSVGAVVMGPYADLAEGMKVKCTGRILEVPVGRGLLGRVVNTLGAPIDGKGP
LDHDGFSAVEAIAPGVIERQSVDQPVQTGYKAVDSMIPIGRGQRELIIGDRQTGKTALAI
DAIINQRDSGIKCIYVAIGQKASTISNVVRKLEEHGALANTIVVVATASESAALQYLAPY
AGCAMGEYFRDRGEDALIIYDDLSKQAVAYRQISLLLRRPPGREAFPGDVFYLHSRLLER
AARVNAEYVEAFTKGEVKGKTGSLTALPIIETQAGDVSAFVPTNVISITDGQIFLETNLF
NAGIRPAVNPGISVSRVGGAAQTKIMKKLSGGIRTALAQYRELAAFSQFASDLDDATRKQ
LDHGQKVTELLKQKQYAPMSVAQQSLVLFAAERGYLADVELSKIGSFEAALLAYVDRDHA
PLMQEINQTGGYNDEIEGKLKGILDSFKATQSW
>P0AF16
MNLLKSLAAVSSMTMFSRVLGFARDAIVARIFGAGMATDAFFVAFKLPNLLRRIFAEGAF
SQAFVPILAEYKSKQGEDATRVFVSYVSGLLTLALAVVTVAGMLAAPWVIMVTAPGFADT
ADKFALTSQLLKITFPYILLISLASLVGAILNTWNRFSIPAFAPTLLNISMIGFALFAAP
YFNPPVLALAWAVTVGGVLQLVYQLPHLKKIGMLVLPRINFHDAGAMRVVKQMGPAILGV
SVSQISLIINTIFASFLASGSVSWMYYADRLMEFPSGVLGVALGTILLPSLSKSFASGNH
DEYNRLMDWGLRLCFLLALPSAVALGILSGPLTVSLFQYGKFTAFDALMTQRALIAYSVG
LIGLIVVKVLAPGFYSRQDIKTPVKIAIVTLILTQLMNLAFIGPLKHAGLSLSIGLAACL
NASLLYWQLRKQKIFTPQPGWMAFLLRLVVAVLVMSGVLLGMLHIMPEWSLGTMPWRLLR
LMAVVLAGIAAYFAALAVLGFKVKEFARRTV
>P77804
MNKSLVAVGVIVALGVVWTGGAWYTGKKIETHLEDMVAQANAQLKLTAPESNLEVSYQNY
HRGVFSSQLQLLVKPIAGKENPWIKSGQSVIFNESVDHGPFPLAQLKKLNLIPSMASIQT
TLVNNEVSKPLFDMAKGETPFEINSRIGYSGDSSSDISLKPLNYEQKDEKVAFSGGEFQL
NADRDGKAISLSGEAQSGRIDAVNEYNQKVQLTFNNLKTDGSSTLASFGERVGNQKLSLE
KMTISVEGKELALLEGMEISGKSDLVNDGKTINSQLDYSLNSLKVQNQDLGSGKLTLKVG
QIDGEAWHQFSQQYNAQTQALLAQPEIANNPELYQEKVTEAFFSALPLMLKGDPVITIAP
LSWKNSQGESALNLSLFLKDPATTKEAPQTLAQEVDRSVKSLDAKLTIPVDMATEFMTQV
AKLEGYQEDQAKKLAKQQVEGASAMGQMFRLTTLQDNTITTSLQYANGQITLNGQKMSLE
DFVGMFAMPALNVPAVPAIPQQ
>P0AG00
MTQPMPGKPAEDAENELDIRGLFRTLWAGKLWIIGMGLAFALIALAYTFFARQEWSSTAI
TDRPTVNMLGGYYSQQQFLRNLDVRSNMASADQPSVMDEAYKEFVMQLASWDTRREFWLQ
TDYYKQRMVGNSKADAALLDEMINNIQFIPGDFTRAVNDSVKLIAETAPDANNLLRQYVA
FASQRAASHLNDELKGAWAARTIQMKAQVKRQEEVAKAIYDRRMNSIEQALKIAEQHNIS
RSATDVPAEELPDSEMFLLGRPMLQARLENLQAVGPAFDLDYDQNRAMLNTLNVGPTLDP
RFQTYRYLRTPEEPVKRDSPRRAFLMIMWGIVGGLIGAGVALTRRCSK

>P18777

MGSGWHEWPLMIFTVFGQCVAGGFIVLALALLKGDLRAEAQQRVIACMFGLWVLMGIGFI
ASMLHLGSPMRAFNSLNRVGASALSNEIASGSIFFAVGGIGWLLAMLKKLSPALRTLWLI
VTMVLGVIFVWMMVRVYNSIDTVPTWYSIWTPMGFFLTMFMGGPLLGYLLLSLAGVDGWA
MRLLPAISVLALVVSGVVSVMQGAELATIHSSVQQAAALVPDYGALMSWRIVLLAVALCL
WIAPQLKGYQPAVPLLSVSFILLLAGELIGRGVFYGLHMTVGMAVAS

>P75885

MKHKLSAILMAFMLTTPAAFAAPEATNGTEATTGTTGTTTTTTGATTTATTTGGVAAGAV
GTATVVGVATAVGVATLAVVAANDSGDGGSHNTSTTTSTTR

>P76657

MILFADYNTPYLFAISFVLLIGLLEIFALICGHMLSGALDAHLDHYDSITTGHISQALHY
LNIGRLPALVVLCLLAGFFGLIGILLQHACIMVWQSPLSNLFVVPVSLLFTIIAVHYTGK
IVAPWIPRDHSSAITEEEYIGSMALITGHQATSGNPCEGKLTDQFGQIHYLLLEPEEGKI
FTKGDKVLIICRLSATRYLAENNPWPQIL

>P77921

MASHHEITDHKHGEMDIRHQQATFAGFIKGATWVSILSIAVLVFLALANS

>P77400

MMNTEGNNGNKPLGLWNVVSIGIGAMVGAGIFALLGQAALLMEASTWVAFAFGGIVAMFS
GYAYARLGASYPSNGGIIDFFRRGLGNGVFSLALSLLYLLTLAVSIAMVARAFGAYAVQF
LHEGSQEEHLILLYALGIIAVMTLFNSLSNHAVGRLEVILVGIKMMILLLLIIAGVWSLQ
PAHISVSAPPSSGAFFSCIGITFLAYAGFGMMANAADKVKDPQVIMPRAFLVAIGVTTLL
YISLALVLLSDVSALELEKYADTAVAQAASPLLGHVGYVIVVIGALLATASAINANLFAV
FNIMDNMGSERELPKLMNKSLWRQSTWGNIIVVVLIMLMTAALNLGSLASVASATFLICY
LAVFVVAIRLRHDIHASLPILIVGTLVMLLVIVGFIYSLWSQGSRALIWIIGSLLLSLIV
AMVMKRNKTV

>P08400

MLERLSWKRLVLELLLCCLPAFILGAFFGYLPWFLLASVTGLLIWHFWNLLRLSWWLWVD
RSMTPPPGRGSWEPLLYGLHQMQLRNKKRRRELGNLIKRFRSGAESLPDAVVLTTEEGGI
FWCNGLAQQILGLRWPEDNGQNILNLLRYPEFTQYLKTRDFSRPLNLVLNTGRHLEIRVM
PYTHKQLLMVARDVTQMHQLEGARRNFFANVSHELRTPLTVLQGYLEMMNEQPLEGAVRE
KALHTMREQTQRMEGLVKQLLTLSKIEAAPTHLLNEKVDVPMMLRVVEREAQTLSQKKQT
FTFEIDNGLKVSGNEDQLRSAISNLVYNAVNHTPEGTHITVRWQRVPHGAEFSVEDNGPG
IAPEHIPRLTERFYRVDKARSRQTGGSGLGLAIVKHAVNHHESRLNIESTVGKGTRFSFV
IPERLIAKNSD

>P80588

SAPAQWKLWLVMDPRTVMIGTAAWLGVLALLIHFLLLGTERFNWIDTGLKEQKATAAAQA
AITPAPVTAAAK

>P0AGM0

METPQPDKTGMHILLKLASLVVILAGIHAAADIIVQLLLALFFAIVLNPLVTWFIRRGVQ
RPVAITIVVVVMLIALTALVGVLAASFNEFISMLPKFNKELTRKLFKLQEMLPFLNLHMS
PERMLQRMDSEKVVTFTTALMTGLSGAMASVLLLVMTVVFMLFEVRHVPYKMRFALNNPQ
IHIAGLHRALKGVSHYLALKTLLSLWTGVIVWLGLELMGVQFALMWAVLAFLLNYVPNIG
AVISAVPPMIQVLLFNGVYECILVGALFLVVHMVIGNILEPRMMGHRLGMSTMVVFLSLL
IWGWLLGPVGMLLSVPLTSVCKIWMETTKGGSKLAILLGPGRPKSRLPG

>P39284
MSARMFVLCCIWFIVAFLWITITSALDKEWMIDGRGINNVCDVLMYLEEDDTRDVGVIMT
LPLFFPFLWFALWRKKRGWFMYATALAIFGYWLWQFFLRYQFCL
>P33011
MRADKSLSPFEIRVYRHYRIVHGTRVALAFLLTFLIIRLFTIPESTWPLVTMVVIMGPIS
FWGNVVPRAFERIGGTVLGSILGLIALQLELISLPLMLVWCAAAMFLCGWLALGKKPYQG
LLIGVTLAIVVGSPTGEIDTALWRSGDVILGSLLAMLFTGIWPQRAFIHWRIQLAKSLTE
YNRVYQSAFSPNLLERPRLESHLQKLLTDAVKMRGLIAPASKETRIPKSIYEGIQTINRN
LVCMLELQINAYWATRPSHFVLLNAQKLRDTQHMMQQILLSLVHALYEGNPQPVFANTEK
LNDAVEELRQLLNNHHDLKVVETPIYGYVWLNMETAHQLELLSNLICRALRK
>P75835
MAGNVQEKQLRWYNIALMSFITVWGFGNVVNNYANQGLVVVFSWVFIFALYFTPYALIVG
QLGSTFKDGKGGVSTWIKHTMGPGLAYLAAWTYWVVHIPYLAQKPQAILIALGWAMKGDG
SLIKEYSVVALQGLTLVLFIFFMWVASRGMKSLKIVGSVAGIAMFVMSLLYVAMAVTAPA
ITEVHIATTNITWETFIPHIDFTYITTISMLVFAVGGAEKISPYVNQTRNPGKEFPKGML
CLAVMVAVCAILGSLAMGMMFDSRNIPDDLMTNGQYYAFQKLGEYYNMGNTLMVIYAIAN
TLGQVAALVFSIDAPLKVLLGDADSKYIPASLCRTNASGTPVNGYFLTLVLVAILIMLPT
LGIGDMNNLYKWLLNLNSVVMPLRYLWVFVAFIAVVRLAQKYKPEYVFIRNKPLAMTVGI
WCFAFTAFACLTGIFPKMEAFTAEWTFQLALNVATPFVLVGLGLIFPLLARKANSK
>P0AEB2
MNTIFSARIMKRLALTTALCTAFISAAHADDLNIKTMIPGVPQIDAESYILIDYNSGKVL
AEQNADVRRDPASLTKMMTSYVIGQAMKAGKFKETDLVTIGNDAWATGNPVFKGSSLMFL
KPGMQVPVSQLIRGINLQSGNDACVAMADFAAGSQDAFVGLMNSYVNALGLKNTHFQTVH
GLDADGQYSSARDMALIGQALIRDVPNEYSIYKEKEFTFNGIRQLNRNGLLWDNSLNVDG
IKTGHTDKAGYNLVASATEGQMRLISAVMGGRTFKGREAESKKLLTWGFRFFETVNPLKV
GKEFASEPVWFGDSDRASLGVDKDVYLTIPRGRMKDLKASYVLNSSELHAPLQKNQVVGT
INFQLDGKTIEQRPLVVLQEIPEGNFFGKIIDYIKLMFHHWFG
>P0AAZ0
MEDETLGFFKKTSSSHARLNVPALVQVAALAIIMIRGLDVLMIFNTLGVRGIGEFIHRSV
QTWSLTLVFLSSLVLVFIEIWCAFSLVKGRRWARWLYLLTQITAASYLWAASLGYGYPEL
FSIPGESKREIFHSLMLQKLPDMLILMLLFVPSTSRRFFQLQ
>P0ACX0
MGLVIKAALGALVVLLIGVLAKTKNYYIAGLIPLFPTFALIAHYIVASERGIEALRATII
FSMWSIIPYFVYLVSLWYFTGMMRLPAAFVGSVACWGISAWVLIICWIKLH
>P64423
MEAIKGSDVNVPDAVFAWMLDGRGGVKPLENTDVIDEAHPCWLHLNYVHHDSAQWLATTP
LLPNNVRDALAGESTRPRVSRLGEGTLITLRCINGSTDERPDQLVAMRVYMDGRLIVSTR
QRKVLALDDVVSDLEEGTGPTDCGGWLVDVCDALTDHSSEFIEQLHDKIIDLEDNLLDQQ
IPPRGFLALLRKQLIVMRRYMAPQRDVYARLASERLPWMSDDQRRRMQDIADRLGRGLDE
IDACIARTGVMADEIAQVMQENLARRTYTMSLMAMVFLPSTFLTGLFGVNLGGIPGGGWQ
FGFSIFCILLVVLIGGVALWLHRSKWL
>P37746
MNTNKLSLRRNVIYLAVVQGSNYLLPLLTFPYLVRTLGPENFGIFGFCQATMLYMIMFVE
YGFNLTATQSIAKAADSKDKVTSIFWAVIFSKIVLIVITLIFLTSMTLLVPEYNKHAVII

WSFVPALVGNLIYPIWLFQGKEKMKWLTLSSILSRLAIIPLTFIFVNTKSDIAIAGFIQS
SANLVAGIIALAIVVHEGWIGKVTLSLHNVRRSLADGFHVFISTSAISLYSTGIVIILGF
ISGPTSVGNFNAANTIRNALQGLLNPITQAIYPRISSTLVLNRVKGVILIKKSLTCLSLI
GGAFSLILLLGASILVKISIGPGYDNAVIVLMIISPLPFLISLSNVYGIQVMLTHNYKKE
FSKILIAAGLLSLLLIFPLTTLFKEIGAAITLLATECLVTSLMLMFVRNNKLLVC
>P37002
MLQLLLAVFIGGGTGSVARWLLSMRFNPLHQAIPLGTLTANLIGAFIIGIGFAWFSRMTN
IDPVWKVLITTGFCGGLTTFSTFSAEVVFLLQEGRFGWALLNVFVNLLGSFAMTALAFWL
FSASTAH
>P37617
MSTPDNHGKKAPQFAAFKPLTTVQNANDCCCDGACSSTPTLSENVSGTRYSWKVSGMDCA
ACARKVENAVRQLAGVNQVQVLFATEKLVVDADNDIRAQVESALQKAGYSLRDEQAAEEP
QASRLKENLPLITLIVMMAISWGLEQFNHPFGQLAFIATTLVGLYPIARQALRLIKSGSY
FAIETLMSVAAIGALFIGATAEAAMVLLLFLIGERLEGWAASRARQGVSALMALKPETAT
RLRKGEREEVAINSLRPGDVIEVAAGGRLPADGKLLSPFASFDESALTGESIPVERATGD
KVPAGATSVDRLVTLEVLSEPGASAIDRILKLIEEAEERRAPIERFIDRFSRIYTPAIMA
VALLVTLVPPLLFAASWQEWIYKGLTLLLIGCPCALVISTPAAITSGLAAAARRGALIKG
GAALEQLGRVTQVAFDKTGTLTVGKPRVTAIHPATGISESELLTLAAAVEQGATHPLAQA
IVREAQVAELAIPTAESQRALVGSGIEAQVNGERVLICAAGKHPADAFTGLINELESAGQ
TVVLVVRNDDVLGVIALQDTLRADAATAISELNALGVKGVILTGDNPRAAAAIAGELGLE
FKAGLLPEDKVKAVTELNQHAPLAMVGDGINDAPAMKAAAIGIAMGSGTDVALETADAAL
THNHLRGLVQMIELARATHANIRQNITIALGLKGIFLVTTLLGMTGLWLAVLADTGATVL
VTANALRLLRRR
>P37739
MRDTTGGPAGAEVWTVPGLLGARKLDLLALIPLVAIVALMTLVGALLFAVAQSDANRARA
KLATDALWVEQTLRFQMAVDEDVLVRLALDASAGASQQALSARARLHLAANPETLGLRWY
DATGRLIAAVPEGPGPAEAALVRQLLASGALPPRPVYGPVRDGRVVLAERVSASGGVVVA
TVSLPMMLERHLPWWIAEQYGVRISDTSGVLAERARRPIAAAAPRHGISFDPPLAGTTLE
IMAYDAPDAFGNAALLAAIGALSVFAVLAMVVLHRNALRRRMAEDRLRAEMAFRRAMEES
LTVGMRAKDLSGRILYVNGAFCKLVGLAAEDLVGRAQPMPYWAPDFLEETLARQRQLIEG
QPVPQAFETRFRRSDGSEIEVQVFEAPLIDAGGRHRGWMGSVIDITQAKQAARLARAQDE
SLARTGRLVTLGEMASTLAHELNQPLAAIASYAAGGLNLFDQPEPNLTMLRQAFEKMGAQ
ARRAGLVIRRVQDFVKKRTPQLAALDLSEVLAEALSITAPVAREHRVKLASLIEGRIPGV
QADRILIEQVLVNLIRNGVEAMAEGPRTGDDLTVRLARAGAAVTIEVMDRGPGISDAVAA
SLFDPFTSTKSEGMGMGLNICRSIVEMHHGSLSHGPRAGGGTVFTVTLPVPQEGAPA
>P0A6E6
MAMTYHLDVVSAEQQMFSGLVEKIQVTGSEGELGIYPGHAPLLTAIKPGMIRIVKQHGHE
EFIYLSGGILEVQPGNVTVLADTAIRGQDLDEARAMEAKRKAEEHISSSHGDVDYAQASA
ELAKAIAQLRVIELTKKAM
>P0AE16
MSSQYLRIFQQPRSAILLILGFASGLPLALTSGTLQAWMTVENIDLKTIGFFSLVGQAYV
FKFLWSPLMDRYTPPFFGRRRGWLLATQILLLVAIAAMGFLEPGTQLRWMAALAVVIAFC
SASQDIVFDAWKTDVLPAEERGAGAAISVLGYRLGMLVSGGLALWLADKWLGWQGMYWLM
AALLIPCIIATLLAPEPTDTIPVPKTLEQAVVAPLRDFFGRNNAWLILLLIVLYKLGDAF

AMSLTTTFLIRGVGFDAGEVGVVNKTLGLLATIVGALYGGILMQRLSLFRALLIFGILQG
ASNAGYWLLSITDKHLYSMGAAVFFENLCGGMGTSAFVALLMTLCNKSFSATQFALLSAL
SAVGRVYVGPVAGWFVEAHGWSTFYLFSVAAAVPGLILLLVCRQTLEYTRVNDNFISRTA
YPAGYAFAMWTLAAGVSLLAVWLLLLTMDALDLTHFSFLPALLEVGVLVALSGVVLGGLL
DYLALRKTHLT
>P0ABJ1
MRLRKYNKSLGWLSLFAGTVLLSGCNSALLDPKGQIGLEQRSLILTAFGLMLIVVIPAIL
MAVGFAWKYRASNKDAKYSPNWSHSNKVEAVVWTVPILIIIFLAVLTWKTTHALEPSKPL
AHDEKPITIEVVSMDWKWFFIYPEQGIATVNEIAFPANTPVYFKVTSNSVMNSFFIPRLG
SQIYAMAGMQTRLHLIANEPGTYDGISASYSGPGFSGMKFKAIATPDRAAFDQWVAKAKQ
SPNTMSDMAAFEKLAAPSEYNQVEYFSNVKPDLFADVINKFMAHGKSMDMTQPEGEHSAH
EGMEGMDMSHAESAH
>P77589
MSTRTPSSSSSRLMLTIGLCFLVALMEGLDLQAAGIAAGGIAQAFALDKMQMGWIFSAGI
LGLLPGALVGGMLADRYGRKRILIGSVALFGLFSLATAIAWDFPSLVFARLMTGVGLGAA
LPNLIALTSEAAGPRFRGTAVSLMYCGVPIGAALAATLGFAGANLAWQTVFWVGGVVPLI
LVPLLMRWLPESAVFAGEKQSAPPLRALFAPETATATLLLWLCYFFTLLVVYMLINWLPL
LLVEQGFQPSQAAGVMFALQMGAASGTLMLGALMDKLRPVTMSLLIYSGMLASLLALGTV
SSFNGMLLAGFVAGLFATGGQSVLYALAPLFYSSQIRATGVGTAVAVGRLGAMSGPLLAG
KMLALGTGTVGVMAASAPGILVAGLAVFILMSRRSRIQPCADA
>P52636
MAENKRTRWQRRPGTTGGKLPWNDWRNATTWRKATQLLLLAMNIYIAITFWYWVRYYETA
SSTTFVARPGGIEGWLPIAGLMNLKYSLVTGQLPSVHAAAMLLLVAFIVISLLLKKAFCS
WLCPVGTLSELIGDLGNKLFGRQCVLPRWLDIPLRGVKYLLLSFFIYIALLMPAQAIHYF
MLSPYSVVMDVKMLDFFRHMGTATLISVTVLLIASLFIRHAWCRYLCPYGALMGVVSLLS
PFKIRRNAESCIDCGKCAKNCPSRIPVDKLIQVRTVECTGCMTCVESCPVASTLTFSLQK
PAANKKAFALSGWLMTLLVLGIMFAVIGYAMYAGVWQSPVPEELYRRLIPQAPMIGH
>P45130
MRFTKSIKFSLKCGRFFIMFTTMLVKKFSPKFTALFRTLLYAIFALIIILCLLVDRGISFY
VRDKIFTNIDELPFRPCALVLGTSKYTVSGKPNVYYDSRLMAAKSLIEQQKVNYLLLSGD
NRTLQYNEPRAMFRDLRKMGVPKTLMFRDFAGFRTLDSVIRADKIFQVKTFTIVSQKFHC
ERALLIAQAHNIDAICFVAKQPELHFSTQIREVFARIKAVFDLILGVEPYFLGEPQPLPN
STTL
>Q47377
MIWLTLVFASLLSVAGQLCQKQATCFVAINKRRKHIVLWLGLALACLGLAMVLWLLVLQN
VPVGIAYPMLSLNFVWVTLAAVKLWHEPVSPRHWCGVAFIIGGIVILGSTV
>P75712
MEHQRKLFQQRGYSEDLLPKTQSQRTWKTFNYFTLWMGSVHNVPNYVMVGGFFILGLSTF
SIMLAIILSAFFIAAVMVLNGAAGSKYGVPFAMILRASYGVRGALFPGLLRGGIAAIMWF
GLQCYAGSLACLILIGKIWPGFLTLGGDFTLLGLSLPGLITFLIFWLVNVGIGFGGGKVL
NKFTAILNPCIYIVFGGMAIWAISLVGIGPIFDYIPSGIQKAENGGFLFLVVINAVVAVW
AAPAVSASDFTQNAHSFREQALGQTLGLVVAYILFAVAGVCIIAGASIHYGADTWNVLDI
VQRWDSLFASFFAVLVILMTTISTNATGNIIPAGYQIAAIAPTKLTYKNGVLIASIISLL
ICPWKLMENQDSIYLFLDIIGGMLGPVIGVMMAHYFVVMRGQINLDELYTAPGDYKYYDN

GFNLTAFSVTLVAVILSLGGKFIHFMEPLSRVSWFVGVIVAFAAYALLKKRTTAEKTGEQ
KTIG
>P30878
MSISLTTKLSYGFGAFGKDFAIGIVYMYLMYYYTDVVGLSVGLVGTLFLVARIWDAINDP
IMGWIVNATRSRWGKFKPWILIGTLTNSLVLFLLFSAHLFEGTAQVVFVCVTYILWGMTY
TIMDIPFWSLVPTITLDKREREQLVPFPRFFASLAGFVTAGITLPFVSYVGGADRGFGFQ
MFTLVLIAFFIASTIVTLRNVHEVYSSDNGVTAGRPHLTLKTIVGLIYKNDQLSCLLGMA
LAYNIASNIINGFAIYYFTYVIGDADLFPYYLSYAGAANLLTLIVFPRLVKMLSRRILWA
GASVMPVLSCAGLFAMALADIHNAALIVAAGIFLNIGTALFWVLQVIMVADTVDYGEFKL
NIRCESIAYSVQTMVVKGGSAFAAFFIALVLGLIGYTPNVAQSAQTLQGMQFIMIVLPVL
FFMMTLVLYFRYYRLNGDMLRKIQIHLLDKYRKTPPFVEQPDSPAISVVATSDVKA
>P54702
MIQVTSEQWLYWLHLYFWPLLRVLALISTAPILSERAIPKRVKLGLGIMITLVIAPSLPA
NDTPLFSIAALWLAMQQILIGIALGFTMQFAFAAVRTAGEFIGLQMGLSFATFVDPGSHL
NMPVLARIMDMLAMLLFLTFNGHLWLISLLVDTFHTLPIGSNPVNSNAFMALARAGGLIF
LNGLMLALPVITLLLTLNLALGLLNRMAPQLSIFVIGFPLTLTVGIMLMAALMPLIAPFC
EHLFSEIFNLLADIVSEMPINNNP
>Q56973
MQNLLKNLAASLGRKPFVADKQGVYRLTIDKHLVMLAPHGSELVLRTPIDAPMLREGNNV
NVTLLRSLMQQALAWAKRYPQTLVLDDCGQLVLEARLRLQELDTHGLQEVINKQLALLEH
LIPQLTPFSVASRVGWN
>P23910
MKKVILSLALGTFGLGMAEFGIMGVLTELAHNVGISIPAAGHMISYYALGVVVGAPIIAL
FSSRYSLKHILLFLVALCVIGNAMFTLSSSYLMLAIGRLVSGFPHGAFFGVGAIVLSKII
KPGKVTAAVAGMVSGMTVANLLGIPLGTYLSQEFSWRYTFLLIAVFNIAVMASVYFWVPD
IRDEAKGNLREQFHFLRSPAPWLIFAATMFGNAGVFAWFSYVKPYMMFISGFSETAMTFI
MMLVGLGMVLGNMLSGRISGRYSPLRIAAVTDFIIVLALLMLFFCGGMKTTSLIFAFICC
AGLFALSAPLQILLLQNAKGGELLGAAGGQIAFNLGSAVGAYCGGMMLTLGLAYNYVALP
AALLSFAAMSSLLLYGRYKRQQAADTPVLAKPLG
>P09852
MTATDRTPPPLKWLCLGNRDANDGFELFAHGIYARNGALVGSKLSLRERRQRVDLSAFLS
GAPPLLAEAAVKHLLARLLCVHRHNTDLELLGKNFIPLHASSLGNAGVCERILASARQLQ
QHQVELCLLLAIDEQEPASAEYLTSLARLRDSGVRIALHPQRIDTDARQCFAEVDAGLCD
YLGLDARLLAPGPLTRNLRQRKSIEYLNRLLVAQDIQMLCLNVDNEELHQQANALPFAFR
HGRHYSEPFQAWPFSSPAC
>P69428
MGGISIWQLLIIAVIVVLLFGTKKLGSIGSDLGASIKGFKKAMSDDEPKQDKTSQDADFT
AKTIADKQADTNQEQAKTEDAKRHDKEQV
>P0A1W8
MTHQLKSRDIIALGFMTFALFVGAGNIIFPPMVGLQAGEHVWTAAIGFLITAVGLPVLTV
VALAKVGGGVDSLSTPIGKVAGLLLATVCYLAVGPLFATPRTATVSFEVGIAPLTGDSAM
PLLIYSVVYFAIVILVSLYPGKLLDTVGNFLAPLKIIALVILSVAAIVWPAGPISNALDA
YQNAAFSNGFVNGYLTMDTLGAMVFGIVIVNAARSRGVTEARLLTRYTVWAGLMAGVGLT
LLYLALFRLGSDSATLVDQSANGAAILHAYVQHTFGGAGSFLLAALIFIACLVTAVGLTC

ACAEFFAQYIPLSYRTLVFILGGFSMVVSNLGLSHLIQISIPVLTAIYPPCIALVVLSFT
RSWWHNSTRIIAPAMFISLLFGILDGIKASAFGDMLPAWSQRLPLAEQGLAWLMPTVVMV
ILAIIWDRAAGRQVTSSAH
>P04825
MTQQPQAKYRHDYRAPDYQITDIDLTFDLDAQKTVVTAVSQAVRHGASDAPLRLNGEDLK
LVSVHINDEPWTAWKEEEGALVISNLPERFTLKIINEISPAANTALEGLYQSGDALCTQC
EAEGFRHITYYLDRPDVLARFTTKIIADKIKYPFLLSNGNRVAQGELENGRHWVQWQDPF
PKPCYLFALVAGDFDVLRDTFTTRSGREVALELYVDRGNLDRAPWAMTSLKNSMKWDEER
FGLEYDLDIYMIVAVDFFNMGAMENKGLNIFNSKYVLARTDTATDKDYLDIERVIGHEYF
HNWTGNRVTCRDWFQLSLKEGLTVFRDQEFSSDLGSRAVNRINNVRTMRGLQFAEDASPM
AHPIRPDMVIEMNNFYTLTVYEKGAEVIRMIHTLLGEENFQKGMQLYFERHDGSAATCDD
FVQAMEDASNVDLSHFRRWYSQSGTPIVTVKDDYNPETEQYTLTISQRTPATPDQAEKQP
LHIPFAIELYDNEGKVIPLQKGGHPVNSVLNVTQAEQTFVFDNVYFQPVPALLCEFSAPV
KLEYKWSDQQLTFLMRHARNDFSRWDAAQSLLATYIKLNVARHQQGQPLSLPVHVADAFR
AVLLDEKIDPALAAEILTLPSVNEMAELFDIIDPIAIAEVREALTRTLATELADELLAIY
NANYQSEYRVEHEDIAKRTLRNACLRFLAFGETHLADVLVSKQFHEANNMTDALAALSAA
VAAQLPCRDALMQEYDDKWHQNGLVMDKWFILQATSPAANVLETVRGLLQHRSFTMSNPN
RIRSLIGAFAGSNPAAFHAEDGSGYLFLVEMLTDLNSRNPQVASRLIEPLIRLKRYDAKR
QEKMRAALEQLKGLENLSGDLYEKITKALA
>P76221
MMMMQSRKIWYYRITLIIILLFAMLLAWALLPGVHEFINRSVAAFAAVDQQGIERFIQSYG
ALAAVVSFLLMILQAIAAPLPAFLITFANASLFGAFWGGLLSWTSSMAGAALCFFIARVM
GREVVEKLTGKTVLDSMDGFFTRYGKHTILVCRLLPFVPFDPISYAAGLTSIRFRSFFIA
TGLGQLPATIVYSWAGSMLTGGTFWFVTGLFILFALTVVIFMAKKIWLERQKRNA
>P0ABC0
MSVSLVSRNVARKLLLVQLLVVIASGLLFSLKDPFWGVSAISGGLAVFLPNVLFMIFAWR
HQAHTPAKGRVAWTFAFGEAFKVLAMLVLLVVALAVLKAVFLPLIVTWVLVLVVQILAPA
VINNKG
>P0AFL1
MNNLPVVRSPWRIVILLLGFTFLYAPMLMLVIYSFNSSKLVTVWAGWSTRWYGELLRDDA
MMSAVGLSLTIAACAATAAAILGTIAAVVLVRFGRFRGSNGFAFMITAPLVMPDVITGLS
LLLLFVALAHAIGWPADRGMLTIWLAHVTFCTAYVAVVISSRLRELDRSIEEAAMDLGAT
PLKVFFVITLPMIMPAIISGWLLAFTLSLDDLVIASFVSGPGATTLPMLVFSSVRMGVNP
EINALATLILGAVGIVGFIAWYLMARAEKQRIRDIQRARRG
>P18814
MAMVQPKSQKLRLFTTHLLLLIFIAAIMFPLLMVIAISLREGNFATGSLIPDTISWEHWR
LALGFSVEHADGRVTPPPFPVLLWLWNSIKVAGITAIGIVALSTTCAYAFARMRFPGKAT
LLKGMLIFQMFPAVLSLVALYALFDRLGQYLPFVGLNTHGGVIFAYMGGIALHVWTIKGY
FETIDGSLEEAAALDGATPWQAFRLVLVPLSVPILAVVFILSFIAAITEVPVASLLLRDV
NSYTLAVGMQQYLNPQNYLWGDFAAAAVLSAIPITVVFLLAQRWLVNGLTAGGVKG
>P0AET5
MLYIDKATILKFDLEMLKKHRRAIQFIAVLLFIVGLLCISFPFVSGDILSTVVGALLICS
GIALIVGLFSNRSHNFWPVLSGFLVAVAYLLIGYFFIRAPELGIFAIAAFIAGLFCVAGV
IRLMSWYRQRSMKGSWLQLVIGVLDIVIAWIFLGATPMVSVTLVSTLVGIELIFSAASLF

SFASLFVKQQ
>P67601
MTITDLVLILFIAALLAFAIYDQFIMPRRNGPTLLAIPLLRRGRIDSVIFVGLIVILIYN
NVTNHGALITTWLLSALALMGFYIFWIRVPKIIFKQKGFFFANVWIEYSRIKAMNLSEDG
VLVMQLEQRRLLIRVRNIDDLEKIYKLLVSTQ
>Q06916
MGAGLVASLLAGTATGLGALPVLVTSELSRKAQGPDVGLQRGRDAGGQSFSLVIPAMELV
RGQGHDGPSAALRVAAGVLLGGLFLRVWHDLMPHEHALKGHEGHGGTKWNSALLFVLAMT
LHNFPEGLAVGVSFAAPQPELGLSVALGIGAQNIPEGLVVALALRASGASASRAAFLALL
TGMVEPVGALFGVLALSLSSALLPWGLAFAGGAMLYVISHEMIPESHRGGFEREATTGLM
WGFVLALVLDMSLG
>P0AFS1
MRIRYGWELALAALLVIEIVAFGAINPRMLDLNMLLFSTSDFICIGIVALPLTMVIVSGG
IDISFGSTIGLCAIALGVLFQSGVPMPLAILLTLLLGALCGLINAGLIIYTKVNPLVITL
GTLYLFAGSALLLSGMAGATGYEGIGGFPMAFTDFANLDVLGLPVPLIIFLICLLVFWLW
LHKTHAGRNVFLIGQSPRVALYSAIPVNRTLCALYAMTGLASAVAAVLLVSYFGSARSDL
GASFLMPAITAVVLGGANIYGGSGSIIGTAIAVLLVGYLQQGLQMAGVPNQVSSALSGAL
LIVVVVGRSVSLHRQQIKEWLARRANNPLP
>P75747
MPVLQWGMLCVLSLLLSIGFLAVHLPAALLLGPMIAGIIFSMRGITLQLPRSAFLAAQAI
LGCMIAQNLTGSILTTLAVNWPIVLAILLVTLLSSAIVGWLLVRYSSLPGNTGAWGSSPG
GAAAMVAMAQDYGADIRLVAFMQYLRVLFVAGAAVLVTRMMLGDNAEAVNQHIVWFPPVS
INLLLTILLAVVAGTVGCLLRLPSGTMLIPMLAGAVLQSGQLITIELPEWLLAMAYMAIG
WRIGLGFDKQILLRALRPLPQILLSIFALLAICAGMAWGLTRFMHIDFMTAYLATSPGGL
DTVAVIAAGSNADMALIMAMQTLRLFSILLTGPAIARFISTYAPKRSA
>P0ABH4
MASYRSQGRWVIWLSFLIALLLQIMPWPDNLIVFRPNWVLLILLYWILALPHRVNVGTGF
VMGAILDLISGSTLGVRVLAMSIIAYLVALKYQLFRNLALWQQALVVMLLSLVVDIIVFW
AEFLVINVSFRPEVFWSSVVNGVLWPWIFLLMRKVRQQFAVQ
>P0A8Q3
MINPNPKRSDEPVFWGLFGAGGMWSAIIAPVMILLVGILLPLGLFPGDALSYERVLAFAQ
SFIGRVFLFLMIVLPLWCGLHRMHHAMHDLKIHVPAGKWVFYGLAAILTVVTLIGVVTI
>P0AEY8
MQNKLASGARLGRQALLFPLCLVLYEFSTYIGNDMIQPGMLAVVEQYQAGIDWVPTSMTA
YLAGGMFLQWLLGPLSDRIGRRPVMLAGVVWFIVTCLAILLAQNIEQFTLLRFLQGISLC
FIGAVGYAAIQESFEEAVCIKITALMANVALIAPLLGPLVGAAWIHVLPWEGMFVLFAAL
AAISFFGLQRAMPETATRIGEKLSLKELGRDYKLVLKNGRFVAGALALGFVSLPLLAWIA
QSPIIIITGEQLSSYEYGLLQVPIFGALIAGNLLLARLTSRRTVRSLIIMGGWPIMIGLL
VAAAATVISSHAYLWMTAGLSIYAFGIGLANAGLVRLTLFASDMSKGTVSAAMGMLQMLI
FTVGIEISKHAWLNGGNGLFNLFNLVNGILWLSLMVIFLKDKQMGNSHEG
>P75916
MNILLSIAITTGILSGIWGWVAVSLGLLSWAGFLGCTAYFACPQGGLKGLAISAATLLSG
VVWAMVIIYGSALAPHLEILGYVITGIVAFLMCIQAKQLLLSFVPGTFIGACATFAGQGD
WKLVLPSLALGLIFGYAMKNSGLWLAARSAKTAHREQEIKNKA

>P36561
MSKLFWAMLSFITRLPVPRRWSQGLDFEHYSRGIITFPLIGLLLGAISGLVFMVLQAWCG
APLAALFSVLVLVLMTGGFHLDGLADTCDGVFSARSRDRMLEIMRDSRLGTHGGLALIFV
VLAKILVLSELALRGESILASLAAACAVSRGTAALLMYRHRYAREEGLGNVFIGKIDGRQ
TCVTLGLAAIFAAVLLPGMHGVAAMVVTMVAIFILGQLLKRTLGGQTGDTLGAAIELGEL
VFLLALL
>P76278
MLAFTWIALRFIHFTSLMLVFGFAMYGAWLAPLTIRRLLAKRFLRLQQHAAVWSLISATA
MLAVQGGLMGTGWTDVFSPNIWQAVLQTQFGGIWLWQIVLALVTLIVALMQPRNMPRLLF
MLTTAQFILLAGVGHATLNEGVTAKIHQTNHAIHLICAAAWFGGLLPVLWCMQLIKGRWR
HQAIQALMRFSWCGHFAVIGVLASGVLNALLITGFPPTLTTYWGQLLLLKAILVMIMVVI
ALANRYVLVPRMRQDEDRAAPWFVWMTKLEWAIGAVVLVIISLLATLEPF
>P64481
MSFIMTAEGHLLFSIACAVFAKNAELTPVLAQGDWWHIVPSAILTCLLPDIDHPKSFLGQ
RLKWISKPIARAFGHRGFTHSLLAVFALLATFYLKVPEGWFIPADALQGMVLGYLSHILA
DMLTPAGVPLLWPCRWRFRLPILVPQKGNQLERFICMALFVWSVWMPHSLPENSAVRWSS
QMINTLQIQFHRLIKHQVEY
>P46927
MTTIGTPLRTNATKVMMLGSGELGKEVVIELQPLGVEVIAVDRYDNAPAQQVAHRAYTIS
MLDGNALRDLVEKEKPDFIVPEVEAIATATLVELEQEGYNVIPTAKATQLTMNREGIRRL
AAEELGLKTSPYRFVDNFEQFQQAIQEIGIPCVVKPIMSSSGHGQSVIKSEADIQQAWDY
SQQGGRAGGGRVIVEGFIKFDYEITQLTVRHIHGIVFSSHRHIQVDGDYRESWQPQQMSD
IALKKAQETAEKITSALGGRGIFGVELFVCGDEIIFNEVSPRPHDTGIVTMASQELSQFA
LHARAILGLPIPEIYRISPAASKAIVVEGKSDNVRFGGVDKVLAEIGTNIRLFGKGEVNG
HRRLGVILARDENTVRALETSRRAYDKLDIQL
>P0AGH8
MAATKPAFNPPGKKGDIIFSVLVKLAALIVLLMLGGIIVSLIISSWPSIQKFGLAFLWTK
EWDAPNDIYGALVPIYGTLVTSFIALLIAVPVSFGIALFLTELAPGWLKRPLGIAIELLA
AIPSIVYGMWGLFIFAPLFAVYFQEPVGNIMSNIPIVGALFSGPAFGIGILAAGVILAIM
IIPYIAAVMRDVFEQTPVMMKESAYGIGCTTWEVIWRIVLPFTKNGVIGGIMLGLGRALG
ETMAVTFIIGNTYQLDSASLYMPGNSITSALANEFAEAESGLHVAALMELGLILFVITFI
VLAASKFMIMRLAKNEGAR
>O87576
MAFASLIERQRIRLLLALLFGACGTLAFSPYDVWPAAIVSLIGLQALTFNRRPLQSAAIG
YCWGLGLFGSGINWVYVSIAQFGGMPGPVNVFLVVLLAAYLSLYTGLFAGILSRLWPKTN
WLRVAIAAPAIWQITEFLRGWVLTGFPWLQFGYSQVDGPLKGLAPVMGVEAINFLLMMVS
GLLALALATRNWRPLAVAVILFALPFPLRYIQWFTLEPAKATQVSLVQGDIPQSLKWDEN
QLLNTLKIYLNETRPELGKSQIIIWPESAIPDLEINQQPFLRSLDEMLREKNSTLITGIV
DARLNKQNRYDTYNTIITLGKDNPYSYDSPNRYNKNHLVPFGEFVPLESILRPLAPFFDL
PMSSFSRGPYIQPQLHAHDYKLTAAICYEIILGEQVRDNFRPDTDYLLTISNDAWFGKSI
GPWQHFQMARMRSLELARPLLRSTNNGITAVIGPQGEIQAMIPQFTRQVLTTNVTPTTGL
TPYARTGNWPLWVLTALFAFGAVVMSLRQRRK
>O87656
MSRKREMPDGGAKSVLSDLRFGRFVGRIRRSRHPALLLLALFVAACWLTWVNFSVALPRS

QWQQAIWSPDIDIIEQMIFHYSQLPRLAISLLVGAGLGLVGVLFQQVLRNPLAEPTTLGV
ATGAQLGITVTTLWAIPGALTTQFAALTGACIVGALVFGVAWGKRLSPVTLILAGLVVSL
YCGAINQLLVIFHHDQLQSMFLWSTGTLTQTDWSGVQRLWPQLLGGVMLTLLLLRPMTLM
GLDDGVARNLGLALSLARLAALSLAIVLSALLVNAVGIIGFIGLFAPLLAKMLGARRLLA
RLMLAPLIGALILWLSDQIILWLTRVWMEVSTGSVTALIGAPLLLWLLPRLKSMSAPDMN
ASDRVAAERRHVLAFAVAGGALLLLATWVALSFGRDAHGWTWASGTLLEELMPWRWPRIL
AALMAGVMLAVAGCIIQRLTGNPMASPEVLGISSGAAFGVVLMLFLVPGNAFGWLLPAGS
LGAAATLLIIMIAAGRGGFSPQRMLLAGMALSTAFTMLLMMLQASGDPRMAEVLTWLSGS
TYNATGGQVTRTAIVMVILLAIVPLCRRWLTILPLGGDAARAVGMALTPSRIALLALAAC
LTATATMTIGPLSFVGLMAPHIARMLGFRRTMPHMVISALAGGVLLVFADWCGRMALFPY
QIPAGLLSSFIGAPYFIYLLRKQSR
>P31801
MSNAQEAVKTRHKETSLIFPVLALVVLFLWGSSQTLPVVIAINLLALIGILSSAFSVVRH
ADVLAHRLGEPYGSLILSLSVVILEVSLISALMATGDAAPTLMRDTLYSIIMIVTGGLVG
FSLLLGGRKFATQYMNLFGIKQYLIALFPLAIIVLVFPMALPAANFSTGQALLVALISAA
MYGVFLLIQTKTHQSLFVYEHEDDSDDDDPHHGKPSAHSSLWHAIWLIIHLIAVIAVTKM
NASSLETLLDSMNAPVAFTGFLVALLILSPEGLGALKAVLNNQVQRAMNLFFGSVLATIS
LTVPVVTLIAFMTGNELQFALGAPEMVVMVASLVLCHISFSTGRTNVLNGAAHLALFAAY
LMTIFA
>P00804
MSQSICSTGLRWLWLVVVVLIIDLGSKYLILQNFALGDTVPLFPSLNLHYARNYGAAFSF
LADSGGWQRWFFAGIAIGISVILAVMMYRSKATQKLNNIAYALIIGGALGNLFDRLWHGF
VVDMIDFYVGDWHFATFNLADTAICVGAALIVLEGFLPSRAKKQ
>Q46839
MVTWTQMYMPMGGLGLSALVALIPIIFFFVALAVLRLKGHVAGAITLILSILIAIFAFKM
PIDMAFAAAGYGFIYGLWPIAWIIVAAVFLYKLTVASGQFDIIRSSVISITDDQRLQVLL
IGFSFGALLEGAAGFGAPVAITGALLVGLGFKPLYAAGLCLIANTAPVAFGALGVPILVA
GQVTGIDPFHIGAMAGRQLPFLSVLVPFWLVAMMDGWKGVKETWPAALVAGGSFAVTQFF
TSNYIGPELPDITSALVSIVSLALFLKVWRPKNTETAISMGQSAGAMVVNKPSSGGPVPS
EYSLGQIIRAWSPFLILTVLVTIWTMKPFKALFAPGGAFYSLVINFQIPHLHQQVLKAAP
IVAQPTPMDAVFKFDPLSAGGTAIFIAAIISIFILGVGIKKGIGVFAETLISLKWPILSI
GMVLAFAFVTNYSGMSTTLALVLAGTGVMFPFFSPFLGWLGVFLTGSDTSSNALFGSLQS
TTAQQINVSDTLLVAANTSGGVTGKMISPQSIAVACAATGMVGRESELFRYTVKHSLIFA
SVIGIITLLQAYVFTGMLVS
>P0A710
MKQFLDFLPLVVFFAFYKIYDIYAATAALIVATAIVLIYSWVRFRKVEKMALITFVLVVV
FGGLTLFFHNDEFIKWKVTVIYALFAGALLVSQWVMKKPLIQRMLGKELTLPQPVWSKLN
LAWAVFFILCGLANIYIAFWLPQNIWVNFKVFGLTALTLIFTLLSGIYIYRHMPQEDKS
>P0AC05
MRRLLSVAPVLLWLITPLAFAQLPGITSQPLPGGGQSWSLPVQTLVFITSLTFIPAILLM
MTSFTRIIIVFGLLRNALGTPSAPPNQVLLGLALFLTFFIMSPVIDKIYVDAYQPFSEEK
ISMQEALEKGAQPLREFMLRQTREADLGLFARLANTGPLQGPEAVPMRILLPAYVTSELK
TAFQIGFTIFIPFLIIDLVIASVLMALGMMMVPPATIALPFKLMLFVLVDGWQLLVGSLA
QSFYS

>P45768

MTKVLLSHPPRPASHNSSRAMVWVRKNLFSSWSNSLLTIGCIWLMWELIPPLLNWAFLQA
NWVGSTRADCTKAGACWVFIHERFGQFMYGLYPHDQRWRINLALLIGLVSIAPMFWKILP
HRGRYIAAWAVIYPLIVWWLMYGGFFALERVETRQWGGLTLTLIIASVGIAGALPWGILL
ALGRRSHMPIVRILSVIFIEFWRGVPLITVLFMSSVMLPLFMAEGTSIDKLIRALVGVIL
FQSAYVAEVVRGGLQALPKGQYEAAESLALGYWKTQGLVILPQALKLVIPGLVNTIIALF
KDTSLVIIIGLFDLFSSVQQATVDPAWLGMSTEGYVFAALIYWIFCFSMSRYSQYLEKRF
NTGRTPH

>P0ADW3

MTWEYALIGLVVGIIIGAVAMRFGNRKLRQQQALQYELEKNKAELDEYREELVSHFARSA
ELLDTMAHDYRQLYQHMAKSSSSLLPELSAEANPFRNRLAESEASNDQAPVQMPRDYSEG
ASGLLRTGAKRD

>P34009

MADELDDQAAMAQWASENPPGGGEGVNEFGDFSGGMGGWDDGGGDGASERILNQDEIDSL
LGFDLSGDGSDDRTGIRAIINSALVSYERLPMLEIVFDRLVRLMTTSLRNFTSDNVEVSL
DNISSIRFGDYLNSIPLPGILAVFRAEELDNYGLLTVDSNLIYSIVDVLLGGRRGTAAMR
IEGRPYTTIERVLVQRMIDVVLHDLKSAFEPLHPVSFSLDRLETNPPFAAIARPANAAIL
VKLRIDMEDRGGRIELLLPYATLEPIRKMLLQQFMGEKFGRDNIWEGHLATELWTTQMEV
RAVLDEQQVPLSRVLNMQVGDTLMLNATSRQPGGAARRRIPLTRGRMGRRNHSIAVRAEA
PVDRRKKAVQKLK

>P37674

MKKILEAILAINLAVLSCIVFINIILRYGFQTSILSVDELSRYLFVWLTFIGAIVAFMDN
AHVQVTFLVEKLSPAWQRRVALVTHSLILFICGALAWGATLKTIQDWSDYSPILGLPIGL
MYAACLPTSLVIAFFELRHLYQLITRSNSLTSPPQGA

>P69380

MNQSYGRLVSRAAIAATAMASLLLLIKIFAWWYTGSVSILAALVDSLVDIGASLTNLLVV
RYSLQPADDNHSFGHGKAESLAALAQSMFISGSALFLFLTGIQHLISPTPMTDPGVGVIV
TIVALICTIILVSFQRWVVRRTQSQAVRADMLHYQSDVMMNGAILLALGLSWYGWHRADA
LFALGIGIYILYSALRMGYEAVQSLLDRALPDEERQEIIDIVTSWPGVSGAHDLRTRQSG
PTRFIQIHLEMEDSLPLVQAHMVADQVEQAILRRFPGSDVIIHQDPCSVVPREGKRSMLS

>P39396

MDTKKIFKHIPWVILGIIGAFCLAVVALRRGEHISALWIVVASVSVYLVAYRYYSLYIAQ
KVMKLDPTRATPAVINNDGLNYVPTNRYVLFGHHFAAIAGAGPLVGPVLAAQMGYLPGTL
WLLAGVVLAGAVQDFMVLFISSRRNGASLGEMIKEEMGPVPGTIALFGCFLIMIIILAVL
ALIVVKALAESPWGVFTVCSTVPIALFMGIYMRFIRPGRVGEVSVIGIVLLVASIYFGGV
IAHDPYWGPALTFKDTTITFALIGYAFVSALLPVWLILAPRDYLATFLKIGVIVGLALGI
VVLNPELKMPAMTQYIDGTGPLWKGALFPFLFITIACGAVSGFHALISSGTTPKLLANET
DARFIGYGAMLMESFVAIMALVAASIIEPGLYFAMNTPPAGLGITMPNLHEMGGENAPII
MAQLKDVTAHAAATVSSWGFVISPEQILQTAKDIGEPSVLNRAGGAPTLAVGIAHVFHKV
LPMADMGFWYHFGILFEALFILTALDAGTRSGRFMLQDLLGNFIPFLKKTDSLVAGIIGT
AGCVGLWGYLLYQGVVDPLGGVKSLWPLFGISNQMLAAVALVLGTVVLIKMKRTQYIWVT
VVPAVWLLICTTWALGLKLFSTNPQMEGFFYMASQYKEKIANGTDLTAQQIANMNHIVVN
NYTNAGLSILFLIVVYSIIFYGFKTWLAVRNSDKRTDKETPYVPIPEGGVKISSHH

>P31119
MLFSFFRNLCRVLYRVRVTGDTQALKGERVLITPNHVSFIDGILLGLFLPVRPVFAVYTS
ISQQWYMRWLKSFIDFVPLDPTQPMAIKHLVRLVEQGRPVVIFPEGRITTTGSLMKIYDG
AGFVAAKSGATVIPVRIEGAELTHFSRLKGLVKRRLFPQITLHILPPTQVAMPDAPRARD
RRKIAGEMLHQIMMEARMAVRPRETLYESLLSAMYRFGAGKKCVEDVNFTPDSYRKLLTK
TLFVGRILEKYSVEGERIGLMLPNAGISAAVIFGAIARRRMPAMMNYTAGVKGLTSAITA
AEIKTIFTSRQFLDKGKLWHLPEQLTQVRWVYLEDLKADVTTADKVWIFAHLLMPRLAQV
KQQPEEEALILFTSGSEGHPKGVVHSHKSILANVEQIKTIADFTTNDRFMSALPLFHSFG
LTVGLFTPLLTGAEVFLYPSPLHYRIVPELVYDRSCTVLFGTSTFLGHYARFANPYDFYR
LRYVVAGAEKLQESTKQLWQDKFGLRILEGYGVTECAPVVSINVPMAAKPGTVGRILPGM
DARLLSVPGIEEGGRLQLKGPNIMNGYLRVEKPGVLEVPTAENVRGEMERGWYDTGDIVR
FDEQGFVQIQGRAKRFAKIAGEMVSLEMVEQLALGVSPDKVHATAIKSDASKGEALVLFT
TDNELTRDKLQQYAREHGVPELAVPRDIRYLKQMPLLGSGKPDFVTLKSWVDEAEQHDE
>P0AAM1
MQQKSDNVVSHYVFEAPVRIWHWLTVLCMAVLMVTGYFIGKPLPSVSGEATYLFYMGYIR
LIHFSAGMVFTVVLLMRIYWAFVGNRYSRELFIVPVWRKSWWQGVWYEIRWYLFLAKRPS
ADIGHNPIAQAAMFGYFLMSVFMIITGFALYSEHSQYAIFAPFRYVVEFFYWTGGNSMDI
HSWHRLGMWLIGAFVIGHVYMALREDIMSDDTVISTMVNGYRSHKFGKISNKERS
>P42910
MHEITLLQGLSLAALVFVLGIDFWLEALFLFRPIIVCTLTGAILGDIQTGLITGGLTELA
FAGLTPAGGVQPPNPIMAGLMTTVIAWSTGVDAKTAIGLGLPFSLLMQYVILFFYSAFSL
FMTKADKCAKEADTAAFSRLNWTTMLIVASAYAVIAFLCTYLAQGAMQALVKAMPAWLTH
GFEVAGGILPAVGFGLLLRVMFKAQYIPYLIAGFLFVCYIQVSNLLPVAVLGAGFAVYEF
FNAKSRQQAQPQPVASKNEEEDYSNGI
>Q53174
MDIAAAIGLIGAIVMVVGSMIYAGGVAPFVDIPSLVIVVAGTAFIVLAMKPLPVFLGHFK
AMMKVFKPSRFDMNEVISTMVELSNLARKDGIMALEGKAVPDAFFEKGLQLLVDGTDEAK
LVKQLKYEIKAMKARHEAYQGAVKAWIDIGPAMGMVGTLIGLVLMLGNMSDPKSIGPAMA
VALLTTLYGALMANVIFAPILNKLEGYSADEVTYRELVIEGLRGIARGESARMIEDQMVC
ALDRKQQMKRKAA
>P43009
MKKFDEINIIPFIDIMLVLLTVVLITASFISQGKIQVNVPKASTAVAFKSDELAKLLTVT
ADKQLYFNDKPISQEALEAEIAQWNKDQKVTLKIDAEASFQDFVTITDMLSKNEIKNVAI
VSMKDKGKSAGKNSQESTPSQSVPTTP
>P0ABG1
MLKYRLISAFVLIPVVIAALFLLPPVGFAIVTLVVCMLAAWEWGQLSGFTTRSQRVWLAV
LCGLLLALMLFLLPEYHRNIHQPLVEISLWASLGWWIVALLLVLFYPGSAAIWRNSKTLR
LIFGVLTIVPFFWGMLALRAWHYDENHYSGAIWLLYVMILVWGADSGAYMFGKLFGKHKL
APKVSPGKTWQGFIGGLATAAVISWGYGMWANLDVAPVTLLICSIVAALASVLGDLTESM
FKREAGIKDSGHLIPGHGGILDRIDSLTAAVPVFACLLLLVFRTL
>P0AD19
MMANIWWSLPLTLIVFFAARKLAARYKFPLLNPLLVAMVVIIPFLMLTGISYDSYFKGSE
VLNDLLQPAVVALAYPLYEQLHQIRARWKSIITICFIGSVVAMVTGTSVALLMGASPEIA
ASILPKSVTTPIAMAVGGSIGGIPAISAVCVIFVGILGAVFGHTLLNAMRIRTKAARGLA

MGTASHALGTARCAELDYQEGAFSSLALVLCGIITSLIAPFLFPIILAVMG
>P31553
MKNEKRKTGIEPKVFFPPLIIVGILCWLTVRDLDAANVVINAVFSYVTNVWGWAFEWYMV
VMLFGWFWLVFGPYAKKRLGNEPPEFSTASWIFMMFASCTSAAVLFWGSIEIYYYISTPP
FGLEPNSTGAKELGLAYSLFHWGPLPWATYSFLSVAFAYFFFVRKMEVIRPSSTLVPLVG
EKHAKGLFGTIVDNFYLVALIFAMGTSLGLATPLVTECMQWLFGIPHTLQLDAIIITCWI
ILNAICVACGLQKGVRIASDVRSYLSFLMLGWVFIVSGASFIMNYFTDSVGMLLMYLPRM
LFYTDPIAKGGFPQGWTVFYWAWWVIYAIQMSIFLARISRGRTVRELCFGMVLGLTASTW
ILWTVLGSNTLLLIDKNIINIPNLIEQYGVARAIIETWAALPLSTATMWGFFILCFIATV
TLVNACSYTLAMSTCREVRDGEEPPLLVRIGWSILVGIIGIVLLALGGLKPIQTAIIAGG
CPLFFVNIMVTLSFIKDAKQNWKD
>P0ABM9
MRFLLGVLMLMISGSALATIDVLQFKDEAQEQQFRQLTEELRCPKCQNNSIADSNSMIAT
DLRQKVYELMQEGKSKKEIVDYMVARYGNFVTYDPPLTPLTVLLWVLPVVAIGIGGWVIY
ARSRRRVRVVPEAFPEQSVPEGKRAGYVVYLPGIVVALIVAGVSYYQTGNYQQVKIWQQA
TAQAPALLDRALDPKADPLNEEEMSRLALGMRTQLQKNPGDIEGWIMLGRVGMALGNASI
ATDAYATAYRLDPKNSDAALGYAEALTRSSDPNDNRLGGELLRQLVRTDHSNIRVLSMYA
FNAFEQQRFGEAVAAWEMMLKLLPANDTRRAVIERSIAQAMQHLSPQESK
>P0AC98
MGNTKLANPAPLGLMGFGMTTILLNLHNVGYFALDGIILAMGIFYGGIAQIFAGLLEYKK
GNTFGLTAFTSYGSFWLTLVAILLMPKLGLTDAPNAQFLGVYLGLWGVFTLFMFFGTLKG
ARVLQFVFFSLTVLFALLAIGNIAGNAAIIHFAGWIGLICGASAIYLAMGEVLNEQFGRT
VLPIGESH
>P37147
MRWLPFIAIFLYVYIEISIFIQVAHVLGVLLTLVLVIFTSVIGMSLVRNQGFKNFVLMQQ
KMAAGENPAAEMIKSVSLIIAGLLLLLPGFFTDFLGLLLLLPPVQKHLTVKLMPHLRFSR
MPGGGFSAGTGGGNTFDGEYQRKDDERDRLDHKDDRQD
>P0AD68
MKAAAKTQKPKRQEEHANFISWRFALLCGCILLALAFLLGRVAWLQVISPDMLVKEGDMR
SLRVQQVSTSRGMITDRSGRPLAVSVPVKAIWADPKEVHDAGGISVGDRWKALANALNIP
LDQLSARINANPKGRFIYLARQVNPDMADYIKKLKLPGIHLREESRRYYPSGEVTAHLIG
FTNVDSQGIEGVEKSFDKWLTGQPGERIVRKDRYGRVIEDISSTDSQAAHNLALSIDERL
QALVYRELNNAVAFNKAESGSAVLVDVNTGEVLAMANSPSYNPNNLSGTPKEAMRNRTIT
DVFEPGSTVKPMVVMTALQRGVVRENSVLNTIPYRINGHEIKDVARYSELTLTGVLQKSS
NVGVSKLALAMPSSALVDTYSRFGLGKATNLGLVGERSGLYPQKQRWSDIERATFSFGYG
LMVTPLQLARVYATIGSYGIYRPLSITKVDPPVPGERVFPESIVRTVVHMMESVALPGGG
GVKAAIKGYRIAIKTGTAKKVGPDGRYINKYIAYTAGVAPASQPRFALVVVINDPQAGKY
YGGAVSAPVFGAIMGGVLRTMNIEPDALTTGDKNEFVINQGEGTGGRS
>P31135
MSTLEPAAQSKPPGGFKLWLSQLQMKHGRKLVIALPYIWLILLFLLPFLIVFKISLAEMA
RAIPPYTELMEWADGQLSITLNLGNFLQLTDDPLYFDAYLQSLQVAAISTFCCLLIGYPL
AWAVAHSKPSTRNILLLLVILPSWTSFLIRVYAWMGILKNNGVLNNFLLWLGVIDQPLTI
LHTNLAVYIGIVYAYVPFMVLPIYTALIRIDYSLVEAALDLGARPLKTFFTVIVPLTKGG
IIAGSMLVFIPAVGEFVIPELLGGPDSIMIGRVLWQEFFNNRDWPVASAVAIIMLLLLIV

PIMWFHKHQQKSVGEHG
>P37661
MRYIKSITQQKLSFLLAIYIGLFMNGAVFYRRFGSYAHDFTVWKGISAVVELAATVLVTF
FLLRLLSLFGRRSWRILASLVVLFSAGASYYMTFLNVVIGYGIIASVMTTDIDLSKEVVG
LNFILWLIAVSALPLILIWNNRCRYTLLRQLRTPGQRIRSLAVVVLAGIMVWAPIRLLDI
QQKKVERATGVDLPSYGGVVANSYLPSNWLSALGLYAWARVDESSDNNSLLNPAKKFTYQ
APQNVDDTYVVFIIGETTRWDHMGIFGYERNTTPKLAQEKNLAAFRGYSCDTATKLSLRC
MFVRQGGAEDNPQRTLKEQNIFAVLKQLGFSSDLYAMQSEMWFYSNTMADNIAYREQIGA
EPRNRGKPVDDMLLVDEMQQSLGRNPDGKHLIILHTKGSHFNYTQRYPRSFAQWKPECIG
VDSGCTKAQMINSYDNSVTYVDHFISSVIDQVRDKKAIVFYAADHGESINEREHLHGTPR
ELAPPEQFRVPMMVWMSDKYLENPANAQAFAQLKKEADMKVPRRHVELYDTIMGCLGYTS
PDGGINENNNWCHIPQAKEAAAN
>P75955
MKQKELWINQIKGLCICLVVIYHSVITFYPHLTTFQHPLSEVLSKCWIYFNLYLAPFRMP
VFFFISGYLIRRYIDSVPWGNCLDKRIWNIFWVLALWGVVQWLALSALNQWLAPERDLSN
ASNAAYADSTGEFLHGMITASTSLWYLYALIVYFVVCKIFSRLALPLFALFVLLSVAVNF
VPTPWWGMNSVIRNLPYYSLGAWFGATIMTCVKEVPLRRHLLMASLLTVLAVGAWLFTIS
LLLSLVSIVVIMKLFYQYEQRFGMRSTSLLNVIGSNTIAIYTTHRILVEIFSLTLLAQMN
AARWSPQVELTLLLVYPFVSLFICTVAGLLVRKLSQRAFSDLLFSPPSLPAAVSYSR
>P0ABM1
MWKTLHQLAIPPRLYQICGWFIPWLAIASVVVLTVGWIWGFGFAPADYQQGNSYRIIYLH
VPAAIWSMGIYASMAVAAFIGLVWQMKMANLAVAAMAPIGAVFTFIALVTGSAWGKPMWG
TWWVWDARLTSELVLLFLYVGVIALWHAFDDRRLAGRAAGILVLIGVVNLPIIHYSVEWW
NTLHQGSTRMQQSIDPAMRSPLRWSIFGFLLLSATLTLMRMRNLILLMEKRRPWVSELIL
KRGRK
>P75742
MNKHASQPRAIYYVVALQIWEYFSFYGMRALLILYLTNQLKYNDTHAYELFSAYCSLVYV
TPILGGFLADKVLGNRMAVMLGALLMAIGHVVLGASEIHPSFLYLSLAIIVCGYGLFKSN
VSCLLGELYEPTDPRRDGGFSLMYAAGNVGSIIAPIACGYAQEEYSWAMGFGLAAVGMIA
GLVIFLCGNRHFTHTRGVNKKVLRATNFLLPNWGWLLVLLVATPALITILFWKEWSVYAL
IVATIIGLGVLAKIYRKAENQKQRKELGLIVTLTFFSMLFWAFAQQGGSSISLYIDRFVN
RDMFGYTVPTAMFQSINAFAVMLCGVFLAWVVKESVAGNRTVRIWGKFALGLGLMSAGFC
ILTLSARWSAMYGHSSLPLMVLGLAVMGFAELFIDPVAMSQITRIEIPGVTGVLTGIYML
LSGAIANYLAGVIADQTSQASFDASGAINYSINAYIEVFDQITWGALACVGLVLMIWLYQ
ALKFRNRALALES
>P0A6Z3
MKGQETRGFQSEVKQLLHLMIHSLYSNKEIFLRELISNASDAADKLRFRALSNPDLYEGD
GELRVRVSFDKDKRTLTISDNGVGMTRDEVIDHLGTIAKSGTKSFLESLGSDQAKDSQLI
GQFGVGFYSAFIVADKVTVRTRAAGEKPENGVFWESAGEGEYTVADITKEDRGTEITLHL
REGEDEFLDDWRVRSIISKYSDHIALPVEIEKREEKDGETVISWEKINKAQALWTRNKSE
ITDEEYKEFYKHIAHDFNDPLTWSHNRVEGKQEYTSLLYIPSQAPWDMWNRDHKHGLKLY
VQRVFIMDDAEQFMPNYLRFVRGLIDSSDLPLNVSREILQDSTVTRNLRNALTKRVLQML
EKLAKDDAEKYQTFWQQFGLVLKEGPAEDFANQEAIAKLLRFASTHTDSSAQTVSLEDYV
SRMKEGQEKIYYITADSYAAAKSSPHLELLRKKGIEVLLLSDRIDEWMMNYLTEFDGKPF

QSVSKVDESLEKLADEVDESAKEAEKALTPFIDRVKALLGERVKDVRLTHRLTDTPAIVS
TDADEMSTQMAKLFAAAGQKVPEVKYIFELNPDHVLVKRAADTEDEAKFSEWVELLLDQA
LLAERGTLEDPNLFIRRMNQLLVS
>Q51393
MTQSISRPLQYAYIAAFGGLLLGLAGWSLKSVPGFSAAADTPLLNGKLAHAFEAHYDKEF
PIKRLGTNLWAALDYTLFHEGRPGVVIGKDGWLFTDEEFKPAPSGQQLEDNWALVRGVQR
ELNRRGVKLVLAVIPAKARLYPEHIGREQPAALHDSLYQDFLARARAAGIDSPDLLGSLR
QAKDNGAVFLRTDTHWSPLGAETVAQRLGAEIRETHLLDVPAQNFVTRVGEERTHKGDLL
SFLPLDPLFDELLPRPEQLQQRTTEAAPALPGGQQSGAGDDLFGDSQQPRLALVGTSYSA
NPRWNFEGALKQALSADLINYAKEGKGPLEPMLELLQDEGFRKDPPQLLVWEFPERYLPM
ASDLSQFDADWVAQLKASGGRDERLAASRND
>P69801
MEITTLQIVLVFIVACIAGMGSILDEFQFHRPLIACTLVGIVLGDMKTGIIIGGTLEMIA
LGWMNIGAAVAPDAALASIISTILVIAGHQSIGAGIALAIPLAAAGQVLTIIVRTITVAF
QHAADKAADNGNLTAISWIHVSSLFLQAMRVAIPAVIVALSVGTSEVQNMLNAIPEVVTN
GLNIAGGMIVVVGYAMVINMMRAGYLMPFFYLGFVTAAFTNFNLVALGVIGTVMAVLYIQ
LSPKYNRVAGAPAQAAGNNDLDNELD
>P76343
MRLTAKQVTWLKVCLHLAGLLPFLWLVWAINHGGLGADPVKDIQHFTGRTALKFLLATLL
ITPLARYAKQPLLIRTRRLLGLWCFAWATLHLTSYALLELGVNNLALLGKELITRPYLTL
GIISWVILLALAFTSTQAMQRKLGKHWQQLHNFVYLVAILAPIHYLWSVKIISPQPLIYA
GLAVLLLALRYKKLRSLFNRLRKQVHNKLSV
>P22708
MSFNAKDMTQGGQIASMRIRMFSQIANIMLYCLFIFFWILIGLVLWVKISWQTFINGCIY
WWCTSLEGMRDLIKSQPVYEIQYYGKTFRMNAAQVLHDKYMIWCGEQLWSAFVLASVVAL
VICLITFFVVSWILGRQGKQQSENEVTGGRQLTDNPKDVARMLKKDGKDSDIRIGDLPII
RDSEIQNFCLHGTVGAGKSEVIRRLANYARQRGDMVVIYDRSGEFVKSYYDPSIDKILNP
LDARCAAWDLWKECLTQPDFDNTANTLIPMGTKEDPFWQGSGRTIFAEAAYLMRNDPNRS
YSKLVDTLLSIKIEKLRTFLRNSPAANLVEEKIEKTAISIRAVLTNYVKAIRYLQGIEHN
GDPFTIRDWMRGVREDQKNGWLFISSNADTHASLKPVISMWLSIAIRGLLAMGENRNRRV
WFFCDELPTLHKLPDLVEILPEARKFGGCYVFGIQSYAQLEDIYGEKAAATLFDVMNTRA
FFRSPSHKIAEFAAGEIGEKEHLKASEQYSYGADPVRDGVSTGKDMERQTLVSYSDIQSL
PDLTCYVTLPGPYPAVKLSLKYQARPKVAPEFIPRDINPEMENRLSAVLAAREAEGRQMA
SLFEPEVASGEDVTQAEQPQQPQQPQQPQQPQQPQQPQQPQQPQQPVSSVINDKKSDAGV
SVPAGGIEQELKMKPEEEMEQQLPPGISESGEVVDMAAYEAWQQENHPDIQQHMQRREEV
NINVHRERGEDVEPGDDF
>Q2YQ73
MSLLKIYWRAMQYLAVERTATITMCVASVLVALVTLAEPVLFGRVIQSISDKGDIFSPLL
MWAALGGFNIMAAVFVARGADRLAHRRRLGVMIDSYERLITMPLAWHQKRGTSNALHTLI
RATDSLFTLWLEFMRQHLTTVVALATLIPVAMTMDMRMSLVLIVLGVIYVMIGQLVMRKT
KDGQAAVEKHHHKLFEHVSDTISNVSVVQSYNRIASETQALRDYAKNLENAQFPVLNWWA
LASGLNRMASTFSMVVVLVLGAYFVTKGQMRVGDVIAFIGFAQLMIGRLDQISAFINQTV
TARAKLEEFFQMEDATADRQEPENVADLNDVKGDIVFDNVTYEFPNSGQGVYDVSFEVKP
GQTVAIVGPTGAGKTTLINLLQRVFDPAAGRIMIDGTDTRTVSRRSLRHAIATVFQDAGL

FNRSVEDNIRVGRANATHEEVHAAAKAAAAHDFILAKSEGYDTFVGERGSQLSGGERQRL
AIARAILKDSPILVLDEATSALDVETEEKVTQAVDELSHNRTTFIIAHRLSTVRSADLVL
FMDKGHLVESGSFNELAERGGRFSDLLRAGGLKLEDKQPKQPVVEGSNVMPFPVKGAVA
>Q05807
MMKVIFTSNRFIDFLIRLLITAIVISPVIIWSWDTVKETTADGMLAAAFVILYSGVLLFI
LYFCFSALTDLQKPDERKSDERNEDE
>P09127
MTEQEKTSAVVEETREAVDTTSQPVATEKKSKNNTALILSAVAIAIALAAGIGLYGWGKQ
QAVNQTATSDALANQLTALQKAQESQKAELEGIIKQQAAQLKQANRQQETLAKQLDEVQQ
KVATISGSDAKTWLLAQADFLVKLAGRKLWSDQDVTTAAALLKSADASLADMNDPSLITV
RRAITDDIASLSAVSQVDYDGIILKLNQLSNQVDNLRLADNDSDGSPMDSDGEELSSSIS
EWRINLQKSWQNFMDNFITIRRRDDTAVPLLAPNQDIYLRENIRSRLLVAAQAVPRHQEE
TYRQALENVSTWVRAYYDTDDATTKAFLDEVDQLSQQNISMDLPETLQSQAMLEKLMQTR
VRNLLAQPAAGTTEAKPAPAPQADTPAAAPQGE
>Q46867
MLKQKIKTIFEALLYIMLTYWLIDSFFAFNKYDWMLESGGNICSIPSVSGEDRILQAMIA
AFFLLTPLIIILILRKLFMREMFEFWVYVFSLGICLVCGWWLFWGRFIFCY
>P0ABL8
MMFWRIFRLELRVAFRHSAEIANPLWFFLIVITLFPLSIGPEPQLLARIAPGIIWVAALL
SSLLALERLFRDDLQDGSLEQLMLLPLPLPAVVLAKVMAHWMVTGLPLLILSPLVAMLLG
MDVYGWQVMALTLLLGTPTLGFLGAPGVALTVGLKRGGVLLSILVLPLTIPLLIFATAAM
DAASMHLPVDGYLAILGALLAGTATLSPFATAAALRISIQ
>P69739
MNNEETFYQAMRRQGVTRRSFLKYCSLAATSLGLGAGMAPKIAWALENKPRIPVVWIHGL
ECTCCTESFIRSAHPLAKDVILSLISLDYDDTLMAAAGTQAEEVFEDIITQYNGKYILAV
EGNPPLGEQGMFCISSGRPFIEKLKRAAAGASAIIAWGTCASWGCVQAARPNPTQATPID
KVITDKPIIKVPGCPPIPDVMSAIITYMVTFDRLPDVDRMGRPLMFYGQRIHDKCYRRAH
FDAGEFVQSWDDDAARKGYCLYKMGCKGPTTYNACSSTRWNDGVSFPIQSGHGCLGCAEN
GFWDRGSFYSRVVDIPQMGTHSTADTVGLTALGVVAAAVGVHAVASAVDQRRRHNQQPTE
TEHQPGNEDKQA
>P0AAA1
MNIFEQTPPNRRRYGLAAFIGLIAGVVSAFVKWGAEVPLPPRSPVDMFNAACGPESLIRA
AGQIDCSRNFLNPPYIFLRDWLGLTDPNAAVYTFAGHVFNWVGVTHIIFSIVFAVGYCVV
AEVFPKIKLWQGLLAGALAQLFVHMISFPLMGLTPPLFDLPWYENVSEIFGHLVWFWSIE
IIRRDLRNRITHEPDPEIPLGSNR
>Q09068
MLGLVLLYVGIVLISNGICGLTKVDPKSTAVMNFFVGGLSIICNIVVITYSALHPTAPVE
GAEDIAQVSHHLTSFYGPATGLLFGFTYLYAAINHTFGLDWRPYSWYSLFVAINTIPAAI
LSHYSDMLDDHKVLGITEGDWWAIIWLAWGVLWLTAFIENILKIPLGKFTPWLAIIEGIL
TAWIPAWLLFIQHWV
>P0ABJ6
MSHSTDHSGASHGSVKTYMTGFILSIILTVIPFWMVMTGAASPAVILGTILAMAVVQVLV
HLVCFLHMNTKSDEGWNMTAFVFTVLIIAILVVGSIWIMWNLNYNMMMH
>Q46841

MAGFNIKHWFADGAFRTIIRNSAWLGSSNVVSALLGLLALSCAGKGMTPAMFGVLVIVQS
YAKSISDFIKFQTWQLVVQYGTPALTNNNPQQFRNVVSFSFSLDIVSGAVAIVGGIALLP
FLSHSLGLDDQSFWLAALYCTLIPSMASSTPTGILRAVDRFDLIAVQQATKPFLRAAGSV
VAWYFDFGFAGFVIAWYVSNLVGGTMYWWFAARELRRRNIHNAFKLNLFESARYIKGAWS
FVWSTNIAHSIWSARNSCSTVLVGIVLGPAAAGLFKIAMTFFDAAGTPAGLLGKSFYPEV
MRLDPRTTRPWLLGVKSGLLAGGIGILVALAVLIVGKPLISLVFGVKYLEAYDLI
>P23849
MNTSHVRVVTHMCGFLVWLYSLSMLPPMVVALFYKEKSLFVFFITFVIFFCIGGGAWYTT
KKSGIQLRTRDGFIIIVMFWILFSVISAFPLWIDSELNLTFIDALFEGVSGITTTGATVI
DDVSSLPRAYLYYRSQLNFIGGLGVIVLAVAVLPLLGIGGAKLYQSEMPGPFKDDKLTPR
LADTSRTLWITYSLLGIACIVCYRLAGMPLFDAICHGISTVSLGGFSTHSESIGYFNNYL
VELVAGSFSLLSAFNFTLWYIVISRKTIKPLIRDIELRFFLLIALGVIIVTSFQVWHIGM
YDLHGSFIHSFFLASSMLTDNGLATQDYASWPTHTIVFLLLSSFFGGCIGSTCGGIKSLR
FLILFKQSKHEINQLSHPRALLSVNVGGKIVTDRVMRSVWSFFFLYTLFTVFFILVLNGM
GYDFLTSFATVAACINNMGLGFGATASSFGVLNDIAKCLMCIAMILGRLEIYPVIILFSG
FFWRS
>P15877
MAINNTGSRRLLVTLTALFAALCGLYLLIGGGWLVAIGGSWYYPIAGLVMLGVAWMLWRS
KRAALWLYAALLLGTMIWGVWEVGFDFWALTPRSDILVFFGIWLILPFVWRRLVIPASGA
VAALVVALLISGGILTWAGFNDPQEINGTLSADATPAEAISPVADQDWPAYGRNQEGQRF
SPLKQINADNVHNLKEAWVFRTGDVKQPNDPGEITNEVTPIKVGDTLYLCTAHQRLFALD
AASGKEKWHYDPELKTNESFQHVTCRGVSYHEAKAETASPEVMADCPRRIILPVNDGRLI
AINAENGKLCETFANKGVLNLQSNMPDTKPGLYEPTSPPIITDKTIVMAGSVTDNFSTRE
TSGVIRGFDVNTGELLWAFDPGAKDPNAIPSDEHTFTFNSPNSWAPAAYDAKLDLVYLPM
GVTTPDIWGGNRTPEQERYASSILALNATTGKLAWSYQTVHHDLWDMDLPAQPTLADITV
NGQKVPVIYAPAKTGNIFVLDRRNGELVVPAPEKPVPQGAAKGDYVTPTQPFSELSFRPT
KDLSGADMWGATMFDQLVCRVMFHQMRYEGIFTPPSEQGTLVFPGNLGMFEWGGISVDPN
REVAIANPMALPFVSKLIPRGPGNPMEQPKDAKGTGTESGIQPQYGVPYGVTLNPFLSPF
GLPCKQPAWGYISALDLKTNEVVWKKRIGTPQDSMPFPMPVPVPFNMGMPMLGGPISTAG
NVLFIAATADNYLRAYNMSNGEKLWQGRLPAGGQATPMTYEVNGKQYVVISAGGHGSFGT
KMGDYIVAYALPDDVK
>P69826
MENKSARAKVQAFGGFLTAMVIPNIGAFIAWGFITALFIPTGWLPNEHFAKIVGPMITYL
LPVMIGSTGGHLVGGKRGAVMGGIGTIGVIVGAEIPMFLGSMIMGPLGGLVIKYVDKALE
KRIPAGFEMVINNFSLGIAGMLLCLLGFEVIGPAVLIANTFVKECIEALVHAGYLPLLSV
INEPAKVLFLNNAIDQGVYYPLGMQQASVNGKSIFFMVASNPGPGLGLLLAFTLFGKGMS
KRSAPGAMIIHFLGGIHELYFPYVLMKPLTIIAMIAGGMSGTWMFNLLDGGLVAGPSPGS
IFAYLALTPKGSFLATIAGVTVGTLVSFAITSLILKMEKTVETESEDEFAQSANAVKAMK
QEGAFSLSRVKRIAFVCDAGMGSSAMGATTFRKRLEKAGLAIEVKHYAIENVPADADIVV
THASLEGRVKRVTDKPLILINNYIGDPKLDTLFNQLTAEHKH
>P29918
MTGLNTAGADRDLATAELNRELQDKGFLLTTTEDIINWARNGSLHWMTFGLACCAVEMMQ
TSMPRYDLERFGTAPRASPRQSDLMIVAGTLTNKMAPALRKVYDQMPEPRYVISMGSCAN
GGGYYHYSYSVVRGCDRIVPVDIYVPGCPPTAEALLYGILQLQRRASGAPARW

>O85093
MSALRLRKVDALLAQATRELGAGQSLGFSAAGQDAELTLLPLLADAGEPAGAVWLSTAIG
PLLLSDAEALLSLLGDIPLTLGGEQQAWYWQLFNQRLSPTVARLLAPVEPLHNKPQAPTL
GCRVQIRRGGEQLHAHMHATPDTLLRLLRSASWQARTRTVDESWSVASPLIIGEMSLTRE
QIASLRPGDVVLPAHCQFDSAGQGFLSLAGRQWAAQTDQHAQRLFLRLSHEEHRHHEY
>P76299
MSDESDDKTEAPTPHRLEKAREEGQIPRSRELTSLLILLVGVSVIWFGGVSLARRLSGML
SAGLHFDHSIINDPNLILGQIILLIREAMLALLPLISGVVLVALISPVMLGGLVFSGKSL
QPKFSKLNPLPGIKRMFSAQTGAELLKAILKTILVGSVTGFFLWHHWPQMMRLMAESPIT
AMGNAMDLVGLCALLVVLGVIPMVGFDVFFQIFSHLKKLRMSRQDIRDEFKQSEGDPHVK
GRIRQMQRAAARRRMMADVPKADVIVNNPTHYSVALQYDENKMSAPKVVAKGAGLVALRI
REIGAENNVPTLEAPPLARALYRHAEIGQQIPGQLYAAVAEVLAWVWQLKRWRLAGGQRP
VQPTHLPVPEALDFINEKPTHE
>P0ADD5
MQTEQQRAVTRLCIQCGLFLLQHGAESALVDELSSRLGRALGMDSVESSISSNAIVLTTI
KDGQCLTSTRKNHDRGINMHVVTEVQHIVILAEHHLLDYKGVEKRFSQIQPLRYPRWLVA
LMVGLSCACFCKLNNGGWDGAVITFFASTTAMYIRQLLAQRHLHPQINFCLTAFAATTIS
GLLLQLPTFSNTPTIAMAASVLLLVPGFPLINAVADMFKGHINTGLARWAIASLLTLATC
VGVVMALTIWGLRGWV
>P0AAC4
MDRFPRSDSIVQPRAGLQTYMAQVYGWMTVGLLLTAFVAWYAANSAAVMELLFTNRVFLI
GLIIAQLALVIVLSAMIQKLSAGVTTMLFMLYSALTGLTLSSIFIVYTAASIASTFVVTA
GMFGAMSLYGYTTKRDLSGFGNMLFMALIGIVLASLVNFWLKSEALMWAVTYIGVIVFVG
LTAYDTQKLKNMGEQIDTRDTSNLRKYSILGALTLYLDFINLFLMLLRIFGNRR
>P76334
MEKCDFYHIIVLSLNFPGYLKMEYGSTKMEERLSRSPGGKLALWAFYTWCGYFVWAMARY
IWVMSRIPDAPVSGFESDLGSTAGKWLGALVGFLFMALVGALLGSIAWYTRPRPARSRRY
E
>P62517
MNKTTEYIDAMPIAASEKAALPKTDIRAVHQALDAEHRTWAREDDSPQGSVKARLEQAWP
DSLADGQLIKDDEGRDQLKAMPEAKRSSMFPDPWRTNPVGRFWDRLRGRDVTPRYLARLT
KEEQESEQKWRTVGTIRRYILLILTLAQTVVATWYMKTILPYQGWALINPMDMVGQDLWV
SFMQLLPYMLQTGILILFAVLFCWVSAGFWTALMGFLQLLIGRDKYSISASTVGDEPLNP
EHRTALIMPICNEDVNRVFAGLRATWESVKATGNAKHFDVYILSDSYNPDICVAEQKAWM
ELIAEVGGEGQIFYRRRRRRVKRKSGNIDDFCRRWGSQYSYMVVLDADSVMTGDCLCGLV
RLMEANPNAGIIQSSPKASGMDTLYARCQQFATRVYGPLFTAGLHFWQLGESHYWGHNAI
IRVKPFIEHCALAPLPGEGSFAGSILSHDFVEAALMRRAGWGVWIAYDLPGSYEELPPNL
LDELKRDRRWCHGNLMNFRLFLVKGMHPVHRAVFLTGVMSYLSAPLWFMFLALSTALQVV
HALTEPQYFLQPRQLFPVWPQWRPELAIALFASTMVLLFLPKLLSILLIWCKGTKEYGGF
WRVTLSLLLEVLFSVLLAPVRMLFHTVFVVSAFLGWEVVWNSPQRDDDSTSWGEAFKRHG
SQLLLGLVWAVGMAWLDLRFLFWLAPIVFSLILSPFVSVISSRATVGLRTKRWKLFLIPE
EYSPPQVLVDTDRFLEMNRQRSLDDGFMHAVFNPSFNALATAMATARHRASKVLEIARDR
HVEQALNETPEKLNRDRRLVLLSDPVTMARLHFRVWNSPERYSSWVSYYEGIKLNPLALR
KPDAASQ

>P58593
MTQNLSQPPAVNAPESELDLVRYLDVLVANRWLIAGIAAVVMLLGATYAFLARPVYEADV
LVQVEDNPNSAKSLLGDVSSLFDVKTDANAEIEILRSRMVVGKAVDNLHLYITAKPHYFP
LIGAWVASRAKQLSEPGLFGLGGYTWGTELIDVDGFDVPEALEGQPFKLTALGNGRYRLE
NKSLDTPIEGVVGEPLEAKQSVGTIQLLVNTLAAKAGAAFELQRDSRLKTLEMLQDKLKI
SEKGKQSGIIGASLEGKNPALTAAIMNQIATEYVAQNIKRKAEEAERSLVFLDGLLPQLK
LQLERAEMKYNEMRNLRGTFDLSEEGKAFLQESVTTETSLQELKQKRAELLTRFTASHPG
VQAIDQQIAVMSGKVGAMTRRLKSLPNIEQDTVRLMRDVQVDNDLYVSLLNDMQQLKLVK
AGKVGNVRLVDGAAVPEEPVKPKKLTVTALAGVLGVVLGVVAAFVRNTLFGGITEPQDIE
EHTGLSVYATVPLSDVQIDLSSQLTTHKRGQYLLARRVPDDPSIESLRSLRTALQFAMQD
SGNNLVVLTGPTPGVGKSFVSANLAAVIATGGKRVLLVDADMRKGYLHQYFGKDRKPGLL
DLLAGDRSIEQVVHREVVPGLDFIATGLFPHNPSELLLNPRMVELMDTFRAQYDLVLIDT
PPVLAVTDTAILAARAGTVLMVTRFERSTLGEIRETIKQLQHANVEVRGVVFNALDPNTY
RYGYGSRYGRYRYVQYGYTSKPSAEAEAESA
>P76228
MKKVLLQNHPGSEKYSFNGWEIFNSNFERMIKENKAMLLCKWGFYLTCVVAVMFVFAAIT
SNGLNERGLITAGCSFLYLLIMMGLIVRAGFKAKKEQLHYYQAKGIEPLSIEKLQALQLI
APYRFYHKQWSETLEFWPRKPEPGKDTFQYHVLPFDSIDIISKRRESLEDQWGIEDSESY
CALMEHFLSGDHGANTFKANMEEAPEQVIALLNKFAVFPSDYISDCANHSSGKSSAKLIW
AAELSWMISISSTAFQNGTIEEELAWHYIMLASRKAHELFESEEDYQKNSQMGFLYWHIC
CYRRKLTDAELEACYRYDKQFWEHYSKKCRWPIRNVPWGASSVKYS
>P0ABK2
MIDYEVLRFIWWLLVGVLLIGFAVTDGFDMGVGMLTRFLGRNDTERRIMINSIAPHWDGN
QVWLITAGGALFAAWPMVYAAAFSGFYVAMILVLASLFFRPVGFDYRSKIEETRWRNMWD
WGIFIGSFVPPLVIGVAFGNLLQGVPFNVDEYLRLYYTGNFFQLLNPFGLLAGVVSVGMI
ITQGATYLQMRTVGELHLRTRATAQVAALVTLVCFALAGVWVMYGIDGYVVKSTMDHYAA
SNPLNKEVVREAGAWLVNFNNTPILWAIPALGVVLPLLTILTARMDKAAWAFVFSSLTLA
CIILTAGIAMFPFVMPSSTMMNASLTMWDATSSQLTLNVMTWVAVVLVPIILLYTAWCYW
KMFGRITKEDIERNTHSLY
>P24586
MIKIENLTKSYRTPTGRHYVFKDLNIEIPSGKSVAFIGRNGAGKSTLLRMIGGIDRPDSG
KIITNKTISWPVGLAGGFQGSLTGRENVKFVARLYAKQEELKEKIEFVEEFAELGKYFDM
PIKTYSSGMRSRLGFGLSMAFKFDYYIVDEVTAVGDARFKEKCAQLFKERHKESSFLMVS
HSLNSLKEFCDVAIVFKNSYIIGYYENVQSGIDEYKMYQDLDIE
>P0ADZ7
MSFFISDAVAATGAPAQGSPMSLILMLVVFGLIFYFMILRPQQKRTKEHKKLMDSIAKGD
EVLTNGGLVGRVTKVAENGYIAIALNDTTEVVIKRDFVAAVLPKGTMKAL
>P77328
MFNFAVSRESLLSGFQWFFFIFCNTVVVPPTLLSAFQLPQSSLLTLTQYAFLATALACFA
QAFCGHRRAIMEGPGGLWWGTILTITLGEASRGTPINDIATSLAVGIALSGVLTMLIGFS
GLGHRLARLFTPSVMVLFMLMLGAQLTTIFFKGMLGLPFGIADPNFKIQLPPFALSVAVM
CLVLAMIIFLPQRFARYGLLVGTITGWLLWYFCFPSSHSLSGELHWQWFPLGSGGALSPG
IILTAVITGLVNISNTYGAIRGTDVFYPQQGAGNTRYRRSFVATGFMTLITVPLAVIPFS
PFVSSIGLLTQTGDYTRRSFIYGSVICLLVALVPALTRLFCSIPLPVSSAVMLVSYLPLL

FSALVFSQQITFTARNIYRLALPLFVGIFLMALPPVYLQDLPLTLRPLLSNGLLVGILLA
VLMDNLIPWERIE
>P0A8S5
MISTVALFWALCVVCIVNMARYFSSLRALLVVLRNCDPLLYQYVDGGGFFTSHGQPNKQV
RLVWYIYAQRYRDHHDDEFIRRCERVRRQFILTSALCGLVVVSLIALMIWH
>P0AF52
MSTPSARTGGSLDAWFKISQRGSTVRQEVVAGLTTFLAMVYSVIVVPGMLGKAGFPPAAV
FVATCLVAGLGSIVMGLWANLPLAIGCAISLTAFTAFSLVLGQHISVPVALGAVFLMGVL
FTVISATGIRSWILRNLPHGVAHGTGIGIGLFLLLIAANGVGLVIKNPLDGLPVALGDFA
TFPVIMSLVGLAVIIGLEKLKVPGGILLTIIGISIVGLIFDPNVHFSGVFAMPSLSDENG
NSLIGSLDIMGALNPVVLPSVLALVMTAVFDATGTIRAVAGQANLLDKDGQIIDGGKALT
TDSMSSVFSGLVGAAPAAVYIESAAGTAAGGKTGLTAITVGVLFLLILFLSPLSYLVPGY
ATAPALMYVGLLMLSNVAKIDFADFVDAMAGLVTAVFIVLTCNIVTGIMIGFATLVIGRL
VSGEWRKLNIGTVVIAVALVTFYAGGWAI
>P02919
MAGNDREPIGRKGKPTRPVKQKVSRRRYEDDDDYDDYDDYEDEEPMPRKGKGKGKGRKPR
GKRGWLWLLLKLAIVFAVLIAIYGVYLDQKIRSRIDGKVWQLPAAVYGRMVNLEPDMTIS
KNEMVKLLEATQYRQVSKMTRPGEFTVQANSIEMIRRPFDFPDSKEGQVRARLTFDGDHL
ATIVNMENNRQFGFFRLDPRLITMISSPNGEQRLFVPRSGFPDLLVDTLLATEDRHFYEH
DGISLYSIGRAVLANLTAGRTVQGASTLTQQLVKNLFLSSERSYWRKANEAYMALIMDAR
YSKDRILELYMNEVYLGQSGDNEIRGFPLASLYYFGRPVEELSLDQQALLVGMVKGASIY
NPWRNPKLALERRNLVLRLLQQQQIIDQELYDMLSARPLGVQPRGGVISPQPAFMQLVRQ
ELQAKLGDKVKDLSGVKIFTTFDSVAQDAAEKAAVEGIPALKKQRKLSDLETAIVVVDRF
SGEVRAMVGGSEPQFAGYNRAMQARRSIGSLAKPATYLTALSQPKIYRLNTWIADAPIAL
RQPNGQVWSPQNDDRRYSESGRVMLVDALTRSMNVPTVNLGMALGLPAVTETWIKLGVPK
DQLHPVPAMLLGALNLTPIEVAQAFQTIASGGNRAPLSALRSVIAEDGKVLYQSFPQAER
AVPAQAAYLTLWTMQQVVQRGTGRQLGAKYPNLHLAGKTGTTNNNVDTWFAGIDGSTVTI
TWVGRDNNQPTKLYGASGAMSIYQRYLANQTPTPLNLVPPEDIADMGVDYDGNFVCSGGM
RILPVWTSDPQSLCQQSEMQQQPSGNPFDQSSQPQQQPQQQPAQQEQKDSDGVAGWIKDM
FGSN
>P0AFA2
MLKRCLSPLTLVNQVALIVLLSTAIGLAGMAVSGWLVQGVQGSAHAINKAGSLRMQSYRL
LAAVPLSEKDKPLIKEMEQTAFSAELTRAAERDGQLAQLQGLQDYWRNELIPALMRAQNR
ETVSADVSQFVAGLDQLVSGFDRTTEMRIETVVLVHRVMAVFMALLLVFTIIWLRARLLQ
PWRQLLAMASAVSHRDFTQRANISGRNEMAMLGTALNNMSAELAESYAVLEQRVQEKTAG
LEHKNQILSFLWQANRRLHSRAPLCERLSPVLNGLQNLTLLRDIELRVYDTDDEENHQEF
TCQPDMTCDDKGCQLCPRGVLPVGDRGTTLKWRLADSHTQYGILLATLPQGRHLSHDQQQ
LVDTLVEQLTATLALDRHQERQQQLIVMEERATIARELHDSIAQSLSCMKMQVSCLQMQG
DALPESSRELLSQIRNELNASWAQLRELLTTFRLQLTEPGLRPALEASCEEYSAKFGFPV
KLDYQLPPRLVPSHQAIHLLQIAREALSNALKHSQASEVVVTVAQNDNQVKLTVQDNGCG
VPENAIRSNHYGMIIMRDRAQSLRGDCRVRRRESGGTEVVVTFIPEKTFTDVQGDTHE
>P77338
MTMFQYYKRSRHFVFSAFIAFVFVLLCQNTAFARASSNGDLPTKADLQAQLDSLNKQKDL
SAQDKLVQQDLTDTLATLDKIDRIKEETVQLRQKVAEAPEKMRQATAALTALSDVDNDEE

TRKILSTLSLRQLETRVAQALDDLQNAQNDLASYNSQLVSLQTQPERVQNAMYNASQQLQ
QIRSRLDGTDVGETALRPSQKVLMQAQQALLNAEIDQQRKSLEGNTVLQDTLQKQRDYVT
ANSARLEHQLQLLQEAVNSKRLTLTEKTAQEAVSPDEAARIQANPLVKQELEINQQLSQR
LITATENGNQLMQQNIKVKNWLERALQSERNIKEQIAVLKGSLLLSRILYQQQQTLPSAD
ELENMTNRIADLRLEQFEVNQQRDALFQSDAFVNKLEEGHTNEVNSEVHDALLQVVDMRR
ELLDQLNKQLGNQLMMAINLQINQQQLMSVSKNLKSILTQQIFWVNSNRPMDWDWIKAFP
QSLKDEFKSMKITVNWQKAWPAVFIAFLAGLPLLLIAGLIHWRLGWLKAYQQKLASAVGS
LRNDSQLNTPKAILIDLIRALPVCLIIILAVGLILLTMQLNISELLWSFSKKLAIFWLVFG
LCWKVLEKNGVAVRHFGMPEQQTSHWRRQIVRISLALLPIHFWSVVAELSPLHLMDDVLG
QAMIFFNLLLIAFLVWPMCRESWRDKESHTMRLVTITVLSIIPIALMVLTATGYFYTTLR
LAGRWIETVYLVIIWNLLYQTVLRGLSVAARRIAWRRALARRQNLVKEGAEGAEPPEEPT
IALEQVNQQTLRITMLLMFALFGVMFWAIWSDLITVFSYLDSITLWHYNGTEAGAAVVKN
VTMGSLLFAIIASMVAWALIRNLPGLLEVLVLSRLNMRQGASYAITTILNYIIIAVGAMT
VFGSLGVSWDKLQWLAAALSVGLGFGLQEIFGNFVSGLIILFERPVRIGDTVTIGSFSGT
VSKIRIRATTITDFDRKEVIIPNKAFVTERLINWSLTDTTTRLVIRLGVAYGSDLEKVRK
VLLKAATEHPRVMHEPMPEVFFTAFGASTLDHELRLYVRELRDRSRTVDELNRTIDQLCR
ENDINIAFNQLEVHLHNEKGDEVTEVKRDYKGDDPTPAVG
>P37180
MSHDPQPLGGKIISKPVMIFGPLIVICMLLIVKRLVFGLGSVSDLNGGFPWGVWIAFDLL
IGTGFACGGWALAWAVYVFNRGQYHPLVRPALLASLFGYSLGGLSITIDVGRYWNLPYFY
IPGHFNVNSVLFETAVCMTIYIGVMALEFAPALFERLGWKVSLQRLNKVMFFIIALGALL
PTMHQSSMGSLMISAGYKVHPLWQSYEMLPLFSLLTAFIMGFSIVIFEGSLVQAGLRGNG
PDEKSLFVKLTNTISVLLAIFIVLRFGELIYRDKLSLAFAGDFYSVMFWIEVLLMLFPLV
VLRVAKLRNDSRMLFLSALSALLGCATWRLTYSLVAFNPGGGYAYFPTWEELLISIGFVA
IEICAYIVLIRLLPILPPLKQNDHNRHEASKA
>P0AEA5
MMFKQYLQVTKPGIIFGNLISVIGGFLLASKGSIDYPLFIYTLVGVSLVVASGCVFNNYI
DRDIDRKMERTKNRVLVKGLISPAVSLVYATLLGIAGFMLLWFGANPLACWLGVMGFVVY
VGVYSLYMKRHSVYGTLIGSLSGAAPPVIGYCAVTGEFDSGAAILLAIFSLWQMPHSYAI
AIFRFKDYQAANIPVLPVVKGISVAKNHITLYIIAFAVATLMLSLGGYAGYKYLVVAAAV
SVWWLGMALRGYKVADDRIWARKLFGFSIIAITALSVMMSVDFMVPDSHTLLAAVW
>P0AA73
MKQQAGIGILLALTTAICWGALPIAMKQVLEVMEPPTIVFYRFLMASIGLGAILAVKKRL
PPLRVFRKPRWLILLAVATAGLFGNFILFSSSLQYLSPTASQVIGQLSPVGMMVASVFIL
KEKMRSTQVVGALMLLSGLVMFFNTSLVEIFTKLTDYTWGVIFGVGAATVWVSYGVAQKV
LLRRLASPQILFLLYTLCTIALFPLAKPGVIAQLSHWQLACLIFCGLNTLVGYGALAEAM
ARWQAAQVSAIITLTPLFTLFFSDLLSLAWPDFFARPMLNLLGYLGAFVVVAGAMYSAIG
HRIWGGLRKHTTVVSQPRAGE
>P23516
MFQLLAGVRMNSTGRPRAKIILLYALLIAFNIGAWLCALAAFRDHPVLLGTALLAYGLGL
RHAVDADHLAAIDNVTRKLMQDGRRPITAGLWFSLGHSSVVVLASVLIAVMATTLQERLD
AFHEVGSVIGTLASALFLFAIAAINLVILRSAYRAFRRVRRGGIYVEEDFDLLFGNRGFL
ARIFRPLFRFITRSWHMYPLGMLFALGFDTATEVALLGISTMEASRGVPIWSILVFPALF
TAGMALIDTIDSILMCGAYAWAYAKPVRKLYYNMTITFVSAIVALIVGGIETLGLLADKF

MLKGVFWNAVGALNENFCQLGFVIIGIFTVCWVVSIVVYRLRRYDDSEVRA
>P77682
MLKLFAKYTSIGVLNTLIHWVVFGVCIYVAHTNQALANFAGFVVAVSFSFFANAKFTFKA
STTTMRYMLYVGFMGTLSATVGWAADRCALPPMITLVTFSAISLVCGFVYSKFIVFRDAK

>P80586
MTNGKIWLVVKPTVGLPIGMLFAALLAVLIHGLLFVDGRLKSWWSEFPVAKPAVVSVQAA
PAPVAAEVK
>P0AB98
MASENMTPQDYIGHHLNNLQLDLRTFSLVDPQNPPATFWTINIDSMFFSVVLGLLFLVLF
RSVAKKATSGVPGKFQTAIELVIGFVNGSVKDMYHGKSKLIAPLALTIFVWVFLMNLMDL
LPIDLLPYIAEHVLGLPALRVVPSADVNVTLSMALGVFILILFYSIKMKGIGGFTKELTL
QPFNHWAFIPVNLILEGVSLLSKPVSLGLRLFGNMYAGELIFILIAGLLPWWSQWILNVP
WAIFHILIITLQAFIFMVLTIVYLSMASEEH
>P0AEC0
MEFLMDPSIWAGLLTLVVLEIVLGIDNLVFIAILADKLPPKQRDKARLLGLSLALIMRLG
LLSLISWMVTLTKPLFTVMDFSFSGRDLIMLFGGIFLLFKATTELHERLENRDHDSGHGK
GYASFWVVVTQIVILDAVFSLDAVITAVGMVNHLPVMMAAVVIAMAVMLLASKPLTRFVN
QHPTVVVLCLSFLLMIGLSLVAEGFGFHIPKGYLYAAIGFSIIIEVFNQIARRNFIRHQS
TLPLRARTADAILRLMGGKRQANVQHDADNPMPMPIPEGAFAEEERYMINGVLTLASRSL
RGIMTPRGEISWVDANLGVDEIREQLLSSPHSLFPVCRGELDEIIGIVRAKELLVALEEG
VDVAAIASASPAIIVPETLDPINLLGVLRRARGSFVIVTNEFGVVQGLVTPLDVLEAIAG
EFPDADETPEIITDGDGWLVKGGTDLHALQQALDVEHLADDDDIATVAGLVISANGHIPR
VGDVIDVGPLHITIIEANDYRVDLVRIVKEQPAHDEDE
>Q3J6C1
MILGPDGILNRDTRGDWVRLRTLILLRWMAVAGQLAAIVVTDWYLGVRLPMGLCFMAVGA
SVIANVIATFVFPQNRRLTEFQALMILLFDLTQLSFLLFLTGGLTNPFALLILAPVTISA
LALELRTTVILGAIAIGLLTFTAYFHLPLILADGSSLSVPRMFEFGFWLAIVIGILFLGL
YSRRVAIEIRSMSDALLATQMALDREQKLTDLGGVVAAAAHELGTPLATIKLVSSELAEE
LSEQPALRDDAELIREQADRCRDILRSMGRAGKDDLQMRQAPLGEVLREAAEPHVGRGKR
VEFDLYPSRGGDERQPVILRRPEVIHGLRNLIQNAVDFARSTVWIDGEWTGDRIAIRIVD
DGEGYPPAIIGRIGDPFVRQRRAEESQSRRPGYEGMGLGLFIAKTLLERSGAELSFANAA
DPFLRSHERPERCGAIVEVIWPVDRLVVVRNAPLGENVLIQT
>P23597
MTGMDITTQDELNEAAMRDRASRDEERALRLGWWLVLAGFGGFLLWALLAPLDKGVAVQG
NVVVSGNRKVIQHMQGGIVDRIQVKDGDRVAAGQVLLTLNAVDARTTSEGLGSQYDQLIA
REARLLAEQRNQSSLAATPRLTQARQRPEMAAIIALQEDLLRSRQQSLKLEIDGVRASID
GLETSLGALQKVMSSKQSEQATLSQQLQGLRPLAADNYVPRNKMLETERLFAQVSGELAQ
TSGEVGRTRRDIQQQKLRIAQRQQEYDKEVNSELSDVQAKLNEVISQREKADFNLANVQV
RAPVAGTVVDMKIFTEGGVIAPGQVMMDIVPEDQPLLVDGRIPVEMVDKVWSGLPVELQF
TAFSQSTTPRVPGTVTLLSADRLVDEKDGTPYYGLRIQVSEEGKRSLHGLEIKPGMPVQG
FVRTGERSFINYLFKPLMDRMHLALTEE
>P31125
MSRKDGVLALLVVVVWGLNFVVIKVGLHNMPPLMLAGLRFMLVAFPAIFFVARPKVPLNL

LLGYGLTISFAQFAFLFCAINFGMPAGLASLVLQAQAFFTIMLGAFTFGERLHGKQLAGI
ALAIFGVLVLIEDSLNGQHVAMLGFMLTLAAAFSWACGNIFNKKIMSHSTRPAVMSLVIW
SALIPIIPFFVASLILDGSATMIHSLVTIDMTTILSLMYLAFVATIVGYGIWGTLLGRYE
TWRVAPLSLLVPVVGLASAALLLDERLTGLQFLGAVLIMTGLYINVFGLRWRKAVKVGS
>Q08120
MFQSFFPKPKLFFISSAVWSLLAVLAWYAGGRDIGAYLGLPPLPPGQEPVIGVSVFWSTP
FLWFYIYYAVVAGLFAAFWFAYSPHRWQYWSVLGTALIIFNTYFSVQVSVAINAWYGPFY
DLIQQALARTAPVTAGQLYSGMIGFSGIAFVAVTVGVLNLFFVSHYIFRWRTAMNEFYVA
HWPRLRHVEGASQRVQEDTMRFSSTVERLGVGLVSSIMTLIAFLPVLFKFSEQVNVLPIV
GEIPHALVWAAVFWSVFGTVFLAAVGIKLPGLEFRNQRVEAAYRKELVYGEDHEDRADPI
TLAQLFDNVRRNYFRLYFHYMYFNIARIFYLQADNLFGTFVLVPAIVAGKLTLGVMNQVL
NVFGQVRESFQYLVNSWTTIVELLSIYKRLKAFESVLVDEPLPEIDRQFIDAGGKEELAL

>P0AB67
MSGGLVTAAYIVAAILFIFSLAGLSKHETSRQGNNFGIAGMAIALIATIFGPDTGNVGWI
LLAMVIGGAIGIRLAKKVEMTEMPELVAILHSFVGLAAVLVGFNSYLHHDAGMAPILVNI
HLTEVFLGIFIGAVTFTGSVVAFGKLCGKISSKPLMLPNRHKMNLAALVVSFLLLIVFVR
TDSVGLQVLALLIMTAIALVFGWHLVASIGGADMPVVVSMLNSYSGWAAAAAGFMLSNDL
LIVTGALVGSSGAILSYIMCKAMNRSFISVIAGGFGTDGSSTGDDQEVGEHREITAEETA
ELLKNSHSVIITPGYGMAVAQAQYPVAEITEKLRARGINVRFGIHPVAGRLPGHMNVLLA
EAKVPYDIVLEMDEINDDFADTDTVLVIGANDTVNPAAQDDPKSPIAGMPVLEVWKAQNV
IVFKRSMNTGYAGVQNPLFFKENTHMLFGDAKASVDAILKAL
>P08306
MMAIATKRRGVAAVMSLGVATMTAVPALAQDVLGDLPVIGKPVNGGMNFQPASSPLAHDQ
QWLDHFVLYIITAVTIFVCLLLLICIVRFNRRANPVPARFTHNTPIEVIWTLVPVLILVA
IGAFSLPILFRSQEMPNDPDLVIKAIGHQWYWSYEYPNDGVAFDALMLEKEALADAGYSE
DEYLLATDNPVVVPVGKKVLVQVTATDVIHAWTIPAFAVKQDAVPGRIAQLWFSVDQEGV
YFGQCSELCGINHAYMPIVVKAVSQEKYEAWLAGAKEEFAADASDYLPASPVKLASAE
>P24077
MNKQSWLLNLSLLKTHPAFRAVFLARFISIVSLGLLGVAVPVQIQMMTHSTWQVGLSVTL
TGGAMFVGLMVGGVLADRYERKKVILLARGTCGIGFIGLCLNALLPEPSLLAIYLLGLWD
GFFASLGVTALLAATPALVGRENLMQAGAITMLTVRLGSVISPMIGGLLLATGGVAWNYG
LAAAGTFITLLPLLSLPALPPPPQPREHPLKSLLAGFRFLLASPLVGGIALLGGLLTMAS
AVRVLYPALADNWQMSAAQIGFLYAAIPLGAAIGALTSGKLAHSARPGLLMLLSTLGSFL
AIGLFGLMPMWILGVVCLALFGWLSAVSSLLQYTMLQTQTPEAMLGRINGLWTAQNVTGD
AIGAALLGGLGAMMTPVASASASGFGLLIIGVLLLLVLVELRHFRQTPPQVTASDS
>P54745
MVLFYRAHWRDYKNDQVRIMMNLTTLTHRDALCLNARFTSREEAIHALTQRLAALGKISS
TEQFLEEVYRRESLGPTALGEGLAVPHGKTAAVKEAAFAVATLSEPLQWEGVDGPEAVDL
VVLLAIPPNEAGTTHMQLLTALTTRLADDEIRARIQSATTPDELLSALDDKGGTQPSASF
SNAPTIVCVTACPAGIAHTYMAAEYLEKAGRKLGVNVYVEKQGANGIEGRLTADQLNSAT
ACIFAAEVAIKESERFNGIPALSVPVAEPIRHAEALIQQALTLKRSDETRTVQQDTQPVK
SVKTELKQALLSGISFAVPLIVAGGTVLAVAVLLSQIFGLQDLFNEENSWLWMYRKLGGG
LLGILMVPVLAAYTAYSLADKPALAPGFAAGLAANMIGSGFLGAVVGGLIAGYLMRWVKN

HLRLSSKFNGFLTFYLYPVLGTLGAGSLMLFVVGEPVAWINNSLTAWLNGLSGSNALLLG
AILGFMCSFDLGGPVNKAAYAFCLGAMANGVYGPYAIFASVKMVSAFTVTASTMLAPRLF
KEFEIETGKSTWLLGLAGITEGAIPMAIEDPLRVIGSFVLGSMVTGAIVGAMNIGLSTPG
AGIFSLFLLHDNGAGGVMAAIGWFGAALVGAAISTAILLMWRRHAVKHGNYLTDGVMP
>P38101
MTPTLLSAFWTYTLITAMTPGPNNILALSSATSHGFRQSTRVLAGMSLGFLIVMLLCAGI
SFSLAVIDPAAVHLLSWAGAAYIVWLAWKIATSPTKEDGLQAKPISFWASFALQFVNVKI
ILYGVTALSTFVLPQTQALSWVVGVSVLLAMIGTFGNVCWALAGHLFQRLFRQYGRQLNI
VLALLLVYCAVRIFY
>P18006
MSSTQKPADVTAERRSHWWWTVPGCLAMVLLNAAVSYGIVRLNAPVTVAFNMKQTVDAFF
DSASQKQLSEAQSKALSARFNTALEASLQAWQQKHHAVILVSPAVVQGAPDISREIQQDI
ARRMRAEP
>P0AFJ7
MLHLFAGLDLHTGLLLLLALAFVLFYEAINGFHDTANAVATVIYTRAMRSQLAVVMAAVF
NFLGVLLGGLSVAYAIVHMLPTDLLLNMGSSHGLAMVFSMLLAAIIWNLGTWYFGLPASS
SHTLIGAIIGIGLTNALMTGTSVVDALNIPKVLSIFGSLIVSPIVGLVFAGGLIFLLRRY
WSGTKKRARIHLTPAEREKKDGKKKPPFWTRIALILSAIGVAFSHGANDGQKGIGLVMLV
LIGVAPAGFVVNMNATGYEITRTRDAINNVEAYFEQHPALLKQATGADQLVPAPEAGATQ
PAEFHCHPSNTINALNRLKGMLTTDVESYDKLSLDQRSQMRRIMLCVSDTIDKVVKMPGV
SADDQRLLKKLKSDMLSTIEYAPVWIIMAVALALGIGTMIGWRRVATTIGEKIGKKGMTY
AQGMSAQMTAAVSIGLASYTGMPVSTTHVLSSSVAGTMVVDGGGLQRKTVTSILMAWVFT
LPAAVLLSGGLYWLSLQFL
>P26185
MPLKKMFLNRRISVPFVLSVGLHSALVAGLLYASVKEVVELPKPEDAPISVMMVNTAAMA
EPPPPAPAEPEPPQVEPEPEPEPEPIVEPPPKAIVKPEPVKPKPKPKPKPKVEKQVKPEP
KKVEPREPSPFNNDSPAKPIDKAPVKQAPAAPVQGNSREVGPRPISRANPLYPPRAQALQ
IEGNVRVQFDIDSDGRVSNVRILSAEPRNMFEREVKQAMRKWRYEAKEAKDRTVTIRFKL
NGTTELN
>P0AD27
MVTHRQRYREKVSQMVSWGHWFALFNILLSLVIGSRYLFIADWPTTLAGRIYSYVSIIGH
FSFLVFATYLLILFPLTFIVGSQRLMRFLSVILATAGMTLLLIDSEVFTRFHLHLNPIVW
QLVINPDENEMARDWQLMFISVPVILLLELVFATWSWQKLRSLTRRRRFARPLAAFLFIA
FIASHVVYIWADANFYRPITMQRANLPLSYPMTARRFLEKHGLLDAQEYQRRLIEQGNPD
AVSVQYPLSELRYRDMGTGQNVLLITVDGLNYSRFEKQMPALAGFAEQNISFTRHMSSGN
TTDNGIFGLFYGISPSYMDGILSTRTPAALITALNQQGYQLGLFSSDGFTSPLYRQALLS
DFSMPSVRTQSDEQTATQWINWLGRYAQEDNRWFSWVSFNGTNIDDSNQQAFARKYSRAA
GNVDDQINRVLNALRDSGKLDNTVVIITAGRGIPLSEEEETFDWSHGHLQVPLVIHWPGT
PAQRINALTDHTDLMTTLMQRLLHVSTPASEYSQGQDLFNPQRRHYWVTAADNDTLAITT
PKKTLVLNNNGKYRTYNLRGERVKDEKPQLSLLLQVLTDEKRFIAN
>P46136
MTRQKATLIGLIAIVLWSTMVGLIRGVSEGLGPVGGAAAIYSLSGLLLIFTVGFPRIRQI
PKGYLLAGSLLFVSYEICLALSLGYAATHHQAIEVGMVNYLWPSLTILFAILFNGQKTNW
LIVPGLLLALVGVCWVLGGDNGLHYDEIINNITTSPLSYFLAFIGAFIWAAYCTVTNKYA

RGFNGITVFVLLTGASLWVYYFLTPQPEMIFSTPVMIKLISAAFTLGFAYAAWNVGILHG
NVTIMAVGSYFTPVLSSALAAVLLSAPLSFSFWQGALMVCGGSLLCWLATRRG
>P56621
MADVEEWLTHARKVTQEASIGVDVTSIQECISAEPAQRVLVARRDAWRAICCAAFAALVA
FAAINRVATIMLEKPAPTWVATPSAASPFGLLIGK
>P39277
MSGLKQELGLAQGIGLLSTSLLGTGVFAVPALAALVAGNNSLWAWPVLIILVFPIAIVFA
ILGRHYPSAGGVAHFVGMAFGSRLERVTGWLFLSVIPVGLPAALQIAAGFGQAMFGWHSW
QLLLAELGTLALVWYIGTRGASSSANLQTVIAGLIVALIVAIWWAGDIKPANIPFPAPGN
IELTGLFAALSVMFWCFVGLEAFAHLASEFKNPERDFPRALMIGLLLAGLVYWGCTVVVL
HFDAYGEKMAAAASLPKIVVQLFGVGALWIACVIGYLACFASLNIYIQSFARLVWSQAQH
NPDHYLARLSSRHIPNNALNAVLGCCVVSTLVIHALEINLDALIIYANGIFIMIYLLCML
AGCKLLQGRYRLLAVVGGLLCVLLLAMVGWKSLYALIMLAGLWLLLPKRKTPENGITT
>P68699
MENLNMDLLYMAAAVMMGLAAIGAAIGIGILGGKFLEGAARQPDLIPLLRTQFFIVMGLV
DAIPMIAVGLGLYVMFAVA
>P60955
MTSSYLHFPEFDPVIFSIGPVALHWYGLMYLVGFIFAMWLATRRANRPGSGWTKNEVENL
LYAGFLGVFLGGRIGYVLFYNFPQFMADPLYLFRVWDGGMSFHGGLIGVIVVMIIFARRT
KRSFFQVSDFIAPLIPFGLGAGRLGNFINGELWGRVDPNFPFAMLFPGSRTEDILLLQTN
PQWQSIFDTYGVLPRHPSQLYELLLEGVVLFIILNLYIRKPRPMGAVSGLFLIGYGAFRI
IVEFFRQPDAQFTGAWVQYISMGQILSIPMIVAGVIMMVWAYRRSPQQHVS
>P26273
MAKFYKIWLIFDPRRVFVAQGVFLFLLAAMIHLVVLSSGLNWFEAAAAVGGQ
>P46141
MRNSHNITLTNNDSLTEDEETTWSLPGAVVGFISWLFALAMPMLIYGSNTLFFFIYTWPF
FLALMPVAVVVGIALHSLMDGKLRYSIVFTLVTVGIMFGALFMWLLG
>P29914
MLRRLSPIQPDSFEFTPANLEWARAQMTKYPEGRQQSAIIPVLWRAQEQEGWLSRPAIEY
CADLLGMPYIRALEVATFYFMFQLQPVGSVAHIQICGTTTCMICGAEDLIRVCKEKIAPE
PHALSADGRFSWEEVECLGACTNAPMAQIGKDFYEDLTVEKLAALIDRFAAGEVPVPGPQ
NGRFSAEALGGPTALADLKGGEAHNASVARALRLGDSIKRIDGTEVPITTPWLATQNGV
>P0AEH8
MTTQVRKNVMDMFIDGARRGFTIATTNLLPNVVMAFVIIQALKITGLLDWVGHICEPVMA
LWGLPGEAATVLLAALMSMGGAVGVAASLATAGALTGHDVTVLLPAMYLMGNPVQNVGRC
LGTAEVNAKYYPHIITVCVINALLSIWVMQLIV
>P0ADY1
MMDSLRTAANSLVLKIIFGIIIVSFILTGVSGYLIGGGNNYAAKVNDQEISRGQFENAFN
SERNRMQQQLGDQYSELAANEGYMKTLRQQVLNRLIDEALLDQYARELKLGISDEQVKQA
IFATPAFQVDGKFDNSRYNGILNQMGMTADQYAQALRNQLTTQQLINGVAGTDFMLKGET
DELAALVAQQRVVREATIDVNALAAKQPVTEQEIASYYEQNKNNFMTPEQFRVSYIKLDA
ATMQQPVSDADIQSYYDQHQDQFTQPQRTRYSIIQTKTEDEAKAVLDELNKGGDFAALAK
EKSADIISARNGGDMGWLEDATIPDELKNAGLKEKGQLSGVIKSSVGFLIVRLDDIQPAK
VKSLDEVRDDIAAKVKHEKALDAYYALQQKVSDAASNDTESLAGAEQAAGVKATQTGWFS

KDNLPEELNFKPVADAIFNGGLVGENGAPGINSDIITVDGDRAFVLRISEHKPEAVKPLA
DVQEQVKALVQHNKAEQQAKVDAEKLLVDLKAGKGAEAMQAAGLKFGEPKTLSRSGRDPI
SQAAFALPLPAKDKPSYGMATDMQGNVVLLALDEVKQGSMPEDQKKAMVQGITQNNAQIV
FEALMSNLRKEAKIKIGDALEQQ
>P16683
MQTITIAPPKRSWFSLLSWAVVLAVLVVSWQGAEMAPLTLIKDGGNMATFAADFFPPDFS
QWQDYLTEMAVTLQIAVWGTALAVVLSIPFGLMSAENLVPWWVYQPVRRLMDACRAINEM
VFAMLFVVAVGLGPFAGVLALFIHTTGVLSKLLSEAVEAIEPGPVEGIRATGANKLEEIL
YGVLPQVMPLLISYSLYRFESNVRSATVVGMVGAGGIGVTLWEAIRGFQFQQTCALMVLI
IVTVSLLDFLSQRLRKHFI
>P11696
TDIRTGLTDEECQEIHEMNMLGMHAYWSIGLIANALAYAWRPFHQGRAGNRLEDHAPDYV
RSALT
>P04683
MDRRVVITGLGGLCGLGTDASSIWTEMREGRSAIGPISNSEIHELKGMIGTEIKVLPQHD
IDRKQLISMDRFSLLAVLAAKQAMLQAGLSCNEGNAHRFGATVGVGFGGWDATEKAYRTL
LLGGATRTELFTGVKAMPSAAACQVSMNLGLRGPVFGATSACASANHAIASAVDQIKLGR
ADVMLAGGSDAPLVWIVLKAWEAMRVLAPDTCRPFSADRKGLVLGEGAGMAVLESYEHAA
ARGATMLAEVAGIGLSADAYHIAAPAVHGPEAAMRACLVDASLNAEDVDYLNAHGTGTKA
NDQIETTAIKRVFGDHARSMSISSTKSTHAHCLGAASALEMIACVMAIQEGVVPPTANYR
EPDPDCDLDVTPNVPRERKVRVAMSNAFAMGGMNAVLAFKQVP
>P46481
MGIFSIANQHIRFAVKLATAIVLALFVGFHFQLETPRWAVLTAAIVAAGTAFAAGGEPYS
GAIRYRGFLRIIGTFIGCIAGLVIIIAMIRAPLLMILVCCIWAGFCTWISSLVRIENSYA
WGLAGYTALIIVITIQPEPLLTPQFAVERCSEIVIGIVCAIMADLLFSPRSIKQEVDREL
ESLLVAQYQLMQLCIKHGDGEVVDKAWGDLVRRTTALQGMRSNLNMESSRWARANRRLKA
INTLSLTLITQSCETYLIQNTRPELITDTFREFFDTPVETAQDVHKQLKRLRRVIAWTGE
RETPVTIYSWVAAATRYQLLKRGVISNTKINATEEEILQGEPEVKVESAERHHAMVNFWR
TTLSCILGTLFWLWTGWTSGSGAMVMIAVVTSLAMRLPNPRMVAIDFIYGTLAALPLGLL
YFLVIIPNTQQSMLLLCISLAVLGFFLGIEVQKRRLGSMGALASTINIIVLDNPMTFHFS
QFLDSALGQIVGCVLAFTVILLVRDKSRDRTGRVLLNQFVSAAVSAMTTNVARRKENHLP
ALYQQLFLLMNKFPGDLPKFRLALTMIIAHQRLRDAPIPVNEDLSAFHRQMRRTADHVIS
ARSDDKRRRYFGQLLEELEIYQEKLRIWQAPPQVTEPVNRLAGMLHKYQHALTDS
>P97253
MSNPKDDYKIWLVINPSTWLPVIWIVATVVAIAVHAAVLAAPGFNWIALGAAKSAAK
>P0AFA7
MEISWGRALWRNFLGQSPDWYKLALIIFLIVNPLIFLISPFVAGWLLVAEFIFTLAMALK
CYPLLPGGLLAIEAVFIGMTSAEHVREEVAANLEVLLLLMFMVAGIYFMKQLLLFIFTRL
LLSIRSKMLLSLSFCVAAAFLSAFLDALTVVAVVISVAVGFYGIYHRVASSRTEDTDLQD
DSHIDKHYKVVLEQFRGFLRSLMMHAGVGTALGGVMTMVGEPQNLIIAKAAGWHFGDFFL
RMSPVTVPVLICGLLTCLLVEKLRWFGYGETLPEKVREVLQQFDDQSRHQRTRQDKIRLI
VQAIIGVWLVTALALHLAEVGLIGLSVIILATSLTGVTDEHAIGKAFTESLPFTALLTVF
FSVVAVIIDQQLFSPIIQFVLQASEHAQLSLFYIFNGLLSSISDNVFVGTIYINEAKAAM
ESGAITLKQYELLAVAINTGTNLPSVATPNGQAAFLFLLTSALAPLIRLSYGRMVWMALP

YTLVLTLVGLLCVEFTLAPVTEWFMQMGWIATL
>P18200
MTILPRHKDVAKSRLKMSNPWHLLAVGFGSGLSPIVPGTMGSLAAIPFWYLMTFLPWQLY
SLVVMLGICIGVYLCHQTAKDMGVHDHGSIVWDEFIGMWITLMALPTNDWQWVAAGFVIF
RILDMWKPWPIRWFDRNVHGGMGIMIDDIVAGVISAGILYFIGHHWPLGILS
>P16429
MSAISLINSGVAWFVAAAVLAFLFSFQKALSGWIAGIGGAVGSLYTAAAGFTVLTGAVGV
SGALSLVSYDVQISPLNAIWLITLGLCGLFVSLYNIDWHRHAQVKCNGLQINMLMAAAVC
AVIASNLGMFVVMAEIMALCAVFLTSNSKEGKLWFALGRLGTLLLAIACWLLWQRYGTLD
LRLLDMRMQQLPLGSDIWLLGVIGFGLLAGIIPLHGWVPQAHANASAPAAALFSTVVMKI
GLLGILTLSLLGGNAPLWWGIALLVLGMITAFVGGLYALVEHNIQRLLAYHTLENIGIIL
LGLGAGVTGIALEQPALIALGLVGGLYHLLNHSLFKSVLFLGAGSVWFRTGHRDIEKLGG
IGKKMPVISIAMLVGLMAMAALPPLNGFAGEWVIYQSFFKLSNSGAFVARLLGPLLAVGL
AITGALAVMCMAKVYGVTFLGAPRTKEAENATCAPLLMSVSVVALAICCVIGGVAAPWLL
PMLSAAVPLPLEPANTTVSQPMITLLLIACPLLPFIIMAICKGDRLPSRSRGAAWVCGYD
HEKSMVITAHGFAMPVKQAFAPVLKLRKWLNPVSLVPGWQCEGSALLFRRMALVELAVLV
VIIVSRGA
>P05055
MLNPIVRKFQYGQHTVTLETGMMARQATAAVMVSMDDTAVFVTVVGQKKAKPGQDFFPLT
VNYQERTYAAGRIPGSFFRREGRPSEGETLIARLIDRPIRPLFPEGFVNEVQVIATVVSV
NPQVNPDIVAMIGASAALSLSGIPFNGPIGAARVGYINDQYVLNPTQDELKESKLDLVVA
GTEAAVLMVESEAQLLSEDQMLGAVVFGHEQQQVVIQNINELVKEAGKPRWDWQPEPVNE
ALNARVAALAEARLSDAYRITDKQERYAQVDVIKSETIATLLAEDETLDENELGEILHAI
EKNVVRSRVLAGEPRIDGREKDMIRGLDVRTGVLPRTHGSALFTRGETQALVTATLGTAR
DAQVLDELMGERTDTFLFHYNFPPYSVGETGMVGSPKRREIGHGRLAKRGVLAVMPDMDK
FPYTVRVVSEITESNGSSSMASVCGASLALMDAGVPIKAAVAGIAMGLVKEGDNYVVLSD
ILGDEDHLGDMDFKVAGSRDGISALQMDIKIEGITKEIMQVALNQAKGARLHILGVMEQA
INAPRGDISEFAPRIHTIKINPDKIKDVIGKGGSVIRALTEETGTTIEIEDDGTVKIAAT
DGEKAKHAIRRIEEITAEIEVGRVYTGKVTRIVDFGAFVAIGGGKEGLVHISQIADKRVE
KVTDYLQMGQEVPVKVLEVDRQGRIRLSIKEATEQSQPAAAPEAPAAEQGE
>P75870
MLSPLLKRYTWNSAWLYYARIFIALCGTTAFPWWLGDVKLTIPLTLGMVAAALTDLDDRL
AGRLRNLIITLFCFFIASASVELLFPWPWLFAIGLTLSTSGFILLGGLGQRYATIAFGAL
LIAIYTMLGTSLYEHWYQQPMYLLAGAVWYNVLTLIGHLLFPVRPLQDNLARCYEQLARY
LELKSRMFDPDIEDQSQAPLYDLALANGLLMATLNQTKLSLLTRLRGDRGQRGTRRTLHY
YFVAQDIHERASSSHIQYQTLREHFRHSDVLFRFQRLMSMQGQACQQLSRCILLRQPYQH
DPHFERAFTHIDAALERMRDNGAPADLLKTLGFLLNNLRAIDAQLATIESEQAQALPHNN
DENELADDSPHGLSDIWLRLSRHFTPESALFRHAVRMSLVLCFGYAIIQITGMHHGYWIL
LTSLFVCQPNYNATRHRLKLRIIGTLVGIAIGIPVLWFVPSLEGQLVLLVITGVLFFAFR
NVQYAHATMFITLLVLLCFNLLGEGFEVALPRVIDTLIGCAIAWAAVSYIWPDWQFRNLP
RMLERATEANCRYLDAILEQYHQGRDNRLAYRIARRDAHNRDAELASVVSNMSSEPNVTP
QIREAAFRLLCLNHTFTSYISALGAHREQLTNPEILAFLDDAVCYVDDALHHQPADEERV
NEALASLKQRMQQLEPRADSKEPLVVQQVGLLIALLPEIGRLQRQITQVPQETPVSA
>P77757

MFEIHPVKKVSVVIPVYNEQESLPELIRRTTTACESLGKEYEILLIDDGSSDNSAHMLVE
ASQAENSHIVSILLNRNYGQHSAIMAGFSHVTGDLIITLDADLQNPPEEIPRLVAKADEG
YDVVGTVRQNRQDSWFRKTASKMINRLIQRTTGKAMGDYGCMLRAYRRHIVDAMLHCHER
STFIPILANIFARRAIEIPVHHAEREFGESKYSFMRLINLMYDLVTCLTTTPLRMLSLLG
SIIAIGGFSIAVLLVILRLTFGPQWAAEGVFMLFAVLFTFIGAQFIGMGLLGEYIGRIYT
DVRARPRYFVQQVIRPSSKENE
>P44607
MEFIGKIIGVFLGWKVGGFFGAIAGLILGSIADKKLYELGSVSSSFFKKKTTRQDLFMQT
SFAVLGHLSKSKGRVTEEDIQLANQLMIQLKLDDAGRKLAQDAFRRGKESDFPIRQVIRE
FRIGCGQRADLLRMFLQVQVQAAFADSELHENEKEVLYVIAEELGLSRMQFEQMIAMEMA
ARAFTQGGFYQKYQQGAYQGGYQYQQQNSGGYQHASGPTLNDAYKVLGVTESDEQSTVKR
AYRRLMNEHHPDKLVAKGLPPEMMEMAKEKTQQIQAAYDLICKAKGWK
>P77179
MSEIKDVIVQGLWKNNSALVQLLGLCPLLAVTSTATNALGLGLATTLVLTLTNLTISTLR
HWTPAEIRIPIYVMIIASVVSAVQMLINAYAFGLYQSLGIFIPLIVTNCIVVGRAEAFAA
KKGPALSALDGFSIGMGATCAMFVLGSLREIIGNGTLFDGADALLGSWAKVLRVEIFHTD
SPFLLAMLPPGAFIGLGLMLAGKYLIDERMKKRRAEAAAERALPNGETGNV
>P0AGI4
MSKSNPSEVKLAVPTSGGFSGLKSLNLQVFVMIAAIIAIMLFFTWTTDGAYLSARNVSNL
LRQTAITGILAVGMVFVIISAEIDLSVGSMMGLLGGVAAICDVWLGWPLPLTIIVTLVLG
LLLGAWNGWWVAYRKVPSFIVTLAGMLAFRGILIGITNGTTVSPTSAAMSQIGQSYLPAS
TGFIIGALGLMAFVGWQWRGRMRRQALGLQSPASTAVVGRQALTAIIVLGAIWLLNDYRG
VPTPVLLLTLLLLGGMFMATRTAFGRRIYAIGGNLEAARLSGINVERTKLAVFAINGLMV
AIAGLILSSRLGAGSPSAGNIAELDAIAACVIGGTSLAGGVGSVAGAVMGAFIMASLDNG
MSMMDVPTFWQYIVKGAILLLAVWMDSATKRRS
>P0A7B1
MGQEKLYIEKELSWLSFNERVLQEAADKSNPLIERMRFLGIYSNNLDEFYKVRFAELKRR
IIISEEQGSNSHSRHLLGKIQSRVLKADQEFDGLYNELLLEMARNQIFLINERQLSVNQQ
NWLRHYFKQYLRQHITPILINPDTDLVQFLKDDYTYLAVEIIRGDTIRYALLEIPSDKVP
RFVNLPPEAPRRRKPMILLDNILRYCLDDIFKGFFDYDALNAYSMKMTRDAEYDLVHEME
ASLMELMSSSLKQRLTAEPVRFVYQRDMPNALVEVLREKLTISRYDSIVPGGRYHNFKDF
INFPNVGKANLVNKPLPRLRHIWFDKAQFRNGFDAIRERDVLLYYPYHTFEHVLELLRQA
SFDPSVLAIKINIYRVAKDSRIIDSMIHAAHNGKKVTVVVELQARFDEEANIHWAKRLTE
AGVHVIFSAPGLKIHAKLFLISRKENGEVVRYAHIGTGNFNEKTARLYTDYSLLTADARI
TNEVRRVFNFIENPYRPVTFDYLMVSPQNSRRLLYEMVDREIANAQQGLPSGITLKLNNL
VDKGLVDRLYAASSSGVPVNLLVRGMCSLIPNLEGISDNIRAISIVDRYLEHDRVYIFEN
GGDKKVYLSSADWMTRNIDYRIEVATPLLDPRLKQRVLDIIDILFSDTVKARYIDKELSN
RYVPRGNRRKVRAQLAIYDYIKSLEQPE
>Q06401
MAEQEYDLIVVGSGAGACWAPIRAQEQGLKTLVVEKTELFGGTSALSGGGIWIPLNYDQK
TAGIKDDLETAFGYMKRCVRGMATDDRVLAYVETASKMAEYLRQIGIPYRAMAKYADYYP
HIEGSRPGGRTMDPVDFNAARLRVTALETMRPGPPGNQLFGRMSISAFEAHSMLSRELKS
RFTILGIMLKYFLDYPWRNKTRRDRRMTGGQALVAGLLTAANKARVEMWCNSPLKELVQD
ASGRVTGVIVERNGQRQQINARRGVLLGAGGFERNQEMRDQYLNKPTRLVDGNPCGRQYG

DAHRAGQAWAHTGADGLVLGRAHHGCSQGAGLSRHFRGTLAAGVHGGQRQGAALPQRVRP
VSGIPAAMLAENAKGNGGVPAWIVFDASFRAQNPMGPLMPGSAVPDSKVRKSWLNNVYWK
GRRWKIWRADRRGRAGLQVSARRMTEYARAGKDLDFDRGGNVFDRYYGDPRLKNPNLGPI
EKGPFYAMRLWPGEIGTKGGLLTDREGRVLDTQGRIIEGLYCVGNNSASVMAPAYAGAGS
TLGPAMTFAFRAVADMVGKPLPLENPHLLGKTV
>P06136
MSQAALNTRNSEEEVSSRRNNGTRLAGILFLLTVLTTVLVSGWVVLGWMEDAQRLPLSKL
VLTGERHYTRNDDIRQSILALGEPGTFMTQDVNIIQTQIEQRLPWIKQVSVRKQWPDELK
IHLVEYVPIARWNDQHMVDAEGNTFSVPPERTSKQVLPMLYGPEGSANEVLQGYREMGQM
LAKDRFTLKEAAMTARRSWQLTLNNDIKLNLGRGDTMKRLARFVELYPVLQQQAQTDGKR
ISYVDLRYDSGAAVGWAPLPPEESTQQQNQAQAEQQ
>Q05613
MTKTRHNLARYSGSLALVLGVHAVAVLLTLNWSVPQAIELPPAAMMVELAPLPEPAPPPP
PKAAPQPPAPVEELPLPKLVEAPKPKIAIAKPPKPKAKPQPPKPEKKPEPPKEAPPTEEV
VDAPPSNTPPQKSAAPAPSIASNSNALPTWQSDPVRHLAKYKRYPEDARRRGLQGINRLR
FVVDAEGKVVSYAMAGGSGSAALDRATLEMIRRAGTVPKPPPELLNNGTIEVVAPFVYSL
DRR
>P76538
MKSTEFHPVHYDAHGRLRLPLLFWLVLLLQARTWVLFVIAGASREQGTALLNLFYPDHDN
FWLGLIPGIPAVLAFLLSGRRATFPRTWRVLYFLLLLAQVVLLCWQPWLWLNGESVSGIG
LALVVADIVALIWLLTNRRLRACFYEVKE
>P32482
MSSKNFSWRYSLAATVLLLSPFDLLASLGMDMYLPAVPFMPNALGTTASTIQLTLTTYLV
MIGAGQLLFGPLSDRLGRRPVLLGGGLAYVVASMGLALTSSAEVFLGLRILQACGASACL
VSTFATVRDIYAGREESNVIYGILGSMLAMVPAVGPLLGALVDMWLGWRAIFAFLGLGMI
AASAAAWRFWPETRVQRVAGLQWSQLLLPVKCLNFWLYTLCYAAGMGSFFVFFSIAPGLM
MGRQGVSQLGFSLLFATVAIAMVFTARFMGRVIPKWGSPSVLRMGMGCLIAGAVLLAITE
IWALQSVLGFIAPMWLVGIGVATAVSVAPNGALRGFDHVAGTVTAVYFCLGGVLLGSIGT
LIISLLPRNTAWPVVVYCLTLATVVLGLSCVSRVKGSRGQGEHDVVALQSAGSTSNPNR
>P11667
MFSYYFQGLALGAAMILPLGPQNAFVMNQGIRRQYHIMIALLCAISDLVLICAGIFGGSA
LLMQSPWLLALVTWGGVAFLLWYGFGAFKTAMSSNIELASAEVMKQGRWKIIATMLAVTW
LNPHVYLDTFVVLGSLGGQLDVEPKRWFALGTISASFLWFFGLALLAAWLAPRLRTAKAQ
RIINLVVGCVMWFIALQLARDGIAHAQALFS
>P33927
MMPEIGNGLLCLALGIALLLSVYPLWGVARGDARMMASSRLFAWLLFMSVAGAFLVLVNA
FVVNDFTVTYVASNSNTQLPVWYRVAATWGAHEGSLLLWVLLMSGWTFAVAIFSQRIPLD
IVARVLAIMGMVSVGFLLFILFTSNPFSRTLPNFPIEGRDLNPLLQDPGLIFHPPLLYMG
YVGFSVAFAFAIASLLSGRLDSTYARFTRPWTLAAWIFLTLGIVLGSAWAYYELGWGGWW
FWDPVENASFMPWLVGTALMHSLAVTEQRASFKAWTLLLAISAFSLCLLGTFLVRSGVLV
SVHAFASDPARGMFILAFMVLVIGGSLLLFAARGHKVRSRVNNALWSRESLLLANNVLLV
AAMLVVLLGTLLPLVHKQLGLGSISIGEPFFNTMFTWLMVPFALLLGVGPLVRWGRDRPR
KIRNLLIIAFISTLVLSLLLPWLFESKVVAMTVLGLAMACWIAVLAIAEAALRISRGTKT
TFSYWGMVAAHLGLAVTIVGIAFSQNYSVERDVRMKSGDSVDIHEYRFTFRDVKEVTGPN

WRGGVATIGVTRDGKPETVLYAEKRYYNTAGSMMTEAAIDGGITRDLYAALGEELENGAW
AVRLYYKPFVRWIWAGGLMMALGGLLCLFDPRYRKRVSPQKTAPEAV
>P43531
MSRTTTVDGAPASDTDKQSISQPNQFIKRGTPQFMRVTLALFSAGLATFALLYCVQPILP
VLSQEFGLTPANSSISLSISTAMLAIGLLFTGPLSDAIGRKPVMVTALLLASICTLLSTM
MTSWHGILIMRALIGLSLSGVAAVGMTYLSEEIHPSFVAFSMGLYISGNSIGGMSGRLIS
GVFTDFFNWRIALAAIGCFALASALMFWKILPESRHFRPTSLRPKTLFINFRLHWRDRGL
PLLFAEGFLLMGSFVTLFNYIGYRLMLSPWHVSQAVVGLLSLAYLTGTWSSPKAGTMTTR
YGRGPVMLFSTGVMLFGLLMTLFSSLWLIFAGMLLFSAGFFAAHSVASSWIGPRAKRAKG
QASSLYLFSYYLGSSIAGTLGGVFWHNYGWNGVGAFIALMLVIALLVGTRLHRRLHA
>P31547
MSEPMMWLLVRGVWETLAMTFVSGFFGFVIGLPVGVLLYVTRPGQIIANAKLYRTVSAIV
NIFRSIPFIILLVWMIPFTRVIVGTSIGLQAAIVPLTVGAAPFIARMVENALLEIPTGLI
EASRAMGATPMQIVRKVLLPEALPGLVNAATITLITLVGYSAMGGAVGAGGLGQIGYQYG
YIGYNATVMNTVLVLLVILVYLIQFAGDRIVRAVTRK
>P0AC78
MNLLTVSTDLISIFLFTTLFLFFARKVAKKVGLVDKPNFRKRHQGLIPLVGGISVYAGIC
FTFGIVDYYIPHASLYLACAGVLVFIGALDDRFDISVKIRATIQAAVGIVMMVFGKLYLS
SLGYIFGSWEMVLGPFGYFLTLFAVWAAINAFNMVDGIDGLLGGLSCVSFAAIGMILWFD
GQTSLAIWCFAMIAAILPYIMLNLGILGRRYKVFMGDAGSTLIGFTVIWILLETTQGKTH
PISPVTALWIIAIPLMDMVAIMYRRLRKGMSPFSPDRQHIHHLIMRAGFTSRQAFVLITL
AAALLASIGVLAEYSHFVPEWVMLVLFLLAFFLYGYCIKRAWKVARFIKRVKRRLRRNRG
GSPNLTK
>P76473
MKSVRYLIGLFAFIACYYLLPISTRLLWQPDETRYAEISREMLASGDWIVPHLLGLRYFE
KPIAGYWINSIGQWLFGANNFGVRAGVIFATLLTAALVTWFTLRLWRDKRLALLATVIYL
SLFIVYAIGTYAVLDPFIAFWLVAGMCSFWLAMQAQTWKGKSAGFLLLGITCGMGVMTKG
FLALAVPVLSVLPWVATQKRWKDLFIYGWLAVISCVLTVLPWGLAIAQREPNFWHYFFWV
EHIQRFALDDAQHRAPFWYYVPVIIAGSLPWLGLLPGALYTGWKNRKHSATVYLLSWTIM
PLLFFSVAKGKLPTYILSCFASLAMLMAHYALLAAKNNPLALRINGWINIAFGVTGIIAT
FVVSPWGPMNTPVWQTFESYKVFCAWSIFSLWAFFGWYTLTNVEKTWPFAALCPLGLALL
VGFSIPDRVMEGKHPQFFVEMTQESLQPSRYILTDSVGVAAGLAWSLQRDDIIMYRQTGE
LKYGLNYPDAKGRFVSGDEFANWLNQHRQEGIITLVLSVDRDEDINSLAIPPADAIDRQE
RLVLIQYRPK
>P45570
MPTVITHAAVPLCIGLGLGSKVIPPRLLFAGIILAMLPDADVLSFKFGVAYGNVFGHRGF
THSLVFAFVVPLLCVFIGRRWFRAGLIRCWLFLTVSLLSHSLLDSVTTGGKGVGWLWPWS
DERFFAPWQVIKVAPFALSRYTTPYGHQVIISELMWVWLPGMLLMGMLWWRRR
>P10121
MAKEKKRGFFSWLGFGQKEQTPEKETEVQNEQPVVEEIVQAQEPVKASEQAVEEQPQAHT
EAEAETFAADVVEVTEQVAESEKAQPEAEVVAQPEPVVEETPEPVAIEREELPLPEDVNA
EAVSPEEWQAEAETVEIVEAAEEEAAKEEITDEELETALAAEAAEEAVMVVPPAEEEQPV
EEIAQEQEKPTKEGFFARLKRSLLKTKENLGSGFISLFRGKKIDDDLFEELEEQLLIADV
GVETTRKIITNLTEGASRKQLRDAEALYGLLKEEMGEILAKVDEPLNVEGKAPFVILMVG

VNGVGKTTTIGKLARQFEQQGKSVMLAAGDTFRAAAVEQLQVWGQRNNIPVIAQHTGADS
ASVIFDAIQAAKARNIDVLIADTAGRLQNKSHLMEELKKIVRVMKKLDVEAPHEVMLTID
ASTGQNAVSQAKLFHEAVGLTGITLTKLDGTAKGGVIFSVADQFGIPIRYIGVGERIEDL
RPFKADDFIEALFARED
>P77172
MKLNATYIKIRDKWWGLPLFLPSLILPIFAHINTFAHISSGEVFLFYLPLALMISMMMFF
SWAALPGIALGIFVRKYAELGFYETLSLTANFIIIIILCWGGYRVFTPRRNNVSHGDTRL
ISQRIFWQIVFPATLFLILFQFAAFVGLLASRENLVGVMPFNLGTLINYQALLVGNLIGV
PLCYFIIRVVRNPFYLRSYYSQLKQQVDAKVTKKEFALWLLALGALLLLLCMPLNEKSTI
FSTNYTLSLLLPLMMWGAMRYGYKLISLLWAVVLMISIHSYQNYIPIYPGYTTQLTITSS
SYLVFSFIVNYMAVLATRQRAVVRRIQRLAYVDPVVHLPNVRALNRALRDAPWSALCYLR
IPGMEMLVKNYGIMLRIQYKQKLSHWLSPLLEPGEDVYQLSGNDLALRLNTESHQERITA
LDSHLKQFRFFWDGMPMQPQIGVSYCYVRSPVNHIYLLLGELNTVAELSIVTNAPENMQR
RGAMYLQRELKDKVAMMNRLQQALEHNHFFLMAQPITGMRGDVYHEILLRMKGENDELIS
PDSFLPVAHEFGLSSSIDMWVIEHTLQFMAENRAKMPAHRFAINLSPTSVCQARFPVEVS
QLLAKYQIEAWQLIFEVTESNALTNVKQAQITLQHLQELGCQIAIDDFGTGYASYARLKN
VNADLLKIDGSFIRNIVSNSLDYQIVASICHLARMKKMLVVAEYVENEEIREAVLSLGID
YMQGYLIGKPQPLIDTLNEIEPIRESA
>P75830
MKKRKTVKKRYVIALVIVIAGLITLWRILNAPVPTYQTLIVRPGDLQQSVLATGKLDALR
KVDVGAQVSGQLKTLSVAIGDKVKKDQLLGVIDPEQAENQIKEVEATLMELRAQRQQAEA
ELKLARVTYSRQQRLAQTKAVSQQDLDTAATEMAVKQAQIGTIDAQIKRNQASLDTAKTN
LDYTRIVAPMAGEVTQITTLQGQTVIAAQQAPNILTLADMSAMLVKAQVSEADVIHLKPG
QKAWFTVLGDPLTRYEGQIKDVLPTPEKVNDAIFYYARFEVPNPNGLLRLDMTAQVHIQL
TDVKNVLTIPLSALGDPVGDNRYKVKLLRNGETREREVTIGARNDTDVEIVKGLEAGDEV
VIGEAKPGAAQ
>P29925
MTNLLSIITFLPIVAAIIMALFLRGQDEAAARNAKWLALLTTTATFVISLFVLFRFDPAN
TGFQFVEDHAWIMGVCYKMGVDGISVLFVLLTTFMMPLTILSTWQVQDKVKEYMIAFLVL
EGLMIGVFTALDLVLFYLFFEAGLIPMFLIIGIWGGKDRIYASFKFFLYTFLGSVLMLVA
MIAMYRMAGTTDIPTLLTFDFPSENFRLLGMTVVGGMQMLLFLAFFASFAVKMPMWPVHT
WLPDAHVQAPTAGSVLLAAVLLKMGGYGFLRFSLPMFPVASGVAQPYVFWLSAIAIVYTS
LVALAQSDMKKVIAYSSVAHMGYVTMGVFAANQIGVDGAIFQMLSHGFISGALFLCVGVI
YDRMHTREIDAYGGLVNRMPAYAAVFMFFTMANVGLPGTSGFVGEFLTLMGVFRVDTWVA
LVATSGVILSAAYALWLYRRVTLGQLIKESLKSITDMTPRERWVFIPLIAMTLILGVYPR
LVTDVTGPAVAALVQDYNQSQPAAPVATAQASH
>P0AF80
MHILDSLLAFSAYFFIGVAMVIIFLFIYSKITPHNEWQLIKNNNTAASLAFSGTLLGYVI
PLSSAAINAVSIPDYFAWGGIALVIQLLVFAGVRLYMPALSEKIINHNTAAGMFMGTAAL
AGGIFNAACMTW
>P42640
MTVFNKFARTFKSHWLLYLCVIVFGITNLVASSGAHMVQRLLFFVLTILVVKRISSLPLR
LLVAAPFVLLTAADMSISLYSWCTFGTTFNDGFAISVLQSDPDEVVKMLGMYIPYLCAFA
FLSLLFLAVIIKYDVSLPTKKVTGILLLIVISGSLFSACQFAYKDAKNKKAFSPYILASR

FATYTPFFNLNYFALAAKEHQRLLSIANTVPYFQLSVRDTGIDTYVLIVGESVRVDNMSL
YGYTRSTTPQVEAQRKQIKLFNQAISGAPYTALSVPLSLTADSVLSHDIHNYPDNIINMA
NQAGFQTFWLSSQSAFRQNGTAVTSIAMRAMETVYVRGFDELLLPHLSQALQQNTQQKKL
IVLHLNGSHEPACSAYPQSSAVFQPQDDQDACYDNSIHYTDSLLGQVFELLKDRRASVMY
FADHGLERDPTKKNVYFHGGREASQQAYHVPMFIWYSPVLGDGVDRTTENNIFSTAYNNY
LINAWMGVTKPEQPQTLEEVIAHYKGDSRVVDANHDVFDYVMLRKEFTEDKQGNPTPEGQ
G
>P76445
MIKNLPQIVLLNIVGLALFLSWYIPVNHGFWLPIDADIFYFFNQKLVESKAFLWLVALTN
NRAFDGCSLLAMGMLMLSFWLKENAPGRRRIVIIGLVMLLTAVVLNQLGQALIPVKRASP
TLTFTDINRVSELLSVPTKDASRDSFPGDHGMMLLIFSAFMWRYFGKVAGLIALIIFVVF
AFPRVMIGAHWFTDIIVGSMTVILIGLPWVLLTPLSDRLITFFDKSLPGKNKHFQNK
>Q46821
MSAIDSQLPSSSGQDRPTDEVDRILSPGKLIILGLQHVLVMYAGAVAVPLMIGDRLGLSK
EAIAMLISSDLFCCGIVTLLQCIGIGRFMGIRLPVIMSVTFAAVTPMIAIGMNPDIGLLG
IFGATIAAGFITTLLAPLIGRLMPLFPPLVTGVVITSIGLSIIQVGIDWAAGGKGNPQYG
NPVYLGISFAVLIFILLITRYAKGFMSNVAVLLGIVFGFLLSWMMNEVNLSGLHDASWFA
IVTPMSFGMPIFDPVSILTMTAVLIIVFIESMGMFLALGEIVGRKLSSHDIIRGLRVDGV
GTMIGGTFNSFPHTSFSQNVGLVSVTRVHSRWVCISSGIILILFGMVPKMAVLVASIPQF
VLGGAGLVMFGMVLATGIRILSRCNYTTNRYNLYIVAISLGVGMTPTLSHDFFSKLPAVL
QPLLHSGIMLATLSAVVLNVFFNGYQHHADLVKESVSDKDLKVRTVRMWLLMRKLKKNEH
GE
>P75757
MAHSHSHTSSHLPEDNNARRLLYAFGVTAGFMLVEVVGGFLSGSLALLADAGHMLTDTAA
LLFALLAVQFSRRPPTIRHTFGWLRLTTLAAFVNAIALVVITILIVWEAIERFRTPRPVE
GGMMMAIAVAGLLANILSFWLLHHGSEEKNLNVRAAALHVLGDLLGSVGAIIAALIIIWT
GWTPADPILSILVSLLVLRSAWRLLKDSVNELLEGAPVSLDIAELKRRMCREIPEVRNVH
HVHVWMVGEKPVMTLHVQVIPPHDHDALLDQIQHYLMDHYQIEHATIQMEYQPCHGPDCH
LNEGVSGHSHHHH
>P42603
MTAYWLAQGVGVIAFLIGITTFFNRDERRFKKQLSVYSAVIGVHFFLLGTYPAGASAILN
AIRTLITLRTRSLWVMAIFIVLTGGIGLAKFHHPVELLPVIGTIVSTWALFCCKGLTMRC
VMWFSTCCWVIHNFWAGSIGGTMIEGSFLLMNGLNIIRFWRMQKRGIDPFKVEKTPSAVD
ERG
>P0A6M2
MLRFLNQCSQGRGAWLLMAFTALALELTALWFQHVMLLKPCVLCIYERCALFGVLGAALI
GAIAPKTPLRYVAMVIWLYSAFRGVQLTYEHTMLQLYPSPFATCDFMVRFPEWLPLDKWV
PQVFVASGDCAERQWDFLGLEMPQWLLGIFIAYLIVAVLVVISQPFKAKKRDLFGR
>P27843
MLRIFIPTSNGKISRRRYIFSFILINFIFAFLIIFFNDGEAGFLVIVSTIVLHYLVINMN
CQRLRDSGFIYIKTYVFGTLAVYIISIITMIAEDFACSGNGSMIFLICYFSTFSMLMLAP
TDSSKQ
>Q51392
MVFSSNVFLFLFLPVFLGLYYLSGERYRNLLLLIASYVFYAWWRVDFLLLFAGVTVFNYW

IGLRIGAAGVRTRAAQRWLILGVVVDLCVLGYFKYANFGVDSLNEIITSFGMQPFVLTHI
LLPIGISFYTFESISYIIDVYRGDTPATHNLIDFAAFVAIFPHLIAGPVLRFKDLVDQFN
HRTHTVDKFAEGCTRFMQGFVKKVFIADTLAALADHCFALQNPTTGDAWLGALAYTAQLY
FDFSGYSDMAIGLGLMMGFRFMENFNQPYISQSITEFWRRWHISLSTWLRDYLYISLGGN
RGSTFQTYRNLFLTMLLGGLWHGANFTYIIWGAWHGMWLAIERALGVNAAPRVLNPLKWV
ITFLLVVIGWVIFRAENLQVAWRMYEAMFSFGTWQLSELNRANLTGLQVGTLVLAYLVLA
FFGLRQFYNQPLQTKAPKAAANSDEVAADGPASAQPRAPREAAGDPAAIAYSPSGALVYQ
PSWLSQLPVLATRLALLLLFAASVLKLSAQSYSPFLYFQF
>P40608
MKLRTVAASLLLMLSATTVRASAADVGAPVPIYTEAELIKLIEQNKHLQRVRADNCQLVE
DIVARATRINLPAYEFLYGDMLAWGVCVEQDVELGLYYMENAAQQGLPAALEQIGRYYSR
GTLVQQDKERAIPYLREAASMGNLNARIHLAELLLRDYGSPLDYEDAYRWLYNSVTADQR
QHKRIAVLRRGLEQRMPQNIVARAKRRDMFW
>P14376
MKYLASFRTTLKASRYMFRALALVLWLLIAFSSVFYIVNALHQRESEIRQEFNLSSDQAQ
RFIQRTSDVMKELKYIAENRLSAENGVLSPRGRETQADVPAFEPLFADSDCSAMSNTWRG
SLESLAWFMRYWRDNFSAAYDLNRVFLIGSDNLCMANFGLRDMPVERDTALKALHERINK
YRNAPQDDSGSNLYWISEGPRPGVGYFYALTPVYLANRLQALLGVEQTIRMENFFLPGTL
PMGVTILDENGHTLISLTGPESKIKGDPRWMQERSWFGYTEGFRELVLKKNLPPSSLSIV
YSVPVDKVLERIRMLILNAILLNVLAGAALFTLARMYERRIFIPAESDALRLEEHEQFNR
KIVASAPVGICILRTADGVNILSNELAHTYLNMLTHEDRQRLTQIICGQQVNFVDVLTSN
NTNLQISFVHSRYRNENVAICVLVDVSSRVKMEESLQEMAQAAEQASQSKSMFLATVSHE
LRTPLYGIIGNLDLLQTKELPKGVDRLVTAMNNSSSLLLKIISDILDFSKIESEQLKIEP
REFSPREVMNHITANYLPLVVRKQLGLYCFIEPDVPVALNGDPMRLQQVISNLLSNAIKF
TDTGCIVLHVRADGDYLSIRVRDTGVGIPAKEVVRLFDPFFQVGTGVQRNFQGTGLGLAI
CEKLISMMDGDISVDSEPGMGSQFTVRIPLYGAQYPQKKGVEGLSGKRCWLAVRNASLCQ
FLETSLQRSGIVVTTYEGQEPTPEDVLITDEVVSKKWQGRAVVTFCRRHIGIPLEKAPGE
WVHSVAAPHELPALLARIYLIEMESDDPANALPSTDKAVSDNDDMMILVVDDHPINRRLL
ADQLGSLGYQCKTANDGVDALNVLSKNHIDIVLSDVNMPNMDGYRLTQRIRQLGLTLPVI
GVTANALAEEKQRCLESGMDSCLSKPVTLDVIKQTLTLYAERVRKSRDS
>P37652
MKRKLFWICAVAMGMSAFPSFMTQATPATQPLINAEPAVAAQTEQNPQVGQVMPGVQGAD
APVVAQNGPSRDVKLTFAQIAPPPGSMVLRGINPNGSIEFGMRSDEVVTKAMLNLEYTPS
PSLLPVQSQLKVYLNDELMGVLPVTKEQLGKKTLAQMPINPLFISDFNRVRLEFVGHYQD
VCEKPASTTLWLDVGRSSGLDLTYQTLNVKNDLSHFPVPFFDPSDNRTNTLPMVFAGAPD
VGLQQASAIVASWFGSRSGWRGQNFPVLYNQLPDRNAIVFATNDKRPDFLRDHPAVKAPV
IEMINHPQNPYVKLLVVFGRDDKDLLQAAKGIAQGNILFRGESVVVNEVKPLLPRKPYDA
PNWVRTDRPVTFGELKTYEEQLQSSGLEPAAINVSLNLPPDLYLMRSTGIDMDINYRYTM
PPVKDSSRMDISLNNQFLQSFNLSSKQEANRLLLRIPVLQGLLDGKTDVSIPALKLGATN
QLRFDFEYMNPMPGGSVDNCITFQPVQNHVVIGDDSTIDFSKYYHFIPMPDLRAFANAGF
PFSRMADLSQTITVMPKAPNEAQMETLLNTVGFIGAQTGFPAINLTVTDDGSTIQGKDAD
IMIIGGIPDKLKDDKQIDLLVQATESWVKTPMRQTPFPGIVPDESDRAAETRSTLTSSGA
MAAVIGFQSPYNDQRSVIALLADSPRGYEMLNDAVNDSGKRATMFGSVAVIRESGINSLR
VGDVYYVGHLPWFERVWYALANHPILLAVLAAISVILLAWVLWRLLRIISRRRLNPDNE

>P0AG38
MLMLFLTVAMVHIVALMSPGPDFFFVSQTAVSRSRKEAMMGVLGITCGVMVWAGIALLGL
HLIIEKMAWLHTLIMVGGGLYLCWMGYQMLRGALKKEAVSAPAPQVELAKSGRSFLKGLL
TNLANPKAIIYFGSVFSLFVGDNVGTTARWGIFALIIVETLAWFTVVASLFALPQMRRGY
QRLAKWIDGFAGALFAGFGIHLIISR
>P34008
MLVLGGGGGAAAFFLLKPEPAAEAGEHGEKKEEKKKEKKKEEKGDKKDAEKGAERRWDSG
DQGGPDGVVFYTLPDIVVNMQTADGKSTFLKLKLTFELPDEETADELTPNLPRLQDMFQT
FLRELRPEDLNGSQGTYQLRVELLRRVNLVAAPAKVNAVLIEEMLIN
>P46187
MIKEWATVVSWQNGQALVSCDVKASCSSCASRAGCGSRVLNKLGPQTTHTIVVPCDEPLV
PGQKVELGIAEGSLLSSALLVYMSPLVGLFLIASLFQLLFASDVAALCGAILGGIGGFLI
ARGYSRKFAARAEWQPIILSVALPPGLVRFETSSEDASQ
>P0AA93
MHEIFNMLLAVFDRAALMLICLFFLIRIRLFRELLHKSAHSPKELLAVTAIFSLFALFST
WSGVPVEGSLVNVRIIAVMSGGILFGPWVGIITGVIAGIHRYLIDIGGVTAIPCFITSIL
AGCISGWINLKIPKAQRWRVGILGGMLCETLTMILVIVWAPTTALGIDIVSKIGIPMILG
SVCIGFIVLLVQSVEGEKEASAARQAKLALDIANKTLPLFRHVNSESLRKVCEIIRDDIH
ADAVAITNTDHVLAYVGVGEHNYQNGDDFISPTTRQAMNYGKIIIKNNDEAHRTPEIHSM
LVIPLWEKGVVTGTLKIYYCHAHQITSSLQEMAVGLSQIISTQLEVSRAEQLREMANKAE
LRALQSKINPHFLFNALNAISSSIRLNPDTARQLIFNLSRYLRYNIELKDDEQIDIKKEL
YQIKDYIAIEQARFGDKLTVIYDIDEEVNCCIPSLLIQPLVENAIVHGIQPCKGKGVVTI
SVAECGNRVRIAVRDTGHGIDPKVIERVEANEMPGNKIGLLNVHHRVKLLYGEGLHIRRL
EPGTEIAFYIPNQRTPVASQATLLL
>P0AEY1
MLDLFKAIGLGLVVLLPLANPLTTVALFLGLAGNMNSAERNRQSLMASVYVFAIMMVAYY
AGQLVMDTFGISIPGLRIAGGLIVAFIGFRMLFPQQKAIDSPEAKSKSEELEDEPSANIA
FVPLAMPSTAGPGTIAMIISSASTVRQSSTFADWVLMVAPPLIFFLVAVILWGSLRSSGA
IMRLVGKGGIEAISRLMGFLLVCMGVQFIINGILEIIKTYH
>P31437
MGSTRKGMLNVLIAAVLWGSSGVCAQYIMEQSQMSSQFLTMTRLIFAGLILLTLSFVHGD
KIFSIINNHKDAISLLIFSVVGALTVQLTFLLTIEKSNAATATVLQFLSPTIIVAWFSLV
RKSRPGILVFCAILTSLVGTFLLVTHGNPTSLSISPAALFWGIASAFAAAFYTTYPSTLI
ARYGTLPVVGWSMLIGGLILLPFYARQGTNFVVNGSLILAFFYLVVIGTSLTFSLYLKGA
QLIGGPKASILSCAEPLSSALLSLLLLGITFTLPDWLGTLLILSSVILISMDSRRRARKI
NRPARHK
>P0ABA0
MNLNATILGQAIAFVLFVLFCMKYVWPPLMAAIEKRQKEIADGLASAERAHKDLDLAKAS
ATDQLKKAKAEAQVIIEQANKRRSQILDEAKAEAEQERTKIVAQAQAEIEAERKRAREEL
RKQVAILAVAGAEKIIERSVDEAANSDIVDKLVAEL
>P0AA78
MATFGACRFFFGYPVVTNIFSLWRDDGRASCGYNKTMRFYMRKIKGLRWYMIALVTLGTV
LGYLTRNTVAAAAPTLMEELNISTQQYSYIIAAYSAAYTVMQPVAGYVLDVLGTKIGYAM
FAVLWAVFCGATALAGSWGGLAVARGAVGAAEAAMIPAGLKASSEWFPAKERSIAVGYFN

VGSSIGAMIAPPLVVWAIVMHSWQMAFIISGALSFIWAMAWLIFYKHPRDQKHLTDEERD
YIINGQEAQHQVSTAKKMSVGQILRNRQFWGIALPRFLAEPAWGTFNAWIPLFMFKVYGF
NLKEIAMFAWMPMLFADLGCILGGYLPPLFQRWFGVNLIVSRKMVVTLGAVLMIGPGMIG
LFTNPYVAIMLLCIGGFAHQALSGALITLSSDVFGRNEVATANGLTGMSAWLASTLFALV
VGALADTIGFSPLFAVLAVFDLLGALVIWTVLQNKPAIEVAQETHNDPAPQH
>P0AAW5
MKWQQRVRVATGLSCWQIMLHLLVVALLVVGWMSKTLVHVGVGLCALYCVTVVMMLVFQR
HPEQRWREVADVLEELTTTWYFGAALIVLWLLSRVLENNFLLAIAGLAILAGPAVVSLLA
KDKKLHHLTSKHRVRR
>P31826
MITIPITLRMLIAKYLCLLKPFWLRKNNKTSVLLIIIILAMILGVVKIQVWLNDWNNDFF
NALSQKETDKLWQLVLWFPALLGIFVLISVNKTWLIKLLTIRWREWLTDYYLNRWFADKN
YYFTQIYGEHKNTDNPDQRIAEDILLLISKTLSLSFGFIQSLSMLITFTVILWESAGTLS
FTVGGTEWNIQGYMVYTVVLIVIGGTLFTHKVGKRIRPLNVEKQRSEATFRTNLVQHNKQ
AELIALSNAESLQRQELSDNFHTIKENWHRLMNRQRWLDYWQNIYSRSLSVLPYFLLLPQ
FISGQINLGGLMKSRQAFMLVSNNLSWFIYKYDELAELAAVIDRLYEFHQLTEQRPTNKP
KNCQHAVQVADASIRTPDNKIILENLNFHVSPGKWLLLKGYSGAGKTTLLKTLSHCWPWF
KGDISSPADSWYVSQTPLIKTGLLKEIICKALPLPVDDKSLSEVLHQVGLGKLAARIHDH
DRWGDILSSGEKQRIALARLILRRPKWIFLDETTSHLEEQEAIRLLRLVREKLPTSGVIM
VTHQPGVWNLADDICDISAVL
>P31448
MNSLQILSFVGFTLLVAVITWWKVRKTDTGSQQGYFLAGRSLKAPVIAASLMLTNLSTEQ
LVGLSGQAYKSGMSVMGWEVTSAVTLIFLALIFLPRYLKRGIATIPDFLEERYDKTTRII
IDFCFLIATGVCFLPIVLYSGALALNSLFHVGESLQISHGAAIWLLVILLGLAGILYAVI
GGLRAMAVADSINGIGLVIGGLMVPVFGLIAMGKGSFMQGIEQLTTVHAEKLNSIGGPTD
PLPIGAAFTGLILVNTFYWCTNQGIVQRTLASKSLAEGQKGALLTAVLKMLDPLVLVLPG
LIAFHLYQDLPKADMAYPTLVNNVLPVPMVGFFGAVLFGAVISTFNGFLNSASTLFSMGI
YRRIINQNAEPQQLVTVGRKFGFFIAIVSVLVAPWIANAPQGLYSWMKQLNGIYNVPLVT
IIMGFFFPRIPALAAKVAMGIGIISYITINYLVKFDFHFLYVLACTFCINVVVMLVIGF
IKPRATPFTFKDAFAVDMKPWKNVKIASIGILFAMIGVYAGLAEFGGYGTRWLAMISYFI
AAVVIVYLIFDSWRHRHDPAVTFTPDGKDSL
>P0AFK2
MDFFSVQNILVHIPIGAGGYDLSWIEAVGTIAGLLCIGLASLEKISNYFFGLINVTLFGI
IFFQIQLYASLLLQVFFFAANIYGWYAWSRQTSQNEAELKIRWLPLPKALSWLAVCVVSI
GLMTVFINPVFAFLTRVAVMIMQALGLQVVMPELQPDAFPFWDSCMMVLSIVAMILMTRK
YVENWLLWVIINVISVVIFALQGVYAMSLEYIILTFIALNGSRMWINSARERGSRALSH
>P0AFC3
MSMSTSTEVIAHHWAFAIFLIVAIGLCCLMLVGGWFLGGRARARSKNVPFESGIDSVGSA
RLRLSAKFYLVAMFFVIFDVEALYLFAWSTSIRESGWVGFVEAAIFIFVLLAGLVYLVRI
GALDWTPARSRRERMNPETNSIANRQR
>P37642
MTQENEIKRPIQDLEHEPIKPLDNSEKGSKVSQALETVTTTAEKVQRQPVIAHLIRATER
FNDRLGNQFGAAITYFSFLSMIPILMVSFAAGGFVLASHPMLLQDIFDKILQNISDPTLA
ATLKNTINTAVQQRTTVGLVGLAVALYSGINWMGNLREAIRAQSRDVWERSPQDQEKFWV

KYLRDFISLIGLLIALIVTLSITSVAGSAQQMIISALHLNSIEWLKPTWRLIGLAISIFA
NYLLFFWIFWRLPRHRPRKKALIRGTFLAAIGFEVIKIVMTYTLPSLMKSPSGAAFGSVL
GLMAFFYFFARLTLFCAAWIATAEYKDDPRMPGKTQP
>P21345
MKNIKFSLAWQILFAMVLGILLGSYLHYHSDSRDWLVVNLLSPAGDIFIHLIKMIVVPIV
ISTLVVGIAGVGDAKQLGRIGAKTIIYFEVITTVAIILGITLANVFQPGAGVDMSQLATV
DISKYQSTTEAVQSSSHGIMGTILSLVPTNIVASMAKGEMLPIIFFSVLFGLGLSSLPAT
HREPLVTVFRSISETMFKVTHMVMRYAPVGVFALIAVTVANFGFSSLWPLAKLVLLVHFA
ILFFALVVLGIVARLCGLSVWILIRILKDELILAYSTASSESVLPRIIEKMEAYGAPVSI
TSFVVPTGYSFNLDGSTLYQSIAAIFIAQLYGIDLSIWQEIILVLTLMVTSKGIAGVPGV
SFVVLLATLGSVGIPLEGLAFIAGVDRILDMARTALNVVGNALAVLVIAKWEHKFDRKKA
LAYEREVLGKFDKTADQ
>P0AF40
MKQANQDRGTLLLALVAGLSINGTFAALFSSIVPFSVFPIISLVLTVYCLHQRYLNRTMP
VGLPGLAAACFILGVLLYSTVVRAEYPDIGSNFFPAVLSVIMVFWIGAKMRNRKQEVAE
>P0ADL6
MPDSRKARRIADPGLQPERTSLAWFRTMLGYGALMALAIKHNWHQAGMLFWISIGILAIV
ALILWHYTRNRNLMDVTNSDFSQFHVVRDKFLISLAVLSLAILFAVTHIHQLIVFIERVA

>P0A8D9
MSTPDNRSVNFFSLFRRGQHYSKTWPLEKRLAPVFVENRVIKMTRYAIRFMPPIAVFTLC
WQIALGGQLGPAVATALFALSLPMQGLWWLGKRSVTPLPPAILNWFYEVRGKLQESGQVL
APVEGKPDYQALADTLKRAFKQLDKTFLDDL
>P25396
MQSDKVLNLPAGYFGIVLGTIGMGFAWRYASQVWQVSHWLGDGLVILAMIIWGLLTSAFI
ARLIRFPHSVLAEVRHPVLSSFVSLFPATTMLVAIGFVPWFRPLAVCLFSFGVVVQLAYA
AWQTAGLWRGSHPEEATTPGLYLPTVANNFISAMACGALGYTDAGLVFLGAGVFSWLSLE
PVILQRLRSSGELPTALRTSLGIQLAPALVACSAWLSVNGGEGDTLAKMLFGYGLLQLLF
MLRLMPWYLSQPFNASFWSFSFGVSALATTGLHLGSGSDNGFFHTLAVPLFIFTNFIIAI
LLIRTFALLMQGKLLVRTERAVLMKAEDKE
>P0AER0
MSQTSTLKGQCIAEFLGTGLLIFFGVGCVAALKVAGASFGQWEISVIWGLGVAMAIYLTA
GVSGAHLNPAVTIALWLFACFDKRKVIPFIVSQVAGAFCAAALVYGLYYNLFFDFEQTHH
IVRGSVESVDLAGTFSTYPNPHINFVQAFAVEMVITAILMGLILALTDDGNGVPRGPLAP
LLIGLLIAVIGASMGPLTGFAMNPARDFGPKVFAWLAGWGNVAFTGGRDIPYFLVPLFGP
IVGAIVGAFAYRKLIGRHLPCDICVVEEKETTTPSEQKASL
>P25714
MDSQRNLLVIALLFVSFMIWQAWEQDKNPQPQAQQTTQTTTTAAGSAADQGVPASGQGKL
ISVKTDVLDLTINTRGGDVEQALLPAYPKELNSTQPFQLLETSPQFIYQAQSGLTGRDGP
DNPANGPRPLYNVEKDAYVLAEGQNELQVPMTYTDAAGNTFTKTFVLKRGDYAVNVNYNV
QNAGEKPLEISSFGQLKQSITLPPHLDTGSSNFALHTFRGAAYSTPDEKYEKYKFDTIAD
NENLNISSKGGWVAMLQQYFATAWIPHNDGTNNFYTANLGNGIAAIGYKSQPVLVQPGQT
GAMNSTLWVGPEIQDKMAAVAPHLDLTVDYGWLWFISQPLFKLLKWIHSFVGNWGFSIII
ITFIVRGIMYPLTKAQYTSMAKMRMLQPKIQAMRERLGDDKQRISQEMMALYKAEKVNPL

GGCFPLLIQMPIFLALYYMLMGSVELRQAPFALWIHDLSAQDPYYILPILMGVTMFFIQK
MSPTTVTDPMQQKIMTFMPVIFTVFFLWFPSGLVLYYIVSNLVTIIQQQLIYRGLEKRGL
HSREKKKS
>P33218
MANWLNQLQSLLGQSSSSTSSSADQGLVKLLVPGALGGLAGLLVANKSARKLLTKYGTNA
LLVGGGAVAGTVLWNKYKDKIRAAHQDEPQFGAQSTPLDERTARLILALVFAAKSDGHID
AKERAAIDQQLRGAGVEEQGRVLIEQAIEQPLDPQRLATGVRNEEEALEIYFLSCAAIDI
DHFMERSYLNALGDALKIPQDVRDGIERDLEQQKRTLAE
>P09348
MLILLGYLVVLGTVFGGYLMTGGSLGALYQPAELVIIAGAGIGSFIVGNNGKAIKGTLKA
LPLLFRRSKYTKAMYMDLLALLYRLMAKSRQMGMFSLERDIENPRESEIFASYPRILADS
VMLDFIVDYLRLIISGHMNTFEIEALMDEEIETHESEAEVPANSLALVGDSLPAFGIVAA
VMGVVHALGSADRPAAELGALIAHAMVGTFLGILLAYGFISPLATVLRQKSAETSKMMQC
VKVTLLSNLNGYAPPIAVEFGRKTLYSSERPSFIELEEHVRAVKNPQQQTTTEEA
>P0C0L7
MLKRKKVKPITLRDVTIIDDGKLRKAITAASLGNAMEWFDFGVYGFVAYALGKVFFPGAD
PSVQMVAALATFSVPFLIRPLGGLFFGMLGDKYGRQKILAITIVIMSISTFCIGLIPSYD
TIGIWAPILLLICKMAQGFSVGGEYTGASIFVAEYSPDRKRGFMGSWLDFGSIAGFVLGA
GVVVLISTIVGEANFLDWGWRIPFFIALPLGIIGLYLRHALEETPAFQQHVDKLEQGDRE
GLQDGPKVSFKEIATKYWRSLLTCIGLVIATNVTYYMLLTYMPSYLSHNLHYSEDHGVLI
IIAIMIGMLFVQPVMGLLSDRFGRRPFVLLGSVALFVLAIPAFILINSNVIGLIFAGLLM
LAVILNCFTGVMASTLPAMFPTHIRYSALAAAFNISVLVAGLTPTLAAWLVESSQNLMMP
AYYLMVVAVVGLITGVTMKETANRPLKGATPAASDIQEAKEILVEHYDNIEQKIDDIDHE
IADLQAKRTRLVQQHPRIDE
>P06282
MKKAGLLFLVMIVIAVVAAGIGYWKLTGEESDTLRKIVLEECLPNQQQNQNPSPCAEVKP
NAGYVVLKDLNGPLQYLLMPTYRINGTESPLLTDPSTPNFFWLAWQARDFMSKKYGQPVP
DRAVSLAINSRTGRTQNHFHIHISCIRPDVRKQLDNNLANISSRWLPLPGGLRGHEYLAR
RVTESELVQRSPFMMLAEEVPEAREHMGRYGLAMVRQSDNSFVLLATQRNLLTLNRASAE
EIQDHQCEILR
>P29924
MEKFVLFAPLIASLIAGLGWRAIGEKAAQYLTTGVLFLSCLISWYLFLSFDGVPRHIPVL
DWVVTGDFHAEWAIRLDRLTAIMLIVVTTVSALVHMYSLGYMAHDDNWTHDEHYKARFFA
YLSFFTFAMLMLVTADNLLQMFFGWEGVGVASYLLIGFYYKKASANAAAMKAFIVNRVGD
FGFLLGIFGIYWLTGSVQFDEIFRQVPQLAQTEIDFLWRDWNAANLLGFLLFVGAMGKSA
QLLLHTWLPDAMEGPTPVSALIHAATMVTAGVFLVCRMSPLYEFAPDAKNFIVIIGATTA
FFAATVGLVQNDIKRVIAYSTCSQLGYMFVAAGVGVYSAAMFHLLTHAFFKAMLFLGAGS
VIHAMHHEQDMRNYGGLRKKIPLTFWAMMIGTFAITGVGIPLTHLGFAGFLSKDAIIESA
YAGSGYAFWLLVIAACFTSFYSWRLIFLTFYGKPRGDHHAHDHAHESPPVMTIPLGVLAI
GAVFAGMVWYGPFFGDHHKVTEYFHIAGAHHEAAEGEEAEHATAEAPVEHAVADTATAEG
EAAAEAEHAEIAAPVGGAIYMHPDNHIMDEAHHAPAWVKVSPFVAMVLGLITAWTFYIAN
PSLPRRLAAHEPALYRFLLNKWYFDEIYEFIFVRPAKWLGRVLWKGGDGAVIDGTINGVA
MGLIPRLTRAAVRVQSGYLFHYAFAMVLGIVGLLIWVMMRGAH
>P36655

MAQRIFTLILLLCSTSVFAGLFDAPGRSQFVPADQAFAFDFQQNQHDLNLTWQIKDGYYL
YRKQIRITPEHAKIADVQLPQGVWHEDEFYGKSEIYRDRLTLPVTINQASAGATLTVTYQ
GCADAGFCYPPETKTVPLSEVVANNAAPQPVSVPQQEQPTAQLPFSALWALLIGIGIAFT
PCVLPMYPLISGIVLGGKQRLSTARALLLTFIYVQGMALTYTALGLVVAAAGLQFQAALQ
HPYVLIGLAIVFTLLAMSMFGLFTLQLPSSLQTRLTLMSNRQQGGSPGGVFVMGAIAGLI
CSPCTTAPLSAILLYIAQSGNMWLGGGTLYLYALGMGLPLMLITVFGNRLLPKSGPWMEQ
VKTAFGFVILALPVFLLERVIGDVWGLRLWSALGVAFFGWAFITSLQAKRGWMRIVQIIL
LAAALVSVRPLQDWAFGATHTAQTQTHLNFTQIKTVDELNQALVEAKGKPVMLDLYADWC
VACKEFEKYTFSDPQVQKALADTVLLQANVTANDAQDVALLKHLNVLGLPTILFFDGQGQ
EHPQARVTGFMDAETFSAHLRDRQP
>P09129
MITQQIISSELEVLKKHIDSGDIRIPSLWQGLKPGLIIMGWMIFCPLLMSFLITQKTSET
LTAVLAGGWLGLIIILFIVARIRMLYFSLPEEFLKTSSVMRVISSKLKVYFIVYMGVIFLW
SFLGGGIIYGFGAILVTVIMAFLIQLDIGRYQFVGVIDAINSYVKNKKLSRVK
>P31468
MSVSRRVIHHGLYFAVLGPLIGVLFLVLYIFFAKEPLVLWVIIHPIFLLLSITTGAIPAL
LTGVMVACLPEKIGSQKRYRCLAGGIGGVVITEIYCAVIVHIKGMASSELFENILSGDSL
VVRIIPALLAGVVMSRIITRLPGLDISCPETDSLS
>P08401
MRIGMRLLLGYFLLVAVAAWFVLAIFVKEVKPGVRRATEGTLIDTATLLAELARPDLLSG
DPTHGQLAQAFNQLQHRPFRANIGGINKVRNEYHVYMTDAQGKVLFDSANKAVGQDYSRW
NDVWLTLRGQYGARSTLQNPADPESSVMYVAAPIMDGSRLIGVLSVGKPNAAMAPVIKRS
ERRILWASAILLGIALVIGAGMVWWINRSIARLTRYADSVTDNKPVPLPDLGSSELRKLA
QALESMRVKLEGKNYIEQYVYALTHELKSPLAAIRGAAEILREGPPPEVVARFTDNILTQ
NARMQALVETLLRQARLENRQEVVLTAVDVAALFRRVSEARTVQLAEKKITLHVTPTEVN
VAAEPALLEQALGNLLDNAIDFTPESGCITLSAEVDQEHVTLKVLDTGSGIPDYALSRIF
ERFYSLPRANGQKSSGLGLAFVSEVARLFNGEVTLRNVQEGGVLASLRLHRHFT
>P0A221
MSEAKNSNLAPFRLLVKLTNGVGDEFPLYYGNNLIVLGRTIETLEFGNDNFPENIIPVTD
SKSDGIIYLTISKDNICQFSDEKGEQIDINSQFNSFEYDGISFHLKNMREDKSRGHILNG
MYKNHSVFFFFAVIVVLIIIFSLSLKKDEVKEIAEIIDDKRYGIVNTGQCNYILAETQND
AVWASVALNKTGFTKCRYILVSNKEINRIQQYINQRFPFINLYVLNLVSDKAELLVFLSK
ERNSSKDTELDKLKNALIVEFPYIKNIKFNYLSDHNARGDAKGIFTKVNVQYKEICENNK
VTYSVREELTDEKLELINRLISEHKNIYGDQYIEFSVLLIDDDFKGKSYLNSKDSYVMLN
DKHWFFLDKNK
>P09323
MNILGFFQRLGRALQLPIAVLPVAALLLRFGQPDLLNVAFIAQAGGAIFDNLALIFAIGV
ASSWSKDSAGAAALAGAVGYFVLTKAMVTINPEINMGVLAGIITGLVGGAAYNRWSDIKL
PDFLSFFGGKRFVPIATGFFCLVLAAIFGYVWPPVQHAIHAGGEWIVSAGALGSGIFGFI
NRLLIPTGLHQVLNTIAWFQIGEFTNAAGTVFHGDINRFYAGDGTAGMFMSGFFPIMMFG
LPGAALAMYFAAPKERRPMVGGMLLSVAVTAFLTGVTEPLEFLFMFLAPLLYLLHALLTG
ISLFVATLLGIHAGFSFSAGAIDYALMYNLPAASQNVWMLLVMGVIFFAIYFVVFSLVIR
MFNLKTPGREDKEDEIVTEEANSNTEEGLTQLATNYIAAVGGTDNLKAIDACITRLRLTV
ADSARVNDTMCKRLGASGVVKLNKQTIQVIVGAKAESIGDAMKKVVARGPVAAASAEATP

ATAAPVAKPQAVPNAVSIAELVSPITGDVVALDQVPDEAFASKAVGDGVAVKPTDKIVVS
PAAGTIVKIFNTNHAFCLETEKGAEIVVHMGIDTVALEGKGFKRLVEEGAQVSAGQPILE
MDLDYLNANARSMISPVVCSNIDDFSGLIIKAQGHIVAGQTPLYEIKK
>P33950
MKLKATLTLAAATLVLAACDQSSSANKSTAQTEAKSSSNNTFVYCTAKAPLGFSPALIIE
GTSYNASSQQVYNRLVEFKKGSTDIEPALAESWEISDDGLSYTFHLRKGVKFHTTKEFTP
TRDFNADDVVFSFQRQLDPNHPYHNVSKGTYPYFKAMKFPELLKSVEKVDDNTIRITLNK
TDATFLASLGMDFISIYSAEYADSMLKAGKPETLDSRPVGTGPFVFVDYKTDQAIQYVAH
ENYWKGRTPLDRLVISIVPDATTRYAKLQAGTCDLILFPNVADLAKMKTDPKVQLLEQKG
LNVAYIAFNTEKAPFDNVKVRQALNYAVDKKAIIEAVYQGAGTSAKNPLPPTIWSYNDEI
QDYPYDPEKAKQLLAEAGYPNGFETDFWIQPVIRASNPNPKRMAELIMADWAKIGVKTNP
VTYEWADYRKRAKEGELTAGIFGWSGDNGDPDNFLSPLLGSSNIGNSNMARFNNSEFDAL
LNEAIGLTNKEERAKLYKQAQVIVHNQAPWIPVAHSVGFAPLSPRVKGYVQSPFGYDAFY
GVSVDGK
>P0AF32
MIQYLNVFFYDIYPYICATVFFLGSWLRYDYGQYTWRASSSQMLDKRGMVIWSNLFHIGI
LGIFFGHLFGMLTPHWMYAWFLPVAAKQLMAMVLGGICGVLTLIGGAGLLWRRLTNQRVR
ATSTTPDIIIMSILLIQCLLGLSTIPFSAQYPDGSEMMKLVGWAQSIVTFRGGSSEMLNG
VAFVFRLHLVLGMTIFLLFPFTRLVHVWSAPFEYFTRRYQIVRSRR
>Q51330
MNNPQTGQSTGLLGNRWFYLVLAVLLMCMISGVQYSWTLYANPVKDNLGVSLAAVQTAFT
LSQVIQAGSQPGGGYFVDKFGPRIPLMFGGAMVLAGWTFMGMVDSVPALYALYTLAGAGV
GIVYGIAMNTANRWFPDKRGLASGFTAAGYGLGVLPFLPLISSVLKVEGVGAAFMYTGLI
MGILIIILIAFVIRFPGQQGAKKQIVVTDKDFNSGEMLRTPQFWVLWTAFFSVNFGGLLLV
ANSVPYGRSLGLAAGVLTIGVSIQNLFNGGCRPFWGFVSDKIGRYKTMSVVFGINAVVLA
LFPTIAALGDVAFIAMLAIAFFTWGGSYALFPSTNSDIFGTAYSARNYGFFWAAKATASI
FGGGLGAAIATNFGWNTAFLITAITSFIAFALATFVIPRMGRPVKKMVKLSPEEKAVH
>A5F389
MIKKIISVFLLLACIITSAFTAFFYHSKLSDQTKSISSLSSQQAQERLQSYQDSLDFYKK
LNISLSVAIANSLRDKAVEELNAIALRIQENHGFIGVTFASLDGTMFTDIGTLDWNAKTL
RRDWFVKTVELGTKHYTAFDIDKTTGQHVLTIATPVYVGNDIVGSVALDIAGDQIASPNG
SGMFMMTDRNFNVFASDLTHSTLIGKDLTKEKPLFKNLVSGQYVTFSDADSHWFAVSQTE
IDGENKLFTIIDIQQIVQTYKRDIQLIIAGFSGFSCVMLIGLYWVLSKELSGVRQIREWI
LSLSDGQIKERRPIKFHNELDTIAQSLENLQFRLLDVVRNSHRTMNDLSIKQTDITYSIE
GNTNNSQQELGLIEQVATATTQLSCTSFDVMQQAQSAELNAETAQKLIAESHDIIDSSSK
QTEMVTLSIHESQQIINQLREFSDNISSVTDVINNISDQTNLLALNAAIEAARAGEQGRG
FAVVADEVRSLAVKTQQSTIDIQGIILKLQEQSQLADQVMTRNVSLIHETQVANRALIAS
FNLISDKVLEISNINSIVSTAANEQKIVTEDVAKQMEDIRYLVQENLSAMERTKQANQNI
SDLTTNLNDALSFFKIELTS
>P0AG14
MELLSEYGLFLAKIVTVVLAIAAIAAIIVNVAQRNKRQRGELRVNNLSEQYKEMKEELAA
ALMDSHQQKQWHKAQKKKHKQEAKAAKAKAKLGEVATDSKPRVWVLDFKGSMDAHEVNSL
REEITAVLAAFKPQDQVVLRLESPGGMVHGYGLAASQLQRLRDKNIPLTVTVDKVAASGG
YMMACVADKIVSAPFAIVGSIGVVAQMPNFNRFLKSKDIDIELHTAGQYKRTLTLLGENT

EEGREKFREELNETHQLFKDFVKRMRPSLDIEQVATGEHWYGQQAVEKGLVDEINTSDEV
ILSLMEGREVVNVRYMQRKRLIDRFTGSAAESADRLLLRWWQRGQKPLM
>P0ADJ8
MDNKISTYSPAFSIVSWIALVGGIVTYLLGLWNAEMQLNEKGYYFAVLVLGLFSAASYQK
TVRDKYEGIPTTSIYYMTCLTVFIISVALLMVGLWNATLLLSEKGFYGLAFFLSLFGAVA
VQKNIRDAGINPPKETQVTQEEYSE
>P76361
MLQIVGALILLIAGFAILRLLFRALISTASALAGLILLCLFGPALLAGYITERITRLFHI
RWLAGVFLTIAGMIISFMWGLDGKHIALEAHTFDSVKFILTTALAGGLLAVPLQIKNIQQ
NGITPEDISKEINGYYCCFYTAFFLMACSACAPLIALQYDISPSLMWWGGLLYWLAALVT
LLWAASQIQALKKLTCAISQTLEEQPVLNSKSWLTSLQNDYSLPDSLTERIWLTLISQRI
SRGELREFELADGNWLLNNAWYERNMAGFNEQLKENLSFTPDELKTLFRNRLNLSPEAND
DFLDRCLDGGDWYPFSEGRRFVSFHHVDELRICASCGLTEVHHAPENHKPDPEWYCSSLC
RETETLCQEIYERPYNSFISDATANGLILMKLPETWSTNEKMFASGGQGHGFAAERGNHI
VDRVRLKNARILGDNNARNGADRLVSGTEIQTKYCSTAARSVGAAFDGQNGQYRYMGNNG
PMQLEVPRDQYAGAVETMRNKIREGKVEER
>P0AAS0
MTEIQRLLTETIESLNTREKRDNKPRFSISFIRKHPGLFIGMYVAFFATLAVMLQSETLS
GSVWLLVVLFILLNGFFFFDVYPRYRYEDIDVLDFRVCYNGEWYNTRFVPAALVEAILNS
PRVADVHKEQLQKMIVRKGELSFYDIFTLARAESTS
>P15643
MSKGIKMHNSVMRLTIPNKKIINYAPHIVTSIILFFICQQLAQLTWKIILPVNFTDNALS
SADMTSPAAPSAETALPRFTLFGLAEKTSASAPGGNLDQAPVSALRLRVTGLLASTDPSR
AIAIMMKGNQQVSLGIGDNTPGGEAKIIAISPDRLIVNYRGRNEAIPLFNDPPAVGKNSA
APPARHLAQELRAQPQNILHYLNISPVMVNDKLSGYRLNPGKDPALFRQSGLRENDLAIA
LNGLDLRDKEQARQVLAQLPELTEITLTVERDGQKNDIYLALRDE
>P0ABA6
MAGAKEIRSKIASVQNTQKITKAMEMVAASKMRKSQDRMAASRPYAETMRKVIGHLAHGN
LEYKHPYLEDRDVKRVGYLVVSTDRGLCGGLNINLFKKLLAEMKTWTDKGVQCDLAMIGS
KGVSFFNSVGGNVVAQVTGMGDNPSLSELIGPVKVMLQAYDEGRLDKLYIVSNKFINTMS
QVPTISQLLPLPASDDDDLKHKSWDYLYEPDPKALLDTLLRRYVESQVYQGVVENLASEQ
AARMVAMKAATDNGGSLIKELQLVYNKARQASITQELTEIVSGAAAV
>Q46892
MSTITLLCIALAGVIMLLLLVIKAKVQPFVALLLVSLLVALAAGIPAGEVGKVMIAGMGG
VLGSVTIIIGLGAMLGRMIEHSGGAESLANYFSRKLGDKRTIAALTLAAFFLGIPVFFDV
GFIILAPIIYGFAKVAKISPLKFGLPVAGIMLTVHVAVPPHPGPVAAAGLLHADIGWLTI
IGIAISIPVGVVGYFAAKIINKRQYAMSVEVLEQMQLAPASEEGATKLSDKINPPGVALV
TSLIVIPIAIIMAGTVSATLMPPSHPLLGTLQLIGSPMVALMIALVLAFWLLALRRGWSL
QHTSDIMGSALPTAAVVILVTGAGGVFGKVLVESGVGKALANMLQMIDLPLLPAAFIISL
ALRASQGSATVAILTTGGLLSEAVMGLNPIQCVLVTLAACFGGLGASHINDSGFWIVTKY
LGLSVADGLKTWTVLTTILGFTGFLITWCVWAVI
>P17334
MSNVIASLEKVLLPFAVKIGKQPHVNAIKNGFIRLMPLTLAGAMFVLINNVFLSFGEGSF
FYSLGIRLDASTIETLNGLKGIGGNVYNGTLGIMSLMAPFFIGMALAEERKVDALAAGLL

SVAAFMTVTPYSVGEAYAVGANWLGGANIISGIIIGLVVAEMFTFIVRRNWVIKLPDSVP
ASVSRSFSALIPGFIILSVMGIIAWALNTWGTNFHQIIMDTISTPLASLGSVVGWAYVIF
VPLLWFFGIHGALALTALDNGIMTPWALENIATYQQYGSVEAALAAGKTFHIWAKPMLDS
FIFLGGSGATLGLILAIFIASRRADYRQVAKLALPSGIFQINEPILFGLPIIMNPVMFIP
FVLVQPILAAITLAAYYMGIIPPVTNIAPWTMPTGLGAFFNTNGSVAALLVALFNLGIAT
LIYLPFVVVANKAQNAIDKEESEEDIANALKF
>P45306
MVMKFKMSKNVICYTWLSVCLSSAIPAFAVQPTLKPSDISIPAISEESQLATKRATTRLT
QSHYRKIKLDDDFSEKIFDRYIKNLDFNHNTFLQSDIDELRQKYGTKLDEQLNQGDLSAA
FDIYDVMMKRRYERYTYALSLLDKEPDLNGQDQIEIDREKAAAPQTEADANKLWDARVKN
DIINLKLKDKKWSEIKAKLTKRYNLAIRRLTQTKADDIVQIYLNAFAREIDPHTSYLSPR
TAKSFNESINLSLEGIGTTLQSEDDEISIKSLVPGAPAERSKKLHPGDKIIGVGQATGDI
EDVVGWRLEDLVEKIKGKKGTKVRLEIEPAKGGKSRIITLVRDKVRIEDQAAKLTFEKVS
GKNIAVIKIPSFYIGLTEDVKKLLVKLENQKAEALIVDLRENGGGALTEAVALSGLFITD
GPVVQVRDAYQRIRVHEDDDATQQYKGLLFVMINRYSASASEIFAAAMQDYRRGIIIGQN
TFGKGTVQQSRSLNFIYDLDQSPLGVLQYTIQKFYRVNGGSTQLKGVAADINFPEIIDAK
EYGEDKEDNALAWDKIPSASYMEVGNINYIDNAVNILNEKHLARIAKDPEFVALNEELKV
RNERRDRKFLSLNYKMRKAENDKDDARRLKDLNERFKREGKKALKDIDDLPKDYEAPDFF
LKEAEKIAADFVIFNSDQKINQANGLSEAKTESKK
>P0ABN1
MANNTTGFTRIIKAAGYSWKGLRAAWINEAAFRQEGVAVLLAVVIACWLDVDAITRVLLI
SSVMLVMIVEILNSAIEAVVDRIGSEYHELSGRAKDMGSAAVLIAIIVAVITWCILLWSH
FG
>P76236
MIQSTRISMGLFFKYFLSLTKIDPGQNYISLPSIKSSTHIALLFMVSMGTQKLKAQSFFI
FSLLLTLILFCITTLYNENTNVKLIPQMNYLMVVVALFFLNAVIFLFMLMKYFTNKQILP
TLILSLAFLSGLIYLVETIVIIHKPINGSTLIQTKSNDVSIFYIFRQLSFICLTSLALFC
YGKDNILDNNKKKTGILLLALIPFLVFPLLAHNLSSYNADYSLYVVDYCPDNHTATWGIN
YTKILVCLWAFLLFFIIMRTRLASELWPLIALLCLASLCCNLLLLTLDEYNYTIWYISRG
IEVSSKLFVVSFLIYNIFQELQLSSKLAVHDVLTNIYNRRYFFNSVESLLSRPVVKDFCV
MLVDINQFKRINAQWGHRVGDKVLVSIVDIIQQSIRPDDILARLEGEVFGLLFTELNSAQ
AKIIAERMRKNVELLTGFSNRYDVPEQMTISIGTVFSTGDTRNISLVMTEADKALREAKS
EGGNKVIIHHI
>P60932
MSDMHSLLIAAILGVVEGLTEFLPVSSTGHMIIVGHLLGFEGDTAKTFEVVIQLGSILAV
VVMFWRRLFGLIGIHFGRPLQHEGESKGRLTLIHILLGMIPAVVLGLLFHDTIKSLFNPI
NVMYALVVGGLLLIAAECLKPKEPRAPGLDDMTYRQAFMIGCFQCLALWPGFSRSGATIS
GGMLMGVSRYAASEFSFLLAVPMMMGATALDLYKSWGFLTSGDIPMFAVGFITAFVVALI
AIKTFLQLIKRISFIPFAIYRFIVAAAVYVVFF
>P0ADK4
MFLDYFALGVLIFVFLVIFYGIIILHDIPYLIAKKRNHPHADAIHVAGWVSLFTLHVIWP
FLWIWATLYRPERGWGMQSHDSSVMQLQQRIAGLEKQLADIKSSSAE
>P0AEB5
MIAELFTNNALNLVIIFGSCAALILMSFWFRRGNRKRKGFLFHAVQFLIYTIIISAVGSI

INYVIENYKLKFITPGVIDFICTSLIAVILTIKLFLLINQFEKQQIKKGRDITSARIMSR
IIKITIIVVLVLLYGEHFGMSLSGLLTFGGIGGLAVGMAGKDILSNFFSGIMLYFDRPFS
IGDWIRSPDRNIEGTVAEIGWRITKITTFDNRPLYVPNSLFSSISVENPGRMTNRRITTT
IGLRYEDAAKVGVIVEAVREMLKNHPAIDQRQTLLVYFNQFADSSLNIMVYCFTKTTVWA
EWLAAQQDVYLKIIDIVQSHGADFAFPSQTLYMDNITPPEQGR
>P0AFP0
MLVYWLDIVGTAVFAISGVLLAGKLRMDPFGVLVLGVVTAVGGGTIRDMALDHGPVFWVK
DPTDLVVAMVTSMLTIVLVRQPRRLPKWMLPVLDAVGLAVFVGIGVNKAFNAEAGPLIAV
CMGVITGVGGGIIRDVLAREIPMILRTEIYATACIIGGIVHATAYYTFSVPLETASMMGM
VVTLLIRLAAIRWHLKLPTFALDENGR
>P09833
MLELNFSQTLGNHCLTINETLPANGITAIFGVSGAGKTSLINAISGLTRPQKGRIVLNGR
VLNDAEKGICLTPEKRRVGYVFQDARLFPHYKVRGNLRYGMSKSMVDQFDKLVALLGIEP
LLDRLPGSLSGGEKQRVAIGRALLTAPELLLLDEPLASLDIPRKRELLPYLQRLTREINI
PMLYVSHSLDEILHLADRVMVLENGQVKAFGALEEVWGSSVMNPWLPKEQQSSILKVTVL
EHHPHYAMTALALGDQHLWVNKLDEPLQAALRIRIQASDVSLVLQPPQQTSIRNVLRAKV
VNSYDDNGQVEVELEVGGKTLWARISPWARDELAIKPGLWLYAQIKSVSITA
>P77726
MNDYKMTPGERRATWGLGTVFSLRMLGMFMVLPVLTTYGMALQGASEALIGIAIGIYGLT
QAVFQIPFGLLSDRIGRKPLIVGGLAVFAAGSVIAALSDSIWGIILGRALQGSGAIAAAV
MALLSDLTREQNRTKAMAFIGVSFGITFAIAMVLGPIITHKLGLHALFWMIAILATTGIA
LTIWVVPNSSTHVLNRESGMVKGSFSKVLAEPRLLKLNFGIMCLHILLMSTFVALPGQLA
DAGFPAAEHWKVYLATMLIAFGSVVPFIIYAEVKRKMKQVFVFCVGLIVVAEIVLWNAQT
QFWQLVVGVQLFFVAFNLMEALLPSLISKESPAGYKGTAMGVYSTSQFLGVAIGGSLGGW
INGMFDGQGVFLAGAMLAAVWLTVASTMKEPPYVSSLRIEIPANIAANEALKVRLLETEG
IKEVLIAEEEHSAYVKIDSKVTNRFEIEQAIRQA
>P16256
MQLEVILPLVAYLVVVFGISVYAMRKRSTGTFLNEYFLGSRSMGGIVLAMTLTATYISAS
SFIGGPGAAYKYGLGWVLLAMIQLPAVWLSLGILGKKFAILARRYNAVTLNDMLFARYQS
RLLVWLASLSLLVAFVGAMTVQFIGGARLLETAAGIPYETGLLIFGISIALYTAFGGFRA
SVLNDTMQGLVMLIGTVVLLIGVVHAAGGLSNAVQTLQTIDPQLVTPQGADDILSPAFMT
SFWVLVCFGVIGLPHTAVRCISYKDSKAVHRGIIIGTIVVAILMFGMHLAGALGRAVIPD
LTVPDLVIPTLMVKVLPPFAAGIFLAAPMAAIMSTINAQLLQSSATIIKDLYLNIRPDQM
QNETRLKRMSAVITLVLGALLLLAAWKPPEMIIWLNLLAFGGLEAVFLWPLVLGLYWERA
NAKGALSAMIVGGVLYAVLATLNIQYLGFHPIVPSLLLSLLAFLVGNRFGTSVPQATVLT
TDK
>P0AG96
MSANTEAQGSGRGLEAMKWVVVVALLLVAIVGNYLYRDIMLPLRALAVVILIAAAGGVAL
LTTKGKATVAFAREARTEVRKVIWPTRQETLHTTLIVAAVTAVMSLILWGLDGILVRLVS
FITGLRF
>P0AFU0
MGAYLIRRLLLVIPTLWAIITINFFIVQIAPGGPVDQAIAAIEFGNAGVLPGAGGEGVRA
SHAQTGVGNISDSNYRGGRGLDPEVIAEITHRYGFDKPIHERYFKMLWDYIRFDFGDSLF
RSASVLTLIKDSLPVSITLGLWSTLIIYLVSIPLGIRKAVYNGSRFDVWSSAFIIIGYAI

PAFLFAILLIVFFAGGSYFDLFPLRGLVSANFDSLPWYQKITDYLWHITLPVLATVIGGF
AALTMLTKNSFLDEVRKQYVVTARAKGVSEKNILWKHVFRNAMLLVIAGFPATFISMFFT
GSLLIEVMFSLNGLGLLGYEATVSRDYPVMFGTLYIFTLIGLLLNIVSDISYTLVDPRID
FEGR
>P0AGE4
MTTQRSPGLFRRLAHGSLVKQILVGLVLGILLAWISKPAAEAVGLLGTLFVGALKAVAPI
LVLMLVMASIANHQHGQKTNIRPILFLYLLGTFSAALAAVVFSFAFPSTLHLSSSAGDIS
PPSGIVEVMRGLVMSMVSNPIDALLKGNYIGILVWAIGLGFALRHGNETTKNLVNDMSNA
VTFMVKLVIRFAPIGIFGLVSSTLATTGFSTLWGYAQLLVVLVGCMLLVALVVNPLLVWW
KIRRNPFPLVLLCLRESGVYAFFTRSSAANIPVNMALCEKLNLDRDTYSVSIPLGATINM
AGAAITITVLTLAAVNTLGIPVDLPTALLLSVVASLCACGASGVAGGSLLLIPLACNMFG
ISNDIAMQVVAVGFIIGVLQDSCETALNSSTDVLFTAAACQAEDDRLANSALRN
>P00803
MANMFALILVIATLVTGILWCVDKFFFAPKRRERQAAAQAAAGDSLDKATLKKVAPKPGW
LETGASVFPVLAIVLIVRSFIYEPFQIPSGSMMPTLLIGDFILVEKFAYGIKDPIYQKTL
IETGHPKRGDIVVFKYPEDPKLDYIKRAVGLPGDKVTYDPVSKELTIQPGCSSGQACENA
LPVTYSNVEPSDFVQTFSRRNGGEATSGFFEVPKNETKENGIRLSERKETLGDVTHRILT
VPIAQDQVGMYYQQPGQQLATWIVPPGQYFMMGDNRDNSADSRYWGFVPEANLVGRATAI
WMSFDKQEGEWPTGLRLSRIGGIH
>Q92J98
MVYDFKAEIVKAKNSGSAKSGSHHWLLQRVTGIILALCSVWLIYFTLTNKNNDINIIMLW
ELKKPFNVVALLITVVISLYHAMLGMRVVIEDYISYHKLRNTLIIIVQLFCIVTIVAFVV
ALFYKG
>P29926
MTSLDFSTILPEVVLAGYALAALMAGAYLGKDRLARTLLWVTVAAFLVVAAMVGLGNHVD
GAAFHGMFIDDGFSRFAKVVTLVAAAGVLAMSADYMQRRNMLRFEFPIIVALAVLGMMFM
VSAGDLLTLYMGLELQSLALYVVAAMRRDSVRSSEAGLKYFVLGSLSSGLLLYGASLVYG
FAGTTGFEGIISTIEAGHLSLGVLFGLVFMLVGLSFKVSAVPFHMWTPDVYEGSPTPVTA
FFATAPKVAAMALIARLVFDAFGHVIGDWSQIVAALAVMSMFLGSIAGIGQTNIKRLMAY
SSIAHMGFALVGLAAGTAIGVQNMLLYMTIYAVMNIGTFAFILSMERDGVPVTDLAALNR
FAWTDPVKALAMLVLMFSLAGVPPTLGFFAKFGVLTAAVDAGMGWLAVLGVIASVIGAFY
YLRIVYYMYFGGESEGMTSRMGAVQYLALMVPALAMLVGAISMFGVDSAAGRAAETLVGP
VAAIEQPAEAAQAEPVQGE
>P37340
MQKYISEARLLLALAIPVILAQIAQTAMGFVDTVMAGGYSATDMAAVAIGTSIWLPAILF
GHGLLLALTPVIAQLNGSGRRERIAHQVRQGFWLAGFVSVLIMLVLWNAGYIIRSMENID
PALADKAVGYLRALLWGAPGYLFFQVARNQCEGLAKTKPGMVMGFIGLLVNIPVNYIFIY
GHFGMPELGGVGCGVATAAVYWVMFLAMVSYIKRARSMRDIRNEKGTAKPDPAVMKRLIQ
LGLPIALALFFEVTLFAVVALLVSPLGIVDVAGHQIALNFSSLMFVLPMSLAAAVTIRVG
YRLGQGSTLDAQTAARTGLMVGVCMATLTAIFTVSLREQIALLYNDNPEVVTLAAHLMLL
AAVYQISDSIQVIGSGILRGYKDTRSIFYITFTAYWVLGLPSGYILALTDLVVEPMGPAG
FWIGFIIGLTSAAIMMMLRMRFLQRLPSAIILQRASR
>P29131
MAQRDYVRRSQPAPSRRKKSTSRKKQRNLPAVSPAMVAIAAAVLVTFIGGLYFITHHKKE

ESETLQSQKVTGNGLPPKPEERWRYIKELESRQPGVRAPTEPSAGGEVKTPEQLTPEQRQ
LLEQMQADMRQQPTQLVEVPWNEQTPEQRQQTLQRQRQAQQLAEQQRLAQQSRTTEQSWQ
QQTRTSQAAPVQAQPRQSKPASSQQPYQDLLQTPAHTTAQSKPQQAAPVARAADAPKPTA
EKKDERRWMVQCGSFRGAEQAETVRAQLAFEGFDSKITTNNGWNRVVIGPVKGKENADST
LNRLKMAGHTNCIRLAAGG
>P76345
MNRFSKTQIYLHWITLLFVAITYAAMELRGWFPKGSSTYLLMRETHYNAGIFVWVLMFSR
LIIKHRYSDPSIVPPPPAWQMKAASLMHIMLYITFLALPLLGIALMAYSGKSWSFLGFNV
SPFVTPNSEIKALIKNIHETWANIGYFLIAAHAGAALFHHYIQKDNTLLRMMPRRK
>P41441
MNYRYRAMTQDGQKLQGIIDANDERQARLRLREEGLFLLDIRPQKSSGVKTRRPRISHSE
LTLFTRQLATLSAAALPLEESLAVIGQQSSNKRLGDVLNQVRSAILEGHPLSDALQHFPT
LFDSLYRTLVKAGEKSGLLAPVLEKLADYNENRQKIRSKLIQSLIYPCMLTTVAIGVVII
LLTAVVPKITEQFVHMKQQLPLSTRILLGLSDTLQRTGPTLLATVFIVAVGFWLWLKRGN
NRHRFHAMLLRVALIGPLICAINSARYLRTLSILQSSGVPLLDGMNLSTESLNNLEIRQR
LANAAENVRQGNSIHLSLEQTAIFPPMMLYMVASGEKSGQLGTLMVRAADNQETLQQNRI
ALTLSIFEPALIITMALIVLFIVVSVLQPLLQLNSMIN
>P0ABI8
MFGKLSLDAVPFHEPIVMVTIAGIILGGLALVGLITYFGKWTYLWKEWLTSVDHKRLGIM
YIIVAIVMLLRGFADAIMMRSQQALASAGEAGFLPPHHYDQIFTAHGVIMIFFVAMPFVI
GLMNLVVPLQIGARDVAFPFLNNLSFWFTVVGVILVNVSLGVGEFAQTGWLAYPPLSGIE
YSPGVGVDYWIWSLQLSGIGTTLTGINFFVTILKMRAPGMTMFKMPVFTWASLCANVLII
ASFPILTVTVALLTLDRYLGTHFFTNDMGGNMMMYINLIWAWGHPEVYILILPVFGVFSE
IAATFSRKRLFGYTSLVWATVCITVLSFIVWLHHFFTMGAGANVNAFFGITTMIIAIPTG
VKIFNWLFTMYQGRIVFHSAMLWTIGFIVTFSVGGMTGVLLAVPGADFVLHNSLFLIAHF
HNVIIGGVVFGCFAGMTYWWPKAFGFKLNETWGKRAFWFWIIGFFVAFMPLYALGFMGMT
RRLSQQIDPQFHTMLMIAASGAVLIALGILCLVIQMYVSIRDRDQNRDLTGDPWGGRTLE
WATSSPPPFYNFAVVPHVHERDAFWEMKEKGEAYKKPDHYEEIHMPKNSGAGIVIAAFST
IFGFAMIWHIWWLAIVGFAGMIITWIVKSFDEDVDYYVPVAEIEKLENQHFDEITKAGLK
NGN
>P0A8K8
MLKTIQDKARHRTRPLWAWLKLLWQRIDEDNMTTLAGNLAYVSLLSLVPLVAVVFALFAA
FPMFSDVSIQLRHFIFANFLPATGDVIQRYIEQFVANSNKMTAVGACGLIVTALLLMYSI
DSALNTIWRSKRARPKIYSFAVYWMILTLGPLLAGASLAISSYLLSLRWASDLNTVIDNV
LRIFPLLLSWISFWLLYSIVPTIRVPNRDAIVGAFVAALLFEAGKKGFALYITMFPSYQL
IYGVLAVIPILFVWVYWTWCIVLLGAEITVTLGEYRKLKQAAEQEEDDEP
>P26417
MTDSAINKKSKRSIWIPLLVLITLAACATAGYSYWRMQQQPTTNAKAEPAPPPAPVFFAL
DTFTVNLGDADRVLYIGVTLRLKDEATRARLNEYLPEVRSRLLLLFSRQNAAELSTEAGK
QKLIAAIKETLAAPLVAGQPKQVVTDVLYTAFILR
>P69367
MSRVSQARNLGKYFLLIDNMLVVLGFFVVFPLISIRFVDQMGWAAVMVGIALGLRQFIQQ
GLGIFGGAIADRFGAKPMIVTGMLMRAAGFATMGIAHEPWLLWFSCLLSGLGGTLFDPPR
SALVVKLIRPQQRGRFFSLLMMQDSAGAVIGALLGSWLLQYDFRLVCATGAVLFVLCAAF

NAWLLPAWKLSTVRTPVREGMTRVMRDKRFVTYVLTLAGYYMLAVQVMLMLPIMVNDVAG
APSAVKWMYAIEACLSLTLLYPIARWSEKHFRLEHRLMAGLLIMSLSMMPVGMVSGLQQL
FTLICLFYIGSIIAEPARETLSASLADARARGSYMGFSRLGLAIGGAIGYIGGGWLFDLG
KSAHQPELPWMMLGIIGIFTFLALGWQFSQKRAARRLLERDA
>P76090
MTLYQIKPLFQSLLRPTMFWLYKHHVTANHITLAALALSLLTGLLLMLAAQPILFLLLPI
VLFIRMALNALDGMLARECNQQTRLGAILNETGDVISDIALYLPFLFLPESNASLVILML
FCTILTEFCGLLAQTINGVRSYAGPFGKSDRALIFGLWGLAVAIYPQWMQWNNLLWSIAS
ILLLWTAINRCRSVLLMSAEI
>P15279
MSRFVTSVSALAMLALAPAALSSVAYANDKLVELSKSDDNWVMPGKNYDSNNYSELKQVN
KSNVKQLRPAWTFSTGLLNGHEGAPLVVDGKMYVHTSFPNNTFALDLDDPGHILWQDKPK
QNPAARAVACCDLVNRGLAYWPGDGKTPALILKTQLDRHVVALNAETGETVWKVENSDIK
VGSTLTIAPYVVKDKVIIGSSGAELGVRGYLTAYDVKTGGQVWRAYATGPDKDLLLADDF
NVKNAHYGQKGLGTATWEGDAWKIGGGTNWGWYAYDPGTNLIYFGTGNPAPWNETMRPGD
NKWTMTIFGRDADTGEAKFGYQKTPHDEWDYAGVNVMMPSEQKDKDGKTRKLLTHPDRNG
IVYTLDRTDGALVSANKLDDTVNVFKTVDLKTGQPVRDPEYGTRMDHLAKDVCPSAMGYH
NQGHDSYDPKRELFFMGINHICMDWEPFMLPYRAGQFFVGATLNMYPGPKGDRQNYEGLG
QIKAYNAITGSYKWEKMERFAVWGGTLATAGDLVFYGTLDGYLKARDSDTGDLLWKFKIP
SGAIGYPMTYTHKGTQYVAIYYGVGGWPGVGLVFDLADPTAGLGAVGAFKKLANYTQQGG
GVIVFSLDGKGPYDDPNVGEWKSASK
>P76474
MGLMWGLFSVIIASVAQLSLGFAASHLPPMTHLWDFIAALLAFGLDARILLLGLLGYLLS
VFCWYKTLHKLALSKAYALLSMSYVLVWIASMVLPGWEGTFSLKALLGVACIMSGLMLIF
LPTTKQRY
>P0ACV4
MKYLLIFLLVLAIFVISVTLGAQNDQQVTFNYLLAQGEYRISTLLAVLFAAGFAIGWLIC
GLFWLRVRVSLARAERKIKRLENQLSPATDVAVVPHSSAAKE
>P0AD03
MALNTPQITPTKKITVRAIGEELPRGDYQRCPQCDMLFSLPEINSHQSAYCPRCQAKIRD
GRDWSLTRLAAMAFTMLLLMPFAWGEPLLHIWLLGIRIDANVMQGIWQMTKQGDAITGSM
VFFCVIGAPLILVTSIAYLWFGNRLGMNLRPVLLMLERLKEWVMLDIYLVGIGVASIKVQ
DYAHIQAGVGLFSFVALVILTTVTLSHLNVEELWERFYPQRPATRRDEKLRVCLGCHFTG
YPDQRGRCPRCHIPLRLRRRHSLQKCWAALLASIVLLLPANLLPISIIYLNGGRQEDTIL
SGIMSLASSNIAVAGIVFIASILVPFTKVIVMFTLLLSIHFKCQQGLRTRILLLRMVTWI
GRWSMLDLFVISLTMSLINRDQILAFTMGPAAFYFGAAVILTILAVEWLDSRLLWDAHES
GNARFDD
>P37630
MHISILAGIPEEFKVNIYIGWLFKLIPLIMGLICIALGGFVLESSGQSEYFVAGHVLISL
AAICLALFTTAFIIISQLTRGVNTFYNTLFPIIGYAGSIITMIWGWALLAGNDVMADEFV
AGHVIFGVGMIAACVSTVAASSGHFLLIPKNAAGSKSDGTPVQAYSSLIGNCLIAVPVLL
TLLGFIWSITLLRSADITPHYVAGHVLLGLTAICACLIGLVATIVHQTRNTFSTKEHWLW
CYWVIFLGSITVLQGIYVLVSSDASARLAPGIILICLGMICYSIFSKVWLLALVWRRTCS
LANRIPMIPVFTCLFCLFLASFLAEMAQTDMGYFIPSRVLVGLGAVCFTLFSIVSILEAG

SAKK
>P18004
MNNPLEAVTQAVNSLVTALKLPDESAKANEVLGEMSFPQFSRLLPYRDYNQESGLFMNDT
TMGFMLEAIPINGANESIVEALDHMLRTKLPRGIPLCIHLMSSQLVGDRIEYGLREFSWS
GEQAERFNAITRAYYMKAAATQFPLPEGMNLPLTLRHYRVFISYCSPSKKKSRADILEME
NLVKIIRASLQGASITTQTVDAQAFIDIVGEMINHNPDSLYPKRRQLDPYSDLNYQCVED
SFDLKVRADYLTLGLRENGRNSTARILNFHLARNPEIAFLWNMADNYSNLLNPELSISCP
FILTLTLVVEDQVKTHSEANLKYMDLEKKSKTSYAKWFPSVEKEAKEWGELRQRLGSGQS
SVVSYFLNITAFCKDNNETALEVEQDILNSFRKNGFELISPRFNHMRNFLTCLPFMAGKG
LFKQLKEAGVVQRAESFNVANLMPLVADNPLTPAGLLAPTYRNQLAFIDIFFRGMNNTNY
NMAVCGTSGAGKTGLIQPLIRSVLDSGGFAVVFDMGDGYKSLCENMGGVYLDGETLRFNP
FANITDIDQSAERVRDQLSVMASPNGNLDEVHEGLLLQAVRASWLAKENRARIDDVVDFL
KNASDSEQYAESPTIRSRLDEMIVLLDQYTANGTYGQYFNSDEPSLRDDAKMVVLELGGL
EDRPSLLVAVMFSLIIYIENRMYRTPRNLKKLNVIDEGWRLLDFKNHKVGEFIEKGYRTA
RRHTGAYITITQNIVDFDSDKASSAARAAWGNSSYKIILKQSAKEFAKYNQLYPDQFLPL
QRDMIGKFGAAKDQWFSSFLLQVENHSSWHRLFVDPLSRAMYSSDGPDFEFVQQKRKEGL
SIHEAVWQLAWKKSGPEMASLEAWLEEHEKYRSVA
>P0A9P0
MSTEIKTQVVVLGAGPAGYSAAFRCADLGLETVIVERYNTLGGVCLNVGCIPSKALLHVA
KVIEEAKALAEHGIVFGEPKTDIDKIRTWKEKVINQLTGGLAGMAKGRKVKVVNGLGKFT
GANTLEVEGENGKTVINFDNAIIAAGSRPIQLPFIPHEDPRIWDSTDALELKEVPERLLV
MGGGIIGLEMGTVYHALGSQIDVVEMFDQVIPAADKDIVKVFTKRISKKFNLMLETKVTA
VEAKEDGIYVTMEGKKAPAEPQRYDAVLVAIGRVPNGKNLDAGKAGVEVDDRGFIRVDKQ
LRTNVPHIFAIGDIVGQPMLAHKGVHEGHVAAEVIAGKKHYFDPKVIPSIAYTEPEVAWV
GLTEKEAKEKGISYETATFPWAASGRAIASDCADGMTKLIFDKESHRVIGGAIVGTNGGE
LLGEIGLAIEMGCDAEDIALTIHAHPTLHESVGLAAEVFEGSITDLPNPKAKKK
>P33788
MPKIQTYVNNNVYEQITDLVTIRKQEGIEEASLSNVSSMLLELGLRVYMIQQEKREGGFN
QMEYNKLMLENVSRVRAMCTEILKMSVLNQESIASGNFDYAVIKPAIDKFAREQVSIFFP
DDEDDQE
>P0AA47
MSHNVTPNTSRVELRKTLTLVPVVMMGLAYMQPMTLFDTFGIVSGLTDGHVPTAYAFALI
AILFTALSYGKLVRRYPSAGSAYTYAQKSISPTVGFMVGWSSLLDYLFAPMINILLAKIY
FEALVPSIPSWMFVVALVAFMTAFNLRSLKSVANFNTVIVVLQVVLIAVILGMVVYGVFE
GEGAGTLASTRPFWSGDAHVIPMITGATILCFSFTGFDGISNLSEETKDAERVIPRAIFL
TALIGGMIFIFATYFLQLYFPDISRFKDPDASQPEIMLYVAGKAFQVGALIFSTITVLAS
GMAAHAGVARLMYVMGRDGVFPKSFFGYVHPKWRTPAMNIILVGAIALLAINFDLVMATA
LINFGALVAFTFVNLSVISQFWIREKRNKTLKDHFQYLFLPMCGALTVGALWVNLEESSM
VLGLIWAAIGLIYLACVTKSFRNPVPQYEDVA
>P08691
MLLAGAIFILTIVLVIWQPKGLGIGWSATLGAVLALASGVIHIADIPVVWNIVWNATATF
IAVIIISLLLDESGFFEWAALHVSRWGNGRGRLLFTYIVLLGAAVAALFANDGAALILTP
IVIAMLLALGFSKSTTLAFVMAAGFISDTASLPLIVSNLVNIVSADFFKLGFTEYASVMV
PVDIAAIIATLVMLHLFFRKDIPPTYELARLKEPAKAIKDPATFRTGWVVLLLLLLVGFFV

LEPMGIPVSAIAAVGAAVLFAVAKKGHGINTGKVLRGAPWQIVIFSLGMYLVIYGLRNAG
LTDYLSDVLNELADKGLWAATLGTGFLTALLSSIMNNMPTVLIGALSIDGSTATGVIKEA
MIYANVIGCDLGPKITPIGSLATLLWLHVLSQKNMTITWGYYFRTGIVMTLPVLFVTLAA
LALRLSVTL
>Q00593
MYDYIIVGAGSAGCVLANRLSADPSKRVCLLEAGPRDTNPLIHMPLGIALLSNSKKLNWA
FQTAPQQNLNGRSLFWPRGKTLGGSSSINAMVYIRGHEDDYHAWEQAAGRYWGWYRALEL
FKRLECNQRFDKSEHHGVDGELAVSDLKYINPLSKAFVQAGMEANINFNGDFNGEYQDGV
GFYQVTQKNGQRWSSARAFLHGVLSRPNLDIITDAHASKILFEDRKAVGVSYIKKNMHHQ
VKTTSGGEVLLSLGAVGTPHLLMLSGVGAAAELKEHGVSLVHDLPEVGKNLQDHLDITLM
CAANSREPIGVALSFIPRGVSGLFSYVFKREGFLTSNVAESGGFVKSSPDRDRPNLQFHF
LPTYLKDHGRKIAGGYGYTLHICDLLPKSRGRIGLKSANPLQPPLIDPNYLSDHEDIKTM
IAGIKIGRAILQAPSMAKHFKHEVVPGQAVKTDDEIIEDIRRRAETIYHPVGTCRMGKDP
ASVVDPCLKIRGLANIRVVDASIMPHLVAGNTNAPTIMIAENAAEIIMRNLDVEALEASA
EFAREGAELELAMIAVCM
>P27457
MARNNTITLYDLQLESGCTISPYVWRTKYALKHKGFDIDIVPGGFTGILERTGGRSERVP
VIVDDGEWVLDSWVIAEYLDEKYPDRPMLFEGPTQKNLMKFLDNWLWSTAVGPWFRCYIL
DYHDLSLPQDRDYVRWSREQWFLGGQRLEDVQAGREDRLPLVPPTLEPFRRILAETKWLG
GDQPNFADYSALAVFLWTASVARTPPLTEDDPLRDWLDRGFDLFDGLGRHPGMNPLFGLK
LREGDPEPFVRQTGPAGAGGQALNKGPQTTKMPPRVAEKAD
>P64606
MLLNALASLGHKGIKTLRTFGRAGLMLFNALVGKPEFRKHAPLLVRQLYNVGVLSMLIIV
VSGVFIGMVLGLQGYLVLTTYSAETSLGMLVALSLLRELGPVVAALLFAGRAGSALTAEI
GLMRATEQLSSMEMMAVDPLRRVISPRFWAGVISLPLLTVIFVAVGIWGGSLVGVSWKGI
DSGFFWSAMQNAVDWRMDLVNCLIKSVVFAITVTWISLFNGYDAIPTSAGISRATTRTVV
HSSLAVLGLDFVLTALMFGN
>P0ABV6
MARARGRGRRDLKSEINIVPLLDVLLVLLLIFMATAPIITQSVEVDLPDATESQAVSSND
NPPVIVEVSGIGQYTVVVEKDRLERLPPEQVVAEVSSRFKANPKTVFLIGGAKDVPYDEI
IKALNLLHSAGVKSVGLMTQPI
>P0A1J9
MSNLSGTDKSVILLMTIGEDRAAEVFKHLSTREVQALSTAMANVRQISNKQLTDVLSEFE
QEAEQFAALNINANEYLRSVLVKALGEERASSLLEDILETRDTTSGIETLNFMEPQSAAD
LIRDEHPQIIATILVHLKRSQAADILALFDERLRHDVMLRIATFGGVQPAALAELTEVLN
GLLDGQNLKRSKMGGVRTAAEIINLMKTQQEEAVITAVREFDGELAQKIIDEMFLFENLV
DVDDRSIQRLLQEVDSESLLIALKGAEPPLREKFLRNMSQRAADILRDDLANRGPVRLSQ
VENEQKAILLIVRRLAETGEMVIGSGEDTYV
>P10903
MSHSSAPERATGAVITDWRPEDPAFWQQRGQRIASRNLWISVPCLLLAFCVWMLFSAVAV
NLPKVGFNFTTDQLFMLTALPSVSGALLRVPYSFMVPIFGGRRWTAFSTGILIIPCVWLG
FAVQDTSTPYSVFIIISLLCGFAGANFASSMANISFFFPKQKQGGALGLNGGLGNMGVSV
MQLVAPLVVSLSIFAVFGSQGVKQPDGTELYLANASWIWVPFLAIFTIAAWFGMNDLATS
KASIKEQLPVLKRGHLWIMSLLYLATFGSFIGFSAGFAMLSKTQFPDVQILQYAFFGPFI

GALARSAGGALSDRLGGTRVTLVNFILMAIFSGLLFLTLPTDGQGGSFMAFFAVFLALFL
TAGLGSGSTFQMISVIFRKLTMDRVKAEGGSDERAMREAATDTAAALGFISAIGAIGGFF
IPKAFGSSLALTGSPVGAMKVFLIFYIACVVITWAVYGRHSKK
>P77610
MSKHDTDTSDQHAAKRRWLNAHEEGYHKAMGNRQVQMIAIGGAIGTGLFLGAGARLQMAG
PALALVYLICGLFSFFILRALGELVLHRPSSGSFVSYAREFLGEKAAYVAGWMYFINWAM
TGIVDITAVALYMHYWGAFGGVPQWVFALAALTIVGTMNMIGVKWFAEMEFWFALIKVLA
IVTFLVVGTVFLGSGQPLDGNTTGFHLITDNGGFFPHGLLPALVLIQGVVFAFASIEMVG
TAAGECKDPQTMVPKAINSVIWRIGLFYVGSVVLLVMLLPWSAYQAGQSPFVTFFSKLGV
PYIGSIMNIVVLTAALSSLNSGLYCTGRILRSMAMGGSAPSFMAKMSRQHVPYAGILATL
VVYVVGVFLNYLVPSRVFEIVLNFASLGIIASWAFIIVCQMRLRKAIKEGKAADVSFKLP
GAPFTSWLTLLFLLSVLVLMAFDYPNGTYTIAALPIIGILLVIGWFGVRKRVAEIHSTAP
VVEEDEEKQEIVFKPETAS
>P64432
MPVFALLALVAYSVSLALIVPGLLQKNGGWRRMAIISAVIALVCHAIALEARILPDGDSG
QNLSLLNVGSLVSLMICTVMTIVASRNRGWLLLPIVYAFALINLALATFMPNEYITHLEA
TPGMLVHIGLSLFSYATLIIAALYALQLAWIDYQLKNKKLAFNQEMPPLMSIERKMFHIT
QIGVVLLTLTLCTGLFYMHNLFSMENIDKAVLSIVAWFVYIVLLWGHYHEGWRGRRVVWF
NVAGAVILTLAYFGSRIVQQLIS
>O52043
MESLQQLQQQLGLMAWPLFICSALTVMLLAERLFQVLLSLTVGKGAIRHALQATSPKNPK
QLAELTEHFASKRPVLYRGVAMLLAHHQFDKSLREDAAGIWLQEQRHQFNSGLRLLTLIG
VISPLLGLLGTVLGLIEMFKGVAATTGSITPNVLADGLGVAMYTTAAGLLIAVPAVAGAQ
LLSLWADRTMAKLEHTLNYVNLWLEGMTLHADASLTVVTPQEATTENL
>P0A8R7
MTEPLKPRIDFDGPLEVDQNPKFRAQQTFDENQAQNFAPATLDEAQEEEGQVEAVMDAAL
RPKRSLWRKMVMGGLALFGASVVGQGVQWTMNAWQTQDWVALGGCAAGALIIGAGVGSVV
TEWRRLWRLRQRAHERDEARDLLHSHGTGKGRAFCEKLAQQAGIDQSHPALQRWYASIHE
TQNDREVVSLYAHLVQPVLDAQARREISRSAAESTLMIAVSPLALVDMAFIAWRNLRLIN
RIATLYGIELGYYSRLRLFKLVLLNIAFAGASELVREVGMDWMSQDLAARLSTRAAQGIG
AGLLTARLGIKAMELCRPLPWIDDDKPRLGDFRRQLIGQVKETLQKGKTPSEK
>Q46755
MTSFSTLLSVHLISIALSVGLLTLRFWLRYQKHPQAFARWTRIVPPVVDTLLLLSGIALM
AKAHILPFSGQAQWLTEKLFGVIIYIVLGFIALDYRRMHSQQARIIAFPLALVVLYIIIK
LATTKVPLLG
>P03819
MDSHTLIQALIYLGSAALIVPIAVRLGLGSVLGYLIAGCIIGPWGLRLVTDAESILHFAE
IGVVLMLFIIGLELDPQRLWKLRAAVFGCGALQMVICGGLLGLFCMLLGLRWQVAELIGM
TLALSSTAIAMQAMNERNLMVTQMGRSAFAVLLFQDIAAIPLVAMIPLLATSSASTTMGA
FALSALKVAGALVLVVLLGRYVTRPALRFVARSGLREVFSAVALFLVFGFGLLLEEVGLS
MAMGAFLAGVLLASSEYRHALESDIEPFKGLLLGLFFIGVGMSIDFGTLLENPLRIVILL
LGFLIIKIAMLWLIARPLQVPNKQRRWFAVLLGQGSEFAFVVFGAAQMANVLEPEWAKSL
TLAVALSMAATPILLVILNRLEQSSTEEAREADEIDEEQPRVIIAGFGRFGQITGRLLLS
SGVKMVVLDHDPDHIETLRKFGMKVFYGDATRMDLLESAGAAKAEVLINAIDDPQTNLQL

TEMVKEHFPHLQIIARARDVDHYIRLRQAGVEKPERETFEGALKTGRLALESLGLGPYEA
RERADVFRRFNIQMVEEMAMVENDTKARAAVYKRTSAMLSEIITEDREHLSLIQRHGWQG
TEEGKHTGNMADEPETKPSS
>P75831
MTPLLELKDIRRSYPAGDEQVEVLKGISLDIYAGEMVAIVGASGSGKSTLMNILGCLDKA
TSGTYRVAGQDVATLDADALAQLRREHFGFIFQRYHLLSHLTAEQNVEVPAVYAGLERKQ
RLLRAQELLQRLGLEDRTEYYPAQLSGGQQQRVSIARALMNGGQVILADEPTGALDSHSG
EEVMAILHQLRDRGHTVIIVTHDPQVAAQAERVIEIRDGEIVRNPPAIEKVNVTGGTEPV
VNTVSGWRQFVSGFNEALTMAWRALAANKMRTLLTMLGIIIGIASVVSIVVVGDAAKQMV
LADIRSIGTNTIDVYPGKDFGDDDPQYQQALKYDDLIAIQKQPWVASATPAVSQNLRLRY
NNVDVAASANGVSGDYFNVYGMTFSEGNTFNQEQLNGRAQVVVLDSNTRRQLFPHKADVV
GEVILVGNMPARVIGVAEEKQSMFGSSKVLRVWLPYSTMSGRVMGQSWLNSITVRVKEGF
DSAEAEQQLTRLLSLRHGKKDFFTWNMDGVLKTVEKTTRTLQLFLTLVAVISLVVGGIGV
MNIMLVSVTERTREIGIRMAVGARASDVLQQFLIEAVLVCLVGGALGITLSLLIAFTLQL
FLPGWEIGFSPLALLLAFLCSTVTGILFGWLPARNAARLDPVDALARE
>Q59660
MRYITPRKAAEGLGSAHEGTQHHWAMTVSAVALTVLTPLFMIVVARAIGLSQEQLLAYFG
RPFPALITALFVIVGMVHFIKGTRIMIDDYFQGGTRKAAIIFSVIFGWAVIAAAVYALAR
MGLGAIVVL
>P16552
MNSASTHKNTDFWIFGLFFFLYFFIMATCFPFLPVWLSDVVGLSKTDTGIVFSCLSLFAI
SFQPLLGVISDRLGLKKNLIWSISLLLVFFAPFFLYVFAPLLHLNIWAGALTGGVFIGFV
FSAGAGAIEAYIERVSRSSGFEYGKARMFGCLGWALCATMAGILFNVDPSLVFWMGSGGA
LLLLLLLYLARPSTSQTAMVMNALGANSSLISTRMVFSLFRMRQMWMFVLYTIGVACVYD
VFDQQFAIFFRSFFDTPQAGIKAFGFATTAGEICNAIIMFCTPWIINRIGAKNTLLVAGG
IMTIRITGSAFATTMTEVVILKMLHALEVPFLLVGAFKYITGVFDTRLSATVYLIGFQFS
KQLAAILLSTFAGHLYDRMGFQNTYFVLGMIVLTVTVISAFTLSSSPGIVHPSVEKAPVA
HSEIN
>P0A6Y8
MGKIIGIDLGTTNSCVAIMDGTTPRVLENAEGDRTTPSIIAYTQDGETLVGQPAKRQAVT
NPQNTLFAIKRLIGRRFQDEEVQRDVSIMPFKIIAADNGDAWVEVKGQKMAPPQISAEVL
KKMKKTAEDYLGEPVTEAVITVPAYFNDAQRQATKDAGRIAGLEVKRIINEPTAAALAYG
LDKGTGNRTIAVYDLGGGTFDISIIEIDEVDGEKTFEVLATNGDTHLGGEDFDSRLINYL
VEEFKKDQGIDLRNDPLAMQRLKEAAEKAKIELSSAQQTDVNLPYITADATGPKHMNIKV
TRAKLESLVEDLVNRSIEPLKVALQDAGLSVSDIDDVILVGGQTRMPMVQKKVAEFFGKE
PRKDVNPDEAVAIGAAVQGGVLTGDVKDVLLLDVTPLSLGIETMGGVMTTLIAKNTTIPT
KHSQVFSTAEDNQSAVTIHVLQGERKRAADNKSLGQFNLDGINPAPRGMPQIEVTFDIDA
DGILHVSAKDKNSGKEQKITIKASSGLNEDEIQKMVRDAEANAEADRKFEELVQTRNQGD
HLLHSTRKQVEEAGDKLPADDKTAIESALTALETALKGEDKAAIEAKMQELAQVSQKLME
IAQQQHAQQQTAGADASANNAKDDDVVDAEFEEVKDKK
>P75770
MSKSHPRWRLAKKILTWLFFIAVIVLLVVYAKKVDWEEVWKVIRDYNRVALLSAVGLVVV
SYLIYGCYDLLARFYCGHKLAKRQVMLVSFICYAFNLTLSTWVGGIGMRYRLYSRLGLPG
STITRIFSLSITTNWLGYILLAGIIFTAGVVELPDHWYVDQTTLRILGIGLLMIIAVYLW

FCAFAKHRHMTIKGQKLVLPSWKFALAQMLISSVNWMVMGAIIWLLLGQSVNYFFVLGVL
LVSSIAGVIVHIPAGIGVLEAVFIALLAGEHTSKGTIIAALLAYRVLYYFIPLLLALICY
LLLESQAKKLRAKNEAAM
>P31710
MKLKSGIVTGVALVLAYGLFLASYAPARLLTAVPLPAGMVVAEAAGTLWQGSLQRFSWRT
LTLDDVHWNITFSDFMPALDIAFKNPEGIAGRGIIRGWQRAQFYQWQLSVPAGYLFSHMR
FIVPIGAEGNVQLNLQEATVDRSGCQSLDANVTWPGARVKTPLGGLVLATPQATLRCQQG
ALEANLRQTSSHLQLSGKGSVTPKGEYRFTGQLSSGNDLPATMKKLLATTGKANEQGART
LNFQGRLL
>P08369
MLKSPLFWKMTSLFGAVLLLLIPIMLIRQVIVERADYRSDVEDAIRQSTSGPQKLVGPLI
AIPVTELYTVQEEDKTVERKRSFIHFWLPESLMVDGNQNVEERKIGIYTGQVWHSDLTLK
ADFDVSRLSELNAPNITLGKPFIVISVGDARGIGVVKAPEVNGTALTIEPGTGLEQGGQG
VHIPLPEGDWRKQNLKLNMALNLSGTGDLSVVPGGRNSEMTLTSNWPHPSFLGDFLPAKR
EVSESGFQAHWQSSWFANNLGERFASGNDTGWENFPAFSVAVTTPADQYQLTDRATKYAI
LLIALTFMAFFVFETLTAQRLHPMQYLLVGLSLVMFYLLLLALSEHTGFTVAWIIASLIG
AIMNGIYLQAVLKGWCNSMLFTLALLLLDGVMWGLLNSADSALLLGTSVLVVALAGMMFV
TRNIDWYAFSLPKMKASKEVTTDDELRIWK

(2) $\mathbb{S}_2$: 124 cell outer membrane proteins

>P41052
MFKRRYVTLLPLFVLLAACSSKPKPTETDTTTGTPSGGFLLEPQHNVMQMGGDFANNPNA
QQFIDKMVNKHGFDRQQLQEILSQAKRLDSVLRLMDNQAPTTSVKPPSGPNGAWLRYRKK
FITPDNVQNGVVFWNQYEDALNRAWQVYGVPPEIIVGIIGVETRWGRVMGKTRILDALAT
LSFNYPRRAEYFSGELETFLLMARDEQDDPLNLKGSFAGAMGYGQFMPSSYKQYAVDFSG
DGHINLWDPVDAIGSVANYFKAHGWVKGDQVAVMANGQAPGLPNGFKTKYSISQLAAAGL
TPQQPLGNHQQASLLRLDVGTGYQYWYGLPNFYTITRYNHSTHYAMAVWQLGQAVALARV
Q
>P07110
MKDRIPFAVNNITCVILLSLFCNAASAVEFNTDVLDAADKKNIDFTRFSEAGYVLPGQYL
LDVIVNGQSISPASLQISFVEPALSGDKAEKKLPQACLTSDMVRLMGLTAESLDKVVYWH
DGQCADFHGLPGVDIRPDTGAGVLRINMPQAWLEYSDATWLPPSRWDDGIPGLMLDYNLN
GTVSRNYQGGDSHQFSYNGTVGGNLGPWRLRADYQGSQEQSRYNGEKTTNRNFTWSRFYL
FRAIPRWRANLTLGENNINSDIFRSWSYTGASLESDDRMLPPRLRGYAPQITGIAETNAR
VVVSQQGRVLYDSMVPAGPFSIQDLDSSVRGRLDVEVIEQNGRKKTFQVDTASVPYLTRP
GQVRYKLVSGRSRGYGHETEGPVFATGEASWGLSNQWSLYGGAVLAGDYNALAAGAGWDL
GVPGTLSADITQSVARIEGERTFQGKSWRLSYSKRFDNADADITFAGYRFSERNYMTMEQ
YLNARYRNDYSSREKEMYTVTLNKNVADWNTSFNLQYSRQTYWDIRKTDYYTVSVNRYFN
VFGLQGVAVGLSASRSKYLGRDNDSAYLRISVPLGTGTASYSGSMSNDRYVNMAGYTDTF
NDGLDSYSLNAGLNSGGGLTSQRQINAYYSHRSPLANLSANIASLQKGYTSFGVSASGGA
TITGKGAALHAGGMSGGTRLLVDTDGVGGVPVDGGQVVTNRWGTGVVTDISSYYRNTTSV
DLKRLPDDVEATRSVVESALTEGAIGYRKFSVLKGKRLFAILRLADGSQPPFGASVTSEK
GRELGMVADEGLAWLSGVTPGETLSVNWDGKIQCQVNVPETAISDQQLLLPCTPQK
>Q00738

MCAMDRRERALISQLHFTHSLGEAISFLKYPVCVRFEDGSFIKENNCFEKLIRSSFNSCD
EWFDSLKLECKLQLSRAEIESCSSIYGVNCNNDILLNNVLWSVIIESVITPCGYFFIWRF
IWVANDNLSSFVVSKYSNELIVPSDEYVGIEPYLIGFSHHYSSAKMNITVSKSKKKTMKL
FKRYGFSSRDLWLDEMIRTEKILPLYAKVKEILGR
>P16239
MKEDKVLILRTCANNMADHGGIIWPLSGIVECKYWKPVKGFENGLTGLIWGKGSDSPLSL
HADARWVVAEVDADECIAIETHGWIKFPRAEVLHVGTKTSAMQFILHHRADYVACTEMQA
GPGSPDVTSEVKVGNRSLPVTDDIDATIESGSTQPTQTIEIATYGSTLSGTHQSQLIAGY
GSTETAGDSSTLIAGYGSTGTAGADSTLVAGYGSTQTAGEESSQMAGYGSTQTGMKGSDL
TAGYGSTGTAGDDSSLIAGYGSTQTAGEDSSLTAGYGSTQTAQKGSDLTAGYGSTGTAGA
DSSLIAGYGSTQTAGEESTQTAGYGSTQTAQKGSDLTAGYGSTGTAGDDSSLIAGYGSTQ
TAGEDSSLTAGYGSTQTAQKGSDLTAGYGSTGTAGADSSLIAGYGSTQTAGEESTQTAGY
GSTQTAQKGSDLTAGYGSTGTAGDDSSLIAGYGSTQTAGEDSSLTAGYGSTQTAQKGSDL
TAGYGSTSTAGYESSLIAGYGSTQTAGYGSTLTAGYGSTQTAQNESDLITGYGSTSTAGA
NSSLIAGYGSTQTASYNSVLTAGYGSTQTAREGSDLTAGYGSTGTAGSDSSIIAGYGSTQ
TASYHSSLTAGYGSTQTAREQSVLTTGYGSTSTAGADSSLIAGYGSTQTAGYNSILTAGY
GSTQTAQEGSDLTAGYGSTSTAGADSSLIAGYGSTQTAGYNSILTAGYGSTQTAQEGSDL
TSGYGSTSTAGADSSLIAGYGSTQTASYHSSLTAGYGSTQTAREQSVLTTGYGSTSTAGA
DSSLIAGYGSTQTAGYHSILTAGYGSTQTAQERSDLTTGYGSTSTAGADSSLIAGYGSTQ
TAGYNSILTAGYGSTQTAQENSDLTTGYGSTSTAGYDSSLIAGYGSTQTAGYNSILTAGY
GSTQTAQENSDLTTGYGSTSTAGYESSLIAGYGSTQTASFKSTLMAGYGSSQTAREQSSL
TAGYGSTSMAGYDSSLIAGYGSTQTAGYQSTLTAGYGSTQTAEHSSTLTAGYGSTATAGA
DSSLIAGYGSSLTSGIRSFLTAGYGSTLISGLRSVLTAGYGSSLISGRRSSLTAGYGSNQ
IASHRSSLIAGPESTQITGNRSMLIAGKGSSQTAGYRSTLISGADSVQMAGERGKLIAGA
DSTQTAGDRSKLLAGNNSYLTAGDRSKLTAGNDCILMAGDRSKLTAGINSILTAGCRSKL
IGSNGSTLTAGENSVLIFRCWDGKRYTNVVAKTGKGGIEADMPYQMDEDNNIVNKPEE
>P05825
MNKKIHSLALLVNLGIYGVAQAQEPTDTPVSHDDTIVVTAAEQNLQAPGVSTITADEIRK
NPVARDVSKIIRTMPGVNLTGNSTSGQRGNNRQIDIRGMGPENTLILIDGKPVSSRNSVR
QGWRGERDTRGDTSWVPPEMIERIEVLRGPAAARYGNGAAGGVVNIITKKGSGEWHGSWD
AYFNAPEHKEEGATKRTNFSLTGPLGDEFSFRLYGNLDKTQADAWDINQGHQSARAGTYA
TTLPAGREGVINKDINGVVRWDFAPLQSLELEAGYSRQGNLYAGDTQNTNSDSYTRSKYG
DETNRLYRQNYALTWNGGWDNGVTTSNWVQYEHTRNSRIPEGLAGGTEGKFNEKATQDFV
DIDLDDVMLHSEVNLPIDFLVNQTLTLGTEWNQQRMKDLSSNTQALTGTNTGGAIDGVST
TDRSPYSKAEIFSLFAENNMELTDSTIVTPGLRFDHHSIVGNNWSPALNISQGLGDDFTL
KMGIARAYKAPSLYQTNPNYILYSKGQGCYASAGGCYLQGNDDLKAETSINKEIGLEFKR
DGWLAGVTWFRNDYRNKIEAGYVAVGQNAVGTDLYQWDNVPKAVVEGLEGSLNVPVSETV
MWTNNITYMLKSENKTTGDRLSIIPEYTLNSTLSWQAREDLSMQTTFTWYGKQQPKKYNY
KGQPAVGPETKEISPYSIVGLSATWDVTKNVSLTGGVDNLFDKRLWRAGNAQTTGDLAGA
NYIAGAGAYTYNEPGRTWYMSVNTHF
>P0A903
MAYSVQKSRLAKVAGVSLVLLLAACSSDSRYKRQVSGDEAYLEAAPLAELHAPAGMILPV
TSGDYAIPVTNGSGAVGKALDIRPPAQPLALVSGARTQFTGDTASLLVENGRGNTLWPQV
VSVLQAKNYTITQRDDAGQTLTTDWVQWNRLDEDEQYRGRYQISVKPQGYQQAVTVKLLN

LEQAGKPVADAASMQRYSTEMMNVISAGLDKSATDAANAAQNRASTTMDVQSAADDTGLP
MLVVRGPFNVVWQRLPAALEKVGMKVTDSTRSQGNMAVTYKPLSDSDWQELGASDPGLAS
GDYKLQVGDLDNRSSLQFIDPKGHTLTQSQNDALVAVFQAAFSK
>Q03155
MNKAYSIIWSHSRQAWIVASELARGHGFVLAKNTLLVLAVVSTIGNAFAVNISGTVSSGG
TVSSGETQIVYSGRGNSNATVNSGGTQIVNNGGKTTATTVNSSGSQNVGTSGATISTIVN
SGGIQRVSSGGVASATNLSGGAQNIYNLGHASNTVIFSGGNQTIFSGGITDSTNISSGGQ
QRVSSGGVASNTTINSSGAQNILSEEGAISTHISSGGNQYISAGANATETIVNSGGFQRV
NSGAVATGTVLSGGTQNVSSGGSAISTSVYNSGVQTVFAGATVTDTTVNSGGNQNISSGG
IVSETTVNVSGTQNIYSGGSALSANIKGSQIVNSEGTAINTLVSDGGYQHIRNGGIASGT
IVNQSGYVNISSGGYAESTIINSGGTLRVLSDGYARGTILNNSGRENVSNGGVSYNAMIN
TGGNQYIYSDGEATAAIVNTSGFQRINSGGTAPVQNSVVVTRTVSSAAKPFDAEVYSGGK
QTVYLWRGIWYSNFLTAVWSMFPGTASGANVNLSGRLNAFAGNVVGTILNQEGRQYVYSG
ATATSTVGNNEGREYVLSGGITDGTVLNSGGLQAVSSGGKASATVINEGGAQFVYDGGQV
TGTNIKNGGTIRVDSGASALNIALSSGGNLFTSTGATLPELTTMAALSVSQNHASNIVLE
NGGLLRVTSGGTATDTTVNSAGRLRIDDGGTINGTTTINADGIVAGTNIQNDGNFILNLA
ENYDFETELSGSGVLVKDNTGIMTYAGTLTQAQGVNVKNGGIIFDSAVVNADMAVNQNAY
INISDQATINGSVNNNGSIVINNSIINGNITNDADLSFGTAKLLSATVNGSLVNNKNIIL
NPTKESAGNTLTVSNYTGTPGSVISLGGVLEGDNSLTDRLVVKGNTSGQSDIVYVNEDGS
GGQTRDGINIISVEGNSDAEFSLKNRVVAGAYDYTLQKGNESGTDNKGWYLTSHLPTSDT
RQYRPENGSYATNMALANSLFLMDLNERKQFRAMSDNTQPESASVWMKITGGISSGKLND
GQNKTTTNQFINQLGGDIYKFHAEQLGDFTLGIMGGYANAKGKTINYTSNKAARNTLDGY
SVGVYGTWYQNGENATGLFAETWMQYNWFNASVKGDGLEEEKYNLNGLTASAGGGYNLNV
HTWTSPEGITGEFWLQPHLQAVWMGVTPDTHQEDNGTVVQGAGKNNIQTKAGIRASWKVK
STLDKDTGRRFRPYIEANWIHNTHEFGVKMSDDSQLLSGSRNQGEIKTGIEGVITQNLSV
NGGVAYQAGGHGSNAISGALGIKYSF
>P41069
MKQTSFFIPLLGTLLLYGCAGTSTEFECNATTSDTCMTMEQANEKAKKLERSSEAKPVAA
SLPRLAEGNFRTMPVQTVTATTPSGSRPAVTAHPEQKLLAPRPLFTAAREVKTVVPVSSV
TPVTPPRPLRTGEQTAALWIAPYIDNQDVYHQPSSVFFVIKPSAWGKPRIN
>Q05811
MNIRMVLLASAAAFAASTPVLAADAIVAAEPEPVEYVRVCDAYGTGYFYIPGTETCLKIE
GYIRFQVNVGDNPGGDNDSDWDAVTAVRFSSRKSDTEYGPLTGVIVMQFNADNASDQDAI
LDSAYLDVAGFRAGLFYSWWDDGLSGETDDIGSVVTLHNSIRYQYESGTFYAGLSVDELE
DGVYQGTFTPGVIPGTTDFTADDGPNNVGVAFGIGGTAGAFSYQVTGGWDVDNEDGAIRA
MGTVEIGPGTFGLAGVYSSGPNSYYSSAEWAVAAEYAIKATDKLKITPGRWHGHVPEDFD
GLGDAWKVGLTVDYQIVENFYAKASVQYLDPQDGEDSTSGYFACSVRSNHLVDAPGLRIG
STTISF
>P84838
MRLRTALLATTLMAAAPVAANATIITGPYVDLGGGYNLVQNQHGHFSNDPANASMLTKSS
SQYRHDAGFTGFGAVGWGFGNGLRLEAEGLYNYSEINHRAPTAATGVTSGHDQSYGGMLN
VLYDIDLKQFGIDVPVTPFVGVGAGYLWQNVSPTTTRYSNGNVSRLGGTNGGFAYQGIVG
AAYDIPNMPGLQLTAQYRMVGQAFSDGPFTMTSYTNGVGKSVGHAFFDNRFNHQFILGLR
YAFNTAPPPPPPAPVVVPPAPTPARTYLVFFDWDRSDLTARAREIVAEAAQASTHVQTTR

IEVNGYTDNSAAHPGPRGEKYNMGLSIRRAQSVKAELIRDGVPTGAIDIHGYGEQHPLVP
TGPNTREPQNRRVEIILH
>P39180
MKRHLNTCYRLVWNHMTGAFVVASELARARGKRGGVAVALSLAAVTSLPVLAADIVVHPG
ETVNGGTLANHDNQIVFGTTNGMTISTGLEYGPDNEANTGGQWVQDGGTANKTTVTSGGL
QRVNPGGSVSDTVISAGGGQSLQGRAVNTTLNGGEQWMHEGAIATGTVINDKGWQVVKPG
TVATDTVVNTGAEGGPDAENGDTGQFVRGDAVRTTINKNGRQIVRAEGTANTTVVYAGGD
QTVHGHALDTTLNGGYQYVHNGGTASDTVVNSDGWQIVKNGGVAGNTTVNQKGRLQVDAG
GTATNVTLKQGGALVTSTAATVTGINRLGAFSVVEGKADNVVLENGGRLDVLTGHTATNT
RVDDGGTLDVRNGGTATTVSMGNGGVLLADSGAAVSGTRSDGKAFSIGGGQADALMLEKG
SSFTLNAGDTATDTTVNGGLFTARGGTLAGTTTLNNGAILTLSGKTVNNDTLTIREGDAL
LQGGSLTGNGSVEKSGSGTLTVSNTTLTQKAVNLNEGTLTLNDSTVTTDVIAQRGTALKL
TGSTVLNGAIDPTNVTLASGATWNIPDNATVQSVVDDLSHAGQIHFTSTRTGKFVPATLK
VKNLNGQNGTISLRVRPDMAQNNADRLVIDGGRATGKTILNLVNAGNSASGLATSGKGIQ
VVEAINGATTEEGAFVQGNRLQAGAFNYSLNRDSDESWYLRSENAYRAEVPLYASMLTQA
MDYDRIVAGSRSHQTGVNGENNSVRLSIQGGHLGHDNNGGIARGATPESSGSYGFVRLEG
DLMRTEVAGMSVTAGVYGAAGHSSVDVKDDDGSRAGTVRDDAGSLGGYLNLVHTSSGLWA
DIVAQGTRHSMKASSDNNDFRARGWGWLGSLETGLPFSITDNLMLEPQLQYTWQGLSLDD
GKDNAGYVKFGHGSAQHVRAGFRLGSHNDMTFGEGTSSRAPLRDSAKHSVSELPVNWWVQ
PSVIRTFSSRGDMRVGTSTAGSGMTFSPSQNGTSLDLQAGLEARVRENITLGVQAGYAHS
VSGSSAEGYNGQATLNVTF
>Q05202
MNQTISSRAPQKRLAPRLLCVMIGAALGTLSASSWAAAATDSTAENAKKTSATAATAKAE
DSKTNDTITVVGAQETFRAGGNDLIPTYLDGQVANGGRIGFLGQQDARNVPFNVIGYTSK
MIEDQQANSIADVVKNDASVQNVRGYGNPSQNYRIRGYNLDGDDISFGGLFGVLPRQIVS
TSMVERVEVFKGANAFINGISPSGSGVGGMINLEPKRAGDTPLTRVTVDYGSASQVGGAL
DVGRRYGDDDQFGVRVNVLHREGESAIHDQKERTTAVSTGLDYRGDRARTSLDVGYQKQT
IHHMRTDVAIGGATVIPEPPSSTLNYGQSWVYTDMETTFGMLRSEYDVSQNWTVYGSVGA
SRNEETGQYGAPMLTNNNGDATISRLYVPYVADSVAGLGGIRGHFDTGPITHKVNLGYAA
NYRTTKSAWNMSGQEDTNIYNPGVIGFPQTVMGSDSQDPQLTSQVRASGLSLSDTLSMMD
DKVSLMLGVRRQEVTIRNFDSGVPNSAGSLDAMKVTPIYGIMVKPWEKVSLYANHIEALG
PGKSAPYQYNGKPVVNAGQIPGIIHSKQNEIGVKFDNQRYGGTLALFEITRPTGMVDPAT
NVYGFYGEQRNRGIELNVFGEPVFGTRLLASATWLDPKLTKAADSANNGNDAVGVANYQL
VFGGEYDIPVVEGLTATGTVVRSGSQYANEANTLKLKPWTRLDLGVRYTMPMKDTSLTWR
ANIENVTNERYWESVEDSGTYIYQGDPRALKLSVSMDF
>Q70M91
MNTKLTKIISGLFVATAAFQTASAGNITDIKVSSLPNKQKIVKVSFDKEIVNPTGFVTSS
PARIALDFEQTGISMDQQVLEYADPLLSKISAAQNSSRARLVLNLNKPGQYNTEVRGNKV
WIFINESDDTVSAPARPAVKAAPAAPAKQQAAAPSTKSAVSVSEPFTPAKQQAAAPFTES
VVSVSAPFSPAKQQAAASAKQQAAAPAKQQAAAPAKQQAAAPAKQTNIDFRKDGKNAGII
ELAALGFAGQPDISQQHDHIIVTLKNHTLPTTLQRSLDVADFKTPVQKVTLKRLNNDTQL
IITTAGNWELVNKSAAPGYFTFQVLPKKQNLESGGVNNAPKTFTGRKISLDFQDVEIRTI
LQILAKESGMNIVASDSVNGKMTLSLKDVPWDQALDLVMQARNLDMRQQGNIVNIAPRDE
LLAKDKALLQAEKDIADLGALYSQNFQLKYKNVEEFRSILRLDNADTTGNRNTLISGRGS

VLIDPATNTLIVTDTRSVIEKFRKLIDELDVPAQQVMIEARIVEAADGFSRDLGVKFGAT
GKKKLKNDTSAFGWGVNSGFGGDDKWGAETKINLPITAAANSISLVRAISSGALNLELSA
SESLSKTKTLANPRVLTQNRKEAKIESGYEIPFTVTSIANGGSSTNTELKKAVLGLTVTP
NITPDGQIIMTVKINKDSPAQCASGNQTILCISTKNLNTQAMVENGGTLIVGGIYEEDNG
NTLTKVPLLGDIPVIGNLFKTRGKKTDRRELLIFITPRIMGTAGNSLRY
>P14916
MKLTPKELDKLMLHYAGELAKKRKEKGIKLNYVEAVALISAHIMEEARAGKKTAAELMQE
GRTLLKPDDVMDGVASMIHEVGIEAMFPDGTKLVTVHTPIEANGKLVPGELFLKNEDITI
NEGKKAVSVKVKNVGDRPVQIGSHFHFFEVNRCLDFDREKTFGKRLDIASGTAVRFEPGE
EKSVELIDIGGNRRIFGFNALVDRQADNESKKIALHRAKERGFHGAKSDDNYVKTIKE
>Q9JTK4
MNKKHGFSLTLTALAIAAAFPSYAANPETAASDAAQSQSLKEITVRAAKVGRRSKEATGL
GKIVKTSETLNKEQVLGIRDLTRYDPGVAVVEQGNGASGGYSIRGVDKNRVAVSVDGVAQ
IQAFTVQGSLSGYGGRGGSGAINEIEYENISTVEIDKGAGSSDHGSGALGGAVAFRTKEA
ADLISDGKSWGIQAKTAYGSKNRQFMKSLGAGFSKDGWEGLLIRTERQGRETRPHGDIAD
GVEYGIDRLDAFRQTYDIQKQNKKAEYFLAEGEREPKPVAKLAGNGNYLKNQLNRWVEER
KKNNQPLNAEEEAMVREAQARHENLSAQSYTGGGRILPDPMDYRSGSWLAKLGYRFGGRH
YVGGVFEDTKQRYDIRDMTEKQYYGTDEAKKFSNKSGVYDGNDFRDGLYFVPNIEEWKGD
TNLVKGIGLKYSRTKFIDEHHRRRRMGLLYRYENEAYSDNWADKAVLSFDKQGVATDNNT
LKLNCAVYPAVDKSCRASADKPYSYDSSDRFHYREQHNVLNASFEKSLKNKWTKHHLTLG
FGYDASKAISRPEQLSHNAARISESTGFDDNNQDKYLLGKPEVVEGSVCGYIETLRSRKC
VPRKINGSNIHISLNDRFSIGKYFDFSLGGRYDRQNFTTSEELVRSGRYVDRSWNSGIVF
KPNRHFSVSYRASSGFRTPSFQELFGIDIYHDYPKGWQRPALKSEKAANREIGLQWKGDF
GFLEISSFRNRYTDMIAVADHKTQLPDSTGRLTEIDIRDYYNAQNMSLQGVNILGKIDWN
GVYGKLPEGLYTTLAYNRIKPKSVSNRPDLSLRSYALDAVQPSRYVLGFGYDQPEGKWGA
NIMLTYSKGKNPDELAYLAGDQKRYSTKRASSSWSTADVSAYLNLKKRLTLRAAIYNIGN
YRYVTWESLRQTAESTANRHGGDSNYGRYAAPGRNFSLALEMKF
>P20440
MRNFILFPMMAVVLLSGCQQNRPTTLSPAVSGQAQLEQLASVAAGARYLKNKCNRSDLPA
DEAINRAAINVGKKRGWANIDANLLSQRSAQLYQQLQQDSTPEATKCSQFNRQLAPFIDS
LRDNK
>P13972
MYPTDPRQLNTERQIYLDKQFFVDVFSIPACVRNTNGDLSATNEKFSKEFIGSLDIKEWF
YSLPVQVATSFLREELDAMSLPSSMNKIQSVAIGDKLWLVQFIPLIYGEVVNVLWLFFCK
NSNVIVDYCRGLRTNITNDRMLEFKNKSTEIQWKVFILYSFGFCHESIASLLSITNGSSR
NAISEVYKFFGIHSKHDLLMIFHTSRMHSLFFDELFFILKCAE
>P0A924
MRSIARRTAVGAALLLVMPVAVWISGWRWQPGEQSWLLKAAFWVTETVTQPWGVITHLIL
FGWFLWCLRFRIKAAFVLFAILAAAILVGQGVKSWIKDKVQEPRPFVIWLEKTHHIPVDE
FYTLKRAERGNLVKEQLAEEKNIPQYLRSHWQKETGFAFPSGHTMFAASWALLAVGLLWP
RRRTLTIAILLVWATGVMGSRLLLGMHWPRDLVVATLISWALVAVATWLAQRICGPLTPP
AEENREIAQREQES
>P0A927
MKKTLLAAGAVLALSSSFTVNAAENDKPQYLSDWWHQSVNVVGSYHTRFGPQIRNDTYLE

YEAFAKKDWFDFYGYADAPVFFGGNSDAKGIWNHGSPLFMEIEPRFSIDKLTNTDLSFGP
FKEWYFANNYIYDMGRNKDGRQSTWYMGLGTDIDTGLPMSLSMNVYAKYQWQNYGAANEN
EWDGYRFKIKYFVPITDLWGGQLSYIGFTNFDWGSDLGDDSGNAINGIKTRTNNSIASSH
ILALNYDHWHYSVVARYWHDGGQWNDDAELNFGNGNFNVRSTGWGGYLVVGYNF
>Q7VNU1
MKMKKQCATLTFFIGLHGYTIAEDNPKNISLSVITVPGHHERQPDRSIITQNEIDQKQSD
NVADLVNTVPGVSMAGGFRPSGQTLNIRGMGDTEDIRVQVDGATKNFEKYQQGSLFIEPE
LLRRVSIDKGNHYPQYGNGGFAGTIKLETKNAKDFLQENQLLGGLLKYGYNTNNNQRTFS
GAIFMQNDQKNIDALVYATVRRAHDYKRADKTPIKYSANNQANFLAKVNWWLTPSQLLAF
SKVHGNHNGWEPFAAKRDLLPGPTEAEITKYGLDLAWKRKLVAREQQDRSYSLQYQFLPE
NNPWINTVAQLSHSSTYQHDTRSEQASKTYLASLGNESWTRYTDLTFDVNNTSLFNVAKT
SHTLLVGLQWVKHKRQTLIFDPSKLQKAEYNHGYFQPSYMPSGHQYTHAFYAQDKIKIHN
LTVSIGARYDYVKNIGKPNIATIYNDPTAGHDYSSKHYPGWSSYLGLNYKLTPYLNLFSN
ISNTWRAPVIDEQYETQYAKATLSPTASSLDLKKERITQLRVGKQIHFDHILSNNDQLSF
NSTFFYYKGKDEIFKTRGVRCFESAQNNNNEVCSKKIGNYRNLPGYQIKGFELEANYDST
YWFTNLSYSHTIGKRLASPRNPWLASTSWIAEIPPRKAVVTLGSHIPDTNLTLGWKSEFV
RRQDRSPTDQDKDAGHWALPKSSGYALHGIFATWQPKQIKHLRIQFTVDNLLNRSYRPYL
SELAAGTGRNIKLSISKQF
>P14789
MQHKRSRAMASPRSPFLFVLLALAVGGTANAHDDGLPAFRYSAELLGQLQLPSVALPLND
DLFLYGRDAEAFDLEAYLALNAPALRDKSEYLEHWSGYYSINPKVLLTLMVMQSGPLGAP
DERALAAPLGRLSAKRGFDAQVRDVLQQLSRRYYGFEEYQLRQAAARKAVGEDGLNAASA
ALLGLLREGAKVSAVQGGNPLGAYAQTFQRLFGTPAAELLQPSNRVARQLQAKAALAPPS
NLMQLPWRQGYSWQPNGAHSNTGSGYPYSSFDASYDWPRWGSATYSVVAAHAGTVRVLSR
CQVRVTHPSGWATNYYHMDQIQVSNGQQVSADTKLGVYAGNINTALCEGGSSTGPHLHFS
LLYNGAFVSLQGASFGPYRINVGTSNYDNDCRRYYFYNQSAGTTHCAFRPLYNPGLAL
>Q51487
MKRSFLSLAVAAVVLSGCSLIPDYQRPEAPVAAAYPQGQAYGQNTGAAAVPAADIGWREF
FRDPQLQQLIGVALENNRDLRVAALNVEAFRAQYRIQRADLFPRIGVDGSGTRQRLPGDL
STTGSPAISSQYGVTLGTTAWELDLFGRLRSLRDQALEQYLATEQAQRSAQTTLVASVAT
AYLTLKADQAQLQLTKDTLGTYQKSFDLTQRSYDVGVASALDLRQAQTAVEGARATLAQY
TRLVAQDQNALVLLLGSGIPANLPQGLGLDQTLLTEVPAGLPSDLLQRRPDILEAEHQLM
AANASIGAARAAFFPSISLTANAGTMSRQLSGLFDAGSGSWLFQPSINLPIFTAGSLRAS
LDYAKIQKDINVAQYEKAIQTAFQEVADGLAARGTFTEQLQAQRDLVKASDEYYQLADKR
YRTGVDNYLTLLDAQRSLFTAQQQLITDRLNQLTSEVNLYKALGGGWNQQTVTQQQTAKK
EDPQA
>P22340
MYRKSTLAMLIALLTSAASAHAQTDISTIEARLNALEKRLQEAENRAQTAENRAGAAEKK
VQQLTAQQQKNQNSTQEVAQRTARLEKKADDKSGFEFHGYARSGVIMNDSGASTKSGAYI
TPAGETGGAIGRLGNQADTYVEMNLEHKQTLDNGATTRFKVMVADGQTSYNDWTASTSDL
NVRQAFVELGNLPTFAGPFKGSTLWAGKRFDRDNFDIHWIDSDVVFLAGTGGGIYDVKWN
DGLRSNFSLYGRNFGDIDDSSNSVQNYILTMNHFAGPLQMMVSGLRAKDNDERKDSNGNL
AKGDAANTGVHALLGLHNDSFYGLRDGSSKTALLYGHGLGAEVKGIGSDGALRPGADTWR
IASYGTTPLSENWSVAPAMLAQRSKDRYADGDSYQWATFNLRLIQAINQNFALAYEGSYQ

YMDLKPEGYNDRQAVNGSFYKLTFAPTFKVGSIGDFFSRPEIRFYTSWMDWSKKLNNYAS
DDALGSDGFNSGGEWSFGVQMETWF
>P0A263
MKVKVLSLLVPALLVAGAANAAEIYNKDGNKLDLFGKVDGLHYFSDDKGSDGDQTYMRIG
FKGETQVNDQLTGYGQWEYQIQGNQTEGSNDSWTRVAFAGLKFADAGSFDYGRNYGVTYD
VTSWTDVLPEFGGDTYGADNFMQQRGNGYATYRNTDFFGLVDGLDFALQYQGKNGSVSGE
NTNGRSLLNQNGDGYGGSLTYAIGEGFSVGGAITTSKRTADQNNTANARLYGNGDRATVY
TGGLKYDANNIYLAAQYSQTYNATRFGTSNGSNPSTSYGFANKAQNFEVVAQYQFDFGLR
PSVAYLQSKGKDISNGYGASYGDQDIVKYVDVGATYYFNKNMSTYVDYKINLLDKNDFTR
DAGINTDDIVALGLVYQF
>P77774
MQLRKLLLPGLLSVTLLSGCSLFNSEEDVVKMSPLPTVENQFTPTTAWSTSVGSGIGNFY
SNLHPALADNVVYAADRAGLVKALNADDGKEIWSVSLAEKDGWFSKEPALLSGGVTVSGG
HVYIGSEKAQVYALNTSDGTVAWQTKVAGEALSRPVVSDGLVLIHTSNGQLQALNEADGA
VKWTVNLDMPSLSLRGESAPTTAFGAAVVGGDNGRVSAVLMEQGQMIWQQRISQATGSTE
IDRLSDVDTTPVVVNGVVFALAYNGNLTALDLRSGQIMWKRELGSVNDFIVDGNRIYLVD
QNDRVMALTIDGGVTLWTQSDLLHRLLTSPVLYNGNLVVGDSEGYLHWINVEDGRFVAQQ
KVDSSGFQTEPVAADGKLLIQAKDGTVYSITR
>P02930
MKKLLPILIGLSLSGFSSLSQAENLMQVYQQARLSNPELRKSAADRDAAFEKINEARSPL
LPQLGLGADYTYSNGYRDANGINSNATSASLQLTQSIFDMSKWRALTLQEKAAGIQDVTY
QTDQQTLILNTATAYFNVLNAIDVLSYTQAQKEAIYRQLDQTTQRFNVGLVAITDVQNAR
AQYDTVLANEVTARNNLDNAVEQLRQITGNYYPELAALNVENFKTDKPQPVNALLKEAEK
RNLSLLQARLSQDLAREQIRQAQDGHLPTLDLTASTGISDTSYSGSKTRGAAGTQYDDSN
MGQNKVGLSFSLPIYQGGMVNSQVKQAQYNFVGASEQLESAHRSVVQTVRSSFNNINASI
SSINAYKQAVVSAQSSLDAMEAGYSVGTRTIVDVLDATTTLYNAKQELANARYNYLINQL
NIKSALGTLNEQDLLALNNALSKPVSTNPENVAPQTPEQNAIADGYAPDSPAPVVQQTSA
RTTTSNGHNPFRN
>P29842
MAAKDVQFGNEVRQKMVNGVNILANAVRVTLGPKGRNVVVDRAFGGPHITKDGVTVAKEI
ELKDKFENMGAQMVKEVASKTNDVAGDGTTTATVLAQSIVAEGIKAVTAGMNPTDLKRGI
DKAVAALVEELKNIAKPCDTSKEIAQVGSISANSDEQVGAIIAEAMEKVGKEGVITVEDG
KSLENELDVVEGMQFDRGYLSPYFINDAEKQIAGLDNPFVLLFDKKISNIRDLLPVLEQV
AKASRPLLIIAEDVEGEALATLVVNNIRGVLKTVAVKAPGFGDRRKAMLQDIAILTGAVV
ISEEVGLSLEKATLDDLGQAKRIEIGKENTTVIDGFGDAAQIEARVAEIRQQIETATSDY
DKEKLQERVAKLAGGVAVIKVGAATEVEMKEKKDRVEDALHATRAAVEEGVVAGGGVALL
RARAALENLHTGNADQDAGVQIVLRAVESPLRQIVANAGGEPSVVVNKVLEGKGNYGYNA
GSGEYGDMIGMGVLDPAKVTRSALQHAASIAGLMLTTDCMIAEIPEEKPAVPDMGGMGGM
GGMM
>P31499
MPRSTSDRFRWSPLSLAIACTLSLAVQAADTSSTQTNSKKRIADTMVVTATGNERSSFEA
PMMVTVVEADTPTSETATSATDMLRNIPGLTVTGSGRVNGQDVTLRGYGKQGVLTLVDGI
RQGTDTGHLNSTFLDPALVKRVEIVRGPSALLYGSGALGGVISYETVDAADLLLPGQNSG
YRVYSAAATGDHSFGLGASAFGRTDDVDGILSFGTRDIGNIRQSDGFNAPNDETISNVLA

KGTWRIDQIQSLSANLRYYNNSALEPKNPQTSAASSTNLMTDRSTIQRDAQLKYNIKPLD
QEWLNATAQVYYSEVEINARPQGTPEEGRKQTTKGGKLENRTRLFTDSFASHLLTYGTEA
YKQEQTPSGATESFPQADIRFGSGWLQDEITLRDLPVSILAGTRYDNYRGSSEGYADVDA
DKWSSRGAVSVTPTDWLMLFGSYAQAFRAPTMGEMYNDSKHFSMNIMGNTLTNYWVPNPN
LKPETNETQEYGFGLRFNDLMMAEDDLQFKASYFDTNAKDYISTGVTMDFGFGPGGLYCK
NCSTYSTNIDRAKIWGWDATMTYQTQWFNLGLAYNRTRGKNQNTNEWLDTINPDTVTSTL
DVPVANSGFAVGWIGTFADRSSRVSSSGTPQAGYGVNDFYVSYKGQEQFKGMTTTVVLGN
AFDKGYYGPQGVPQDGRNAKFFVSYQW
>P31631
MYKIKHSFNKTLIAISISSFLSIAYATESIENPQPIIQLSESLSSKYSGKGVKLGVMDEG
FMVKHPRHSSHLHPLIHQLTTPEGEVRIYDASYPQFEVNPVEKEDGIDLIPSLETHGAGV
AGIIAAQADKTLGDGYSGGIAKGAELYVATKSYKRTLEKVIQDAKKELENAKDEEDEKTP
SLDQMAKNDLLASKEKEMAIERAEWASGLNKLLDNNVFAINNSWNPFSISDDINVVDKFY
QSIKQNKHNPLLQAIMRAKNSNTLLVFAAGNESKKQPGVMALLPRYFPELEKNLISAVAV
DKEQKIASYSNHCGASKNWCVAAPGDLHVLIGVADEHKKPQYGLTKEQGTSFSAPAITAS
LAVLKERFDYLTATQIRDTLLTTATDLGEKGVDNVYGWGLINLKKAVNGPTQFLNDETIT
VTRDDHWSNPLASQFKITKKGDKSLHLDGENHLDTVAVEEGRLALNGKTKVKTISNHANL
AVNGTEVEQNYSSSGQSQLEVLGKSGLIANAQANIHLAGSLKIDDKLTEKTEAGDVSATV
VQLKDKATYQGGFTQLVENENLAKRGLIQDLYFKESEIIAKVNKPLTDEKADTNGQAGLA
LLNALRTTPIAYRRSWYNGWLQSALEQRKLDNLHYAVSNNIYADSLELLRSQNRKGLTQA
QQHLFTAYHTPLQTTVWAEHLNQKQSASSKHTDVKHHQSQLGVNHKLADKTVLSATLSQQ
KNRLEKPFAQATLKQTALNIGLRYHLDNAWFSEATLQFARQKYQQSRRFASHQLGTAETR
GSTLGGEMRIGYQFMPNQWIIEPSLGVQWIQTKMNGLNESGELATQTAAMRYRDVNIVPS
VKLQRTFQLEQGSISPYIGLNYLHRLNGKITKITSNIAGKTLHSEATTKRNRQLNGEVGV
KLHYKNWFTAMNLDYSRVKSCKPIWLESKCWL
>P12375
MTVLNRLHVCSLLAVSSLGMLPVGVFAAEAAMPGVDHSQMQGMDHSKMQGMDHSQMQGMD
HSKMQGMDHSQMQGMDSDMTTMAPSKPAAPTQSRTPIAPVTDANRAAVYRSAKGHTVHDE
AANYFLLFDQLEWQDADNGSVLNWDVNGWVGGDIDRLWIRSEGERTNGKTESAELQALWG
HAISPWWDLVGGVRQDFKPGSPQTWAAFGLQGLALYNFEAEATAFLGEGGQTGLRLEGDY
DILLTNRLILQPTAEVNFYGQSDPQRGIGSGLSETEVGVRLRYEIRREFAPYIGVTWNRS
YGNTADFAREEGEDRSEARLVLGVRMWF
>P69411
MRALPICLVALMLSGCSMLSRSPVEPVQSTAPQPKAEPAKPKAPRATPVRIYTNAEELVG
KPFRDLGEVSGDSCQASNQDSPPSIPTARKRMQINASKMKANAVLLHSCEVTSGTPGCYR
QAVCIGSALNITAK
>P09169
MRAKLLGIVLTTPIAISSFASTETLSFTPDNINADISLGTLSGKTKERVYLAEEGGRKVS
QLDWKFNNAAIIKGAINWDLMPQISIGAAGWTTLGSRGGNMVDQDWMDSSNPGTWTDESR
HPDTQLNYANEFDLNIKGWLLNEPNYRLGLMAGYQESRYSFTARGGSYIYSSEEGFRDDI
GSFPNGERAIGYKQRFKMPYIGLTGSYRYEDFELGGTFKYSGWVESSDNDEHYDPGKRIT
YRSKVKDQNYYSVAVNAGYYVTPNAKVYVEGAWNRVTNKKGNTSLYDHNNNTSDYSKNGA
GIENYNFITTAGLKYTF
>Q9S142

MKLGRYSLFLLCPLLASCSGNGFYKDNLGVIDKNILHADTSLLKSKNKEHYKSSDMVSKT
DSIYIGNSSFQTYHGEPLPGKLEGVHGIILRSSTPLGFDEVLSMIQDSSGIPIVKHTTKD
VISGGVSSKSLAATVAEKMNSATGGKSTDQFDHLLLEVSSEHQLMDVNYQGALSTFLDKV
AANYNLYWTYESGRIAFSNEETKRFSISILPGGKYTSKNSISSDSNSSSGSSGSSGSSSS
DSGAELKFDSDVDFWKDIENSIKLILGSDGSYSISTSTSSVIVRTSSANMKKINEYINTL
NAQLERQVTIDVAIYNVTTTDSSDLAMSLEALLKHNGGVLGSVSTSNFAATSGTPSFTGY
LNGNGDSSNQVLLNLLAEKGKVSVVTSASVTTMSGQPVPLKVGNDRTYVSEIGTVLSQSS
TSTTASTSTVTSGFLMNLLPQVADDGNILLQYGVTLSELVGSNNGFDQATVNGTVIQLPN
VDSTTFVQSSMLRNGNTLVLAGYEKKRNESVDQGVGTTSFKLLGGALNGSASRTVTVICI
TPRIIDLKASGE
>P18149
MKKIIKLSLLSLSIAGLASCSTLGLGGSDDAKASAKDTAAAQTATTEQAAAVSKPTAKVS
LNKLGQDKIKATVYTAYNNNPQGSVRLQWQAPEGSKCHDTSFPITKYAEKNDKTWATVTV
KQGNNFCSGKWTANVVYDKEVIASDSINI
>P80604
MKNKSKLLACCLMALPISSFSIGNNNLIGVGVSAGNSIYQVKKKTAVEPFLMLDLSFGNF
YMRGAAGLSELGYQHVFTPSFSTSLFLSPFDGAPIKRKDLKPGYDSIQDRKTQVAVGLGL
DYDLSDLFNLPNTNISLEMKKGRRGFNSDITLTRTFMLTDKLSISPSFGLSYYSAKYTNY
YFGIKKAELNKTKLKSVYHPKKAYSGHIALNSHYAITDHIGMGLSFSWETYSKAIKKSPI
VKRSGEISSALNFYYMF
>P69973
MKVKTSLSTLILILFLTGCKVDLYTGISQKEGNEMLALLRQEGLSADKEPDKDGKIKLLV
EESDVAQAIDILKRKGYPHESFSTLQDVFPKDGLISSPIEELARLNYAKAQEISRTLSEI
DGVLVARVHVVLPEEQNNKGKKGVAASASVFIKHAADIQFDTYIPQIKQLVNNSIEGLAY
DRISVILVPSVDVRQSSHLPRNTSILSIQVSEESKGHLIGLLSLLILLLPVTNLAQYFWL
QRKK
>P10641
MKKFNQSLLATAMLLAAGGANAAAFQLAEVSTSGLGRAYAGEAAIADNASVVATNPALMS
LFKTAQFSTGGVYIDSRINMNGDVTSYAQIITNQIGMKAIKDGSASQRNVVPGAFVPNLY
FVAPVNDKFALGAGMNVNFGLKSEYDDSYDAGVFGGKTDLSAINLNLSGAYRVTEGLSLG
LGVNAVYAKAQVERNAGLIADSVKDNQITSALSTQQEPFRDLKKYLPSKDKSVVSLQDRA
AWGFGWNAGVMYQFNEANRIGLAYHSKVDIDFADRTATSLEANVIKEGKKGNLTFTLPDY
LELSGFHQLTDKLAVHYSYKYTHWSRLTKLHASFEDGKKAFDKELQYSNNSRVALGASYN
LYEKLTLRAGIAYDQAASRHHRSAAIPDTDRTWYSLGATYKFTPNLSVDLGYAYLKGKKV
HFKEVKTIGDKRTLTLNTTANYTSQAHANLYGLNLNYSF
>P37423
MKKFYSCLPVFLLIGCAQVPLPSSVSKPVQQPGAQKEQLANANSIDECQSLPYVPSDLAK
NKSLSNHNADNSASKNSAISSSIFCEKYKQTKEQALTFFQEHPQYMRSKEDEEQLMTEFK
KVLLEPGSKNLSIYQTLLAAHERLQAL
>P57041
MRKKLTALVLSALPLAAVADVSLYGEIKAGVEGRNYQLQLTEAQAANGGASGQVKVTKVT
KAKSRIRTKISDFGSFIGFKGSEDLGEGLKAVWQLEQDVSVAGGGATQWGNRESFIGLAG
EFGTLRAGRVANQFDDASQAIDPWDSNNDVASQLGIFKRHDDMPVSVRYDSPEFSGFSGS
VQFVPAQNSKSAYKPAYWTTVNTGSATTTTFVPAVVGKPGSDVYYAGLNYKNGGFAGNYA

FKYARHANVGRDAFELFLLGSGSDQAKGTDPLKNHQVHRLTGGYEEGGLNLALAAQLDLS
ENGDKTKNSTTEIAATASYRFGNAVPRISYAHGFDFIERGKKGENTSYDQIIAGVDYDFS
KRTSAIVSGAWLKRNTGIGNYTQINAASVGLRHKF
>P0A901
MRLLPLVAAATAAFLVVACSSPTPPRGVTVVNNFDAKRYLGTWYEIARFDHRFERGLEKV
TATYSLRDDGGLNVINKGYNPDRGMWQQSEGKAYFTGAPTRAALKVSFFGPFYGGYNVIA
LDREYRHALVCGPDRDYLWILSRTPTISDEVKQEMLAVATREGFDVSKFIWVQQPGS
>P61320
MPLPDFRLIRLLPLAALVLTACSVTTPKGPGKSPDSPQWRQHQQDVRNLNQYQTRGAFAY
ISDQQKVYARFFWQQTGQDRYRLLLTNPLGSTELELNAQPGNVQLVDNKGQRYTADDAEE
MIGKLTGMPIPLNSLRQWILGLPGDATDYKLDDQYRLSEITYSQNGKNWKVVYGGYDTKT
QPAMPANMELTDGGQRIKLKMDNWIVK
>P38399
MKKTAIALAVALVGFATVAQAAPKDNTWYTGGKLGWSQYQDTGSIINNDGPTHKDQLGAG
AFFGYQANQYLGFEMGYDWLGRMPYKGDINNGAFKAQGVQLAAKLSYPVAQDLDVYTRLG
GLVWRADAKGSFDGGLDRASGHDTGVSPLVALGAEYAWTKNWATRMEYQWVNNIGDRETV
GARPDNGLLSVGVSYRFGQEDAAAPIVAPTPAPAPIVDTKRFTLKSDVLFGFNKANLKPE
GQQALDQLYAQLSSIDPKDGSVVVLGFADRIGQPAPNLALSQRRADSVRDYLVSKGIPAD
KITARGEGQANPVTGNTCDNVKPRAALIECLAPDRRVEIEVKGYKEVVTQPQA
>P15320
MKNNNFRLSAAGKLAAALAIILAASAGAYAAEIVAANGANGPGVSTAATGAQVVDIVAPN
GNGLSHNQYQDFNVNQPGAVLNNSREAGLSQLAGQLGANPNLGGREASVILNEVIGRNPS
LLHGQQEIFGMAADYVLANPNGISCQSCGFINTSHSSLVVGNPLVENGVLQGYSTFGNRN
TLSLNGTLNAGGVLDLIAPKIDSRGEVIVQDFKQSNGKVTSAAINAISGLNRVARDGTVQ
ASQQMPTALDSYYLGSMQAGRINIINTAQGSGVKLAGSLNAGDELKVKAYDIRSESRVDD
ASSNKNGGDNYQNYRGGIYVNDRSSSQTLTRTELKGKNISLVADNHAHLTATDIRGEDIT
LQGGKLTLDGQQLKQTQGHTDDRWFYSWQYDVTREREQLQQAGSTVAASGSAKLISTQED
VKLLGANVSADRALSVKAARDVHLAGLVEKDKSSERGYQRNHTSSLRTGRWSNSDESESL
KASELRSEGELTLKAGRNVSTQGAKVHAQRDLTIDADNQIQVGVQKTANAKAVRDDKTSW
GGIGGGDNKNNSNRREISHASELTSGGTLRLNGQQGVTITGSKARGQKGGEVTATHGGLR
IDNALSTTVDKIDARTGTAFNITSSSHKADNSYQSSTASELKSDTNLTLVSHKDADVIGS
QVASGGELSVESKTGNINVKAAERQQNIDEQKTALTVNGYAKEAGDKQYRAGLRIEHTRD
SEKTTRTENSASSLSGGSVKLKAEKDVTFSGSKLVADKGDASVSGNKVSFLAADDKTASN
TEQTKIGGGFYYTGGIDKLGSGVEAGYENNKTQAQSSKAITSGSDVKGNLTINARDKLTQ
QGAQHSVGGAYQENAAGVDHLAAADTASTTTTKTDVGVNIGANVDYSAVTRPVERAVGKA
AKLDATGVINDIGGIGAPNVGLDIGAQGGSSEKRSSSSQAVVSSVQAGSIDINAKGEVRD
QGTQYQASKGAVNLTADSHRSEAAANRQDEQSRDTRGSAGVRVYTTTGSDLTVDAKGEGG
TQRSNSSASQAVTGSIDAANGINVNVKKDAIYQGTALNGGRGKTAVNAGGDIRLDQASDK
QSESRSGFNVKASAKGGFTADSKNFGAGFGGGTHNGESSSSTAQVGNISGQQGVELKAGR
DLTLQGTDVKSQGDVSLSAGNKVALQAAESTQTRKESKLSGNIDLGAGSSDSKEKTGGNL
SAGGAFDIAKVNESATERQGATIASDGKVTLSANGKGDDALHLQGAKVSGGSAALEAKNG
GILLESAKNEQHKDNWSLGIKANAKGGQTFNKDAGGKVDPNTGKDTHTLGAGLKVGVEQQ
DKTTHANTGITAGDVTLNSGKDTRLAGARVDADSVQGKVGGDLHVESRKDVENGVKVDVD
AGLSHSNDPGSSITSKLSKVGTPRYAGKVKEKLEAGVNKVADATTDKYNSVARRLDPQQD

TTGAVSFSKAEGKVTLPATPAGEKPQGPLWDRGARTVGGAVKDSITGPAGRQGHLKVNAD
VVNNNAVGEQSAIAGKNGVALQVGGQTQLTGGEIRSQQGKVELGGSQVSQQDVNGQRYQG
GGRVDAAATVGGLLGGAAKQSVAGNVPFASGHASTQQADAKAGVFSGK
>P0C2M9
MKMTRLYPLALGGLLLPAIANAQTSQQDESTLEVTASKQSSRSASANNVSSTVVSAPELS
DAGVTASDKLPRVLPGLNIENSGNMLFSTISLRGVSSAQDFYNPAVTLYVDGVPQLSTNT
IQALTDVQSVELLRGPQGTLYGKSAQGGIINIVTQQPDSTPRGYIEGGVSSRDSYRSKFN
LSGPIQDGLLYGSVTLLRQVDDGDMINPATGSDDLGGTRASIGNVKLRLAPDDQPWEMGF
AASRECTRATQDAYVGWNDIKGRKLSLSDGSPDPYMRRCTDSQTLSGKYTTDDWVFNLIS
AWQQQHYSRTFPSGSLIVNMPQRWNQDVQELRAATLGDARTVDMVFGLYRQNTREKLNSA
YNMPTMPYLSSTGYTTAETLAAYSDLTWHLTDRFDIGGGVRFSHDKSSTQYHGSMLGNPF
GDQGKSNDDQVLGQLSAGYMLTDDWRVYTRIAQGYKPSGYNIVPTAGLDAKPFVAEKSIN
YELGTRYETADVTLQAATFYTHTKDMQLYSGPVGMQTLSNAGKADATGVELEAKWRFAPG
WSWDINGNVIRSEFTNDSELYHGNRVPFVPRYGAGSSVNGVIDTRYGALMPRLAVNLVGP
HYFDGDNQLRQGTYATLDSSLGWQATERINISVHVDNLFDRRYRTYGYMNGSSAVAQVNM
GRTVGINTRIDFF
>P16945
MTTLHNLSYGNTPLRNEHPEIASSQIVNQTLGQFRGESVQIVSGTLQSIADMAEEVTFVF
SERKELSLDKRKLSDSQARVSDVEEQVNQYLSKVPELKQKQNVSELLSLLSNSPNISLSQ
LKAYLEGKSEEPSEQFKMLCGLRDALKGRPELAHLLHLVEQALVSMVEEQEEAIVLGARI
TPEAYRESQSGVNPLQPLRDTYRDAVMGYQGINAIWSDLQKRFPNGDIDSVILFLQKALS
ADLQSQQSGSEREKLEIVISDLQKLKEFRSVSDQVKGFWQLFSEGITNGLRPF
>Q8K9A7
MLNSCINKIKKKKNIKKIILMLIFIFSVNFFTKNTNQGKQYEDKLNKDFTNIKKINIKNK
LVNQKEFLLQLEKIKIFSPNLYSKNISIYNAILKWLKKRAEINELNKFRIKLFQMKGVDQ
YGNVKITGYYTPIVKASKIKKNNFIYPIYRTPSNFKKNEKLPQRKDIYNGFLKKEYILAY
SDSLINNFIMEIQGSGFIDYGDNKPLIFFGYAKKNNWPYTSIGQILIKNGDIQKKNISMN
TIKNWCTHHTQKEIQNLLEKNKSFVFFQETKRKEVYGSSAVPLVEKAAIAVDKSVIKIGS
VVLVKIPVLDKNGIFIHKYEMHLLIALDVGGVIKGQHFDVYQGIGEKAGKLAGFYNHYGY
AWVLKI
>P24328
MNMSLSRIVKAAPLRRTTLAMALGALGAAPAAYADWNNQSIIKAGERQHGIHIKQSDGAG
VRTATGTTIKVSGRQAQGVLLENPAAELRFQNGSVTSSGQLFDEGVRRFLGTVTVKAGKL
VADHATLANVSDTRDDDGIALYVAGEQAQASIADSTLQGAGGVRVERGANVTVQRSTIVD
GGLHIGTLQPLQPEDLPPSRVVLGDTSVTAVPASGAPAAVFVFGANELTVDGGHITGGRA
AGVAAMDGAIVHLQRATIRRGDAPAGGAVPGGAVPGGAVPGGFGPLLDGWYGVDVSDSTV
DLAQSIVEAPQLGAAIRAGRGARVTVSGGSLSAPHGNVIETGGGARRFPPPASPLSITLQ
AGARAQGRALLYRVLPEPVKLTLAGGAQGQGDIVATELPPIPGASSGPLDVALASQARWT
GATRAVDSLSIDNATWVMTDNSNVGALRLASDGSVDFQQPAEAGRFKVLMVDTLAGSGLF
RMNVFADLGLSDKLVVMRDASGQHRLWVRNSGSEPASGNTMLLVQTPRGSAATFTLANKD
GKVDIGTYRYRLAANGNGQWSLVGAKAPPAPKPAPQPGPQPGPQPPQPPQPPQPPQPPQP
PQRQPEAPAPQPPAGRELSAAANAAVNTGGVGLASTLWYAESNALSKRLGELRLNPDAGG
AWGRGFAQRQQLDNRAGRRFDQKVAGFELGADHAVAVAGGRWHLGGLAGYTRGDRGFTGD
GGGHTDSVHVGGYATYIANSGFYLDATLRASRLENDFKVAGSDGYAVKGKYRTHGVGVSL

EAGRRFAHADGWFLEPQAELAVFRVGGGAYRAANGLRVRDEGGSSVLGRLGLEVGKRIEL
AGGRQVQPYIKASVLQEFDGAGTVRTNGIAHRTELRGTRAELGLGMAAALGRGHSLYASY
EYSKGPKLAMPWTFHAGYRYSW
>P0A3P1
MGISKASLLSLAAAGIVLAGCQSSRLGNLDNVSPPPPPAPVNAVPAGTVQKGNLDSPTQF
PNAPSTDMSAQSGTQVASLPPASAPDLTPGAVAGVWNASLGGQSCKIATPQTKYGQGYRA
GPLRCPGELANLASWAVNGKQLVLYDANGGTVASLYSSGQGRFDGQTTGGQAVTLSR
>Q51485
MYKNKKTRPAARTVGCLFALGALGLGSAAHAAEAFSPNSKWMLGDWGGKRTELLEKGYDF
KLEYVGEAAANLDGGYDDDKTGRYTDQFALGVHMDLEKILGWKATEFQFTVTERNGKNLS
NDRIGDPRAGHISSVQEVWGRGQTWRLTQLWLKQQYFDGALDVKFGRFGEGEDFNSFPCD
FQNLAFCGSQVGNWAGSIWYNWPVSQWALRVKYNFAPDWYVQVGAYEQNPSNLETGNGFK
MSGSGTKGALLPVELIWQPKVGAEQLPGEYRLGYYYSTAKADDVYDDVDGQPQGLTGNDF
KSRGSKHGWWVVAQQQVTSHNGDASRGLSLFANLTVHDKATNVVDNYQQLGVVYKGPFDA
RPKDDIGLGIARIHVNDDVKKRQRLVNQVNGIDDYDNPLYQPLQDTEYNAELYYGVHVTD
WLTVRPNLQYIKQPGGVDEVDNALVAGIKIQTVF
>Q9K187
MARLFSLKPLVLALGLCFGTHCAAADAVAAEETDNPTAGESVRSVSEPIQPTSLSLGSTC
LFCSNESGSPERTEAAVQGSGEASIPEDYTRIVADRMEGQSQVQVRAEGNVVVERNRTTL
NTDWADYDQSGDTVTAGDRFALQQDGTLIRGETLTYNLEQQTGEAHNVRMEIEQGGRRLQ
SVSRTAEMLGEGHYKLTETQFNTCSAGDAGWYVKAASVEADREKGIGVAKHAAFVFGGVP
IFYTPWADFPLDGNRKSGLLVPSLSAGSDGVSLSVPYYFNLAPNLDATFAPSVIGERGAV
FDGQVRYLRPDYAGQSDLTWLPHDKKSGRNNRYQAKWQHRHDISDTLQAGVDFNQVSDSG
YYRDFYGNKEIAGNVNLNRRVWLDYGGRAAGGSLNAGLSVLKYQTLANQSGYKDKPYALM
PRLSVEWRKNTGRAQIGVSAQFTRFSHDSRQDGSRLVVYPDIKWDFSNSWGYVRPKLGLH
ATYYSLNRFGSQEARRVSRTLPIVNIDSGATFERNTRMFGGEVLQTLEPRLFYNYIPAKS
QNDLPNFDSSESSFGYGQLFRENLYYGNDRINTANSLSAAVQSRILDGATGEERFRAGIG
QKFYFKDDAVMLDGSVGKKPRNRSDWVAFASGSIGSRFILDSSIHYNQNDKRAENYAVGA
SYRPAQGKVLNARYKYGRNEKIYLKSDGSYFYDKLSQLDLSAQWPLTRNLSAVVRYNYGF
EAKKPIEVLAGAEYKSSCGCWGAGVYAQRYVTGENTYKNAVFFSLQLKDLSSVGRNPADR
MDVAVPGYITAHSLSAGRNKRP
>P10325
MKKTNMALALLVAFSVTGCANTDIFSGDVYSASQAKEARSITYGTIVSVRPVKIQADNQG
VVGTLGGGALGGIAGSTIGGGRGQAIAAVVGAIGGAIAGSKIEEKMSQVNGAELVIKKDD
GQEIVVVQKADSSFCSLVAEFVFVGGGSSLNVSVL
>P0A221
MSEAKNSNLAPFRLLVKLTNGVGDEFPLYYGNNLIVLGRTIETLEFGNDNFPENIIPVTD
SKSDGIIYLTISKDNICQFSDEKGEQIDINSQFNSFEYDGISFHLKNMREDKSRGHILNG
MYKNHSVFFFFAVIVVLIIIFSLSLKKDEVKEIAEIIDDKRYGIVNTGQCNYILAETQND
AVWASVALNKTGFTKCRYILVSNKEINRIQQYINQRFPFINLYVLNLVSDKAELLVFLSK
ERNSSKDTELDKLKNALIVEFPYIKNIKFNYLSDHNARGDAKGIFTKVNVQYKEICENNK
VTYSVREELTDEKLELINRLISEHKNIYGDQYIEFSVLLIDDDFKGKSYLNSKDSYVMLN
DKHWFFLDKNK
>P15492

MIINKFSLKWMLAIAVAIPAIALLFVAFTSLNTMSVMQAQSNSLYANTAAPMRAMAEATS
RIPRMRVGIDMMLLQETALKDAKGVLKRVEEARTEDIPEMRQAMQVAVDSQVNPELKEQA
RKLQARFEQMVREELEPMLQAFANNDMTTAQNIYRDKYAPTYGEMRKQANQILDTLLQQA
EQQNHASVESFEAGRTKQMVIIAAGLIISFITSLVIITNLRSRVAYLKDRMSSAAANLSL
RTRLELDGNDELCDIGKSFNAFIDKVHHSIEEVAENSKELATMASSVSQRAHMTQSNCAS
QRDRTVQVATAIHELGATVSEIASNAAMAADVAKQATLHSGEGKKVVGEVQNRIQTLVNE
LDNATQVVSSLATQINGISSTLDTIRSISEQTNLLALNAAIEAARAGEQGRGFAVVADEV
RTLASRSAASTEEIQQVINRLQTESTRAVEAMEKGRSQSDVVVEFSAKANQSLTEINSQI
DQINDQNIQVATATEEQSTVVEDINRNVEDINQLTTETSHVADELSRASASLQRLSSQLD
KLVGSFEL
>P19809
MITHGFYARTRHKHKLKKTFIMLSAGLGLFFYVNQNSFANGENYFKLGSDSKLLTHNSYQ
NRLFYTLKTGETVADLSKSQDINLSTIWSLNKHLYSSESEMMKAEPGQQIILPLKKLPFE
YSALPLLGSAPLVAAGGVAGHTNKLTKMSPDVTKSNMTDDKALNYAAQQAASLGSQLQSR
SLNGDYAKDTALGIAGNQASSQLQAWLQHYGTAEVNLQSGNNFDGSSLDFLLPFYDSEKM
LAFGQVGARYIDSRFTANLGAGQRFFLPENMLGYNVFIDQDFSGDNTRLGIGGEYWRDYF
KSSVNGYFRMSGWHESYNKKDYDERPANGFDIRFNGYLPSYPALGAKLMYEQYYGDNVAL
FNSDKLQSNPGAATVGVNYTPIPLVTMGIDYRHGTGNENDLLYSMQFRYQFDKPWSQQIE
PQYVNELRTLSGSRYDLVQRNNNIILEYKKQDILSLNIPHDINGTERSTQKIQLIVKSKY
GLDRIVWDDSALRSQGGQIQHSGSQSAQDYQAILPAYVQGGSNVYKVTARAYDRNGNSSN
NVLLTITVLSNGQVVDQVGVTDFTADKTSAKADGTEAITYTATVKKNGVAQANVPVSFNI
VSGTAVLSANSANTNGSGKATVTLKSDKPGQVVVSAKTAEMTSALNANAVIFVDQTKASI
TEIKADKTTAVANGQDAITYTVKVMKGDKPVSNQEVTFTTTLGKLSNSTEKTDTNGYAKV
TLTSTTPGKSLVSARVSDVAVDVKAPEVEFFTTLTIDDGNIEIVGTGVKGKLPTVWLQYG
QVNLKASGGNGKYTWRSANPAIASVDASSGQVTLKEKGTTTISVISSDNQTATYTIATPN
SLIVPNMSKRVTYNDAVNTCKNFGGKLPSSQNELENVFKAWGAANKYEYYKSSQTIISWV
QQTAQDAKSGVASTYDLVKQNPLNNIKASESNAYATCVK
>P37194
MNMTKGALILSLSFLLAACSSIPQNIKGNNQPDIQKSFVAVHNQPGLYVGQQARFGGKVI
NVINGKTDTLLEISVLPLDSYAKPDIEANYQGRLLARQSGFLDPVNYRNHFVTILGTIQG
EQPGFINKVPYNFLEVNMQGIQVWHLREVVNTTYNLWDYGYGAFWPEPGWGAPYYTNAVS
QVTPELVK
>P18895
MNSSRSVNPRPSFAPRALSLAIALLLGAPAFAANSGEAPKNFGLDVKITGESENDRDLGT
APGGTLNDIGIDLRPWAFGQWGDWSAYFMGQAVAATDTIETDTLQSDTDDGNNSRNDGRE
PDKSYLAAREFWVDYAGLTAYPGEHLRFGRQRLREDSGQWQDTNIEALNWSFETTLLNAH
AGVAQRFSEYRTDLDELAPEDKDRTHVFGDISTQWAPHHRIGVRIHHADDSGHLRRPGEE
VDNLDKTYTGQLTWLGIEATGDAYNYRSSMPLNYWASATWLTGDRDNLTTTTVDDRRIAT
GKQSGDVNAFGVDLGLRWNIDEQWKAGVGYARGSGGGKDGEEQFQQTGLESNRSNFTGTR
SRVHRFGEAFRGELSNLQAATLFGSWQLREDYDASLVYHKFWRVDDDSDIGTSGINAALQ
PGEKDIGQELDLVVTKYFKQGLLPASMSQYVDEPSALIRFRGGLFKPGDAYGPGTDSTMH
RAFVDFIWRF
>P14542
MMISKKYTLWALNPLLLTMMAPAVAQQTDDETFVVSANRSNRTVAEMAQTTWVIENAELE

QQIQGGKELKDALAQLIPGLDVSSRSRTNYGMNVRGRPLVVLVDGVRLNSSRTDSRQLDS
IDPFNMHHIEVIFGATSLYGGGSTGGLINIVTKKGQPETMMEFEAGTKSGFSSSKDHDER
IAGAVSGGNEHISGRLSVAYQKFGGWFDGNGDATLLDNTQTGLQYSDRLDIMGTGTLNID
ESRQLQLITQYYKSQGDDDYGLNLGKGFSAIRGTSTPFVSNGLNSDRIPGTDGHLISLQY
SDSAFLGQELVGQVYYRDESLRFYPFPTVNANKQVTAFSSSQQDTDQYGMKLTLNSKPMD
GWQITWGLDADHERFTSNQMFFDLAQASASGGLNNKKIYTTGRYPSYDITNLAAFLQSGY
DINNLFTLNGGVRYQYTENKIDDFIGYAQQRQIGAGKATSADAFWRLSRLRHFLFNAGLL
MHITEPQQAWLNFSQGLELPDPGKYYGRGIYGAAVNGHLPLTKSVNVSDSKLEGVKVDSY
ELGWRFTGNNLRTQIAAYYSISDKSVVANKDLTISVVDDKRRIYGVEGAVDYLIPDTDWS
TGVNFNVLKTESKVNGTWQKYDVKTASPSKATAYIGWAPDPWSLRVQSTTSFDVSDAQGY
KVDGYTTVDLLGSYQLPVGTLSFSIENLFDRDYTTVWGQRAPLYYSPGYGPASLYDYKGR
GRTFGLNYSVLF
>P0A3N8
MKRFRIVAPLALMSLALAACETTGPGSGNAPIIAHTPAGIEGSWVDPNGIASSFNGGIFE
TRTTDTNEKLAEGNYLYLSPQLVEINMRSIVRGTTSKVNCALVSPTQLNCTSSAGSRFSL
TRRNAG
>P16465
MKKKVVLLTLLSCFSTSGLSANETGNLGSISESRRALQDSQREINQLIEQNRYQQLQEKA
VNISPTPTLITESEHCLPIKGVYIQGITLLTEKDLNSLSPLPDQCIKSADINRLVKELTQ
RYLQHGYITARIQFLRPNQHGELGLYAIEGFVERIEGGDRGVNTTLLFPRIKGQPLKLAT
LDQGLDQANRLQSNKVTVDILPGTELGGSVIKLSNQRKSPWHLNIASDNYGQKNSGRWLI
RTNASLDSPLGLSDFVSLNANITTDNPNTRFNRAYTLLYSIPYGGFTFSSFGSYSEYQFH
QKLQTRTVNLYGDTTQVGIRGDYAFSRSQKQIDTLNIQVTHKRIRNYFSQIRLDLSSPKL
TTIELGINHLQIIPNGVLSTNLSVEKAVGWFGAEETPYIANGNGNDYRFTKVKLFTNWYQ
RFSLWHSTFLFNSTFLGQYSHDTLPGVEWLSLTDKNAIRGFDQSTLSGDNGGYLRNTLSY
PYRLNHFSITPRIGVDIGQVKQHGNYKGWQGGYGLSSGLNIQYQQAQLDLEVAKGELLYH
QTNSNKTKDPTQLLVKFSYLF
>P46026
MKKTLAALIVGAFAASAANAAVVYNNEGTNVELGGRLSIITEQSNSTVDDQEQQHGALRN
AGSRFHIKATHNFGDGFYAQGYLETRLVSDYPGSSSDHFGGITTKYAYVTLGNKAFGEVK
LGRAKTIADGITSAEDKEYGVLNNKKYILTNGNTVGYTYKGIDGLDGLVLGANYLLAQSR
VPGGPGLFPRKQGEVYPQQISNGVQVGAKYDANNIIAGIAFGRTNYKTAGADFDFSDAFG
LGRKEQVEGVLSTLGYRFSDLGLLVSLDSGYAKTKYHTTTTDSDDGRQTITNPAYDEKRS
FVSPGFQYELMEDTNVYGNFKYERTSVNQGKNTREQAVLFGVDHKLHKQVLTYIEGAYAR
TKTNDKGKTEKTGKEKSVGVGLRVYF
>P06626
MYPMDRIQQKHARQIDLLENLTAVIQDYPNPACIRDETGKFIFCNTLFHESFLTQDQSAE
KWLLSQRDFCELISVTEMEAYRNEHTHLNLVEDVFIQNRFWTISVQSFLNGHRNIILWQF
YDAAHVRHKDSYNQKTIVSDDIRNIIRRMSDDSSVSSYVNDVFYLYSTGISHNAIARILN
ISISTSKKHASLICDYFSVSNKDELIIILLYNKKFIYYLYEKAMCIINTR
>P26093
MKTTLKMTALAALSAFVLAGCGSHQMKSEGHANMQLQQQAVLGLNWMQDSGEYKALAYQA
YNAAKVAFDHAKVAKGKKKAVVADLDETMLDNSPYAGWQVQNNKPFDGKDWTRWVDARQS
RAVPGAVEFNNYVNSHNGKVFYVTNRKDSTEKSGTIDDMKRLGFNGVEESAFYLKKDKSA

KAARFAEIEKQGYEIVLYVGDNLDDFGNTVYGKLNADRRAFVDQNQGKFGKTFIMLPNAN
YGGWEGGLAEGYFKKDTQGQIKARLDAVQAWDGK
>P13670
MLKHSLIAASVITTLAGCSSLQSSEQQVVNSLADNLDIQYEVLTNHGANEGLACQDMGAE
WASCNKVNMTLVNQGEAVDSKDWAIYFHSIRLILDVDNEQFKISRVTGDLHKLEPTDKFD
GFAAGEEVVLPLVGEYWQLFETDFMPGAFVSAPNAEPKMIASLNTEDVASFVTGLEGNNL
KRTPDDNNVFANAVSRFEKNEDLATQDVSTTLLPTPMHVEAGKGKVDIADGIALPKDAFD
ATQFAAIQDRAEVVGVDVRGDLPVSITVVPADFTGELAKSGAYEMSIKGDGIVIKAFDQA
GAFYAVQSIFGLVDSQNADSLPQLSIKDAPRFDYRGVMVDVARNFHSKDAILATLDQMAA
YKMNKLHLHLTDDEGWRLEIPGLPELTEVGANRCFDTQEKSCLLPQLGSGPTTDNFGSGY
FSKADYVEILKYAKARNIEVIPEIDMPAHARAAVVSMEARYDRLMEEGKEAEANEYRLMD
PQDTSNVTTVQFYNKQSFINPCMESSTRFVDKVISEVAAMHQEAGAPLTTWHFGGDEAKN
IKLGAGFQDVNAEDKVSWKGTIDLSKQDKPFAQSPQCQTLITDGTVSDFAHLPSHFAEEV
SKIVAEKGIPNFQAWQDGLKYSDGEKAFATENTRVNFWDVLYWGGTSSVYEWSKKGYDVI
VSNPDYVYMDMPYEVDPKERGYYWATRATDTRKMFGFAPENMPQNAETSVDRDGNGFTGK
GEIEAKPFYGLSAQLWSETVRNDEQYEYMVFPRVLAAAQRAWHRADWENDYKVGVEYSQN
SNLVDKASLNQDYNRFANVLGQRELAKLEKSGIDYRLPVPGAKVEDGKLAMNVQFPGVTL
QYSLDGENWLTYADNARPNVTGEVFIRSVSATGEKVSRITSVK
>P17907
MENDEKEHGRRRRQHLRVPVFPEEKDEIEANAKRAGVSVARYLRDVGQGYQIKGVMDYQH
VRELVRVNGDLGRLGGLLKLWLTDDVRTLQFGEATILALLGRIEATQDEMSRIMKAVVQP
RAEP
>P05695
MIRRHSCKGVGSSVAWSLLGLAISAQSLAGTVTTDGADIVIKTKGGLEVATTDKEFSFKL
GGRLQADYGRFDGYYTNNGNTADAAYFRRAYLEFGGTAYRDWKYQINYDLSRNVGNDSAG
YFDEASVTYTGFNPVNLKFGRFYTDFGLEKATSSKWVTALERNLTYDIADWVNDNVGTGI
QASSVVGGMAFLSGSVFSENNNDTDGDSVKRYNLRGVFAPLHEPGNVVHLGLQYAYRDLE
DSAVDTRIRPRMGMRGVSTNGGNDAGSNGNRGLFGGSSAVEGLWKDDSVWGLEGAWALGA
FSAQAEYLRRTVKAERDREDLKASGYYAQLAYTLTGEPRLYKLDGAKFDTIKPENKEIGA
WELFYRYDSIKVEDDNIVVDSATREVGDAKGKTHTLGVNWYANEAVKVSANYVKAKTDKI
SNANGDDSGDGLVMRLQYVF
>P31600
MKENNLNRVIGWSGLLLTSLLSTSALADNIGTSAEELGLSDYRHFVIYPRLDKALKAQKN
NDEATAIREFEYIHQQVPDNIPLTLYLAEAYRHFGHDDRARLLLEDQLKRHPGDARLERS
LAAIPVEVKSVTTVEELLAQQKACDAAPTLRCRSEVGQNALRLAQLPVARAQLNDATFAA
SPEGKTLRTDLLQRAIYLKQWSQADTLYNEARQQNTLSAAERRQWFDVLLAGQLDDRILA
LQSQGIFTDPQSYITYATALAYRGEKARLQHYLIENKPLFTTDAQEKSWLYLLSKYSANP
VQALANYTVQFADNRQYVVGATLPVLLKEGQYDAAQKLLATLPANEMLEERYAVSVATRN
KAEALRLARLLYQQEPANLTRLDQLTWQLMQNEQSREAADLLLQRYPFQGDARVSQTLMA
RLASLLESHPYLATPAKVAILSKPLPLAEQRQWQSQLPGIADNCPAIVRLLGDMSPSYDA
AAWNRLAKCYRDTLPGVALYAWLQAEQRQPSAWQHRAVAYQAYQVEDYATALAAWQKISL
HDMSNEDLLAAANTAQAAGNGAARDRWLQQAEKRGLGSNALYWWLHAQRYIPGQPELALN
DLTRSINIAPSANAYVARATIYRQRHNVPAAVSDLRAALELEPNNSNTQAALGYALWDSG
DIAQSREMLEPAHKGLPDDPALIRQLAYVNQRLDDMPATQHYARLVIDDIDNQALITPLT

PEQNQQRFNFRRLHEEVGRRWTFSFDSSIGLRSGAMSTANNNVGGAAPGKSYRSYGQLEA
EYRIGRNMLLEGDLLSVYSRVFADTGENGVMMPVKNPMSGTGLRWKPLRDQIFFIAVEQQ
LPLNGQNGASDTMLRASASFFNGGKYSDEWHPNGSGWFAQNLYLDAAQYIRQDIQAWTAD
YRVSWHQKVANGQTIEPYAHVQDNGYRDKGTQGAQLGGVGVRWNIWTGETHYDAWPHKVS
LGVEYQHTFKAINQRNGERNNAFLTIGVHW
>P69776
MKATKLVLGAVILGSTLLAGCSSNAKIDQLSSDVQTLNAKVDQLSNDVNAMRSDVQAAKD
DAARANQRLDNMATKYRK
>P31106
MKMRLVAAAAMGLAMSTTIAATATTDATTSAPGTSLTTDTEKLSYSIGADLGKNFKKQGI
EISPAAMAKGLQDGMSGGQLLLTDDQMKDVLNKFQKDLMMKRSAEFNKKAEENKSKGEAF
LNENKSKEGVVSLPSGLQYNILERGDGAKPTKDDVVTVEYTGKLIDGQVFDSTEKTGKPA
TFKVSQVIPGWTEALQLMPAGSTWEVYIPSNLAYGPRSVGGPIGPNETLIFKIHLISVKK
SDA
>P38370
MHLNRVLRETGVVVAAGLLYGSAAFAQSSTIIGTVIDAQSRQPAADVVVTATSPNLQGEQ
TVVTDAQGNYRIPQLPPGDYTLRFEKEQFKPYARSAIQLRLNRTIRVNVELLPEALGEVV
EIVGAPPTIDVGSTTMGVNVDQEFIKRIAVARPGGKGGATRSFESLAELAPGAQNDNYGV
SINGSTSPENGYVVDGLSTNDPAFGVNASPLSIEFVQDVNIITGGYMPEFGRSTGGVINA
VTRSGSNEFHGSVFANWTPGTLEGTRKQIREEGTVITGQNQLQNLGDFGATLGGPILKDK
LWFFAGFAPSFTRYQHTRTLNALRVDDEGNTIKDETDFTVADAIPGSARKYYADSRTIQY
MGKLTYLINQDHNVSFALNGTPTSTGGLGKLSVNPQSGGLPGVLATRPGDFGLTETKANT
TSLALKYAGAFADKKVLVDANLGWFHQTASTLPGDGSNLGDRTGLAGYSRMVYTTPRALT
LFEALPEGQEGACGSTPEEQLVRSPVTGYGVGGPGFMSDQTLDRYQANAKATYLLNALGT
HVFKAGVDVELLSFDQVKAYGGGVFFQEGSNYGVAGQGPAVHDARRYGYQTGPDSAVTQF
TQVAKTTSTTVGGFLQDSWSIANRVTLNLGVRYDVQALYGGNGDLSLLLGNQWSPRIGAI
VDPFANGRAKVFVNFARYYEQVPLNLMDRAFPGENRISARRSLAEPGQGTATSCDPSSFE
SQQATCNTDSNLLAIPESSRNVNRFYTGGTVGGTPVDPDIKAQSSDEIVVGAEYEVLANT
RLGASYTHKDMNSVIEDMSRDDGNTYFLGNPGSGFAGEFPTPVRNYDNVTVYLNRTFADG
WLAQANYTWSRLYGNYPGLFRPETGQLDPNILSDFDLIELLENRTGLLPFDRTHQIKVFG
AKEFNISNALSASVGVSYRGSSGTPINYWGSHWAYLQDESFVLPRGAGGRTPWINTIDSN
IGVNYRVSKDSVVSFTLDVFNLFNFQGVNTVDQTYTLRDIKPIPGGTPADLENLPGRVEF
QDQAPRDEPFGSVDGDVNKNFKNPLSYQAPRQVRFGIRYTF
>P15921
MANISPKLFKKAIQQGLKAALFTTSTAAIMLSSSGALGVATGVIATNNNAAFSNNVGNNN
WNEITAAGVANGTPAGGPQNNWAFTYGGDYTVTADAADRIIKAINVAGTTPVGLNITQNT
VVGSIITKGNLLPVTLNAGKSLTLNGNNAVAANHGFDAPADNYTGLGNIALGGANAALII
QSAAPSKITLAGNIDGGGIITVKTDAAINGTIGNTNALATVNVGAGTATLGGAVIKATTT
KLTNAASVLTLTNANAVLTGAIDNTTGGDNVGVLNLNGALSQVTGDIGNTNSLATISVGA
GTATLGGAVIKATTTKLTDAASAVKFTNPVVVTGAIDNTGNANNGIVTFTGNSTVTGNVG
NTNALATVNVGAGLLQVQGGVVKANTINLTDNASAVTFTNPVVVTGAIDNTGNANNGIVT
FTGNSTVTGDIGNTNALATVNVGAGTATLGGAVIKATTTKLTNAASVLTLTNANAVLTGA
IDNTTGGDNVGVLNLNGALSQVTGNIGNTNSLATISVGAGTATLGGAVIKATTTKLTDAA
SAVKFTNPVVVTGAIDNTGNANNGIVTFTGNSTVTGDIGNTNSLATISVGAGTATLGGAV

IKATTTKLTNAASVLTLTNANAVLTGAIDNTTGGDNVGVLNLNGALSQVTGDIGNTNSLA
TISVGAGTATLGGAVIKATTTKITNAVSAVKFTNPVVVTGAIDSTGNANNGIVTFTGNST
VTGDIGNTNALATVNVGAGTATLGGAVIKATTTKLTNAASVLTLTNANAVLTGAIDNTTG
GDNVGVLNLNGALSQVTGDIGNTNSLATISVGAGTATLGGAVIKATTTKLTNAASVLTLT
NANAVLTGAVDNTTGGDNVGVLNLNGALSQVTGDIGNTNSLATISVGAGTATLGGAVIKA
TTTKLTNAASVLTLTNANAVLTGAIDNTTGGDNVGVLNLNGALSQVTGDIGNTNSLATIS
VGAGTATLGGAVIKATTTKLTDAASAVKFTNPVVVTGAIDNTGNANNGIVTFTGNSTVTG
NVGNTNALATVNVGAGLLQVQGGVVKANTINLTDNASAVTFTNPVVVTGAIDNTGNANNG
IVTFTGNSTVTGNVGNTNALATVNVGAGLLQVQGGVVKANTINLTDNASAVTFTNPVVVT
GAIDNTGNANNGIVTFTGNSTVTGDIGNTNALATVNVGAGITLQAGGSLAANNIDFGARS
TLEFNGPLDGGGKAIPYYFKGAIANGNNAILNVNTKLLTASHLTIGTVAEINIGAGNLFT
IDASVGDVTILNAQNINFRARDSVLVLSNLTGVGVNNILLAADLVAPGADEGTVVFNGGV
NGLNVGSNVAGTARNIGDGGGNKFNTLLIYNAVTITDDVNLEGIQNVLINKNADFTSSTA
FNAGAIQINDATYTIDANNGNLNIPAGNIQFAHADAQLVLQNSSGNDRTITLGANIDPDN
DDEGIVILNSVTAGKKLTIAGGKTFGGAHKLQTILFKGAGDCSTAGTTFNTTNIVLDITG
QLELGATTANVVLFNDAVQLTQTGNIGGFLDFNAKNGMVTLNNNVNVAGAVQNTGGTNNG
TLIVLGASNLNRVNGIAMLKVGAGNVTIAKGGKVKIGEIQGTGTNTLTLPAHFNLTGSIN
KTGGQALKLNFMNGGSVSGVVGTAANSVGDITTAGATSFASSVNAKGTATLGGTTSFANT
FTNTGAVTLAKGSITSFAKNVTATSFVANSATINFSNSLAFNSNITGGGTTLTLGANQVT
YTGTGSFTDTLTLNTTFDGAAKSGGNILIKSGSTLDLSGVSTLALVVTATNFDMNNISPD
TKYTVISAETAGGLKPTSKENVKITINNDNRFVDFTFDASTLTLFAEDIAADVIDGDFAP
GGPLANIPNAANIKKSLELMEDAPNGSDARQAFNNFGLMTPLQEADATTHLIQDVVKPSD
TIAAVNNQVVASNISSNITALNARMDKVQSGNKGPVSSGDEDMDAKFGAWISPFVGNATQ
KMCNSISGYKSDTTGGTIGFDGFVSDDLALGLAYTRADTDIKLKNNKTGDKNKVESNIYS
LYGLYNVPYENLFVEAIASYSDNKIRSKSRRVIATTLETVGYQTANGKYKSESYTGQLMA
GYTYMMPENINLTPLAGLRYSTIKDKGYKETGTTYQNLTVKGKNYNTFDGLLGAKVSSNI
NVNEIVLTPELYAMVDYAFKNKVSAIDARLQGMTAPLPTNSFKQSKTSFDVGVGVTAKHK
MMEYRINYDTNIGSKYFAQQGSVKVRVNF
>Q53047
MAQKPNFLKKLISAGLVTASTATIVASFAGSAMGAAIQQNRTTNGAATTVDGAGFDQTAA
PANVGVALNAVITANANNGINFNTPAGSFNGLLLNTANNLAVTVSEDTTLGFITNVVHNA
HSFNLTLNAGKTLTITGQGVTNAQAAATKNAQNVVVQFNNGAAIDNNDLKGVGRIDFGAP
ASTLVFNLANPTTQKAPLILGDNAVIANGVNGTLNVTNGFIQVSNKSFATVKAINIADGQ
GIIFNTDANNANTLNLQAGGTTINFTGTDGTGRLVLLSKHAAATNFNITGSLGGNLKGVI
EFNTVAVDGQLTANAGAANAVIGTNNGAGRAAGFVVSVDNGKVATIDGQVYAKDMVIQSA
NATGQVNFRHIVDVGADGTTAFKTAASKVTITQDSNFGNTDFGNLAAQIKVPNAITLTGN
FTGDASNPGNTAGVITFDANGTLESASADANVAVTNNITAIEASGAGVVQLSGTHAAELR
LGNAGSIFKLADGTVINGKVNQTALVGGALAAGTITLDGSATITGDIGNAGGAAALQRIT
LANDAKKTLTLGGANIIGAGGGTIDLQANGGTIKLTSTQNNIVVDFDLAIATDQTGVVDA
SSLTNAQTLTINGKIGTIGANNKTLGQFNIGSSKTVLSNGNVAINELVIGNDGAVQFAHD
TYLITRTTNAAGQGKIIFNPVVNNGTTLAAGTNLGSATNPLAEINFGSKGVNVDTVLNVG
EGVNLYATNITTTDANVGSFVFNAGGTNIVSGTVGGQQGNKFNTVALENGTTVKFLGNAT
FNGNTTIAANSTLQIGGNYTADCVASADGTGIVEFVNTGPITVTLNKQAAPVNALKQITV
SGPGNVVINEIGNAGNHHGAVTDTIAFENSSLGAVVFLPRGIPFNDAGNTMPLTIKSTVG

NKTAKGFDVPSVVVLGVDSVIADGQVIGDQNNIVGLGLGSDNGIIVNATTLYAGISTLNN
NQGTVTLSGGVPNTPGTVYGLGTGIGASKFKQVTFTTDYNNLGNIIATNATINDGVTVTT
GGIAGIGFDGKITLGSVNGNGNVRFADGILSNSTSMIGTTKANNGTVTYLGNAFVGNIGD
SDTPVASVRFTGSDSGAGLQGNIYSQVIDFGTYNLGIVNSNIILGGGTTAINGKIDLVTN
TLTFASGTSTWGNNTSIETTLTLANGNIGHIVILEGAQVNTTTTGTTTIKVQDNANANFS
GTQTYTLIQGGARFNGTLGSPNFAVTGSNRFVNYSLIRAANQDYVITRTNNAENVVTNDI
ANSPFGGAPGVDQNVTTFVNATNTAAYNNLLLAKNSANSANFVGAIVTDTSAAITNVQLD
LAKDIQAQLGNRLGALRYLGTPETAEMAGPEAGAISAAVAAGDEAIDNVAYGIWAKPFYT
DAHQSKKGGLAGYKAKTTGVVIGLDTLANDNLMIGAAIGITKTDIKHQDYKKGDKTDVNG
FSFSLYGAQQLVKNFFAQGSAIFSLNQVKNKSQRYFFDANGNMSKQIAAGHYDNMTFGGN
LTVGYDYNAMQGVLVTPMAGLSYLKSSDENYKETGTTVANKQVNSKFSDRTDLIVGAKVA
GSTMNITDLAVYPEVHAFVVHKVTGRLSKTQSVLDGQVTPCINQPDRTTKTSYNLGLSAS
IRSDAKMEYGIGYDAQISSKYTAHQGTLKVRVNF
>P31489
MTKDFKISVSAALISALFSSPYAFADDYDGIPNLTAVQISPNADPALGLEYPVRPPVPGA
GGLNASAKGIHSIAIGATAEAAKGAAVAVGAGSIATGVNSVAIGPLSKALGDSAVTYGAA
STAQKDGVAIGARASTSDTGVAVGFNSKADAKNSVAIGHSSHVAANHGYSIAIGDRSKTD
RENSVSIGHESLNRQLTHLAAGTKDTDAVNVAQLKKEIEKTQENTNKRSAELLANANAYA
DNKSSSVLGIANNYTDSKSAETLENARKEAFAQSKDVLNMAKAHSNSVARTTLETAEEHA
NSVARTTLETAEEHANKKSAEALASANVYADSKSSHTLKTANSYTDVTVSNSTKKAIRES
NQYTDHKFRQLDNRLDKLDTRVDKGLASSAALNSLFQPYGVGKVNFTAGVGGYRSSQALA
IGSGYRVNENVALKAGVAYAGSSDVMYNASFNIEW
>P13036
MTPLRVFRKTTPLVNTIRLSLLPLAGLSFSAFAAQVNIAPGSLDKALNQYAAHSGFTLSV
DASLTRGKQSNGLHGDYDVESGLQQLLDGSGLQVKPLGNNSWTLEPAPAPKEDALTVVGD
WLGDARENDVFEHAGARDVIRREDFAKTGATTMREVLNRIPGVSAPENNGTGSHDLAMNF
GIRGLNPRLASRSTVLMDGIPVPFAPYGQPQLSLAPVSLGNMDAIDVVRGGGAVRYGPQS
VGGVVNFVTRAIPQDFGIEAGVEGQLSPTSSQNNPKETHNLMVGGTADNGFGTALLYSGT
RGSDWREHSATRIDDLMLKSKYAPDEVHTFNSLLQYYDGEADMPGGLSRADYDADRWQST
RPYDRFWGRRKLASLGYQFQPDSQHKFNIQGFYTQTLRSGYLEQGKRITLSPRNYWVRGI
EPRYSQIFMIGPSAHEVGVGYRYLNESTHEMRYYTATSSGQLPSGSSPYDRDTRSGTEAH
AWYLDDKIDIGNWTITPGMRFEHIESYQNNAITGTHEEVSYNAPLPALNVLYHLTDSWNL
YANTEGSFGTVQYSQIGKAVQSGNVEPEKARTWELGTRYDDGALTAEMGLFLINFNNQYD
SNQTNDTVTARGKTRHTGLETQARYDLGTLTPTLDNVSIYASYAYVNAEIREKGDTYGNL
VPFSPKHKGTLGVDYKPGNWTFNLNSDFQSSQFADNANTVKESADGSTGRIPGFMLWGAR
VAYDFGPQMADLNLAFGVKNIFDQDYFIRSYDDNNKGIYAGQPRTLYMQGSLKF
>P0ABZ6
MKNWKTLLLGIAMIANTSFAAPQVVDKVAAVVNNGVVLESDVDGLMQSVKLNAAQARQQL
PDDATLRHQIMERLIMDQIILQMGQKMGVKISDEQLDQAIANIAKQNNMTLDQMRSRLAY
DGLNYNTYRNQIRKEMIISEVRNNEVRRRITILPQEVESLAQQVGNQNDASTELNLSHIL
IPLPENPTSDQVNEAESQARAIVDQARNGADFGKLAIAHSADQQALNGGQMGWGRIQELP
GIFAQALSTAKKGDIVGPIRSGVGFHILKVNDLRGESKNISVTEVHARHILLKPSPIMTD
EQARVKLEQIAADIKSGKTTFAAAAKEFSQDPGSANQGGDLGWATPDIFDPAFRDALTRL
NKGQMSAPVHSSFGWHLIELLDTRNVDKTDAAQKDRAYRMLMNRKFSEEAASWMQEQRAS

AYVKILSN
>Q06282
MKLKTLALSLLAAGVLAGCSSHSSNMANTQMKSDKIIIAHRGASGYLPEHTLESKALAFA
QHSDYLEQDLAMTKDGRLVVIHDHFLDGLTDVAKKFPYRHRKDGRYYVIDFTLKEIQSLE
MTENFETKDGKQAQVYPNRFPLWKSHFRIHTFEDEIEFIQGLEKSTGKKVGIYPEIKAPW
FHHQNGKDIATETLKVLKKYGYDKKTDMVYLQTFDFNELKRIKTELLPQMGMDLKLVQLI
AYTDWKETQEKDPKGYWVNYNYDWMFKPGAMAEVVKYADGVGPGWYMLVNKEESKPDNIV
YTPLVKELAQYNVEVHPYTVRKDALPEFFTDVNQMYDALLNKSGATGVFTDFPDTGVEFL
KGIK
>P43262
MKLRLSALALGTTLLVGCASSGTDQQGRSDPLEGFNRTMYNFNFNVLDPYIVRPVAVAWR
DYVPQPARNGLSNFTGNLEEPAVMVNYFLQGDPYQGMVHFTRFFLNTILGMGGFIDVAGM
ANPKLQRTEPHRFGSTLGHYGVGYGPYVQLPFYGSFTLRDDGGDMADALYPVLSWLTWPM
SVGKWTLEGIETRAQLLDSDGLLRQSSDPYIMVREAYFQRHDFIANGGELKPQENPNAQA
IQDDLKDIDSE
>P80672
MKLVKLSLVAALAAGAFSAANATPLEEAIKDVDVSGVLRYRYDTGNFDKNFVNNSNLNNS
KQDHKYRAQVNFSAAIADNFKAFVQFDYNAADGGYGANGIKNDQKGLFVRQLYLTYTNED
VATSVIAGKQQLNLIWTDNAIDGLVGTGVKVVNNSIDGLTLAAFAVDSFMAAEQGADLLE
HSNISTTSNQAPFKVDSVGNLYGAAAVGSYDLAGGQFNPQLWLAYWDQVAFFYAVDAAYS
TTIFDGINWTLEGAYLGNSLDSELDDKTHANGNLFALKGSIEVNGWDASLGGLYYGDKEK
ASTVVIEDQGNLGSLLAGEEIFYTTGSRLNGDTGRNIFGYVTGGYTFNETVRVGADFVYG
GTKTEAANHLGGGKKLEAVARVDYKYSPKLNFSAFYSYVNLDQGVNTNESADHSTVRLQA
LYKF
>Q83EK8
METTTKLAIGVSALCCLASAAFAGGPDIPMIDMNGFHIGLGFGYKSYTYDQVGTVTVTTN
GGTVLSVLHPVSASITQFGPVGELGYTFASDWWIAGVKAQYQYDNVRSVHIMDAPLVGSN
YSYRTRLGSHLTAMLLAGIKVNEANAVYLEAGYSTVWGKTTLFGPGPVAVSMKNRLNGGI
AGIGWRHYFMNNVFLDLSYDYALYRSKSNSVTLSSATASAEGTAIGVSGTVQNPKRVAIN
GITATVNYLFNI
>Q04064
MKKTLLAAALLAGFAGAAQAETSVTLYGIIDTGIGYNDVDFKVKGANADDSDFKYNHSRF
GMINGVQNGSRWGLRGTEDLGDGLQAVFQLESGFNSGNGNSAQDGRLFGRQATIGLQSES
WGRLDFGRQTNIASKYFGSIDPFGAGFGQANIGMGMSAMNTVRYDNMVMYQTPSYSGFQF
GIGYSFSANDKDADAVNRVGFATADNVRAITTGLRYVNGPLNVALSYDQLNASNNQAQGE
VDATPRSYGLGGSYDFEVVKLALAYARTTDGWFGGQGYPVAVTLPSGDKFGGFGVNTFAD
GFKANSYMVGLSAPIGGASNVFGSWQMVDPKLTGGDEKMNVFSLGYTYDLSKRTNLYAYG
SYAKNFAFLEDAKSTAVGVGIRHRF
>O68562
MQNAKLMLTCLAFAGLAALAGCSFPGVYKIDIQQGNVVTQDMIDQLRPGMTRRQVRFIMG
NPLIVDTFHANRWDYLYSIQPGGGRRQQERVSLFFNDSDQLAGLNGDFMPGVSRDEAILG
KEGSTTVTQPADQQKPEAQKEEPPKPGSTLEQLQREVDEAQPVPVPTPEPLDPSPQ
>O08237
MKKIALFITASLIAGNTLAAQTYIRNGNIYTHEGQWVAEVGAFGSTDLLKDQDKSYSALL

NFGYHGEDLNADLTGINYRFFGNTGDVVNLGTYLTGSGVTYDQDSANSVKGMDKRKATID
LGLNADIALGDGTVSTYFQHDILNENKGYKTGVNYFHVIDLGAVDLVPFAGISYQSSDYN
NYYFGVKDKEATAQRKAYHAGGDFSYNLGYKLAYPINDRWEITQTSSYTRLGSDVTNSPI
VESANQWLVGATVAYHF
>P08321
MSGDENKLKKYRFPETLTNQSRWFGLPLDELIPAAICIGWGITTSKYLFGIGAAVLVYFG
IKKLKKGRGSSWLRDLIYWYMPTALLRGIFS
>A5F934
MNKTLIALAVSAAAVATGAYADGINQSGDKAGSTVYSAKGTSLEVGGRAEARLSLKDGKA
QDNSRVRLNFLGKAEINDSLYGVGFYEGEFTTNDQGKNASNNSLDNRYTYAGIGGTYGEV
TYGKNDGALGVITDFTDIMSYHGNTAAEKIAVADRVDNMLAYKGQFGDLGVKASYRFADR
NAVDAMGNVVTETNAAKYSDNGEDGYSLSAIYTFGDTGFNVGAGYADQDDQNEYMLAASY
RMENLYFAGLFTDGELAKDVDYTGYELAAGYKLGQAAFTATYNNAETAKKTSADNFAIDA
TYYFKPNFRSYISYQFNLLDSDKASKVASEDELAIGLRYDF
>P24082
MKRILPLILALVAGMAQADSNSDYRAGSDFAHQIKGQGSSSIQGFKPQESIPGYNANPDE
TKYYGGVTAGGDGGLKNDGTTEWATGETGKTITESFMNKPKDILSPDAPFIQTGRDVVNR
ADSIVGNTGQQCSAQEISRSEYTNYTCERDLQVEQYCTRTARMELQGSTTWETRTLEYEM
SQLPAREVNGQYVVSITSPVTGEIVDAHYSWSRTYLQKSVPMTITVLGTPLSWNAKYSAD
ASFTPVQKTLTAGVAFTSSHPVRVGNTKFKRHTAMKLRLVVRVKKASYTPYVVWSESCPF
SKELGKLTKTECTEAGGNRTLVKDGQSYSMYQSCWAYRDTYVTQSADKGTCQTYTDNPAC
TLVSHQCAFYSEEGACLHEYATYSCESKTSGKVMVCGGDVFCLDGECDKAQSGKSNDFAE
AVSQLAALAAAGKDVAALNGVDVRAFTGQAKFCKKAAAGYSNCCKDSGWGQDIGLAKCSS
DEKALAKAKSNKLTVSVGEFCSKKVLGVCLEKKRSYCQFDSKLAQIVQQQGRNGQLRISF
GSAKHPDCRGITVDELQKIQFNRLDFTNFYEDLMNNQKIPDSGVLTQKVKEQIADQLKQA
GQ
>P32722
MKVMKWSAIALAVSAGSTQFAVADAFVSDQAEAKGFIEDSSLDLLLRNYYFNRDGKSGSG
DRVDWTQGFLTTYESGFTQGTVGFGVDAFGYLGLKLDGTSDKTGTGNLPVMNDGKPRDDY
SRAGGAVKVRISKTMLKWGEMQPTAPVFAAGGSRLFPQTATGFQLQSSEFEGLDLEAGHF
TEGKEPTTVKSRGELYATYAGETAKSADFIGGRYAITDNLSASLYGAELEDIYRQYYLNS
NYTIPLASDQSLGFDFNIYRTNDEGKAKAGDISNTTWSLAAAYTLDAHTFTLAYQKVHGD
QPFDYIGFGRNGSGAGGDSIFLANSVQYSDFNGPGEKSWQARYDLNLASYGVPGLTFMVR
YINGKDIDGTKMSDNNVGYKNYGYGEDGKHHETNLEAKYVVQSGPAKDLSFRIRQAWHRA
NADQGEGDQNEFRLIVDYPLSIL
>P05837
MQGGLNSLHRLSIESLTEVPMCALDRRERPLNSQSVNKYILNVQNIYRNSPVPVCVRNKN
WKILYANGAFIELFSREDKPLSGESYIRLQVEIFLSSLELECQALGHGSAFCRRFNFHGE
IYQIRMENVSFYNDESVVLWQINPFPDYPFFALNQSGSNTNTSDKLTIWNDLSPGTLVVF
SFYMLGVGHATIARELGITDRASEDRIKPVKRKIKEFFEHFDLFRVSCIYKGEIDSLLSI
IREFYGVK
>P69996
MKKISRKEYVSMYGPTTGDKVRLGDTDLIAEVEHDYTIYGEELKFGGGKTLREGMSQSNN
PSKEELDLIITNALIVDYTGIYKADIGIKDGKIAGIGKGGNKDMQDGVKNNLSVGPATEA

LAGEGLIVTAGGIDTHIHFISPQQIPTAFASGVTTMIGGGTGPADGTNATTITPGRRNLK
WMLRAAEEYSMNLGFLAKGNASNDASLADQIEAGAIGFKIHEDWGTTPSAINHALDVADK
YDVQVAIHTDTLNEAGCVEDTMAAIAGRTMHTFHTEGAGGGHAPDIIKVAGEHNILPAST
NPTIPFTVNTEAEHMDMLMVCHHLDKSIKEDVQFADSRIRPQTIAAEDTLHDMGIFSITS
SDSQAMGRVGEVITRTWQTADKNKKEFGRLKEEKGDNDNFRIKRYLSKYTINPAIAHGIS
EYVGSVEVGKVADLVLWSPAFFGVKPNMIIKGGFIALSQMGDANASIPTPQPVYYREMFA
HHGKAKYDANITFVSQAAYDKGIKEELGLERQVLPVKNCRNITKKDMQFNDTTAHIEVNP
ETYHVFVDGKEVTSKPANKVSLAQLFSIF
>Q9ZA21
MTNFRLNVLAYSVMLGLTASVAYAEPTNQPTNQPTNQPTNQPTNQPTNQPTNQPTNQPTN
QPTNQPTNQNSNASEQLEQINVSGSTENTDTKAPPKIAETVKTAKKLEKEQAQDVKDLVR
YETGITVVEAGRFGNSGFAVRGVEENRVAVQIDGLHQAETISSQGFKELFEGYGNFNNTR
NSAEIETLKQVTIRKGADSLKSGSGALGGSVSLDTKDARDYLLNKNYYASYKRGYNTADN
QNLNTLTLGGRYKYFDAIAVLTSRKGHELENFGYKNYNDKIQGKTREKADPYRRTQDSAL
LKIGFQPTENHRFSVVADLYKQTSKGHDFSYTLKPNTQYMTYDEKELRHTNDKVERKNIA
FVYENFTETPFWDTLKITYSHQKITTSARTDDYCDGNDKCALAGNPLGMKYNQDNQLVGK
DGKSAKYQDINKTQVIKERLPFTKPNGRWRFHKVDWDALKKKYPGVPIYASCLEEDNDPS
EFCTYEVKTTKKENTFEINGKRYDLLSEADKNVISDEQRLPTNVSYLFSCDGLNCDKKTI
LGFKKRRNLLKIFLFEVIEKRCQKYGKTKVKANDQLSGPYLFMPNKKGYQANLWSQRDLT
SETKQINLDLTKHLELGKTQHDLSYGGLWSEMEKSMTNLAGDTPLNVKWWAQYPHNCATF
LPPSTMTPNAKPTLNPERTSTLCNNVNVFSFLIPVKTKTGALYFINDFRVNNYVAFNLGY
RYDRVKYEPEYIPGKTPKIPDDMVTNLYIKTPEFDASKADSDPDELSKKEANAAANIKEI
AQPKKFSASSYSFGTTLDPLNWLRLQAKYSKGFRAPTSDEIYFTFKHPDFSIQPNRDLQP
ETAKTKELSLTVHNDMGYITTSVFDTRYQNFIDLSYQGRRDVHGHSKLIPFHFYQNVNRP
NAKVTGFEIASQISLGNITKLFNGFSLSYKYTYQKGRINGNIPMNAIQPRTAVYGVSYVH
PDDKYGLDLYISHASAKNAEDTYNMFYKEEGKTDSTIKWRSKSYTTIDLLGYIKPIKNLT
LRAGVYNLTNRKYITWDSARSIRPFGTSNMINQDTGLGINRFYAPERNYRMSVQFEF
>P0A3W4
MKYCLLCLVVALSGCQTNDTIASCKGPIFPLNVGRWQPTPSDLQLRNSGGRYDGA
>P26466
MMITLRKLPLAVAVAAGVMSAQAMAVDFHGYARSGIGWTGSGGEQQCFQATGAQSKYRLG
NECETYAELKLGQEVWKEGDKSFYFDTNVAYSVNQQNDWESTDPAFREANVQGKNLIEWL
PGSTIWAGKRFYQRHDVHMIDFYYWDISGPGAGIENIDLGFGKLSLAATRSTEAGGSYTF
SSQNIYDEVKDTANDVFDVRLAGLQTNPDGVLELGVDYGRANTTDGYKLADGASKDGWMF
TAEHTQSMLKGYNKFVVQYATDAMTTQGKGQARGSDGSSSFTEELSDGTKINYANKVINN
NGNMWRILDHGAISLGDKWDLMYVGMYQNIDWDNNLGTEWWTVGVRPMYKWTPIMSTLLE
VGYDNVKSQQTGDRNNQYKITLAQQWQAGDSIWSRPAIRIFATYAKWDEKWGYIKDGDNI
SRYAAATNSGISTNSRGDSDEWTFGAQMEIWW
>P44833
MKKSFLLLPLSLVVLSACTSNFPAPISDADGNFSPSVIQSVNGSNVGGAWQPEIQKNSLP
TTGNMVTPQQNFQPINQQPTMPTAPAQPAFQPSPKTVVSAPTVQTKTVTKTVADCVDGQH
INIPRNPNTNAPDYSKISKGSYKGNTYKVNKGDTMFLIAYLAGIDVKELAALNNLSEPYN
LSLGQVLKISNCDIKTVTTTVSVKQPAVTASTATPVKPAVTYTPGANGTQIGSDGTIIGP
IKSEAGTSPSVPVATSSTQVTSSVNNANSTPINSNVVAPIASNVVWQWPTSGNIIQGFSS

TDGGNKGIDISGSRGQAVKAAAAGRIVYAGNALRGYGNLIIIKHNDDFLSAYAHNDKILV
ADQQEVKAGQDIAKMGSSGTNTVKLHFEIRYKGKSVDPVRYLPRH
>Q934G3
MKIKLLTLAVASLVSVNALAVSIDYRHEMQDTAQAGHKDRLLISHRFANGFGLSSEVKWA
QSSADKTPNKPFNEQVSNGTEVVASYVYKFNSVFSIEPGFSLESGSSNNNYRPYLRGRAN
VTDDLSVALRYRPYFKRNSGNIGKDNTMDKGYTLTGNIDYTFLKDYTIGYELEYKKGTSG
KTILSDNDDYDITHNVKLSYKWDKNWKPYVEVGNVSGSETTDERQTRYRVGVQYSF
>Q47474
MSLTHYSGLAAAVSMSLILTACGGQTPNSARFQPVFPGTVSRPVLSAQEAGRFTPQHYFA
HGGEYAKPVADGWTPTPIDTSRVTAAYVVGPRAGVAGATHTSIQQAVNAALRQHPGQTRV
YIKLLPGTYTGTVYVPEGAPPLTLFGAGDRPEQVVVSLALDSMMSPADYRARVNPHGQYQ
PADPAWYMYNACATKAGATINTTCSAVMWSQSNDFQLKNLTVVNALLDTVDSGTHQAVAL
RTDGESGATGKCPPAQPSDTFFVNTSDRQNSYVTDHYSRAYIKDSYIEGDVDYVFGRATA
VFDRVRFHTVSSRGSKEAYVFAPDSIPSVKYGFLVINSQLTGDNGYRGAQKAKLGRAWDQ
GAKQTGYLPGKTANGQLVIRDSTIDSSYDLANPWGAAATTDRPFKGNISPQRDLDDIHFN
RLWEYNTQVLLHE
>O33407
MIRMALKPLVAACLLASLSTAPQAAPSPYSTLVVFGDSLSDAGQFPDPAGPAGSTSRFTN
RVGPTYQNGSGEIFGPTAPMLLGNQLGIAPGDLAASTSPVNAQQGIADGNNWAVGGYRTD
QIYDSITAANGSLIERDNTLLRSRDGYLVDRARQGLGADPNALYYITGGGNDFLQGRILN
DVQAQQAAGRLVDSVQALQQAGARYIVVWLLPDLGLTPATFGGPLQPFASQLSGTFNAEL
TAQLSQAGANVIPLNIPLLLKEGMANPASFGLAADQNLIGTCFSGNGCTMNPTYGINGST
PDPSKLLFNDSVHPTITGQRLIADYTYSLLSAPWELTLLPEMAHGTLRAYQDELRSQWQA
DWENWQNVGQWRGFVGGGGQRLDFDSQDSAASGDGNGYNLTLGGSYRIDEAWRAGVAAGF
YRQKLEAGAKDSDYRMNSYMASAFVQYQENRWWADAALTGGYLDYDDLKRKFALGGGERS
EKGDTNGHLWAFSARLGYDIAQQADSPWHLSPFVSADYARVEVDGYSEKGASATALDYDD
QKRSSKRLGAGLQGKYAFGSDTQLFAEYAHEREYEDDTQDLTMSLNSLPGNRFTLEGYTP
QDHLNRVSLGFSQKLAPELSLRGGYNWRKGEDDTQQSVSLALSLDF
>Q04877
MNPARKKPSLLFSSLLFSSLLFSSLLFSSLLFPSAAQAAGEGNGRGPYVQADLAYAYEHI
THDYPEPTGTKKDKISTVSDYFRNIRTHSIHPRVSVGYDFGGWRIAADYARYRKWNNNKY
SVNIKELLRNDNANSGGNKHLNIKTRKTEHRENGTFHAASSLGLSAVYDFDTGSRFKPYI
GMRVAYGHVRHQVRSVQQETIAVTTYPQNAASSVTTNAPIRKLPHHESRSISSLGFGAVA
GVGIDITPKLTLDAGYRYHNWGRLENTRFKTHEASLGVRYRF
>P0A3U4
MKSVILASIAAMFATSAMAADVVVSEPSAPTAAPVDTFSWTGGYIGINAGYAGGKFKHPF
SSFDKEDNEQVSGSLDVTAGGFVGGVQAGYNWQLDNGVVLGAETDFQGSSVTGSISAGAS
GLEGKAETKVEWFGTVRARLGYTATERLMVYGTGGLAYGKVKSAFNLGDDASALHTWSDK
TKAGWTLGAGAEYAINNNWTLKSEYLYTDLGKRNLVDVDNSFLESKVNFHTVRVGLNYKF

>P0A231
MRAILRGLLPATLLPLAAYAQEATIKEVHDAPAVRGSIIANMLQEHDNPFTLYPYDTNYL
IYTNTSDLNKEAISTYNWSENARKDEVKFQLSLAFPLWRGILGPNSVLGASYTQKSWWQL
SNSKESSPFRETNYEPQLFLGFATDYRFAGWTLRDVEMGYNHDSNGRSDPTSRSWNRLYT

RLMAENGNWLVEVKPWYVIGSTDDNPDITKYMGYYQLKIGYHLGEAVLSAKGQYNWNTGY
GGAEVGLSYPVTKHVRLYTQVYSGYGESLIDYNFNQTRVGVGVMLNDIF
>P45357
MRFSKLSLAIATTLVTANALAQSVELDSINVIATRDPSRFAYTPEKQSKDSLLSKQATSV
AAALEDIPNVDIRGGSRSIAQKPNIRGLSDNRVVQVIDGVRQNFDLAHRGSYFLPMSLIQ
EIEVIKGPSSSLWGSGALGGVVAMRTPNALDLLKNNDKFGVKIRQGYQTANNLSERDVSV
FAANDKFDVLISGFYNNADNLRTGKGNKLNNTAYKQFGGLAKFGWQINDANRVELSHRET
RFKQTAPSNNEVENELTNEQITDQIREFHKPNNGSPPKAKPSQEEFYSGVKTRFGSVSYL
TDQQIPDQSTVFNYYLTPDNPYLNTHIALYNNKTIEKEQRKVSGVKDQTKLTTRGINLRN
SSELSHISFVYGVDYMRDKIRTERGTNNKDAQFRADPYNANSNTTGVYLIAHIPLFGEKL
LLSPSVRYDHYDTSSKTVKYKDNHLSPATKLTWIVTNWLDFTAKYNEAFRAPSMQERFVS
GSHFGTSILGRNEINKFVANPNLRPETAKNKEITANLHFDSLFKQGDKFKIEATYFRNDV
KDFINLKIFNDAKTNTNASASAGAGAGANPNGALLPTKSQYQNITNARLSGIELQAQYQT
ERLTLFTNYGSTKGKDKDSGEALSNIAASKIGVGVNYALVKDKFTVGATVTHYAAQRRVP
KDHSVTYPSYILTDLRATYAPLKGEWKNLRLDFALENLFDRKYQPAFSLMEGTGRNAKIS
AVYSF
>Q04641
MKKFNIKSLTLLIVLLPLIVNANNIDSHLLEQNDIAKYVAQSDTVGSFFERFSALLNYPI
VVSKQAAKKRISGEFDLSNPEEMLEKLTLLVGLIWYKDGNALYIYDSGELISKVILLENI
SLNYLIQYLKDANLYDHRYPIRGNISDKTFYISGPPALVELVANTATLLDKQVSSIGTDK
VNFGVIKLKNTFVSDRTYNMRGEDIVIPGVATVVERLLNNGKALSNRQAQNDPMPPFNIT
QKVSEDSNDFSFSSVTNSSILEDVSLIAYPETNSILVKGNDQQIQIIRDIITQLDVAKRH
IELSLWIIDIDKSELNNLGVNWQGTASFGDSFGASFNMSSSASISTLDGNKFIASVMALN
QKKKANVVSRPVILTQENIPAIFDNNRTFYVSLVGERNSSLEHVTYGTLINVIPRFSSRG
QIEMSLTIEDGTGNSQSNYNYNNENTSVLPEVGRTKISTIARVPQGKSLLIGGYTHETNS
NEIISIPFLSSIPVIGNVFKYKTSNISNIVRVFLIQPREIKESSYYNTAEYKSLISEREI
QKTTQIIPSETTLLEDEKSLVSYLNY
>P0A908
MTKLKLLALGVLIATSAGVAHAEGKFSLGAGVGVVEHPYKDYDTDVYPVPVINYEGDNFW
FRGLGGGYYLWNDATDKLSITAYWSPLYFKAKDSGDHQMRHLDDRKSTMMAGLSYAHFTQ
YGYLRTTLAGDTLDNSNGIVWDMAWLYRYTNGGLTVTPGIGVQWNSENQNEYYYGVSRKE
SARSGLRGYNPNDSWSPYLELSASYNFLGDWSVYGTARYTRLSDEVTDSPMVDKSWTGLI
STGITYKF
>O54339
MKKTIVALAVAAVAATSANAATVYNQDGTKVDVNGSLRLILKKEKNERGDLVDNGSRVSF
KASHDLGEGLSALAYTELRFSKNVPVQVKDQQGEVVREYEVEKLGNNVHVKRLYAGFAYE
GLGTLTFGNQLTIGDDVGLSDYTYFNSGINNLLSSGEKAINFKSAEFNGFTFGGAYVFSA
DADKQALRDGRGFVVAGLYNRKMGDVGFAFEAGYSQKYVKQEVEQNPPAAQKVFKDEKEK
AFMVGAELSYAGLALGVDYAQSKVTNVDGKKRALEVGLNYDLNDRAKVYTDFIWEKEGPK
GDVTRNRTVAVGFGYKLHKQVETFVEAAWGREKDSDGVTTKNNVVGTGLRVHF
>Q57483
MKNIAKVTALALGIALASGYASAEEKIAFINAGYIFQHHPDRQAVADKLDAEFKPVAEKL
AASKKEVDDKIAAARKKVEAKVAALEKDAPRLRQADIQKRQQEINKLGAAEDAELQKLMQ
EQDKKVQEFQAQNEKRQAEERGKLLDSIQTATNNLAKAKGYTYVLDANSIVFAVEGKDIT

EEVLKSIPASEKAQEKK
>P76773
MKKINAIILLSSLTSASVFAGAYVENREAYNLASDQGEVMLRVGYNFDMGAGIMLTNTYN
FQREDELKHGYNEIEGWYPLFKPTDKLTIQPGGLINDKSIGSGGAVYLDVNYKFVPWFNL
TVRNRYNHNNYSSTDLSGELDNNDTYEIGTYWNFKITDKFSYTFEPHYFMRVNDFNSSNG
KDHHWEITNTFRYRINEHWLPYFELRWLDRNVEPYHREQNQIRIGTKYFF
>P0A0V8
MFKVKFYIRHAVLLLCGSLIVGCSAIPSSGPSAKKIVSLGQQSEVQIPEVELIDVNHTVA
QLLYKAQINQSFTQFGDGYASAGTLNIGDVLDIMIWEAPPAVLFGGGLSSMGSGSAHQTK
LPEQLVTARGTVSVPFVGDISVVGKTPGQVQEIIKGRLKKMANQPQVMVRLVQNNAANVS
VIRAGNSVRMPLTAAGERVLDAVAAVGGSTANVQDTNVQLTRGNVVRTVALEDLVANPRQ
NILLRRGDVVTMITNPYTFTSMGAVGRTQEIGFSARGLSLSEAIGRMGGLQDRRSDARGV
FVFRYTPLVELPAERQDKWIAQGYGSEAEIPTVYRVNMADAHSLFSMQRFPVKNKDVLYV
SNAPLAEVQKFLSFVFSPVTSGANSINNLTN
>P08008
MKISSFISTSLPLPTSVSGSSSVGEMSGRSVSQQTSDQYANNLAGRTESPQGSSLASRII
ERLSSVAHSVIGFIQRMFSEGSHKPVVTPAPTPAQMPSPTSFSDSIKQLAAETLPKYMQQ
LNSLDAEMLQKNHDQFATGSGPLRGSITQCQGLMQFCGGELQAEASAILNTPVCGIPFSQ
WGTIGGAASAYVASGVDLTQAANEIKGLAQQMQKLLSLM
>P35823
MFKKTLIAAAIVVGSAAPAFADVVISPNDNTFVTTSLASVTKQPVLDFSTAQQNLTLNFS
EVGDLKNNGFIVLEIQGEGQFNDAEIRQWLSNGFWRRPFTGLLVNPNDHGNFANSGEVND
VRKFFKIISDGTQLTIVHTIDSNGKRLRLALASDVEETINFADAEVELKLNLANQAFKLT
SGSQGTVALTAGALWNASYTADPVATKPLFKLGKLFQLSLTNAGKATALVSEGFLKLNIG
DANISATDFAITNVTTNQTIQRDKVNLTLTGDVSAFKKDANGNLVNKAGASIGWKAAADG
QSATAVLGAGNMAGGVQNALAAFGTLYVAADNTVPVPAVNFNVKAEIQGDSQATYNYFKD
ELADLFILTRDGMKFDTITTGTTSANLIHIRDVSNILPTEGGKIFVTITEYADHAANGRG
EGTVLVTRKALSVTLPSGGAVTLKPADVAADVGASITAGRQARLVFEVETNQGEVAVKKS
NAEGVDIQNGTRGTAPLVDFTL
>P13980
MKHNVKLMAMTAVLSSVLVLSGCGAMSTAIKKRNLEVKTQMSETIWLEPSSQKTVYLQIK
NTSDKDMSGLQAKVTKAVQDKGYTITSSPDSAHYWIQANVLKADKMDLRTAQGFLSQGYE
GAIAGAALGAGITGYNSSSAGATLGVGLAAGLVGMAADAMVEDINYTMVTDIQISEKTTA
SVQTDNVAALKQGTSGYKVQTSTQTGNQHKYQTRIVSSANKVNLKFEEAKPVLEDQLAKS
VANIL
>P0A940
MAMKKLLIASLLFSSATVYGAEGFVVKDIHFEGLQRVAVGAALLSMPVRTGDTVNDEDIS
NTIRALFATGNFEDVRVLRDGDTLLVQVKERPTIASITFSGNKSVKDDMLKQNLEASGVR
VGESLDRTTIADIEKGLEDFYYSVGKYSASVKAVVTPLPRNRVDLKLVFQEGVSAEIQQI
NIVGNHAFTTDELISHFQLRDEVPWWNVVGDRKYQKQKLAGDLETLRSYYLDRGYARFNI
DSTQVSLTPDKKGIYVTVNITEGDQYKLSGVEVSGNLAGHSAEIEQLTKIEPGELYNGTK
VTKMEDDIKKLLGRYGYAYPRVQSMPEINDADKTVKLRVNVDAGNRFYVRKIRFEGNDTS
KDAVLRREMRQMEGAWLGSDLVDQGKERLNRLGFFETVDTDTQRVPGSPDQVDVVYKVKE
RNTGSFNFGIGYGTESGVSFQAGVQQDNWLGTGYAVGINGTKNDYQTYAELSVTNPYFTV

DGVSLGGRLFYNDFQADDADLSDYTNKSYGTDVTLGFPINEYNSLRAGLGYVHNSLSNMQ
PQVAMWRYLYSMGEHPSTSDQDNSFKTDDFTFNYGWTYNKLDRGYFPTDGSRVNLTGKVT
IPGSDNEYYKVTLDTATYVPIDDDHKWVVLGRTRWGYGDGLGGKEMPFYENFYAGGSSTV
RGFQSNTIGPKAVYFPHQASNYDPDYDYECATQDGAKDLCKSDDAVGGNAMAVASLEFIT
PTPFISDKYANSVRTSFFWDMGTVWDTNWDSSQYSGYPDYSDPSNIRMSAGIALQWMSPL
GPLVFSYAQPFKKYDGDKAEQFQFNIGKTW
>P11910
MKKSLFAAALLSLALAACGGEKAAEAPAAEASSTEAPAAEAPAAEAPAAEAAAAEAPAAE
APAAEAPAAEAAATEAPAAEAPAAEAAK
>P44601
MKMRPRYSVIASAVSLGFVLSKSVMALDRPDTGSLNRELEQRQIQSEAKPSGELFNQTAN
SPYTAQYKQGLKFPLKQVQILDRNNQEVVTDELAHILKNYVGKEVSLSDLSNLANEISEF
YRHNNYLVAKAILPPQEIEQGTVKILLLKGNVGEIRLQNHSALSNKFVSRLSNTTVNASE
FILKDELEKFALTINDVPGVNAGLQLSAGKKVGEANLLIKINDAKRFSSYVSVDNQGNKY
TGRYRLAAGTKVSNLNGWGDELKLDLMSSNQANLKNARIDYSSLIDGYSTRFGVTANYLD
YKLGGNFKSLQSQGHSHTLGAYLLHPTIRTPNFRLSTKVSFNHQNLTDKQQAVYVKQKRK
INSLTAGIDGSWNLIKDGTTYFSLSTLFGNLANQTSEKKQYTKEDFQPQSHFTVYNYRLS
HEQILPKSFAFNIGINGQFADKTLESSQKMLLGGLSGVRGHQAGAASVDEGHLIQTEFKH
YLPVFSQSVLVSSLFYDYGLGKYYKNSQFLEKGVKNSVKLQSVGAGLSLSDAGSYAINVS
VAKPLDNNINNADKHQFWLSMIKTF
>P76045
MKKLLPCTALVMCAGMACAQAEERNDWHFNIGAMYEIENVEGYGEDMDGLAEPSVYFNAA
NGPWRIALAYYQEGPVDYSAGKRGTWFDRPELEVHYQFLENDDFSFGLTGGFRNYGYHYV
DEPGKDTANMQRWKIAPDWDVKLTDDLRFNGWLSMYKFANDLNTTGYADTRVETETGLQY
TFNETVALRVNYYLERGFNMDDSRNNGEFSTQEIRAYLPLTLGNHSVTPYTRIGLDRWSN
WDWQDDIEREGHDFNRVGLFYGYDFQNGLSVSLEYAFEWQDHDEGDSDKFHYAGVGVNYS
F
>A5F661
MAVLCPARVSVAENKKFKLHTLSAMMMGLFTGSFAYAETQNTSNQEQEMPVLVVIGEKTQ
RSIYETSASVEVFDQDTIERTPGATEIDDLLQLIPNLVDSGQSNNMPTIRGIDGSGPSVG
GLASFAGTSPRLNMSIDGRSLTYSEIAFGPRSLWDMQQVEIYLGPQSYIQGRNTSAGAIV
MKSNDPTHHFESAVKAGIGESDYSQTAGMISAPIIQDELAFRLSFDQQKRDSFVDLAAFE
PAGDPKKIEMNSVRGKLLYEPSALDGFKTTLTLSHMDSRGPQTENINVAGNEAFRPVYET
ASFTTAWDIIWHLNDLFTFENNLVYADFSYDRYTNPNSRGDFNTDGKEFHIEPLLRYIAL
DGSVNTLIGARYYQSSQDDMYIDAASAYPMDGRTKAKSVFAEVTYALTPSINVNLAGRFE
REQVKRNVSHPRYKLDYDETSSVFLPKLDVAYTPVQGQTYGIKAAKGYNASGAGLAFNSM
QFTGFRPYEFEQESIWNYEFYTRHRFSHSVEVLTNLFYNDFDSMQMTQTTSSGDVFIANL
DEASTYGAEIGSRWYATSSLELFANLGLLKTEFKETTGNTKELPRAPKMSANVGLLYDFG
QGFEFSSNAAYTGSYFSESGNSEKFAIDSYWVANAQLAYVFEHGRATLYATNLLDSDKTT
LYLSTNNTLDQLKQQPRMIGASVQLNF
>Q00595
MSFSNYKVIAMPVLVANFVLGAATAWANENYPAKSAGYNQGDWVASFNFSKVYVGEELGD
LNVGGGALPNADVSIGNDTTLTFDIAYFVSSNIAVDFFVGVPARAKFQGEKSISSLGRVS
EVDYGPAILSLQYHYDSFERLYPYVGVGVGRVLFFDKTDGALSSFDIKDKWAPAFQVGLR

YDLGNSWMLNSDVRYIPFKTDVTGTLGPVPVSTKIEVDPFILSLGASYVF
>P40710
MVKKAIVTAMAVISLFTLMGCNNRAEVDTLSPAQAAELKPMPQSWRGVLPCADCEGIETS
LFLEKDGTWVMNERYLGAREEPSSFASYGTWARTADKLVLTDSKGEKSYYRAKGDALEML
DREGNPIESQFNYTLEAAQSSLPMTPMTLRGMYFYMADAATFTDCATGKRFMVANNAELE
RSYLAARGHSEKPVLLSVEGHFTLEGNPDTGAPTKVLAPDTAGKFYPNQDCSSLGQ
>P55127
MNEGEVVLTPEQIQTLRGYASRGDTYGGWRYLANLGDRYADNAAAIVGKDTNLNGLNLWM
KKGVENLWDDTVGKKTRLEKFDRVALQHFSQYVDLINKNNGRLPNTSEIERSYYKAVTYH
GVSSSAAIDLVINRSLPDMADGYWALGLGIEAERIHNEQAVNNPNGSERDNRKQLISALD
KGFDGSFKEKHFTFLQSVMMDLTKLGVEYTIDGWQKIGGWGNGIINDLYKSVVKREWTGI
FEIVNNNIKQGNEAFKNEINSLVHDMKAAGKEFGDDLNTQWNNLTQAAEIIYNDIVDNTS
QGIEKGVKAIKELSEKMKNAASDLADGSAEKAKQVVEDLAQAAKEAYENAKSTAEKAAQA
AREFFKGLPSFKDLAEKFRDLFPNPEGWIDDGHQCFAPWVKETKKRNGKYHVYDPLALDL
DGDGIETVATKGFSGSLFDHNRDGIRTATGWVAADDGLLVRDLNGNGIIDNGAELFGDNT
KLADGSFAKHGYAALAELDSNGDNIINAADAAFQTLRVWQDLNQDGISQANELRTLEELG
IQSLDLAYKDVNKNLGNGNTLAQQGSYTKTDGTTAKMGDLLLAADNLHSRFKDKVELTAE
QAKAANLAGIGRLRDLREAAALSGDLANMLKAYSAAETKEAQLALLDNLIHKWAETDSNW
GKKSPMRLSTDWTQTANEGIALTPSQVAQLKKNALVSLSDKAKAAIDAARDRIAVLDAYT
GQDSSTLYYMSEEDALNIVKVTNDTYDHLAKNIYQNLLFQTRLQPYLNQISFKMENDTFT
LDFSGLVQAFNHVKETNPQKAFVDLAEMLAYGELRSWYEGRRLMADYVEEAKKAGKFEDY
QKVLGQETVALLAKTSGTQADDILQNVGFGHNKNVSLYGNDGNDTLIGGAGNDYLEGGSG
SDTYVFGKGFGQDTVYNYDYATGRKDIIRFTDGITADMLTFTREGNHLLIKAKDDSGQVT
VQSYFQNDGSGAYRIDEIHFDNGKVLDVATVKELVQQSTDGSDRLYAYQSGNTLNGGLGD
DYLYGADGDDLLNGDAGNDSIYSGNGNDTLNGGEGNDALYGYNGNDALNGGEGNDHLNGE
DGNDTLIGGAGNDYLEGGSGSDTYVFGKGFGQDTVYNYDYATGRKDIIRFTDGITADMLT
FTREGNHLLIKAKDGSGQVTVQSYFQNDGSGAYRIDEIHFDNGKVLDVATVKELVQQSTD
GSDRLYAYQSGNTLNGGLGDDYLYGADGDDLLNGDAGNDSIYSGNGNDTLDGGEGNDALY
GYNGNDALNGGEGNDHLNGEDGNDTLIGGAGNDYLEGGSGSDTYVFGKGFGQDTVYNYDY
ATGRKDIIRFTDGITADMLTFTREGNHLLIKAKDDSGQVTVQSYFQNDGSGAYRIDEIHF
DNGKVLDVATVKELVQQSTDGSDRLYAYQSGSTLNGGLGDDYLYGADGDDLLNGDAGNDS
IYSGNGNDTLDGGEGNDALYGYNGNDALNGGEGNDHLNGEDGNDTLIGGAGNDYLEGGSG
SDTYVFGKGFGQDTVYNYDYATGRKDIIRFTDGITADMLTFTREGNHLLIKAKDGSGQVT
VQSYFQNDGSGAYRIDEIHFDNGKVLDVATVKKLVQQSTDGSDRLYAYQSGNTLNGGLGD
DYLYGADGDDLLNGDAGNDSIYSGNGNDTLNGGEGNDALYGYNGNDVLNGGEGNDHLNGE
DGNDTLIGGAGNDYLEGGSGSDTYVFGKGFGQDTVYNYHVDKNSDTMHFKGFKAADVHFI
RSGSDLVLSASEQDNVRISGFFYGENHRVDTFVFDDAAISNPDFAKYINAGNNLVQSMSV
FGSNTAATGGNVDANTQSVQQPLLVTPSA
>Q02937
MNIATKLMASLVASVVLTACSGGGSSGSSSKPNSELTPKVDMSAPKAEQPKKEEVPQADN
SKAEEPKEMAPQVDSPKAEEPKNMAPQMGNPKLNDPQVMAPKMDNPQKDAPKGEELSKDK
SNAEILKELGVKDINSGIINNADVVLNLKIDEKDHITVVLDKGKINRNHLKVTNTISAQD
IKTLKDSSGKLLGYYGYMQLNQVRQDENYSDEKVSLNEYYLLSMNDADKIRPTKSISYKG
DMFYSYKDVGNQKLKASVEASYDDVTKKVSMKVFGENNDYWKLGEFGRTNLLENQVTGAK

VGEDGTIINGTLYSKIDNFPLKLTPDANFSGGIFGKNGEVLAGSAISEKWQGVIGATATT
KEDKK
>P31243
EVKLSGDARMGVMYNGDDWNFSSRSRVLFTMSGTTDSGLEFGASFKAHESVGAETGEDGT
VFLSGAFGKIEMGDALGASEALFGDLYEVGYTDLDDRGGNDIPYLTGDERLTAEDNPVLL
YTYSAGAFSVAASMSDGKVGETSEDDAQEMAVAAAYTFGNYTVGLGYEKIDSPDTALMAD
MEQLELAAIAKFGATNVKAYYADGELDRDFARAVFDLTPVAAAATAVDHKAYGLSVDSTF
GATTVGGYVQVLDIDTIDDVTYYGLGASYDLGGGASIVGGIADNDLPNSDMVADLGVKFK
F
>P75780
MENNRNFPARQFHSLTFFAGLCIGITPVAQALAAEGQTNADDTLVVEASTPSLYAPQQSA
DPKFSRPVADTTRTMTVISEQVIKDQGATNLTDALKNVPGVGAFFAGENGNSTTGDAIYM
RGADTSNSIYIDGIRDIGSVSRDTFNTEQVEVIKGPSGTDYGRSAPTGSINMISKQPRND
SGIDASASIGSAWFRRGTLDVNQVIGDTTAVRLNVMGEKTHDAGRDKVKNERYGVAPSVA
FGLGTANRLYLNYLHVTQHNTPDGGIPTIGLPGYSAPSAGTAALNHSGKVDTHNFYGTDS
DYDDSTTDTATMRFEHDINDNTTIRNTTRWSRVKQDYLMTAIMGGASNITQPTSDVNSWT
WSRTANTKDVSNKILTNQTNLTSTFYTGSIGHDVSTGVEFTRETQTNYGVNPVTLPAVNI
YHPDSSIHPGGLTRNGANANGQTDTFAIYAFDTLQITRDFELNGGIRLDNYHTEYDSATA
CGGSGRGAITCPTGVAKGSPVTTVDTAKSGNLMNWKAGALYHLTENGNVYINYAVSQQPP
GGNNFALAQSGSGNSANRTDFKPQKANTSEIGTKWQVLDKRLLLTAALFRTDIENEVEQN
DDGTYSQYGKKRVEGYEISVAGNITPAWQVIGGYTQQKATIKNGKDVAQDGSSSLPYTPE
HAFTLWSQYQATDDISVGAGARYIGSMHKGSDGAVGTPAFTEGYWVADAKLGYRVNRNLD
FQLNVYNLFDTDYVASINKSGYRYHPGEPRTFLLTANMHF
>P69856
MKKAKILSGVLLLCFSSPLISQAATLDVRGGYRSGSHAYETRLKVSEGWQNGWWASMESN
TWNTIHDNKKENAALNDVQVEVNYAIKLDDQWTVRPGMLTHFSSNGTRYGPYVKLSWDAT
KDLNFGIRYRYDWKAYRQQDLSGDMSRDNVHRWDGYVTYHINSDFTFAWQTTLYSKQNDY
RYANHKKWATENAFVLQYHMTPDITPYIEYDYLDRQGVYNGRDNLSENSYRIGVSFKL
>P17778
MFINPRNVSNTFLQEPLRHSSNLTEMPVEAENVKSKTEYYNAWSEWERNAPPGNGEQREM
AVSRLRDCLDRQAHELELNNLGLSSLPELPPHLESLVASCNSLTELPELPQSLKSLLVDN
NNLKALSDLPPLLEYLGVSNNQLEKLPELQNSSFLKIIDVDNNSLKKLPDLPPSLEFIAA
GNNQLEELPELQNLPFLTAIYADNNSLKKLPDLPLSLESIVAGNNILEELPELQNLPFLT
TIYADNNLLKTLPDLPPSLEALNVRDNYLTDLPELPQSLTFLDVSENIFSGLSELPPNLY
YLNASSNEIRSLCDLPPSLEELNVSNNKLIELPALPPRLERLIASFNHLAEVPELPQNLK
QLHVEYNPLREFPDIPESVEDLRMNSERVVDPYEFAHETTDKLEDDVFE
>P06971
MARSKTAQPKHSLRKIAVVVATAVSGMSVYAQAAVEPKEDTITVTAAPAPQESAWGPAAT
IAARQSATGTKTDTPIQKVPQSISVVTAEEMALHQPKSVKEALSYTPGVSVGTRGASNTY
DHLIIRGFAAEGQSQNNYLNGLKLQGNFYNDAVIDPYMLERAEIMRGPVSVLYGKSSPGG
LLNMVSKRPTTEPLKEVQFKAGTDSLFQTGFDFSDSLDDDGVYSYRLTGLARSANAQQKG
SEEQRYAIAPAFTWRPDDKTNFTFLSYFQNEPETGYYGWLPKEGTVEPLPNGKRLPTDFN
EGAKNNTYSRNEKMVGYSFDHEFNDTFTVRQNLRFAENKTSQNSVYGYGVCSDPANAYSK
QCAALAPADKGHYLARKYVVDDEKLQNFSVDTQLQSKFATGDIDHTLLTGVDFMRMRNDI

NAWFGYDDSVPLLNLYNPVNTDFDFNAKDPANSGPYRILNKQKQTGVYVQDQAQWDKVLV
TLGGRYDWADQESLNRVAGTTDKRDDKQFTWRGGVNYLFDNGVTPYFSYSESFEPSSQVG
KDGNIFAPSKGKQYEVGVKYVPEDRPIVVTGAVYNLTKTNNLMADPEGSFFSVEGGEIRA
RGVEIEAKAALSASVNVVGSYTYTDAEYTTDTTYKGNTPAQVPKHMASLWADYTFFDGPL
SGLTLGTGGRYTGSSYGDPANSFKVGSYTVVDALVRYDLARVGMAGSNVALHVNNLFDRE
YVASCFNTYGCFWGAERQVVATATFRF
>P23988
MKNIILSTLVITTSVLVVNVAQADTNAFSVGYAQSKVQDFKNIRGVNVKYRYEDDSPVSF
ISSLSYLYGDRQASGSVEPEGIHYHDKFEVKYGSLMVGPAYRLSDNFSLYALAGVGTVKA
TFKEHSTQDGDSFSNKISSRKTGFAWGAGVQMNPLENIVVDVGYEGSNISSTKINGFNVG
VGYRF
>Q08017
MSASRFMLRPLTRALLMHGATRTRLAGTGLGLALTLTAAPYVQAQEWTLNIPSQPLAQAL
QTLGQQTSLQIIYSPESLQGLRSTALNGRYQDDESLKAMLNGTGIRYQRDGNTVTVLGPA
TGSAMELAPTNVNASRLGATTEGSNSYTTGGVTIGKGVHSLKETPQSVTVMTRKMLDDQN
LNTIEQVMEKTPGITVYDSPMGGKYFYSRGFRMSGQYQYDGVPLDIGSSYVQADSFNSDM
AIYDRVEVLRGAAGMMKGAGGTAGGVNFVRKRGQDTAHTQLSLSAGTWDNYRGQVDTGGP
LNDSGTIRGRAVVTEQTRQYFYDVGSRKDQIYYGALDFDLSPDTTLGLGFAWEDVDATPC
WGGLPRYADGSDLHLKRSTCLNTAWNNQRSKRATYFADLKHQFNDDWSLKVAGVYSRNTQ
DMEYAFPSGAVPVGATATNTLMLGSIYDYDQRDYGFDAYVDGKFDAFGQQHELTIGANAS
RSHKDDFYAVAALPQRQNVLDPNHHIPQPDESYYLANASRGGPVDMHIKQYGAYSIARLK
LADPLTLVLGSRVSWYKSDTDSVQYFRGEGTQVDTKSTETGQVTPFAGVLFDLNDNLTAY
ASYTDIFTPQGAYKTIDGSTLKPLVGQSYELGIKGEWFDGRLNSTFNLFRTLQKDAAQDD
PRCEDSSCSINSGKVRAQGFEAEVSGEVIDRLQLLAGYTYTQTKVLEDADATQDGVVYNS
YVPRHLLRVWGDYSLSGPLDRVTIGAGVNAQTGNYRTSPIGGDNIDGAGYAVWNGRIGYR
IDDTWSVALNGNNLFDKRYYSTIGTEGFGNFYGDPRNFVMSVKADF
>P07211
MKAYLALISAAVIGLAACSQEPAAPAAEATPAGEAPASEAPAAEAAPADAAEAPAAGNCA
ATVESNDNMQFNTKDIQVSKACKEFTITLKHTGTQPKASMGHNLVIAKAEDMDGVFKDGV
GAADTDYVKPDDARVVAHTKLIGGGEESSLTLDPAKLADGDYKFACTFPGHGALMNGKVT
LVD

(3) $\mathbb{S}_3$: 410 cytoplasm proteins

>P0AGJ9
MASSNLIKQLQERGLVAQVTDEEALAERLAQGPIALYCGFDPTADSLHLGHLVPLLCLKR
FQQAGHKPVALVGGATGLIGDPSFKAAERKLNTEETVQEWVDKIRKQVAPFLDFDCGENS
AIAANNYDWFGNMNVLTFLRDIGKHFSVNQMINKEAVKQRLNREDQGISFTEFSYNLLQG
YDFACLNKQYGVVLQIGGSDQWGNITSGIDLTRRLHQNQVFGLTVPLITKADGTKFGKTE
GGAVWLDPKKTSPYKFYQFWINTADADVYRFLKFFTFMSIEEINALEEEDKNSGKAPRAQ
YVLAEQVTRLVHGEEGLQAAKRITECLFSGSLSALSEADFEQLAQDGVPMVEMEKGADLM
QALVDSELQPSRGQARKTIASNAITINGEKQSDPEYFFKEEDRLFGRFTLLRRGKKNYCL
ICWK
>P42321
MEKKKLTTAAGAPVVDNNNVITAGPRGPMLLQDVWFLEKLAHFDREVIPERRMHAKGSGA

FGTFTVTHDITKYTRAKIFSEVGKKTEMFARFSTVAGERGAADAERDIRGFALKFYTEEG
NWDMVGNNTPVFYLRDPLKFPDLNHIVKRDPRTNMRNMAYKWDFFSHLPESLHQLTIDMS
DRGLPLSYRFVHGFGSHTYSFINKDNERFWVKFHFRCQQGIKNLMDDEAEALVGKDRESS
QRDLFEAIERGDYPRWKLQIQIMPEKEASTVPYNPFDLTKVWPHADYPLMDVGYFELNRN
PDNYFSDVEQAAFSPANIVPGISFSPDKMLQGRLFSYGDAHRYRLGVNHHQIPVNAPKCP
FHNYHRDGAMRVDGNSGNGITYEPNSGGVFQEQPDFKEPPLSIEGAADHWNHREDEDYFS
QPRALYELLSDDEHQRMFARIAGELSQASKETQQRQIDLFTKVHPEYGAGVEKAIKVLEG
KDAK
>P0A944
MNTISSLETTDLPAAYHIEQRAHAFPWSEKTFASNQGERYLNFQLTQNGKMAAFAITQVV
LDEATLFNIAVDPDYQRQGLGRALLEHLIDELEKRGVATLWLEVRASNAAAIALYESLGF
NEATIRRNYYPTTDGREDAIIMALPISM
>P36662
MTTLTAQQIACVYAWLAQLFSRELDDEQLTQIASAQMAEWFSLLKSEPPLTAAVNELENR
IATLTVRDDARLELAADFCGLFLMTDKQAALPYASAYKQDEQEIKRLLVEAGMETSGNFN
EPADHLAIYLELLSHLHFSLGEGTVPARRIDSLRQKTLTALWQWLPEFVARCRQYDSFGF
YAALSQLLLVLVECDHQNR
>P0ACC7
MLNNAMSVVILAAGKGTRMYSDLPKVLHTLAGKAMVQHVIDAANELGAAHVHLVYGHGGD
LLKQALKDDNLNWVLQAEQLGTGHAMQQAAPFFADDEDILMLYGDVPLISVETLQRLRDA
KPQGGIGLLTVKLDDPTGYGRITRENGKVTGIVEHKDATDEQRQIQEINTGILIANGADM
KRWLAKLTNNNAQGEYYITDIIALAYQEGREIVAVHPQRLSEVEGVNNRLQLSRLERVYQ
SEQAEKLLLAGVMLRDPARFDLRGTLTHGRDVEIDTNVIIEGNVTLGHRVKIGTGCVIKN
SVIGDDCEISPYTVVEDANLAAACTIGPFARLRPGAELLEGAHVGNFVEMKKARLGKGSK
AGHLTYLGDAEIGDNVNIGAGTITCNYDGANKFKTIIGDDVFVGSDTQLVAPVTVGKGAT
IAAGTTVTRNVGENALAISRVPQTQKEGWRRPVKKK
>P74863
MSEEGFMLAVLKGIPLIQDIRAEGNSRSWIMTIDGHPARGEIFSEAFSISLFLNDLESLP
KPCLAYVTLLLAAHPDVHDYAIQLTADGGWLNGYYTTSSSSELIAIEIEKHLALTCILKN
VIRNHHKLYSGGV
>P0A9K1
MVTSCTGHVLDNQRATTRGVFSSGSHLVTLHFQPHPFFSCVTDAVNGARSRFSAFYPKAN
YGLQGSQPSDVRAHNRAANGACDEYKQLKVLSMGRQKAVIKARREAKRVLRRDSRSHKQR
EEESVTSLVQMGGVEAIGMARDSRDTSPILARNEAQLHYLKAIESKQLIFATGEAGCGKT
WISAAKAAEALIHKDVDRIIVTRPVLQADEDLGFLPGDIAEKFAPYFRPVYDVLVRRLGA
SFMQYCLRPEIGKVEIAPFAYMRGRTFENAVVILDEAQNVTAAQMKMFLTRLGENVTVIV
NGDITQCDLPRGVCSGLSDALERFEEDEMVGIVRFGKEDCVRSALCQRTLHAYS
>Q02286
MNPDNPLLALRDKISAVDKKLLTLLAERRLLAVEVAQAKLATHRPIRDVERERALLENLI
VLGKAHNLDAHYITRLFQLVIEDSVLTQQALLQKNLNHPHAHAARIAFLGPKGSYSHLAA
RNYASRHFDSMVECGCLKFHDIIKQVENGVADYAVMPIENTSSGSINDVYDLLQQTSLSI
VGELTLPIDHCVLVNGPTDLQQIETVYSHPQPFQQCSQFINRFPHWKIEYTESTAAAMEK
VAALNSPKVAALGSEAGGELYQLQVLERNLANQQQNHTRFIVLARKAIEVSDQVPAKTTL
IMATGQQAGALVDALLVLRQHNLIMSKLESRPINGNPWEEMFYIDVQGNLQSERMQQALQ

ELQTMTRSLKVLGCYPSENVVPAEPGR
>P06110
MTGMSNVSKLAGEPSGQEFLVFTLGNEEYGIDILKVQEIRGYDQVTRIANTPAFIKGVTN
LRGVIVPIVDLRVKFCEGDVEYDDNTVVIVLNLGQRVVGIVVDGVSDVLSLTAEQIRPAP
EFAVTLSTEYLTGLGALGERMLILVNIEKLLNSEEMALLDIAASHVA
>P14916
MKLTPKELDKLMLHYAGELAKKRKEKGIKLNYVEAVALISAHIMEEARAGKKTAAELMQE
GRTLLKPDDVMDGVASMIHEVGIEAMFPDGTKLVTVHTPIEANGKLVPGELFLKNEDITI
NEGKKAVSVKVKNVGDRPVQIGSHFHFFEVNRCLDFDREKTFGKRLDIASGTAVRFEPGE
EKSVELIDIGGNRRIFGFNALVDRQADNESKKIALHRAKERGFHGAKSDDNYVKTIKE
>P44521
MKLYGLIGACSFVPHVALEWVKIRENADYEFEPVTRELIKSPEFLSLNPRGAVPVLVDGD
LVLSQNQAILHYLDELYPNSKLFGSKTVRDKAKAARWLAFFNSDVHKSFVPLFRLPNYAK
DNETLAHTIRQQAVEQILDQLAVANEHLESHIYFGENISVADAYLYIMLNWCKAVKIDFS
HLTQLSAFMQRVETDQAVENVRKSEELKV
>P0A796
MIKKIGVLTSGGDAPGMNAAIRGVVRSALTEGLEVMGIYDGYLGLYEDRMVQLDRYSVSD
MINRGGTFLGSARFPEFRDENIRAVAIENLKKRGIDALVVIGGDGSYMGAMRLTEMGFPC
IGLPGTIDNDIKGTDYTIGFFTALSTVVEAIDRLRDTSSSHQRISVVEVMGRYCGDLTLA
AAIAGGCEFVVVPEVEFSREDLVNEIKAGIAKGKKHAIVAITEHMCDVDELAHFIEKETG
RETRATVLGHIQRGGSPVPYDRILASRMGAYAIDLLLAGYGGRCVGIQNEQLVHHDIIDA
IENMKRPFKGDWLDCAKKLY
>Q9A5I5
MSARILVVDDIEANVRLLEAKLTAEYYEVSTAMDGPTALAMAARDLPDIILLDVMMPGMD
GFTVCRKLKDDPTTRHIPVVLITALDGRGDRIQGLESGASDFLTKPIDDVMLFARVRSLT
RFKLVIDELRQREASGRRMGVIAGAAARLDGLGGRVLIVDDNERQAQRVAAELGVEHRPV
IESDPEKAKISAGGPVDLVIVNAAAKNFDGLRFTAALRSEERTRQLPVLAMVDPDDRGRM
VKALEIGVNDILSRPIDPQELSARVKTQIQRKRYTDYLRNNLDHSLELAVTDQLTGLHNR
RYMTGQLDSLVKRATLGGDPVSALLIDIDFFKKINDTFGHDIGDEVLREFALRLASNVRA
IDLPCRYGGEEFVVIMPDTALADALRIAERIRMHVSGSPFTVAHGREMLNVTISIGVSAT
AGEGDTPEALLKRADEGVYQAKASGRNAVVGKAA
>O87386
MSSYRLPKRGLVDRNVPLSFTFDGRPMQGLEGDTLASALLANGRMLVGRSFKYHRPRGIL
TAGAAEPNALVTVGRGGRAEPNTRATMQELYEGLEARSQNRWPSLAFDIGALNGLLSPFL
GAGFYYKTFMWPAPLWEKLYEPVIRRAAGLGKASYEADPDAYEKSWAHCDLLVIGAGPTG
LAAALTAGRAGARVILVDEGSLPGGSLLSDTATIDGKAAADFARDTSDELRSMPNVQVLV
RTTAFGWYDGNVFGAVERVQKHVREPASHLPVERLWRIVAGKALLATGAEERPLVFGGND
RPGVMMAGAMRAYLNRYGVAPGRTPAIFTTNDTGYTLAQELEAAGVDVVAIVDSRPAAGV
DYRGKARLVREAVVCGTKGGKAISAIEVHHGGRTETIAVDALAMAGGFDPIIHLACHRGG
KPVWSAEKAAFLAPGSLKGLEVAGGAAATTGLAACLGEGAARAEAIVRELGLPCPPVAVV
KVESEEGIRSPAPLWSIPGIKDKAFVDFQNDVHLKDIGLAVREGYSHVELAKRYTTSGMA
TDQGKLSNVNAIGLIAKARGVSPAEVGTTTFRPFYTPVSFGALTGAHTGHHFQPVRKSPL
HDWAKKHGAVFVETGLWYRSSWFPRSGERTWRESVEREVLNVRKNAGLCDVSMLGKIEIT
GSDAAEFLNRVYCNAFLKLPVGKARYGLMLREDGFIYDDGTTSRLEENRFFMTTTTAYAA

GVMNHLEFCAQVLWPQLDVRLASITDQWAQMAIAGPKARMILQKIVDEDISDAAFPFLAA
KEVSLFGGALHGCLFRISFSGELAYELAVPAGYGESIADALLEAGKDHGIMPYGVETLSV
LRIEKGHVTHNEINGTIVPADLGFGKMVSAGKPDFVGKAMLQREGLTAPDRPQLVGVVPL
DPQQSFRSGSHILAKGAAATLENDEGYVTSSAYSPHVGSTIALALVRNGRNRHGEEVLVW
SGLHGESTPARLCNPVFFDPQNERLHV
>P29930
MSDETTVGGEAPAEKDDARHAMKMAKKKAAREKIMATKTDEKGLIIVNTGKGKGKSTAGF
GMIFRHIAHGMPCAVVQFIKGAMATGERELIEKHFGDVCQFYTLGEGFTWETQDRARDVA
MAEKAWEKAKELIRDERNSMVLLDEINIALRYDYIDVAEVVRFLKEEKPHMTHVVLTGRN
AKEDLIEVADLVTEMELIKHPFRSGIKAQQGVEF
>P28894
MAKSARTKTARPATRTETDSFGPIEVPSDRYWGAQTERSRQNFRIGTDRMPISLVHALGI
VKLAAAQSNRELGLLDQRRASAIIRAAREVIDGSLDDHFPLVVWQTGSGTQTNMNLNEVI
ANRANELLGGELGAKKPVHPNDHVNMSQSSNDSFPTAMHIAAASRITADLVPALGELLRA
LRKKEKEFAKIVKIGRTHTQDATPLTLGQEFSGYAAQVESGIARLKVAVKELYPLAQGGT
AVGTGLNAKPRFARLFAKHVAGITKLPFTSAANKFEALASNDAYVLAHGAISSVATGLFK
IANDIRLLGSGPRSGLGELILPENEPGSSIMPGKVNPTQCEAMTMVCCQVFGNHTAITVA
GSQGHFELNVYKPVLAYNMLHSIRLMADAARSFTEHCVSGIRADEKRISELMQRSLMLVT
ALAPKIGYDNAAKVAKTAHANGTTLKEEALRLGFVTADEFDRLVQPEKMTKPG
>P31658
MTVQTSKNPQVDIAEDNAFFPSEYSLSQYTSPVSDLDGVDYPKPYRGKHKILVIAADERY
LPTDNGKLFSTGNHPIETLLPLYHLHAAGFEFEVATISGLMTKFEYWAMPHKDEKVMPFF
EQHKSLFRNPKKLADVVASLNADSEYAAIFVPGGHGALIGLPESQDVAAALQWAIKNDRF
VISLCHGPAAFLALRHGDNPLNGYSICAFPDAADKQTPEIGYMPGHLTWYFGEELKKMGM
NIINDDITGRVHKDRKLLTGDSPFAANALGKLAAQEMLAAYAG
>P04772
MTKYKLEYIWLDGYTPTPNLRGKTQIKEFASFPTLEQLPLWGFDGSSTQQAEGHSSDCVL
KPVAVFPDAARTNGVLVMCEVMMPDGKTPHASNKRATILDDAGAWFGFEQEYFFYKDGRP
LGFPTSGYPAPQGPYYTGVGFSNVGDVARKIVEEHLDLCLAAGINHEGINAEVAKGQWEF
QIFGKGSKKAADEMWMARYLMLRLTEKYGIDIEFHCKPLGDTDWNGSGMHANFSTEYMRT
VGGKEYFEALMAAFDKNLMDHIAVYGPDNDKRLTGKHETAPWNKFSYGVADRGASIRVPH
SFVNNGYKGYLEDRRPNSQGDPYQIASQILKTISSVPTEKKAVA
>P0A7D4
MGNNVVVLGTQWGDEGKGKIVDLLTERAKYVVRYQGGHNAGHTLVINGEKTVLHLIPSGI
LRENVTSIIGNGVVLSPAALMKEMKELEDRGIPVRERLLLSEACPLILDYHVALDNAREK
ARGAKAIGTTGRGIGPAYEDKVARRGLRVGDLFDKETFAEKLKEVMEYHNFQLVNYYKAE
AVDYQKVLDDTMAVADILTSMVVDVSDLLDQARQRGDFVMFEGAQGTLLDIDHGTYPYVT
SSNTTAGGVATGSGLGPRYVDYVLGILKAYSTRVGAGPFPTELFDETGEFLCKQGNEFGA
TTGRRRRTGWLDTVAVRRAVQLNSLSGFCLTKLDVLDGLKEVKLCVAYRMPDGREVTTTP
LAADDWKGVEPIYETMPGWSESTFGVKDRSGLPQAALNYIKRIEELTGVPIDIISTGPDR
TETMILRDPFDA
>P0A948
MFGYRSNVPKVRLTTDRLVVRLVHDRDAWRLADYYAENRHFLKPWEPVRDESHCYPSGWQ
ARLGMINEFHKQGSAFYFGLFDPDEKEIIGVANFSNVVRGSFHACYLGYSIGQKWQGKGL

MFEALTAAIRYMQRTQHIHRIMANYMPHNKRSGDLLARLGFEKEGYAKDYLLIDGQWRDH
VLTALTTPDWTPGR
>P27888
MLNINFVNEESSTNQGLIVFIDEQLKLNNNLIALDQQHYELISKTIQNKLQFSGNYGQIT
VVPSVIKSCAVKYLIIVGLGNVEKLTEAKIEELGGKILQHATCAKIATIGLKIINRINRF
TSPTFTSLIASGAFLASYRFHKYKTTLKEVEKFAVESIEILTDNNSEAMKLFEVKKLIAE
AVFFTRDISNEPSNIKTPQVYAERIVEILEPLGVNIDVIGEHDIKNLGMGALLGVGQGSQ
NESKLVVMEYKGGSRDDSTLALVGKGVIFDTGGISLKPSSNMHLMRYDMAGSAAVVGTII
ALASQKVPVNVVGVVGLVENMQSGNAQRPGDVVVTMSGQTAEVLNTDAEGRLVLADTVWY
VQEKFNPKCVIDVATLTGAITVALGSTYAGCFSNNDELADKLIKAGEAVNEKLWRMPLHD
DYDAMINSDIADIANIGNVPGAAGSCTAAHFIKRFIKDGVDWAHLDIAGVANSNNASALC
PKGAVGYGVRLLEKFIKEYN
>Q56973
MQNLLKNLAASLGRKPFVADKQGVYRLTIDKHLVMLAPHGSELVLRTPIDAPMLREGNNV
NVTLLRSLMQQALAWAKRYPQTLVLDDCGQLVLEARLRLQELDTHGLQEVINKQLALLEH
LIPQLTPFSVASRVGWN
>P43859
MSEKYVVTWDMFQMHARRLSERLLPASQWKGIIAVSRGGLFPAAVLARELGLRHIETVCI
ASYHDHNNQGELQVLHAAQVPNGGEGFIVVDDLVDTGNTARAIRQMYPNAKFVTVFAKPA
GAELVDDYVIDIPQNTWIEQPWDLGLTFVPPLSRK
>P80193
NAIADYRTFPLISPLASAASFASGVSVTWADGRVSPFHNLWLRDNCPCGDCVYEVTREQV
FLVADVPEDIQVQAVTIGDDGRLVVQWDDGHASAYHPGWLRAHAYDAQSLAEREAARPHK
HRWMQGLSLPVYDHGAVMQDDDTLLEWLLAVRDVGLTQLHGVPTEPGALIPLAKRISFIR
ESNFGVLFDVRSKADADSNAYTAFNLPLHTDLPTRELQPGLQFLHCLVNDATGGNSTFVD
GFAIAEALRIEAPAAYRLLCETPVEFRNKDRHSDYRCTAPVIALDSSGEVREIRLANFLR
APFQMDAQRMPDYYLAYRRFIQMTREPRFCFTRRLEAGQLWCFDNRRVLHARDAFDPASG
DRHFQGCYVDRDELLSRILVLQR
>P61380
MSWIEPIISHFCQDLGVPTSSPLSPLIQLEMAQSGTLQLEQHGATLTLWLARSLAWHRCE
DAMVKALTLTAAQKSGALPLRAGWLGESQLVLFVSLDERSLTLPLLHQAFEQLLRLQQEV
LAP
>A1WCP8
MQDKYNHTEVERAAHAHWNANDAYRVTEDQAKPKFYACSMLPYPSGKLHMGHVRNYTIND
MLTRSLRMKGHNVLMPMGWDAFGLPAENAALKNGVPPAQWTYDNIAYMKKQMQAMGLAID
WSREIATCDPDYYKWNQWLFLKMLDKGIAYRKTQVVNWDPVDQTVLANEQVIDGRGWRTG
ALVEKREIPGYYLKITDYAQELLDHVQIGNEKATLTGWPDKVRLMQENWIGKSAGVRFAF
PHDIRNAAGERIQDGKLYVFTTRADTIMGVTFCAVAPEHPLAQHAAASNAALAAFIEECK
KGGTTEAELALKEKEGMPTGLFVTHPLTGEQVEVWVGNYVLMSYGDGAVMGVPAHDERDF
AFALKYQLPIKQVVLVDGETFDFHQWQDWYGDKERGVTINSGNFSGLSYQDAVAAVAHAL
AEKGLGELKTTWRLRDWGISRQRYWGTPIPIIHCESCGAVPVPEKDLPVVLPQDLVPDGS
GNPLAKCEAFLKVDCPCCGKPARRETDTMDTFVDSSWYFMRYCDPKNRDAMVAGGTDYWM
RDQKAATGGSGMDQYIGGIEHAILHLLYARFWTKVMRDLGLVKVDEPFTKLLTQGMVLNH
IYSRRTAKGAKDYFWPHDVEHVYDEAGKIVGAKLKNPAESGDGLLPVGTPIDYEGVGTMS

KSKNNGVDPQQLIEKYGADTARLYTMFTAPPELTLEWNDAAVEGSYRFLRRVWNFGVKLS
AIDKDAALASVAGAASLKDVQFGKEAKALRLEIHTVLKQVDYDYQRMQYNTVVSGAMKMI
NALEDFKATDSAGAQVALIEGFGILLRVLYPATPHIAHVLWDELGYAGTLGDLLDAAWPQ
VAPDALVQDELELMLQVNGKLRGAIRVAASADKAAIEQAALASEDFHKFAEGKAPKKVII
VPGRLVNVVV
>P24518
MSSFDYLKTAIKQQGCTLQQVADASGMTKGYLSQLLNAKIKSPSAQKLEALHRFLGLEFP
RRQKNIGVVFGKFYPLHTGHIYLIQRACSQVDELHIIMGYDDTRDRGLFEDSAMSQQPTV
SDRLRWLLQTFKYQKNIRIHAFNEEGMEPYPHGWDVWSNGIKAFMAEKGIQPSWIYTSEE
ADAPQYLEHLGIETVLVDPERTFMNISGAQIRENPFRYWEYIPTEVKPFFVRTVAILGGE
SSGKSTLVNKLANIFNTTSAWEYGRDYVFSHLGGDEMALQYSDYDKIALGHAQYIDFAVK
YANKVAFIDTDFVTTQAFCKKYEGREHPFVQALIDEYRFDLVILLENNTPWVADGLRSLG
SSVDRKAFQNLLVEMLKENNIEFVHVKEADYDGRFLRCVELVKEMMGEQG
>P0A6T1
MKNINPTQTAAWQALQKHFDEMKDVTIADLFAKDGDRFSKFSATFDDQMLVDYSKNRITE
ETLAKLQDLAKECDLAGAIKSMFSGEKINRTENRAVLHVALRNRSNTPILVDGKDVMPEV
NAVLEKMKTFSEAIISGEWKGYTGKAITDVVNIGIGGSDLGPYMVTEALRPYKNHLNMHF
VSNVDGTHIAEVLKKVNPETTLFLVASKTFTTQETMTNAHSARDWFLKAAGDEKHVAKHF
AALSTNAKAVGEFGIDTANMFEFWDWVGGRYSLWSAIGLSIVLSIGFDNFVELLSGAHAM
DKHFSTTPAEKNLPVLLALIGIWYNNFFGAETEAILPYDQYMHRFAAYFQQGNMESNGKY
VDRNGNVVDYQTGPIIWGEPGTNGQHAFYQLIHQGTKMVPCDFIAPAITHNPLSDHHQKL
LSNFFAQTEALAFGKSREVVEQEYRDQGKDPATLDYVVPFKVFEGNRPTNSILLREITPF
SLGALIALYEHKIFTQGVILNIFTFDQWGVELGKQLANRILPELKDDKEISSHDSSTNGL
INRYKAWRG
>P69428
MGGISIWQLLIIAVIVVLLFGTKKLGSIGSDLGASIKGFKKAMSDDEPKQDKTSQDADFT
AKTIADKQADTNQEQAKTEDAKRHDKEQV
>P60293
MSEFSQTVPELVAWARKNDFSISLPVDRLSFLLAVATLNGERLDGEMSEGELVDAFRHVS
DAFEQTSETIGVRANNAINDMVRQRLLNRFTSEQAEGNAIYRLTPLGIGITDYYIRQREF
STLRLSMQLSIVAGELKRAADAAEEGGDEFHWHRNVYAPLKYSVAEIFDSIDLTQRLMDE
QQQQVKDDIAQLLNKDWRAAISSCELLLSETSGTLRELQDTLEAAGDKLQANLLRIQDAT
MTHDDLHFVDRLVFDLQSKLDRIISWGQQSIDLWIGYDRHVHKFIRTAIDMDKNRVFAQR
LRQSVQTYFDEPWALTYANADRLLDMRDEEMALRDEEVTGELPEDLEYEEFNEIREQLAA
IIEEQLAVYKTRQVPLDLGLVVREYLSQYPRARHFDVARIVIDQAVRLGVAQADFTGLPA
KWQPINDYGAKVQAHVIDKY
>P04825
MTQQPQAKYRHDYRAPDYQITDIDLTFDLDAQKTVVTAVSQAVRHGASDAPLRLNGEDLK
LVSVHINDEPWTAWKEEEGALVISNLPERFTLKIINEISPAANTALEGLYQSGDALCTQC
EAEGFRHITYYLDRPDVLARFTTKIIADKIKYPFLLSNGNRVAQGELENGRHWVQWQDPF
PKPCYLFALVAGDFDVLRDTFTTRSGREVALELYVDRGNLDRAPWAMTSLKNSMKWDEER
FGLEYDLDIYMIVAVDFFNMGAMENKGLNIFNSKYVLARTDTATDKDYLDIERVIGHEYF
HNWTGNRVTCRDWFQLSLKEGLTVFRDQEFSSDLGSRAVNRINNVRTMRGLQFAEDASPM
AHPIRPDMVIEMNNFYTLTVYEKGAEVIRMIHTLLGEENFQKGMQLYFERHDGSAATCDD

FVQAMEDASNVDLSHFRRWYSQSGTPIVTVKDDYNPETEQYTLTISQRTPATPDQAEKQP
LHIPFAIELYDNEGKVIPLQKGGHPVNSVLNVTQAEQTFVFDNVYFQPVPALLCEFSAPV
KLEYKWSDQQLTFLMRHARNDFSRWDAAQSLLATYIKLNVARHQQGQPLSLPVHVADAFR
AVLLDEKIDPALAAEILTLPSVNEMAELFDIIDPIAIAEVREALTRTLATELADELLAIY
NANYQSEYRVEHEDIAKRTLRNACLRFLAFGETHLADVLVSKQFHEANNMTDALAALSAA
VAAQLPCRDALMQEYDDKWHQNGLVMDKWFILQATSPAANVLETVRGLLQHRSFTMSNPN
RIRSLIGAFAGSNPAAFHAEDGSGYLFLVEMLTDLNSRNPQVASRLIEPLIRLKRYDAKR
QEKMRAALEQLKGLENLSGDLYEKITKALA
>P69811
MFQLSVQDIHPGEKAGDKEEAIRQVAAALVQAGNVAEGYVNGMLAREQQTSTFLGNGIAI
PHGTTDTRDQVLKTGVQVFQFPEGVTWGDGQVAYVAIGIAASSDEHLGLLRQLTHVLSDD
SVAEQLKSATTAEELRALLMGEKQSEQLKLDNEMLTLDIVASDLLTLQALNAARLKEAGA
VDATFVTKAINEQPLNLGQGIWLSDSAEGNLRSAIAVSRAANAFDVDGETAAMLVSVAMN
DDQPIAVLKRLADLLLDNKADRLLKADAATLLALLTSDDAPTDDVLSAEFVVRNEHGLHA
RPGTMLVNTIKQFNSDITVTNLDGTGKPANGRSLMKVVALGVKKGHRLRFTAQGADAEQA
LKAIGDAIAAGLGEGA
>Q05526
MSIFTDLNTSRKWQIDQWLSAVNSHIEKIQQYGHSVVNPTPLLADGFEIKTQSPVVWQFP
DGHDAPISNFASQQNWLRLLISMSVITETEKYRHLAFCQSEYFLNRFVDENSGLFYWGGH
RFINLDTLASEGPESKSMVHELKHHLPYYEFLHQVNPEKTRHFIQGFWNAHVEDWSCLDL
GRHGDYARQRDPDVFLHSRHDVVTPANWPELPLTKGLTFVNAGTDLIYAAFVYARHTGDA
HAAAWGKHLYRQYVLARNPETGMPVYQFSSPLQRQPVPADDNQTQSWFGDRAQRQFGPEF
GAIAREANVLFRDMRPLLIDNPLAMLDILRHQPDAEILTWVIAGLKNYYQYAYDVNSNSL
RPMWNNGQDMTDYCFKRDGYYGKAGTVLKPFPLEGDYLLPLVRAWLLSDDDDLHTLIVTM
LSRLEKQGIHQSASPFLLLAITELAHAKQSAQWAEYAWQMAEILFKRYFHHGLFVRSEHH
RYVRLDDPFPAILLTLIAACRNKWSEVPAVLTQGGYIHGDYRINGESRVIYDTGIYLPRK
INPLILFLQIHHY
>P60595
MNVVILDTGCANLNSVKSAIARHGYEPKVSRDPDVVLLADKLFLPGVGTAQAAMDQVRER
ELFDLIKACTQPVLGICLGMQLLGRRSEESNGVDLLGIIDEDVPKMTDFGLPLPHMGWNR
VYPQAGNRLFQGIEDGAYFYFVHSYAMPVNPWTIAQCNYGEPFTAAVQKDNFYGVQFHPE
RSGAAGAKLLKNFLEM
>P0A1K1
MAQHGALETLKDLAEKEVDDAARLLGEMRRGCQQAEEQLKMLIDYQNEYRSNLNTDMGNG
IASNRWINYQQFIQTLEKAIEQHRLQLTQWTQKVDLALKSWREKKQRLQAWQTLQDRQTA
AALLAENRMDQKKMDEFAQRAAMRKPE
>P0A715
MKQKVVSIGDINVANDLPFVLFGGMNVLESRDLAMRICEHYVTVTQKLGIPYVFKASFDK
ANRSSIHSYRGPGLEEGMKIFQELKQTFGVKIITDVHEPSQAQPVADVVDVIQLPAFLAR
QTDLVEAMAKTGAVINVKKPQFVSPGQMGNIVDKFKEGGNEKVILCDRGANFGYDNLVVD
MLGFSIMKKVSGNSPVIFDVTHALQCRDPFGAASGGRRAQVAELARAGMAVGLAGLFIEA
HPDPEHAKCDGPSALPLAKLEPFLKQMKAIDDLVKGFEELDTSK
>P0A9B6
MTVRVAINGFGRIGRNVVRALYESGRRAEITVVAINELADAAGMAHLLKYDTSHGRFAWE

VRQERDQLFVGDDAIRVLHERSLQSLPWRELGVDVVLDCTGVYGSREHGEAHIAAGAKKV
LFSHPGSNDLDATVVYGVNQDQLRAEHRIVSNASCTTNCIIPVIKLLDDAYGIESGTVTT
IHSAMHDQQVIDAYHPDLRRTRAASQSIIPVDTKLAAGITRFFPQFNDRFEAIAVRVPTI
NVTAIDLSVTVKKPVKANEVNLLLQKAAQGAFHGIVDYTELPLVSVDFNHDPHSAIVDGT
QTRVSGAHLIKTLVWCDNEWGFANRMLDTTLAMATVAFR
>P22320
MSRKLVIDPVTRIEGHGKVVVHLDDDNKVVDAKLHVVEFRGFEKFVQGHPFWEAPMFLQR
ICGICFVSHHLCGAKALDDMVGVGLKSGIHVTPTAEKMRRLGHYAQMLQSHTTAYFYLIV
PEMLFGMDAPPAQRNVLGLIEANPDLVKRVVMLRKWGQEVIKAVFGKKMHGINSVPGGVN
NNLSIAERDRFLNGEEGLLSVDQVIDYAQDGLRLFYDFHQKHRAQVDSFADVPALSMCLV
GDDDNVDYYHGRLRIIDDDKHIVREFDYHDYLDHFSEAVEEWSYMKFPYLKELGREQGSV
RVGPLGRMNVTKSLPTPLAQEALERFHAYTKGRTNNMTLHTNWARAIEILHAAEVVKELL
HDPDLQKDQLVLTPPPNAWTGEGVGVVEAPRGTLLHHYRADERGNITFANLVVATTQNNQ
VMNRTVRSVAEDYLGGHGEITEGMMNAIEVGIRAYDPCLSCATHALGQMPLVVSVFDAAG
RLIDERAR
>P55913
MKFIIKLFPEITIKSQSVRLRFIKILTGNIRNVLKHYDETLAVVRHWDNIEVRAKDENQR
LVIRDALTRIPGIHHILEVEDVPFTDMHDIFEKALAQYREQLEGKTFCVRVKRRGKHEFS
SIEVERYVGGGLNQHIESARVKLTNPDVTVHLEVEDDRLLLIKGRYEGIGGFPIGTQEDV
LSLISGGFDSGVSSYMLMRRGCRVHYCFFNLGGAAHEIGVRQVAHYLWNRFGSSHRVRFV
AINFEPVVGEILEKVDDGQMGVVLKRMMVRAASKVAERYGVQALVTGEALGQVSSQTLTN
LRLIDNVSDTLILRPLISYDKEHIINLARQIGTEDFARTMPEYCGVISKSPTVKAIKAKI
EAEEENFDFSILDKVVEEANNVDIREIAQQTQQEVVEVETVSGFGPNDVILDIRSVDEQD
DKPLKVEGVDVVSLPFYKLSTKFGDLDQSKTWLLWCERGVMSRLQALYLREQGFANVKVY
RP
>Q8GHL1
MTALYWQTIGEGERDLVLLHGWGLNAEVWSCIQALTPHFRLHLVDLPGYGRSQGFGALSL
AQMTEIVLAAAPPQAWWLGWSLGGLVASQAALMQPQRVSGLITVASSPCFAARDEWPGIR
PDVLSGFQHQLSLDFQRTVERFLALQTLGTESARQDARQLKAVVLNQPTPSVEVLNGGLE
ILRTADLRAPLAELNLPLLRIYGYLDGLVPRKVAELLDAAWPNSTSQIVAKAAHAPFISH
PDEFVTMIEAFIAAH
>P0A6F5
MAAKDVKFGNDARVKMLRGVNVLADAVKVTLGPKGRNVVLDKSFGAPTITKDGVSVAREI
ELEDKFENMGAQMVKEVASKANDAAGDGTTTATVLAQAIITEGLKAVAAGMNPMDLKRGI
DKAVTAAVEELKALSVPCSDSKAIAQVGTISANSDETVGKLIAEAMDKVGKEGVITVEDG
TGLQDELDVVEGMQFDRGYLSPYFINKPETGAVELESPFILLADKKISNIREMLPVLEAV
AKAGKPLLIIAEDVEGEALATLVVNTMRGIVKVAAVKAPGFGDRRKAMLQDIATLTGGTV
ISEEIGMELEKATLEDLGQAKRVVINKDTTTIIDGVGEEAAIQGRVAQIRQQIEEATSDY
DREKLQERVAKLAGGVAVIKVGAATEVEMKEKKARVEDALHATRAAVEEGVVAGGGVALI
RVASKLADLRGQNEDQNVGIKVALRAMEAPLRQIVLNCGEEPSVVANTVKGGDGNYGYNA
ATEEYGNMIDMGILDPTKVTRSALQYAASVAGLMITTECMVTDLPKNDAADLGAAGGMGG
MGGMGGMM
>P0A7G6
MAIDENKQKALAAALGQIEKQFGKGSIMRLGEDRSMDVETISTGSLSLDIALGAGGLPMG

RIVEIYGPESSGKTTLTLQVIAAAQREGKTCAFIDAEHALDPIYARKLGVDIDNLLCSQP
DTGEQALEICDALARSGAVDVIVVDSVAALTPKAEIEGEIGDSHMGLAARMMSQAMRKLA
GNLKQSNTLLIFINQIRMKIGVMFGNPETTTGGNALKFYASVRLDIRRIGAVKEGENVVG
SETRVKVVKNKIAAPFKQAEFQILYGEGINFYGELVDLGVKEKLIEKAGAWYSYKGEKIG
QGKANATAWLKDNPETAKEIEKKVRELLLSNPNSTPDFSVDDSEGVAETNEDF
>P0A9L5
MAKTAAALHILVKEEKLALDLLEQIKNGADFGKLAKKHSICPSGKRGGDLGEFRQGQMVP
AFDKVVFSCPVLEPTGPLHTQFGYHIIKVLYRN
>Q8Y211
MLTFQQLILKLQSYWDAQGCALLQPIDLEVGAGTSHVHTFLRAIGPEPWRAAYVQPSRRP
KDGRYGENPNRLQHYYQYQVVLKPAPENILDLYLGSLQALGLDLKQNDVRFVEDDWENPT
LGAWGLGWEVWLNGMEVTQFTYFQQVGGLDCKPITGEITYGIERLAMYLQQVENVYDLVW
TEWEEPGPNGPVKRRLTYGDVYHQNEVEQSTYNFEHADTAVLFRRFAEHEAEAKRLMGVR
EEGAADDGAAVPQLALPAYEQVLKAGHTFNLLDARGAISVTERAAYIGRIRNLSRLVAQA
YYDSRERLGFPMCGTAAAPAATEAA
>P0A9J6
MQNAGSLVVLGSINADHILNLQSFPTPGETVTGNHYQVAFGGKGANQAVAAGRSGANIAF
IACTGDDSIGESVRQQLATDNIDITPVSVIKGESTGVALIFVNGEGENVIGIHAGANAAL
SPALVEAQRERIANASALLMQLESPLESVMAAAKIAHQNKTIVALNPAPARELPDELLAL
VDIITPNETEAEKLTGIRVENDEDAAKAAQVLHEKGIRTVLITLGSRGVWASVNGEGQRV
PGFRVQAVDTIAAGDTFNGALITALLEEKPLPEAIRFAHAAAAIAVTRKGAQPSVPWREE
IDAFLDRQR
>Q59685
MYLYIETLKQRLDAINQLRLERAFASMSDVFKQVYGLIPVLLHYHHPQLPGYIQGNVPHG
TCFFEPDDVQRQWVNKLTNASCDEPMNGYTSGELPITGIYSMGSTSSIGQSHCSDIDIWV
CHQSWLDQDERARLQRKCLLIEQWAGELGIDVTFFLIDENRFRHHASGSLGGEDCGSTQH
ILLLDEFYRTAVRLAGKRLLWTMVPVEEEYHYDEYVNSLYAQGVLTPNEWLDLGGLGELS
AEEYFGASLWQLYKSVDSPYKAVLKSILLEAYSADYPNGKLLALEMKQHLHRGEIVNYGL
DAYCMMLERVTRYLVSINDLTRLDLIRRCFYLKVCEKLSNEKNENEPAGWRRQVLSQLVT
QWQWDHERLAILDNRDSWKIERVRNAHNELLDTMMQSYRNLIRFARRNNLSVSASPQDIG
VLTRKLYAAFEALPGKVTLVNPQISPDLSEPHLTFIYVPPGRANRSGWYLYNRAPDFAHI
VGHQPLEYNRYLNKLVAWSYFNGLLTKDSQVYIHQGDSSCDEIKLHELVRDISSHFPIRL
PAPTPKALYSPCEIRHLAIIVNLEVDPTERFSDQVVHFDFRKLDVFSFGEEEQCLIGSID
LLYRNSWNEVRTLHFNGTQSMLESLKTILGKMHQDAAPPASVEVFCYSQHLRGLIRTRVQ
QLVSECIELRLSTNRLEPGRFKALRIAGQTWGLFFERLNVSVQKLENAIEFYGAISYNKL
HGLPVKLGKDARYLPAVIDGFACEGIIQFFFETTEDNNVFNIYILDEANRVEIYSHCEGS
KEELVKDVSRFYSSSHDRFTYGSSFINFNLPQFYQIVKVDGATQVLPFAGGSFGKLSDLG
KTAPKEEMSTKPIQGFNDYQAVHHH
>P78055
MELYLDTSDVVAVKALSRIFPLAGVTTNPSIIAAGKKPLDVVLPQLHEAMGGQGRLFAQV
MATTAEGMVNDALKLRSIIADIVVKVPVTAEGLAAIKMLKAEGIPTLGTAVYGAAQGLLS
ALAGAEYVAPYVNRIDAQGGSGIQTVTDLHQLLKMHAPQAKVLAASFKTPRQALDCLLAG
CESITLPLDVAQQMISYPAVDAAVAKFEQDWQGAFGRTSI
>P0AED0

MAYKHILIAVDLSPESKVLVEKAVSMARPYNAKVSLIHVDVNYSDLYTGLIDVNLGDMQK
RISEETHHALTELSTNAGYPITETLSGSGDLGQVLVDAIKKYDMDLVVCGHHQDFWSKLM
SSARQLINTVHVDMLIVPLRDEEE
>Q1DES4
MIREQRSSRGGSRDQRTNRRIRAREVRVVGSDGSQLGVMPLEAALDRARTEGLDLVEISP
MASPPVCKIMDYGKFKYEEKKKASEAKRAQVTVLLKEVKLRPKTEEHDYEFKVRNTRRFI
EDGNKAKVVIQFRGREITHREQGTAILDDVAKDLKDVAVVEQMPRMEGRLMFMILAPTPK
VAQKARELVRQAATAAKRPPPPGAPGAGKSAAGASSGAEEKAEETAEEKKEAQAAPAAAE
AQSPTAS
>P22523
MIERGKFRSLTLINWNGFFARTFDLDELVTTLSGGNGAGKSTTMAAFVTALIPDLTLLHF
RNTTEAGATSGSRDKGLHGKLKAGVCYSMLDTINSRHQRVVVGVRLQQVAGRDRKVDIKP
FAIQGLPMSVQPTQLVTETLNERQARVLPLNELKDKLEAMEGVQFKQFNSITDYHSLMFD
LGIIARRLRSASDRSKFYRLIEASLYGGISSAITRSLRDYLLPENSGVRKAFQDMEAALR
ENRMTLEAIRVTQSDRDLFKHLISEATNYVAADYMRHANERRVHLDKALEFRRELHTSRQ
QLAAEQYKHVDMARELAEHNGAEGDLEADYQAASDHLNLVQTALRQQEKIERYEADLDEL
QIRLEEQNEVVAEAIERQQENEARAEAAELEVDELKSQLADYQQALDVQQTRAIQYNQAI
AALNRAKELCHLPDLTADCAAEWLETFQAKELEATEKMLSLEQKMSMAQTAHSQFEQAYQ
LVVAINGPLARNEAWDVARELLREGVDQRHLAEQVQPLRMRLSELEQRLREQQEAERLLA
DFCKRQGKNFDIDELEALHQELEARIASLSDSVSNAREERMALRQEQEQLQSRIQSLMQR
APVWLAAQNSLNQLSEQCGEEFTSSQDVTEYLQQLLEREREAIVERDEVGARKNAVDEEI
ERLSQPGGSEDQRLNALAERFGGVLLSEIYDDVSLEDAPYFSALYGPSRHAIVVPDLSQV
TEHLEGLTDCPEDLYLIEGDPQSFDDSVFSVDELEKAVVVKIADRQWRYSRFPEVPLFGR
AARESRIESLHAEREVLSERFATLSFDVQKTQRLHQAFSRFIGSHLAVAFESDPEAEIRQ
LNSRRVELERALSNHENDNQQQRIQFEQAKEGVTALNRILPRLNLLADDSLADRVDEIRE
RLDEAQEAARFVQQFGNQLAKLEPIVSVLQSDPEQFEQLKEDYAYSQQMQRDARQQAFAL
TEVVQRRAHFSYSDSAEMLSGNSDLNEKLRERLEQAEAERTRAREALRGHAAQLSQYNQV
LASLKSSYDTKKELLNDLQRELQDIGVRADSGAEERARIRRDELHAQLSNNRSRRNQLEK
ALTFCEAEMDNLTRKLRKLERDYFEMREQVVTAKAGWCAVMRMVKDNGVERRLHRRELAY
LSADDLRSMSDKALGALRLAVADNEHLRDVLRMSEDPKRPERKIQFFVAVYQHLRERIRQ
DIIRTDDPVEAIEQMEIELSRLTEELTSREQKLAISSRSVANIIRKTIQREQNRIRMLNQ
GLQNVSFGQVNSVRLNVNVRETHAMLLDVLSEQHEQHQDLFNSNRLTFSEALAKLYQRLN
PQIDMGQRTPQTIGEELLDYRNYLEMEVEVNRGSDGWLRAESGALSTGEAIGTGMSILVM
VVQSWEDESRRLRGKDISPCRLLFLDEAARLDARSIATLFELCERLQMQLIIAAPENISP
EKGTTYKLVRKVFQNTEHVHVVGLRGFAPQLPETLPGTDEAPSQAS
>Q09064
MIIERLVGNLRDLNPLDFSVDHVDLEWFETRKKIARFKTRQGKDIAIRLKDAPKLGLSQG
DILFKEEKEIIAVNILDSEVIHIQAKSVAEVAKICYEIGNRHAALYYGESQFEFKTPFEK
PTLALLEKLGVQNRVLSSKLDSKERLTVSMPHSEPNFKVSLASDFKVVVK
>Q9ABY6
MFEGLSPLARDARSWPFEQARATIARVLRVRLPDRADQDAAKALIDAGKTDEAVKAYPAL
AKAVIFETGYGPSGLPHLGTFGEVARTTMVRQAFRALTDEAIPTRLIAFSDDMDGLRKVP
DNIENKQPLIEDLGKPLTVVRDPFGTHDSFGAHNNARLRAFLDGFGFEYEFVSSTDCYKG
GLFDATLLTALERFDAIQKVMLPTLGEERRATYSPFLPISPSTGKVLQVPTLERNVEKGT

IVFEDEDGSKVEVPVTGGHVKMQWKPDWAMRWTALGVDYEMSGKDLIDSVKASGAICKAL
GGVPPEGFNYELFLDENNQKISKSKGNGLSMEDWLRYGAPESLSYYMFQSPKSAKKLYFD
VIPKASDEYLQQLDGFGRQEPAKQLDNPVWHIHGGKPPQQGSPVSFSLMLNLVSAADAST
KEILWGFLSRYIPGASPETQPLLDRLAGYAINYYEDFVKPSKVFRAPSDQERAAMLDLLA
KLKAMPAGTQDAELIQNEVFEVGKTHGFDPLRAWFQALYEVLLGQSQGPRFGSFAAIFGI
DRTVALIEEKLG
>P11071
MPRGLELLIAQTILQGFDAQYGRFLEVTSGAQQRFEQADWHAVQQAMKNRIHLYDHHVGL
VVEQLRCITNGQSTDAAFLLRVKEHYTRLLPDYPRFEIAESFFNSVYCRLFDHRSLTPER
LFIFSSQPERRFRTIPRPLAKDFHPDHGWESLLMRVISDLPLRLRWQNKSRDIHYIIRHL
TETLGTDNLAESHLQVANELFYRNKAAWLVGKLITPSGTLPFLLPIHQTDDGELFIDTCL
TTTAEASIVFGFARSYFMVYAPLPAALVEWLREILPGKTTAELYMAIGCQKHAKTESYRE
YLVYLQGCNEQFIEAPGIRGMVMLVFTLPGFDRVFKVIKDRFAPQKEMSAAHVRACYQLV
KEHDRVGRMADTQEFENFVLEKRHISPALMELLLQEAAEKITDLGEQIVIRHLYIERRMV
PLNIWLEQVEGQQLRDAIEEYGNAIRQLAAANIFPGDMLFKNFGVTRHGRVVFYDYDEIC
YMTEVNFRDIPPPRYPEDELASEPWYSVSPGDVFPEEFRHWLCADPRIGPLFEEMHADLF
RADYWRALQNRIREGHVEDVYAYRRRQRFSVRYGEMLF
>P33393
MPEGKFCNRKPVNTEEDLKALLGDKGGAQYYKEMEELEVDQEALWANIEKTCQSRTKTWL
EICAHCGMCADSCFLYRVNDRDPKQVPAYKIQSTLGEIIRRKGKVDTQFMLHAMEVAWSQ
CTCCNRCGQYCPHGIDMGVMFSYLRGLLYSQGFVPWELKIGSGMHRVYGAQMDVTTEDWV
ETCEWMAEEQQEEWPGLEIPVDVENADIMYVLNAREPKHYPEDVAEAAILFHIAGENWTV
PSEGWEQTSLAMFAGDWAACKMQVERVYAAIERLKPKCVVGTECGHAHRASAIEGPYWAG
YEDGKTPAPWLHYVEWVAMALRTGKIKIDPEKRIKEPVTLQDSCNYIRNHGLAKCTREIM
SYIADDFREMTPNREHNYCCGGGGGFNGIGKFRKQRNKALQTKRDQILATGAKLVVAPCH
NCWDAIRDLEEEYRIGIRWSFLKPLIIKMAIIPEHLRPEEE
>P0AC69
MSTTIEKIQRQIAENPILLYMKGSPKLPSCGFSAQAVQALAACGERFAYVDILQNPDIRA
ELPKYANWPTFPQLWVDGELVGGCDIVIEMYQRGELQQLIKETAAKYKSEEPDAE
>Q9EXP1
MAQLPDPQKLLRNFSRCSNWEEKYLYIIELGAGLAPLSDAQRQDGNRVSGCQSQVWIDLA
SNEQGNVVLHGDSDAAIVKGLIAIVFSLYQGLSVREIVELDVRPFFASLALTQHLTPSRS
QGLEAMLRAVRARASALI
>P50176
MTAEKAEGATGFAGFDPKSVEPYIVKDPESLAINMARAAEQLGKAASAWLAPREAGEKTD
SFAEPVSDMVKTLSKVSEYWLSDPRRTLEAQTHLLGSFFDMWSRTLQRMAGDAVEDPANL
QRNDKRFADEDWVKNPFFDFIRQAYFVTSDWAERMVRDAEGLDDHTRHKAAFYVRQIASA
LSPTNFITTNPQLYRETVASSGANLVKGMQMLAEDIAAGRGELRLRQTDTSKFAIGENIA
ITPGKVIAQNDVCQVLQYEASTETVLKRPLLICPPWINKFYVLDLNPEKSFIKWAVDQGQ
TVFVISWVNPDERHASKDWEAYAREGIGFALDIIEQATGEREVNSIGYCVGGTLLAATLA
LHAAEGDERIRSATLFTTQVDFTHAGDLKVFVDDDQIRHLEANMSATGYLEGSKMASAFN
MLRASELIWPYFVNNYLKGQDPLPFDLLYWNSDSTRMPAANHSFYLRNCYLENRLSKGEM
VLAGRRVSLGDVKIPIYNLATKEDHIAPAKSVFLGSSSFGGKVTFVLSGSGHIAGVVNPP
ARSKYQYWTGGAPKGDIETWMGKAKETAGSWWPHWQGWVERLDKRRVPARKAGGPLNSIE

EAPGSYVRVRA
>Q46822
MQTEHVILLNAQGVPTGTLEKYAAHTADTRLHLAFSSWLFNAKGQLLVTRRALSKKAWPG
VWTNSVCGHPQLGESNEDAVIRRCRYELGVEITPPESIYPDFRYRATDPSGIVENEVCPV
FAARTTSALQINDDEVMDYQWCDLADVLHGIDATPWAFSPWMVMQATNREARKRLSAFTQ
LK
>P68739
MDLASLRAQQIELASSVIREDRLDKDPPDLIAGADVGFEQGGEVTRAAMVLLKYPSLELV
EYKVARIATTMPYIPGFLSFREYPALLAAWEMLSQKPDLVFVDGHGISHPRRLGVASHFG
LLVDVPTIGVAKKRLCGKFEPLSSEPGALAPLMDKGEQLAWVWRSKARCNPLFIATGHRV
SVDSALAWVQRCMKGYRLPEPTRWADAVASERPAFVRYTANQP
>Q9ZKG9
MQKSLITTPIYYVNDIPHIGHAYTTLIADTLKKYYTLQGEEVFFLTGTDEHGQKIEQSAR
LRNQSPKAYADSISAIFKNQWDFFNLDYDGFIRTTDSEHQKCVQNAFEIMFEKGDIYKGT
YSGYYCVSCESYCAVSKVDNTDSKVLCPDCLRETTLLEEESYFFKLSAYEKPLLEFYAKN
PEAILPIYRKNEVTSFIEQGLLDLSITRTSFEWGIPLPKKMNDPKHVVYVWLDALLNYAS
ALGYLNGLDNKMAHFERARHIVGKDILRFHAIYWPAFLMSLNLPLFKQLCVHGWWTIEGV
KMSKSLGNVLDAQKLAMEYGIEELRYFLLREVPFGQDGDFSKKALVERINANLNNDLGNL
LNRLLGMAKKYFNYSLKSTKITAYYPKELEKAHQILDNANSFVPKMQLHKALEELFNIYD
FLNKLIAKEEPWVLHKNNESEKLEALLSLIANTLLQSSFLLYAFMPKSAMKLASAFRVEI
TPNNYERFFKAKKLQDMVLQDTEPLFSKIEKIEKIEKIEKIEKIEKIEKGEEALAEKAEKKEK
EKAPPTQENYISIEDFKKVEIKVGLIKEAQRIEKSNKLLRLKVDLGENRLRQIISGIALD
YEPESLVGQMVCVVANLKPAKLMGEMSEGMILAVRDNDNLALISPTREKIAGSLIS
>P69853
MTHFSQQDNFSVAARVLGALFYYAPESAEAAPLVAVLTSDGWETQWPLPEASLAPLVTAF
QTQCEETHAQAWQRLFVGPWALPSPPWGSVWLDRESVLFGDSTLALRQWMREKGIQFEMK
QNEPEDHFGSLLLMAAWLAENGRQTECEELLAWHLFPWSTRFLDVFIEKAEHPFYRALGE
LARLTLAQWQSQLLIPVAVKPLFR
>P21338
MKAFWRNAALLAVSLLPFSSANALALQAKQYGDFDRYVLALSWQTGFCQSQHDRNRNERD
ECRLQTETTNKADFLTVHGLWPGLPKSVAARGVDERRWMRFGCATRPIPNLPEARASRMC
SSPETGLSLETAAKLSEVMPGAGGRSCLERYEYAKHGACFGFDPDAYFGTMVRLNQEIKE
SEAGKFLADNYGKTVSRRDFDAAFAKSWGKENVKAVKLTCQGNPAYLTEIQISIKADAIN
APLSANSFLPQPHPGNCGKTFVIDKAGY
>P33920
MSLDINQIALHQLIKRDEQNLELVLRDSLLEPTETVVEMVAELHRVYSAKNKAYGLFSEE
SELAQTLRLQRQGEEDFLAFSRAATGRLRDELAKYPFADGGFVLFCHYRYLAVEYLLVAV
LSNLSSMRVNENLDINPTHYLDINHADIVARIDLTEWETNPESTRYLTFLKGRVGRKVAD
FFMDFLGASEGLNAKAQNRGLLQAVDDFTAEAQLDKAERQNVRQQVYSYCNEQLQAGEEI
ELKSLSKELAGVSEVSFTEFAAEKGYELEESFPADRSTLRQLTKFAGSGGGLTINFDAML
LGERIFWDPATDTLTIKGTPPNLRDQLQRRTSGGN
>P0A962
MQKKSIYVAYTGGTIGMQRSEQGYIPVSGHLQRQLALMPEFHRPEMPDFTIHEYTPLMDS
SDMTPEDWQHIAEDIKAHYDDYDGFVILHGTDTMAYTASALSFMLENLGKPVIVTGSQIP

LAELRSDGQINLLNALYVAANYPINEVTLFFNNRLYRGNRTTKAHADGFDAFASPNLPPL
LEAGIHIRRLNTPPAPHGEGELIVHPITPQPIGVVTIYPGISADVVRNFLRQPVKALILR
SYGVGNAPQNKAFLQELQEASDRGIVVVNLTQCMSGKVNMGGYATGNALAHAGVIGGADM
TVEATLTKLHYLLSQELDTETIRKAMSQNLRGELTPDD
>P17052
MAIVVVGAGTAGVNAAFWLRQYGYKGEIRIFSRESVAPYQRPPLSKAFLTSEIAESAVPL
KPEGFYTNNNITISLNTPIVSIDVGRKIVSSKDGKEYAYEKLILATPASARRLTCEGSEL
SGVCYLRSMEDAKNLRRKLVESASVVVLGGGVIGLEVASAAVGLGKRVTVIEATPRVMAR
VVTPAAANLVRARLEAEGIEFKLNAKLTSIKGRNGHVEQCVLESGEEIQADLIVVGIGAI
PELELATEAALEVSNGVVVDDQMCTSDTSIYAIGDCAMARNPFWGTMVRLETIHNAVTHA
QIVASSICGTSTPAPTPPRFWSDLKGMALQGLGALKDYDKLVVAINNETLELEVLAYKQE
RLIATETINLPKRQGALAGSIKLPD
>P75726
MTQKIEQSQRQERVAAWNRRAECDLAAFQNSPKQTYQAEKARDRKLCANLEEAIRRSGLQ
DGMTVSFHHAFRGGDLTVNMVMDVIAKMGFKNLTLASSSLSDCHAPLVEHIRQGVVTRIY
TSGLRGPLAEEISRGLLAEPVQIHSHGGRVHLVQSGELNIDVAFLGVPSCDEFGNANGYT
GKACCGSLGYAIVDADNAKQVVMLTEELLPYPHNPASIEQDQVDLIVKVDRVGDAAKIGA
GATRMTTNPRELLIARSAADVIVNSGYFKEGFSMQTGTGGASLAVTRFLEDKMRSRDIRA
DFALGGITATMVDLHEKGLIRKLLDVQSFDSHAAQSLARNPNHIEISANQYANWGSKGAS
VDRLDVVVLSALEIDTQFNVNVLTGSDGVLRGASGGHCDTAIASALSIIVAPLVRGRIPT
LVDNVLTCITPGSSVDILVTDHGIAVNPARPELAERLQEAGIKVVSIEWLRERARLLTGE
PQPIEFTDRVVAVVRYRDGSVIDVVHQVKE
>Q9I742
MSEISRVALFGKLNSLAYKAIEAATVFCKLRGNPYVELVHWFHQILQLPDSDLHQIVRQS
GIDPARLAKDLTEALDRLPRGSTSITDLSSHVEEAVERGWVYGSLMFGESQVRTGYLVIG
ILKTPSLRHALTGLSAEFAKLKVEALTERFDEYVGASPENGLSASDGFNAGAAPGEASGA
LAPSAMGKQEALKRFTVDLTEQARSGKLDPIVGRDEEIRQLVDILMRRRQNNPILTGEAG
VGKTAVVEGFALRIVAGDVPPALKDVELRALDVGLLQAGASMKGEFEQRLRQVIEDVQSS
EKPIILFIDEAHTLVGAGGAAGTGDAANLLKPALARGTLRTVAATTWAEYKKHIEKDPAL
TRRFQVVQVDEPSEHKAILMMRGVASTMEKHHQVQILDEALEAAVRLSHRYIPARQLPDK
SVSLLDTACARTAISLHAVPAEVDDSRRRIEALETELAIIRRESAIGVATAERQRNAETL
LAEERERLAALEQRWAEEKRLVDELLETRARLRAAAEAVDAGGVPLGEGEVRLDEEQRQA
LHARLAELQAQLSALQGEEPLILPTVDYQAVASVVADWTGIPVGRMARNEIETVLNLDRH
LKKRIIGQDHALEMIAKRIQTSRAGLDNPSKPIGVFMLAGTSGVGKTETALALAEAMYGG
EQNVITINMSEFQEAHTVSTLKGAPPGYIGYGEGGVLTEAVRRKPYSVVLLDEVEKAHPD
VHEIFFQVFDKGVMEDGEGRVIDFKNTLILLTTNAGTEMIASLCADPELMPEPEAIAKSL
REPLLKIFPPALLGRLVTIPYYPLSDDMLKAISRLQLGRIKKRVEATHKVPFEFDEGVVD
LIVSRCTETESGGRMIDAILTNTLLPDMSREFLTRMLEGKPLAGVRISSRDNQFHYDFAE
AE
>P76346
MIKWPWKVQESAHQTALPWQEALSIPLLTCLTEQEQSKLVTLAERFLQQKRLVPLQGFEL
DSLRSCRIALLFCLPVLELGLEWLDGFHEVLIYPAPFVVDDEWEDDIGLVHNQRIVQSGQ
SWQQGPIVLNWLDIQDSFDASGFNLIIHEVAHKLDTRNGDRASGVPFIPLREVAGWEHDL
HAAMNNIQEEIELVGENAASIDAYAASDPAECFAVLSEYFFSAPELFAPRFPSLWQRFCQ

FYQQDPLQRLHHANDTDSFSATNVH
>P0A805
MISDIRKDAEVRMDKCVEAFKTQISKIRTGRASPSLLDGIVVEYYGTPTPLRQLASVTVE
DSRTLKINVFDRSMSPAVEKAIMASDLGLNPNSAGSDIRVPLPPLTEERRKDLTKIVRGE
AEQARVAVRNVRRDANDKVKALLKDKEISEDDDRRSQDDVQKLTDAAIKKIEAALADKEA
ELMQF
>P12994
MKLISNDLRDGDKLPHRHVFNGMGYDGDNISPHLAWDDVPAGTKSFVVTCYDPDAPTGSG
WWHWVVVNLPADTRVLPQGFGSGLVAMPDGVLQTRTDFGKTGYDGAAPPKGETHRYIFTV
HALDIERIDVDEGASGAMVGFNVHFHSLASASITAMFS
>P26477
MSIDRTSPLKPVSTVQTRETSDTPVQKTRQEKTSAATSASVTLSDAQAKLMQPGVSDINM
ERVEALKTAIRNGELKMDTGKIADSLIREAQSYLQSK
>P45573
MAEVTYKGKSFEVDEDGFLLRFDDWCPEWVEYVKESEGISDISPDHQKIIDFLQDYYKKN
GIAPMVRILSKNTGFKLKEVYELFPSGPGKGACKMAGLPKPTGCV
>P22608
MNDSIQLSGLSRQLVQANLLDEKTAVQAQAQAQRNKLSLVTHLVQSKLVSGLALAELSAE
QFGIAYCDLNSLDKESFPRDAISEKLVRQHRVIPLWRRGNKLFVGISDPANHQAINDVQF
STGLTTEAILVEDDKLGLAIDKLFESATDGLAGLDDVDLEGLDIGSADKSTQEDASAEAD
DAPVVRFVNKMLLDAIKGGSSDLHFEPYEKIYRVRFRTDGMLHEVAKPPIQLASRISARL
KVMAGLDISERRKPQDGRIKMRVSKTKSIDFRVNTLPTLWGEKIVMRILDSSSAQMGIDA
LGYEEDQKELYLAALKQPQGMILVTGPTGSGKTVSLYTGLNILNTTDINISTAEDPVEIN
LEGINQVNVNPRQGMDFSQALRAFLRQDPDVIMVGEIRDLETAEIAIKAAQTGHMVMSTL
HTNSAAETLTRLLNMGVPAFNLATSVNLIIAQRLARKLCSHCKKEHEVPRETLLHEGFPE
DKIGTFKLYSPVGCDHCKNGYKGRVGIYEVVKNTPALQRIIMEEGNSIEIAEQARKEGFN
DLRTSGLLKAMQGITSLEEVNRVTKD
>Q1DB04
MSFINYSSREINCKIVYYGPGLCGKTTNLQYIYNKTAAETKGKLISLSTETDRTLFFDFL
PLSLGEIRGFKTRFHLYTVPGQVFYDASRKLILKGVDGVVFVADSQIERMEANMESLENL
RINLAEQGYDLNKIPYVIQYNKRDLPNAVTVEEMRKALNHRNIPEYQAVAPTGVGVFDTL
KAVAKLVLTELKKGG
>P23869
MVTFHTNHGDIVIKTFDDKAPETVKNFLDYCREGFYNNTIFHRVINGFMIQGGGFEPGMK
QKATKEPIKNEANNGLKNTRGTLAMARTQAPHSATAQFFINVVDNDFLNFSGESLQGWGY
CVFAEVVDGMDVVDKIKGVATGRSGMHQDVPKEDVIIESVTVSE
>P0A6P9
MSKIVKIIGREIIDSRGNPTVEAEVHLEGGFVGMAAAPSGASTGSREALELRDGDKSRFL
GKGVTKAVAAVNGPIAQALIGKDAKDQAGIDKIMIDLDGTENKSKFGANAILAVSLANAK
AAAAAKGMPLYEHIAELNGTPGKYSMPVPMMNIINGGEHADNNVDIQEFMIQPVGAKTVK
EAIRMGSEVFHHLAKVLKAKGMNTAVGDEGGYAPNLGSNAEALAVIAEAVKAAGYELGKD
ITLAMDCAASEFYKDGKYVLAGEGNKAFTSEEFTHFLEELTKQYPIVSIEDGLDESDWDG
FAYQTKVLGDKIQLVGDDLFVTNTKILKEGIEKGIANSILIKFNQIGSLTETLAAIKMAK
DAGYTAVISHRSGETEDATIADLAVGTAAGQIKTGSMSRSDRVAKYNQLIRIEEALGEKA

PYNGRKEIKGQA
>P76008
MSLFHLIAPSGYCIKQHAALRGIQRLTDAGHQVNNVEVIARRCERFAGTETERLEDLNSL
ARLTTPNTIVLAVRGGYGASRLLADIDWQALVARQQHDPLLICGHSDFTAIQCGLLAHGN
VITFSGPMLVANFGADELNAFTEHHFWLALRNETFTIEWQGEGPTCRAEGTLWGGNLAML
ISLIGTPWMPKIENGILVLEDINEHPFRVERMLLQLYHAGILPRQKAIILGSFSGSTPND
YDAGYNLESVYAFLRSRLSIPLITGLDFGHEQRTVTLPLGAHAILNNTREGTQLTISGHP
VLKM
>P80354
MITRETLKSLPANVQAPPYDIDGIKPGIVHFGVGNFFRAHEAFYVEQILEHAPDWAIVGV
GLTGSDRSKKKAEEFKAQDCLYSLTETAPSGKSTVRVMGALRDYLLAPADPEAVLKHLVD
PAIRIVSMTITEGGYNINETTGAFDLENAAVKADLQNPEKPSTVFGYVVEALRRRRDAGG
KAFTVMSCDNLRHNGNVARKAFLGYAKARDPELAKWIEENATFPNGMVDRITPTVSAEIA
KKLNAASGLDDDLPLVAEDFHQWVLEDRFANGRPPLEKAGVQLVDDVTDWEHVKIRMLNA
GHITLCFPGILVGYENVDDAIEDKDLRGNLENYLNKDVIPTLKAPPGMTLEGYRDSVISR
FSNKAMSDQTLRIASDGCSKIQVFWTETVRRAIECKRDLSRIAFGIASYLEMLRGRDEKG
GTYESSEPTYGEAQKKLAKADDFESALKLPAFDGWRDLDTSELDQKVIALRKVIREKGVK
AAIPA
>P06722
MSQPRPLLSPPETEEQLLAQAQQLSGYTLGELAALVGLVTPENLKRDKGWIGVLLEIWLG
ASAGSKPEQDFAALGVELKTIPVDSLGRPLETTFVCVAPLTGNSGVTWETSHVRHKLKRV
LWIPVEGERSIPLAQRRVGSPLLWSPNEEEDRQLREDWEELMDMIVLGQVERITARHGEY
LQIRPKAANAKALTEAIGARGERILTLPRGFYLKKNFTSALLARHFLIQ
>P24251
MTLPSGHPKSRLIKKFTALGPYIREGKCKDNRFFFDCLAVCVNVKPAPEVREFWGWWMEL
EAQESRFTYSYQFGLFDKAGDWKSVPVKDTEVVERLEHTLREFHEKLRELLTTLNLKLEP
ADDFRDEPVKLTA
>P33012
MNYEIKQEEKRTVAGFHLVGPWEQTVKKGFEQLMMWVDSKNIVPKEWVAVYYDNPDETPA
EKLRCDTVVTVPGYFTLPENSEGVILTEITGGQYAVAVARVVGDDFAKPWYQFFNSLLQD
SAYEMLPKPCFEVYLNNGAEDGYWDIEMYVAVQPKHH
>A6SX19
MKPAKEIRVGNIIMVDSKPMIVLRSDVNGSSRTGFTYKWKMKNLLTNTPMENVFRGDDKF
DVVVLDKKPVTYSYFADPLYVFMDEEYNQYEIEEENLGDALHYLKDGMECEAVFYDGKAI
SVELPITIARQVVYSEPAVKGNTSGNVLKEAKIENAVEAHRHTVQVPLFVSQDDVIEIDT
RTNEYKRVVRN
>P82998
MLKLHGFSVSNYYNMVKLALLEKGLPFEEVTFYGGQAPQALEVSPRGKVPVLETEHGFLS
ETSVILDYIEQTQSGKALLPADPFEQAKVRELLKEIELYIELPARTCYAESFFGMSVEPL
IKEKARADLLAGFATLKRNGRFAPYVAGEQLTLADLMFCFSVDLANAVGKKVLSIDFLAD
FPQAKALLQLMGENPHMARIMADKEASMPAFMEMIRSGKR
>P07642
MYHRMAHESQPNPLLPGYSFNAYLVAGLTPILAEGPLDFFIDRPDGMKGYIINLTMKGQG
QIFDGDETFFCNPGDLLLFPPKSTHFYGRSPSSDCWYHRWVYFRPRAYWADWLEWHTKSS

GIGRMSLPNNQLLLEFDRLFANIEQTQRSGRRFSEELGMNLLERLLLRAMEEDPQSPQKI
MDPRVIEACQFITSNLAGELRIDEVARHVCLSPSRLAHLFREQVGINILRWREDQRVIRA
KLLLQTTQESIANIGRVVGYDDQLYFSRVFRKRVGVSPSDFRRRSSEINYPAAKTLPVAW
GEQIPHAVSS
>P0A9C9
MRRELAIEFSRVTESAALAGYKWLGRGDKNTADGAAVNAMRIMLNQVNIDGTIVIGEGEI
DEAPMLYIGEKVGTGRGDAVDIAVDPIEGTRMTAMGQANALAVLAVGDKGCFLNAPDMYM
EKLIVGPGAKGTIDLNLPLADNLRNVAAALGKPLSELTVTILAKPRHDAVIAEMQQLGVR
VFAIPDGDVAASILTCMPDSEVDVLYGIGGAPEGVVSAAVIRALDGDMNGRLLARHDVKG
DNEENRRIGEQELARCKAMGIEAGKVLRLGDMARSDNVIFSATGITKGDLLEGISRKGNI
ATTETLLIRGKSRTIRRIQSIHYLDRKDPEMQVHIL
>P0A6F9
MNIRPLHDRVIVKRKEVETKSAGGIVLTGSAAAKSTRGEVLAVGNGRILENGEVKPLDVK
VGDIVIFNDGYGVKSEKIDNEEVLIMSESDILAIVEA
>P0A6R0
MYTKIIGTGSYLPEQVRTNADLEKMVDTSDEWIVTRTGIRERHIAAPNETVSTMGFEAAT
RAIEMAGIEKDQIGLIVVATTSATHAFPSAACQIQSMLGIKGCPAFDVAAACAGFTYALS
VADQYVKSGAVKYALVVGSDVLARTCDPTDRGTIIIFGDGAGAAVLAASEEPGIISTHLH
ADGSYGELLTLPNADRVNPENSIHLTMAGNEVFKVAVTELAHIVDETLAANNLDRSQLDW
LVPHQANLRIISATAKKLGMSMDNVVVTLDRHGNTSAASVPCALDEAVRDGRIKPGQLVL
LEAFGGGFTWGSALVRF
>Q00594
MLGQMMRNQLVIGSLVEHAARYHGAREVVSVETSGEVTRSCWKEVELRARKLASALGKMG
LTPSDRCATIAWNNIRHLEVYYAVSGAGMVCHTINPRLFIEQITYVINHAEDKVVLLDDT
FLPIIAEIHGSLPKVKAFVLMAHNNSNASAQMPGLIAYEDLIGQGDDNYIWPDVDENEAS
SLCYTSGTTGNPKGVLYSHRSTVLHSMTTAMPDTLNLSARDTILPVVPMFHVNAWGTPYS
AAMVGAKLVLPGPALDGASLSKLIASEGVSIALGVPVVWQGLLAAQAGNGSKSQSLTRVV
VGGSACPASMIREFNDIYGVEVIHAWGMTELSPFGTANTPLAHHVDLSPDEKLSLRKSQG
RPPYGVELKIVNDEGIRLPEDGRSKGNLMARGHWVIKDYFHSDPGSTLSDGWFSTGDVAT
IDSDGFMTICDRAKDIIKSGGEWISTVELESIAIAHPHIVDAAVIAARHEKWDERPLLIA
VKSPNSELTSGEVCNYFADKVARWQIPDAAIFVEELPRNGTGKILKNRLREKYGDILLRS
SSSVCE
>P60919
MAEKPKKPQKLRARLPRGLADRGPAEIAATRAMVEKIREVYERYGFEPVETPAFEYTDAL
GKFLPDQDRPNEGVFSLQDDDEQWISLRYDLTAPLARYVAENFDQLPKPHRSYRFGWVFR
NEKPGPGRFRQFMQFDADTVGAPTAAADAEMCMMAADTMEALGIPRGSYVVKVNNRKVLD
GVLESIGLGGDENAGRRLTVLRAIDKSDKFAPEEIKKLLGPGRWDGGEEGKGDFTKGAML
SDDQSELILRATSPSFIAGRFNADGSGGISNIDTVELLRSTADNETLKQGCDELAVIADL
LDACGYGATSANPDVRVVIDPSVVRGLEYYTGPVYEVELLLDTKDEKGRPVRFGSVGGGG
RYDGLVSRFRGEPVPATGFSIGVSRLQAALTLIGQLGNKPQAGPVVVTVFGGEIAGYQKM
VATLRKAGIRAELYLGNPKHSLGQQMKYADKRNSPCAIIQGSDEKQQGIVQIKDLILGAE
LASLEKDRDEYLKKQAEAQFSCNEDEMVAKVQELLQRRGVAWG
>P39405
MAYRSAPLYEDVIWRTHLQPQDPTLAQAVRATIAKHREHLLEFIRLDEPAPLNAMTLAQW

SSPNVLSSLLAVYSDHIYRNQPMMIRENKPLISLWAQWYIGLMVPPLMLALLTQEKALDV
SPEHFHAEFHETGRVACFWVDVCEDKNATPHSPQHRMETLISQALVPVVQALEATGEING
KLIWSNTGYLINWYLTEMKQLLGEATVESLRHALFFEKTLTNGEDNPLWRTVVLRDGLLV
RRTCCQRYRLPDVQQCGDCTLK
>O06644
MTKPLDGINVLDFTHVQAGPACTQMMGFLGANVIKIERRGSGDMTRGWLQDKPNVDSLYF
TMFNCNKRSIELDMKTPEGKELLEQMIKKADVMVENFGPGALDRMGFTWEYIQELNPRVI
LASVKGYAEGHANEHLKVYENVAQCSGGAAATTGFWDGPPTVSGAALGDSNSGMHLMIGI
LAALEMRHKTGRGQKVAVAMQDAVLNLVRIKLRDQQRLERTGILAEYPQAQPNFAFDRDG
NPLSFDNITSVPRGGNAGGGGQPGWMLKCKGWETDADSYVYFTIAANMWPQICDMIDKPE
WKDDPAYNTFEGRVDKLMDIFSFIETKFADKDKFEVTEWAAQYGIPCGPVMSMKELAHDP
SLQKVGTVVEVVDEIRGNHLTVGAPFKFSGFQPEITRAPLLGEHTDEVLKELGLDDAKIK
ELHAKQVV
>Q7VK65
MIFSKHLLSHFVDISHLDIEQMCMRLSSMGLEVESAYPLKMPQKVVVGKILSLTPHPDAD
KLNVCKVSIGSQELQIVCGANNVKANQYVAVALEGAVIPHTKSGEIVIKQTNLRGIESCG
MLCSSVELGLPKINDGIMVLDSTAGHLELGVELGNLPLFNDYVIEVGITPNRGDCLSVLG
IARELATSYDLRLKHEVDMDNVVTLGLGRVLQILCDEKIEAHLLYRVVEVKQAYLPLDIA
LCLARNGSLVDDIMCNFLEYGTYMTGVILNAYKLYDCENKDIVLDNGLVAQLRIKKDENG
LGAVFAHQKLSIIGVSYGERHFGTRSEIYIIEASYVNPTLIAKSLYKHAIKGDVQLTYRS
TRGSNPNLEQGIDFLCRKMVLVSDALVYSGSHNIVQNIDEITIKTTFKAINQIIGIELDK
EEIATILKRLNFKLDATCDENFFMVTVPNYRHDIQSIQDVAEEVLRIYGIDNVSSVPLLC
SQSHNINNTYFTYKNTRKLAYGLIAYGFVECIHYVFASSQNLERLGFVRLDEDLELLNPI
TNELDTLRTSLLPAMLDSVKRNENLGFKNITLFEIGSVYSSKREEKSKLALVASGCMQDE
CYPHTKAVKWNLFAFGTICQRCVGDLSFRNIRDEANAKELLSHFCFTDERLLHPYQSAFV
YQKDKPIGIIAKLHPQVATNMDLSEEIFICEIEIGMSDFSLPQAYEFSKYQKSTRDLTIL
IDKDIPFYRVRQILLEDQIAYVKNIYPIDVYYDKALGEKMALSIRIVLQSDEGTLQEMQL
VQAVESVLSVLVREFDAVLRT
>P32099
MSTLRLLISDSYDPWFNLAVEECIFRQMPATQRVLFLWRNADTVVIGRAQNPWKECNTRR
MEEDNVRLARRSSGGGAVFHDLGNTCFTFMAGKPEYDKTISTSIVLNALNALGVSAEASG
RNDLVVKTVEGDRKVSGSAYRETKDRGFHHGTLLLNADLSRLANYLNPDKKKLAAKGITS
VRSRVTNLTELLPGITHEQVCEAITEAFFAHYGERVEAEIISPNKTPDLPNFAETFARQS
SWEWNFGQAPAFSHLLDERFTWGGVELHFDVEKGHITRAQVFTDSLNPAPLEALAGRLQG
CLYRADMLQQECEALLVDFPEQEKELRELSAWMAGAVR
>P56104
MKQLFLIIGAPGSGKTTDAELIAKNNSETIAHFSTGDLLRAESAKKTERGLLIEKFTSQG
ELVPLEIVVETILSAIKSSGKGIILIDGYPRSVEQMQALDKELNAQNEVILKSVIEVEVS
ENTAKERVLGRSRGADDNEKVFHNRMRVFLDPLGEIQNFYKNKKVYKAIDGERSIEEIVG
EMQEYILSFGN
>P23830
MLSKFKRNKHQQHLAQLPKISQSVDDVDFFYAPADFRETLLEKIASAKQRICIVALYLEQ
DDGGKGILNALYEAKRQRPELDVRVLVDWHRAQRGRIGAAASNTNADWYCRMAQENPGVD
VPVYGVPINTREALGVLHFKGFIIDDSVLYSGASLNDVYLHQHDKYRYDRYHLIRNRKMS

DIMFEWVTQNIMNGRGVNRLDDVNRPKSPEIKNDIRLFRQELRDAAYHFQGDADNDQLSV
TPLVGLGKSSLLNKTIFHLMPCAEQKLTICTPYFNLPAILVRNIIQLLREGKKVEIIVGD
KTANDFYIPEDEPFKIIGALPYLYEINLRRFLSRLQYYVNTDQLVVRLWKDDDNTYHLKG
MWVDDKWMLITGNNLNPRAWRLDLENAILIHDPQLELAPQREKELELIREHTTIVKHYRD
LQSIADYPVKVRKLIRRLRRIRIDRLISRIL
>P75949
MGPVMLDVEGYELDAEEREILAHPLVGGLILFTRNYHDPAQLRELVRQIRAASRNRLVVA
VDQEGGRVQRFREGFTRLPAAQSFAALSGMEEGGKLAQEAGWLMASEMIAMDIDISFAPV
LDVGHISAAIGERSYHADPQKALAIASRFIDGMHEAGMKTTGKHFPGHGAVTADSHKETP
CDPRPQAEIRAKDMSVFSSLIRENKLDAIMPAHVIYSDVDPRPASGSPYWLKTVLRQELG
FDGVIFSDDLSMEGAAIMGSYAERGQASLDAGCDMILVCNNRKGAVSVLDNLSPIKAERV
TRLYHKGSFSRQELMDSARWKAISTRLNQLHERWQEEKAGH
>P12758
MSKSDVFHLGLTKNDLQGATLAIVPGDPDRVEKIAALMDKPVKLASHREFTTWRAELDGK
PVIVCSTGIGGPSTSIAVEELAQLGIRTFLRIGTTGAIQPHINVGDVLVTTASVRLDGAS
LHFAPLEFPAVADFECTTALVEAAKSIGATTHVGVTASSDTFYPGQERYDTYSGRVVRHF
KGSMEEWQAMGVMNYEMESATLLTMCASQGLRAGMVAGVIVNRTQQEIPNAETMKQTESH
AVKIVVEAARRLL
>P13024
MSIRIIPQDELGSSEKRTADMIPPLLFPRLKNLYNRRAERLRELAENNPLGDYLRFAALI
AHAQEVVLYDHPLEMDLTARIKEASAQGKPPLDIHVLPRDKHWQKLLMALIAELKPEMSG
PALAVIENLEKASTQELEDMASALFASDFSSVSSDKAPFIWAALSLYWAQMANLIPGKAR
AEYGEQRQYCPVCGSMPVSSMVQIGTTQGLRYLHCNLCETEWHVVRVKCSNCEQSGKLHY
WSLDDEQAAIKAESCDDCDTYLKILYQEKDPKIEAVADDLASLVLDARMEQEGYARSSIN
PFLFPGEGE
>P0A9D8
MQQLQNIIETAFERRAEITPANADTVTREAVNQVIALLDSGALRVAEKIDGQWVTHQWLK
KAVLLSFRINDNQVIEGAESRYFDKVPMKFADYDEARFQKEGFRVVPPAAVRQGAFIARN
TVLMPSYVNIGAYVDEGTMVDTWATVGSCAQIGKNVHLSGGVGIGGVLEPLQANPTIIED
NCFIGARSEVVEGVIVEEGSVISMGVYIGQSTRIYDRETGEIHYGRVPAGSVVVSGNLPS
KDGKYSLYCAVIVKKVDAKTRGKVGINELLRTID
>P59505
MEFSEKWLLDWLGFSISNVFYEQMTKSGIEVEAIAKISKNFERVIVGEVVERLYVNTMHN
IVFLRVKLSEKKMIFSISSNDIEFSRGTKIAIATQDSKLFNNRLISMLRFKEKISEGMVC
SFKDLGILNIKNKIVEICSEVPVGTDISKFLWFDDDRIIKVSSAPNRADGMSILGIARDM
SALNNLCLPTLKEYHINITNHEKFRILINIPDVCLNFIGRTIQSVNLNRQTPLWILERLR
RSSISSENVLVDIINYVLIELGQPIFSFNIHGIVQNIIIRTARDNEQFFDSCSQRVPIDK
RTILLSDDKEILVLGNHTNSYNSRLSLSSHNIFLGCALFNPEYINNDSHFNFGFKNKITE
YYSRGVDSDIQYKALNYVTYLVLKICGGNASNVVLANSSRVTVIQKKIFVLKKTLHRYVN
NIISDTLVVKYLLQLGYLVEKQKHCWLVIPPSWRFDIQIQEDVISDLVRVFGYHNIPACA
LVTNYKLVHDDNIYTSLNRIKLLLVDLGYNEVITYSFVDSQIQKYLFPKRKQFFLLNPIS
RKMSSMRLSLWNGLLSSVLYNQNRQEKVMRFFESGLCFEEDGNEYLGVKQDLYLAGVISG
YKNETDWRSFNKIVSFYDLKGDIELIMALLRKLDKVSFKKMLFQNLCPKQSAAIYFEREM
IGVIGVISSNISKKMGLKYKTIVFELIWKKIAQSNDYRIRDVSLYPRCSRDISIIVNDSI

AADEILKVSKNVFLDKIVEVKLFDVFYGKNVGLNKKSLSLRFIFGSSKRTLSEEIISNCL
NECIRILQEKFNAILRDRNFLF
>Q9I317
MMTALETRLSVADGTHAAALRQRLQAALAECRRELARGACPEHFQFLQQQARALEGGLGI
LSQLTED
>Q62LE0
MSASDLTSVQAGAPQGRRQILVTSALPYANGQIHIGHLVEYIQTDIWVRTMRMHGHEIYY
IGADDTHGTPVMLRAEQEGVSPKQLIERVWREHKRDFDSFGVSFDNFYTTDSDENRVLSE
TIYLALKEAGFIAEREIEQAYDPVRQMFLPDRFIKGECPKCHAKDQYGDSCEVCGTTYQP
TDLIHPYSVVSGAAPVRKTSTHYFFRLSDPRCEAFLREWVSGLAQPEATNKMREWLGEAG
EAKLADWDISRDAPYFGFEIPGAPGKYFYVWLDAPVGYYASFKNLCQRRGLDFDAWIRKD
STTEQYHFIGKDILYFHTLFWPAMLEFSGHRTPTNVFAHGFLTVDGAKMSKSRGTFITAQ
SYIDTGLNPEWLRYYFAAKLNATMEDIDLNLEDFQARVNSDLVGKYVNIASRAAGFLLKR
FDGRVQASAMNHPLLATLRGAIPQIAAHYEAREYGRALRQTMELADAVNGYVDSAKPWEL
AKDPANAVALHETCSVSLEAFRLLSLALKPVLPRVAQGVEAFLGIAPLTWADAGTPLSPE
QPVRAYQHLMTRVDPKQIDALLAANRGSLQGTAAAAEAGAANGNGAGSKNGKGAKAAAQP
AASAANADDGASPIISIDDFAKIDLRIAKIVACQAVEGSDKLLQLTLDVGEERTRNVFSG
IKSAYRPEQLVGKLTVMVANLAPRKMKFGLSEGMVLAASAADEKAEPGLYILEPHSGAKP
GMRVK
>O85300
MGLPLTFDDNNQCLLLLDSDIFTSIEAKDDIWLLNGMIIPLSPVCGDSIWRQIMVINGEL
AANNEGTLAYIDAAETLLLIHAITDLTNTYHIISQLESFVNQQEALKNILQEYAKV
>Q0K9H3
MHSTQIPPQQKQKRRLRLTVLAAAASMLAAACVSGDDNNNGNGSNPNTKPANIGTVTINS
YNGTTDDLLTAGLGKDGLASATAPLPANPTAPTAAELRRYAIHTNYRAIVDTTASGGYGS
LYGPNVDAQGNVTGSDGKVAGVEYLAFSDDGSGQQNVTMLVQIPASFNTSKPCMITATSS
GSRGVYGAIATGEWGLKRGCAVAYTDKGTGAAPHDLDTDTVPLIDGTRATRAAAGKNAQF
AAPAGATSLADFTAANPHRLAFKHAHSQRNPEKDWGKFTLQAVEFAIWAINDRFGAVSAN
GTRQRTLDKDRIVVIASSVSNGGGAAVAAAEQDAGGLIDGVAVGEPNLNMPPNTGIVVQR
GATPVAASGRTLYDYTTTANLLQHCAARATALTQAPFYTNPATATFFANRCQTLAEKGLV
SGANTDEQSASALQALHDAGWEAESDDLHPSLAVFDVAAAISVNYANAYAQASVTDRLCG
YSFASTLTDLKPAAIAPAALASMFATGNGVPPQPPVQLINDLDPQHGPYLNLASVSPSTL
REDLNYDGANCLRSLLAGSDAAARALQAGQALTLRNGNLRGKPAVIVHGRSDGLLPVNHT
SRPYLGLNRQQEGVTSKLSYVEVENAQHFDAFIGLVPGYSNRYVPLHVYLNRALDAVYDN
LTAGKALPPSQVLRTTPRGGTLNTPAPALLPSNVPPFAASPAAGNAITVNANAVQVPD
>P0A6Z3
MKGQETRGFQSEVKQLLHLMIHSLYSNKEIFLRELISNASDAADKLRFRALSNPDLYEGD
GELRVRVSFDKDKRTLTISDNGVGMTRDEVIDHLGTIAKSGTKSFLESLGSDQAKDSQLI
GQFGVGFYSAFIVADKVTVRTRAAGEKPENGVFWESAGEGEYTVADITKEDRGTEITLHL
REGEDEFLDDWRVRSIISKYSDHIALPVEIEKREEKDGETVISWEKINKAQALWTRNKSE
ITDEEYKEFYKHIAHDFNDPLTWSHNRVEGKQEYTSLLYIPSQAPWDMWNRDHKHGLKLY
VQRVFIMDDAEQFMPNYLRFVRGLIDSSDLPLNVSREILQDSTVTRNLRNALTKRVLQML
EKLAKDDAEKYQTFWQQFGLVLKEGPAEDFANQEAIAKLLRFASTHTDSSAQTVSLEDYV
SRMKEGQEKIYYITADSYAAAKSSPHLELLRKKGIEVLLLSDRIDEWMMNYLTEFDGKPF

QSVSKVDESLEKLADEVDESAKEAEKALTPFIDRVKALLGERVKDVRLTHRLTDTPAIVS
TDADEMSTQMAKLFAAAGQKVPEVKYIFELNPDHVLVKRAADTEDEAKFSEWVELLLDQA
LLAERGTLEDPNLFIRRMNQLLVS
>P0A8S9
MHTSELLKHIYDINLSYLLLAQRLIVQDKASAMFRLGINEEMATTLAALTLPQMVKLAET
NQLVCHFRFDSHQTITQLTQDSRVDDLQQIHTGIMLSTRLLNDVNQPEEALRKKRA
>P31473
MAHPHLLAERISRLSSSLEKGLYERSHAIRLCLLAALSGESVFLLGPPGIAKSLIARRLK
FAFQNARAFEYLMTRFSTPEEVFGPLSIQALKDEGRYERLTSGYLPEAEIVFLDEIWKAG
PAILNTLLTAINERQFRNGAHVEKIPMRLLVAASNELPEADSSLEALYDRMLIRLWLDKV
QDKANFRSMLTSQQDENDNPVPDALQVTDEEYERWQKEIGEITLPDHVFELIFMLRQQLD
KLPDAPYVSDRRWKKAIRLLQASAFFSGRSAVAPVDLILLKDCLWYDAQSLNLIQQQIDV
LMTGHAWQQQGMLTRLGAIVQRHLQLQQQSDKTALTVIRLGGIFSRRQQYQLPVNVTAS
TLTLLLQKPLKLHDMEVVHISFERSALEQWLSKGGEIRGKLNGIGFAQKLNLEVDSAQHL
VVRDVSLQGSTLALPGSSAEGLPGEIKQQLEELESDWRKQHALFSEQQKCLFIPGDWLGR
IEASLQDVGAQIRQAQQC
>P0A2Q4
MIEIKHLKTLQALRNSGSLAAAAAVLHQTQSALSHQFSDLEQRLGFRLFVRKSQPLRFTP
QGEVLLQLANQVLPQISRALQACNEPQQTRLRIAIECHSCIQWLTPALENFRASWPQVEM
DFTSGVTFDPQPALQQGELDLVMTSDILPRSGLHYSPMFDFEVRLVLAPDHPLASKTQIT
PEDLASETLLIYPVQRSRLDVWRHFLQPAGISPLLKSVDNTLLLIQMVAARMGIAALPHW
VVESVERQGLVVTKTLGDGLWSRLYAAVRDGDQRQAVTEAFIRSTRDHACDHLPFVRSAE
RPIFDAPTAKPGSQPRL
>P55711
MSASNLLPMISSNPAQFAQASLAKAFAPRVAQGQQSVLSFEAMLSTNMLDRIGPLASRED
LPPPDAESTLEDLQKDPLALLPPHMRAAIESMDQTPQSAVVIDDHYVAPAPIQSSRITWN
GGSLTKPELQIVAVLNRHKDLCPLSWESLEAKANDPSTPPDLKAAIEALLQDPELFYAIG
SQGDGRCGGKISAKDLSEFSKHHPQVAAFQESQAQSYAQNYIPSDSAENAQPSVMTENDA
LRELYRYSEYLPKNLSLADFKQIVDGEAKTGKCPPQVIAAAQYFVSHPEEWKQLYGGNID
KVHKEDFLQVASSSMSLTQAELDTLKTINSHQELFFGSGDLTRDKLASMADDKSLDPKVR
EAASQLLSDPLLFGLLNNAITGYKTHHGFFDFGGGHTVDSGNVSKEDFGRFYTNMTTANR
TVQQPKFHVPETEAAQNAVADMKMGLADQPDIKSPKKNGGALMHVVDSVLRVGSKVLDWA
ATAVGVLSFIPGIGQVADLVSMTLACEAQAANLLRTAITGGNMKQALIEAGIGVAAQAVG
LVSGPGVKLAIRNGLARKAIEEAATAGINLPLSMAQHYAEGYLNDLKARLAADHPA
>Q057T0
MKISVKLIKELRIKTGSGYLECKRALQKSNGNIINAINYLRIVGTDIAQRKVLRKTKFGR
IFSYCSKNLGVLLELTSETDFVSKNEEFKNFGEKIVNFSGNNKIFDLTEINEIFNSKKIN
FISRVRENIEINKIKYITGNIIESYQHLGKIGVIISGKMLSPNTHLNTTKCFKNIAMHVA
AAAPLYLSELDIPNNVLQKETDIQKSIAKKTGKSSKILQAIIKGRLKKFISEITLINQNF
IINPKITIHDYLKENQVWINNFIRLQVGENIDNLNT
>A4XZ93
MARNTAINRYRNIGICAHVDAGKTTTTERILFYTGLSHKMGEVHDGAATTDWMVQEQERG
ITITSAAITTFWEGSRRQYDKYRVNVIDTPGHVDFTIEVERSLRVLDGAVVVFCGTSGVE
PQSETVWRQANKYGVPRIVYVNKMDRQGANFLRVVGQIKNRLGHTPVPIQIAIGAEENFE

GQVDLIKMKAIYWNDDDKGTTYREEEIPAELVDLANEWRSNMVEAAAEASEELMNKYLEE
GDLSAEDIKAGLRARTLASEIVPAVCGSSFKNKGVPLVLDAVIDFLPAPTEIPAIKGIHP
DLADKPKEQMEESDYDERHADDNEPFSALAFKIATDPFVGTLTFVRVYSGVLESGQSVIN
SVKGKKERVGRMVQMHANQRDEIKEVRAGDIAALIGMKDVTTGETLCDPDKPIILERMDF
PEPVISVAVEPKTKADQEKMGIALGKLAQEDPSFRVKTDEETGQTIISGMGELHLDILVD
RMKREFNVEANIGKPQVSYRETITKDSVEIEGKFVRQSGGRGQFGHCWIRFSAPDVDDKG
NITEGLVFTNEVVGGVVPKEYIPAIQKGIEEQMKNGVVAGYPLIGLKATVFDGSYHDVDS
NEMAFKIAASMATKQLAQKGGGKVLEPIMKVEVVTPEDYMGDVMGDLNRRRGLIQGMEDS
VSGKVIRAEVPLGEMFGYATDVRSMSQGRASYSMEFSKYAEAPSNIVEALVKKQG
>P0A9K7
MDSLNLNKHISGQFNAELESIRTQVMTMGGMVEQQLSDAITAMHNQDSDLAKRVIEGDKN
VNMMEVAIDEACVRIIAKRQPTASDLRLVMVISKTIAELERIGDVADKICRTALEKFSQQ
HQPLLVSLESLGRHTIQMLHDVLDAFARMDIDEAVRIYREDKKVDQEYEGIVRQLMTYMM
EDSRTIPSVLTALFCARSIERIGDRCQNICEFIFYYVKGQDFRHVGGDELDKLLAGKDSD
K
>P10908
MSNWPYPRIVAHRGGGKLAPENTLASIDVGAKYGHKMIEFDAKLSKDGEIFLLHDDNLER
TSNGWGVAGELNWQDLLRVDAGSWYSKMFKGEPLPLLSQVAERCREHGMMANIEIKPTTG
TGPLTGKMVALAARELWAGMTPPLLSSFEIDALEAAQQAAPELPRGLLLDEWRDDWRELT
ARLGCVSIHLNHKLLNKARVMQLKDAGLRILVYTVNKPQRAAELLRWGVDCICTDAIDVI
GPNFTAQ
>P77915
MLDTIRWDADLIRRYDLSGPRYTSYPTAVQFHEGIGPFDQLHALRDSRKAGHPLSLYVHI
PFCANICYYCACNKVITKDRGRSAPYLARLVREIEIVSRHLSREQVVEQLHFGGGTPTFL
SPGQLRELMSQLRTHLNLLDDDSGDYGIEIDPREADWSTMGLLRELGFNRVSLGVQDFDM
EVQKAVNRMQTPEETRTIVEAARTLQYRSINLDLIYGLPKQTPDSFARTVDEVIALQPDR
LSVFNYAHLPERFMPQRRINADDLPSPGQKLEMLQRTTEQLAAAGYRYIGMDHFALPDDE
LASAQEDGTLQRNFQGYTTHGHCDLVGLGVSAISQIGDLYSQNSSDINDYQTSLDNGQLA
IRRGLHCNSDDRVRRAVIQQLICHFELAFEDIETEFGIDFRSYFAELWPDLERFAADGLI
RLDAKGIDITSSGRLLVRSICMLFDRYLPSLNRQRFSRVI
>P10183
MELRDLDLNLLVVFNQLLVDRRVSITAENLGLTQPAVSNALKRLRTSLQDPLFVRTHQGM
EPTPYAAHLAEPVTSAMHALRNALQHHESFDPLTSERTFTLAMTDIGEIYFMPRLMDVLA
HQAPNCVISTVRDSSMSLMQALQNGTVDLAVGLLPNLQTGFFQRRLLQNHYVCLCRKDHP
VTREPLTLERFCSYGHVRVIAAGTGHGEVDTYMTRVGIRRDIRLEVPHFAAVGHILQRTD
LLATVPIRLADCCVEPFGLSALPHPVVLPEIAINMFWHAKYHKDLANIWLRQLMFDLFTD

>P03030
MAAVNLRHIEIFHAVMTAGSLTEAAHLLHTSQPTVSRELARFEKVIGLKLFERVRGRLHP
TVQGLRLFEEVQRSWYGLDRIVSAAESLREFRQGELSIACLPVFSQSFLPQLLQPFLARY
PDVSLNIVPQESPLLEEWLSAQRHDLGLTETLHTPAGTERTELLSLDEVCVLPPGHPLAV
KKVLTPDDFQGENYISLSRTDSYRQLLDQLFTEHQVKRRMIVETHSAASVCAMVRAGVGI
SVVNPLTALDYAASGLVVRRFSIAVPFTVSLIRPLHRPSSALVQAFSGHLQAGLPKLVTS
LDAILSSATTA

>P27828
MKVLTVFGTRPEAIKMAPLVHALAKDPFFEAKVCVTAQHREMLDQVLKLFSIVPDYDLNI
MQPGQGLTEITCRILEGLKPILAEFKPDVVLVHGDTTTTLATSLAAFYQRIPVGHVEAGL
RTGDLYSPWPEEANRTLTGHLAMYHFSPTETSRQNLLRENVADSRIFITGNTVIDALLWV
RDQVMSSDKLRSELAANYPFIDPDKKMILVTGHRRESFGRGFEEICHALADIATTHQDIQ
IVYPVHLNPNVREPVNRILGHVKNVILIDPQEYLPFVWLMNHAWLILTDSGGIQEEAPSL
GKPVLVMRDTTERPEAVTAGTVRLVGTDKQRIVEEVTRLLKDENEYQAMSRAHNPYGDGQ
ACSRILEALKNNRISL
>P15977
MESKRLDNAALAAGISPNYINAHGKPQSISAETKRRLLDAMHQRTATKVAVTPVPNVMVY
TSGKKMPMVVEGSGEYSWLLTTEEGTQYKGHVTGGKAFNLPTKLPEGYHTLTLTQDDQRA
HCRVIVAPKRCYEPQALLNKQKLWGACVQLYTLRSEKNWGIGDFGDLKAMLVDVAKRGGS
FIGLNPIHALYPANPESASPYSPSSRRWLNVIYIDVNAVEDFHLSEEAQAWWQLPTTQQT
LQQARDADWVDYSTVTALKMTALRMAWKGFAQRDDEQMAAFRQFVAEQGDSLFWQAAFDA
LHAQQVKEDEMRWGWPAWPEMYQNVDSPEVRQFCEEHRDDVDFYLWLQWLAYSQFAACWE
ISQGYEMPIGLYRDLAVGVAEGGAETWCDRELYCLKASVGAPPDILGPLGQNWGLPPMDP
HIITARAYEPFIELLRANMQNCGALRIDHVMSMLRLWWIPYGETADQGAYVHYPVDDLLS
ILALESKRHRCMVIGEDLGTVPVEIVGKLRSSGVYSYKVLYFENDHEKTFRAPKAYPEQS
MAVAATHDLPTLRGYWECGDLTLGKTLGLYPDEVVLRGLYQDRELAKQGLLDALHKYGCL
PKRAGHKASLMSMTPTLNRGLQRYIADSNSALLGLQPEDWLDMAEPVNIPGTSYQYKNWR
RKLSATLESMFADDGVNKLLKDLDRRRRAAAKKK
>P33771
MKPDAHHVKQFLLRLQDDICQTLSAVDGANFVEDSWRREAGGGGRSRVLRNGGIFEQAGV
NFSHVHGDAMPASATAHRPELAGRSFEAMGVSLVVHPHNPYIPTSHANVRFFIAEKPGAD
PVWWFGGGFDLTPYYGFEEDAVHWHRTARDLCQPFGDDVYPRYKKWCDDYFFLKHRNEQR
GVGGLFFDDLNTPDFDHCFDFMQAVGNGYTRAYLPIVERRKAMVWGERERNFQLYRRGRY
VEFNLVWDRGTLFGLQTGGRTESILMSMPPLVRWEYDWQPEAGSPEAALSEFIQVRDWI
>Q2YQ56
MTVTVRFAPSPTGYIHIGNTRTALSNWLYASKNNGKFILRYDDTDVERSKDEYAQAIAVD
LDWLGVRPDRVEYQSKRFDIYAKAVEKLKTAGLLYACYETADELERRRKFRLARRLPPVY
GREALKLTDAEKAALEAEGRKPHWRFLLPNFESDPFATQRTEVHWDDLVRGPQTVDLASM
SDPILVREDGTYLYTLPSVVDDIDMGVTHIIRGDDHVTNTGVQISIFKALGATPPVFGHH
NLLTTISGEGLSKRTGALSVGSLREAGYEPMAVASLAILIGTSESVTAAPDMAALAEHFD
LASISKSSAKFDPSELDALNRSLLHEMPFEKAKPRLEALGICGAKAESFWLAVRGNLDRF
SDVSHWWQVVSGDLPEAPDLSGEDRDFVRHAFDLLPPEPWNGQTWKSWTEAVKSATGRKG
KNLFMPLRLALTGQAHGPELADLLVLVGLERTKSRRP
>P33697
MNPPAIDKVPDVTFVVAAYNSADTIVRAIESALAQEGVTVEVVVVDDCSADATPALVAAI
PDPRVRLIALDRNRGPGGARNAGIGAARGRWIAVLDSDDTVRPDRLRRMIERADAAGAQI
AVDNLDVVSLDGRSLRMFSEAELARLPQLTLPAFIESNVLFRSEHNFGYMKPIFERRFLE
NQQLRFDEALRIGEDYILLASALACGGRCAVEPSAGYIYHIREGSISRVLRLDHIDAMIA
ADEAFLRRYALDGLAQKMQHRRMRGFREARSFLVLVEQLKKRSLAGALKTALADPFALRH
LSMPIAARLRRLAARFVHPSSHSAPRAAPVTAAAERSPLGNDPRISKG
>P0A7E9

MATNAKPVYKRILLKLSGEALQGTEGFGIDASILDRMAQEIKELVELGIQVGVVIGGGNL
FRGAGLAKAGMNRVVGDHMGMLATVMNGLAMRDALHRAYVNARLMSAIPLNGVCDSYSWA
EAISLLRNNRVVILSAGTGNPFFTTDSAACLRGIEIEADVVLKATKVDGVFTADPAKDPT
ATMYEQLTYSEVLEKELKVMDLAAFTLARDHKLPIRVFNMNKPGALRRVVMGEKEGTLIT
E
>P06149
MSSMTTTDNKAFLNELARLVGSSHLLTDPAKTARYRKGFRSGQGDALAVVFPGSLLELWR
VLKACVTADKIILMQAANTGLTEGSTPNGNDYDRDVVIISTLRLDKLHVLGKGEQVLAYP
GTTLYSLEKALKPLGREPHSVIGSSCIGASVIGGICNNSGGSLVQRGPAYTEMSLFARIN
EDGKLTLVNHLGIDLGETPEQILSKLDDDRIKDDDVRHDGRHAHDYDYVHRVRDIEADTP
ARYNADPDRLFESSGCAGKLAVFAVRLDTFEAEKNQQVFYIGTNQPEVLTEIRRHILANF
ENLPVAGEYMHRDIYDIAEKYGKDTFLMIDKLGTDKMPFFFNLKGRTDAMLEKVKFFRPH
FTDRAMQKFGHLFPSHLPPRMKNWRDKYEHHLLLKMAGDGVGEAKSWLVDYFKQAEGDFF
VCTPEEGSKAFLHRFAAAGAAIRYQAVHSDEVEDILALDIALRRNDTEWYEHLPPEIDSQ
LVHKLYYGHFMCYVFHQDYIVKKGVDVHALKEQMLELLQQRGAQYPAEHNVGHLYKAPET
LQKFYRENDPTNSMNPGIGKTSKRKNWQEVE
>P23619
MKIVSDAKHTGRTRCTIHCQNCSISQLCLPFTLSEHELTQLDNIIERKKPVQKSQIIFQS
GDELRSIYAIRSGTIKSYTISESGEEQITAFHLPGDLVGFDAIMNMKHVGFAQALETSMI
CEIPFDILDDLAGKMPKIRHQIMRLMSNEIKSDQEMILLLSKMSAEEKLAAFLHNLSQRY
AAPGFSAREFRLTMTRGDIGNYLGLTIETISRLLGRFQKSGMITVQGKYITINRMDELTV

>Q04713
MDFCLLNEKSQIFVHAEPYAVSDYVNQYVGTHSIRLPKGGRPAGRLHHRIFGCLDLCRIS
YGGSVRVISPGLETCYHLQIILKGHCLWRGYGQEHYFSPGELLLLNPDDQADLTYSEDCE
KFIVKLPSVVLDRACSDNNWHKPREGIRFAARHNLQQLDGFINLLGLVCDEAEHTKSMPR
VQEHYAGIIASKLLEMLGSNVSREIFSKGNPSFERVVQFIEENLKRNISLERLAELALMS
PRSLYTLFEKHAGTTPKNYIRNRKLECIRARLSDPNANVRSVTEMALDYGFFHTGRFAEN
YRSTFGELPSDTLRRRKMKWLDPEESLPPLP
>P0A8G9
MPKKNEAPASFEKALSELEQIVTRLESGDLPLEEALNEFERGVQLARQGQAKLQQAEQRV
QILLSDNEDASLTPFTPDNE
>P06987
MSQKYLFIDRDGTLISEPPSDFQVDRFDKLAFEPGVIPELLKLQKAGYKLVMITNQDGLG
TQSFPQADFDGPHNLMMQIFTSQGVQFDEVLICPHLPADECDCRKPKVKLVERYLAEQAM
DRANSYVIGDRATDIQLAENMGITGLRYDRETLNWPMIGEQLTRRDRYAHVVRNTKETQI
DVQVWLDREGGSKINTGVGFFDHMLDQIATHGGFRMEINVKGDLYIDDHHTVEDTGLALG
EALKIALGDKRGICRFGFVLPMDECLARCALDISGRPHLEYKAEFTYQRVGDLSTEMIEH
FFRSLSYTMGVTLHLKTKGKNDHHRVESLFKAFGRTLRQAIRVEGDTLPSSKGVL
>P16100
MSTPKIIYTLTDEAPALATYSLLPIIKAFTGSSGIAVETRDISLAGRLIATFPEYLTDTQ
KISDDLAELGKLATTPDANIIKLPNISASVPQLKAAIKELQQQGYKLPDYPEEPKTDTEK
DVKARYDKIKGSAVNPVLREGNSDRRAPLSVKNYARKHPHKMGAWSADSKSHVAHMDNGD
FYGSEKAALIGAPGSVKIELIAKDGSSTVLKAKTSVQAGEIIDSSVMSKNALRNFIAAEI

EDAKKQGVLLSVHLKATMMKVSDPIMFGQIVSEFYKDALTKHAEVLKQIGFDVNNGIGDL
YARIKTLPEAKQKEIEADIQAVYAQRPQLAMVNSDKGITNLHVPSDVIVDASMPAMIRDS
GKMWGPDGKLHDTKAVIPDRCYAGVYQVVIEDCKQHGAFDPTTMGSVPNVGLMAQKAEEY
GSHDKTFQIPADGVVRVTDESGKLLLEQSVEAGDIWRMCQAKDAPIQDWVKLAVNRARAT
NTPAVFWLDPARAHDAQVIAKVERYLKDYDTSGLDIRILSPVEATRFSLARIREGKDTIS
VTGNVLRDYLTDLFPIMELGTSAKMLSIVPLMSGGGLFETGAGGSAPKHVQQFLEEGYLR
WDSLGEFLALAASLEHLGNAYKNPKALVLASTLDQATGKILDNNKSPARKVGEIDNRGSH
FYLALYWAQALAAQTEDKELQAQFTGIAKALTDNETKIVGELAAAQGKPVDIAGYYHPNT
DLTSKAIRPSATFNAALAPLA
>P30335
MPITDLVAPEAILPALKVNSKKQALQELAAKAAELTGQNERAVFEVLLQREKLGTTAVGY
GVAIPHGKLPKLEKIFGLFARLDRPIDFESMDGQPVDLVFLLLAPEGAGADHLKALARIA
RLLRDQDIAKKLRASRDAQAIYSVLALPPATAA
>P0A8P6
MTDLHTDVERYLRYLSVERQLSPITLLNYQRQLEAIINFASENGLQSWQQCDVTMVRNFA
VRSRRKGLGAASLALRLSALRSFFDWLVSQNELKANPAKGVSAPKAPRHLPKNIDVDDMN
RLLDIDINDPLAVRDRAMLEVMYGAGLRLSELVGLDIKHLDLESGEVWVMGKGSKERRLP
IGRNAVAWIEHWLDLRDLFGSEDDALFLSKLGKRISARNVQKRFAEWGIKQGLNNHVHPH
KLRHSFATHMLESSGDLRGVQELLGHANLSTTQIYTHLDFQHLASVYDAAHPRAKRGK
>Q4QLY5
MTTQNFLVEIGTEELPPKALKTLATSFADNVEAELNQAGLSFDKIEWFAAPRRLAVKVLN
LATQQPSKEIEKRGPAVSAAFDAEGKPTKAAEGWARGCGITVEQAERIATDKGEWLVHRT
KIKGQPTKNLLNDIVANALAKLPIPKPMRWADKTVQFIRPVHTVTMLLGDELIEGEILGV
ASARTIRGHRFLGEKEFDIQHADQYPQLLRDKGSVVADFNERKAEILAKSQAKATALGGV
ADIEESLLEEVTSLVEYPNVLAAKFEERFLAVPAEALVYTMKGDQKYFPIYDKDGRLLPH
FIFVSNINPEDPTAIIEGNEKVVRPRLTDAEFFFKTDLKQKLVDRLPRLETVLFQQQLGT
LKDKTDRIEQLAGEIAKQIGADEAKAKRAGLLSKCDLMTNMVFEFTDTQGVMGMHYARHD
GEDEEVAVALNEQYMPRFAGDELPKSLVASAVALADKFDTLTGIFGIGQAPKGSADPFAL
RRAALGALRIIVEKNLPLDLNDIISKAFDLYKELDNERLRNAPIAKTRGGFSEYPEGYVS
FFTRGDDLVPKQKILDEVVDFMLGRFRAWYQDEGIAVDVIQAVLARRPTRPADFDARVRA
VSHFRTLDSAEALAAANKRVSNILAKAGAAIGEINLTACVEPAEKALAEAVLALRTEVQP
LIAQGDYTAVLDKLANLRAPVDSFFDNVMVNAEDPALRQNRLAILNTLQGLFLQVADISV
LQ
>P24150
MTAPALLCEVHGERDDGTGRPSLYLTFDDGPHPFCTPEILDILAEHRVPATFFVIGEFLA
DQSKLIQRMIAEGHHEVANHTMTHPDLSDCEPRRVQRQILETNRAIKMASPGGGAAHPRA
PYGIWTEEVLKASANAATHAVHWSVDPRDWSSPGADAIVNDVLQSVRPGAIVLLHDGCPP
DEMQQGADHSLRHQTIMALSSIIPALHDRPLCIYTLRLLGSSEDPMDIA
>P0A6V5
MDQFECINVADAHQKLQEKEAVLVDIRDPQSFAMGHAVQAFHLTNDTLGAFMRDNDFDTP
VMVMCYHGNSSKGAAQYLLQQGYDVVYSIDGGFEAWQRQFPAEVAYGA
>P23536
MPFALHGIPVSRGVAIGRAHLLAPAALDVSHYLVDEDQLDAEVERLRAARAAVRAELAAL
KRDLPRDAPEELGAFLDVHAMILDDEALAREPEALIRGRRYNAEWALTTRLEELMRQFDE

IEDEYLRERKTDIRQVVERILKALAGAPVLVPAPVPALAADGEAATGVIVVAHDIAPADM
LQFRHTVFHGFVTDMGGRTSHTAIVARSLDIPAAVGVQSASELIRQDDWIIIDGDAGLVI
VDPTAIILEEYRHRQSERALEKKRLQRLRHTPAVTLDGLEIDLLANIEMAEDAGAALAAG
AVGVGLFRSEFLFMNRRDELPGEDEQFQAYRGAVDAMHGLPVTIRTIDIGADKPLDARGD
EFETALNPALGLRAIRWSLSEPGMFLTQLRALLRASAFGPVRLLVPMLAHASEIDQTLAL
IAKAKRQLDERGEAYDPGMKVGAMIEIPAAVLLLPLFLRKMDFLSIGTNDLIQYTLAIDR
ADNAVAHLFDPLHPAVLQLVARTIREANRAGVPVAVCGEMAGDPSMTRLLLGMGLREFSM
HPAQLLRVKQEILHAHCERLEPLVDQVLQAFDPEEQAAALRQLARP
>Q7VFL0
MFSNFYVQQRIKKMELMRQEGFNPYANKTTRTISNYNFLNKYNHLKMQSSDDTQDCTQNK
EIESIVGRVRFIRLMGKACFIKIQDESGILQAYVSKNDIGEDFLLIKKVLEVGDIINVSG
YAFVTKTGELSIHTLTLQILTKSIVPLPEKFHGLNDIELRYRQRYVDLIVNNKVKETFKL
RSQIVSCIRQFFEQKGFLEVETPMLHSIPGGANARPFITHHNALDVDRYLRIAPELYLKR
LIVGGFEAIFELNRNFRNEGMDHSHNPEFSMIEFYWAYKTYEDLITLTQELFAFLFKKLN
LPHSLIHDELEIDFSQWHIIGYKEALIKIGGLDKNIIDNQDALLSFLMSKHLKVDKSMSY
GKLLGEAFDEFVEHKLINPTFITQYPIEISPLARRNDENPNVADRFELFIGGKEIANGFS
ELNDPIDQFERFKEQAKAKDAGDEEAQYMDEDYVWALAHGMPPTAGEGIGIDRLVMLLSN
AKTIKDVILFPALKPTKSNFDIILSQDALSNAQIIKEN
>P13039
MFEVTFWWRDPQGSEEYSTIKRVWVYITGVTDHHQNSQPQSMQRIAGTNVWQWTTQLNAN
WRGSYCFIPTERDDIFSVPSPDRLELREGWRKLLPQAIADPLNLQSWKGGRGHAVSALEM
PQAPLQPGWDCPQAPEIPAKEIIWKSERLKKSRRVWIFTTGDATAEERPLAVLLDGEFWA
QSMPVWPVLTSLTHRQQLPPAVYVLIDAIDTTHRAHELPCNADFWLAVQQELLPLVKAIA
PFSDRADRTVVAGQSFGGLSALYAGLHWPERFGCVLSQSGSYWWPHRGGQQEGVLLEKLK
AGEVSAEGLRIVLEAGIREPMIMRANQALYAQLHPIKESIFWRQVDGGHDALCWRGGLMQ
GLIDLWQPLFHDRS
>P69910
MDKKQVTDLRSELLDSRFGAKSISTIAESKRFPLHEMRDDVAFQIINDELYLDGNARQNL
ATFCQTWDDENVHKLMDLSINKNWIDKEEYPQSAAIDLRCVNMVADLWHAPAPKNGQAVG
TNTIGSSEACMLGGMAMKWRWRKRMEAAGKPTDKPNLVCGPVQICWHKFARYWDVELREI
PMRPGQLFMDPKRMIEACDENTIGVVPTFGVTYTGNYEFPQPLHDALDKFQADTGIDIDM
HIDAASGGFLAPFVAPDIVWDFRLPRVKSISASGHKFGLAPLGCGWVIWRDEEALPQELV
FNVDYLGGQIGTFAINFSRPAGQVIAQYYEFLRLGREGYTKVQNASYQVAAYLADEIAKL
GPYEFICTGRPDEGIPAVCFKLKDGEDPGYTLYDLSERLRLRGWQVPAFTLGGEATDIVV
MRIMCRRGFEMDFAELLLEDYKASLKYLSDHPKLQGIAQQNSFKHT
>P69783
MGLFDKLKSLVSDDKKDTGTIEIIAPLSGEIVNIEDVPDVVFAEKIVGDGIAIKPTGNKM
VAPVDGTIGKIFETNHAFSIESDSGVELFVHFGIDTVELKGEGFKRIAEEGQRVKVGDTV
IEFDLPLLEEKAKSTLTPVVISNMDEIKELIKLSGSVTVGETPVIRIKK
>P46547
MSSPLHYVLDGIHCEPHFFTVPLDHQQPDDEETITLFGRTLCRKDRLDDELPWLLYLQGG
PGFGAPRPSANGGWIKRALQEFRVLLLDQRGTGHSTPIHAELLAHLNPRQQADYLSHFRA
DSIVRDAELIREQLSPDHPWSLLGQSFGGFCSLTYLSLFPDSLHEVYLTGGVAPIGRSAD
EVYRATYQRVADKNRAFFARFPHAQAIANRLATHLQRHDVRLPNGQRLTVEQLQQQGLDL

GASGAFEELYYLLEDAFIGEKLNPAFLYQVQAMQPFNTNPVFAILHELIYCEGAASHWAA
ERVRGEFPALAWAQGKDFAFTGEMIFPWMFEQFRELIPLKEAAHLLAEKADWGPLYDPVQ
LARNKVPVACAVYAEDMYVEFDYSRETLKGLSNSRAWITNEYEHNGLRVDGEQILDRLIR
LNRDC
>P44420
MKQYLELCRRIVSEGEWVANERTGKHCLTVINADLEYDVANNQFPLITTRKSYWKAAIAE
FLGYIRGYDNAADFRALGTKTWDANANENAAWLANPHRRGVDDMGRVYGVQGRAWRKPNG
ETIDQLRKIVNNLTKGIDDRGEILTFFNPGEFDLGCLRPCMHTHTFSLVGDTLHLTSYQR
SCDVPLGLNFNQIQVFTFLALMAQITGKKAGKAYHKIVNAHIYEDQLELMRDVQLKREPF
PLPKLEINPDIKTLEDLETWVTMDDFKVVGYQSHEPIKYPFSV
>P11295
MKKSVDFIGVGTGPFNLSIAALSHQIEELDCLFFDEHPHFSWHPGMLVPDCHMQTVFLKD
LVSAVAPTNPYSFVNYLVKHKKFYRFLTSRLRTVSREEFSDYLRWAAEDMNNLYFSHTVE
NIDFDKKRRLFLVQTSQGEYFARNICLGTGKQPYLPPCVKHMTQSCFHASEMNLRRPDLS
GKRITVVGGGQSGADLFLNALRGEWGEAAEINWVSRRNNFNALDEAAFADEYFTPEYISG
FSGLEEDIRHQLLDEQKMTSDGITADSLLTIYRELYHRFEVLRKPRNIRLLPSRSVTTLE
SSGPGWKLLMEHHLDQGRESLESDVVIFATGYRSALPQILPSLMPLITMHDKNTFKVRDD
FTLEWSGPKENNIFVVNASMQTHGIAEPQLSLMAWRSARILNRVMGRDLFDLSMPPALIQ
WRSGT
>Q65V95
MRIIMATYTTSDFKPGLKFMQDGEPCVIVENEFVKPGKGQAFTRTRIRKLISGKVLDVNF
KSGTSVEAADVMDLNLNYSYKDEDFWYFMHPETFEQYSADSKAVGDAEKWLLDQAECIIT
LWNGSPISVTPPNFVELEVVDTDPGLKGDTAGTGGKPATLSTGAVVKVPLFIQIGEVIKV
DTRSGEYVSRVK
>Q1RIS8
MKFTLSWLKQFLDTSASVTEIAESLTAIGLEVEEIIDKAADLQKFEVAYIVSTKPHPSAD
KLKICEVETKNGNLQIVCGASNARAGIKVVLANIGVEIPNGKFKIKESNIRGEKSCGMLC
SEEELFLASESEGIIELPEDAVVGEPFTKYYGLDDPVFVINVTPNRGDALSVYGIARDLS
AKGIGTLKELEIPAVKSTFSSKVKLNIKDKEACPLFTFREIRNLKNKPSPDWLQKLLKNI
GVKPISSIVDVTNYMSYSFGQPMHAYDADKIGGGIVVDRHCEENVIPRLDCGISGEESVI
QDPVVKPRDDNRGFHALNDKKYLLNKSDLVIKDENGVQALAGIIGGISSSCDSNTMNILL
EAACFNAKMVAASGRRLQIDTDSRYRFERNIDRNFTEKALNIATDLILSICDGGEVSEIL
ISGEKEPAKKTLDFPAGYLEKITGIKLTILLHNEANKGEFVGNTEHSIAAYKEVREDAST
GLTPKLPLEASYVKGLNIKGIEAILNKLGFATDTEKDVIKITPPSWRHDINILEDVVEEI
TRIYGYDKIESIKLPELEQDNNRLREHKRISSFKRILASKGYDEVVTNSFMNSKDAKLFT
ELKDELFLLNPISVEDNYMRPTIVPNLLDIVRKNLARSIKDMAFFEVGPNFIGLNTEATY
LTAILTGSYNSKNPHSIGRSYDIFDLKSDLETVFDYAGLSIEKCIVSNQATPLYYHPTRS
VNLALGKNLLGHFGQIHPKILKHYDIKEEVFAFELNITNLPAPKAKFGKRDEFIISDYQA
NFRDYAFIIARDQPVGEIISYINNFNKKLVKSVILFDIYSGDKLPSGKKSIAIRVGLQAD
DRTLNEDDLNSFSKDLIANIEQKFQGTLRE
>P09373
MSELNEKLATAWEGFTKGDWQNEVNVRDFIQKNYTPYEGDESFLAGATEATTTLWDKVME
GVKLENRTHAPVDFDTAVASTITSHDAGYINKQLEKIVGLQTEAPLKRALIPFGGIKMIE
GSCKAYNRELDPMIKKIFTEYRKTHNQGVFDVYTPDILRCRKSGVLTGLPDAYGRGRIIG

DYRRVALYGIDYLMKDKLAQFTSLQADLENGVNLEQTIRLREEIAEQHRALGQMKEMAAK
YGYDISGPATNAQEAIQWTYFGYLAAVKSQNGAAMSFGRTSTFLDVYIERDLKAGKITEQ
EAQEMVDHLVMKLRMVRFLRTPEYDELFSGDPIWATESIGGMGLDGRTLVTKNSFRFLNT
LYTMGPSPEPNMTILWSEKLPLNFKKFAAKVSIDTSSLQYENDDLMRPDFNNDDYAIACC
VSPMIVGKQMQFFGARANLAKTMLYAINGGVDEKLKMQVGPKSEPIKGDVLNYDEVMERM
DHFMDWLAKQYITALNIIHYMHDKYSYEASLMALHDRDVIRTMACGIAGLSVAADSLSAI
KYAKVKPIRDEDGLAIDFEIEGEYPQFGNNDPRVDDLAVDLVERFMKKIQKLHTYRDAIP
TQSVLTITSNVVYGKKTGNTPDGRRAGAPFGPGANPMHGRDQKGAVASLTSVAKLPFAYA
KDGISYTFSIVPNALGKDDEVRKTNLAGLMDGYFHHEASIEGGQHLNVNVMNREMLLDAM
ENPEKYPQLTIRVSGYAVRFNSLTKEQQQDVITRTFTQSM
>P0A8P1
MRLVQLSRHSIAFPSPEGALREPNGLLALGGDLSPARLLMAYQRGIFPWFSPGDPILWWS
PDPRAVLWPESLHISRSMKRFHKRSPYRVTMNYAFGQVIEGCASDREEGTWITRGVVEAY
HRLHELGHAHSIEVWREDELVGGMYGVAQGTLFCGESMFSRMENASKTALLVFCEEFIGH
GGKLIDCQVLNDHTASLGACEIPRRDYLNYLNQMRLGRLPNNFWVPRCLFSPQE
>P84308
MTDTTNSIKHVISPLARQTLQDRDLTRPVAGKRPIRLLPWLQVVKIGGRVMDRGADAILP
LVEELRKLLPEHRLLILTGAGVRARHVFSVGLDLGLPVGSLAPLAASEAGQNGHILAAML
ASEGVSYVEHPTVADQLAIHLSATRAVVGSAFPPYHHHEFPGSRIPPHRADTGAFLLADA
FGAAGLTIVENVDGIYTADPNGPDRGQARFLPETSATDLAKSEGPLPVDRALLDVMATAR
HIERVQVVNGLVPGRLTAALRGEHVGTLIRTGVRPA
>Q2W3D5
MAATDTPWRPFRQAGALRRPMIDPAFVQGHPAYPDAFAQFFAGHGRYGHFAERVGNRNSR
NGWRRRALAPGPAVGAEIVARHHEAAAQAGQGGDAARHQHRVDRLGQHHVRAAFEQVGRH
FRLRGRSEHDRHRKGIVGADSPAHPPHQIARLLHAEIRVHHQKVDHFRPEVVFGIVAVVK
AQQIPASQHAKGAGDDITAAEVEIAQQGAHTWGVVGHGLPSWQERTTFWPPAFRSPSDRS
ATSEFVAGLVNIGLSRLDKIWPGMPSSRFGFKRAYEGFMHVYRSHTCGQLKAADAGIQAR
LSGWVHRKRDHGNLLFVDLRDHYGITQCVIDVSSPVFAALDKARPESVITVTGKVVKRSA
ETINPRLPTGEIELQVAEVEIQSIADVLPIQVAGDQEYPEDMRLRYRFLDLRREDVHANM
MLRSRVIAYLRQAMIGQGFTEFQTPILTASSPEGARDYLVPSRIHPGKFYALPQAPQQFK
QLLMVAGFDKYFQIAPCFRDEAGRADRSPGEFYQLDFEMSYVTQDDVFAAIEPVLEGVFK
EFGKGRAVTPAPFPRITYADSMLKYGSDKPDLRNPIIIADVTEPFRGSGFGLFAKLVDKG
AVVRAIPAPGAAGQPRSWFDKLNDWARENGAGGLGYIQFAADGPKGPIAKNLEPARVEAI
KAAANLKDGDAVFFACDKALPAAKFAGLVRTKIGNELDLLEKDVFKFCWTVDFPMYEINE
ETGLVEFSHNPFSMPQGGMDALLNQDPLTINAYQYDIVCNGVELSSGAIRNHRPDIMYKA
FEIAGYSAAHVEEHFGGMLNAFKFGAPPHGGSAPGVDRIVMLLADQPNIREIILFPMNQQ
AQDLLMQAPAEIAMERLRELHIKVDLPKPKKEVKEG
>P0A955
MKNWKTSAESILTTGPVVPVIVVKKLEHAVPMAKALVAGGVRVLEVTLRTECAVDAIRAI
AKEVPEAIVGAGTVLNPQQLAEVTEAGAQFAISPGLTEPLLKAATEGTIPLIPGISTVSE
LMLGMDYGLKEFKFFPAEANGGVKALQAIAGPFSQVRFCPTGGISPANYRDYLALKSVLC
IGGSWLVPADALEAGDYDRITKLAREAVEGAKL
>P0A8F4
MTDQSHQCVIIGIAGASASGKSLIASTLYRELREQVGDEHIGVIPEDCYYKDQSHLSMEE

RVKTNYDHPSAMDHSLLLEHLQALKRGSAIDLPVYSYVEHTRMKETVTVEPKKVIILEGI
LLLTDARLRDELNFSIFVDTPLDICLMRRIKRDVNERGRSMDSVMAQYQKTVRPMFLQFI
EPSKQYADIIVPRGGKNRIAIDILKAKISQFFE
>P33694
MLQILYLAQDLADPAVRRRTLTLVAGGARVTLAGFRRGDNPLAAIDGVEPIELGTTADGR
FAQRIGAVARACLSLQRQLGHVRKPDVIIARNLEMLAVARRAVAFFGGTVPIVYECLDIH
RLMLRKDIVGRMLRAAESQLGKDARLLITSSPAFIEHYFRPLSGIGAPPMLLENKVLEID
GTVERRTASPAKSPPPGAPWKIGWFGALRCRRSLALLAEFSRKMEGRFEIVLRGRPAYSE
FDDFDGFVRNEPFMRFEGAYRNPEDLAEIYGEVHFTWAIDFFEEGQNSAWLLPNRLYEGC
RHGRIPIAMKGTETARFLSVRSIGLVLEGADVESLATVLGPLTPNCYADAAERISRCNPG
SWVFDRTDCEALVRQLATLTLQAPQTVPVVAMAGSSHKEGGFL
>Q8XYN6
MAHFSCFPGALALSAFRQQRLLSTLKRIDPEIDAVSAQYLHFVAADAPLSADDAARVQAL
LTYGSPASAETEGDRFVVIPRFGTISPWASKATEIARHCALPQIHRIERGVEFTVTCKKG
LLRGLTGGRKQLDEATRAAVAAHLHDRMTEIVVATREAGYGLFDVLPAKALRFVDLGSGD
AAAGRGALEAANTEMGLALSDDEIDYLVDAYRKLGRNPTDVELMMFAQANSEHCRHKIFN
ADWTIDGETQDKSLFAMIRNTHQLAPQGTVVAYSDNAAVMEGGMAERWFPHAGTDGETGV
PQYGRREALTHTLMKVETHNHPTAISPFPGASTGAGGEIRDEGATGRGAKPKAGLTGFTV
SNLLLPEAVQSWENARDTAQPAAQRNPGDTAPGPVGKPDRIASPLQIMIEGPIGGAAFNN
EFGRPNLGGYFRVYEQNVGGTVRGYHKPIMIAGGIGNIDAGHTHKHGLPAGTLLVQLGGP
GMRIGMGGGAASSMATGTNTADLDFDSVQRGNPEMQRRAQEVINACWALGEDNPILSIHD
VGAGGISNAFPELVDGADRGARFDLRQVHLEESGLSPAEIWCNESQERYTLAIAPGDFPR
FQAMCERERAPFSVVGFATEEQQLQVVDGDAPADAVEHFPVNMPMDVLLGKPPRMHRDVR
RVAQALPEVDVTGLDLETVARDVLRHPTVASKSFLITIGDRTVGGLNTRDQMVGPWQVPV
ADVAVTTLDYKGYAGEAMTMGERTPLAVIDAPASGRMAIGEAITNIAAAPIASLAQLKLS
ANWMAACGVDGEDARLYDTVKAVGMELCPALGISIPVGKDSLSMRTKWDDAGEAKEVVAP
VSLIVSAFAPVTDVRKTLTPQLKPVASAGEAADTTLIVIDLGHGKHRLGGSILAQVTQQI
GNSVPDVGDAEDLKRFFAAIQQLNAAGMLLAYHDRSDGGLWATVCEMAFAGHCGVSINVD
MLTLDGAHASDYGDAKNWAQQVSGRRADMTLRALFAEELGAVIQVPAAQRDAVFAVLREH
GLAACSHVIGAPNASGQIEIWRDAKKVFSAPRIELQRAWTDVSWRIASLRDNPECTQSEY
DRLLDAEDPGISPNLTFDLAEDVAAPFIATGARPRMAILREQGVNSQVEMAYAMDKAGFD
AYDVHMSDLLAGRTRLADFKGFVACGGFSYGDVLGAGEGWAKTILFNGMLAEQFAAFFNR
ADSIALGVCNGCQMMANLAPIIPGAGAWPKFTRNQSEQYEGRLVTVQVEASPSIFYAGME
GSRIPIVVAHGEGYADFSQQGDIGKVAVGLRYVDNRGEVTQTYPLNPNGSPQGIASVTTH
DGRFTVLMPHPERVFRAVQMSWHPKDWAAAGDGSSPWMRMFRNARKQMG
>P18330
MTDYKATLNLPDTAFPMKAGLPQREPQILQRWNSIGLYGKLREIGKDRPKFVLHDGPPYA
NGTIHIGHALNKILKDMILRSKTLSGFDAPYVPGWDCHGLPIEHKVEVTYGKNLGADKTR
ELCRAYATEQIEGQKSEFIRLGVLGEWDNPYKTMNFKNEAGEIRALAEIVKGGFVFKGLK
PVNWCFDCGSALAEAEVEYEDKKSSTIDVAFPIADDAKLAEAFGLASLAKPAAIVIWTTT
PWTIPANQALNVHPEFTYALVDVGDRLLVLAEEMVESCLARYELQGSVIATATGSALELI
NFRHPFYDRLSPVYLADYVELGSGTGIVHCSPAYGVDDFVICKKYGMVNDDIINPVQSNG
VYVPSLEFFGGQFIFKADQPIIEKLREVGALMQTAAIQHSYMHCWRHKTPLIYRATAQWF
IGMDKEPTSGDTLRVRSLKAIEDTKFVPSWGQARLHSMIANRPDWCISRQRNWGVPIPFF

LNKESGELHPRTVELMEVVAQRVEQQGIEAWFKLDAAELLGDEAPLYDKISDTLDVWFDS
GTTHWHVLRGSHPMGHETGPRADLYLEGSDQHRGWFHSSLLTGCAIDNHAPYRELLTHGF
TVDETGRKMSKSLKNVIEPKKINDTLGADIMRLWVASTDYSGEIAVSDQILARSADAYRR
IRNTARFMLSNLTGFNPASDLLPAEDMLALDRWAVDRTLLLQRELQEHYGEYRFWNVYSK
IHNFCVQELGGFYLDIIKDRQYTTGANSKARRSAQTALYHISEALVRWIAPILAFTADEL
WEYLPGERNESVMLNTWYEGLTELPADFELGREYWEGVMAVKVAVNKELEVQRAAKAVGG
NLQAEVTLFAEDGLTADLAKLSNELRFVLITSTASLAPFTQAPADAVATEVPGLKLKVVK
SAFPKCARCWHCREDVGVNPEHPEICGRCVDNISGEGEVRHYA
>P0ABD5
MSLNFLDFEQPIAELEAKIDSLTAVSRQDEKLDINIDEEVHRLREKSVELTRKIFADLGA
WQIAQLARHPQRPYTLDYVRLAFDEFDELAGDRAYADDKAIVGGIARLDGRPVMIIGHQK
GRETKEKIRRNFGMPAPEGYRKALRLMQMAERFKMPIITFIDTPGAYPGVGAEERGQSEA
IARNLREMSRLGVPVVCTVIGEGGSGGALAIGVGDKVNMLQYSTYSVISPEGCASILWKS
ADKAPLAAEAMGIIAPRLKELKLIDSIIPEPLGGAHRNPEAMAASLKAQLLADLADLDVL
STEDLKNRRYQRLMSYGYA
>Q165J6
MRADVQNTIEKIQKSLDLLAQRLDVETAPYRLEEFNARVEDPTLWDDPDAAQKLMRERQM
LVDAMATHDSIKQEMADNIELIELGEMEDDADVVSDAENALRALEETAAKKELEALLDGE
ADSNDTFLEVNAGAGGTESCDWASMLARMYVRWAEKKGYKVELQSMSDGDEAGIKSATYK
ITGLNAYGWLKSESGVHRLVRISPFDSAAKRHTSFSSVWVYPVVDDNIDIEVNPADIRID
TYRSSGAGGQHVNTTDSAVRITHHPTGIVVTSSEKSQHQNRDIAMKALKSRLYQLELDRR
NAAINEAHENKGDAGWGNQIRSYVLQPYQMVKDLRTNFETSDTKGVLDGDLDGFMAATLA
LDASGKTRAEAQNG
>P13266
MRTKIIAIIPARSGSKGLRNKNALMLIDKPLLAYTIEAALQSEMFEKVIVTTDSEQYGAI
AESYGADFLLRPEELATDKASSFEFIKHALSIYTDYESFALLQPTSPFRDSTHIIEAVKL
YQTLEKYQCVVSVTRSNKPSQIIRPLDDYSTLSFFDLDYSKYNRNSIVEYHPNGAIFIAN
KQHYLHTKHFFGRYSLAYIMDKESSLDIDDRMDFELAITIQQKKNRQKIDLYQNIHNRIN
EKRNEFDSVSDITLIGHSLFDYWDVKKINDIEVNNLGIAGINSKEYYEYIIEKELIVNFG
EFVFIFFGTNDIVVSDWKKEDTLWYLKKTCQYIKKKNAASKIYLLSVPPVFGRIDRDNRI
INDLNSYLRENVDFAKFISLDHVLKDSYGNLNKMYTYDGLHFNSNGYTVLENEIAEIVK
>Q9JWJ3
MTTITEKRLTFAFPEDYYVTKYDEWEHYKIFQNSCNLRNKIDTNEKGKNGINQSVDDDNG
SSGVDIIALHESTLWLIEIKDYYRLGLEPNAQSIDEKLSDLPYLIARKIRDSLAGLVSAK
FKAEKQEEKDFSRLALDCNEIKIVLHIEMPSIRSKLYPSSSDLANLLKDKFKLSEFTKNF
ANCYAEPIFTNISHINNPQLRNVPWSVSTGTEQKLSSEQQRLIHNPMTTIYNTLTRQKEP
FAPIDPKNVRMYVCGMTVYDYCHLGHARVMVVFDMIARWLRECGYPLTYVRNITDIDDKI
IARAAENGETIGELTARFIQAMHEDADALGVLRPDIEPKATENIPQMIAMIETLIQNGKA
YPAANGDVYYAVREFSAYGQLSGKSLDDLRAGERVEVDGFKRDPLDFVLWKAAKAGEPAW
ESPWGNGRPGWHIECSAMSENLFGNTFDIHGGGADLQFPHHENEIAQSVGATGHTCGHHH
AQTHHGQSIASHVKYWLHNGFIRVDGEKMSKSLGNFFTIREVLKQYDPEVVRFFILRAHY
RSPLNYSDAHLDDAKGALTRLYTTLKNTPAAEFDLSENVNDYTRRFYAAMNDDFGTVEAV
AVLFELAGEVNKTNDAQLAGCLKALGGIIGLLQRDPTEFLQGGAASDGLSNEEIEDLIAR
RKQARADKNWAESDRIRDLLNEHKIILEDNAGGTTWRRG

>P21762
MLVTKLAPDFKAPAVLGNNEVDEHFELSKNLGKNGAILFFWPKDFTFVCPTEIIAFDKRV
KDFQEKGFNVIGVSIDSEQVHFAWKNTPVEKGGIGQVTFPMVADITKSISRDYDVLFEEA
IALRGAFLIDKNMKVRHAVINDLPLGRNADEMLRMVDALLHFEEHGEVCPAGWRKGDKGM
KATHQGVAEYLKENSIKL
>P0A2J9
MKIIILGAGQVGGTLAENLVGENNDITVVDTNGERLRSLQDKFDLRVVQGHGSHPRVLRE
AGADDADMLVAVTSSDETNMVACQVAYSLFNTPNRIARIRSPDYVRDADKLFHSEAVPID
HLIAPEQLVIDNIYRLIEYPGALQVVNFAEGKVSLAVVKAYYGGPLIGNALSTMREHMPH
IDTRVAAIFRHDRPIRPQGSTIVEAGDEVFFIAASQHIRAVMSELQRLEKPYKRIMLVGG
GNIGAGLARRLEKDYSVKLIERDQQRAAELAEKLQNTIVFFGDASDQELLAEEHIDQVDL
FIAVTNDDEANIMSAMLAKRMGAKKVMVLIQRRAYVDLVQGSVIDIAISPQQATISALLS
HVRKADIVGVSSLRRGVAEAIEAVAHGDESTSRVVGRVIDEIKLPPGTIIGAVVRGNDVM
IANDNLRIEQGDHVIMFLTDKKFITDVERLFQPSPFFL
>P36767
MLWFKNLMVYRLSREISLRAEEMEKQLASMAFTPCGSQDMAKMGWVPPMGSHSDALTHVA
NGQIVICARKEEKILPSPVIKQALEAKIAKLEAEQARKLKKTEKDSLKDEVLHSLLPRAF
SRFSQTMMWIDTVNGLIMVDCASAKKAEDTLALLRKSLGSLPVVPLSMENPIELTLTEWV
RSGSAAQGFQLLDEAELKSLLEDGGVIRAKKQDLTSEEITNHIEAGKVVTKLALDWQQRI
QFVMCDDGSLKRLKFCDELRDQNEDIDREDFAQRFDADFILMTGELAALIQNLIEGLGGE
AQR
>P17854
MSKLDLNALNELPKVDRILALAETNAELEKLDAEGRVAWALDNLPGEYVLSSSFGIQAAV
SLHLVNQIRPDIPVILTDTGYLFPETYRFIDELTDKLKLNLKVYRATESAAWQEARYGKL
WEQGVEGIEKYNDINKVEPMNRALKELNAQTWFAGLRREQSGSRANLPVLAIQRGVFKVL
PIIDWDNRTIYQYLQKHGLKYHPLWDEGYLSVGDTHTTRKWEPGMAEEETRFFGLKRECG
LHEG
>P0A9G6
MKTRTQQIEELQKEWTQPRWEGITRPYSAEDVVKLRGSVNPECTLAQLGAAKMWRLLHGE
SKKGYINSLGALTGGQALQQAKAGIEAVYLSGWQVAADANLAASMYPDQSLYPANSVPAV
VERINNTFRRADQIQWSAGIEPGDPRYVDYFLPIVADAEAGFGGVLNAFELMKAMIEAGA
AAVHFEDQLASVKKCGHMGGKVLVPTQEAIQKLVAARLAADVTGVPTLLVARTDADAADL
ITSDCDPYDSEFITGERTSEGFFRTHAGIEQAISRGLAYAPYADLVWCETSTPDLELARR
FAQAIHAKYPGKLLAYNCSPSFNWQKNLDDKTIASFQQQLSDMGYKFQFITLAGIHSMWF
NMFDLANAYAQGEGMKHYVEKVQQPEFAAAKDGYTFVSHQQEVGTGYFDKVTTIIQGGTS
SVTALTGSTEESQF
>O87712
MAEIIGIDLGTTNSCVAVMEGGKVRVIENAEGSRTTPSIVAYTKDGEVLVGASAKRQAVT
NADRTLYAIKRLIGRRFDDNVVQKDIKMVPYKIIKADNGDAWVEVKDKEGKSQKLAPPQI
SAQVLIKMKKTAEDYLGHEVKDAVITVPAYFNDSQRQATKDAGKIAGLNVKRIINEPTAA
ALAYGMDKKKGDRKIAVYDLGGGTFDISIIEIAEVDGEHQFEVLATNGDTFLGGEDFDLR
LIDYLAGEFKKDEGVDLHNDPLALQRLKEAAEKAKIELSSSQQTDVNLPYITADASGPKH
LNIRLTRAKLESLVEDLVERTIEPCKVAIKDAGLKVSEIDDVILVGGQTRMPKVQEAVKN
FFGKEARKDVNPDEAVAIGAAIQGAVLSGEVKDVLLLDVTPLSLGIETLGGVMTKLIEKN

TTIPTKANQVFSTADDNQTAVTVHVLQGEREMASANKSLGRFDLSDIPPAPRGVPQIEVT
FDIDANGILHVSAKDKATGKEQSIVIKASSGLSDEEVEKMVKDAEAHRDSDRKFHELVDA
RNQADAMIHAAEKSVKDLGSEVSADEKSAIEKAVNELKEAMKGNDKDAIEAKTKALTEHS
SKLAERVYAKKGGAAGAPPGGEAEGEPQAQAGGKKEDVVDAEFEEVKDEKKKDEDK
>P09384
MSMDISDFYQTFFDEADELLADMEQHLLDLVPESPDAEQLNAIFRAAHSIKGGAGTFGFT
ILQETTHLMENLLDEARRGEMQLNTDIINLFLETKDIMQEQLDAYKNSEEPDAASFEYIC
NALRQLALEAKGETTPAVVETAALSAAIQEESVAETESPRDESKLRIVLSRLKANEVDLL
EEELGNLATLTDVVKGADSLSATLDGSVAEDDIVAVLCFVIEADQIAFEKVVAAPVEKAQ
EKTEVAPVAPPAVVAPAAKSAAHEHHAGREKPARERESTSIRVAVEKVDQLINLVGELVI
TQSMLAQRSNELDPVNHGDLITSMGQLQRNARDLQESVMSIRMMPMEYVFSRFPRLVRDL
AGKLGKQVELTLVGSSTELDKSLIERIIDPLTHLVRNSLDHGIEMPEKRLEAGKNVVGNL
ILSAEHQGGNICIEVTDDGAGLNRERILAKAMSQGMAVNENMTDDEVGMLIFAPGFSTAE
QVTDVSGRGVGMDVVKRNIQEMGGHVEIQSKQGSGTTIRILLPLTLAILDGMSVRVAGEV
FILPLNAVMESLQPREEDLHPLAGGERVLEVRGEYLPLVELWKVFDVDGAKTEATQGIVV
ILQSAGRRYALLVDQLIGQHQVVVKNLESNYRKVPGISAATILGDGSVALIVDVSALQGL
NREQRMAITAA
>P44694
MTISKFNPQKPFECFIVQSEAMKSAVENAKRFAMFDAPLLIQGETGSGKDLLAKACHYQS
LRRDKKFIAVNCAGLPDEDAESEMFGRKVGDSETIGFFEYANKGTVLLDGIAELSLSLQA
KLLRFLTDGSFRRVGEEKEHYANVRVICTSQVPLHLLVEQGKVRADLFHRLNVLTINVPA
LRDRMADIEPLAQGFLQEISEELKIAKPTFDKDFLLYLQKYDWKGNVRELYNTLYRACSL
VQDNHLTIESLNLALPQSAVISLDEFENKTLDEIIGFYEAQVLKLFYAEYPSTRKLAQRL
GVSHTAIANKLKQYGIGK
>P0A8U0
MDDLTAQALKDFTARYCDAWHEEHKSWPLSEELYGVPSPCIISTTEDAVYWQPQPFTGEQ
NVNAVERAFDIVIQPTIHTFYTTQFAGDMHAQFGDIKLTLLQTWSEDDFRRVQENLIGHL
VTQKRLKLPPTLFIATLEEELEVISVCNLSGEVCKETLGTRKRTHLASNLAEFLNQLKPL
L
>P69996
MKKISRKEYVSMYGPTTGDKVRLGDTDLIAEVEHDYTIYGEELKFGGGKTLREGMSQSNN
PSKEELDLIITNALIVDYTGIYKADIGIKDGKIAGIGKGGNKDMQDGVKNNLSVGPATEA
LAGEGLIVTAGGIDTHIHFISPQQIPTAFASGVTTMIGGGTGPADGTNATTITPGRRNLK
WMLRAAEEYSMNLGFLAKGNASNDASLADQIEAGAIGFKIHEDWGTTPSAINHALDVADK
YDVQVAIHTDTLNEAGCVEDTMAAIAGRTMHTFHTEGAGGGHAPDIIKVAGEHNILPAST
NPTIPFTVNTEAEHMDMLMVCHHLDKSIKEDVQFADSRIRPQTIAAEDTLHDMGIFSITS
SDSQAMGRVGEVITRTWQTADKNKKEFGRLKEEKGDNDNFRIKRYLSKYTINPAIAHGIS
EYVGSVEVGKVADLVLWSPAFFGVKPNMIIKGGFIALSQMGDANASIPTPQPVYYREMFA
HHGKAKYDANITFVSQAAYDKGIKEELGLERQVLPVKNCRNITKKDMQFNDTTAHIEVNP
ETYHVFVDGKEVTSKPANKVSLAQLFSIF
>P45363
MSDTIVIVDAGRTAIGTFGGALSALQATDIGTTVLKALIERTGIAPEQVSEVILGQVLTA
GCGQNPARQTTLMAGLPHTVPAMTINKVCGSGLKAVHLAMQAVACGDAEIVIAGGQESMS
QSSHVLPRSREGQRMGDWPMKDTMIVDGLWDAFNQCHMGVTAENIAKKYAFTREAQDAFA

AASQQKAEAAIQSGRFADEIIPVSIPQRKGDPLVFDTDEFPRPGTTAETLGRLRPAFDKQ
GTVTAGNASGINDGAAMVVVMKESKAKELGLTPMARLVAFSSAGVDPAIMGTGPIPASTD
CLKKAGWAPADLDLVEANEAFAAQAMSVNQEMGWDLSKVNVNGGAIAIGHPIGASGARVL
VTLLYEMQKRDAKKGLATLCIGGGQGVALAVERL
>P0A2U4
MSLNITENESISTAVIDAINSGATLKDINAIPDDMMDDIYSYAYDFYNKGRIEEAEVFFR
FLCIYDFYNVDYIMGLAAIYQIKEQFQQAADLYAVAFALGKNDYTPVFHTGQCQLRLKAP
LKAKECFELVIQHSNDEKLKIKAQSYLDAIQDIKE
>Q89UH1
MAKEELIQFEGLVTEILPDARYRVQLDAGHEIVAYTAGKMKKNRIKTLAGDRVTVEMSPY
DLEKGRLIFRHKDERPATSGGPPRGGAQRGGQFRRR
>Q84H41
MAATDNRKVVEGVHKMTPSEAFVETCVANGVSEMFGIMGSAFMDAMDIFAPAGIRLIPVV
HEQGAAHMADGYARVSGRHGVVIGQNGPGISNCVTGIAAAYWAHSPVVIVTPETGTMGMG
LGGFQEANQLPMFQEFTKYQGHVCNPKRMAEFTGRVFDRAMSEMGPTQLNIPRDYFYGEI
ECEIPKPMRVDRGHGGEASLQAAVELLKTAKFPVILAGGGVVMGDAVEEAKQLAERLGAP
VATGYLRNDAFPAKHPLWAGPLGYQGSKAAMKLIAQADVVIALGSRMGPFGTLPQHGMDY
WPKAAKIIQIEADHTNLGLVKKIAVGINGDAKAVAAELSRRLADVTLGCDATKAARADTI
ATEKAAWEKELDGWTHERDPYSLDMIEEAKGERTPTGGSYLHPRQVLRELEKAMPARVMV
STDIGNINSVANSYLRFDEPRSFFAPMSFGNCGYALPTIIGAKCAAPDRPAIAYAGDGAW
GMSMMEIMTAVRHDIPVTAVVFHNRQWGAEKKNQVDFYNRRFVAGELESESFSDIAKAMG
AEGIVVDHIEDVGPALQKAIDMQMKEGKTCVIEIMCTRELGDPFRRDALSKPVRMLDKYK
DYV
>P0A7B5
MSDSQTLVVKLGTSVLTGGSRRLNRAHIVELVRQCAQLHAAGHRIVIVTSGAIAAGREHL
GYPELPATIASKQLLAAVGQSRLIQLWEQLFSIYGIHVGQMLLTRADMEDRERFLNARDT
LRALLDNNIVPVINENDAVATAEIKVGDNDNLSALAAILAGADKLLLLTDQKGLYTADPR
SNPQAELIKDVYGIDDALRAIAGDSVSGLGTGGMSTKLQAADVACRAGIDTIIAAGSKPG
VIGDVMEGISVGTLFHAQATPLENRKRWIFGAPPAGEITVDEGATAAILERGSSLLPKGI
KSVTGNFSRGEVIRICNLEGRDIAHGVSRYNSDALRRIAGHHSQEIDAILGYEYGPVAVH
RDDMITR
>P13033
MRFDTVIMGGGLAGLLCGLQLQKHGLRCAIVTRGQSALHFSSGSLDLLSHLPDGQPVTDI
HSGLESLRQQAPAHPYSLLEPQRVLDLACQAQALIAESGAQLQGSVELAHQRVTPLGTLR
STWLSSPEVPVWPLPAKKICVVGISGLMDFQAHLAAASLRELGLAVETAEIELPELDVLR
NNATEFRAVNIARFLDNEENWPLLLDALIPVANTCEMILMPACFGLADDKLWRWLNEKLP
CSLMLLPTLPPSVLGIRLQNQLQRQFVRQGGVWMPGDEVKKVTCKNGVVNEIWTRNHADI
PLRPRFAVLASGSFFSGGLVAERNGIREPILGLDVLQTATRGEWYKGDFFAPQPWQQFGV
TTDETLRPSQAGQTIENLFAIGSVLGGFDPIAQGCGGGVCAVSALHAAQQIAQRAGGQQ
>P0A763
MAIERTFSIIKPNAVAKNVIGNIFARFEAAGFKIVGTKMLHLTVEQARGFYAEHDGKPFF
DGLVEFMTSGPIVVSVLEGENAVQRHRDLLGATNPANALAGTLRADYADSLTENGTHGSD
SVESAAREIAYFFGEGEVCPRTR
>Q05097

MAWKGEVLANNEAGQVTSIIYNPGDVITIVAAGWASYGPTQKWGPQGDREHPDQGLICHD
AFCGALVMKIGNSGTIPVNTGLFRWVAPNNVQGAITLIYNDVPGTYGNNSGSFSVNIGKD
QS
>P0A996
MNDTSFENCIKCTVCTTACPVSRVNPGYPGPKQAGPDGERLRLKDGALYDEALKYCINCK
RCEVACPSDVKIGDIIQRARAKYDTTRPSLRNFVLSHTDLMGSVSTPFAPIVNTATSLKP
VRQLLDAALKIDHRRTLPKYSFGTFRRWYRSVAAQQAQYKDQVAFFHGCFVNYNHPQLGK
DLIKVLNAMGTGVQLLSKEKCCGVPLIANGFTDKARKQAITNVESIREAVGVKGIPVIAT
SSTCTFALRDEYPEVLNVDNKGLRDHIELATRWLWRKLDEGKTLPLKPLPLKVVYHTPCH
MEKMGWTLYTLELLRNIPGLELTVLDSQCCGIAGTYGFKKENYPTSQAIGAPLFRQIEES
GADLVVTDCETCKWQIEMSTSLRCEHPITLLAQALA
>P00499
MLDNTRLRIAIQKSGRLSDDSRELLARCGIKINLHTQRLIAMAENMPIDILRVRDDDIPG
LVMDGVVDLGIIGENVLEEELLNRRAQGEDPRYLTLRRLDFGGCRLSLATPVDEAWDGPA
ALDGKRIATSYPHLLKRYLDQKGVSFKSCLLNGSVEVAPRAGLADAICDLVSTGATLEAN
GLREVEVIYRSKACLIQRDGEMAQSKQELIDKLLTRIQGVIQARESKYIMMHAPSERLEE
VIALLPGAERPTILPLAGEQQRVAMHMVSSETLFWETMEKLKALGASSILVLPIEKMME
>P17169
MCGIVGAIAQRDVAEILLEGLRRLEYRGYDSAGLAVVDAEGHMTRLRRLGKVQMLAQAAE
EHPLHGGTGIAHTRWATHGEPSEVNAHPHVSEHIVVVHNGIIENHEPLREELKARGYTFV
SETDTEVIAHLVNWELKQGGTLREAVLRAIPQLRGAYGTVIMDSRHPDTLLAARSGSPLV
IGLGMGENFIASDQLALLPVTRRFIFLEEGDIAEITRRSVNIFDKTGAEVKRQDIESNLQ
YDAGDKGIYRHYMQKEIYEQPNAIKNTLTGRISHGQVDLSELGPNADELLSKVEHIQILA
CGTSYNSGMVSRYWFESLAGIPCDVEIASEFRYRKSAVRRNSLMITLSQSGETADTLAGL
RLSKELGYLGSLAICNVPGSSLVRESDLALMTNAGTEIGVASTKAFTTQLTVLLMLVAKL
SRLKGLDASIEHDIVHGLQALPSRIEQMLSQDKRIEALAEDFSDKHHALFLGRGDQYPIA
LEGALKLKEISYIHAEAYAAGELKHGPLALIDADMPVIVVAPNNELLEKLKSNIEEVRAR
GGQLYVFADQDAGFVSSDNMHIIEMPHVEEVIAPIFYTVPLQLLAYHVALIKGTDVDQPR
NLAKSVTVE
>Q01551
MTKYNEAYCDVLIVGAGPAGVMAAAHLLSYGTTARPHRVRIFDATKEVNGSDESTESLST
DVIADALNSGASGPEKDAASTTEDLPMLVTTLQVSDVLHDTGDDTKIAYRETATEQQVLL
LADTTANTSSTMNPRSMCEAGCRFHQIYQGHCFPEYELDSERLRSVDGRAQVLEDEHETG
QLRLERLGRPEELLELDEENSMSVVTNLKAAPYKFLMKDVDENFPGELSTSGGKTTSISA
DESAIDAALHAVWDADDLGAAWHLDEASGLRAVDWNAAQWFKSGQPWTPDAAKSLQEGRV
FLAGDARHRHPPLTGIGKNTSIADCYNLTWKLLGVLLGVARADPARTYVAERVYIRMRAA
TDIAVDAEMESLAAKWITVQLTLSRSWISSAKEAERWDAVLRDSAMSASKPMWTTSDMRA
SFDAGLMGHGHAHDHVTPTIKEFASSSISRSISELASTSWWESRGWGNGGPFESLMEDAR
WTGAVESNCRYAAYDRDAPVLHEHVAWVTRFTSRARTAVLEAAVGQAHVVDCWDVGLVEP
ALDDLDSAGAGLHVAHHADQWPAQLDEAVWPRESLSDWRIVTDTSATGEGYQTSPREAPG
DYADLNADNAKAHFNGQFAGHKAYGDAAAADGGGCHGRILVGPAVRGRHLHREIPLGEEC
QRAAQPLFKEV
>P07004
MLEQMGIAAKQASYKLAQLSSREKNRVLEKIADELEAQSEIILNANAQDVADARANGLSE

AMLDRLALTPARLKGIADDVRQVCNLADPVGQVIDGGVLDSGLRLERRRVPLGVIGVIYE
ARPNVTVDVASLCLKTGNAVILRGGKETCRTNAATVAVIQDALKSCGLPAGAVQAIDNPD
RALVSEMLRMDKYIDMLIPRGGAGLHKLCREQSTIPVITGGIGVCHIYVDESVEIAEALK
VIVNAKTQRPSTCNTVETLLVNKNIADSFLPALSKQMAESGVTLHADAAALAQLQAGPAK
VVAVKAEEYDDEFLSLDLNVKIVSDLDDAIAHIREHGTQHSDAILTRDMRNAQRFVNEVD
SSAVYVNASTRFTDGGQFGLGAEVAVSTQKLHARGPMGLEALTTYKWIGIGDYTIRA
>P31858
MKNVGIKKAALQMSRYVLASKQVQRALKLLGLPASRANEVSGIPKPADTSLSFPGYEAFA
LHGEAWQAAGEQKPIILMFGVHPWKRDVLARYFSDFRVAYVRTNTSWTKVQTSFCQFTPQ
AFVFWGMTEIRAAKNYAIKSSIPLWRVEDGFLRSVGLGAQHVLPLSLAVDTTGIYFDPSR
PSTLETLISEIGVTENATLIERARRCMSMISAFGLSKYNVGQDVPLKRLPPSDRRRVLVV
GQVEDDASIVMGCAARYTNNDIVRITQKENPEAEVIYRPHPDVLGGHRKEFSNPRDVANI
CTILSGDYDLGSLLDSVDHVYTITSLLGFEALIRRKKVTVFGAPFYSGWGLTDDRQPTPR
RTRKPSLDELFAAAYILYPRYCVGSLGSSAEIEHAIMSLALEKNGVPRELAEGVSSALPA
EAINVDCVSQTLEGLKSIPS
>P25745
MSETAKKVIVGMSGGVDSSVSAWLLQQQGYQVEGLFMKNWEEDDGEEYCTAAADLADAQA
VCDKLGIELHTVNFAAEYWDNVFELFLAEYKAGRTPNPDILCNKEIKFKAFLEFAAEDLG
ADYIATGHYVRRADVDGKSRLLRGLDSNKDQSYFLYTLSHEQIAQSLFPVGELEKPQVRK
IAEDLGLVTAKKKDSTGICFIGERKFREFLGRYLPAQPGKIITVDGDEIGEHQGLMYHTL
GQRKGLGIGGTKEGTEEPWYVVDKDVENNILVVAQGHEHPRLMSVGLIAQQLHWVDREPF
TGTMRCTVKTRYRQTDIPCTVKALDDDRIEVIFDEPVAAVTPGQSAVFYNGEVCLGGGII
EQRLPLPV
>P07118
MEKTYNPQDIEQPLYEHWEKQGYFKPNGDESQESFCIMIPPPNVTGSLHMGHAFQQTIMD
TMIRYQRMQGKNTLWQVGTDHAGIATQMVVERKIAAEEGKTRHDYGREAFIDKIWEWKAE
SGGTITRQMRRLGNSVDWERERFTMDEGLSNAVKEVFVRLYKEDLIYRGKRLVNWDPKLR
TAISDLEVENRESKGSMWHIRYPLADGAKTADGKDYLVVATTRPETLLGDTGVAVNPEDP
RYKDLIGKYVILPLVNRRIPIVGDEHADMEKGTGCVKITPAHDFNDYEVGKRHALPMINI
LTFDGDIRESAQVFDTKGNESDVYSSEIPAEFQKLERFAARKAVVAAVDALGLLEEIKPH
DLTVPYGDRGGVVIEPMLTDQWYVRADVLAKPAVEAVENGDIQFVPKQYENMYFSWMRDI
QDWCISRQLWWGHRIPAWYDEAGNVYVGRNEDEVRKENNLGADVVLRQDEDVLDTWFSSA
LWTFSTLGWPENTDALRQFHPTSVMVSGFDIIFFWIARMIMMTMHFIKDENGKPQVPFHT
VYMTGLIRDDEGQKMSKSKGNVIDPLDMVDGISLPELLEKRTGNMMQPQLADKIRKRTEK
QFPNGIEPHGTDALRFTLAALASTGRDINWDMKRLEGYRNFCNKLWNASRFVLMNTEGQD
CGFNGGEMTLSLADRWILAEFNQTIKAYREALDSFRFDIAAGILYEFTWNQFCDWYLELT
KPVMNGGTEAELRGTRHTLVTVLEGLLRLAHPIIPFITETIWQRVKVLCGITADTIMLQP
FPQYDASQVDEAALADTEWLKQAIVAVRNIRAEMNIAPGKPLELLLRGCSADAERRVNEN
RGFLQTLARLESITVLPADDKGPVSVTKIIDGAELLIPMAGLINKEDELARLAKEVAKIE
GEISRIENKLANEGFVARAPEAVIAKEREKLEGYAEAKAKLIEQQAVIAAL
>P11880
MISVTLSQLTDILNGELQGADITLDAVTTDTRKLTPGCLFVALKGERFDAHDFADQAKAG
GAGALLVSRPLDIDLPQLIVKDTRLAFGELAAWVRQQVPARVVALTGSSGKTSVKEMTAA
ILSQCGNTLYTAGNLNNDIGVPMTLLRLTPEYDYAVIELGANHQGEIAWTVSLTRPEAAL

VNNLAAAHLEGFGSLAGVAKAKGEIFSGLPENGIAIMNADNNDWLNWQSVIGSRKVWRFS
PNAANSDFTATNIHVTSHGTEFTLQTPTGSVDVLLPLPGRHNIANALAAAALSMSVGATL
DAIKAGLANLKAVPGRLFPIQLAENQLLLDDSYNANVGSMTAAVQVLAEMPGYRVLVVGD
MAELGAESEACHVQVGEAAKAAGIDRVLSVGKQSHAISTASGVGEHFADKTALITRLKLL
IAEQQVITILVKGSRSAAMEEVVRALQENGTC
>P72324
MRTRAAVAVEAGKPLEIMEVNLEGPKAGEVMVEIKATGICHTDEFTLSGADPEGMFPAIL
GHEGAGVVVEVGPGVTSVKPGDHVIPLYTPECRQCPSCLSQKTNLCTAIRGTQGQGLMPD
GTSRFSMLDGTPILHYMGCSTFSNYTVLPEIAVAKVRPDAPFDKICYIGCGVTTGIGAVI
NTAKVEIGAKAVVFGLGGIGLNVIQGLKLAGADMIIGVDLNNAKKEWGERFGMTHFVNPS
EIDGDVVAHLVNMTKTPFDQIGGADYTFDCTGNVKVMRQALEACHRGWGQSIVIGVAPAG
AEIQTRPFQLVTGRVWKGSAFGGARGRTDVPKIVDWYMEGKIQIDPMITHILSLEEINKG
FDLMHAGESIRSVVVF
>P0A9A6
MFEPMELTNDAVIKVIGVGGGGGNAVEHMVRERIEGVEFFAVNTDAQALRKTAVGQTIQI
GSGITKGLGAGANPEVGRNAADEDRDALRAALEGADMVFIAAGMGGGTGTGAAPVVAEVA
KDLGILTVAVVTKPFNFEGKKRMAFAEQGITELSKHVDSLITIPNDKLLKVLGRGISLLD
AFGAANDVLKGAVQGIAELITRPGLMNVDFADVRTVMSEMGYAMMGSGVASGEDRAEEAA
EMAISSPLLEDIDLSGARGVLVNITAGFDLRLDEFETVGNTIRAFASDNATVVIGTSLDP
DMNDELRVTVVATGIGMDKRPEITLVTNKQVQQPVMDRYQQHGMAPLTQEQKPVAKVVND
NAPQTAKEPDYLDIPAFLRKQAD
>P22319
MRAPHKDEIASHELPATPMDPALAANREGKIKVATIGLCGCWGCTLSFLDMDERLLPLLE
KVTLLRSSLTDIKRIPERCAIGFVEGGVSSEENIETLEHFRENCDILISVGACAVWGGVP
AMRNVFELKDCLAEAYVNSATAVPGAKAVVPFHPDIPRITTKVYPCHEVVKMDYFIPGCP
PDGDAIFKVLDDLVNGRPFDLPSSINRYD
>P76145
MSDWNPSLYLHFSAERSRPAVELLARVPLENVEYVADLGCGPGNSTALLQQRWPAARITG
IDSSPAMIAEARSALPDCQFVEADIRNWQPVQALDLIFANASLQWLPDHYELFPHLVSLL
NPQGVLAVQMPDNWLEPTHVLMREVAWEQNYPDRGREPLAGVHAYYDILSEAGCEVDIWR
TTYYHQMPSHQAIIDWVTATGLRPWLQDLTESEQQLFLKRYHQMLEEQYPLQENGQILLA
FPRLFIVARRME
>Q7VZ05
MNTRVLTGITTTGTPHLGNYAGAIRPAIQASTQPGVDAFFFLADYHALIKCDDPARVARS
RLELAATWLAAGLDPERVTFYRQSDIPEITELCWLLTCVTPKGLMNRAHAYKASVDQNAA
KGVEPDDGVTMGLFSYPVLMAADILLFNANQVPVGRDQVQHLEMARDIAQRFNHLYGREF
FVLPEVVIAEEVATLPGLDGRKMSKSYNNTIPLFEGGAAGLRNATQRIVTDSRLPGEPKD
AEASHLYMLYRAFSTQQESMAFRRQLEEGMGWGDAKQALYERLERDLAPMRERYVELISN
PGLIEDILQVGAAKARKLAQPLVRTLRDAVGLGVLQPAAAKAAQPARKAAKDARFVSFRD
EDGSFRFRLLAADGEELLCSVPFANPKEAGALMRRLQDEAPEQALRGHDDVSYAAWLDGK
EVAYGPQAADAGARDALLAKAREALAQLAAA
>P0A8G0
MSDQFDAKAFLKTVTSQPGVYRMYDAGGTVIYVGKAKDLKKRLSSYFRSNLASRKTEALV
AQIQQIDVTVTHTETEALLLEHNYIKLYQPRYNVLLRDDKSYPFIFLSGDTHPRLAMHRG

AKHAKGEYFGPFPNGYAVRETLALLQKIFPIRQCENSVYRNRSRPCLQYQIGRCLGPCVE
GLVSEEEYAQQVEYVRLFLSGKDDQVLTQLISRMETASQNLEFEEAARIRDQIQAVRRVT
EKQFVSNTGDDLDVIGVAFDAGMACVHVLFIRQGKVLGSRSYFPKVPGGTELSEVVETFV
GQFYLQGSQMRTLPGEILLDFNLSDKTLLADSLSELAGRKINVQTKPRGDRARYLKLART
NAATALTSKLSQQSTVHQRLTALASVLKLPEVKRMECFDISHTMGEQTVASCVVFDANGP
LRAEYRRYNITGITPGDDYAAMNQVLRRRYGKAIDDSKIPDVILIDGGKGQLAQAKNVFA
ELDVSWDKNHPLLLGVAKGADRKAGLETLFFEPEGEGFSLPPDSPALHVIQHIRDESHDH
AIGGHRKKRAKVKNTSSLETIEGVGPKRRQMLLKYMGGLQGLRNASVEEIAKVPGISQGL
AEKIFWSLKH
>P08997
MTEQATTTDELAFTRPYGEQEKQILTAEAVEFLTELVTHFTPQRNKLLAARIQQQQDIDN
GTLPDFISETASIRDADWKIRGIPADLEDRRVEITGPVERKMVINALNANVKVFMADFED
SLAPDWNKVIDGQINLRDAVNGTISYTNEAGKIYQLKPNPAVLICRVRGLHLPEKHVTWR
GEAIPGSLFDFALYFFHNYQALLAKGSGPYFYLPKTQSWQEAAWWSEVFSYAEDRFNLPR
GTIKATLLIETLPAVFQMDEILHALRDHIVGLNCGRWDYIFSYIKTLKNYPDRVLPDRQA
VTMDKPFLNAYSRLLIKTCHKRGAFAMGGMAAFIPSKDEEHNNQVLNKVKADKSLEANNG
HDGTWIAHPGLADTAMAVFNDILGSRKNQLEVMREQDAPITADQLLAPCDGERTEEGMRA
NIRVAVQYIEAWISGNGCVPIYGLMEDAATAEISRTSIWQWIHHQKTLSNGKPVTKALFR
QMLGEEMKVIASELGEERFSQGRFDDAARLMEQITTSDELIDFLTLPGYRLLA
>P05827
MDLRDLKTFLHLAESRHFGRSARAMHVSPSTLSRQIQRLEEDLGQPLFVRDNRTVTLTEA
GEELRVFAQQTLLQYQQLRHTIDQQGPSLSGELHIFCSVTAAYSHLPPILDRFRAEHPSV
EIKLTTGDAADAMEKVVTGEADLAIAGKPETLPGAVAFSMLENLAVVLIAPALPCPVRNQ
VSVEKPDWSTVPFIMADQGPVRRRIELWFRRNKISNPMIYATVGGHEAMVSMVALGCGVA
LLPEVVLENSPEPVRNRVMILERSDEKTPFELGVCAQKKRLHEPLIEAFWKILPNHK
>P23996
MASEQNPLGLLGIEFTEFATPDLDFMHKVFIDFGFSKLKKHKQKDIVYYKQNDINFLLNN
EKQGFSAQFAKTHGPAISSMGWRVEDANFAFEGAVARGAKPAADEVKDLPYPAIYGIGDS
LIYFIDTFGDDNNIYTSDFEALDEPIITQEKGFIEVDHLTNNVHKGTMEYWSNFYKDIFG
FTEVRYFDIKGSQTALISYALRSPDGSFCIPINEGKGDDRNQIDEYLKEYDGPGVQHLAF
RSRDIVASLDAMEGSSIQTLDIIPEYYDTIFEKLPQVTEDRDRIKHHQILVDGDEDGYLL
QIFTKNLFGPIFIEIIQRKNNLGFGEGNFKALFESIERDQVRRGVL
>P0A1F0
MAKLRVGIVFGGKSAEHEVSLQSAKNIVDAIDKTRFDVVLLGIDKAGQWHVNDAENYLQN
ADDPAHIALRPSAISLAQVPGKHQHQLINAQNGQPLPTVDVIFPIVHGTLGEDGSLQGML
RVANLPFVGSDVLSSAACMDKDVAKRLLRDAGLNIAPFITLTRTNRHAFSFAEVESRLGL
PLFVKPANQGSSVGVSKVANEAQYQQAVALAFEFDHKVVVEQGIKGREIECAVLGNDNPQ
ASTCGEIVLNSEFYAYDTKYIDDNGAQVVVPAQIPSEVNDKIRAIAIQAYQTLGCAGMAR
VDVFLTADNEVVINEINTLPGFTNISMYPKLWQASGLGYTDLISRLIELALERHTANNAL
KTTM
>P0AGG4
MNTVCTHCQAINRIPDDRIEDAAKCGRCGHDLFDGEVINATGETLDKLLKDDLPVVIDFW
APWCGPCRNFAPIFEDVAQERSGKVRFVKVNTEAERELSSRFGIRSIPTIMIFKNGQVVD
MLNGAVPKAPFDSWLNESL

>P69819
MTKIIAVTACPSGVAHTYMAAEALESAAKAKGWEVKVETQGSIGLENELTAEDVASADMV
ILTKDIGIKFEERFAGKTIVRVNISDAVKRADAIMSKIEAHLAQTA
>P0A6G7
MSYSGERDNFAPHMALVPMVIEQTSRGERSFDIYSRLLKERVIFLTGQVEDHMANLIVAQ
MLFLEAENPEKDIYLYINSPGGVITAGMSIYDTMQFIKPDVSTICMGQAASMGAFLLTAG
AKGKRFCLPNSRVMIHQPLGGYQGQATDIEIHAREILKVKGRMNELMALHTGQSLEQIER
DTERDRFLSAPEAVEYGLVDSILTHRN
>P08178
MTDKTSLSYKDAGVDIDAGNALVGRIKGVVKKTRRPEVMGGLGGFGALCALPQKYREPVL
VSGTDGVGTKLRLAMDLKRHDTIGIDLVAMCVNDLVVQGAEPLFFLDYYATGKLDVDTAS
AVISGIAEGCLQSGCSLVGGETAEMPGMYHGEDYDVAGFCVGVVEKSEIIDGSKVSDGDV
LIALGSSGPHSNGYSLVRKILEVSGCDPQTTELDGKPLADHLLAPTRIYVKSVLELIEKV
DVHAIAHLTGGGFWENIPRVLPDNTQAVIDESSWQWPEVFNWLQTAGNVEHHEMYRTFNC
GVGMIIALPAPEVDKALALLNANGENAWKIGIIKASDSEQRVVIE
>Q07739
MMVHNYCAPENVTAPPHVSRVTQYNKRGIRMISKVTWRVAWESDLTNGDHAELSDFFKSV
YGATGAFNALPFAGGRSWAGARPELRGIAYDESGVAAHMGVLRRFIKVGGEQIAVAELGL
YGVRRDLEGLGIGHSTLAMLPVLKALGVPFAFGCFRNELRIHFQRFCRNGKGAIVDNVNI
KSTQPDIYPDLPPTKIEKKAAVILPLTETLDRWPEGVDIERNGPEL
>P37330
MSQTITQSRLRIDANFKRFVDEEVLPGTGLDAAAFWRNFDEIVHDLAPENRQLLAERDRI
QAALDEWHRSNPGPVKDKAAYKSFLRELGYLVPQPERVTVETTGIDSEITSQAGPQLVVP
AMNARYALNAANARWGSLYDALYGSDIIPQEGAMVSGYDPQRGEQVIAWVRRFLDESLPL
ENGSYQDVVAFKVVDKQLRIQLKNGKETTLRTPAQFVGYRGDAAAPTCILLKNNGLHIEL
QIDANGRIGKDDPAHINDVIVEAAISTILDCEDSVAAVDAEDKILLYRNLLGLMQGTLQE
KMEKNGRQIVRKLNDDRHYTAADGSEISLHGRSLLFIRNVGHLMTIPVIWDSEGNEIPEG
ILDGVMTGAIALYDLKVQKNSRTGSVYIVKPKMHGPQEVAFANKLFTRIETMLGMAPNTL
KMGIMDEERRTSLNLRSCIAQARNRVAFINTGFLDRTGDEMHSVMEAGPMLRKNQMKSTP
WIKAYERNNVLSGLFCGLRGKAQIGKGMWAMPDLMADMYSQKGDQLRAGANTAWVPSPTA
ATLHALHYHQTNVQSVQANIAQTEFNAEFEPLLDDLLTIPVAENANWSAQEIQQELDNNV
QGILGYVVRWVEQGIGCSKVPDIHNVALMEDRATLRISSQHIANWLRHGILTKEQVQASL
ENMAKVVDQQNAGDPAYRPMAGNFANSCAFKAASDLIFLGVKQPNGYTEPLLHAWRLREK
ESH
>P23872
MKPENKLPVLDLISAEMKTVVNTLQPDLPPWPATGTIAEQRQYYTLERRFWNAGAPEMAT
RAYMVPTKYGQVETRLFCPQPDSPATLFYLHGGGFILGNLDTHDRIMRLLASYSQCTVIG
IDYTLSPEARFPQAIEEIVAACCYFHQQAEDYQINMSRIGFAGDSAGAMLALASALWLRD
KQIDCGKVAGVLLWYGLYGLRDSVTRRLLGGVWDGLTQQDLQMYEEAYLSNDADRESPYY
CLFNNDLTREVPPCFIAGAEFDPLLDDSRLLYQTLAAHQQPCEFKLYPGTLHAFLHYSRM
MKTADEALRDGAQFFTAQL
>P0A9I3
MTLSSQHYLVITALGADRPGIVNTITRHVSSCGCNIEDSRLAMLGEEFTFIMLLSGSWNA
ITLIESTLPLKGAELDLLIVMKRTTARPRPPMPASVWVQVDVADSPHLIERFTALFDAHH

MNIAELVSRTQPAENERAAQLHIQITAHSPASADAANIEQAFKALCTELNAQGSINVVNY
SQHDEQDGVK
>P0A821
MTTETRSLYSQLPAIDRLLRDSSFLSLRDTYGHTRVVELLRQMLDEAREVIRGSQTLPAW
CENWAQEVDARLTKEAQSALRPVINLTGTVLHTNLGRALQAEAAVEAVAQAMRSPVTLEY
DLDDAGRGHRDRALAQLLCRITGAEDACIVNNNAAAVLLMLAATASGKEVVVSRGELVEI
GGAFRIPDVMRQAGCTLHEVGTTNRTHANDYRQAVNENTALLMKVHTSNYSIQGFTKAID
EAELVALGKELDVPVVTDLGSGSLVDLSQYGLPKEPMPQELIAAGVSLVSFSGDKLLGGP
QAGIIVGKKEMIARLQSHPLKRALRADKMTLAALEATLRLYLHPEALSEKLPTLRLLTRS
AEVIQIQAQRLQAPLAAHYGAEFAVQVMPCLSQIGSGSLPVDRLPSAALTFTPHDGRGSH
LESLAARWRELPVPVIGRIYDGRLWLDLRCLEDEQRFLEMLLK
>P0A2W5
MTATFDKVADIIAETSEIDRATITPESHTIDDLGIDSLDFLDIVFAIDKEFGIKIPLEKW
TQEVNEGKVSTEEYFVLKNLCAKIDELKAAKA
>P07294
MAKVQAYVSDEIVYKINKIVERRRAEGAKSTDVSFSSISTMLLELGLRVYEAQMERKESA
FNQAEFNKVLLECAVKTQSTVAKILGIESLSPHVSGNPKFEYANMVEDIRDKVSSEMERF
FPENDEE
>P62601
MLNQKIQNPNPDELMIEVDLCYELDPYELKLDEMIEAEPEPEMIEGLPASDALTPADRYL
ELFEHVQSAKIFPDSKTFPDCAPKMDPLDILIRYRKVRRHRDFDLRKFVENHFWLPEVYS
SEYVSDPQNSLKEHIDQLWPVLTREPQDHIPWSSLLALPQSYIVPGGRFSETYYWDSYFT
MLGLAESGREDLLKCMADNFAWMIENYGHIPNGNRTYYLSRSQPPVFALMVELFEEDGVR
GARRYLDHLKMEYAFWMDGAESLIPNQAYRHVVRMPDGSLLNRYWDDRDTPRDESWLEDV
ETAKHSGRPPNEVYRDLRAGAASGWDYSSRWLRDTGRLASIRTTQFIPIDLNAFLFKLES
AIANISALKGEKETEALFRQKASARRDAVNRYLWDDENGIYRDYDWRREQLALFSAAAIV
PLYVGMANHEQADRLANAVRSRLLTPGGILASEYETGEQWDKPNGWAPLQWMAIQGFKMY
GDDLLGDEIARSWLKTVNQFYLEQHKLIEKYHIADGVPREGGGGEYPLQDGFGWTNGVVR
RLIGLYGEP
>P44853
MSEQKQDVAATEEQQPVLQIQRIYVKDVSFEAPNLPHIFQQEWKPKLGFDLSTETTQVGD
DLYEVVLNISVETTLEDSGDVAFICEVKQAGVFTISGLEDVQMAHCLTSQCPNMLFPYAR
ELVSNLVNRGTFPALNLSPVNFDALFVEYMNRQQAENAEEKSEEEQTKH
>P22939
MDFPQQLEACVKQANQALSRFIAPLPFQNTPVVETMQYGALLGGKRLRPFLVYATGHMFG
VSTNTLDAPAAAVECIHAYSLIHDDLPAMDDDDLRRGLPTCHVKFGEANAILAGDALQTL
AFSILSDADMPEVSDRDRISMISELASASGIAGMCGGQALDLDAEGKHVPLDALERIHRH
KTGALIRAAVRLGALSAGDKGRRALPVLDKYAESIGLAFQVQDDILDVVGDTATLGKRQG
ADQQLGKSTYPALLGLEQARKKARDLIDDARQSLKQLAEQSLDTSALEALADYIIQRNK
>P57777
MTQKLAVVLFNLGGPDGPDAVRPFLFNLFRDPAIIGAPALIRYPLAALISTTREKSAKAN
YAIMGGGSPLLPETEKQARALEAALALAMPGVEAKCFIAMRYWHPLTDETARQVAAFAPD
QVVLLPLYPQFSTTTTGSSLKAWKKTYKGSGVQTTVGCYPTEGGLIEAHARMIRESWEKA
GSPTNIRLLFSAHGLPEKVILAGDPYQKQVEATAAAVAAHLPPQIEWTVCYQSRVGPLKW

IGPSTDDEIRRAGGEDKGVMITPIAFVSEHVETLVELDHEYAELAEEVGAAPYLRVSALG
TAPEFIDGLAKAVRDSVGKAPGTVSSACGWRCGADWSKCPCREGASA
>P21517
MMLNAWHLPVPPFVKQSKDQLLITLWLTGEDPPQRIMLRTEHDNEEMSVPMHKQRSQPQP
GVTAWRAAIDLSSGQPRRRYSFKLLWHDRQRWFTPQGFSRMPPARLEQFAVDVPDIGPQW
AADQIFYQIFPDRFARSLPREAEQDHVYYHHAAGQEIILRDWDEPVTAQAGGSTFYGGDL
DGISEKLPYLKKLGVTALYLNPVFKAPSVHKYDTEDYRHVDPQFGGDGALLRLRHNTQQL
GMRLVLDGVFNHSGDSHAWFDRHNRGTGGACHNPESPWRDWYSFSDDGTALDWLGYASLP
KLDYQSESLVNEIYRGEDSIVRHWLKAPWNMDGWRLDVVHMLGEAGGARNNMQHVAGITE
AAKETQPEAYIVGEHFGDARQWLQADVEDAAMNYRGFTFPLWGFLANTDISYDPQQIDAQ
TCMAWMDNYRAGLSHQQQLRMFNQLDSHDTARFKTLLGRDIARLPLAVVWLFTWPGVPCI
YYGDEVGLDGKNDPFCRKPFPWQVEKQDTALFALYQRMIALRKKSQALRHGGCQVLYAED
NVVVFVRVLNQQRVLVAINRGEACEVVLPASPFLNAVQWQCKEGHGQLTDGILALPAISA
TVWMN
>P50199
MSHPDLFSLSGARALVTGASRGIGLTLAKGLARYGAEVVLNGRNAESLDSAQSGFEAEGL
KASTAVFDVTDQDAVIDGVAAIERDMGPIDILINNAGIQRRAPLEEFSRKDWDDLMSTNV
NAVFFVGQAVARHMIPRGRGKIVNICSVQSELARPGIAPYTATKGAVKNLTKGMATDWGR
HGLQINGLAPGYFATEMTERLVADEEFTDWLCKRTPAGRWGQVEELVGAAVFLSSRASSF
VNGQVLMVDGGITVSL
>P69506
MAYRDQPLGELALSIPRASALFRKYDMDYCCGGKQTLARAAARKELDVEVIEAELAKLAE
QPIEKDWRSAPLAEIIDHIIVRYHDRHREQLPELILQATKVERVHADKPSVPKGLTKYLT
MLHEELSSHMMKEEQILFPMIKQGMGSQAMGPISVMESEHDEAGELLEVIKHTTNNVTPP
PEACTTWKAMYNGINELIDDLMDHISLENNVLFPRALAGE
>A4JIA2
MLPAHKQTLEALLADSVAQVAHALKGADAEFVIPAITLERPKVAAHGDVACNVAMQLAKP
LGTNPRQLAERIVAALVAQPAAQGLVDAAEIAGPGFINLRVSAAAKQAVIAAVFEQGRAF
GTSQREKGKRVLVEFVSANPTGPLHVGHGRQAALGDVLANVIASQGYAVHREFYYNDAGV
QIANLAISTQARARGLKPGDAGWPEAAYNGEYIADIARDYLNGATVAAKDGEPVTGARDI
ENLDAIRKFAVAYLRHEQDMDLQAFGVKFDQYYLESSLYSEGRVEKTVDALVKAGMTYEQ
DGALWLRTTDEGDDKDRVMRKSDGTYTYFVPDVAYHVTKWERGFTKVINIQGSDHHGTIA
RVRAGLQGLHIGIPKGYPDYVLHKMVTVMRDGQEVKLSKRAGSYVTVRDLIEWSGGAAPG
QEAAPDMIDEATITRGRDAVRFFLISRKADTEFVFDIDLALKQNDENPVYYVQYAHARIC
SVLNELKARYNVDVAQLPGADLSQLTSPQAVSLMQKLAEYPDLLTHAANELAPHAVAFYL
RDLAGEFHSFYNAERVLVDDEAPRNARAALLAATRQVLENGLAMLGVSAPAKM
>P45754
MYTQFFGLSEPPFSISPNPKYLYMSERHGEALAHLNYGLQDGGGFVLLTGEVGTGKTTVS
RCLLQQLPTETEIAYILNPSLTERDLLAAICDEFQLPYDKDAGLKLLFDLIRDHLLANLA
AGKRSVVLVDEAQHLLPGVLEQLRLLTNLETDEKKLLQVVLIGQPELQQMLRQPLLRQLA
QRITARYHLLPLSHQDVDAYVRFRLQVAGCVQPIFTPKALQTLHRLSGGIPRLINLICDR
ALIAAFARGSHKIVHGDISLAAYEVSGIRDEGTWQSGLMVALAGALLVATGWWGWQFFGF
FPERPVIKVEVPVKVDDTPEQQEQLTRAINQALEPDSAMQNLYKVWGYQTELEEATCDNA
PRAGLRCQEGDASLAELQALQHPALISLTDETGGIYYATLVNLGPDKANLLIGNQSWQVD

RQWLSDFWGGSYTLLWRMPKGGVALIGNNAGATQVQWLDNALSRALQQPDRKVRRFDAEL
KNKLQQFQREQGLNPDGIAGSNTLLRLNVMAGEPMPKLEDESQRASTPATPDTMNDEPMV
TLSEEAS
>P51837
MNHLNKLMERLGHQFNNLELLKIALTHCSSGADNNERLEFLGDSVLGFIIASELYQRRPQ
AREGDLSRMRASMVNGDELAQMSTKLGINEYLQLGVGEQKSGGKRRRSILADALEAIVGA
IYIDAGLETCRRCVLNWYGERVDDLSKLSPKKDAKSLLQEWLQARRLPLPTYEVKITGEA
HAQTFTVNCYVKGLPHKTEGVNTTRRRAEQIAAKRFLELLDDGKGDGITERDQ
>P0A6D0
MRSSAKQEELVKAFKALLKEEKFSSQGEIVAALQEQGFDNINQSKVSRMLTKFGAVRTRN
AKMEMVYCLPAELGVPTTSSPLKNLVLDIDYNDAVVVIHTSPGAAQLIARLLDSLGKAEG
ILGTIAGDDTIFTTPANGFTVKDLYEAILELFDQEL
>P00963
MKTAYIAKQRQISFVKSHFSRQLEERLGLIEVQAPILSRVGDGTQDNLSGCEKAVQVKVK
ALPDAQFEVVHSLAKWKRQTLGQHDFSAGEGLYTHMKALRPDEDRLSPLHSVYVDQWDWE
RVMGDGERQFSTLKSTVEAIWAGIKATEAAVSEEFGLAPFLPDQIHFVHSQELLSRYPDL
DAKGRERAIAKDLGAVFLVGIGGKLSDGHRHDVRAPDYDDWSTPSELGHAGLNGDILVWN
PVLEDAFELSSMGIRVDADTLKHQLALTGDEDRLELEWHQALLRGEMPQTIGGGIGQSRL
TMLLLQLPHIGQVQCGVWPAAVRESVPSLL
>P56601
MHHMPRTTGMNVAVVGGGISGLAVAHHLRSRGTDAVLLESSARLGGAVGTHALAGYLVEQ
GPNSFLDREPATRALAAALNLEGRIRAADPAAKRRYVYTRGRLRSVPASPPAFLASDILP
LGARLRVAGELFSRRAPEGVDESLAAFGRRHLGHRATQVLLDAVQTGIYAGDVEQLSVAA
TFPMLVKMEREHRSLILGAIRAQKAQRQAALPAGTAPKLSGALSTFDGGLQVLIDALAAS
LGDAAHVGARVEGLAREDGGWRLIIEEHGRRAELSVAQVVLAAPAHATAKLLRPLDDALA
ALVAGIAYAPIAVVHLGFDAGTLPAPDGFGFLVPAEEQRRMLGAIHASTTFPFRAEGGRV
LYSCMVGGARQPGLVEQDEDALAALAREELKALAGVTARPSFTRVFRWPLGIPQYNLGHL
ERVAAIDAALQRLPGLHLIGNAYKGVGLNDCIRNAAQLADALVAGNTSHAP
>P34895
MSSAPAAGTASTSRFFKSHVSETDPDIFSAIQKEFGRQQHEIELIASENIVSQAVLDAAG
SVLTNKYAEGYPGKRYYGGCQYVDIVEDIAIDRAKKLFNCEFANVQPNSGSQANQGVFNA
LAQPGDTILGLSLAAGGHLTHGAPVNQSGKWFKAVHYMVKPDSHLIDMDEVRKLAQEHKP
RIIIAGGSAYPRKIDFAAFRAIADEVGAIFLVDMAHFAGLVAAGLIPSPFPHAHVVTTTT
HKTLRGPRGGMILTNDADIAKKINSAIFPGIQGGPLMHVIAGKAVAFGEALRPDFKVYIK
QVMDNARALGEVLVQNGFALVSGGTDTHLVLVDLRPKKLTGTKAEKALGRANITCNKNGI
PFDPEKPMVTSGIRLGSPAGTTRGFGVAEFQEIGRLISEVLDGVAKNGEDGNGAVEAAVK
AKAIALCDRFPIYA
>P0A6Q3
MVDKRESYTKEDLLASGRGELFGAKGPQLPAPNMLMMDRVVKMTETGGNFDKGYVEAELD
INPDLWFFGCHFIGDPVMPGCLGLDAMWQLVGFYLGWLGGEGKGRALGVGEVKFTGQVLP
TAKKVTYRIHFKRIVNRRLIMGLADGEVLVDGRLIYTASDLKVGLFQDTSAF
>Q492P3
MNVVSVVDILSGHVSKNTEITIQGWIRTRRDSKAKISFLDLYDGSCINSLQIIAYDKLHN
YKNEILRLTSGCSVIIVGIIVKSIGIKQHVEVIAKNIKILGWIEDPSTYPITAKKHTMEY

LREVSHLRPRTNTIGAVARIRDTLSQAIHNFLHKQGFIWIPTPIITACDTEGSSKMFCVS
TSETQKILNNPNEKLHHTHDTTYDFFSKKAFLTVSGQLNAEAYACALSKVYTFGPTFRAE
YSNTNRHLAEFWMIEPEAAFMTLDDIIILAESLLKNIIRILLEKRSDDIKYLVDKINKNI
ITILENFSEIKFNHIEYTEAIKLLEICNRKFNNPIHWGTDLFSEHEKYLSEEYFKSPVII
KNFPKNIKAFYMRLNDDNKTVASMDILVPGIGEIIGGSQREERLSKLDQRLQENCLTQEN
YWWYRDLRRYGTVPHSGFGLGFERLMIYVTGIKNIRDVIPFPRTSKNINF
>P08308
MAFNMHNRNLLSLMHHSTRELRYLLDLSRDLKRAKYTGTEQQHLKRKNIALIFEKTSTRT
RCAFEVAAYDQGANVTYIDPNSSQIGHKESMKDTARVLGRMYDAIEYRGFKQEIVEELAK
FAGVPVFNGLTDEYHPTQMLADVLTMREHSDKPLHDISYAYLGDARNNMGNSLLLIGAKL
GMDVRIAAPKALWPHDEFVAQCKKFAEESGAKLTLTEDPKEAVKGVDFVHTDVWVSMGEP
VEAWGERIKELLPYQVNMEIMKATGNPRAKFMHCLPAFHNSETKVGKQIAEQYPNLANGI
EVTEDVFESPYNIAFEQAENRMHTIKAILVSTLADI
>P0A6K1
MQFSKMHGLGNDFMVVDAVTQNVFFSPELIRRLADRHLGVGFDQLLVVEPPYDPELDFHY
RIFNADGSEVAQCGNGARCFARFVRLKGLTNKRDIRVSTANGRMVLTVTDDDLVRVNMGE
PNFEPSAVPFRANKAEKTYIMRAAEQTILCGVVSMGNPHCVIQVDDVDTAAVETLGPVLE
SHERFPERANIGFMQVVKREHIRLRVYERGAGETQACGSGACAAVAVGIQQGLLAEEVRV
ELPGGRLDIAWKGPGHPLYMTGPAVHVYDGFIHL
>Q3SVA1
MGMSTAVASPVALDSGKLEPLRVMVVDDSVVIRGLISRWIEAEPDMMVAASLRTGRDAVS
QVERADPDVVVLDIEMPELDGISALPQLLAKKRNLIVIMASTLTRRNAEISFKALSLGAS
DYIPKPESTREVAAADIFRHDLMQKIRHLAAKRRRPATVASPPPDHDDYGSNASTIMNAV
DSNISERDAGGKPRRTFPHPALVQREQQPRSAQAARAMSRPQPTLRSFSAHLPRALLIGS
STGGPQALMTLVAGIGPVIDRCPVLITQHMPPTFTTILAEHLARAAGRPAHEGVDQEIVK
QGHIYLAPGGRHMRVARKGADAVIVLDNGPAVNFCKPAVDPLFMSAIDVWQGGALAVILT
GMGSDGMRGGTQIVAAGGSIIAQDEASSVVWGMPGAAVQAGICAAVLPLQQIAPKLVRLF
AGDGL
>P38434
MAVNLTEKTAEQLPDIDGIALYTAQAGVKKPGHTDLTLIAVAAGSTVGAVFTTNRFCAAP
VHIAKSHLFDEDGVRALVINTGNANAGTGAQGRIDALAVCAAAARQIGCKPNQVMPFSTG
VILEPLPADKIIAALPKMQPAFWNEAARAIMTTDTVPKAASREGKVGDQHTVRATGIAKG
SGMIHPNMATMLGFIATDAKVSQPVLQLMTQEIADETFNTITVDGDTSTNDSFVIIATGK
NSQSEIDNIADPRYAQLKELLCSLALELAQAIVRDGEGATKFITVRVENAKTCDEARQAA
YAAARSPLVKTAFFASDPNLGKRLAAIGYADVADLDTDLVEMYLDDILVAEHGGRAASYT
EAQGQAVMSKDEITVRIKLHRGQAAATVYTCDLSHGYVSINADYRS
>P0A1Y2
MSIDIDIIKARAKNEYRLSKVRGEAMISVRIPGGILPAHLLTVARDIAETWGNGQIHLTT
RQKLAMPGIRYEDIDNVNAALEPFLREIEIELCDVQVEDTKAGYLAIGGRNIVACQGNRI
CQKANTDTTGLSRRLEKLVYPSPYHLKTVIVGCPNDCAKASMADLGIIGVAKMRFTADRC
IGCGACVKACSHHAVGCLALKNGKAVKEESACIGCGECVLACPTLAWQRKPDQLWQVRLG
GRTSKKTPRVGKLFLNWVTEDVIKQVIVNLYEFEKEMLGGKPIYLHMGHLIDKGGYLRFK
ERVLRGVQLNPEAMVAERIYWAEDESVARMHLKPAGH
>P43313

MKTFEILKHLQADAIVLFMKVHNFHWNVKGTDFFNVHKATEEIYEEFADMFDDLAERIVQ
LGHHPLVTLSEAIKLTRVKEETKTSFHSKDIFKEILEDYKYLEKEFKELSNTAEKEGDKV
TVTYADDQLAKLQKSIWMLQAHLA
>P28629
MKVLIVESEFLHQDTWVGNAVERLADALSQQNVTVIKSTSFDDGFAILSSNEAIDCLMFS
YQMEHPDEHQNVRQLIGKLHERQQNVPVFLLGDREKALAAMDRDLLELVDEFAWILEDTA
DFIAGRAVAAMTRYRQQLLPPLFSALMKYSDIHEYSWAAPGHQGGVGFTKTPAGRFYHDY
YGENLFRTDMGIERTSLGSLLDHTGAFGESEKYAARVFGADRSWSVVVGTSGSNRTIMQA
CMTDNDVVVVDRNCHKSIEQGLMLTGAKPVYMVPSRNRYGIIGPIYPQEMQPETLQKKIS
ESPLTKDKAGQKPSYCVVTNCTYDGVCYNAKEAQDLLEKTSDRLHFDEAWYGYARFNPIY
ADHYAMRGEPGDHNGPTVFATHSTHKLLNALSQASYIHVREGRGAINFSRFNQAYMMHAT
TSPLYAICASNDVAVSMMDGNSGLSLTQEVIDEAVDFRQAMARLYKEFTADGSWFFKPWN
KEVVTDPQTGKTYDFADAPTKLLTTVQDCWVMHPGESWHGFKDIPDNWSMLDPIKVSILA
PGMGEDGELEETGVPAALVTAWLGRHGIVPTRTTDFQIMFLFSMGVTRGKWGTLVNTLCS
FKRHYDANTPLAQVMPELVEQYPDTYANMGIHDLGDTMFAWLKENNPGARLNEAYSGLPV
AEVTPREAYNAIVDNNVELVSIENLPGRIAANSVIPYPPGIPMLLSGENFGDKNSPQVSY
LRSLQSWDHHFPGFEHETEGTEIIDGIYHVMCVKA
>Q46877
MSIVVKNNIHWVGQRDWEVRDFHGTEYKTLRGSSYNSYLIREEKNVLIDTVDHKFSREFV
QNLRNEIDLADIDYIVINHAEEDHAGALTELMAQIPDTPIYCTANAIDSINGHHHHPEWN
FNVVKTGDTLDIGNGKQLIFVETPMLHWPDSMMTYLTGDAVLFSNDAFGQHYCDEHLFND
EVDQTELFEQCQRYYANILTPFSRLVTPKITEILGFNLPVDMIATSHGVVWRDNPTQIVE
LYLKWAADYQEDRITIFYDTMSNNTRMMADAIAQGIAETDPRVAVKIFNVARSDKNEILT
NVFRSKGVLVGTSTMNNVMMPKIAGLVEEMTGLRFRNKRASAFGSHGWSGGAVDRLSTRL
QDAGFEMSLSLKAKWRPDQDALKLCREHGREIARQWALAPLPQSTVNTVVKEETSATTTA
DLGPRMQCSVCQWIYDPAKGEPMQDVAPGTPWSEVPDNFLCPECSLGKDVFEELASEAK
>P0A7B8
MTTIVSVRRNGHVVIAGDGQATLGNTVMKGNVKKVRRLYNDKVIAGFAGGTADAFTLFEL
FERKLEMHQGHLVKAAVELAKDWRTDRMLRKLEALLAVADETASLIITGNGDVVQPENDL
IAIGSGGPYAQAAARALLENTELSAREIAEKALDIAGDICIYTNHFHTIEELSYKA
>P10772
MAINIILLGPPGAGKGTQARRLIDERGLVQLSTGDMLREARSSGTEMGKRVAEVMDRGEL
VTDEIVIGLIREKLGQGGKGFIFDGFPRTLAQADALQALMAEMDQRIDAVIEMRVDDAAL
VSRISGRFTCGNCGEVYHDVTKPTKEPGKCDVCGSTDLRRRADDNEESLKTRLMEYYKKT
SPLIGYYYVKGNLNPVDGLAEIDEVAAQVAKVMDKIPA
>P0A8T1
MPWIQLKLNTTGANAEDLSDALMEAGAVSITFQDTHDTPVFEPLPGETRLWGDTDVIGLF
DAETDMNDVVAILENHPLLGAGFAHKIEQLEDKDWEREWMDNFHPMRFGERLWICPSWRD
VPDENAVNVMLDPGLAFGTGTHPTTSLCLQWLDSLDLTGKTVIDFGCGSGILAIAALKLG
AAKAIGIDIDPQAIQASRDNAERNGVSDRLELYLPKDQPEEMKADVVVANILAGPLRELA
PLISVLPVSGGLLGLSGILASQAESVCEAYADSFALDPVVEKEEWCRITGRKN
>Q2N6X5
MTELLKISLPDGSVREMEAGATPADVAAAIGPGLAKAALAAKVDGEVRDLNRPFEGDAEL
ALITSRDEEEALELARHDYAHVLAEAVQALWPGTQITFGPATDDGFYYDVKAPDSRDPFG

MDDLPAIEEKMREIIKADKPLVREVWSREQLIEKWEAEGEVFKAEWAKELPEDEELTVYW
SGEPGGEDSWLDMCRGPHLASTGKLDPQAFKLMRVAGAYWRGDQRNPQLTRIYGTGWLNK
KQLNAHLHRLEEAAKRDHRKLGREMDLFHLQEEAHGSVFWHPQGYKIWRELEAYMRRKMD
GAGYREIKTPQVMDARQWEQSGHWGKYRENMFVIPDEIPNTEDEGELVSKDADWMALKPM
NCPAHVLVFKQGITSYRDLPIRLGEMGCCHRNEPHGALHGLMRVRQFTQDDAHIFCTEDQ
VVEEVRAFCKLADEVYRDFGFDYDVKLALRPEQRFGSEEDWDKAEQELRDAVAEAGMAND
DYGWEELPGEGAFYAPKLEWHLTDAIGRTWQVGTIQGDRVLPERLDATYVGEDGGKHRPV
MLHRAIFGSYERFIGILIEHFAGRLPVWLAPTQAVVATIVSDADGYAKEAVAKLEAAGIR
VDGDLRNEKINFKVREHSLAKVPHLLVVGKREAEEGTVAVRTLGEKEQQVMSLDDAIAML
KDAATPPDLRDG
>P43927
MIIVTSGHVDHGKTALLKALTGTSTAHLPEEKKRGMTIDLGYAYLPLENKVLGFIDVPGH
EKFLSNMLAGLGGVHYAMLIVAADEGVAVQTKEHLAILRQLQFHEIIVVITKADRTNSAQ
IESLIQTIKQDYSFLRNANYFVTSAETGQGISELRHYLANLAELADTQKPFRYAIDRVFS
VKGAGTVVTGTAFSGTVKVNDEIYLSTGQKIRIKAIHAQNTSSEQGIAGQRLALNLNADL
DRTPMKRGDWLLQNEPLPPTDRISVQILAEVPLNESQPVHIYHGASRTTGKLTLLQGKNA
AKNDRTLAEIILDSPLFLAFGDKLILRSGDTKTLIAGARVLEINSPKRHKRTEVRLNFLA
NLALAENASQRIALTLQHNATTARQLMWTEQLTSLQLDKALAERDAVRYQDWCFNPNYVQ
EKTQQILTALNIYHEQHNDQLGVSKARLYRMATLNQPENLIHHFIDEMLDDGRLQQTRGW
IHLPEHKIQFNTEEKSRWTDVLNEFEKANGQAIWVRDMANALAIDESIMRNFMYKAGKLG
YLTPIVKDRFFLTETLYAYARLIKQIAEEKGKVSVNEVRDKLNFGRKLTVQLMEYFDRMG
FLRRKGNDHILRDKNVFDL
>P61698
MKSSEIRNAFIKYFEKNGHKVVPSSSLIPENDPTLLFANAGMNQFKNTFLGLEKRDYSRA
VTAQKCVRAGGKHNDLENVGFTARHHTFFEMVGNFSFGDYFKKDAIHFAWEFLTKELAIP
KEKLYVTVHISDDEAADIWHNQEGVPRERIFRFDKDNFWKMGDTGPCGPCTEIFYDHGPK
AGTISDPFKGIEAGEDRFVEIWNLVFMQYFENPPGTLTPLPKPSVDTGGGLERMSAAMQG
VFNNYDTDLFQPMIQLACKIGNIEYISDKEVLAKNPAAAEVTSALRVLADHCRSTSFLIA
DGALPSNEGRGYVLRRIMRRAIRYGRKLSADKSFLPGMAEALIESMGSVYPELKTRRDHI
LNTIRDEEDRFIATLDKGTAILEDELKKAKSKGIKELSGEVVFRMYDTYGFPADLTRVIA
NEQGIEVNEAAFEKEMEDNRAKSKASWKGKSMGADEAHMIKFAKDYLQSGKSVTFLGYEG
TIGDGKVMGLSNGQAEVQELKTGDTGLMILNATTFYGEGGGQSGDVGYIMHDTNRARVIN
TTKIDDIVLHHVEIEHGSFKVGTAVVTGVDPVERRNTAANHSATHLLHAALRKVLGTHVT
QAGSLVDSQKTRFDFTHNKPVSSEEIKKIEDLVNEQIARCNPVQTEMMSHKAALEKGAMA
LFGEKYASDVRVLTMGDFSCELCGGTHVKNTSEIRLFKIVSEAGVSSGVRRIEAITADNA
LQYMMSAVTHLDDALAAAGFQKSPHYIKHLETTGETATLANRVESLKDQVKQLEKEMKKL
QGGQVNVDDLAANALTFKTKAGASAKLVLADVPLDDRQVLAEVTDHLKNKIQSGIVVVVG
QGDGSHPIIVSVSKEISGETKAGDLLKEVAGVMGGKGGGRPDFAQGAAPNRAQLNEAFSK
VKSMLGL
>P46889
MSQEYIEDKEVTLTKLSSGRRLLEALLILIVLFAVWLMAALLSFNPSDPSWSQTAWHEPI
HNLGGMPGAWLADTLFFIFGVMAYTIPVIIVGGCWFAWRHQSSDEYIDYFAVSLRIIGVL
ALILTSCGLAAINADDIWYFASGGVIGSLLSTTLQPLLHSSGGTIALLCVWAAGLTLFTG
WSWVTIAEKLGGWILNILTFASNRTRRDDTWVDEDEYEDDEEYEDENHGKQHESRRARIL

RGALARRKRLAEKFINPMGRQTDAALFSGKRMDDDEEITYTARGVAADPDDVLFSGNRAT
QPEYDEYDPLLNGAPITEPVAVAAAATTATQSWAAPVEPVTQTPPVASVDVPPAQPTVAW
QPVPGPQTGEPVIAPAPEGYPQQSQYAQPAVQYNEPLQQPVQPQQPYYAPAAEQPAQQPY
YAPAPEQPVAGNAWQAEEQQSTFAPQSTYQTEQTYQQPAAQEPLYQQPQPVEQQPVVEPE
PVVEETKPARPPLYYFEEVEEKRAREREQLAAWYQPIPEPVKEPEPIKSSLKAPSVAAVP
PVEAAAAVSPLASGVKKATLATGAAATVAAPVFSLANSGGPRPQVKEGIGPQLPRPKRIR
VPTRRELASYGIKLPSQRAAEEKAREAQRNQYDSGDQYNDDEIDAMQQDELARQFAQTQQ
QRYGEQYQHDVPVNAEDADAAAEAELARQFAQTQQQRYSGEQPAGANPFSLDDFEFSPMK
ALLDDGPHEPLFTPIVEPVQQPQQPVAPQQQYQQPQQPVPPQPQYQQPQQPVAPQPQYQQ
PQQPVAPQQQYQQPQQPVAPQQQYQQPQQPVAPQPQDTLLHPLLMRNGDSRPLHKPTTPL
PSLDLLTPPPSEVEPVDTFALEQMARLVEARLADFRIKADVVNYSPGPVITRFELNLAPG
VKAARISNLSRDLARSLSTVAVRVVEVIPGKPYVGLELPNKKRQTVYLREVLDNAKFRDN
PSPLTVVLGKDIAGEPVVADLAKMPHLLVAGTTGSGKSVGVNAMILSMLYKAQPEDVRFI
MIDPKMLELSVYEGIPHLLTEVVTDMKDAANALRWCVNEMERRYKLMSALGVRNLAGYNE
KIAEADRMMRPIPDPYWKPGDSMDAQHPVLKKEPYIVVLVDEFADLMMTVGKKVEELIAR
LAQKARAAGIHLVLATQRPSVDVITGLIKANIPTRIAFTVSSKIDSRTILDQAGAESLLG
MGDMLYSGPNSTLPVRVHGAFVRDQEVHAVVQDWKARGRPQYVDGITSDSESEGGAGGFD
GAEELDPLFDQAVQFVTEKRKASISGVQRQFRIGYNRAARIIEQMEAQGIVSEQGHNGNR
EVLAPPPFD
>P31101
MFCFQCQETAKNTGCTVKGMCGKPEETANLQDLLIFVLRGIAIYGEKLKELGQPDRSNDD
FVLQGLFATITNANWDDARFEAMISEGLARRDKLRNAFLAVYKAKNGKDFSEPLPEAATW
TGDSTAFAEKAKSVGILATENEDVRSLRELLIIGLKGVAAYAEHAAVLGFRKTEIDEFML
EALASTTKDLSVDEMVALVMKAGGMAVTTMALLDEANTTTYGNPEITQVNIGVGKNPGIL
ISGHDLKDMAELLKQTEGTGVDVYTHGEMLPANYYPAFKKYPHFVGNYGGSWWQQNPEFE
SFNGPILLTTNCLVPLKKENTYLDRLYTTGVVGYEGAKHIADRPAGGAKDFSALIAQAKK
CPPPVEIETGSIVGGFAHHQVLALADKVVEAVKSGAIKRFVVMAGCDGRQKSRSYYTEVA
ENLPKDTVILTAGCAKYRYNKLNLGDIGGIPRVLDAGQCNDSYSLAVIALKLKEVFGLDD
INDLPVSYDIAWYEQKAVAVLLALLFLGVKGIRLGPTLPAFLSPNVAKVLVENFNIKPIG
TVQDDIAAMMAGK
>P11886
MQPIRLGLVGYGKIAQDQHVPAINANPAFTLVSVATQGKPCPGVENFQSLGELLENGPPV
DAIAFCTPPQGRFALVQQALAAGKHVLVEKPPCATLGKAALWIKREQASAPCSPCIAYAP
AIAAARDWLATRTLQSVQIDWKEDVRKWHPGQAWIWQPGLGVFDPGINALSIVTHLLPLP
LFVESAELRVPSNCQSPIAASIKMSDPRLLDVRAEFDFDHGHDELWSIQIRCAEGTLRLD
NGGALLSIDGVRQTVAEEGEYAAVYRHFQQLIGDKTSDVDVQPLRLVADSFFVGSRVSVE
AFYD
>P04036
MHDANIRVAIAGAGGRMGRQLIQAALALEGVQLGAALEREGSSLLGSDAGELAGAGKTGV
TVQSSLDAVKDDFDVFIDFTRPEGTLNHLAFCRQHGKGMVIGTTGFDEAGKQAIRDAAAD
IAIVFAANFSVGVNVMLKLLEKAAKVMGDYTDIEIIEAHHRHKVDAPSGTALAMGEAIAH
ALDKDLKDCAVYSREGHTGERVPGTIGFATVRAGDIVGEHTAMFADIGERLEITHKASSR
MTFANGAVRSALWLSGKESGLFDMRDVLDLNNL
>P05055

MLNPIVRKFQYGQHTVTLETGMMARQATAAVMVSMDDTAVFVTVVGQKKAKPGQDFFPLT
VNYQERTYAAGRIPGSFFRREGRPSEGETLIARLIDRPIRPLFPEGFVNEVQVIATVVSV
NPQVNPDIVAMIGASAALSLSGIPFNGPIGAARVGYINDQYVLNPTQDELKESKLDLVVA
GTEAAVLMVESEAQLLSEDQMLGAVVFGHEQQQVVIQNINELVKEAGKPRWDWQPEPVNE
ALNARVAALAEARLSDAYRITDKQERYAQVDVIKSETIATLLAEDETLDENELGEILHAI
EKNVVRSRVLAGEPRIDGREKDMIRGLDVRTGVLPRTHGSALFTRGETQALVTATLGTAR
DAQVLDELMGERTDTFLFHYNFPPYSVGETGMVGSPKRREIGHGRLAKRGVLAVMPDMDK
FPYTVRVVSEITESNGSSSMASVCGASLALMDAGVPIKAAVAGIAMGLVKEGDNYVVLSD
ILGDEDHLGDMDFKVAGSRDGISALQMDIKIEGITKEIMQVALNQAKGARLHILGVMEQA
INAPRGDISEFAPRIHTIKINPDKIKDVIGKGGSVIRALTEETGTTIEIEDDGTVKIAAT
DGEKAKHAIRRIEEITAEIEVGRVYTGKVTRIVDFGAFVAIGGGKEGLVHISQIADKRVE
KVTDYLQMGQEVPVKVLEVDRQGRIRLSIKEATEQSQPAAAPEAPAAEQGE
>Q6XVY3
MEDLADVICRALGIPLIDIDDQAIMLDDDVLIYIEKEGDSINLLCPFCALPENINDLIYA
LSLNYSEKICLATDDEGGNLIARLDLTGINEFEDVYVNTEYYISRVRWLKDEFARRMKGY

>P21513
MKRMLINATQQEELRVALVDGQRLYDLDIESPGHEQKKANIYKGKITRIEPSLEAAFVDY
GAERHGFLPLKEIAREYFPANYSAHGRPNIKDVLREGQEVIVQIDKEERGNKGAALTTFI
SLAGSYLVLMPNNPRAGGISRRIEGDDRTELKEALASLELPEGMGLIVRTAGVGKSAEAL
QWDLSFRLKHWEAIKKAAESRPAPFLIHQESNVIVRAFRDYLRQDIGEILIDNPKVLELA
RQHIAALGRPDFSSKIKLYTGEIPLFSHYQIESQIESAFQREVRLPSGGSIVIDSTEALT
AIDINSARATRGGDIEETAFNTNLEAADEIARQLRLRDLGGLIVIDFIDMTPVRHQRAVE
NRLREAVRQDRARIQISHISRFGLLEMSRQRLSPSLGESSHHVCPRCSGTGTVRDNESLS
LSILRLIEEEALKENTQEVHAIVPVPIASYLLNEKRSAVNAIETRQDGVRCVIVPNDQME
TPHYHVLRVRKGEETPTLSYMLPKLHEEAMALPSEEEFAERKRPEQPALATFAMPDVPPA
PTPAEPAAPVVAPAPKAAPATPAAPAQPGLLSRFFGALKALFSGGEETKPTEQPAPKAEA
KPERQQDRRKPRQNNRRDRNERRDTRSERTEGSDNREENRRNRRQAQQQTAETRESRQQA
EVTEKARTADEQQAPRRERSRRRNDDKRQAQQEAKALNVEEQSVQETEQEERVRPVQPRR
KQRQLNQKVRYEQSVAEEAVVAPVVEETVAAEPIVQEAPAPRTELVKVPLPVVAQTAPEQ
QEENNADNRDNGGMPRRSRRSPRHLRVSGQRRRRYRDERYPTQSPMPLTVACASPELASG
KVWIRYPIVRPQDVQVEEQREQEEVHVQPMVTEVPVAAAIEPVVSAPVVEEVAGVVEAPV
QVAEPQPEVVETTHPEVIAAAVTEQPQVITESDVAVAQEVAEQAEPVVEPQEETADIEEV
VETAEVVVAEPEVVAQPAAPVVAEVAAEVETVAAVEPEVTVEHNHATAPMTRAPAPEYVP
EAPRHSDWQRPTFAFEGKGAAGGHTATHHASAAPARPQPVE
>P0A1R2
MLAKRIIPCLDVRDGQVVKGVQFRNHEIIGDIVPLAKRYADEGADELVFYDITASSDGRV
VDKSWVARVAEVIDIPFCVAGGIRSIDDAAKILSFGADKISINSPALADPTLITRLADRF
GVQCIVVGIDTWFDDATGKYHVNQYTGDENRTRVTQWETLDWVQEVQQRGAGEIVLNMMN
QDGVRNGYDLTQLKKVRDVCRVPLIASGGAGTMEHFLEAFRDADVDGALAASVFHKQIIN
IGELKAYLAGQGVEIRIC
>P0A6I6
MQKRAIYPGTFDPITNGHIDIVTRATQMFDHVILAIAASPSKKPMFTLEERVALAQQATA
HLGNVEVVGFSDLMANFARNQHATVLIRGLRAVADFEYEMQLAHMNRHLMPELESVFLMP

SKEWSFISSSLVKEVARHQGDVTHFLPENVHQALMAKLA
>P26475
MSHCSCHDKPQHSLLPAAYRILSITRHTPLEWNFRVAVDFPAHWGQFVEVSLPRVGEAPI
SVSDYGDGWIDLLIRNVGKVTSALFTLKEGDNVWLRGCYGNGYPVDTLRHKPLLVVAGGT
GVAPVKGLMRYFVENPQEIGQLDMILGYKNRDCVLYKEEMATWRGKHNLVLTLDEGEADD
RYQIGRVTDRLADMTLSDIDTMQAIVVGPPIMITFTVKMLLQKGLKPEQIWVDYERRMAC
SVGKCGHCRMGEVYVCTDGPIFNYAVAQRFAD
>P55995
MTEDFPKILPLLVEEDTFLYPFMIAPIFLQNNASIKAVAYAKNNKSLVFIACQKDKLNDN
EAPYYDVGVIGSVMREANMPNGRVKLLFNGIAKGRILEPAKENEQGFLEAQISPIEYLEY
DKENIQAIVEVLKEKVITLANVSSLFPPDLIKALEDNDDPNRIADLIAAALHLKKDQAYS
LFANNNTEQRLLDLIDIVIEETKTQKLQKEIKSKVHQKMEQTNKEYFLKEQLKQIQKELG
TDKQRDEDLNQYYQKLESIKPFLKEEAFKEIKKQIDRLSRTHADSSDSATLQNYIETMLD
VPFGQYGKKALDIKHVREQLDKDHYSLKRPKERIVEYFATMQLLEMRRKKKPEKKDKTKG
TILCFYGPPGVGKTSLANSIAKAIERPLVRIALGGLEDVNELRGHRRTYIGSMPGRIVQG
LIEAKKMNPVMVLDEIDKVDRSVRGDPASALLEILDPEQNTAFRDHYANFSIDLSQVIFI
ATANNIDRIPAPLRDRMEFISVSSYTPNEKEEIAKNYLIPQELEKHALKPSEVEISHECL
KLIIEKYTREAGVRDLRRQIATIMRKVALKYLEDNPHQKGRTKKGKNEKSEDQKSEDQKS
ENQKSENKDFCVSITPNNLKEYLERMVFEIDPIDEENKIGIVNGLAWTPVGGDVLKIEVL
KIRGKGELKLTGSLGDVMKESAIIAFSVVKVLLDNETLKVPKIPSETDAEGKKKKKVLKV
YNAYDLHLHVPEGATPKDGPSAGIAMASVMASILCDRATRSEVAMTGELTLSGEVLPIGG
LKEKLIAAFKAGIKTALIPVKNYERDLDEIPAEVRENLNIVAVKNIAEVLEKTLL
>P54737
MPLKVIGPYRVLETLGSGGAGTVYRALDRRTTDEVALKLLSAGPARDARAARRLAREFDT
LVDLSHPNVVKVFESGVHQGVPYLAMELIEGLTLRHYLDLSSGDRQTPPGSHTPRSPLSV
LRTADDDFGPLSRSFSDSMDDSEDSPFDGTFGLEAFAEEAPSEDLESFASSASPHVGIGS
DDSLEGFDLPPPMPRPAEPEEEPGRVVREEDLNRPERMGRLKDAMLQICEALAYIHGHGL
VHRDLKPSNIMVDDDRQVRLMDFGLAKFLADDAAITEAGKLVGTYRYMAPEQILGEPLDG
RADLYSLGVILYELLSGRPPFDAKTPHELWRQVLETEPPPVLALNLHGDPQLARVAHRLI
RKEPDDRFQTAEEVYEALSE
>A1W0G5
MAEITAAMVKELRESTGAGMMDCKNALSETNGDFDKAVQLLREKGLGKAAKKADRLAAEG
LVSVKVSDDFTSATVSEINSETDFVAKNDQFIALTKDTTAHIQSNSLQSVEELHSSTING
VKFEEYLKSQIATIGENLVVRRFATLKAGANGVVNGYIHTNGRVGVVIAAACDSTEVASK
SRDLLRQICMHIAAMRPSYLSYEDLDMTFVENEYKALVAELEKENEERRRLKDPNKPEHK
IPQFASRKQLSDAILKEAEEKIKEELKAQGKPEKIWDNIIPGKINSFIADNSQLDSKLTL
MGQFYVMDDKKTVEQVIAEKEKEFGGKIKIVEFICFEVGEGLEKKTEDFAAEVAAQL
>P0AFK0
MALAMKVISQVEAQRKILEEAVSTALELASGKSDGAEVAVSKTTGISVSTRYGEVENVEF
NSDGALGITVYHQNRKGSASSTDLSPQAIARTVQAALDIARYTSPDPCAGVADKELLAFD
APDLDLFHPAEVSPDEAIELAARAEQAALQADKRITNTEGGSFNSHYGVKVFGNSHGMLQ
GYCSTRHSLSSCVIAEENGDMERDYAYTIGRAMSDLQTPEWVGADCARRTLSRLSPRKLS
TMKAPVIFANEVATGLFGHLVGAIAGGSVYRKSTFLLDSLGKQILPDWLTIEEHPHLLKG
LASTPFDSEGVRTERRDIIKDGILTQWLLTSYSARKLGLKSTGHAGGIHNWRIAGQGLSF

EQMLKEMGTGLVVTELMGQGVSAITGDYSRGAAGFWVENGEIQYPVSEITIAGNLKDMWR
NIVTVGNDIETRSNIQCGSVLLPEMKIAGQ
>P30013
MLPDKGWLVEARRVPSPHYDCRPDDEKPSLLVVHNISLPPGEFGGPWIDALFTGTIDPDA
HPFFAEIAHLRVSAHCLIRRDGEIVQYVPFDKRAWHAGVSNYQGRERCNDFSIGIELEGT
DTLAYTDAQYQQLAAVTRTLIASYPAIADNMTGHCNITPDRKTDPGPAFDWPRFRALVAL
SSHKEMT
>P94212
MTDRSTLDDAPAQADFIIAGATLIDGGGGPARQGDLAVRGGRIVALGDFAHAPGVPVIDA
RGLALAPGFIDSHTHDDGYLLAHPEMLPKVSQGITTVVTGNCGISLAPLSRRQIPQPLDL
LGPPELFRFATFRDWLRALAETPAAVNVIPLVGHTTLRVAVMDDTGRAATDAERAAMRAL
LDEALQAGAFGVSTGTFYPPASAAPTDEIIDVCQPLRGRAGAIYATHLRDEADHIVPAME
EALLIGRELDCRVVFSHHKLAGERNHGRSRETLDMISRAAATQRVCLDCHPYPATSTMLR
LDRARLASRTLITWSKGYPEATGRDFSEVMAELGLDDEAAIARLAPAGAIYFLMDQADVN
RIFSHPLTTVGSDGLPFDPHPHPRQWGTFTNVLRTMVREQRLLSLETAIHKMTGLAAAQY
GLTERGLLRQGYHADLVLFDPANVTDTATFSAPIQVSQGIHAVWVNGRQVWDGERTGAER
PGQVLAPGDAIPWSQQSE
>P52093
MLSKDIIKLLNEQVNKEMNSSNLYMSMSSWCYTHSLDGAGLFLFDHAAEEYEHAKKLIVF
LNENNVPVQLTSISAPEHKFEGLTQIFQKAYEHEQHISESINNIVDHAIKGKDHATFNFL
QWYVSEQHEEEVLFKDILDKIELIGNENHGLYLADQYVKGIAKSRKS
>P21244
MSSRSELLLDRFAEKIGVGSISFNENRLCSFAIDEIYYISLSDANDEYMMIYGVCGKFPT
DNPNFALEILNANLWFAENGGPYLCYESGAQSLLLALRFPLDDATPEKLENEIEVVVKSM
ENLYLVLHNQGITLENEHMKIEEISSSDNKHYYAGR
>P30139
MLRIADKTFDSHLFTGTGKFASSQLMVEAIRASGSQLVTLAMKRVDLRQHNDAILEPLIA
AGVTLLPNTSGAKTAEEAIFAAHLAREALGTNWLKLEIHPDARWLLPDPIETLKAAETLV
QQGFVVLPYCGADPVLCKRLEEVGCAAVMPLGAPIGSNQGLETRAMLEIIIQQATVPVVV
DAGIGVPSHAAQALEMGADAVLVNTAIAVADDPVNMAKAFRLAVEAGLLARQSGPGSRSY
FAHATSPLTGFLEASA
>P10902
MNTLPEHSCDVLIIGSGAAGLSLALRLADQHQVIVLSKGPVTEGSTFYAQGGIAAVFDET
DSIDSHVEDTLIAGAGICDRHAVEFVASNARSCVQWLIDQGVLFDTHIQPNGEESYHLTR
EGGHSHRRILHAADATGREVETTLVSKALNHPNIRVLERSNAVDLIVSDKIGLPGTRRVV
GAWVWNRNKETVETCHAKAVVLATGGASKVYQYTTNPDISSGDGIAMAWRAGCRVANLEF
NQFHPTALYHPQARNFLLTEALRGEGAYLKRPDGTRFMPDFDERGELAPRDIVARAIDHE
MKRLGADCMFLDISHKPADFIRQHFPMIYEKLLGLGIDLTQEPVPIVPAAHYTCGGVMVD
DHGRTDVEGLYAIGEVSYTGLHGANRMASNSLLECLVYGWSAAEDITRRMPYAHDISTLP
PWDESRVENPDERVVIQHNWHELRLFMWDYVGIVRTTKRLERALRRITMLQQEIDEYYAH
FRVSNNLLELRNLVQVAELIVRCAMMRKESRGLHFTLDYPELLTHSGPSILSPGNHYINR

>P69797
MTIAIVIGTHGWAAEQLLKTAEMLLGEQENVGWIDFVPGENAETLIEKYNAQLAKLDTTK

GVLFLVDTWGGSPFNAASRIVVDKEHYEVIAGVNIPMLVETLMARDDDPSFDELVALAVE
TGREGVKALKAKPVEKAAPAPAAAAPKAAPTPAKPMGPNDYMVIGLARIDDRLIHGQVAT
RWTKETNVSRIIVVSDEVAADTVRKTLLTQVAPPGVTAHVVDVAKMIRVYNNPKYAGERV
MLLFTNPTDVERLVEGGVKITSVNVGGMAFRQGKTQVNNAVSVDEKDIEAFKKLNARGIE
LEVRKVSTDPKLKMMDLISKIDK
>P0A6Y5
MPQHDQLHRYLFENFAVRGELVTVSETLQQILENHDYPQPVKNVLAELLVATSLLTATLK
FDGDITVQLQGDGPMNLAVINGNNNQQMRGVARVQGEIPENADLKTLVGNGYVVITITPS
EGERYQGVVGLEGDTLAACLEDYFMRSEQLPTRLFIRTGDVDGKPAAGGMLLQVMPAQNA
QQDDFDHLATLTETIKTEELLTLPANEVLWRLYHEEEVTVYDPQDVEFKCTCSRERCADA
LKTLPDEEVDSILAEDGEIDMHCDYCGNHYLFNAMDIAEIRNNASPADPQVH
>P69795
MEKKHIYLFCSAGMSTSLLVSKMRAQAEKYEVPVIIEAFPETLAGEKGQNADVVLLGPQI
AYMLPEIQRLLPNKPVEVIDSLLYGKVDGLGVLKAAVAAIKKAAAN
>Q9APM5
MTYDKAELVALDKKYVWHHLTQHKNFEPAIYVKGEGMRITDIDGKTYLDAVSGGVWTVNV
GYGRKEIVDAVAKQMMEMCYFANGIGNVPTIKFSEKLISKMPGMSRVYLSNSGSEANEKA
FKIVRQIGQLKHGGKKTGILYRARDYHGTTIGTLSACGQFERKVQYGPFAPGFYEFPDCD
VYRSKFGDCADLGVKMAKQLEEVILTVGPDELGAVIVEPMTAGGGILVPPAGYYETIREI
CDKYELLLIIDEVVCGLGRTGKWFGYQHFNVQPDIVTMAKGVASGYAPISCTVTTEKVFQ
DFVNDPADTDAYFRDISTFGGCTSGPAAALANIEIIERENLLENCTKMGDRLLEGLKGLM
AKHPIIGDVRGKGLFAGIEIVKDRATKEPIAEAVANAMVGAAKQAGVLIGKTSRSFREFN
NTLTLCPALIATEADIDEIVAGIDKAFTTVEQKFGL
>P16525
MARYDLVDRLNTTFRQMEQELAIFAAHLEQHKLLVARVFSLPEVKKEDEHNPLNRIEVKQ
HLGNDAQSLALRHFRHLFIQQQSENRSSKAAVRLPGVLCYQVDNLSQAALVSHIQHINKL
KTTFEHIVTVESELPTAARFEWVHRHLPGLITLNAYRTLTVLHDPATLRFGWANKHIIKN
LHRDEVLAQLEKSLKSPRSVAPWTREEWQRKLEREYQDIAALPQNAKLKIKRPVKVQPIA
RVWYKGDQKQVQHACPTPLIALINRDNGAGVPDVGELLNYDADNVQHRYKPQAQPLRLII
PRLHLYVAD
>P33547
MCRKLYDKLYEITGAKLDFNDKNQAFILLEEQIPVCITDNDEYIFLTGLLNEHELFTENI
INPEHILILNYSLSRDYGSSICLLPDTHQCVLTKKHYKKYLSPDELIESLYEFLFCIKLT
IANITSEVN
>O83004
MNPSTLPAPPLAEVYDVAVIGGGINGVGIAADAAGRGLSVFLCEKDDLASHTSSASSKLI
HGGLRYLEHYEFRLVREALAEREVLLAKAPHIVKQMRFVLPHRPHLRPAWMIRAGLFLYD
HLGKREKLAGSKSLKFGANSPLKSEITKGFEYSDCWVDDARLVVLNAMAAREKGAHIHTQ
TRCISAHRSNGLWEMNMERADGSLFSIRARALVNAAGPWVAKFIRDDLKLDSPYGIRLIQ
GSHLIVPRLYEGAHAHILQNEDQRIVFTIPYLNHLTIIGTTDREYTGDPAKVAITEGETD
YMLKVVNAHFKKQLSRDDIVHTYSGVRPLCNDESDNPSAITRDYTLALSGGNGEAPILSV
FGGKLTTYRKLAESAMAQLAPFFTQMRPSWTAKASLPGGENMTTPERAGRRHPRQIRLGT
ERDAPRRWATTYGSRTWRLLEGVQALADLGDHLGGGLYTREVDYLCAEEWATQPQDILWR
RTKLGLFTTAEEQDNVQRYLSKVGQTRAKIEAA

>P04744
MAHHEVISRSGNAFLLNIRESVLLPGSMSEMHFFLLIGISSIHSDRVILAMKDYLVGGHS
RKEVCEKYQMNNGYFSTTLGRLIRLNALAARLAPYYTDESSAFD
>P0A953
MKRAVITGLGIVSSIGNNQQEVLASLREGRSGITFSQELKDSGMRSHVWGNVKLDTTGLI
DRKVVRFMSDASIYAFLSMEQAIADAGLSPEAYQNNPRVGLIAGSGGGSPRFQVFGADAM
RGPRGLKAVGPYVVTKAMASGVSACLATPFKIHGVNYSISSACATSAHCIGNAVEQIQLG
KQDIVFAGGGEELCWEMACEFDAMGALSTKYNDTPEKASRTYDAHRDGFVIAGGGGMVVV
EELEHALARGAHIYAEIVGYGATSDGADMVAPSGEGAVRCMKMAMHGVDTPIDYLNSHGT
STPVGDVKELAAIREVFGDKSPAISATKAMTGHSLGAAGVQEAIYSLLMLEHGFIAPSIN
IEELDEQAAGLNIVTETTDRELTTVMSNSFGFGGTNATLVMRKLKD
>Q2GCI5
MSKINLEDLKSLSKETSAGLVHCKQALSEAHGDLTRAREILKELGHAVSVKKANRGARDG
VVGALSSGKFGVILELNCETDFVARNEKFQQFAQSVLEAACAAKVKSVEECLSTPLPGGQ
KVRDAIVEQVAVFRENIVLSRCVTYEVSQSGLLGVYVHNKYTENLGKIGVAVAVVSEADP
SFLSTVAKDIAIQVMSECPCAIDVARIPPNLLESEKHKYNLEVEGKPASVAEKIIAGKLS
KFYKKVVLLEQPLFSDPERSVKQYISDKEMESSAKIDVVWYEVFVLGEAS
>P25522
MSDNDTIVAQATPPGRGGVGILRISGFKAREVAETVLGKLPKPRYADYLPFKDADGSVLD
QGIALWFPGPNSFTGEDVLELQGHGGPVILDLLLKRILTIPGLRIARPGEFSERAFLNDK
LDLAQAEAIADLIDASSEQAARSALNSLQGAFSARVNHLVEALTHLRIYVEAAIDFPDEE
IDFLSDGKIEAQLNDVIADLDAVRAEARQGSLLREGMKVVIAGRPNAGKSSLLNALAGRE
AAIVTDIAGTTRDVLREHIHIDGMPLHIIDTAGLREASDEVERIGIERAWQEIEQADRVL
FMVDGTTTDAVDPAEIWPEFIARLPAKLPITVVRNKADITGETLGMSEVNGHALIRLSAR
TGEGVDVLRNHLKQSMGFDTNMEGGFLARRRHLQALEQAAEHLQQGKAQLLGAWAGELLA
EELRLAQQNLSEITGEFTSDDLLGRIFSSFCIGK
>O68703
MNSIHGHYHIQLSNYSAGENLQSATLTEGVIGAHRVKVETALSHSNLQKKLSATIKHNQS
GRSMLDRKLTSDGKANQRSSFTFSMIMYRMIHFVLSTRVPAVRESVANYGGNINFKFAQT
KGAFLHKIIKHSDTASGVCEALCAHWIRSHAQGQSLFDQLYVGGRKGKFQIDTLYSIKQL
QIDGCKADVDQDEVTLDWFKKNGISERMIERHCLLRPVDVTGTTESEGLDQLLNAILDTH
GIGYGYKKIHLSGQMSAHAIAAYVNEKSGVTFFDPNFGEFHFSDKEKFRKWFTNSFWGNS
MYHYPLGVGQRFRVLTFDSKEV
>P0A9I1
MISASLQQRKTRTRRSMLFVPGANAAMVSNSFIYPADALMFDLEDSVALREKDTARRMVY
HALQHPLYRDIETIVRVNALDSEWGVNDLEAVVRGGADVVRLPKTDTAQDVLDIEKEILR
IEKACGREPGSTGLLAAIESPLGITRAVEIAHASERLIGIALGAEDYVRNLRTERSPEGT
ELLFARCSILQAARSAGIQAFDTVYSDANNEAGFLQEAAHIKQLGFDGKSLINPRQIDLL
HNLYAPTQKEVDHARRVVEAAEAAAREGLGVVSLNGKMVDGPVIDRARLVLSRAELSGIR
EE
>P0C093
MAEKQTAKRNRREEILQSLALMLESSDGSQRITTAKLAASVGVSEAALYRHFPSKTRMFD
SLIEFIEDSLITRINLILKDEKDTTARLRLIVLLLLGFGERNPGLTRILTGHALMFEQDR
LQGRINQLFERIEAQLRQVLREKRMREGEGYTTDETLLASQILAFCEGMLSRFVRSEFKY

RPTDDFDARWPLIAAQLQ
>P0A7A5
MVSRRVQALLDQLRAQGIQDEQVLNALAAVPREKFVDEAFEQKAWDNIALPIGQGQTISQ
PYMVARMTELLELTPQSRVLEIGTGSGYQTAILAHLVQHVCSVERIKGLQWQARRRLKNL
DLHNVSTRHGDGWQGWQARAPFDAIIVTAAPPEIPTALMTQLDEGGILVLPVGEEHQYLK
RVRRRGGEFIIDTVEAVRFVPLVKGELA
>P0A794
MAELLLGVNIDHIATLRNARGTAYPDPVQAAFIAEQAGADGITVHLREDRRHITDRDVRI
LRQTLDTRMNLEMAVTEEMLAIAVETKPHFCCLVPEKRQEVTTEGGLDVAGQRDKMRDAC
KRLADAGIQVSLFIDADEEQIKAAAEVGAPFIEIHTGCYADAKTDAEQAQELARIAKAAT
FAASLGLKVNAGHGLTYHNVKAIAAIPEMHELNIGHAIIGRAVMTGLKDAVAEMKRLMLE
ARG
>P16659
MRTSQYLLSTLKETPADAEVISHQLMLRAGMIRKLASGLYTWLPTGVRVLKKVENIVREE
MNNAGAIEVSMPVVQPADLWQESGRWEQYGPELLRFVDRGERPFVLGPTHEEVITDLIRN
ELSSYKQLPLNFYQIQTKFRDEVRPRFGVMRSREFLMKDAYSFHTSQESLQETYDAMYAA
YSKIFSRMGLDFRAVQADTGSIGGSASHEFQVLAQSGEDDVVFSDTSDYAANIELAEAIA
PKEPRAAATQEMTLVDTPNAKTIAELVEQFNLPIEKTVKTLLVKAVEGSSFPQVALLVRG
DHELNEVKAEKLPQVASPLTFATEEEIRAVVKAGPGSLGPVNMPIPVVIDRTVAAMSDFA
AGANIDGKHYFGINWDRDVATPEVADIRNVVAGDPSPDGQGRLLIKRGIEVGHIFQLGTK
YSEALKASVQGEDGRNQILTMGCYGIGVTRVVAAAIEQNYDERGIVWPDAIAPFQVAILP
MNMHKSFRVQELAEKLYSELRAQGIEVLLDDRKERPGVMFADMELIGIPHTIVLGDRNLD
NDDIEYKYRRNGEKQLIKTGDIVEYLVKQIKG
>P0C348
MEINPILNSIKDLSERTQTIRGYLLRSEASPGRSKPRARRPQRLEQPRIRPEPRPRARPA
GAGGGNPGRPDQRPGRLPRPAGHGGRGRRRGRRRRRCQRSRASARDPREAGVPPHVQRRD
GPEQRLPGHPGRLRRHRGPGLGQHAAAHVPALGRQARLRRHYHRAVRGRGRRHQGRHRAH
QGRVRLRLAAHRDRRAPPGAQESVRLRQPSPHLVHRGVRIAGNRRQHRDRDQPGRPAHRY
LPLLRRGRSAREHHRFGGADHPRADQYRGGVPERTLPARQQGHRDEDAAGQVVRAGNAEA
YRRVPGPGGLQVRHRLGPPDPLLRARPVADQGPAYRYRAQRLRQGARRRSRRIPRSQPQT
GAV
>P18775
MKTKIPDAVLAAEVSRRGLVKTTAIGGLAMASSALTLPFSRIAHAVDSAIPTKSDEKVIW
SACTVNCGSRCPLRMHVVDGEIKYVETDNTGDDNYDGLHQVRACLRGRSMRRRVYNPDRL
KYPMKRVGARGEGKFERISWEEAYDIIATNMQRLIKEYGNESIYLNYGTGTLGGTMTRSW
PPGNTLVARLMNCCGGYLNHYGDYSSAQIAEGLNYTYGGWADGNSPSDIENSKLVVLFGN
NPGETRMSGGGVTYYLEQARQKSNARMIIIDPRYTDTGAGREDEWIPIRPGTDAALVNGL
AYVMITENLVDQAFLDKYCVGYDEKTLPASAPKNGHYKAYILGEGPDGVAKTPEWASQIT
GVPADKIIKLAREIGSTKPAFISQGWGPQRHANGEIATRAISMLAILTGNVGINGGNSGA
REGSYSLPFVRMPTLENPIQTSISMFMWTDAIERGPEMTALRDGVRGKDKLDVPIKMIWN
YAGNCLINQHSEINRTHEILQDDKKCELIVVIDCHMTSSAKYADILLPDCTASEQMDFAL
DASCGNMSYVIFNDQVIKPRFECKTIYEMTSELAKRLGVEQQFTEGRTQEEWMRHLYAQS
REAIPELPTFEEFRKQGIFKKRDPQGHHVAYKAFREDPQANPLTTPSGKIEIYSQALADI
AATWELPEGDVIDPLPIYTPGFESYQDPLNKQYPLQLTGFHYKSRVHSTYGNVDVLKAAC

RQEMWINPLDAQKRGIHNGDKVRIFNDRGEVHIEAKVTPRMMPGVVALGEGAWYDPDAKR
VDKGGCINVLTTQRPSPLAKGNPSHTNLVQVEKV
>P30850
MFQDNPLLAQLKQQLHSQTPRAEGVVKATEKGFGFLEVDAQKSYFIPPPQMKKVMHGDRI
IAVIHSEKERESAEPEELVEPFLTRFVGKVQGKNDRLAIVPDHPLLKDAIPCRAARGLNH
EFKEGDWAVAEMRRHPLKGDRSFYAELTQYITFGDDHFVPWWVTLARHNLEKEAPDGVAT
EMLDEGLVREDLTALDFVTIDSASTEDMDDALFAKALPDDKLQLIVAIADPTAWIAEGSK
LDKAAKIRAFTNYLPGFNIPMLPRELSDDLCSLRANEVRPVLACRMTLSADGTIEDNIEF
FAATIESKAKLVYDQVSDWLENTGDWQPESEAIAEQVRLLAQICQRRGEWRHNHALVFKD
RPDYRFILGEKGEVLDIVAEPRRIANRIVEEAMIAANICAARVLRDKLGFGIYNVHMGFD
PANADALAALLKTHGLHVDAEEVLTLDGFCKLRRELDAQPTGFLDSRIRRFQSFAEISTE
PGPHFGLGLEAYATWTSPIRKYGDMINHRLLKAVIKGETATRPQDEITVQMAERRRLNRM
AERDVGDWLYARFLKDKAGTDTRFAAEIVDISRGGMRVRLVDNGAIAFIPAPFLHAVRDE
LVCSQENGTVQIKGETVYKVTDVIDVTIAEVRMETRSIIARPVA
>P35636
MESPSYKNLIKAEDAQKKAGKRLLSSEWYPGFHVTPLTGWMNDPNGLIFFKGEYHLFYQY
YPFAPVWGPMHWGHAKSRDLVHWETLPVALAPGDSFDRDGCFSGCAVDNNGVLTLIYTGH
IVLSNDSLDAIREVQCMATSIDGIHFQKEGIVLEKAPMPQVAHFRDPRVWKENNHWFMVV
GYRTDDEKHQGIGHVALYRSENLKDWIFVKTLLGDNSQLPLGKRAFMWECPDFFSLGNRS
VLMFSPQGLKASGYKNRNLFQNGYILGKWQAPQFTPETSFQELDYGHDFYAAQRFEAKDG
RQILIAWFDMWENQKPSQRDGWAGCMTLPRKLDLIDNKIVMTPVREMEILRQSEKIESVV
TLSDAEHPFTMDSPLQEIELIFDLEKSSAYQAGLALRCNGKGQETLLYIDRSQNRIILDR
NRSGQNVKGIRSCPLPNTSKVRLHIFLDRSSIEIFVGDDQTQGLYSISSRIFPDKDSLKG
RLFAIEGYAVFDSFKRWTLQDANLAAFSSDAC
>O06925
MEGMLNELNFKFKSENPVDVVLPKHHYGVVGSGDLEVLLKKHELEGAVEIRVVSPVRGFD
HVWEKVLEKVISDAEVGNVAIEINDNNATPVVVALRLAQALSEAKSAEQSVN
>P15034
MSEISRQEFQRRRQALVEQMQPGSAALIFAAPEVTRSADSEYPYRQNSDFWYFTGFNEPE
AVLVLIKSDDTHNHSVLFNRVRDLTAEIWFGRRLGQDAAPEKLGVDRALAFSEINQQLYQ
LLNGLDVVYHAQGEYAYADVIVNSALEKLRKGSRQNLTAPATMIDWRPVVHEMRLFKSPE
EIAVLRRAGEITAMAHTRAMEKCRPGMFEYHLEGEIHHEFNRHGARYPSYNTIVGSGENG
CILHYTENECEMRDGDLVLIDAGCEYKGYAGDITRTFPVNGKFTQAQREIYDIVLESLET
SLRLYRPGTSILEVTGEVVRIMVSGLVKLGILKGDVDELIAQNAHRPFFMHGLSHWLGLD
VHDVGVYGQDRSRILEPGMVLTVEPGLYIAPDAEVPEQYRGIGIRIEDDIVITETGNENL
TASVVKKPEEIEALMVAARKQ
>P0A6K6
MKRAFIMVLDSFGIGATEDAERFGDVGADTLGHIAEACAKGEADNGRKGPLNLPNLTRLG
LAKAHEGSTGFIPAGMDGNAEVIGAYAWAHEMSSGKDTPSGHWEIAGVPVLFEWGYFSDH
ENSFPQELLDKLVERANLPGYLGNCHSSGTVILDQLGEEHMKTGKPIFYTSADSVFQIAC
HEETFGLDKLYELCEIAREELTNGGYNIGRVIARPFIGDKAGNFQRTGNRHDLAVEPPAP
TVLQKLVDEKHGQVVSVGKIADIYANCGITKKVKATGLDALFDATIKEMKEAGDNTIVFT
NFVDFDSSWGHRRDVAGYAAGLELFDRRLPELMSLLRDDDILILTADHGCDPTWTGTDHT
REHIPVLVYGPKVKPGSLGHRETFADIGQTLAKYFGTSDMEYGKAMF

>P0A9A9
MTDNNTALKKAGLKVTLPRLKILEVLQEPDNHHVSAEDLYKRLIDMGEEIGLATVYRVLN
QFDDAGIVTRHNFEGGKSVFELTQQHHHDHLICLDCGKVIEFSDDSIEARQREIAAKHGI
RLTNHSLYLYGHCAEGDCREDEHAHEGK
>P12295
MANELTWHDVLAEEKQQPYFLNTLQTVASERQSGVTIYPPQKDVFNAFRFTELGDVKVVI
LGQDPYHGPGQAHGLAFSVRPGIAIPPSLLNMYKELENTIPGFTRPNHGYLESWARQGVL
LLNTVLTVRAGQAHSHASLGWETFTDKVISLINQHREGVVFLLWGSHAQKKGAIIDKQRH
HVLKAPHPSPLSAHRGFFGCNHFVLANQWLEQRGETPIDWMPVLPAESE
>P33599
MVNNMTDLTAQEPAWQTRDHLDDPVIGELRNRFGPDAFTVQATRTGVPVVWIKREQLLEV
GDFLKKLPKPYVMLFDLHGMDERLRTHREGLPAADFSVFYHLISIDRNRDIMLKVALAEN
DLHVPTFTKLFPNANWYERETWDLFGITFDGHPNLRRIMMPQTWKGHPLRKDYPARATEF
SPFELTKAKQDLEMEALTFKPEEWGMKRGTENEDFMFLNLGPNHPSAHGAFRIVLQLDGE
EIVDCVPDIGYHHRGAEKMGERQSWHSYIPYTDRIEYLGGCVNEMPYVLAVEKLAGITVP
DRVNVIRVMLSELFRINSHLLYISTFIQDVGAMTPVFFAFTDRQKIYDLVEAITGFRMHP
AWFRIGGVAHDLPRGWDRLLREFLDWMPKRLASYEKAALQNTILKGRSQGVAAYGAKEAL
EWGTTGAGLRATGIDFDVRKARPYSGYENFDFEIPVGGGVSDCYTRVMLKVEELRQSLRI
LEQCLNNMPEGPFKADHPLTTPPPKERTLQHIETLITHFLQVSWGPVMPANESFQMIEAT
KGINSYYLTSDGSTMSYRTRVRTPSFAHLQQIPAAIRGSLVSDLIVYLGSIDFVMSDVDR

>P0A790
MIRTMLQGKLHRVKVTHADLHYEGSCAIDQDFLDAAGILENEAIDIWNVTNGKRFSTYAI
AAERGSRIISVNGAAAHCASVGDIVIIASFVTMPDEEARTWRPNVAYFEGDNEMKRTAKA
IPVQVA
>P0A6I0
MTAIAPVITIDGPSGAGKGTLCKAMAEALQWHLLDSGAIYRVLALAALHHHVDVASEDAL
VPLASHLDVRFVSTNGNLEVILEGEDVSGEIRTQEVANAASQVAAFPRVREALLRRQRAF
RELPGLIADGRDMGTVVFPDAPVKIFLDASSEERAHRRMLQLQEKGFSVNFERLLAEIKE
RDDRDRNRAVAPLVPAADALVLDSTTLSIEQVIEKALQYARQKLALA
>P62395
MSGILTRWRQFGKRYFWPHLLLGMVAASLGLPALSNAAEPNAPAKATTRNHEPSAKVNFG
QLALLEANTRRPNSNYSVDYWHQHAIRTVIRHLSFAMAPQTLPVAEESLPLQAQHLALLD
TLSALLTQEGTPSEKGYRIDYAHFTPQAKFSTPVWISQAQGIRAGPQRLT
>O85094
MSTEDLYQEDVEMLDDYEDPSTEQHWSEEDGEPSGYATAEPDDHAAQEEQDEPPALDSLA
LDLTLRCGELRLTLAELRRLDAGTILEVTGISPGHATLCHGEQVVAEGELVDVEGRLGLQ
ITRLVTRS
>P51064
MNIKEIPIGNNPPEDVNVIIEVPVDSQPVKYEMDKKSGSLFVDRFISTSMVYPGNYGFIP
HTLSEDSDPIDVLVCNTRPLIPGCVINVRPIGALIMEDDGGKDEKIIAVPTPKLTQQYIG
IHDYTDLTENILKKIEHFFKHYKDLEAGKWAKIEGWRDKNFARELIQQAIERAKAIQ
>O33732
MSVVQSSCAYCGVGCGVSVSSNKPNWTDVDAADLILVGDNKHPANYGHLCAKGERLLDSL

AQPNVLRYPKLRSGMPLDWDKASTLIADTFAKTIAEHGPDSVALYLSGQLLTEDYYVANK
FAKGFLKTANVDTNSRLCMSSAVSAMQRAFGEDVVPGCYDDLEQADVIVLVGANTAWTHP
VLFQRILAAIKANNAQLVVIDPLSTATAKQADLHLAIKPGADLTLFHGLLGYLADQNRVD
HAYIAAHTEGFDTVVLQAQQLSANLADLATQVGVSVTQLTQFYQLVANNKKVLTASCQGV
NQSTIGTDATNAMINCHLALGHIGQAGCGFFSLTGQPNAMGGREVGGLATQLACHMGFSQ
PEQQLLADFWKVDSIADQKGLVAVEMFDALAEGKIKAIWIMGTNPVVSLPNSEKIAQALA
DCPFVVVSEISPDSDTAKLADVLLPAQGWSEKCGTVTNSERTITRQRGFITAKGQAKPDW
WAVSQVAKKMGFDGFEFDDNASIFSEFAALSAKVKQVFPTKVFDLTGLTELSKAQYDALA
PTQWPIASATQIGQQNVRVFGLGEFATATGKAQFVTPAVVSVPQQSLPSNTLLLNTGRSR
DQWHTMTRTGHIASLRASIPEPVVHLHSSQLSALSLTEGGLVRIEAIQNQIEQYSTETAN
PDLFTHASFTMARAVVDDDIPTNMALMSMHWSAQFSLTKGVNQALDARVDPISKQPGFKC
QPVTLTPVELALQGVVFGQHYSSAHGLCWQVAQTLENGVCHHIGFTDTDDGFAYQATVHS
LKWTLTVVGQPLYIQCNMDKGLLKALKVLSHTQVNVALYQMNDFIGKPVDKQLIKQLHQQ
IKAGNSPLICACTGVTEANINDEINQQFNDQVMSDGLANISFEQALDSTQLLLGCGRQCG
SCHSEVKQCAKQSWKDALSYCESYSDIDHQPSVAEDVA
>P0A9N4
MSVIGRIHSFESCGTVDGPGIRFITFFQGCLMRCLYCHNRDTWDTHGGKEVTVEDLMKEV
VTYRHFMNASGGGVTASGGEAILQAEFVRDWFRACKKEGIHTCLDTNGFVRRYDPVIDEL
LEVTDLVMLDLKQMNDEIHQNLVGVSNHRTLEFAKYLANKNVKVWIRYVVVPGWSDDDDS
AHRLGEFTRDMGNVEKIELLPYHELGKHKWVAMGEEYKLDGVKPPKKETMERVKGILEQY
GHKVMF
>P04994
MLPSQSPAIFTVSRLNQTVRLLLEHEMGQVWISGEISNFTQPASGHWYFTLKDDTAQVRC
AMFRNSNRRVTFRPQHGQQVLVRANITLYEPRGDYQIIVESMQPAGEGLLQQKYEQLKAK
LQAEGLFDQQYKKPLPSPAHCVGVITSKTGAALHDILHVLKRRDPSLPVIIYPAAVQGDD
APGQIVRAIELANQRNECDVLIVGRGGGSLEDLWSFNDERVARAIFTSRIPVVSAVGHET
DVTIADFVADLRAPTPSAAAEVVSRNQQELLRQVQSTRQRLEMAMDYYLANRTRRFTQIH
HRLQQQHPQLRLARQQTMLERLQKRMSFALENQLKRTGQQQQRLTQRLNQQNPQPKIHRA
QTRIQQLEYRLAETLRAQLSATRERFGNAVTHLEAVSPLSTLARGYSVTTATDGNVLKKV
KQVKAGEMLTTRLEDGWIESEVKNIQPVKKSRKKVH
>A6Q6H4
MAKEKFERTKPHINIGTIGHVDHGKTTLTAAITAVLAVAGDTELMDYDAIDNAPEERERG
ITIATSHVEYETATRHYAHVDCPGHADYVKNMITGAAQMDGAILVIAATDGPMAQTREHI
LLSKQVGVPYIVVFLNKEDQLDDEDKEEMLELVEMEVRELLSEYDFPGDDTPIVAGSAFQ
ALEEAKTGTLGEWSAKIMELMDAVDEYIPEPKRETDKDFLMAIEDIFTIQGRGTVVTGKV
DRGQVCVGDEVEIVGLKDTQKTTVTGVEMFRKEMDCGIAGDNCGVLIRGIDKEAVQRGMV
LCKPGSITPHTQFEAEVYVLTKEEGGRHTPFFDNYRPQFYVRTTDVTGSVKLQEGTEMVM
PGDNVKINVELIAPIALDEGTRFAIREGGRTVGAGVVSKIIA
>Q7BU69
MQTSNITNHERNDSSWMSTVKSTTEVSWNKLSFCDILLKIITFGIYSPHETLAEKHSEKK
LMDSFSPSLSQDKMDGEFAHANIDGISIRLCLNKGICSVFYLDGDKIQSTQLSSKEYNNL
LSSLPPKQFNLGKVHTITAPVSGNFKTHKPAPEVIETAINCCTSIIPNDDYFHVKDTDFN
SVWHDIYRDIRASDSNSTKIYFNNIEIPLKLIADLINELGINEFIDSKKELQMLSYNQVN
KIINSNFPQQDLCFQTEKLLFTSLFQDPAFISALTSAFWQSLHITSSSVEHIYAQIMSEN

IENRLNFMPEQRVINNCGHIIKINAVVPKNDTAISASGGRAYEVSSSILPSHITCNGVGI
NKIETSYLVHAGTLPSSEGLRNAIPPESRQVSFAIISPDV
>P0C0L2
MTIHKKGQAHWEGDIKRGKGTVSTESGVLNQQPYGFNTRFEGEKGTNPEELIGAAHAACF
SMALSLMLGEAGFTPTSIDTTADVSLDKVDAGFAITKIALKSEVAVPGIDASTFDGIIQK
AKAGCPVSQVLKAEITLDYQLKS
>Q51480
MNLRPLAPLLLTLLAGCSQQPPLRGSGDLGVLIERADGSVQILDGTAKTSLARVEGLGDL
SHASLVFSRDQRYAYVFGRDGGLTKLDLLAQRIDKRLIQGGNSIGGAISQDGRLVAVSNY
EPGGVKVFDSRTLELVAEIPATRLPGQDRNSRVVGLVDAPGQRFVFSLFDSGEIWIADFS
QGDTPHLTRFRDIGKQPYDALISPDGRYYMAGLFGEDGMAQLDLWHPERGVRRVLGDYGR
GQRKLPVYKMPHLEGWTIASDQAFVPAVGHHQVLVLDARDWKQTDAIDVAGQPVFVMTRP
DDRQIWVNFAYPDNDKVQVIDSETHEVIETLRPGPGVLHMEFSGRGDQVWISVRDADQLQ
VWDPYRLKRIGSLPARSPSGIFFSHRAQHIGL
>P07639
MERIVVTLGERSYPITIASGLFNEPASFLPLKSGEQVMLVTNETLAPLYLDKVRGVLEQA
GVNVDSVILPDGEQYKSLAVLDTVFTALLQKPHGRDTTLVALGGGVVGDLTGFAAASYQR
GVRFIQVPTTLLSQVDSSVGGKTAVNHPLGKNMIGAFYQPASVVVDLDCLKTLPPRELAS
GLAEVIKYGIILDGAFFNWLEENLDALLRLDGPAMAYCIRRCCELKAEVVAADERETGLR
ALLNLGHTFGHAIEAEMGYGNWLHGEAVAAGMVMAARTSERLGQFSSAETQRIITLLKRA
GLPVNGPREMSAQAYLPHMLRDKKVLAGEMRLILPLAIGKSEVRSGVSHELVLNAIADCQ
SA
>P52197
MDDFASLPLVIEPADLQARLSAPELILVDLTSAARYAEGHIPGARFVDPKRTQLGQPPAP
GLQPPREQLESLFGELGHRPEAVYVVYDDEGGGWAGRFIWLLDVIGQQRYHYLNGGLTAW
LAEDRPLSRELPAPAGGPVALSLHDEPTASRDYLLGRLGAADLAIWDARSPQEYRGEKVL
AAKGGHIPGAVNFEWTAAMDPSRALRIRTDIAGRLEELGITPDKEIVTHCQTHHRSGLTY
LIAKALGYPRVKGYAGSWGEWGNHPDTPVEL
>P26311
MDKLLERFLHYVSLDTQSKSGVRQVPSTEGQWKLLRLLKQQLEEMGLVNITLSEKGTLMA
TLPANVEGDIPAIGFISHVDTSPDFSGKNVNPQIVENYRGGDIALGIGDEVLSPVMFPVL
HQLLGQTLITTDGKTLLGADDKAGVAEIMTALAVLKGNPIPHGDIKVAFTPDEEVGKGAK
HFDVEAFGAQWAYTVDGGGVGELEFENFNAASVNIKIVGNNVHPGTAKGVMVNALSLAAR
IHAEVPADEAPETTEGYEGFYHLASMKGTVDRAEMHYIIRDFDRKQFEARKRKMMEIAKK
VGKGLHPDCYIELVIEDSYYNMREKVVEHPHILDIAQQAMRDCHITPEMKPIRGGTDGAQ
LSFMGLPCPNLFTGGYNYHGKHEFVTLEGMEKAVQVIVRIAELTAKRGQ
>P32199
MSNSNIHTTAVIAEGAKLGKNVKIGPYCIIGPEVVLNDNVELKSHVVIEGITEIGENTVI
YPFASIGQPPQILKYANERSSTIIGSNNTIREYVTVQAGSQGGGMMTRVGNNNLFMVGVH
IGHDCKIGNNVVFANYVSLAGHIGVGDYAIIGGLSAVHQYARIGEYSMIGGLSPVGADVI
PFGLVSSKRAVLEGLNLIGMNRKGFDKVKSLSALKAIEEIFSGEGNFAERIKQVAEKYNN
NSIVIQIIDFLNQDSSRAFCRFEK
>P33602
MATIHVDGKEYEVNGADNLLEACLSLGLDIPYFCWHPALGSVGACRQCAVKQYQNAEDTR

GRLVMSCMTPASDGTFISIDDEEAKQFRESVVEWLMTNHPHDCPVCEEGGNCHLQDMTVM
TGHSFRRYRFTKRTHRNQDLGPFISHEMNRCIACYRCVRYYKDYADGTDLGVYGAHDNVY
FGRPEDGTLESEFSGNLVEICPTGVFTDKTHSERYNRKWDMQFAPSICQQCSIGCNISPG
ERYGELRRIENRYNGTVNHYFLCDRGRFGYGYVNLKDRPRQPVQRRGDDFITLNAEQAMQ
GAADILRQSKKVIGIGSPRASVESNFALRELVGEENFYTGIAHGEQERLQLALKVLREGG
IYTPALREIESYDAVLVLGEDVTQTGARVALAVRQAVKGKAREMAAAQKVADWQIAAILN
IGQRAKHPLFVTNVDDTRLDDIAAWTYRAPVEDQARLGFAIAHALDNSAPAVDGIEPELQ
SKIDVIVQALAGAKKPLIISGTNAGSLEVIQAAANVAKALKGRGADVGITMIARSVNSMG
LGIMGGGSLEEALTELETGRADAVVVLENDLHRHASAIRVNAALAKAPLVMVVDHQRTAI
MENAHLVLSAASFAESDGTVINNEGRAQRFFQVYDPAYYDSKTVMLESWRWLHSLHSTLL
SREVDWTQLDHVIDAVVAKIPELAGIKDAAPDATFRIRGQKLAREPHRYSGRTAMRANIS
VHEPRQPQDIDTMFTFSMEGNNQPTAHRSQVPFAWAPGWNSPQAWNKFQDEVGGKLRFGD
PGVRLFETSENGLDYFTSVPARFQPQDGKWRIAPYYHLFGSDELSQRAPVFQSRMPQPYI
KLNPADAAKLGVNAGTRVSFSYDGNTVTLPVEIAEGLTAGQVGLPMGMSGIAPVLAGAHL
EDLKEAQQ
>P60546
MAQGTLYIVSAPSGAGKSSLIQALLKTQPLYDTQVSVSHTTRQPRPGEVHGEHYFFVNHD
EFKEMISRDAFLEHAEVFGNYYGTSREAIEQVLATGVDVFLDIDWQGAQQIRQKMPHARS
IFILPPSKIELDRRLRGRGQDSEEVIAKRMAQAVAEMSHYAEYDYLIVNDDFDTALTDLK
TIIRAERLRMSRQKQRHDALISKLLAD
>P0A9G2
MSMSHINYNHLYYFWHVYKEGSVVGAAEALYLTPQTITGQIRALEERLQGKLFKRKGRGL
EPSELGELVYRYADKMFTLSQEMLDIVNYRKESNLLFDVGVADALSKRLVSSVLNAAVVE
GEPIHLRCFESTHEMLLEQLSQHKLDMIISDCPIDSTQQEGLFSVRIGECGVSFWCTNPP
PEKPFPACLEERRLLIPGRRSMLGRKLLNWFNSQGLNVEILGEFDDAALMKAFGAMHNAI
FVAPTLYAYDFYADKTVVEIGRVENVMEEYHAIFAERMIQHPAVQRICNTDYSALFSPAV
R
>P0A6L4
MATNLRGVMAALLTPFDQQQALDKASLRRLVQFNIQQGIDGLYVGGSTGEAFVQSLSERE
QVLEIVAEEAKGKIKLIAHVGCVSTAESQQLAASAKRYGFDAVSAVTPFYYPFSFEEHCD
HYRAIIDSADGLPMVVYNIPALSGVKLTLDQINTLVTLPGVGALKQTSGDLYQMEQIRRE
HPDLVLYNGYDEIFASGLLAGADGGIGSTYNIMGWRYQGIVKALKEGDIQTAQKLQTECN
KVIDLLIKTGVFRGLKTVLHYMDVVSVPLCRKPFGPVDEKYLPELKALAQQLMQERG
>P32169
MQNITQSWFVQGMIKATTDAWLKGWDERNGGNLTLRLDDADIAPYHDNFHQQPRYIPLSQ
PMPLLANTPFIVTGSGKFFRNVQLDPAANLGIVKVDSDGAGYHILWGLTNEAVPTSELPA
HFLSHCERIKATNGKDRVIMHCHATNLIALTYVLENDTAVFTRQLWEGSTECLVVFPDGV
GILPWMVPGTDEIGQATAQEMQKHSLVLWPFHGVFGSGPTLDETFGLIDTAEKSAQVLVK
VYSMGGMKQTISREELIALGKRFGVTPLASALAL
>P0A717
MPDMKLFAGNATPELAQRIANRLYTSLGDAAVGRFSDGEVSVQINENVRGGDIFIIQSTC
APTNDNLMELVVMVDALRRASAGRITAVIPYFGYARQDRRVRSARVPITAKVVADFLSSV
GVDRVLTVDLHAEQIQGFFDVPVDNVFGSPILLEDMLQLNLDNPIVVSPDIGGVVRARAI
AKLLNDTDMAIIDKRRPRANVSQVMHIIGDVAGRDCVLVDDMIDTGGTLCKAAEALKERG

AKRVFAYATHPIFSGNAANNLRNSVIDEVVVCDTIPLSDEIKSLPNVRTLTLSGMLAEAI
RRISNEESISAMFEH
>P60390
MMENYKHTTVLLDEAVNGLNIRPDGIYIDGTFGRGGHSRLILSQLGEEGRLLAIDRDPQA
IAVAKTIDDPRFSIIHGPFSALGEYVAERDLIGKIDGILLDLGVSSPQLDDAERGFSFMR
DGPLDMRMDPTRGQSAAEWLQTAEEADIAWVLKTYGEERFAKRIARAIVERNREQPMTRT
KELAEVVAAATPVKDKFKHPATRTFQAVRIWVNSELEEIEQALKSSLNVLAPGGRLSIIS
FHSLEDRIVKRFMRENSRGPQVPAGLPMTEEQLKKLGGRQLRALGKLMPGEEEVAENPRA
RSSVLRIAERTNA
>Q07566
MHITNLGLHQVSFQSGDSYKGAEETGKHKGVSVISYQRVKNGERNKGIEALNRLYLQNQT
SLTGKSLLFARDKAEVFCEAIKLAGGDTSKIKAMMERLDTYKLGEVNKRHINELNKVISE
EIRAQLGIKNKKELQTKIKQIFTDYLNNKNWGPVNKNISHHGKNYSFQLTPASHMKIGNK
NIFVKEYNGKGICCASTRERDHIANMWLSKVVDDEGKEIFSGIRHGVISAYGLKKNSSER
AVAARNKAEELVSAALYSRPELLSQALSGKTVDLKIVSTSLLTPTSLTGGEESMLKDQVS
ALKGLNSKRGGPTKLLIRNSDGLLKEVSVNLKVVTFNFGVNELALKMGLGWRNVDKLNDE
SICSLLGDNFLKNGVIGGWAAEAIEKNPPCKNDVIYLANQIKEIVNNKLQKNDNGEPYKL
SQRVTLLAYTIGAVPCWNCKSGKDRTGMQDAEIKREIIRKHETGQFSQLNSKLSSEEKRL
FSTILMNSGNMEIQEMNTGVPGNKVMKKLPLSSLELSYSERIGDPKIWNMVKGYSSFV
>P43500
MSLDTPNEKPAGKARARKAPASKAGATNAASTSSSTKAITDTLLTVLSGNLQARVPKELV
GESGVELAHLLNQVLDQFAASEHRKHVAAQEIDQALDALIGLVREGDLSRWNTTTEDPQL
GPLLEGFGKVIETLRTFVREINEAALRLSSSANQVLAASTQHETSSTEQAAAIHETTATM
EELKHASAQIAENAGSVARVAEETLGAARAGRGAIGEFIQAMQQIRSDGVAVADSIAKLS
KRVERIGTVVEVIDEIADRSDLLALNAALEGSRAGEAGKGFSIVAAEMRRLAENVLDSTK
EIKNLITEIREATAAAAGAAEASKSATESGEKLGAVAAQAVEGILAGVQETSDAARVINL
ATQQQRTATEQVVASMAEIEDVTRQTTQASKQATGAAAELTQLAGRLAELIKRFKAD
>P0A6V8
MTKYALVGDVGGTNARLALCDIASGEISQAKTYSGLDYPSLEAVIRVYLEEHKVEVKDGC
IAIACPITGDWVAMTNHTWAFSIAEMKKNLGFSHLEIINDFTAVSMAIPMLKKEHLIQFG
GAEPVEGKPIAVYGAGTGLGVAHLVHVDKRWVSLPGEGGHVDFAPNSEEEAIILEILRAE
IGHVSAERVLSGPGLVNLYRAIVKADNRLPENLKPKDITERALADSCTDCRRALSLFCVI
MGRFGGNLALNLGTFGGVFIAGGIVPRFLEFFKASGFRAAFEDKGRFKEYVHDIPVYLIV
HDNPGLLGSGAHLRQTLGHIL
>Q1GND0
MRNSFLFTSESVSEGHPDKVADQISDSIVDLFLAKDPEARVACETLTTTQLVVLAGEIRC
KGVFEDGEWAPGALDEIEATVRRTVREIGYEQAGFHWNRFRFENNLHGQSPQIAQGVDEG
AGKDEGAGDQGIMFGYASDETPDFMPATLDYSHKILERMASDRKAGIAPFLEPDAKSQVT
LRYANERPVEATAIVVSTQHAPGYYFHNGEGDEAKYTELRKYVLGVIADVLPAELLTANT
VYHINPTGRFEIGGPDGDAGLTGRKIIVDTYGGASPHGGGAFSGKDPTKVDRSAAYITRY
LAKNIVAAGLARRCTIQLSYAIGVAEPLSIYVDLHGTGTVDEGRIEAVLPQLVRLTPKGI
RTHLGLNKPIYRQTAAYGHFGRQADGDAFPWERTDLVDKLKAALAV
>P0A6L0
MTDLKASSLRALKLMDLTTLNDDDTDEKVIALCHQAKTPVGNTAAICIYPRFIPIARKTL

KEQGTPEIRIATVTNFPHGNDDIDIALAETRAAIAYGADEVDVVFPYRALMAGNEQVGFD
LVKACKEACAAANVLLKVIIETGELKDEALIRKASEISIKAGADFIKTSTGKVAVNATPE
SARIMMEVIRDMGVEKTVGFKPAGGVRTAEDAQKYLAIADELFGADWADARHYRFGASSL
LASLLKALGHGDGKSASSY
>P0A8F8
MSKPFKLNSAFKPSGDQPEAIRRLEEGLEDGLAHQTLLGVTGSGKTFTIANVIADLQRPT
MVLAPNKTLAAQLYGEMKEFFPENAVEYFVSYYDYYQPEAYVPSSDTFIEKDASVNEHIE
QMRLSATKAMLERRDVVVVASVSAIYGLGDPDLYLKMMLHLTVGMIIDQRAILRRLAELQ
YARNDQAFQRGTFRVRGEVIDIFPAESDDIALRVELFDEEVERLSLFDPLTGQIVSTIPR
FTIYPKTHYVTPRERIVQAMEEIKEELAARRKVLLENNKLLEEQRLTQRTQFDLEMMNEL
GYCSGIENYSRFLSGRGPGEPPPTLFDYLPADGLLVVDESHVTIPQIGGMYRGDRARKET
LVEYGFRLPSALDNRPLKFEEFEALAPQTIYVSATPGNYELEKSGGDVVDQVVRPTGLLD
PIIEVRPVATQVDDLLSEIRQRAAINERVLVTTLTKRMAEDLTEYLEEHGERVRYLHSDI
DTVERMEIIRDLRLGEFDVLVGINLLREGLDMPEVSLVAILDADKEGFLRSERSLIQTIG
RAARNVNGKAILYGDKITPSMAKAIGETERRREKQQKYNEEHGITPQGLNKKVVDILALG
QNIAKTKAKGRGKSRPIVEPDNVPMDMSPKALQQKIHELEGLMMQHAQNLEFEEAAQIRD
QLHQLRELFIAAS
>P0ABT2
MSTAKLVKSKATNLLYTRNDVSDSEKKATVELLNRQVIQFIDLSLITKQAHWNMRGANFI
AVHEMLDGFRTALIDHLDTMAERAVQLGGVALGTTQVINSKTPLKSYPLDIHNVQDHLKE
LADRYAIVANDVRKAIGEAKDDDTADILTAASRDLDKFLWFIESNIE
>P0A9L8
MEKKIGFIGCGNMGKAILGGLIASGQVLPGQIWVYTPSPDKVAALHDQFGINAAESAQEV
AQIADIIFAAVKPGIMIKVLSEITSSLNKDSLVVSIAAGVTLDQLARALGHDRKIIRAMP
NTPALVNAGMTSVTPNALVTPEDTADVLNIFRCFGEAEVIAEPMIHPVVGVSGSSPAYVF
MFIEAMADAAVLGGMPRAQAYKFAAQAVMGSAKMVLETGEHPGALKDMVCSPGGTTIEAV
RVLEEKGFRAAVIEAMTKCMEKSEKLSKS
>P25080
MTDNNKYRDVEIRAPRGNKLTAKSWLTEAPLRMLMNNLDPQVAENPKELVVYGGIGRAAR
NWECYDKIVETLTRLEDDETLLVQSGKPVGVFKTHSNAPRVLIANSNLVPHWANWEHFNE
LDAKGLAMYGQMTAGSWIYIGSQGIVQGTYETFVEAGRQHYGGTVKAKWVLTAGLGGMGG
AQPLAATLAGACSLNIECQQSRIDFRLETRYVDEQATDLDDALVRIAKYTAEGKAISIAL
HGNAAEILPELVKRGVRPDMVTDQTSAHDPLNGYLPAGWTWEQYRDRAQTEPAAVVKAAK
QSMAVHVQAMLDFQKQGVPTFDYGNNIRQMAKEEGVANAFDFPGFVPAYIRPLFCRGVGP
FRWAALSGEAEDIYKTDAKVKELIPDDAHLHRWLDMARERISFQGLPARICWVGLGLRAK
LGLAFNEMVRSGELSAPVVIGRDHLDSGSVSSPNRETEAMRDGSDAVSDWPLLNALLNTA
GGATWVSLHHGGGVGMGFSQHSGMVIVCDGTDEAAERIARVLTNDPGTGVMRHADAGYDI
AIDCAKEQGLDLPMITG
>P76407
MAEFPASLLILNGKSTDNLPLREAIMLLREEGMTIHVRVTWEKGDAARYVEEARKFGVAT
VIAGGGDGTINEVSTALIQCEGDDIPALGILPLGTANDFATSVGIPEALDKALKLAIAGD
AIAIDMAQVNKQTCFINMATGGFGTRITTETPEKLKAALGSVSYIIHGLMRMDTLQPDRC
EIRGENFHWQGDALVIGIGNGRQAGGGQQLCPNALINDGLLQLRIFTGDEILPALVSTLK
SDEDNPNIIEGASSWFDIQAPHDITFNLDGEPLSGQNFHIEILPAALRCRLPPDCPLLR

>Q9S449
MADQKQRVTVIGGGLAGTECAYQLSRRGVPVVLREMKPQKRSPAHKSDTLAELVCSNSLR
SDNPESAIGLLHAELRALGSLVLSAADANRVPAGDALAVERERFSAAITESLLRQPGVEL
VAGEVEQLPEDGPVVIATGPLTSDALTRELERHVGTRLYFYDSIAPILSADSIDMNVAFR
QSRYGKGGGDDYLNLPMTKDEYYRFIAEVKAGQKVVPHAFEEPKYFEGCLPIEVMAERGD
DTLAYGPMKPVGLRDPRTGQEPYAVVQLRMEDVGGTSWNMVGFQTRLTWGEQKRIFSSFI
PGLQQAEFLRMGQIHRNTFIDSPRLLAKDLSLKTEPRLYFAGQISGVEGYVESAACGYLV
ALALHARLTGTEFVPPPATTAMGALLRHVTGEAHPPDYPHQPSNISFGIFSPLTGRMKKA
EKRAAYSARAKQDLAAWLPHAGVPAAGAPEHVDQRSA
>Q56415
MLQSLNHLTLAVSDLQKSVTFWHELLGLTLHARWNTGAYLTCGDLWVCLSYDEARQYVPP
QESDYTHYAFTVAEEDFEPLSQRLEQAGVTIWKQNKSEGASFYFLDPDGHKLELHVGSLA
ARLAACREKPYAGMVFTSDEA
>P27859
MFDIGVNLTSSQFAKDRDDVVACAFDAGVNGLLITGTNLRESQQAQKLARQYSSCWSTAG
VHPHDSSQWQAATEEAIIELAAQPEVVAIGECGLDFNRNFSTPEEQERAFVAQLRIAADL
NMPVFMHCRDAHERFMTLLEPWLDKLPGAVLHCFTGTREEMQACVAHGIYIGITGWVCDE
RRGLELRELLPLIPAEKLLIETDAPYLLPRDLTPKPSSRRNEPAHLPHILQRIAHWRGED
AAWLAATTDANVKTLFGIAF
>P61417
MNITLTKRQQEFLLLNGWLQLQCGHAERACILLDALLTLNPEHLAGRRCRLVALLNNNQG
ERAEKEAQWLISHDPLQAGNWLCLSRAQQLNGDLDKARHAYQHYLELKDHNESP
>Q6MNT9
MKIKHLEDLNTLVSLSKRRGFVFQSSEIYGGLGSCWDYGPLGSLMKLNVKRAWWNAMTRR
PDIVGLDAAILMHPMVWKASGHVDGFSDPLVDCKECKTRFRADNTDSYINEKKCPNCGSK
NLSEERSFNLMFKTHMGPLEDSGSVVYLRPETAQGHFVNFQNCQQASRYKIPFGIAAIGK
SFRNEITPGNFIFRTREFEQMEMQYFVEPGTDEQFFKEWKERRWNFYIKFGIKPENLKFK
DHDKLAHYAKAAVDVEFKFPMGFSELEGIHNRSDFDLSQHMKFSGKNLEYFDEPNKKKYI
PYVIETAVGCDRLFLAFLCDAYREEVTTDEAGKEDVRVVMGLHPEIAPFKVAVLPLSKKE
ELSSISEKLRDQLAEDFDVNYDESQSIGKRYRRQDEIGTPFCVTVDFDTINDQAVTVRHR
DKMTQERVAITQLNAYIAAKLKTFNQ
>P24404
MTVRSLFLLPGDGIGPEAMTEVRKLIEYMNSAHNAGFTVSEGLVGGSAYDAHGVAISDAD
MEKALAADAILFGAVGGPKWDGVPYEHRPEAGLLRLRKDLELFANLRPAICYPALAAASS
LKPELVEGLDILIVRELTGGVYFGEPKQIIDLGNGQKRGIDTQIYDTFEIERIASVAFEL
ARSRDNRVCSMEKRNVMKSGVLWNQVVTETHAAKYKDVQLEHMLADAGGMQLVRKPKQFD
VIVTDNLFGDMLSDVAAMLTGSLGMLPSASLGAPDAKTGKRKAMYEPVHGSAPDIAGKSI
ANPIAMIASFAMCLRYSFNMVDEATKLEAAIANVLDKGIRTADIMADGCRQVGTSDMGDA
VLAEFKALSA
>P0A229
MSQWQNICKIDDILPGTGVCALSGGEQVAIFRPYHSDQVFAISNIDPFFEASVLSRGLIA
EHQGELWVASPLKKQRFRLSDGLCMEDEQFSVKHYDARVKDGVVQLRG
>P0A6H5
MSEMTPREIVSELDKHIIGQDNAKRSVAIALRNRWRRMQLNEELRHEVTPKNILMIGPTG

VGKTEIARRLAKLANAPFIKVEATKFTEVGYVGKEVDSIIRDLTDAAVKMVRVQAIEKNR
YRAEELAEERILDVLIPPAKNNWGQTEQQQEPSAARQAFRKKLREGQLDDKEIEIDLAAA
PMGVEIMAPPGMEEMTSQLQSMFQNLGGQKQKARKLKIKDAMKLLIEEEAAKLVNPEELK
QDAIDAVEQHGIVFIDEIDKICKRGESSGPDVSREGVQRDLLPLVEGCTVSTKHGMVKTD
HILFIASGAFQIAKPSDLIPELQGRLPIRVELQALTTSDFERILTEPNASITVQYKALMA
TEGVNIEFTDSGIKRIAEAAWQVNESTENIGARRLHTVLERLMEEISYDASDLSGQNITI
DADYVSKHLDALVADEDLSRFIL
>P36936
MELLLLSNSTLPGKAWLEHALPLIANQLNGRRSAVFIPFAGVTQTWDEYTDKTAEVLAPL
GVNVTGIHRVADPLAAIEKAEIIIVGGGNTFQLLKESRERGLLAPMADRVKRGALYIGWS
AGANLACPTIRTTNDMPIVDPNGFDALDLFPLQINPHFTNALPEGHKGETREQRIRELLV
VAPELTVIGLPEGNWIQVSNGQAVLGGPNTTWVFKAGEEAVALEAGHRF
>Q5GTF2
MAERANDIRPGQVLEHNGGLFLVVSIMHTQPGKGGAYIQAEMKNIKTGAKLYERFRSDAT
IRRAILDEEEYIYLFTEGNIVNLMHPSNYEQITINLDLLEEKKIYLQDNMRIKVVTYQDK
IIFAHVPDYVRLTVKETESFIKGQTITSSYKPAVLENGMRINVPQFIKEEDKIVVYTPDD
SYYERVKE
>O86262
MKADFWLQRWSAGQIGFHQSEVNKDLQQYWSSLNVVPGARVLVPLCGKSQDMSWLSGQGY
HVVGAELSEAAVERYFTERGEQPHITSQGDFKVYAAPGIEIWCGDFFALTARDIGHCAAF
YDRAAMIALPADMRERYVQHLEALMPQACSGLLITLEYDQALLEGPPFSVPQTWLHRVMS
GNWEVTKVGGQDTLHSSARGLKAGLERMDEHVYVLERV
>P24171
MTTMNPFLVQSTLPYLAPHFDQIANHHYRPAFDEGMQQKRAEIAAIALNPQMPDFNNTIL
ALEQSGELLTRVTSVFFAMTAAHTNDELQRLDEQFSAELAELANDIYLNGELFARVDAVW
QRRESLGLDSESIRLVEVIHQRFVLAGAKLAQADKAKLKVLNTEAATLTSQFNQRLLAAN
KSGGLVVNDIAQLAGMSEQEIALAAEAAREKGLDNKWLIPLLNTTQQPALAEMRDRATRE
KLFIAGWTRAEKNDANDTRAIIQRLVEIRAQQATLLGFPHYAAWKIADQMAKTPEAALNF
MREIVPAARQRASDELASIQAVIDKQQGGFSAQPWDWAFYAEQVRREKFDLDEAQLKPYF
ELNTVLNEGVFWTANQLFGIKFVERFDIPVYHPDVRVWEIFDHNGVGLALFYGDFFARDS
KSGGAWMGNFVEQSTLNKTHPVIYNVCNYQKPAAGEPALLLWDDVITLFHEFGHTLHGLF
ARQRYATLSGTNTPRDFVEFPSQINEHWATHPQVFARYARHYQSGAAMPDELQQKMRNAS
LFNKGYEMSELLSAALLDMRWHCLEENEAMQDVDDFELRALVAENMDLPAIPPRYRSSYF
AHIFGGGYAAGYYAYLWTQMLADDGYQWFVEQGGLTRENGLRFREAILSRGNSEDLERLY
RQWRGKAPKIMPMLQHRGLNI
>Q1D823
MERRVLIVESEHDFALSMATVLKGAGYQTALAETAADAQRELEKRRPDLVVLRAELKDQS
GFVLCGNIKKGKWGQNLKVLLLSSESGVDGLAQHRQTPQAADGYLAIPFEMGELAALSHG
IVPPGTDDTGASLDAALNGTREAPPPMPPSLKAAAGGPPKLPKRERRSAMTEEDRAFLDR
TFQSIADRKAELLAESRQLKRPPPRRELMGTPEGKIQILRDELKTREAQLARLSEIWNVR
ERELLSGEDRIHEKDVELQGLKMQVDDLLRRFNEAQQATIQKEREHGATVDDLLLQKFSA
EKDLIEVVASKEKDINLLRREVSRAEEELSRRAGELEHGRNEYDKLEKHLGVVTLEFEVK
EQKLQDTVLANEGEIARLTKRGDDFEAELNRTISERDQRFAELDGEIQALQERLQQTEQE
RDTTVRGLEARAARAEEHGTQADAEIHRLNAERDALEAKLSQQVADLEADLARTMGERDQ

LRLDKDAQEAELTQRIEERDAKLGTLERELSETIARNEHTEAELNANIQQQLERIGELEG
EVEAVKTHLEDRENELTAELQALGQAKDELETDLNDRLQALSQAKDALEADLSRQLEELR
SAKAELEADLTGQIQALTSQLEETQRQLDDSQRTGEQLSARVAQLEDTVSQRESTIESLQ
GDVAARDQRISELSGDLEATSQTLAQTQQTLAQTEQQLADTQNTLASTEGALAETRGELD
ATSQTLQQTQQTLAQTEGALAETRGELDATSQTLAQTQQTLAQTEQQLADTQNTLASTEG
TLAETRGELEATSQTLQQTHAALEDTRGALQETSDTLAHTTRERDQRIAELADLGAAKDA
LEQELTGQIGHLRSELSETQGNYEAERAAHEKLAAESSAHIGDLTSERDGLRSELEATSQ
TLEQTHGQLAATRDALAREQHAHQESRKAAASTQTTLEGQLAEARAHGEDLGEHLTLTKH
ELGTRVAELTQLTATLAQTENTRAHLEERLHTLTEESQRREELLQNDLTQKGTELSDTLR
KLTHVTQEKMRQAEVLNREVATRTEQLKAMEAKLQTQATEARRQAEGLGQQITGLNEQLE
QGRKALAGREDQLRAAGAAQQKLTAERDGLAGQLQQAEARLQQQAQQANQERADAKRAAD
ELAAKLAKTEQRITQFAQDAQTQATEADARAKDLQGQLSARAKKIQDLELAVENAQGAKS
RAEKELNAKVAAAESKAHEASTRLAAAQKERKDLEARHAKEQEDLAAKQKAELERRDAIK
AQEVARLQQSVQEKSKALKVAELELARYKSKSATTATPAKAAAKPAAAEDDELAVRTQLN
QVIAPAAAAQAPAPAKKPAAKPAAQAPAKKAPAPAPAPPAALSDESEPTDRTLVIQLPTA
KEDDDWTALVDELDK

>P69791

MMDLDNIPDTQTEAEELEEVVMGLIINSGQARSLAYAALKQAKQGDFAAAKAMMDQSRMA
LNEAHLVQTKLIEGDAGEGKMKVSLVLVHAQDHLMTSMLARELITELIELHEKLKA

>P55704

MDINSTSPLNASPQPDSPPPANASAFAHQLSGFQYSPPHAADSLLPQVEADSPYLDTRHP
YSQYLDSAYPYPSPCEWQHDLYTRTRERSPHPSEQRPHARVLQGAPEHDQDQHLEAAGPR
EGSWQVGPSRSGPSQAGLSPSATPLNPSPPPHATDLETKHPYSQYLDWANPSLLDWQQDL
HTRATASPAPLTAERGRSPQPSEQQPHARALQVPEYDQDLIWQRVDAAGPQAGPWQVGPS
HSGPSQARPSHAWPSSSSAGAEPAELSDFVMDSGVRAWDHWFLAPHMASEDQMSMLRATGL
MPTAEVPTTTFLMMGMRHVAEFRGEGVIRIRPSVDFDI

>Q87DU6

MSEITTTDAQAQPEKKDFIRQIIREDLAHGTHTHIHTRFPPEPNGYLHIGHAKAICLDFG
VAAEFGGHCTLRMDDTNPSKEDPAFAAAIQEDVSWLGFHWNALRHTSDYFEVLYLAAEKL
IADGKAYVCDLNSQQVREYRGTLTEAGRPSPWRERSPDENLELFRQMRAGTFPDGTRTLR
AKIDMASGNINLRDPALYRIKHVEHQNTGNTWPIYPMYDFAHALSDAIEGITHSLCTLEF
EDHRPLYDWCINHVDLPNNSHLLKPLLDKGFPQEPSQPRQIEFSRLNINYTVMSKRKLTA
LVDEKLVEGWDDPRMYTLQGLRRRGYTPAAMRLFVERIGISKQNSIIDFSVLENCLRENL
DTIAPRRMATIAPMKLVLTNLAEDHEEQLIFPNHPKDDTQGTRTVPFSRELWIERDDFSE
APPKGWKRLIPGGEVRLRGAGIARIDEVVKNAEGHVIALHGWLDPTSRPGMEGAHRKVKG
TIHWVSAPHAVAAEIRLYDRLFSIEKPDDNTDGKTYRDFLNPDSKRVVHGYIEPAAAQTA
PEHAFQFERLGYFVTDRHDHDATHPVFNRSVTLRDTWQRD

>P26474

MAIKITPDEFSLLIQRLNKKWRVFAPSAEFRGGRFSDTDNIIYQRISGWRDLIWHEKSHM
SPNTIIAPITETLFYFDKDTIQIAETDTSPIIIFARACDINAMSRLDYMYLSNGNNSDYS
YQLLREHIRFVLIECEESFENCFCVSMGTNKTDCYSAAMRFSDEGALVSIRDPFIEAAIQ
GLGQEADYTPSFVSENRETVVTPDSVCHDPQKIRDILTHHPLWDAYDSRCISCGRCTTGC
PTCTCYSVFDVAYDENPQRGERRRQWASCMVPGFSDMAGGHGFREKPGERLRYRALHKVN
DYKARNGIEHMCVGCGRCDDRCPQYIKFSLIINKMTAAVRQALAEEA

>P07623
MPIRVPDELPAVNFLREENVFVMTTSRASGQEIRPLKVLILNLMPKKIETENQFLRLLSN
SPLQVDIQLLRIDSRESRNTPAEHLNNFYCNFEDIQDQNFDGLIVTGAPLGLVEFNDVAY
WPQIKQVLEWSKDHVTSTLFVCWAVQAALNILYGIPKQTRTEKLSGVYEHHILHPHALLT
RGFDDSFLAPHSRYADFPAALIRDYTDLEILAETEEGDAYLFASKDKRIAFVTGHPEYDA
QTLAQEFFRDVEAGLDPDVPYNYFPHNDPQNTPRASWRSHGNLLFTNWLNYYVYQITPYD
LRHMNPTLD
>P23721
MAQIFNFSSGPAMLPAEVLKQAQQELRDWNGLGTSVMEVSHRGKEFIQVAEEAEKDFRDL
LNVPSNYKVLFCHGGGRGQFAAVPLNILGDKTTADYVDAGYWAASAIKEAKKYCTPNVFD
AKVTVDGLRAVKPMREWQLSDNAAYMHYCPNETIDGIAIDETPDFGADVVVAADFSSTIL
SRPIDVSRYGVIYAGAQKNIGPAGLTIVIVREDLLGKANIACPSILDYSILNDNGSMFNT
PPTFAWYLSGLVFKWLKANGGVAEMDKINQQKAELLYGVIDNSDFYRNDVAKANRSRMNV
PFQLADSALDKLFLEESFAAGLHALKGHRVVGGMRASIYNAMPLEGVKALTDFMVEFERR
HG
>P0A9L3
MTTPTFDTIEAQASYGIGLQVGQQLSESGLEGLLPEALVAGIADALEGKHPAVPVDVVHR
ALREIHERADAVRRQRFQAMAAEGVKYLEENAKKEGVNSTESGLQFRVINQGEGAIPART
DRVRVHYTGKLIDGTVFDSSVARGEPAEFPVNGVIPGWIEALTLMPVGSKWELTIPQELA
YGERGAGASIPPFSTLVFEVELLEIL
>P30621
MNTLRPGDAMLTDWRDAAARFEREIAKAVVGQDRAIRLLTIAIFARGHVMLEGDVGVGKT
TLLRAVARGLGGAYERVEGTVDMMPTDLIYHTYLGEDGRPRVEPGPVLRRAEDLSVFFFN
EINRARPQVHALLLRIMAERSVSAFNREYRFPNLQVFADRNRVEREETFELPAAARDRFL
MEIGMEAPRDAARRDLVFDPRFHDTDRLTEEVEAGVLDFERIGTIASAIQHAISAEPAIE
AYVVGLWEALVRPAPPHPLARHRMDRLVQGGASPRGVAFLVRAARVRAWLEGRDWLVPED
IRAVFPEVMAHRVFLEPVYEMRRAEIVPDLIRAVFETVPAP
>P11458
MSVMFDPDTAIYPFPPKPTPLSIDEKAYYREKIKRLLKERNAVMVAHYYTDPEIQQLAEE
TGGCISDSLEMARFGAKHPASTLLVAGVRFMGETAKILSPEKTILMPTLQAECSLDLGCP
VEEFNAFCDAHPDRTVVVYANTSAAVKARADWVVTSSIAVELIDHLDSLGEKIIWAPDKH
LGRYVQKQTGGDILCWQGACIVHDEFKTQALTRLQEEYPDAAILVHPESPQAIVDMADAV
GSTSQLIAAAKTLPHQRLIVATDRGIFYKMQQAVPDKELLEAPTAGEGATCRSCAHCPWM
AMNGLQAIAEALEQEGSNHEVHVDERLRERALVPLNRMLDFAATLRG
>P0A282
MAIKLIVGLANPGAEYAATRHNAGAWYVDLLAERLRAPLREEPKFFGYTSRITLEGEDVR
LLVPTTFMNLSGKAVGAMASFYRIQPDEILVAHDELDLPPGVAKFKLGGGHGGHNGLKDI
ISKLGNNPNFHRLRVGIGHPGDKNKVVGFVLGKPPVSEQKLIDEAIDEAARCTELWFKEG
LAKATSRLHTFKAQ
>P0A9N8
MNYHQYYPVDIVNGPGTRCTLFVSGCVHECPGCYNKSTWRVNSGQPFTKAMEDQIINDLN
DTRIKRQGISLSGGDPLHPQNVPDILKLVQRIRAECPGKDIWVWTGYKLDELNAAQMQVV
DLINVLVDGKFVQDLKDPSLIWRGSSNQVVHHLR
>Q9JT95

MLVHPEAMSVGALADKIRKIENWPQKGILFHDITPVLQSAEYFRLLVDLLVYRYMDQKID
IVAGLDARGFIIGAALAYQLNVGFVPIRKKGKLPFETVSQSYALEYGEAAVEIHTDAVKL
GSRVLLVDDLVATGGTMLAGLELIRKLGGEIVEAAAILEFTDLQGGKNIRASGAPLFTLL
QNEGCMKG

>P39440

MTQHSPYSSAMAEQRHQEWLRFVELLRQSYDKDLHLPLLQLMLTPDEREALGTRVRIIEE
LLRGEMSQRELKNELGAGIATITRGSNSLKSAPVELRQWLEQILLGAQR

>P43341

MATLFIADLHLCVEEPAITAGFLRFLAGEARKADALYILGDLFEAWIGDDDPNPLHRKMA
AAIKAVSDSGVPCYFIHGNRDFLLGKRFARESGMTLLPEEKVLELYGRRVLIMHGDTLCT
DDAGYQAFRAKVHKPWLQTLFLALPLFVRKRIAARMRANSKEANSSKSLAIMDVNQNAVV
SAMEKHQVQWLIHGHTHRPAVHELIANQQPAFRVVLGAWHTEGSMVKVTADDVELIHFPF

>P0A9H7

MSSSCIEEVSVPDDNWYRIANELLSRAGIAINGSAPADIRVKNPDFFKRVLQEGSLGLGE
SYMDGWWECDRLDMFFSKVLRAGLENQLPHHFKDTLRIAGARLFNLQSKKRAWIVGKEHY
DLGNDLFSRMLDPFMQYSCAYWKDADNLESAQQAKLKMICEKLQLKPGMRVLDIGCGWGG
LAHYMASNYDVSVVGVTISAEQQKMAQERCEGLDVTILLQDYRDLNDQFDRIVSVGMFEH
VGPKNYDTYFAVVDRNLKPEGIFLLHTIGSKKTDLNVDPWINKYIFPNGCLPSVRQIAQS
SEPHFVMEDWHNFGADYDTTLMAWYERFLAAWPEIADNYSERFKRMFTYYLNACAGAFRA
RDIQLWQVVFSRGVENGLRVAR

>Q1CZL0

MSVQRPIRVLVVDDSPTMANMLTALLTEEPRIEVVGRAGDGNRAVQLARLLRPDVITMDL
LLPGLDGPGAIAAIMSQSPARILVVSAVAEQRGVDLGFQAMSAGALELIGKPNVTNAEEL
RRWGKELAHSVCLMAEVPVISRRPRAATAPPPPTGARVDIFGVVASTGGPPALADVLSKL
PRSLPVPLLIAQHITVGFTQGMVRWLSQVTPLPVSIAKDGERLEPGRVYFPLDGHDLLVD
AAGLARLQPSQGGPCPSGDVMLTSLAAAFGRRSGGVVLTGMGEDGARGLLAIRRAGGVTF
SQDEASSVVFGMPRAALDVKATDQGVPLASMPELILQSCTFAPFRGGRPEGGPTR

>P46849

MKRMIALDGAQGEGGGQILRSALSLSMITGQPFTITSIRAGRAKPGLLRQHLTAVKAATE
ICGATVEGAELGSQRLLFRPGTVRGGDYRFAIGSAGSCTLVLQTVLPALWFADGPSRVEV
SGGTDNPSAPPADFIRRVLEPLLAKIGIHQQTTLLRHGFYPAGGGVVATEVSPVASFNTL
QLGERGNIVQMRGEVLLAGVPRHVAEREIATLAGSFSLHEQNIHNLPRDQGPGNTVSLEV
ESENITERFFVVGEKRVSAEVVAAQLVKEVKRYLASTAAVGEYLADQLVLPMALAGAGEF
TVAHPSCHLLTNIAVVERFLPVRFSLIETDGVTRVSIE

>P22317

MDSRITTILERYRSDRTRLIDILWDVQHEYGHIPDAVLPQLGAGLKLSPLDIRETASFYH
FFLDKPSGKYRIYLCNSVIAKINGYQAVREALERETGIRFGETDPNGMFGLFDTPCIGLS
DQEPAMLIDKVVFTRLRPGKITDIIAQLKQGRSPAEIANPAGLPSQDIAYVDAMVESNVR
TKGPVFFRGRTDLRSLLDQCLLLKPEQVIETIVDSRLRGRGGAGFSTGLKWRLCRDAESE
QKYVICNADEGEPGTFKDRVLLTRAPKKVFVGMVIAAYAIGCRKGIVYLRGEYFYLKDYL
ERQLQELREDGLLGRAIGGRAGFDFDIRIQMGAGAYICGDESALIESCEGKRGTPRVKPP
FPVQQGYLGKPTSVNNVETFAAVSRIMEEGADWFRAMGTPDSAGTRLLSVAGDCSKPGIY
EVEWGVTLNEVLAMVGARDARAVQISGPSGECVSVAKDGERKLAYEDLSCNGAFTIFNCK

RDLLEIVRDHMQFFVEESCGICVPCRAGNVDLHRKVEWVIAGKACQKDLDDMVSWGALVR
RTSRCGLGATSPKPILTTLEKFPEIYQNKLVRHEGPLLPSFDLDTALGGYEKALKDLEEV
TR
>P0A890
MTDLFSSPDHTLDALGLRCPEPVMMVRKTVRNMQPGETLLIIADDPATTRDIPGFCTFME
HELVAKETDGLPYRYLIRKGG
>P42195
MARYLEVSDIVQQWRNERPDLDVEPMLVIGTLSRVSLLIDRALDKVFSKYKLSAREFDIL
ATLRRRGAPYAYSPSQIVNALMINNSTLTSRLDRLEQAGWLRRMPIEGDRRSVNIQLTDE
GFALINRVVEEHVENERDILSPFSEEEKTHLRALLGRVEKHLVNNR
>P44527
MQTKYDLSTMFIHSGRQKRFSQGSVNPVLQRASSLLFDSIEDKKHATQRRAKGELFYGRR
GTLTHFALQDLMCEMEGGAGCYLYPCGTAAVTNSILSFVKTGDHVLMSGAAYEPTQYFCN
IVLKKMQIDITYYDPLIGEDIATLIQPNTKVLFLEAPSSITMEIPDIPTIVKAARKVNPN
IVIMIDNTWSAGVLFKALEHDIDISIQAGTKYLVGHSDIMIGTAVANARTWDQLREHSYL
MGQMVDADSAYTTARGIRTLGVRLKQHQESSIKVAKWLSEQPEVKTVYHPALPSCPGHEF
FLRDFSGSSGLFSFELTQRLTSEQVSKFMDHFQLFAMAYSWGGFESLILCNQPEEIAHIR
PNIKRNLTGSLIRVHIGFENVDELIADLKAGFERIA
>P52966
MLGLGYIGRKLFGTPNDRKVKRTRPLVAKINALEPAFEKLSDAEIVAKTRELQARAQAGE
SLDALLVEAFANCREAARRALGLRAFDTQLMGGIFLHQGNIAEMKTGEGKTLVATFPAYL
NALAGKGVHIVTVNDYLARRDSEWMGKVYRHLGLTCGVVYPFQPDDEKRAAYGADITYAT
NNELGFDYLRDNMKSSVAEMYQRDHFFAIVDEVDSILIDEARTPLIISGPSQDRSDMYRT
LDAYIPFLTEEHYKLDEKQRNATFTEEGNEFLEQKLQADGLLPEGQSLYDPESTTIVHHI
GQALRAHKLFFKDQNYVVTDDEIVLIDEFTGRMMKGRRLSDGLHQAIEAKERVTIQPENV
TLASVTFQNYFRLYEKLAGMTGTAVTEAEEFGDIYKLGVVEVPTNRPVARKDEHDRVYRT
AKEKYAAVIEAIKTAHEKGQPTLVGTTSIEKSEMLSEMLKAEGLPHNVLNARQHEQEAQI
VADAGRLGAITIATNMAGRGTDIQLGGNVEMKVQEEIAANPEAAPEEIRARIEAEHAAEK
QKVIEAGGLFVLATERHESRRIDNQLRGRSGRQGDPGRSLFFLSLEDDLMRIFGSDRLEG
VLSKLGMKEGEAIIHPWVNKSLERAQAKVEGRNFDWRKQLLKFDDVMNDQRKAVFGQRRE
IMETDEISEIVADMRQQVIDDLIDDFAPPKSYVDQWDIEGMRAAFIDHAGVDLPLADWAA
EEGVDQDVLRERVTAALDAVMAQKTEAFGAETMRVIEKQILLQTIDAKWREHLVTLEHLR
SVVGFRGYAQRDPLSEYKTESFQLFESMLDSLRYEVTKRLGQIRPMSDEERAEMLRQQAA
ALAAAEGAADPAEAPAPQPAAQVALAAAPGFVESDPTTWGEPSRNDPCPCGSGEKFKHCH
GRLA
>P26612
MRNPTLLQCFHWYYPEGGKLWPELAERADGFNDIGINMVWLPPAYKGASGGYSVGYDSYD
LFDLGEFDQKGSIPTKYGDKAQLLAAIDALKRNDIAVLLDVVVNHKMGADEKEAIRVQRV
NADDRTQIDEEIIECEGWTRYTFPARAGQYSQFIWDFKCFSGIDHIENPDEDGIFKIVND
YTGEGWNDQVDDELGNFDYLMGENIDFRNHAVTEEIKYWARWVMEQTQCDGFRLDAVKHI
PAWFYKEWIEHVQEVAPKPLFIVAEYWSHEVDKLQTYIDQVEGKTMLFDAPLQMKFHEAS
RMGRDYDMTQIFTGTLVEADPFHAVTLVANHDTQPLQALEAPVEPWFKPLAYALILLREN
GVPSVFYPDLYGAHYEDVGGDGQTYPIDMPIIEQLDELILARQRFAHGVQTLFFDHPNCI
AFSRSGTDEFPGCVVVMSNGDDGEKTIHLGENYGNKTWRDFLGNRQERVVTDENGEATFF

CNGGSVSVWVIEEVI
>P10443
MSEPRFVHLRVHSDYSMIDGLAKTAPLVKKAAALGMPALAITDFTNLCGLVKFYGAGHGA
GIKPIVGADFNVQCDLLGDELTHLTVLAANNTGYQNLTLLISKAYQRGYGAAGPIIDRDW
LIELNEGLILLSGGRMGDVGRSLLRGNSALVDECVAFYEEHFPDRYFLELIRTGRPDEES
YLHAAVELAEARGLPVVATNDVRFIDSSDFDAHEIRVAIHDGFTLDDPKRPRNYSPQQYM
RSEEEMCELFADIPEALANTVEIAKRCNVTVRLGEYFLPQFPTGDMSTEDYLVKRAKEGL
EERLAFLFPDEEERLKRRPEYDERLETELQVINQMGFPGYFLIVMEFIQWSKDNGVPVGP
GRGSGAGSLVAYALKITDLDPLEFDLLFERFLNPERVSMPDFDVDFCMEKRDQVIEHVAD
MYGRDAVSQIITFGTMAAKAVIRDVGRVLGHPYGFVDRISKLIPPDPGMTLAKAFEAEPQ
LPEIYEADEEVKALIDMARKLEGVTRNAGKHAGGVVIAPTKITDFAPLYCDEEGKHPVTQ
FDKSDVEYAGLVKFDFLGLRTLTIINWALEMINKRRAKNGEPPLDIAAIPLDDKKSFDML
QRSETTAVFQLESRGMKDLIKRLQPDCFEDMIALVALFRPGPLQSGMVDNFIDRKHGREE
ISYPDVQWQHESLKPVLEPTYGIILYQEQVMQIAQVLSGYTLGGADMLRRAMGKKKPEEM
AKQRSVFAEGAEKNGINAELAMKIFDLVEKFAGYGFNKSHSAAYALVSYQTLWLKAHYPA
EFMAAVMTADMDNTEKVVGLVDECWRMGLKILPPDINSGLYHFHVNDDGEIVYGIGAIKG
VGEGPIEAIIEARNKGGYFRELFDLCARTDTKKLNRRVLEKLIMSGAFDRLGPHRAALMN
SLGDALKAADQHAKAEAIGQADMFGVLAEEPEQIEQSYASCQPWPEQVVLDGERETLGLY
LTGHPINQYLKEIERYVGGVRLKDMHPTERGKVITAAGLVVAARVMVTKRGNRIGICTLD
DRSGRLEVMLFTDALDKYQQLLEKDRILIVSGQVSFDDFSGGLKMTAREVMDIDEAREKY
ARGLAISLTDRQIDDQLLNRLRQSLEPHRSGTIPVHLYYQRADARARLRFGATWRVSPSD
RLLNDLRGLIGSEQVELEFD
>P95435
MDTSLIRELAELALAGSGQHCHEEALCIAEWLERLGQDEAARLIRISSLANQGRYQEALA
FAHGNPWPALEPWFALCEWHLGLGAALDRRLAGLGGSSDPALADFAAGMRAQVRT
>P06989
MLTEQQRRELDWEKTDGLMPVIVQHAVSGEVLMLGYMNPEALDKTLESGKVTFFSRTKQR
LWTKGETSGNFLNVVSIAPDCDNDTLLVLANPIGPTCHKGTSSCFGDTAHQWLFLYQLEQ
LLAERKSADPETSYTAKLYASGTKRIAQKVGEEGVETALAATVHDRFELTNEASDLMYHL
LVLLQDQGLDLTTVIENLRKRHQ
>P77499
MLSIKDLHVSVEDKAILRGLSLDVHPGEVHAIMGPNGSGKSTLSATLAGREDYEVTGGTV
EFKGKDLLALSPEDRAGEGIFMAFQYPVEIPGVSNQFFLQTALNAVRSYRGQETLDRFDF
QDLMEEKIALLKMPEDLLTRSVNVGFSGGEKKRNDILQMAVLEPELCILDESDSGLDIDA
LKVVADGVNSLRDGKRSFIIVTHYQRILDYIKPDYVHVLYQGRIVKSGDFTLVKQLEEQG
YGWLTEQQ
>P0A988
MKFTVEREHLLKPLQQVSGPLGGRPTLPILGNLLLQVADGTLSLTGTDLEMEMVARVALV
QPHEPGATTVPARKFFDICRGLPEGAEIAVQLEGERMLVRSGRSRFSLSTLPAADFPNLD
DWQSEVEFTLPQATMKRLIEATQFSMAHQDVRYYLNGMLFETEGEELRTVATDGHRLAVC
SMPIGQSLPSHSVIVPRKGVIELMRMLDGGDNPLRVQIGSNNIRAHVGDFIFTSKLVDGR
FPDYRRVLPKNPDKHLEAGCDLLKQAFARAAILSNEKFRGVRLYVSENQLKITANNPEQE
EAEEILDVTYSGAEMEIGFNVSYVLDVLNALKCENVRMMLTDSVSSVQIEDAASQSAAYV
VMPMRL

>P22259

MRVNNGLTPQELEAYGISDVHDIVYNPSYDLLYQEELDPSLTGYERGVLTNLGAVAVDTG
IFTGRSPKDKYIVRDDTTRDTFWWADKGKGKNDNKPLSPETWQHLKGLVTRQLSGKRLFV
VDAFCGANPDTRLSVRFITEVAWQAHFVKNMFIRPSDEELAGFKPDFIVMNGAKCTNPQW
KEQGLNSENFVAFNLTERMQLIGGTWYGGEMKKGMFSMMNYLLPLKGIASMHCSANVGEK
GDVAVFFGLSGTGKTTLSTDPKRRLIGDDEHGWDDDGVFNFEGGCYAKTIKLSKEAEPEI
YNAIRRDALLENVTVREDGTIDFDDGSKTENTRVSYPIYHIDNIVKPVSKAGHATKVIFL
TADAFGVLPPVSRLTADQTQYHFLSGFTAKLAGTERGITEPTPTFSACFGAAFLSLHPTQ
YAEVLVKRMQAAGAQAYLVNTGWNGTGKRISIKDTRAIIDAILNGSLDNAETFTLPMFNL
AIPTELPGVDTKILDPRNTYASPEQWQEKAETLAKLFIDNFDKYTDTPAGAALVAAGPKL

>P0A731

MELTTRTLPARKHIALVAHDHCKQMLMSWVERHQPLLEQHVLYATGTTGNLISRATGMNV
NAMLSGPMGGDQQVGALISEGKIDVLIFFWDPLNAVPHDPDVKALLRLATVWNIPVATNV
ATADFIIQSPHFNDAVDILIPDYQRYLADRLK

>P44406

MSKLVLILNCGSSSLKFAILDPATGEEKLSGLAEAFFLPEARIKWKLNGEKGNADLGAGA
AHTEALNFIASNILNDELKNSIAAIGHRIVHGGEKYTQSVIVTDEVVKGIEDAAQFAPLH
NPAHLIGIREAFKAFPHLKDKNVVVFDTAFHQTMPEEAFLYALPYSLYKEHGVRRYGAHG
TSHYFVSREVAKYVGKPADQVNAIICHLGNGGSVSVVRNGQCIDTSMGLTPLEGLVMGTR
CGDIDPAIVFYLYKTLGMSMDQIEETLVKKSGLLGLTEVTSDCRYAEDNYDDESKPETRR
ALNVYSYRLAKYIGAYMAVLGDDHLDAIAFTGGIGENSAHVRELALNHLKLFGIKIDNER
NLATRFGKDGVITTDDSAFKAIVLPTNEELVIAQDTAKLCF

>P60716

MSKPIVMERGVKYRDADKMALIPVKNVATEREALLRKPEWMKIKLPADSTRIQGIKAAMR
KNGLHSVCEEASCPNLAECFNHGTATFMILGAICTRRCPFCDVAHGRPVAPDANEPVKLA
QTIADMALRYVVITSVDRDDLRDGGAQHFADCITAIREKSPQIKIETLVPDFRGRMDRAL
DILTATPPDVFNHNLENVPRIYRQVRPGADYNWSLKLLERFKEAHPEIPTKSGLMVGLGE
TNEEIIEVMRDLRRHGVTMLTLGQYLQPSRHHLPVQRYVSPDEFDEMKAEALAMGFTHAA
CGPFVRSSYHADLQAKGMEVK

>Q46ZI4

MTQNENPSAQSGAKPEDKARPAKALQGVKGMNDMLPADAPLWEHFENAARAMLRAYGYQQ
IRTPIVEHTQLFVRGIGEVTDIVEKEMYSFTDALNGEQLTLRPEGTAAAVRATIEHNLLY
DGPKRLWYTGPMFRHERPQRGRYRQFHQLGAEALGFAGPDVDAEIILMCQRLWDDLGLTG
VRLEINSLGQAHERAAHREELIKYLEGFKDILDEDGKRRLYTNPLRVLDTKNPALQEMAA
NAPKLIDFLGEESLAHFEGVQRLLKANNIPYKINPRLVRGLDYYNLTVFEWITDKLGAQG
TIAGGGRYDPLIAQMGGKAAPACGWAMGIERIIELIREEGVVPDAVGCDVYLVHQGEAAQ
QQATVAAERLRDAGLDVVLHATPDGKSGSFKSQMKRADASGAAYAVIIGEDEVAAGVVQV
KELRQGAQAEGGGQQAQVPAENLVDYLIDAMVGASE

>P03004

MSLSLWQQCLARLQDELPATEFSMWIRPLQAELSDNTLALYAPNRFVLDWVRDKYLNNIN
GLLTSFCGADAPQLRFEVGTKPVTQTPQAAVTSNVAAPAQVAQTQPQRAAPSTRSGWDNV
PAPAEPTYRSNVNVKHTFDNFVEGKSNQLARAAARQVADNPGGAYNPLFLYGGTGLGKTH
LLHAVGNGIMARKPNAKVVYMHSERFVQDMVKALQNNAIEEFKRYYRSVDALLIDDIQFF

ANKERSQEEFFHTFNALLEGNQQIILTSDRYPKEINGVEDRLKSRFGWGLTVAIEPPELE
TRVAILMKKADENDIRLPGEVAFFIAKRLRSNVRELEGALNRVIANANFTGRAITIDFVR
EALRDLLALQEKLVTIDNIQKTVAEYYKIKVADLLSKRRSRSVARPRQMAMALAKELTNH
SLPEIGDAFGGRDHTTVLHACRKIEQLREESHDIKEDFSNLIRTLSS
>P45131
MSVQNVVLFDTQPLTLMLGGKLSHINVAYQTYGTLNAEKNNAVLICHALTGDAEPYFDDG
RDGWWQNFMGAGLALDTDRYFFISSNVLGGCKGTTGPSSINPQTGKPYGSQFPNIVVQDI
VKVQKALLDHLGISHLKAIIGGSFGGMQANQWAIDYPDFMDNIVNLCSSIYFSAEAIGFN
HVMRQAVINDPNFNGGDYYEGTPPDQGLSIARMLGMLTYRTDLQLAKAFGRATKSDGSFW
GDYFQVESYLSYQGKKFLERFDANSYLHLLRALDMYDPSLGYDNVKEALSRIKARYTLVS
VTTDQLFKPIDLYKSKQLLEQSGVDLHFYEFPSDYGHDAFLVDYDQFEKRIRDGLAGN
>P69922
MKKISLPKIGIRPVIDGRRMGVRESLEEQTMNMAKATAALLTEKLRHACGAAVECVISDT
CIAGMAEAAACEEKFSSQNVGLTITVTPCWCYGSETIDMDPTRPKAIWGFNGTERPGAVY
LAAALAAHSQKGIPAFSIYGHDVQDADDTSIPADVEEKLLRFARAGLAVASMKGKSYLSL
GGVSMGIAGSIVDHNFFESWLGMKVQAVDMTELRRRIDQKIYDEAELEMALAWADKNFRY
GEDENNKQYQRNAEQSRAVLRESLLMAMCIRDMMQGNSKLADIGRVEESLGYNAIAAGFQ
GQRHWTDQYPNGDTAEAILNSSFDWNGVREPFVVATENDSLNGVAMLMGHQLTGTAQVFA
DVRTYWSPEAIERVTGHKLDGLAEHGIIHLINSGSAALDGSCKQRDSEGNPTMKPHWEIS
QQEADACLAATEWCPAIHEYFRGGGYSSRFLTEGGVPFTMTRVNIIKGLGPVLQIAEGWS
VELPKDVHDILNKRTNSTWPTTWFAPRLTGKGPFTDVYSVMANWGANHGVLTIGHVGADF
ITLASMLRIPVCMHNVEETKVYRPSAWAAHGMDIEGQDYRACQNYGPLYKR
>Q9FA38
MAKITKVQVGEALVGDGNEVAHIDLIIGPRGSPAETAFCNGLVNNKHGFTSLLAVIAPNL
PCKPNTLMFNKVTINDARQAVQMFGPAQHGVAMAVQDAVAEGIIPADEADDLYVLVGVFI
HWEAADDAKIQKYNYEATKLSIQRAVNGEPKASVVTEQRKSATHPFAANA
>P0A9C0
MKTRDSQSSDVIIIGGGATGAGIARDCALRGLRVILVERHDIATGATGRNHGLLHSGARY
AVTDAESARECISENQILKRIARHCVEPTNGLFITLPEDDLSFQATFIRACEEAGISAEA
IDPQQARIIEPAVNPALIGAVKVPDGTVDPFRLTAANMLDAKEHGAVILTAHEVTGLIRE
GATVCGVRVRNHLTGETQALHAPVVVNAAGIWGQHIAEYADLRIRMFPAKGSLLIMDHRI
NQHVINRCRKPSDADILVPGDTISLIGTTSLRIDYNEIDDNRVTAEEVDILLREGEKLAP
VMAKTRILRAYSGVRPLVASDDDPSGRNVSRGIVLLDHAERDGLDGFITITGGKLMTYRL
MAEWATDAVCRKLGNTRPCTTADLALPGSQEPAEVTLRKVISLPAPLRGSAVYRHGDRTP
AWLSEGRLHRSLVCECEAVTAGEVQYAVENLNVNSLLDLRRRTRVGMGTCQGELCACRAA
GLLQRFNVTTSAQSIEQLSTFLNERWKGVQPIAWGDALRESEFTRWVYQGLCGLEKEQKD
AL
>P43336
MSHFAKVARVPGDPILGLLDAYRNDPRADKLDLGVGVYKDAQGLTPILRSVKLAEQRLVE
QETTKSYVGGHGDALFAARLAELALGAASPLLLEQRADATQTPGGTGALRLAGDFIAHCL
PGRGIWLSDPTWPIHETLFAAAGLKVSHYPYVSADNRLDVEAMLAGLERIPQGDVVLLHA
CCHNPTGFDLSHDDWRRVLDVVRRRELLPLIDFAYQGFGDGLEEDAWAVRLFAGELPEVL
VTSSCSKNFGLYRDRVGALIVCAQNAEKLTDLRSQLAFLARNLWSTPPAHGAEVVAAILG
DSELKGLWQEEVEGMRSRIASLRIGLVEALAPHGLAERFAHVGAQRGMFSYTGLSPQQVA

RLRDEHAVYLVSSGRANVAGLDARRLDRLAQAIAQVCAD
>P50319
MMSLSHATNPQPKPAAAPHTLAALLAAGGLAGKRVFIRADLNVPQDAAGAITDDTRIRAS
VPAIAACLQAGAAVMVTSHLGRPQEGAPDPRHSLAPVGRRLSELLGRQVPLLSGWTEGGF
QVPPGQVVLLENCRMNTGEKKNSDELAQKMAALCDVYVNDAFGTAHRAEATTHGIARYAP
IACAGPLLAAEIDALGKALGQPARPLVAIVAGSKVSTKLTILKSLADKVDNLIVGGGIAN
TFMLAAGLKIGKSLAEPDLLADARAIIDIMARRGASVPIPVDVVCAKEFSATAAATVKDV
KDVADDDMILDIGPKTAAMLAEQLKAAGTIVWNGPVGVFEFDQFGNGTRVLAQAIAESKA
FSIAGGGDTLAAIAKHGIADRVGYISTGGGAFLEFLEGKTLPALAVLAQRAAA
>Q5P409
MADIPAATPSATPFPPELLRDVERRRTFGIISHPDAGKTTLTEKLLLFGGAIQLAGTVKA
RKSARHATSDWMEVEKQRGISVSSSVMQFEYAGHTINLLDTPGHQDFSEDTYRVLTAVDA
AVMVIDAAKGVETQTIKLLEVCRLRNTPIITFINKMDREVRESFDLLQEIEEVLKIDCAP
ITWPIGMGKTFRGVYHLLEDRVLRFTPGEEKRSEAEVIKGIANAQLDELFPLEVGKLREE
VDLIQGASNPFSLGDFLAGKQTPVFFGSGINNFGVQEILQALLDWAPPPQPRVAGVTVAD
TRLVMPAEAPFSGFVFKIQANMDPKHRDRIAFFRICSGRYSSGMKVKHVRMGREMKLANA
LTFMANERVLMEDGVAGDIIGIHNHGQLHIGDTLTEGENLGYKGIPYFSPELFSAARLRD
PLKSKQLQKGLQELGEEGAIQVFEQEGGNMLLGAVGQLQFEVVAQRLKDEYKVDAIFESA
DIYTARWLVFPDEVTRRNFEREQGIRVGKDVDGNPVYLATSRYNLEVTMEKWPKVGFHAT
REHGEKLS
>O85014
MSSNTSAPSLNALAGPLVESLVADAAKLRLIVAQENGARTVDAGANARGSIEAGRRIAEI
CLGGLGTVTIAPIGPVASWPYTVVVHSADPVLACLGSQYAGWSLADEEGDSGFFALGSGP
GRAVAVVEELYKELGYRDNATTTALVLESGSAPPASVVNKVAAATGLAPENVTFIYAPTQ
SLAGSTQVVARVLEVALHKAHTVGFDLHKILDGIGSAPLSPPHPDFIQAMGRTNDAIIYG
GRVQLFVDADDADAKQLAEQIPSTTSADHGAPFAEIFSRVNGDFYKIDGALFSPAEAIVT
SVKTGKSFRGGRLEPQLVDASFV
>O05529
MTKWAASLIRISNHEVETLQKRLAEITERRMAAEMRVTLLDAEAEAEAKNAEGDPSAGWY
MIGYREGSKRRRADMLVQIEQCQQEEAGARDALSEAFENLKKYEHVAEQAKILAAKKMNA
FEAAQMDELSIRRAAVGGR
>P15070
MSDMNNPADDNNGAMDDLWAEALSEQKSTSSKSAAETVFQQFGGGDVSGTLQDIDLIMDI
PVKLTVELGRTRMTIKELLRLTQGSVVALDGLAGEPLDILINGYLIAQGEVVVVADKYGV
RITDIITPSERMRRLSR
>P0C6D6
MIGKKSFQTNVYRMSKFDTYIFNNLYINDYKMFWIDSGIAKLIDKNCLVSYEINSSSIIL
LKKNSIQRFSLTSLSDENINVSVITISDSFIRSLKSYILGDLMIRNLYSENKDLLLWNCE
HNDIAVLSEVVNGFREINYSDEFLKVFFSGFFSKVEKKYNSIFITDDLDAMEKISCLVKS
DITRNWRWADICGELRTNRMILKKELESRGVKFRELINSIRISYSISLMKTGEFKIKQIA
YQSGFASVSYFSTVFKSTMNVAPSEYLFMLTGVAEK
>P0A968
MEKGTVKWFNNAKGFGFICPEGGGEDIFAHYSTIQMDGYRTLKAGQSVQFDVHQGPKGNH
ASVIVPVEVEAAVA

>P0C058
MRNFDLSPLMRQWIGFDKLANALQNAGESQSFPPYNIEKSDDNHYRITLALAGFRQEDLE
IQLEGTRLSVKGTPEQPKEEKKWLHQGLMNQPFSLSFTLAENMEVSGATFVNGLLHIDLI
RNEPEPIAAQRIAISERPALNS
>Q9EXP2
MNYPVEHYPIDRVRADFPILQQSVNGQPLAYLDSAASAQKPLAVIDRERDFYLHEYAAVH
RGIHTLSARATSAMEEVRAKVATFIHAASAEDIVFVRGTTEAINLVANSYGRTAFQPGDN
LVISEMEHHANIVPWQMLAQARGLTLRVLPITDDGELDMAQLPALLDERTRLVAVTQVSN
VLGTVNPLAEIIRQAHACGAKVLVDGAQAVMHQAVDVQALDCDFYAFSGHKLYGPSGIGV
LYGKSELLQAMPPWEGGGAMIREVSLTQGTTYADPPWRFEAGSPHVAGIIGLGAALDYVS
ALGVDAIQAHEGLLMRYALASLAEVPTLRLYGPVHRQGVIAFNLGRHHAFDVGSFLDQYG
IAIRTGHHCAMPLMSRYGVPSMCRASLALYSCQDEIDRLVAGLHRIHRLLGE
>P09378
MAHQLKLLKDDFFASDQQAVAVADRYPQDVFAEHTHDFCELVIVWRGNGLHVLNDRPYRI
TRGDLFYIHADDKHSYASVNDLVLQNIIYCPERLKLNLDWQGAIPGFNASAGQPHWRLGS
MGMAQARQVIGQLEHESSQHVPFANEMAELLFGQLVMLLNRHRYTSDSLPPTSSETLLDK
LITRLAASLKSPFALDKFCDEASCSERVLRQQFRQQTGMTINQYLRQVRVCHAQYLLQHS
RLLISDISTECGFEDSNYFSVVFTRETGMTPSQWRHLNSQKD
>P42673
MRIVLLTGFEPFDQDPVNPSWEAVRQLDGVQLGSDVKIVARRLPCAFATAGECLTRLIDE
LHPAMVIATGLGPGRSDISVERVAININDARIPDNLGEQPIDTAVVADGPAAFFTTLPIK
AMVKAVREAGIAASVSQTAGTFVCNQVFYLLQHALAGSGVRSGFIHVPFLPEQVAGSQRP
SMALDAMVAGLQAAVLTAWHTPVDVKEAGGQVS
>P50286
MAKPQVTILATGGTIAGSGESSVKSSYSAGAVTVDKLLAAVPAINDLATIKGEQISSIGS
QEMTGKVWLKLAKRVNELLAQKETEAVIITHGTDTMEETAFFLNLTVKSQKPVVLVGAMR
SGSSMSADGPMNLYNAVNVAINKASTNKGVVIVMNDEIHAAREATKLNTTAVNAFASPNT
GKIGTVYYGKVEYFTQSVRPHTLASEFDISKIEELPRVDILYAHPDDTDVLVNAALQAGA
KGIIHAGMGNGNPFPLTQNALEKAAKSGVVVARSSRVGSGSTTQEAEVDDKKLGFVATES
LNPQKARVLLMLALTKTSDREAIQKIFSTY
>Q46M54
MEFRQLRYFVAAAEEGNVGAAARRLHISQPPVTRQIHALEQHLGVLLFERSARGVQLTPA
GAAFLEDARRMLELGRTSVDRSRAASRGEIGQLDIGYLGTAIYQTVPALLHAFTQAVPGA
TLSLALMPKVRQIEALRAGTIHLGVGRFYPQEPGITVEHLHYERLYIAAGSSIARQLRQD
PTLLRLKSESLVLFPKEGRPSFADEVIALMRRAGVEPRVTAIVEDVNAALGLVAAGAGVT
LVPASVAAIRRPFVRTMEMADASAKVPVSLTYLTDSRVPVLRAFLDVARRGKGQK
>P0A9K9
MKVAKDLVVSLAYQVRTEDGVLVDESPVSAPLDYLHGHGSLISGLETALEGHEVGDKFDV
AVGANDAYGQYDENLVQRVPKDVFMGVDELQVGMRFLAETDQGPVPVEITAVEDDHVVVD
GNHMLAGQNLKFNVEVVAIREATEEELAHGHVHGAHDHHHDHDHDGCCGGHGHDHGHEHG
GEGCCGGKGNGGCGCH
>P94845
MIVRTQNSESKIKEFFEFCKENEVEFVDFRFSDIKGTWNHIAYSFGALTHGMLKEGIPFD
ASCFKGWQGIEHSDMILTPDLVRYFIDPFSADVSVVVFCDVYDVYKNQPYEKCPRSIAKK

ALQHLKDSGLGDVAYFGAENEFFIFDSIKIKDASNSQYYEVDSEEGEWNRDRSFENGVNF
GHRPGKQGGYMPVPPTDTMMDIRTEIVKVLNQVGLETFVVHHEVAQAQGEVGVKFGDLVE
AADNVQKLKYVVKMVAHLNGKTATFMPKPLYGDNGSGMHTHVSVWKNNENLFSGETYKGL
SEFALHFLGGVLRHARGLAAFTNASTNSYKRLIPGYEAPSILTYSANNRSASVRIPYGIS
KNSARFEFRFPDSSSNPYLAFAAILMAGMDGVKNKIDPGEAMDINLFKLTLDEIREKGIK
QMPHTLRRSLEEMLADKQYLKESQVFSEEFIQAYQSLKFNAEVFPWESKPHPFEFITTYS
C
>P39662
MLTQKTKDIVKATAPVLAEHGYDIIKCFYQRMFEAHPELKNVFNMAHQEQGQQQQALARA
VYAYAENIEDPNSLMAVLKNIANKHASLGVKPEQYPIVGEHLLAAIKEVLGNAATDDIIS
AWAQAYGNLADVLMGMESELYERSAEQPGGWKGWRTFVIREKRPESDVITSFILEPADGG
PVVNFEPGQYTSVAIDVPALGLQQIRQYSLSDMPNGRSYRISVKREGGGPQPPGYVSNLL
HDHVNVGDQVKLAAPYGSFHIDVDAKTPIVLISGGVGLTPMVSMLKVALQAPPRQVVFVH
GARNSAVHAMRDRLREAAKTYENLDLFVFYDQPLPEDVQGRDYDYPGLVDVKQIEKSILL
PDADYYICGPIPFMRMQHDALKNLGIHEARIHYEVFGPDLFAE
>P22524
MPVKLAQALANPLFPALDSALRSGRHIGLDELDNHAFLMDFQEYLEEFYARYNVELIRAP
EGFFYLRPRSTTLIPRSVLSELDMMVGKILCYLYLSPERLANEGIFTQQELYDELLTLAD
EAKLLKLVNNRSTGSDVDRQKLQEKVRSSLNRLRRLGMVWFMGHDSSKFRITESVFRFGA
DVRAGDDPREAQRRLIRDGEAMPIENHLQLNDETEENQPDSGEEE
>P28181
MRHTDRFVKKVVIERIGDQRVLAEEEDVVIKEERISLYLNGTKLMSMMSLPSDQDAHAVG
FLMSEGVIEKIEDLKSVQISSDGSSVYVEALINHENITNLFKEKTLTSGCCVGVTGNLEG
NVLRKFIATPMQISLERIWEGMEEFEMSSHLFHETGCVHKASLLLEDGSKITAEDIGRHN
AIDKVMGKARLGRIDTEKAVLVVSGRLSMEMVVKAVMHNIPMIVSRAAATFLGIKTAQEL
GVTLVGFARGEKMNIYTHSGRVDLRACKRKRGVTLHAPNQSSSLLR
>P69330
MKINQPAVAGTLESGDVMIRIAPLDTQDIDLQINSSVEKQFGDAIRTTILDVLARYNVRG
VQLNVDDKGALDCILRARLEALLARASGIPALPWEDCQ
>P32055
MSKQRVFIAGHRGMVGSAIRRQLEQRGDVELVLRTRDELNLLDSRAVHDFFASERIDQVY
LAAAKVGGIVANNTYPADFIYQNMMIESNIIHAAHQNDVNKLLFLGSSCIYPKLAKQPMA
ESELLQGTLEPTNEPYAIAKIAGIKLCESYNRQYGRDYRSVMPTNLYGPHDNFHPSNSHV
IPALLRRFHEATAQNAPDVVVWGSGTPMREFLHVDDMAAASIHVMELAHEVWLENTQPML
SHINVGTGVDCTIRELAQTIAKVVGYKGRVVFDASKPDGTPRKLLDVTRLHQLGWYHEIS
LEAGLASTYQWFLENQDRFRG
>P0A9P0
MSTEIKTQVVVLGAGPAGYSAAFRCADLGLETVIVERYNTLGGVCLNVGCIPSKALLHVA
KVIEEAKALAEHGIVFGEPKTDIDKIRTWKEKVINQLTGGLAGMAKGRKVKVVNGLGKFT
GANTLEVEGENGKTVINFDNAIIAAGSRPIQLPFIPHEDPRIWDSTDALELKEVPERLLV
MGGGIIGLEMGTVYHALGSQIDVVEMFDQVIPAADKDIVKVFTKRISKKFNLMLETKVTA
VEAKEDGIYVTMEGKKAPAEPQRYDAVLVAIGRVPNGKNLDAGKAGVEVDDRGFIRVDKQ
LRTNVPHIFAIGDIVGQPMLAHKGVHEGHVAAEVIAGKKHYFDPKVIPSIAYTEPEVAWV
GLTEKEAKEKGISYETATFPWAASGRAIASDCADGMTKLIFDKESHRVIGGAIVGTNGGE

LLGEIGLAIEMGCDAEDIALTIHAHPTLHESVGLAAEVFEGSITDLPNPKAKKK
>P63224
MYQDLIRNELNEAAETLANFLKDDANIHAIQRAAVLLADSFKAGGKVLSCGNGGSHCDAM
HFAEELTGRYRENRPGYPAIAISDVSHISCVGNDFGFNDIFSRYVEAVGREGDVLLGIST
SGNSANVIKAIAAAREKGMKVITLTGKDGGKMAGTADIEIRVPHFGYADRIQEIHIKVIH
ILIQLIEKEMVK
>P06614
MKLQQLRYIVEVVNHNLNVSSTAEGLYTSQPGISKQVRMLEDELGIQIFARSGKHLTQVT
PAGQEIIRIAREVLSKVDAIKSVAGEHTWPDKGSLYIATTHTQARYALPGVIKGFIERYP
RVSLHMHQGSPTQIAEAVSKGNADFAIATEALHLYDDLVMLPCYHWNRSIVVTPDHPLAA
TSSVTIEALAQYPLVTYTFGFTGRSELDTAFNRAGLTPRIVFTATDADVIKTYVRLGLGV
GVIASMAVDPLADPDLVRIDAHDIFSHSTTKIGFRRSTFLRSYMYDFIQRFAPHLTRDVV
DTAVALRSNEEIEAMFQDIKLPEK
>P26602
MSHPALTQLRALRYCKEIPALDPQLLDWLLLEDSMTKRFEQQGKTVSVTMIREGFVEQNE
IPEELPLLPKESRYWLREILLCADGEPWLAGRTVVPVSTLSGPELALQKLGKTPLGRYLF
TSSTLTRDFIEIGRDAGLWGRRSRLRLSGKPLLLTELFLPASPLY
>A5VT73
MSDLEQLERQILEDIAAAVDEQGIEAVRVAALGKKGTVSEKLKTLGGMSPEERQMQGPAI
NGLKNRVTEALSERRTELRKAAVAARLEREKVDVTLPVRESAASRGRIHPISQVIDEITA
IFADMGFSIAEGPDIETDYYNFTALNFPEGHPAREMHDTFFFNPDEKGERKLLRTHTSPV
QVHTMEKFAAMRDKEGRDEPIRIVIPGKTYRMDSDATHSPMFHQVEGLVVDKSANVVNMK
WVLEEFCKAFFEVPSVKMRMRPSFFPFTEPSVEVDIQCDRSGPHVKFGEGNDWLEILGCG
MVHPNVLRMSGYDPEVYQGFAWGMGIDRIAMLKYGMPDLRAFFDADVRWIEHYGFRPLDI
PTLFGGLSA
>P13000
MSKRYFVTGTDTEVGKTVASCALLQAAKAAGYRTAGYKPVASGSEKTPEGLRNSDALALQ
RNSSLQLDYATVNPYTFAEPTSPHIISAQEGRPIESLVMSAGLRALEQQADWVLVEGAGG
WFTPLSDTFTFADWVTQEQLPVILVVGVKLGCINHAMLTAQVIQHAGLTLAGWVANDVTP
PGKRHAEYMTTLTRMIPAPLLGEIPWLAENPENAATGKYINLALL
>P0A9G8
MQAEILLTLKLQQKLFADPRRISLLKHIALSGSISQGAKDAGISYKSAWDAINEMNQLSE
HILVERATGGKGGGGAVLTRYGQRLIQLYDLLAQIQQKAFDVLSDDDALPLNSLLAAISR
FSLQTSARNQWFGTITARDHDDVQQHVDVLLADGKTRLKVAITAQSGARLGLDEGKEVLI
LLKAPWVGITQDEAVAQNADNQLPGIISHIERGAEQCEVLMALPDGQTLCATVPVNEATS
LQQGQNVTAYFNADSVIIATLC
>P00944
MQAYFDQLDRVRYEGSKSSNPLAFRHYNPDELVLGKRMEEHLRFAACYWHTFCWNGADMF
GVGAFNRPWQQPGEALALAKRKADVAFEFFHKLHVPFYCFHDVDVSPEGASLKEYINNFA
QMVDVLAGKQEESGVKLLWGTANCFTNPRYGAGAATNPDPEVFSWAATQVVTAMEATHKL
GGENYVLWGGREGYETLLNTDLRQEREQLGRFMQMVVEHKHKIGFQGTLLIEPKPQEPTK
HQYDYDAATVYGFLKQFGLEKEIKLNIEANHATLAGHSFHHEIATAIALGLFGSVDANRG
DAQLGWDTDQFPNSVEENALVMYEILKAGGFTTGGLNFDAKVRRQSTDKYDLFYGHIGAM
DTMALALKIAARMIEDGELDKRIAQRYSGWNSELGQQILKGQMSLADLAKYAQEHHLSPV

HQSGRQEQLENLVNHYLFDK
>Q46604
MLPGTFFEVLKNEGVVAIATQGEDGPHLVNTWNSYLKVLDGNRIVVPVGGMHKTEANVAR
DERVLMTLGSRKVAGRNGPGTGFLIRGSAAFRTDGPEFEAIARFKWARAALVITVVSAEQ
TL
>P0A1J9
MSNLSGTDKSVILLMTIGEDRAAEVFKHLSTREVQALSTAMANVRQISNKQLTDVLSEFE
QEAEQFAALNINANEYLRSVLVKALGEERASSLLEDILETRDTTSGIETLNFMEPQSAAD
LIRDEHPQIIATILVHLKRSQAADILALFDERLRHDVMLRIATFGGVQPAALAELTEVLN
GLLDGQNLKRSKMGGVRTAAEIINLMKTQQEEAVITAVREFDGELAQKIIDEMFLFENLV
DVDDRSIQRLLQEVDSESLLIALKGAEPPLREKFLRNMSQRAADILRDDLANRGPVRLSQ
VENEQKAILLIVRRLAETGEMVIGSGEDTYV
>P0A9F6
MSKRLPPLNALRVFDAAARHLSFTRAAEELFVTQAAVSHQIKSLEDFLGLKLFRRRNRSL
LLTEEGQSYFLDIKEIFSQLTEATRKLQARSAKGALTVSLLPSFAIHWLVPRLSSFNSAY
PGIDVRIQAVDRQEDKLADDVDVAIFYGRGNWPGLRVEKLYAEYLLPVCSPLLLTGEKPL
KTPEDLAKHTLLHDASRRDWQTYTRQLGLNHINVQQGPIFSHSAMVLQAAIHGQGVALAN
NVMAQSEIEAGRLVCPFNDVLVSKNAFYLVCHDSQAELGKIAAFRQWILAKAAAEQEKFR
FRYEQ
>Q1Q845
MMQPSTSTNSIRPVRTRIAPSPTGFPHVGTAYIALFNLAFAKAHGGEFILRIEDTDQTRS
TEQSEKMILDALRWVGLDWAEGPDIGGPHAPYRQSERSDIYKKHAEQLIENDHAFRCFCS
SEELDAMRAEQMANGETPRYDGRCAHLAAEKTEQLVAEGKPHVIRMRVPTEGVCQVQDML
RGTVEIPWTQVDMQVLLKTDGMPTYHLANVVDDHLMDISHVMRGEEWLNSAPKHQLLYEY
FGWEMPVLCHMPLLRNPDKSKLSKRKNPTSITYYRDAGVLPEALLNYLGRMGYSMPDEAE
QFTLEEMIASFDIQRVSLGGPIFDIEKLNWLNSEWLRALTPEELKNKILEWASNSDKLTA
IAAAIQPRIELLSDAVNWSGFYFQNLPDINAESFTHKSLTPEQIMDMLQLSLWQLEVLPT
WSEENIYATLKGLAAHLDIKMRDFMAPFFIAIAGSTSSTPVMNSMAIIGADMTLTRLRHA
VDVLGGLGKKKLKKLEKQAAELPDFLAAE
>Q2QCI9
MAGINGAGPSGAYFVGHTDPEPASGGAHGSSSGASSSNSPRLPAPPDAPASQARDRREML
LRARPLSRQTREWVAQGMPPTAEAGVPIRPQESAEAAAPQARAEERHTPEADAAASHVRT
EGGRTPQALAGTSPRHTGAVPHANRIVQQLVDAGADLAGINTMIDNAMRRHAIALPSRTV
QSILIEHFPHLLAGELISGSELATAFRAALRREVRQQEASAPPRTAARSSVRTPERSTVP
PTSTESSSGSNQRTLLGRFAGLMTPNQRRPSSASNASASQRPVDRSPPRVNQVPTGANRV
VMRNHGNNEADAALQGLAQQGVDMEDLRAALERHILHRRPIPMDIAYALQGVGIAPSIDT
GESLMENPLMNLSVALHRALGPRPARAQAPRPAVPVAPATVSRRPDSARATRLQVIPARE
DYENNVAYGVRLLSLNPGAGVRETVAAFVNNRYERQAVVADIRAALNLSKQFNKLRTVSK
ADAASNKPGFKDLADHPDDATQCLFGEELSLTSSVQQVIGLAGKATDMSESYSREANKDL
VFMDMKKLAQFLAGKPEHPMTRETLNAENIAKYAFRIVP
>P08622
MAKQDYYEILGVSKTAEEREIRKAYKRLAMKYHPDRNQGDKEAEAKFKEIKEAYEVLTDS
QKRAAYDQYGHAAFEQGGMGGGGFGGGADFSDIFGDVFGDIFGGGRGRQRAARGADLRYN
MELTLEEAVRGVTKEIRIPTLEECDVCHGSGAKPGTQPQTCPTCHGSGQVQMRQGFFAVQ

QTCPHCQGRGTLIKDPCNKCHGHGRVERSKTLSVKIPAGVDTGDRIRLAGEGEAGEHGAP
AGDLYVQVQVKQHPIFEREGNNLYCEVPINFAMAALGGEIEVPTLDGRVKLKVPGETQTG
KLFRMRGKGVKSVRGGAQGDLLCRVVVETPVGLNERQKQLLQELQESFGGPTGEHNSPRS
KSFFDGVKKFFDDLTR
>P44442
MSKWDSNIPFLFFSNKKITMIKECQNPPHFRVVTDNTALLEVCNLAQQKSAVALDTEFMR
VSTYFPKLGLIQLYDGEHVSLIDPLAITDFSPFVALLANPKVLKILHSCSEDLLVFLQEF
DQLPRPMIDTQIMARFLGLGTSAGLAKLAQQYLNVEIDKGATRTNWIKRPLSDIQLQYAA
GDVWYLLPLYHILEKELAKTPWEQAVRDDCELVLAKTHKLQERDSEKAYLDIPNAWKLNP
LELSRLRVLAQWRQNVAIERDLALSYIVKSEHLWKVAKNNPRNTSEMLEMGLTENEVRVR
GKEILQLLSQARRISSNDYPKSIERISEDPRYKKTIRLLQEKVNSLTPEGLTPEIVASKR
TLEELIKWVWKYDCSQDKRPELLIGWRKPIGEKLVDALK
>P0A8U6
MAEWSGEYISPYAEHGKKSEQVKKITVSIPLKVLKILTDERTRRQVNNLRHATNSELLCE
AFLHAFTGQPLPDDADLRKERSDEIPEAAKEIMREMGINPETWEY
>Q56019
MVNDASSISRSGYTQNPRLAEAAFEGVRKNTDFLKAADKAFKDVVATKAGDLKAGTKSGE
SAINTVGLKPPTDAAREKLSSEGQLTLLLGKLMTLLGDVSLSQLESRLAVWQAMIESQKE
MGIQVSKEFQTALGEAQEATDLYEASIKKTDTAKSVYDAATKKLTQAQNKLQSLDPADPG
YAQAEAAVEQAGKEATEAKEALDKATDATVKAGTDAKAKAEKADNILTKFQGTANAASQN
QVSQGEQDNLSNVARLTMLMAMFIEIVGKNTEESLQNDLALFNALQEGRQAEMEKKSAEF
QEETRKAEETNRIMGCIGKVLGALLTIVSVVAAVFTGGASLALAAVGLAVMVADEIVKAA
TGVSFIQQALNPIMEHVLKPLMELIGKAITKALEGLGVDKKTAEMAGSIVGAIVAAIAMV
AVIVVVAVVGKGAAAKLGNALSKMMGETIKKLVPNVLKQLAQNGSKLFTQGMQRITSGLG
NVGSKMGLQTNALSKELVGNTLNKVALGMEVTNTAAQSAGGVAEGVFIKNASEALADFML
ARFAMDQIQQWLKQSVEIFGENQKVTAELQKAMSSAVQQNADASRFILRQSRA
>P55818
MSKKLLFQFDTDATPSVFDVVVGYDGGADHITGYGNVTPDNVGAYVDGTIYTRGGKEKQS
TAIFVGGGDMAAGERVFEAVKKRFFGPFRVSCMLDSNGSNTTAAAGVALVVKAAGGSVKG
KKAVVLAGTGPVGMRSAALLAGEGAEVVLCGRKLDKAQAAADSVNKRFKVNVTAAETADD
ASRAEAVKGAHFVFTAGAIGLELLPQAAWQNESSIEIVADYNAQPPLGIGGIDATDKGKE
YGGKRAFGALGIGGLKLKLHRACIAKLFESSEGVFDAEEIYKLAKEMA
>P32173
MNLMTTITGVVLAGGKARRMGGVDKGLLELNGKPLWQHVADALMTQLSHVVVNANRHQEI
YQASGLKVIEDSLADYPGPLAGMLSVMQQEAGEWFLFCPCDTPYIPPDLAARLNHQRKDA
PVVWVHDGERDHPTIALVNRAIEPLLLEYLQAGERRVMVFMRLAGGHAVDFSDHKDAFVN
VNTPEELARWQEKR
>Q51434
MQLIQELSQARDRGLYQEVGKLTRELHNAIVDFQIDPHSPHAQEMSQIADATDRLSYVVE
MTEKAANRTMDLVEQSAPLVNQLGDDSRELHQEWQRFMRREIDADGFRELAKRIEQFLVR
SGENAGQLSSQLNDILLAQDYQDLTGQVIKRVTKLVTEVESNLVKLVWMAGQVDRYAGIE
HDHVSMRHQAALERSAKGEGPQVAAEKREDVVSGQDDVDDLLSSLGF
>Q59516
MTKKVVFLDRESLDATVREFNFPHEYKEYESTWTPEEIVERLQGAEIAMINKVPMRADTL

KQLPDLKLIAVAATGTDVVDKAAAKAQGITVVNIRNYAFNTVPEHVVGLMFALRRAIVPY
ANSVRRGDWNKSKQFCYFDYPIYDIAGSTLGIIGYGALGKSIAKRAEALGMKVLAFDVFP
QDGLVDLETILTQSDVITLHVPLTPDTKNMIGAEQLKKMKRSAILINTARGGLVDEAALL
QALKDGTIGGAGFDVVAQEPPKDGNILCDADLPNLIVTPHVAWASKEAMQILADQLVDNV
EAFVAGKPQNVVEA
>Q72ER1
MTENKTKVKDLAAELGVTTKELGQVLKDMNISAKTSTSVIAQEDLPRIKERVQAQRDGGA
RKEGNPDVIVRRRHRDGDRASARAEAKAPEQEATAAMPETSAPERAEEADKPAVAKPAKA
PETEAHARARKEPQAEPVKARIIRRPDEPAPVAKVVEAAPAETPAPEAPAVKATVTAEAA
PAKTVEPESERPQADKPATARVVRPATPDASAVPDGTSSAPTLPVRSAEPSDTVERADAD
ADGDDDDAQQRRRKKKRRQPEAVVPQVRVISRPDPAAVAQQQMQQQAAQQQREAGGYRPG
GQRPEGGYRPEGQREGGYRPEGQREGGYRPGGAPRPEGGYRPGGPRPEGGYRPGAPRPEG
GYRPAGGPRPEGQREGGYRPGAPRPEGGYRPAGGAPRPEGQREGGYRPAGGPPRPGGAPR
PGGFGGAPGGMPVPGADGRGDQSKKKRQKGRRTVDFQADGPRGRSDDDVMRGPRGRGKRG
KKDVRPAATQPLKAVKRKIKVDEAIRVADMAHQMGLKANEIIKVLFGLGVMATINQSLDI
DTATVVAGEFGYEVEKVGFSEDDYLVPKEEDAPETLVTRPPVVTIMGHVDHGKTSLLDAI
RKSNVTAGEAGGITQHIGAYHVTTKKGEIVFLDTPGHEAFTAMRARGAQITDLVVLVVAA
DDGVMEQTREAVNHSKAAGVPIMVAVNKMDKEGANPDRVIRELSELGLVAEDWGGDTIFA
KVSAKTREGLDELLELIAIQAEILELKANPDKAARGHVVEAKLDKGRGPLATVLVQEGTL
RQGDAFVCGVFAGRVRAMFDDQGRKVKEAGPSTPVEVQGFDGVVEAGEEFVSVADDKVAR
RIAESRAVKQRERELAKESKVTLETFLSRRADAAEALTLNLVLKADVQGTLEAISEAVRK
LSTEKVKINIIHGGAGAITESDILLASASDAIIIGFNVRPTSKVKDIAEQENVDIRFYDI
IYKLVDEIKSAMAGMLAPVQREVYLGQAEVRETFSVPKIGVIAGCHVADGKVTRNAGVRL
LRDGVVVYTGKITSLKRFKDDVRDVQKGYECGMGLENFNDIKVGDVIEAFEMVEEAATL
>P81433
MELKNHKKIIILGSGPAGYTAAIYSSRANLNPLLITGINKGGQLMNTNEIENWPGDFKKI
TGPELMNRMHEHSLKFKTEIVYDNIISVEFKKKPFFLLGEYNKYTCDAVIIATGANPRYL
GLSSENKFKGKGISTCAVCDGFFYKNKEIAVVGGGNTAIEETLYLSNFVKKIYLIHRRNN
FKAEKILIDRLLKIVKTKKVILHLNSTIEDILGNNKGVTHLLIKNKNLKEKKKLKIAVSG
LFVAIGYIPNTDIFTDQLKMKDGYIKIKKGTHGNYTQTNIPGVFAAGDVIDHVYRQAITS
SASGCMAALDSERYLNSLS
>Q9X758
MLNIAALRQQQIPLAAEPRSPVPFHILMKPIGPACNLACRYCYYPQDETPVNKMDDARLE
QFIRRYIAAQPAGAREINFVWQGGEPLLAGLSFYKKALALQARYAPDGVTISNSLQTNGT
LINDAWCRLFREHGFIIGLGLEGNEALQDYHRPDKRGRSTWSAALRGIDLLHQHQVDFNL
LVVVHNEMAAHAAAIYVRLVSLGARYLQFQPLMSEGAALREGYQLSADNWGRFMVGIWRQ
WRKRCDRGRVFVINIEQAWAQYFTHTSGSCVHSARCGSNLVMESDGQLYACDHLINTEHR
LGRLDEQTLAAAVDASVQLPFGQQKSLRRECQTCSVKMVCQGGCPAHLNAAGNNRLCGGY
YRFFSDILAPLRPFSRDLNGLKAWRAAFVGTAHTA

(4) $\mathbb{S}_4$: 133 extracell proteins

>Q9ZEU2
MLTPTQQVGLILQYLKTRILDIYTPEQRAGIEKSEDWRQFSRRMDTHFPKLMNELDSVYG
NNEALLPMLEMLLAQAWQSYSQRNSSLKDIDIARENNPDWILSNKQVGGVCYVDLFAGDL

KGLKDKIPYFQELGLTYLHLMPLFKCPEGKSDGGYAVSSYRDVNPALGTIGDLREVIAAL
HEAGISAVVDFIFNHTSNEHEWAQRCAAGDPLFDNFYYIFPDRRMPDQYDRTLREIFPDQ
HPGGFSQLEDGRWVWTTFNSFQWDLNYSNPWVFRAMAGEMLFLANLGVDILRMDAVAFIW
KQMGTSCENLPQAHALIRAFNAVMRIAAPAVFFKSEAIVHPDQVVQYIGQDECQIGYNPL
QMALLWNTLATREVNLLHQALTYRHNLPEHTAWVNYVRSHDDIGWTFADEDAAYLGISGY
DHRQFLNRFFVNRFDGSFARGVPFQYNPSTGDCRVSGTAAALVGLAQDDPHAVDRIKLLY
SIALSTGGLPLIYLGDEVGTLNDDDWSQDSNKSDDSRWAHRPRYNEALYAQRNDPSTAAG
QIYQGLRHMIAVRQSNPRFDGGRLVTFNTNNKHIIGYIRNNALLAFGNFSEYPQTVTAHT
LQAMPFKAHDLIGGKTVSLNQDLTLQPYQVMWLEIA
>P61416
MSRIITAPHIGIEKLSAISLEELSCGLPERYALPPDGHPVEPHLERLYPTAQSKRSLWDF
ASPGYTFHGLHRAQDYRRELDTLQSLLTTSQSSELQAAAALLKCQQDDDRLLQIILNLLH
KV
>P37773
MRIHILGICGTFMGGLAMLARQLGHEVTGSDANVYPPMSTLLEKQGIELIQGYDASQLEP
QPDLVIIGNAMTRGNPCVEAVLEKNIPYMSGPQWLHDFVLRDRWVLAVAGTHGKTTTAGM
ATWILEQCGYKPGFVIGGVPGNFEVSAHLGESDFFVIEADEYDCAFFDKRSKFVHYCPRT
LIILNNLEFDHADIFDDLKAIQKQFHHLVRIVPGQGRIIWPENDINLKQTMAMGCWSEQEL
VGEQGHWQAKKLTTDASEWEVLLDGEKVGEVKWSLVGEHNMHNGLMAIAAARHVGVAPAD
AANALGSFINARRRLELRGEANGVTVYDDFAHHPTAILATLAALRGKVGGTARIIAVLEP
RSNTMKMGICKDDLAPSLGRADEVFLLQPAHIPWQVAEVAEACVQPAHWSGDVDTLADMV
VKTAQPGDHILVMSNGGFGGIHQKLLDGLAKKAEAAQ
>Q46669
MKKYIISLIVFLSFYAQADLTDFRVATWNLQGASATTESKWNINVRQLISGENAVDILAV
QEAGSPPSTAVDTGTLIPSPGIPVRELIWNLSTNSRPQQVYIYFSAVDALGGRVNLALVS
NRRADEVFVLSPVRQGGRPLLGIRIGNDAFFTAHAIAMRNNDAPALVEEVYNFFRDSRDP
VHQALNWMILGDFNREPADLEMNLTVPVRRASEIISPAAATQTSQRTLDYAVAGNSVAFR
PSPLQAGIVYGARRTQISSDHFPVGVSRR
>Q99289
MMKKTITLLTALLPLASAVAEEPTLSPEMVSASEVISTQENQTYTYVRCWYRTSYSKDDP
ATDWEWAKNEDGSYFTIDGYWWSSVSFKNMFYTNTSQNVIRQRCEATLDLANENADITFF
AADNRFSYNHTIWSNDAAMQPDQINKVVALGDSLSDTGNIFNASQWRFPNPNSWFLGHFS
NGFVWTEYIAKAKNLPLYNWAVGGAAGENQYIALTGVGEQVSSYLTYAKLAKNYKPANTL
FTLEFGLNDFMNYNRGVPEVKADYAEALIRLTDAGAKNFMLMTLPDATKAPQFKYSTQEE
IDKIRAKVLEMNEFIKAQAMYYKAQGYNITLFDTHALFETLTSAPEEHGFVNASDPCLDI
NRSSSVDYMYTHALRSECAASGAEKFVFWDVTHPTTATHRYVAEKMLESSNNLAEYRF
>P20041
MNHRYTLLALAAAALSAGAHATGTSVTAPWGEVAEPSLPADSAVCKTLSASITPIKGSVD
SVDGNPANSQPDASRIQSAIDNCPAGQAVKLVKGSAGESGFLSGSLKLKSGVTLWIDTGV
TLFASRNPADYDNGLGTCGTATTSNDKSCNALIVARDTAGSGIVGAGAIDGRGGSLVTSG
PNANRLTWWDIAYLNKTKGLNQQNPRLIQTYNGSAFTLYGVTVQNSPNFHIVTTGTSGVT
AWGIKIVTPSLAYAVAGYKCPSGSTPDKVTPATCFTPETVKNTDGFDPGQSTNVVLAYSY
INTGDDHVAVKASSGPTRNLLFAHNHFYYGHGLSIGSETNTGVSNMLVTDLTMDGNDSSA
GNGLRIKSDASRGGKVTNIVYDGICMRNVKEPLVFDPFYSSVKGSLYPNFTNIVVKNFHD

LGSAKSIKRTMTFLGYKANKQKNPLTITLDNVVFDGTLPAFEGSHYGGPASPNGVHFTFG
GTGPVSFADAIVTSSTTDVTVTGTPGTAAAVDCSKAFVPLKSVAPTSPI
>P33693
MTIDRYRRFARLAFIATLPLAGLATAAAAQEGANGKSFKDDFDTLDTRVWFVSDGWNNGG
HQNCTWSKKQVKTVDGILELTFEEKKVKERNFACGEIQTRKRFGYGTYEARIKAADGSGL
NSAFFTYIGPADKKPHDEIDFEVLGKNTAKVQINQYVSAKGGNEFLADVPGGANQGFNDY
AFVWEKNRIRYYVNGELVHEVTDPAKIPVNAQKIFFSLWGTDTLTDWMGTFSYKEPTKLQ
VDRVAFTAAGDECQFAESVACQLERAQSE
>P74863
MSEEGFMLAVLKGIPLIQDIRAEGNSRSWIMTIDGHPARGEIFSEAFSISLFLNDLESLP
KPCLAYVTLLLAAHPDVHDYAIQLTADGGWLNGYYTTSSSSELIAIEIEKHLALTCILKN
VIRNHHKLYSGGV
>P35483
MTPGYPLALSLAVSMAVLGSALPAQARQDDPSLFNRQARGELSEYGGARRVEQDLTQALK
QSLSKKKAKNVILLIGDGMGDSEITVARNYARGAGGYFKGIDALPLTGQYTHYSLHKDSG
LPDYVTDSAASATAWSTGVKSYNGAIGVDIHEQPHRNLLELAKLNGKATGNVSTAELQDA
TPAALLAHVTARKCYGPEATSKQCPSNALENGGAGSITEQWLKTRPDVVLGGGAATFAET
AKAGRYAGKTLRAQAEARGYRIVENLDELKAVRRANQKQPLIGLFAPGNMPVRWLGPTAT
YHGNLNQPAVSCEANPKRTADIPTLAQMTSKAIELLKDNPNGFFLQVEGASIDKQDHAAN
PCGQIGETVDLDEAVQKALAFAKADGETLVIVTADHAHSSQIIPPETAAPGLTQLLTTKD
GAPLAISYGNSEESSQEHTGTQLRIAAYGPQAANVTGLTDQTDLFFTIRRALNLRD
>Q9RBS0
MSVGNIQSPSNLPGLQNLNLNTNTNSQQSGQSVQDLIKQVEKDILNIIAALVQKAAQSAG
GNTGNTGNAPAKDGNANAGANDPSKNDPSKSQGPQSANKTGNVDDANNQDPMQALMQLLE
DLVKLLKAALHMQQPGGNDKGNGVGGANGAKGAGGQGGLAEALQEIEQILAQLGGGGAGA
GGAGGGVGGAGGADGGSGAGGAGGANGADGGNGVNGNQANGPQNAGDVNGANGADDGSED
QGGLTGVLQKLMKILNALVQMMQQGGLGGGNQAQGGSKGAGNASPASGANPGANQPGSAD
DQSSGQNNLQSQIMDVVKEVVQILQQMLAAQNGGSQQSTSTQPM
>Q9R9R6
MAITSAGAGSGIDLESVISASVSAKKAQLQQPIITKQNSTQITLSGIGQLKSSISAFTDI
LDKLSAPGAFNKRAINITQSKDDPILKVEGKSGASNGQYNIIVNKLAETSRQEGIFDSST
TPLATQDGQLTFKAGDKTFKVDVKAGDTLQDIRKSINSNGDNFGLSVNIVNTADGKAKLV
IDSGISGDGKDLTITGDNAELGVFEAGGGVMSQTRAASSAEINVDGNVLKSDTNTFDDSI
QDLKVTVLRVSDKDSAGDLKANKVDITTDKTSIQELVQQFVDGYNTLQDKMNSLGKRNTF
VGGVKQDDGGALAGDSTTRAIESFMSNLLVSPSQNSGTYSTVFEIGIKMDNKGKLSLDKT
KFGEAVDKNFDQVVALFGGEKGLASTLNSGLKEYTKSGGMLAQREDVLNSDLRALTQKTA
TANAQLTKYEAALRAQYGSLDALLVKMNSSASALATLQTSYQKS
>P33546
MSLKISNFIDASNTKGPIRVEDTEHGPILIAQKFNLKDLFFRTLSTINAKINSQILNEQL
KNYRLENQKSLLLFLNTLASEKSAESAFAAYEAAKNSIQHSFTGRDIKLMLNTAERFHGI
GTAKNLERHLVFRCWGNRGITHLGHTSISIKNNLLQEPTHTYLSWYPGGNVTKDTEINYL
FEKRSGYSVDTYKQDKLNMISEQTAERLDAGQEVRNLLNSKQDQNNNKKIFFPRANQKKD
PYGYWGVSADKVYIPLSGDNKTKDGKISHNLFGLDETNMSKFICKKKADAFRQLANYKLI
SKSENCAGMALNVLKAGNSEIYFPLPDVKLVATPNDVYAYANKVRQRIESLNQSYNEIMK

YIESDFDLSRLTQLRRSYLKSFNKINLIHTPKTFKPLSISLYKHPTENVSSEDFDAVINA
CHSYLVKSAPSNMTRVLNELKTEATDKKEEIIEKSIKIIDYYNSLKSPDLGTKLYIHDLL
QINKLLLNNSHSNI
>P08038
MMIFRFVTTLAASLPLLTFAAPISFSHAKNEAVKIYRDHPVSFYCGCEIRWQGKKGIPDL
ESCGYQVRKNENRASRIEWEHVVPAWQFGHQLQCWQQGGRKNCTRTSPEFNQMEADLHNL
TPAIGEVNGDRSNFSFSQWNGVDGVTYGQCEMQVNFKERTAMPPERARGAIARTYLYMSE
QYGLRLSKAQSQLMQAWNNQYPVSEWECVRDQRIEKVQGNSNRFVREQCPN
>O06949
MTKITLFPHNFRIQKQEATPLKEKSTEKNSLAKSILAVKNHFIKLNSKLSERFISHKNTE
SSATHFHRGSASEGRAVLTNKVVKNFMLQTLHDIDIRGSASKDPAYASQTREAILSAVYS
KYKDQYCNLLISKGIDIAPFLKEIGEAAQNAGLPGATKNDVFSPSGAGANPFITPLITSA
YSKYPHMFTSQHQKASFNIYAEKIIMTEVVPLFNECAMPTPQQFQQILENIANKYIQNTP

>P22522
MRTLTLNELDSVSGGASGRDIAMAIGTLSGQFVAGGIGAAAGGVAGGAIYDYASTHKPNP
AMSPSGLGGTIKQKPEGIPSEAWNYAAGRLCNWSPNNLSDVCL
>Q8RP17
MSPAQIIRTPHSFPPSFTGTSSSAENSHAQSPQQVLTRAFVASGELNAAFGRTSTASEQD
FTSLLGTLQRELERKTLSFPDIAELANQLAEAAKGDQGGHWLGRDEQQTLKGMIDRCKSQ
LAHTHASDASYDPLAQVCENLKTARLHQSISQMTGEAHAKVRGVPDLLALIQLDPDVLAE
KPVGMTSYVNFGSFICMAKARTAELSEDLRSDPNEVALLLHPHADTILELERLPDALAAL
TENCPDTPTRDDLRSLAKETGELLQQLRANDLLPRSEEVSSYQGETSVRSREVVEPKLTL
CQAGGNGQGQLEASSARPESLRYAPTRAASSGSEARVPGQAVGGKIADDAQKVAGLYAEK
KRTNWTQANGVAGKISHKIQSLLGMRDAGSRVQAFVAFMADGKGRPGATMLDLGDGWMRA
TRVIKGEAALIDFQCDSDGKVVDARHPGRFPVLPQGNEREAFKTVLQELKFRGAETLSKV
PVYYVNRNTRGYVIPTHGYVVAGHPNRGRKSGAVLYGVGGDPKRGPVALDEKLLGHLVGR
SDSKTSSKLSAPVKAAISALAGASFATREDFYDAYCAVRGDAVDPLERHNEISSIYRLLP
LSTMEMWPKKADDYRVARPAAPERDLRAFENLPKDIGRKAQLKKVSNVDSIDLLEAKRQF
TLHQLYQDEMLGRNGTGVPSADFKPKVDAQRRDQLVASTPKFQRLPPHTTDKVGNCNTGA
SSLLQRAVDTYTEKNNLPPEKVTAASIFGIGSSHRLAIWDPLDGSSSNKSSKDR
>Q05489
MVRSMRSRVAARAVAWALAVMPLAGAAGLTMAASPAAVAADTYAATRYPVILVHGLAGTD
KFANVVDYWYGIQSDLQSHGAKVYYVANLSGFQSDDGPNGRGEQLLAYVKQVLAATGATKV
NLIGHSQGGLTSRYVAAVAPQLVASVTTIGTPHRGSEFADFVQDVLKTDPTGLSSTVIAA
FVNVFGTLVSSSHNTDQDALAALRTLTTAQTATYNRNFPSAGLGAPGSCQTGAATETVGG
SQHLLYSWGGTAIQPTSTVLGVTGATDTSTGTLDVANVTDPSTLALLATGAVMINRASGQ
NDGLVSRCSSLFGQVISTSYHWNHLDEINQLLGVRGANAEDPVAVIRTHVNRLKLQGV
>P0C1A7
MKYLNCFISTGLAAFFLVNSTSVLAADCSSDLTSGISTKRIYYVAPNGNSSNNGSSFNAP
MSFSAAMAAVNPGELILLKPGTYTIPYTQGKGNTITFNKSGKDGAPIYVAAANCGRAVFD
FSFPDSQWVQASYGFYVTGDYWYFKGVEVTRAGYQGAYVIGSHNTFENTAFHHNRNTGLE
INNGGSYNTVINSDAYRNYDPKKNGSMADGFGPKQKQGPGNRFVGCRAWENSDDGFDLFD
SPQKVVIENSWAFRNGINYWNDSAFAGNGNGFKLGGNQAVGNHRITRSVAFGNVSKGFDQ

NNNAGGVTVINNTSYKNGINYGFGSNVQSGQKHYFRNNVSLSASVTVSNADAKSNSWDTG
PAASASDFVSLDTSLATVSRDNDGTLPETSLFRLSANSKLINAGTKESNISYSGSAPDLG
AFERN
>Q06517
MPAQRMRSVIPPYMLRALLTRYAPQRDCALHTLNHVQSLLGNKPLRSPTEKNARAGERSA
ISTTPERHPTARQTGAQGGAAQQPRRAVDEAYDHLGVTYDFFWQAYRRNSVDNKGLPLVQ
RALRQGLPEQLSGTASRWCSETATARSSTVSPSPSTLVGHELTHGSDRERSRLIYYQQSG
ALNESLSDVFGSLVKQFHLQQTADKADWLIGAGLLAKGIKGKGLRSMSAPGTAYDDPLLG
KDPQPASMKDYIQTKEDNGGVHLNSGIPNRAFYLAATVLGGFAGKKPVTSGMTRCATKRC
RKTPTSDHLRPRHGETRAGLRTKRGDKVQQAWASGWQWSNETAADAQSGYGH
>Q9PHW6
MAFGSLSSLGFGSGVLTQDTIDKLKEAEQKARIDPYTKKIEENTTKQKDLTEIKTKLLSF
QTAVSSLADATVFAKRKVVGSISDNPPASLTVNSGVALQSMNINVTQLAQKDVYQSKGLA
NDGGFVNAQLNGTADLTFFSNGKEYTVTVDKNTTYRDLADKINEASGGEIVAKIVNTGEK
GTPYRLTLTSKETGEDSAISFYAGKKDSNGKYQKDINAEKIFDDLGWGLDVSASIDPDKD
KKGYGIKDASLHIQTAQNAEFTLDGIKMFRSSNTVTDLGVGMTLTLNKTGEINFDVQQDF
EGVTKAMQDLVDAYNDLVTNLNAATDYNSETGTKGTLQGISEVNSIRSSILADLFDSQVV
DGTTEDANGNKVNTKVMLSMQDFGLSLNDAGTLSFDSSKFEQKVKEDPDSTESFFSNITK
YEDINHTGEVIKTGSLSKYLNSNGGNTNGLEFKPGDFTIVFNNQTYDLSKNSDGTNFKLT
GKTEEELLQNLANHINSKGIEGLKVKVESYNQNNVTGFRLNFSGDGSSDFSIKGDANILK
ELGLSDVNITSKPIEGKGIFSKLKATLQEMTGKDGSITKYDESLTNDIKSLNTSKDSTQA
MIDTRYDTMANQWLQYESILNKLNQQLNTVTNMINAANNSNN
>Q87W65
MLIGHSLHHMRPTAVDSSLPTSATSQTISNTKSRLDPHRVRELTFIGVGSSVAYLLNELN
GRFADSGVTTPFLGKVSIVGKDDSWAENVRGKGYINHQTEIISQWDQQVPKYDPNYAARA
EFSASNRRQLTRTVELGAEHLKAQVTGISRLDDGCFRINLDNGQILQSRQIVLGTGAGPH
TSIWNSVTSHTQAEKRLDNIKLHEQKALRGKVLDLDEFMRASDASPQTFAGKTVVIHGPN
AGIDAAERAGELGANAVWFTRSTNPVLLDGNQLKFAPELAKSAIHKVDKLDIRPTKLENG
FALRLHYSSLGQDSREPKKVLDADYYVYAMGQDIHKPGSAAAILGSLLDHLEPIYDYDQV
YSDQPFKTVIGLQSRGSNSDNGLIIVGAAVAQLATNVQHSYKDHALDRILEEMTRLPEKQ
TEKLSQMLLEGAPSVQIQTYLKTWQLDSGQPPDKQVLQNQVENYLAARDYFQRQTNEQKG
NLDGVAAEVKNQTLTEVASVIVSPQLGTIKASAAALSGLMPAYVANGENNFTTDNRTMLR
AGIAARYPNIGNAEASAFIDEVVTLRHLNSQRFIEKVAGEMMDKGAQPLVSLRPPVLGVP
ASVRTAYEAYLHALNSGAHDGTPLSQRWLPKK
>Q52430
MKIGTQATSLAVLHNQESHAPQAPIAVRPEPAHAIPEIPLDLAIRPRTRGIHPFLAMTLG
DKGCASSSGVSLEDDSHTQVSLSDFSVASRDVNHNNICAGLSTEWLVMSSDGDAESRMDH
LDYNGEGQSRGSERHQVYNDALRAALSNDDEAPFFTASTAVIEDAGFSLRREPKTVHASG
GSAQLGQTVAHDVAQSGRKHLLSLRFANVQGHAIACSCEGSQFKLFDPNLGEFQSSRSAA
PQLIKGLIDHYNSLNYDVACVNEFRVS
>P13717
MRFNNKMLALAALLFAAQASADTLESIDNCAVGCPTGGSSNVSIVRHAYTLNNNSTTKFA
NWVAYHITKDTPASGKTRNWKTDPALNPADTLAPADYTGANAALKVDRGHQAPLASLAGV
SDWESLNYLSNITPQKSDLNQGAWARLEDQERKLIDRADISSVYTVTGPLYERDMGKLPG

TQKAHTIPSAYWKVIFINNSPAVNHYAAFLFDQNTPKGADFCQFRVTVDEIEKRTGLIIW
AGLPDDVQASLKSKPGVLPELMGCKN
>Q56705
MSLGPVGMSGGMDINSMVSKIVDAERVPKQQRIDNDRTTINASISAYGRLRESLDTMKNL
MANFRQEKAFAVRTVETTDDNIVSATATTDAIAGKYAIDVLQLAQSHKVASDVLPEDAKF
GPGKLQISLGDDRFNIEVRSRSKLIDVVRGINGAKDNPGVRASVINDVEGPRLILASNLS
GKDHQIKVSVEAERGNPLKYFEYQTLEDRVNALEEARAAAEEVLGPLQAPQQPDQPEILD
ENGNPLPPEAQKAADNAQDDAQDDASQEPISAAGAEAAKAGQEAIDKANQRSSLRPEERI
PGWTETASGTLLDSYEEPELELDEKAIEKAPDVPGWNNAASGTLTDSYVTTKEAKQLLEQ
EKAEIEQKIADEKQELDAKVERGELSEEQAKQIHRAKLDPQERERLEKIDEAEAKIAKAQ
SSFEEYLGMTEVQAGQDSEVLLDGVAKLSSHNNVIEDAIEGVDLTLKGKSEPNKPPAEIG
VEYDRQSVRSDIENFVSAYNSFYQTSQALSSVDPTTGQKGPLAGDSTVRSADSRLKAVFS
SRIDQAPENLKSLTEFGITTTRQGTLEINYDMLDRQLNNNFNELEKFFGGNTGFAKRIED
AIHGITGITGSIRTREKSLTEQNYRLNDDQAALDRRMEGLEKRTHAKFTAMQDATGKMQG
QLGALMSALG
>Q8VSP9
MPIKKPCLKLNLDSLNVVRSEIPQMLSANERLKNNFNILYNQIRQYPAYYFKVASNVPTY
SDICQSFSVMYQGFQIVNHSGDVFIHACRENPQSKGDFVGDKFHISIAREQVPLAFQILS
GLLFSEDSPIDKWKITDMNRVSQQSRVGIGAQFTLYVKSDQECSQYSALLLHKIRQFIMC
LESNLLRSKIAPGEYPASDVRPEDWKYVSYRNELRSDRDGSERQEQMLREEPFYRLMIE
>Q888Y8
MNPLRSIQHNIATPPISGGQPLDAVGPQAQQSHPKRISPSQLSQSAHQALERLSANAEHQ
RLASLVRNALQDGTFQFQSSNHTQVTYKASICLPADTDTVRTDHLINNELTVQARLNDQS
EYDIVSAHLHGSSKAISFDVPSPPPAHGSASSVLSERTHLGMSRVLSQDAVDSSSLETPL
LSSPDHSRPPSQPKPVHIGSVRRDSGSLVSDNPVVQALLSFAQADQAFPPQAASIAGVQL
EMRPRRDIEKALEEFKGAFTVVKAQLMSGANSSERVDEDVNADIHIPLLLKAIERGAAAF
GPNASIGQNSAKAFLASCAPKITSNDDVLSEFINQKLKGDDDLQVRLGAQELLHVATKKE
FQLGGLAGSIGVSSILGSAWELGASELLKNAIFGKNFSPSQYALQLAGIDSVPPLIIESM
DTMCVLAIIKGMKGEEWSMSDLLPKALKAGAISSVVSFPNNVLQYAGFKSRVGDLAANSV
TTEAAIFGAASGIPPEVKESEELMRAGLFQSMKDGVMAHSGEGVDTKKTIERMTRHALDI
APGESTAVKSMGLASIVGMIPLIASNKATGLLSEQVLRIFRSAVFNPIEAIALNALALGG
RVNVPGLFDSDNAKHARVVQTILARASQHMEAGDRDISAEELHQMLAPRSEFLRHVGSAI
VNGMNASFEAIPALVRKLGYGEAPLAERIPYQDLAVPDTSRQPAP
>P62530
MREITESQLRYISGAGGAPATSANAAGAAAIVGALAGIPGGPLGVVVGAVSAGLTTAIGS
TVGSGSASSSAGGGS
>Q9X2V7
MIKHFHFNKLSSGKKNNVPSPAKGVIQIKKSASQLTKGGAGHVPEYFVGIGTPISFYG
>P23031
MINHNKTPNILAKVFKRTCGLVSTGAALAILSQAASAACTYTIDSEWSTGFTANITLKND
TGAAINNWNVNWQYSSNRMTSGWNANFSGTNPYNATNMSWNGSIAPGQSISFGLQGEKNG
STAERPTVTGAACNSATTSSVASSSSTPTTSSSSASSVASALLLQEAQAGFCRVDGTIDN
NHTGFTGSGFANTNNAQGAAVVWAIDATSSGRRTLTIRYANGGTANRNGSLVINGGSNGN
YTVSLPTTGAWTTWQTATIDVDLVQGNNIVQLSATTAEGLPNIDSLSVVGGTVRAGNCGS

VSSSSSVQSSSSSSSSTPSQTCELKAPLRWTSTGPLISPKNPGWISIKDPSIVKYNDTYHV
YATYYDTAYRSMYTSFTDWNTAQQAPHISMNGSRVGNTVAPQVFYFRPHNKWYLITQWAG
AYATTDDIRNPNWSAKQKLLQGEPNGALDFWVICNDTHCYLYFSRDDGVLYVSKTTLANF
PNFSGYSIVMEDHRGNGNSYLFEAANVYKLDGQNRYLLMVEAYISGRAFSAPGQRPAWMA
HGPLADTEANPFAGMMFCFTMASSLKVYTCY
>Q9KLD5
MKKQPKMTAIALILSGISGLAYGHGYVSAVENGVAEGRVTLCKFAANGTGEKNTHCGAIQ
YEPQSVEGPDGFPVTGPRDGKIASAESALAAALDEQTADRWVKRPIQAGPQTFEWTFTAN
HVTKDWKYYITKPNWNPNQPLSRDAFDLNPFCVVEGNMVQPPKRVSHECIVPEREGYQVI
LAVWDVGDTAASFYNVIDVKFDGNGPVLPDWNPAGQIIPSMDLSIGDTVYTRVFDNDGEN
PAYRTELKIDSETLTKANQWSYALATKINQTQKQQRAGQLNGDQFVPVYGTNPIYLKEGS
GLKSVEIGYQIEAPQPEYSLTVSGLAKEYEIGEQPIQLDLTLEAQGEMSAELTVYNHHQK
PLASWSQAMTDGELKSITLELSEAKAGHHMLVSRIKDRDGNLQDQQTLDFMLVEPQTPPT
PGDYDFVFPNGLKEYVAGTKVLASDGAIYQCKPWPYSGYCQQWTSNATQYQPGTGSHWEM
AWDKR
>P18010
MHNVNNTQAPTFLYKATSPSSTEYSELKSKISDIHSSQTSLKTPASVSEKENFATSFNQK
CLDFLFSSSGKEDVLRSIYSNSMNAYAKSEILEFSNVLYSLVHQNGLNFENEKGLQKIVA
QYSELIIKDKLSQDSAFGPWSAKNKKLHQLRQNIEHRLALLAQQHTSGEALSLGQKLLNT
EVSSFIKNNILAELKLSNETVSSLKLDDLVDAQAKLAFDSLRNQRKNTIDSKGFGIGKLS
RDLNTVAVFPELLRKVLNDILEDIKDSHPIQDGLPTPPEDMPDGGPTPGANEKTSQPVIH
YHINNDNRTYDNRVFDNRVYDNSYHENPENDAQSPTSQTNDLLSRNGNSLLNPQRALVQK
VTSVLPHSISDTVQTFANNSALEKVFNHTPDNSDGIGSDLLTTSSQERSANNSLSRGHRP
LNIQNSSTTPPLHPEGVTSSNDNSSDTTKSSASLSHRVASQINKFNSNTDSKVLQTDFLS
RNGDTYLTRETIFEASKKVTNSLSNLISLIGTKSGTQERELQEKSKDITKSTTEHRINNK
LKVTDANIRNYVTETNADTIDKNHAIYEKAKEVSSALSKVLSKIDDTSAELLTDDISDLK
NNNDITAENNNIYKAAKDVTTSLSKVLKNINKD
>Q56020
MLISNVGINPAAYLNNHSVENSSQTASQSVSAKDILNSIGISSSKVSDLGLSPTLSAPAP
GVLTQTPGTITSFLKASIQNTDMNQDLNALANNVTTKANEVVQTQLREQQAEVGKFFDIS
GMSSSAVALLAAANTLMLTLNQADSKLSGKLSLVSFDAAKTTASSMMREGMNALSGSISQ
SALQLGITGVGAKLEYKGLQNERGALKHNAAKIDKLTTESHSIKNVLNGQNSVKLGAEGV
DSLKSLNMKKTGTDATKNLNDATLKSNAGTSATESLGIKDSNKQISPEHQAILSKRLESV
ESDIRLEQNTMDMTRIDARKMQMTGDLIMKNSVTVGGIAGASGQYAATQERSEQQISQVN
NRVASTASDEARESSRKSTSLIQEMLKTMESINQSKASALAAIAGNIRA
>P36175
MRILGIETSCDETGVAIYDEDKGLVANQLYSQIDMHADYGGVVPELASRDHIRKTLPLIQ
EALKEANLQPSDIDGIAYTAGPGLVGALLVGSTIARSLAYAWNVPALGVHHMEGHLLAPM
LEENAPEFPFVALLISGGHTQLVKVDGVGQYELLGESIDDAAGEAFDKTGKLLGLDYPAG
VAMSKLAESGTPNRFKFPRPMTDRPGLDFSFSGLKTFAANTIKANLNENGELDEQTKCDI
AHAFQQAVVDTILIKCKRALEQTGYKRLVMAGGVSANKQLRADLAEMMKKLKGEVFYPRP
QFCTDNGAMIAYTGFLRLKTMNKPT
>Q56026
MLNIQNYSASPHPGIVAERPQTPSASEHVETAVVPSTTEHRGTDIISLSQAATKIHQAQQ

TLQSTPPISEENNDERTLARQQLTSSLNALAKSGVSLSAEQNENLRSAFSAPTSALFSAS
PMAQPRTTISDAEIWDMVSQNISAIGDSYLGVYENVVAVYTDFYQAFSDILSKMGGWLLP
GKDGNTVKLDVTSLKNDLNSLVNKYNQINSNTVLFPAQSGSGVKVATEAEARQWLSELNL
PNSCLKSYGSGYVVTVDLTPLQKMVQDIDGLGAPGKDSKLEMDNAKYQAWQSGFKAQEEN
MKTTLQTLTQKYSNANSLYDNLVKVLSSTISSSLETAKSFLQG
>P18014
MFSVNNTHSSVSCSPSINSNSTSNEHYLRILTEWEKNSSPGEERGIAFNRLSQCFQNQEA
VLNLSDLNLTSLPELPKHISALIVENNKLTSLPKLPAFLKELNADNNRLSVIPELPESLT
TLSVRSNQLENLPVLPNHLTSLFVENNRLYNLPALPEKLKFLHVYYNRLTTLPDLPDKLE
ILCAQRNNLVTFPQFSDRNNIRQKEYYFHFNQITTLPESFSQLDSSYRINISGNPLSTRV
LQSLQRLTSSPDYHGPQIYFSMSDGQQNTLHRPLADAVTAWFPENKQSDVSQIWHAFEHE
EHANTFSAFLDRLSDTVSARNTSGFREQVAAWLEKLSASAELRQQSFAVAADATESCEDR
VALTWNNLRKTLLVHQASEGLFDNDTGALLSLGREMFRLEILEDIARDKVRTLHFVDEIE
VYLAFQTMLAEKLQLSTAVKEMRFYGVSGVTANDLRTAEAMVRSREENEFTDWFSLWGPW
HAVLKRTEADRWAQAEEQKYEMLENEYSQRVADRLKASGLSGDADAEREAGAQVMRETEQ
QIYRQLTDEVLALRLSENGSRLHHS
>P55724
MYGRIDSSSDFHYTQSASKQMDAETQEFADTFARMHLDRSNGGSSSAARYTLDHEPPVVP
IDLETFRREIRKFHGKEITDIANNPQEYSDFVSAKARRTADVAQQYGIRRDSENARYFSY
QLGNQCVGLMRTEGGFSMEEEFESKSWRDQFPGHQEITSTVDLQVAHPLVENAGDILLEH
QLRRDGERPLLNWRAENPEAKARAAMMGFVEVDDCDMVLDPKQHPDKWTQTSAAEWRRKD
KPPLYLRKFEDAETAQCSTSCSYETYEDDFM
>Q59639
MKIISCKSIIVSSLLALSATATAGSFNDISWTLENEDNLPETDASGCALKPSTSTSTSKT
FEFGLTDDSNCLDGKQRDEFKYQRRTGYNRLTGYFTIDGNYSDFNKMGVAQTHDHSTSDT
GVFSIYQVRKENGSYIFGVQGDSNYSNNGWSDHPQVKISLDTRYELIIKTNGLPNGNSYE
DANLYLDDVKIWSSSIEVGGEEKQYKKIGAYQLTGGEGEFHVKWDSVKLYTGK
>P09167
MQKIKLTGLSLIISGLLMAQAQAAEPVYPDQLRLFSLGQGVCGDKYRPVNREEAQSVKSN
IVGMMGQWQISGLANGWVIMGPGYNGEIKPGTASNTWCYPTNPVTGEIPTLSALDIPDGD
EVDVQWRLVHDSANFIKPTSYLAHYLGYAWVGGNHSQYVGEDMDVTRDGDGWVIRGNNDG
GCDGYRCGDKTAIKVSNFAYNLDPDSFKHGDVTQSDRQLVKTVVGWAVNDSDTPQSGYDV
TLRYDTATNWSKTNTYGLSEKVTTKNKFKWPLVGETELSIEIAANQSWASQNGGSTTTSL
SQSVRPTVPARSKIPVKIELYKADISYPYEFKADVSYDLTLSGFLRWGGNAWYTHPDNRP
NWNHTFVIGPYKDKASSIRYQWDKRYIPGEVKWWDWNWTIQQNGLSTMQNNLARVLRPVR
AGITGDFSAESQFAGNIEIGAPVPLAADSKVRRARSVDGAGQGLRLEIPLDAQELSGLGF
NNVSLSVTPAANQ
>Q47096
MWMRRNQIVRKLTLGVVTTVLGMSLSFSALSATPVETHGQLSIENGRLVDEQGKRVQLRG
ISSHGLQWFGDYVNKDSMKWLRDDWGINVFRVAMYTAADGYISNPSLANKVKEAVAAAQS
LGVYIIIDWHILSDNDPNIYKAQAKTFFAEMAGLYGSSPNVIYEIANEPNGGVTWNGQIR
PYALEVTDTIRSKDPDNLIIVGTGTWSQDIHDAADNQLPDPNTMYALHFYAGTHGQFLRD
RIDYAQSRGAAIFVSEWGTSDASGNGGPFLPESQTWIDFLNNRGVSWVNWSLTDKSEASA
ALAPGASKSGGWTEQNLSTSGKFVREQIRAGANLGGGDTPTTPTEPTNPGNGTTGDVVLQ

YRNVDNNPSDDAIRMAVNIKNTGSTPIKLSDLQVRYYFHDDGKPGANLFVDWANVGPNNI
VTSTGTPAASTDKANRYVLVTFSSGAGSLQPGAETGEVQVRIHAGDWSNVNETNDYSYGA
NVTSYANWDKITVHDKGTLVWGVEP
>P22542
MKKNIAFLLASMFVFSIATNAYASTQSNKKDLCEHYRQIAKESCKKGFLGVRDGTAGACF
GAQIMVAAKGC
>P0C1A9
MLKTISGTLALSLIIAASVHQAQAATTYNAVVSKSSSDGKTFKTIADAIASAPAGSTPFV
ILIKNGVYNERLTITRNNLHLKGESRNGAVIAAATAAGTLKSDGSKWGTAGSSTITISAK
DFSAQSLTIRNDFDFPANQAKSDSDSSKIKDTQAVALYVTKSGDRAYFKDVSLVGYQDTL
YVSGGRSFFSDCRISGTVDFIFGDGTALFNNCDLVSRYRADVKSGNVSGYLTAPSTNINQ
KYGLVITNSRVIRESDSVPAKSYGLGRPWHPTTTFSDGRYADPNAIGQTVFLNTSMDNHI
YGWDKMSGKDKNGNTIWFNPEDSRFFEYKSYGAGATVSKDRRQLTDAQAAEYTQSKVLGD
WTPTLP
>O68703
MNSIHGHYHIQLSNYSAGENLQSATLTEGVIGAHRVKVETALSHSNLQKKLSATIKHNQS
GRSMLDRKLTSDGKANQRSSFTFSMIMYRMIHFVLSTRVPAVRESVANYGGNINFKFAQT
KGAFLHKIIKHSDTASGVCEALCAHWIRSHAQGQSLFDQLYVGGRKGKFQIDTLYSIKQL
QIDGCKADVDQDEVTLDWFKKNGISERMIERHCLLRPVDVTGTTESEGLDQLLNAILDTH
GIGYGYKKIHLSGQMSAHAIAAYVNEKSGVTFFDPNFGEFHFSDKEKFRKWFTNSFWGNS
MYHYPLGVGQRFRVLTFDSKEV
>P04959
MKSLITPIAAGLLLAFSQYSLAADTGGYTKTDGGDVSGAVKKTASSMQDIVNIIEAAKVD
ANGKKVKGGAYPLVITYTGNEDSLINAAAANICGQWSKDARGVEIKDFTKGLTIIGANGS
SANFGIWIVNSSDIVVRNMRIGYLPGGAQDGDMFRIDNSPNVWLDHNELFAANHECDGTK
DGDTTFESAIDIKKGATYVTISYNYIHGVKKVGLSGFSSSDTAERNITYHHNIYSDVNAR
LPLQRGGNVHAYNNLYTGITSSGLNVRQNGKALIENNWFENAVSPVTSRYDGSNFGTWVL
KGNNITKPADFATYNITWTPDTKEYRNADTWTSTGTYPTVPYSYSPVSAQCVKDKLANYA
GVGKNLATLASSACK
>P74849
MVTSVRTQPPVIMPGMQTEIKTQATNLAANLSAVRESATATLSGEIKGQQLEDFPALIKQ
ASLDALFKCGKDAEALKEVFTNSNNVAGKKAIMEFAGLFRSALNATSDSPEAKTLLMKVG
AEYTAQIIKDGLKEKSAFGPWLPETKKAEAKLENLEKQLLDIIKNNTGGELSKLSTNLVM
QEVMPYIASCIEHNFGCTLDPLTRSSLTQLVDKAAAKAVEALDMCHQKLTQEQGTSVGRE
ARHLEMQTLIPLLLRNVFAQIPADKLPDPKIPEPAAGPVPDGGKKAEPTGINININIDSS
NHSVDNSKHINNSRSHVDNSQRHIDNSNHDNSRKTIDNSRTFIDNSQRHGESHHSTNSSN
VSHSHSRVDSTTHQTETAHSASTGTIDHGIAGKIDVTAHATAEAVTNSSSESKDGKVVTS
EKGTTGETTSFDEVDGVTSKSIIGKPLQATVHGVDDNKQQSQTAEIVNVKPLASQLAGVE
NVKIDTLQSDSTVITGNKAGTTDNDNSQTDKTGPFSGLKFKQNSFLSTVPSVTNMHSIHF
NAREAFLGVIRKALEPDASTPFPVRRAFDGLRGEILPNDTIKSAALKAQCSDIDKHPELK
AKMETLKEVITHHPQKEKLAEIALQFAREAGLTRQKGETDYVLSNVLDGLIGDGSWRAGP
AYESYLNKPGVDRVITTVDGLHMQR
>Q56827
MASISSLGAGSGMDLGSLLDKLQAAEKKRLEPLAQQQTSYKAKLTGFGTLKGSLEKLKSA

SEELKKFDKLNTTKTNGDHKTFTPSTDSKASPGNYEIEVQQLAKAQSLQSTEVSGVKKLL
GEQGKGTRTIIITQPGEKEPMKISLKDDETSLVEIRDAINKKEGNVNASIIKADENGTEE
EGKSYLILTSKKAGTRSIMTIKVEGDDELGKLLNYTSDGKGGGSGAMTQKVGAANAKLTV
NGIPIERQTNEIKDAPEGIILNLKKVSETEEVIVKVNGEDKKIPRPKTEILVVSRDIEPM
KEAIKKWVDSYNELQTTFDSLAKFKPVGKGEAASKDNGALLGDGTLKGIQSQLRHQLFAA
QDVADIATLNKLGIKQKLDGTLEISDEKLEKNLKEKSADVKAFFMGDGAKPGSTQTYNLL
KETLDGHEGTIATATEGINKRLKTLERQVEQTNRNIDATMERYKRQFTELDKLVNSLNNT
SSSLFQLLR
>Q887D0
MISSRIGGAGGVELSRVNQQHDTVPAQTAHPNAVTAGMNPPLTPDQSGSHATESSSAGAA
RLNVAARHTQLLQAFKAEHGTAPVSGAPMISSRAALLIGSLLQAEPLPFEVMAEKLSPER
YQLKQFQGSDLQQRLEKFAQPGQIPDKAEVGQLIKGFAQSVADQLEHFQLMHDASPATVG
QHAKADKATLAVSQTALGEYAGRASKAIGEGLSNSIASLDEHISALDLTLQDAEQGNKES
LHADRQALVDAKTTLVGLHADFVKSPEAKRLASVAAHTQLDNVVSDLVTARNTVGGWKGA
GPIVAAAVPQFLSSMTHLGYVRLSTSDKLRDTIPETSSDANMLKASIIGMVAGIAHETVN
SVVKPMFQAALQKTGLNERLNMVPMKAVDTNTVIPDPFELKSEHGELVKKTPEEVAQDKA
FVKSERALLNQKKVQGSSTHPVGELMAYSAFGGSQAVRQMLNDVHQINGQTLSARALASG
FGGAVSASSQTLLQLKSNYVDPQGRKIPVFTPDRAESDLKKDLLKGMDLREPSVRTTFYS
KALSGIQSSALTSALPPVTAQAEGASGTLSAGAILRNMALAATGSVSYLSTLYTNQSVTA
EAKALKAAGMGGATPMLDRTETALNNIRHPNRESLPHTFQKSTLSGIPRVAENAYHMGRG
ALQLPTQMAVDTVRVVDEGVLNAVASAREALKQPTKDDDALRALEEGLLDPR
>O82882
MNTKMNERWRTPMKLKYLSCTILAPLAIGVFSATAADNNSAIYFNTSQPINDLQGSLAAE
VKFAQSQILPAHPKEGDSQPHLTSLRKSLLLVRPVKADDKTPVQVEARDDNNKILGTLTL
YPPSSLPDTIYHLDGVPEGGIDFTPHNGTKKIINTVAEVNKLSDASGSSIHSHLTNNALV
EIHTANGRWVRDIYLPQGPDLEGKMVRFVSSAGYSSTVFYGDRKVTLSVGNTLLFKYVNG
QWFRSGELENNRITYAQHIWSAELPAHWIVPGLNLVIKQGNLSGRLNDIKIGAPGELLLH
TIDIGMLTTPRDRFDFAKDKEAHREYFQTIPVSRMIVNNYAPLHLKEVMLPTGELLTDMD
PGNGGWHSGTMRQRIGKELVSHGIDNANYGLNSTAGLGENSHPYVVAQLAAHNSRGNYAN
GIQVHGGSGGGGIVTLDSTLGNEFSHEVGHNYGLGHYVDGFKGSVHRSAENNNSTWGWDG
DKKRFIPNFYPSQTNEKSCLNNQCQEPFDGHKFGFDAMAGGSPFSAANRFTMYTPNSSAI
IQRFFENKAVFDSRSSTGFSKWNADTQEMEPYEHTIDRAEQITASVNELSESKMAELMAE
YAVVKVHMWNGNWTRNIYIPTASADNRGSILTINHEAGYNSYLFINGDEKVVSQGYKKSF
VSDGQFWKERDVVDTREARKPEQFGVPVTTLVGYYDPEGTLSSYIYPAMYGAYGFTYSDD
SQNLSDNDCQLQVDTKEGQLRFRLANHRANNTVMNKFHINVPTESQPTQATLVCNNKILD
TKSLTPAPEGLTYTVNGQALPAKENEGCIVSVNSGKRYCLPVGQRSGYSLPDWIVGQEVY
VDSGAKAKVLLSDWDNLSYNRIGEFVGNVNPADMKKVKAWNGQYLDFSKPRSMRVVYK
>P45386
MLNKKFKLNFIALTVAYALTPYTEAALVRDDVDYQIFRDFAENKGKFSVGATNVEVRDKK
NQSLGSALPNGIPMIDFSVVDVDKRIATLVNPQYVVGVKHVSNGVSELHFGNLNGNMNNG
NAKSHRDVSSEENRYYTVEKNNFPTENVTSFTTKEEQDAQKRREDYYMPRLDKFVTEVAP
IEASTANNNKGEYNNSDKYPAFVRLGSGSQFIYKKGSRYQLILTEKDKQGNLLRNWDVGG
DNLELVGNAYTYGIAGTPYKVNHENNGLIGFGNSKEEHSDPKGILSQDPLTNYAVLGDSG
SPLFVYDREKGKWLFLGSYDFWAGYNKKSWQEWNIYKHEFAEKIYQQYSAGSLTGSNTQY

TWQATGSTSTITGGGEPLSVDLTDGKDKPNHGKSITLKGSGTLTLNNHIDQGAGGLFFEG
DYEVKGTSDSTTWKGAGVSVADGKTVTWKVHNPKYDRLAKIGKGTLVVEGKGKNEGLLKV
GDGTVILKQKADANNKVQAFSQVGIVSGRSTLVLNDDKQVDPNSIYFGFRGGRLDLNGNS
LTFDHIRNIDDGARVVNHNMTNTSNITITGESLITNPNTITSYNIEAQDDDHPLRIRSIP
YRQLYFNQDNRSYYTLKKGASTRSELPQNSGESNENWLYMGRTSDEAKRNVMNHINNERM
NGFNGYFGEEETKATQNGKLNVTFNGKSDQNRFLLTGGTNLNGDLNVEKGTLFLSGRPTP
HARDIAGISSTKKDPHFTENNEVVVEDDWINRNFKATTMNVTGNASLYSGRNVANITSNI
TASNNAQVHIGYKTGDTVCVRSDYTGYVTCHNSNLSEKALNSFNPTNLRGNVNLTENASF
TLGKANLFGTIQSIGTSQVNLKENSHWHLTGNSNVNQLNLTNGHIHLNAQNDANKVTTYN
TLTVNSLSGNGSFYYWVDFTNNKSNKVVVNKSATGNFTLQVADKTGEPNHNELTLFDASN
ATRNNLEVTLANGSVDRGAWKYKLRNVNGRYDLYNPEVEKRNQTVDTTNITTPNDIQADA
PSAQSNNEEIARVETPVPPPAPATESAIASEQPETRPAETAQPAMEETNTANSTETAPKS
DTATQTENPNSESVPSETTEKVAENPPQENETVAKNEQEATEPTPQNGEVAKEDQPTVEA
NTQTNEATQSEGKTEETQTAETKSEPTESVTVSENQPEKTVSQSTEDKVVVEKEEKAKVE
TEETQKAPQVTSKEPPKQAEPAPEEVPTDTNAEEAQALQQTQPTTVAAAETTSPNSKPAE
ETQQPSEKTNAEPVTPVVSENTATQPTETEETAKVEKEKTQEVPQVASQESPKQEQPAAK
PQAQTKPQAEPARENVLTTKNVGEPQPQAQPQTQSTAVPTTGETAANSKPAAKPQAQAKP
QTEPARENVSTVNTKEPQSQTSATVSTEQPAKETSSNVEQPAPENSINTGSATTMTETAE
KSDKPQMETVTENDRQPEANTVADNSVANNSESSESKSRRRRSVSQPKETSAEETTVAST
QETTVDNSVSTPKPRSRRTRRSVQTNSYEPVELPTENAENAENVQSGNNVANSQPALRNL
TSKNTNAVLSNAMAKAQFVALNVGKAVSQHISQLEMNNEGQYNVWISNTSMNKNYSSEQY
RRFSSKSTQTQLGWDQTISNNVQLGGVFTYVRNSNNFDKASSKNTLAQVNFYSKYYADNH
WYLGIDLGYGKFQSNLQTNNNAKFARHTAQIGLTAGKAFNLGNFAVKPTVGVRYSYLSNA
DFALAQDRIKVNPISVKTAFAQVDLSYTYHLGEFSITPILSARYDANQGNGKINVSVYDF
AYNVENQQQYNAGLKLKYHNVKLSLIGGLTKAKQAEKQKTAEVKLSFSF
>P27458
MKKISKAGLGLALVCALATIGGNAARRATAQRRGSGVFYDEMFDFDIDAHLAKHAPHLHK
HSEEISHWAGYSGISRSVDRADGAAERAVTPSARRIVRSASWRAPTASARRPARSRWRCA
SRCTSAIPTRQGAGDAGPRQSAAGAVRAFRRQRAGGRAARRRRVPAGLRPPVQRTAPGQG
GFGPLRQGRPGRAAVSPNGLLQFPFPRGASWHVGGAHTNTGSGNYPMSSLDMSRGGGWGS
NQNGNWVSASAAGSFKRHSSCFAEIVHTGGWSTTYYHLMNIQYNTGANVSMNTAIANPAN
TQAQALCNGGQSTGPHEHWSLKQNGSFYHLNGTYLSGYRITATGSSYDTNCSRFYLTKNG
QNYCYGYYVNPGPN
>P95434
MAQIFNPNPGNTLDTVANALKEQANAANKDVNDAIKALQGTDNADNPALLAELQHKINKW
SVIYNINSTVTRALRDLMQGILQKI
>P13720
MKKWFPAFLFLSLSGGNDALAGWHNVMFYAFNDYLTTNAGNVKVIDQPQLYIPWNTGSAT
ATYYSCSGPEFASGVYFQEYLAWMVVPKHVYTNEGFNIFLDVQSKYGWSMENENDKDFYF
FVNGYEWDTWTNNGARICFYPGNMKQLNNKFNDLVFRVLLPVDLPKGHYNFPVRYIRGIQ
HHYYDLWQDHYKMPYDQIKQLPATNTLMLSFDNVGGCQPSTQVLNIDHGSIVIDRANGNI
ASQTLSIYCDVPVSVKISLLRNTPPIYNNNKFSVGLGNGWDSIISLDGVEQSEEILRWYT
AGSKTVKIESRLYGEEGKRKPGELSGSMTMVLSFP
>P45354

MYKLNVISLIILTTCSGAAYASTPDFPQHHKTVFGTVTIEKTTADKMTIKQGSDKAQIDW
KSFDIGQKKEVKFEQPNEHAVAYNRVIGGNASQIQGKLTANGKVYLANPNGVIITQGAEI
NVAGLLATTKDLERISENSNSYQFTRRTKDRQVLKEGLVLKDGQVVKEGQVINEGNITAQ
DFVVLNGDEVINKGNINVEKNSTINGKVYLSSGYNFTFTLPDSGISVALEDNTVQGIVKN
EGSIKAGEITLSAKGRKQALDSLVMNNGVLEATKVSNKNGKVVLSADNVELNNESNIKGE
IVTFGADVTSNKELKDNIKITSKTGSKVTSPKINFTGKSVNINGNFGREDSTTHYKDEFK
KLNTEVNIDVPDNENIRIADIEDNTGTGTTGTGTSSFIQTGALSSLLANNGKVNLKGNNV
NISGRIHIDSFRGSDSLLKLTNKGHIDINNADIHSKGRLFFITSLQNEEDFKSNITITDS
KINLGNGAMGLGRSVDEKDYDNRWQKTEGSQRKKFDVKMSNVEFNQVDDVILAGGFEKVN
LDKIVATGQTNFYIDGGVSRNGRKYEYGVLDLDKRTQLSELNQGRRRWGYYYDLELDMNR
AYLYRFDLFATKNTGRSTIKDTEINISNSNINLKNGFVHLLAEKIKLDNSKIDITFDKDN
SQDTLAQTNRLGMNGKVSMINSHIKIVGDEKEGISPTGTYATMFLIGELIGEKSSIFVKS
HQGYTFKTDGNTKIAGKYSKEDLKITAINTGGRAAEEVLINGALGSADNDANIANMAFTI
GDSANTKTTIENADITALAPNGGTAYLSSKDVEIEVKPNSNFTFFELPREKNLNQTKING
ASTKLSERGFARLYDKINGVRASNLSAEQLNVTDASEKIINTKLVSSLDVEKLVSVAVCD
AGNGCEEQQFGDKGNNTKVSVGELEAEQ
>P22251
MGFRINTNVAALNAKANSDLNAKSLDASLSRLSSGLRINSAADDASGMAIADSLRSQANT
LGQAISNGNDALGILQTADKAMDEQLKILDTIKTKATQAAQDGQSLKTRTMLQADINKLM
EELDNIANTTSFNGKQLLSGNFTNQEFQIGASSNQTVKATIGATQSSKIGVTRFETGAQS
FTSGVVGLTIKNYNGIEDFKFDNVVISTSVGTGLGALAEEINKSADKTGVRATYDVKTTG
VYAIKEGTTSQDFAINGVTIGKIEYKDGDGNGSLISAINAVKDTTGVQASKDENGKLVLT
SADGRGIKITGDIGVGSGILANQKENYGRLSLVKNDGRDINISGTNLSAIGMGTTDMISQ
SSVSLRESKGQISATNADAMGFNSYKGGGKFVFTQNVSSISAFMSAQGSGFSRGSGFSVG
SGKNLSVGLSQGIQIISSAASMSNTYVVSAGSGFSSGSGNSQFAALKTTAANTTDETAGV
TTLKGAMAVMDIAETAITNLDQIRADIGSIQNQVTSTINNITVTQVNVKAAESQIRDVDF
ASESANYSKANILAQSGSYAMAQANSSQQNVLRLLQ
>P81717
AVDFGEAIWNPASSSNYSTASNQTSAVIMHTMEGSYAGSISWFQNPSAQVSAHYLIRKSD
GQITQMVREYHQAWHAKNHNYYTIGIEHDGRAADAGNWSAAMVNASARLTKSICARRGVN
CASAWKGPGYDTFHLVPDSVRVKGHGMLSGNENRYDPGKYFPWSNYYNLINGGGGNP
>P0A6P9
MSKIVKIIGREIIDSRGNPTVEAEVHLEGGFVGMAAAPSGASTGSREALELRDGDKSRFL
GKGVTKAVAAVNGPIAQALIGKDAKDQAGIDKIMIDLDGTENKSKFGANAILAVSLANAK
AAAAAKGMPLYEHIAELNGTPGKYSMPVPMMNIINGGEHADNNVDIQEFMIQPVGAKTVK
EAIRMGSEVFHHLAKVLKAKGMNTAVGDEGGYAPNLGSNAEALAVIAEAVKAAGYELGKD
ITLAMDCAASEFYKDGKYVLAGEGNKAFTSEEFTHFLEELTKQYPIVSIEDGLDESDWDG
FAYQTKVLGDKIQLVGDDLFVTNTKILKEGIEKGIANSILIKFNQIGSLTETLAAIKMAK
DAGYTAVISHRSGETEDATIADLAVGTAAGQIKTGSMSRSDRVAKYNQLIRIEEALGEKA
PYNGRKEIKGQA
>P12625
MVRRLWRRIAGWLAACVAILCAFPLHAATAGPGAWSSQQTWAADSVNGGNLTGYFYWPAS
QPTTPNGKRALVLVLHGCVQTASGDVIDNANGAGFNWKSVADQYGAVILAPNATGNVYSN
HCWDYANASPSRTAGHVGVLLDLVNRFVTNSQYAIDPNQVYVAGLSSGGGMTMVLGCIAP

DIFAGIGINAGPPPGTTTAQIGYVPSGFTATTAANKCNAWAGSNAGKFSTQIAGAVWGTS
DYTVAQAYGPMDAAAMRLVYGGNFTQGSQVSISGGGTNTPYTDSNGKVRTHEISVSGMAH
AWPAGTGGDNTNYVDATHINYPVFVMDYWVKNNLRAGSGTGQAGSAPTGLAVTATTSTSV
SLSWNAVANASSYGVYRNGSKVGSATATAYTDSGLIAGTTYSYTVTAVDPTAGESQPSAA
VSATTKSAFTCTATTASNYAHVQAGRAHDSGGIAYANGSNQSMGLDNLFYTSTLAQTAAG
YYIVGNCP
>Q93Q17
MQIQANTVGTQAVAHHSDATTGVGRMGQMEARQVATGQDAILLGSRSEPQKGQGLLSRLG
AQLARPFVAIKEWISNLLGTDKRAAAPKAQTAVSPEDLQRLMKQAAFGSSLGGFAKADVL
NNITGEQLGKDHASLATGNGPLRSLCTALQAVVIGSQQPQLRELATGLLARPIAGIPLQQ
WGSVGGKVTELLTSAPPELLKEAMSQLHTAMGEVADLQRAVKAEVAGEPARSATTAAAVA
PLQSGESEVNVEPADKALAEGLQEQFGLEAEQYLGEQPHGTYSDAEVMALGLYTNGEYQH
LNRSLRQEKQLDAGQALIDQGMSTAFEKSTPTEQLIKTFRGTHGGDAFNEVAEGQVGHDV
AYLSTSRDPKVATNFGGSGSISTIFGRSGIDVSDISVEGDEQEILYNKETDMRVLLSAKD
ERGVTRRVLEEASLGEQSGHSKGLLDGLDLARGAGGADKPQEQDIRLKMRGLDLA
>Q11137
MGSFLLKKAVGLSNISDLLDKSGIFYNYSTKVLPSFDYDTAGKHIAREDSTWNGKYVIGQ
PAEVTYSFPKWEGKFNQFGNKNPYEFNELQKEHARKSLDAWSDIANIKFTEVAVGNVDGM
KASDVKTDITFGNIYDPNGTFQAYATLPNTYAYGKDLSGQAWFSDYHYAGNTTPELGNYG
RLTIIHEIGHTLGLMHPGDYNAGQNVPGYLKSDYAEDSRQYTVMSYWDEYETGAHFQGAY
AGAPLLHDISAMQYLYGANTTTRTGDDVYGFNSNTGIDYYTATDSNDKLIFSVWDSGGND
TFDFSGFYQDQLIDLRAGNFSDVGGLQKNVSIAQNVTIENAIGGFGNDIIHGNDADNTLI
GGEGDDIIYGHSGNNTIYGGRGQDTLHGGTGSNTFIYKEIADSLVTAADKIMDFKTGIDK
IDLSTLIQDTFSSKILNFVDNFTGNAGEATLSYNEVTNASELAINAYGYNYNPDFKIDIV
GFVNYETDFIV
>P15318
MQQSHQAGYANAADRESGIPAAVLDGIKAVAKEKNATLMFRLVNPHSTSLIAEGVATKGL
GVHAKSSDWGLQAGYIPVNPNLSKLFGRAPEVIARADNDVNSSLAHGHTAVDLTLSKERL
DYLRQAGLVTGMADGVVASNHAGYEQFEFRVKETSDGRYAVQYRRKGGDDFEAVKVIGNA
AGIPLTADIDMFAIMPHLSNFRDSARSSVTSGDSVTDYLARTRRAASEATGGLDRERIDL
LWKIARAGARSAVGTEARRQFRYDGDMNIGVITDFELEVRNALNRRAHAVGAQDVVQHGT
EQNNPFPEADEKIFVVSATGESQMLTRGQLKEYIGQQRGEGYVFYENRAYGVAGKSLFDD
GLGAAPGVPSGRSKFSPDVLETVPASPGLRRPSLGAVERQDSGYDSLDGVGSRSFSLGEV
SDMAAVEAAELEMTRQVLHAGARQDDAEPGVSGASAHWGQRALQGAQAVAAAQRLVHAIA
LMTQFGRAGSTNTPQEAASLSAAVFGLGEASSAVAETVSGFFRGSSRWAGGFGVAGGAMA
LGGGIAAAVGAGMSLTDDAPAGQKAAAGAEIALQLTGGTVELASSIALALAAARGVTSGL
QVAGASAGAAAGALAAALSPMEIYGLVQQSHYADQLDKLAQESSAYGYEGDALLAQLYRD
KTAAEGAVAGVSAVLSTVGAAVSIAAAASVVGAPVAVVTSLLTGALNGILRGVQQPIIEK
LANDYARKIDELGGPQAYFEKNLQARHEQLANSDGLRKMLADLQAGWNASSVIGVQTTEI
SKSALELAAITGNADNLKSVDVFVDRFVQGERVAGQPVVLDVAAGGIDIASRKGERPALT
FITPLAAPGEEQRRRTKTGKSEFTTFVEIVGKQDRWRIRDGAADTTIDLAKVVSQLVDAN
GVLKHSIKLDVIGGDGDDVVLANASRIHYDGGAGTNTVSYAALGRQDSITVSADGERFNV
RKQLNNANVYREGVATQTTAYGKRTENVQYRHVELARVGQLVEVDTLEHVQHIIGGAGND
SITGNAHDNFLAGGSGDDRLDGGAGNDTLVGGEGQNTVIGGAGDDVFLQDLGVWSNQLDG

GAGVDTVKYNVHQPSEERLERMGDTGIHADLQKGTVEKWPALNLFSVDHVKNIENLHGSR
LNDRIAGDDQDNELWGHDGNDTIRGRGGDDILRGGLGLDTLYGEDGNDIFLQDDETVSDD
IDGGAGLDTVDYSAMIHPGRIVAPHEYGFGIEADLSREWVRKASALGVDYYDNVRNVENV
IGTSMKDVLIGDAQANTLMGQGGDDTVRGGDGDDLLFGGDGNDMLYGDAGNDTLYGGLGD
DTLEGGAGNDWFGQTQAREHDVLRGGDGVDTVDYSQTGAHAGIAAGRIGLGILADLGAGR
VDKLGEAGSSAYDTVSGIENVVGTELADRITGDAQANVLRGAGGADVLAGGEGDDVLLGG
DGDDQLSGDAGRDRLYGEAGDDWFFQDAANAGNLLDGGDGRDTVDFSGPGRGLDAGAKGV
FLSLGKGFASLMDEPETSNVLRNIENAVGSARDDVLIGDAGANVLNGLAGNDVLSGGAGD
DVLLGDEGSDLLSGDAGNDDLFGGQGDDTYLFGVGYGHDTIYESGGGHDTIRINAGADQL
WFARQGNDLEIRILGTDDALTVHDWYRDADHRVEIIHAANQAVDQAGIEKLVEAMAQYPD
PGAAAAAPPAARVPDTLMQSLAVNWR
>O25001
MLGNVKKTLFGVLCLGTLCLRGLMAEPDAKELVNLGIESAKKQDFAQAKTHFEKACELKN
GFGCVFLGAFYEEGKGVGKDLKKAIQFYTKGCELNDGYGCNLLGNLYYNGQGVSKDAKKA
SQYYSKACDLNHAEGCMVLGSLHHYGVGTPKDLRKALDLYEKACDLKDSPGCINAGYIYS
VTKNFKEAIVRYSKACELKDGRGCYNLGVMQYNAQGTAKDEKQAVENFKKGCKSSVKEAC
DALKELKIEL
>Q05608
MKSVKIMGTMPPSISLAKAHERISQHWQNPVGELNIGGKRYRIIDNQVLRLNPHSGFSLF
REGVGKIFSGKMFNFSIARNLTDTLHAAQKTTSQELRSDIPNALSNLFGAKPQTELPLGW
KGEPLSGAPDLEGMRVAETDKFAEGESHISIIETKDKQRLVAKIERSIAEGHLFAELEAY
KHIYKTAGKHPNLANVHGMAVVPYGNRKEEALLMDEVDGWRCSDTLRTLADSWKQGKINS
EAYWGTIKFIAHRLLDVTNHLAKAGVVHNDIKPGNVVFDRASGEPVVIDLGLHSRSGEQP
KGFTESFKAPELGVGNLGASEKSDVFLVVSTLLHCIEGFEKNPEIKPNQGLRFITSEPAH
VMDENGYPIHRPGIAGVETAYTRFITDILGVSADSRPDSNEARLHEFLSDGTIDEESAKQ
ILKDTLTGEMSPLSTDVRRITPKKLRELSDLLRTHLSSAATKQLDMGGVLSDLDTMLVAL
DKAEREGGVDKDQLKSFNSLILKTYRVIEDYVKGREGDTKNSSTEVSPYHRSNFMLSIVE
PSLQRIQKHLDQTHSFSDIGSLVRAHKHLETLLEVLVTLSQQGQPVSSETYGFLNRLAEA
KITLSQQLNTLQQQQESAKAQLSILINRSGSWADVARQSLQRFDSTRPVVKFGTEQYTAI
HRQMMAAHAAITLQEVSEFTDDMRNFTVDSIPLLIQLGRSSLMDEHLVEQREKLRELTTI
AERLNRLEREWM
>O05465
MKLFTSTLTAKKSSTHKPLISLALSVLISTLLISETAQAADANDRLEQEVDKQAKQLMAQ
YQIPGMAFGIIVDGKSHFYNYGLADKQRNQPVSEDTIFELGSVSKTFAATLASYSELNGT
LSLDDTADKYIPYLKNSAIGNTKLISLVTYSAGGYHYRCLKTLENNKELLQYYKSWHPDF
PVNSKRLYSNASIGLFGYISALSMHSDYTKLIENTVLPSLKMTNTFVDVPANKMEDYAFG
YNAAGEPIRVNPGMLDAEAYGIKSTSADMTRFMAANMGLVTVDSQMQQALDNNRKGYYRT
KSFTQGLAWEMYPLPTTLQQLVEGNSTETILQPQPIQLNEPPTPVLNDVWVNKTGATNGF
GAYIAYMPAKKTGMFILANKNYPNTERVKAAYTILDSVMNN
>P27755
MNAIKTAVAAVTAAASLVAFSPAEAATATANLNVTANVGGACSIGSGAGGGTLNFGTYDP
VVVNSALGVDLFGTGSLSVQCTLLSTAVITLGQGLYPAAGSTAAVPLRRMRNAASTDYLS
YFLYMDVTRLIAWGNTSGTGLPFLGLGLPVPVQVYGTVPRGQNVPSGTYNDTVVATITF
>Q43998

MAHVRRKVATLNMALAGSLLMVLGAQSALAQGNFSRQEAARMAHRPGVMPRGGPLFPGRS
LAGVPGFPLPSIHTQQAYDPQSDFTARWTRADALQIKAHSDATVAAGQNSLPAQLTMPNI
PADFPVINPDVWVWDTWTLIDKHADQFSYNGWEVIFCLTADPNAGYGFDDRHVHARIGFF
YRRAGIPASRRPVNGGWTYGGHLFPDGASAQVYAGQTYTNQAEWSGSSRLMQIHGNTVSV
FYTDVAFNRDANANNITPPQAIITQTLGRIHADFNHVWFTGFTAHTPLLQPDGVLYQNGA
QNEFFNFRDPFTFEDPKHPGVNYMVFEGNTAGQRGVANCTEADLGFRPNDPNAETLQEVL
DSGAYYQKANIGLAIATDSTLSKWKFLSPLISANCVNDQTERPQVYLHNGKYYIFTISHR
TTFAAGVDGPDGVYGFVGDGIRSDFQPMNYGSGLTMGNPTDLNTAAGTDFDPSPDQNPRA
FQSYSHYVMPGGLVESFIDTVENRRGGTLAPTVRVRIAQNASAVDLRYGNGGLGGYGDIP
ANRADVNIAGFIQDLFGQPTSGLAAQASTNNAQVLAQVRQFLNQ
>P77335
MTEIVADKTVEVVKNAIETADGALDLYNKYLDQVIPWQTFDETIKELSRFKQEYSQAASV
LVGDIKTLLMDSQDKYFEATQTVYEWCGVATQLLAAYILLFDEYNEKKASAQKDILIKVL
DDGITKLNEAQKSLLVSSQSFNNASGKLLALDSQLTNDFSEKSSYFQSQVDKIRKEAYAG
AAAGVVAGPFGLIISYSIAAGVVEGKLIPELKNKLKSVQNFFTTLSNTVKQANKDIDAAK
LKLTTEIAAIGEIKTETETTRFYVDYDDLMLSLLKEAAKKMINTCNEYQKRHGKKTLFEV
PEV
>Q7BU69
MQTSNITNHERNDSSWMSTVKSTTEVSWNKLSFCDILLKIITFGIYSPHETLAEKHSEKK
LMDSFSPSLSQDKMDGEFAHANIDGISIRLCLNKGICSVFYLDGDKIQSTQLSSKEYNNL
LSSLPPKQFNLGKVHTITAPVSGNFKTHKPAPEVIETAINCCTSIIPNDDYFHVKDTDFN
SVWHDIYRDIRASDSNSTKIYFNNIEIPLKLIADLINELGINEFIDSKKELQMLSYNQVN
KIINSNFPQQDLCFQTEKLLFTSLFQDPAFISALTSAFWQSLHITSSSVEHIYAQIMSEN
IENRLNFMPEQRVINNCGHIIKINAVVPKNDTAISASGGRAYEVSSSILPSHITCNGVGI
NKIETSYLVHAGTLPSSEGLRNAIPPESRQVSFAIISPDV
>P08407
MKKIRGLCLPVMLGAVLMSQHVHAVDNLTFRGKLIIPACTVSNTTVDWQDVEIQTLSQNG
NHEKEFTVNMRCPYNLGTMKVTITATNTYNNAILVQNTSNTSSDGLLVYLYNSNAGNIGT
AITLGTPFTPGKITGNNADKTISLHAKLGYKGNMQNLIAGPFSATATLVASYS
>P14727
MDPIRSRTPSPARELLPGPQPDGVQPTADRGVSPPAGGPLDGLPARRTMSRTRLPSPPAP
SPAFSAGSFSDLLRQFDPSLFNTSLFDSLPPFGAHHTEAATGEWDEVQSGLRAADAPPPT
MRVAVTAARPPRAKPAPRRRAAQPSDASPAAQVDLRTLGYSQQQQEKIKPKVRSTVAQHH
EALVGHGFTHAHIVALSQHPAALGTVAVKYQDMIAALPEATHEAIVGVGKQWSGARALEA
LLTVAGELRGPPLQLDTGQLLKIAKRGGVTAVEAVHAWRNALTGAPLNLTPEQVVAIASH
DGGKQALETVQRLLPVLCQAHGLTPQQVVAIASNGGGKQALETVQRLLPVLCQAHGLTPQ
QVVAIASNSGGKQALETVQRLLPVLCQAHGLTPEQVVAIASNGGGKQALETVQRLLPVLC
QAHGLTPEQVVAIASNIGGKQALETVQALLPVLCQAHGLTPEQVVAIASNIGGKQALETV
QALLPVLCQAHGLTPEQVVAIASNIGGKQALETVQALLPVLCQAHGLTPEQVVAIASHDG
GKQALETVQRLLPVLCQAHGLTPEQVVAIASHDGGKQALETVQRLLPVLCQAHGLTPQQV
VAIASNGGGKQALETVQRLLPVLCQAHGLTPEQVVAIASNSGGKQALETVQALLPVLCQA
HGLTPEQVVAIASNSGGKQALETVQRLLPVLCQAHGLTPEQVVAIASHDGGKQALETVQR
LLPVLCQAHGLTPEQVVAIASHDGGKQALETVQRLLPVLCQAHGLTPEQVVAIASHDGGK
QALETVQRLLPVLCQAHGLTPQQVVAIASNGGGRPALETVQRLLPVLCQAHGLTPEQVVA

IASHDGGKQALETVQRLLPVLCQAHGLTPQQVVAIASNGGGRPALESIVAQLSRPDPALA
ALTNDHLVALACLGGRPALDAVKKGLPHAPALIKRTNRRIPERTSHRVADHAQVVRVLGF
FQCHSHPAQAFDDAMTQFGMSRHGLLQLFRRVGVTELEARSGTLPPASQRWDRILQASGM
KRAKPSPTSTQTPDQASLHAFADSLERDLDAPSPMHEGDQTRASSRKRSRSDRAVTGPSA
QQSFEVRVPEQRDALHLPLSWRVKRPRTSIGGGLPDPGTPTAADLAASSTVMREQDEDPF
AGAADDFPAFNEEELAWLMELLPQ
>Q9RBS1
MSHSKIKAGGHGSSGIGNDFTPAKTPAPATPAPQSQQVNDLLGRGVGNALNKSNLGSDSQ
TWTPGSTMVSLKSRSSSSHKPDTGGDTKPDSTSGGKRKRDDETDPNAETEGGKKKKKRDD
ENDSSQAGGAGSSAGSSGGSPEDALMNIALQRAIQRQTQTRQKMQEAMKIKDDDD
>Q07566
MHITNLGLHQVSFQSGDSYKGAEETGKHKGVSVISYQRVKNGERNKGIEALNRLYLQNQT
SLTGKSLLFARDKAEVFCEAIKLAGGDTSKIKAMMERLDTYKLGEVNKRHINELNKVISE
EIRAQLGIKNKKELQTKIKQIFTDYLNNKNWGPVNKNISHHGKNYSFQLTPASHMKIGNK
NIFVKEYNGKGICCASTRERDHIANMWLSKVVDDEGKEIFSGIRHGVISAYGLKKNSSER
AVAARNKAEELVSAALYSRPELLSQALSGKTVDLKIVSTSLLTPTSLTGGEESMLKDQVS
ALKGLNSKRGGPTKLLIRNSDGLLKEVSVNLKVVTFNFGVNELALKMGLGWRNVDKLNDE
SICSLLGDNFLKNGVIGGWAAEAIEKNPPCKNDVIYLANQIKEIVNNKLQKNDNGEPYKL
SQRVTLLAYTIGAVPCWNCKSGKDRTGMQDAEIKREIIRKHETGQFSQLNSKLSSEEKRL
FSTILMNSGNMEIQEMNTGVPGNKVMKKLPLSSLELSYSERIGDPKIWNMVKGYSSFV
>P55711
MSASNLLPMISSNPAQFAQASLAKAFAPRVAQGQQSVLSFEAMLSTNMLDRIGPLASRED
LPPPDAESTLEDLQKDPLALLPPHMRAAIESMDQTPQSAVVIDDHYVAPAPIQSSRITWN
GGSLTKPELQIVAVLNRHKDLCPLSWESLEAKANDPSTPPDLKAAIEALLQDPELFYAIG
SQGDGRCGGKISAKDLSEFSKHHPQVAAFQESQAQSYAQNYIPSDSAENAQPSVMTENDA
LRELYRYSEYLPKNLSLADFKQIVDGEAKTGKCPPQVIAAAQYFVSHPEEWKQLYGGNID
KVHKEDFLQVASSSMSLTQAELDTLKTINSHQELFFGSGDLTRDKLASMADDKSLDPKVR
EAASQLLSDPLLFGLLNNAITGYKTHHGFFDFGGGHTVDSGNVSKEDFGRFYTNMTTANR
TVQQPKFHVPETEAAQNAVADMKMGLADQPDIKSPKKNGGALMHVVDSVLRVGSKVLDWA
ATAVGVLSFIPGIGQVADLVSMTLACEAQAANLLRTAITGGNMKQALIEAGIGVAAQAVG
LVSGPGVKLAIRNGLARKAIEEAATAGINLPLSMAQHYAEGYLNDLKARLAADHPA
>P42779
MNLSNISAVKVLTLVVSAAIAGQVCAAESIVNYESANAISKQPEGSVRFIVKYKDGTPSS
QGLKTRSTTKVMASGMQVAGFEAQFVRTTGLGAGIFAVPELKTTKEAHLVMDTIASNPDV
EFVEVDRLAYPKAAPNDPSYRQQWHYFGNYGVKANKVWDRGFTGQGVVVSVVDTGILDHV
DLNGNMLPGYDFISSAPNARDGDQRDNNPADEGDWFDNWDCGGYPDPRREKKFSTWHGSH
VAGTIAAVTNNGVGVAGVAYGAKVIPVRVLGKCGGYDSDITDGMYWSAGGHIDGVPDNQN
PAQVVNMSLGGGGGCSQNSQRMIDKTTNLGALIVIAAGNENQDASRTWPSSCNNVLSVGA
TTPKGKRAPFSNYGARVHLAAPGTNILSTIDVGQAGPVRSSYGMKAGTSMAAPHVSGVAA
LVISAANSIGKTLTPSELSDILVRTTSRFNGRLDRGLGSGIVDANAAVNAVLGDQNRAQP
RPPVNQPINSGNKVYRSDRRVAIRDLRSVTSGIRVNDQARVGSANITLTLDIRYGDRSQL
AVELIAPSGRVYPIYHDGKRQPNIVGPATFSVKNERLQGTWTLKVTDKARGVTGSIDSWS
LTF
>P40601

MKRSFIFAPGMLALSISAISNAHAYNNLYVFGDSLSDGGNNGRYTVDGINGTESKLYNDF
IAQQLGIELVNSKKGGTNYAAGGATAVADLNNKHNTQDQVMGYLASHSNRADHNGMYVHW
IGGNDVDAALRNPADAQKIITESAMAASSQVHALLNAGAGLVIVPTVPDVGMTPKIMEFV
LSKGGATSKDLAKIHAVVNGYPTIDKDTRLQVIHGVFKQIGSDVSGGDAKKAEETTKQLI
DGYNELSSNASKLVDNYNQLEDMALSQENGNIVRVDVNALLHEVIANPLRYGFLNTIGYA
CAQGVNAGSCRSKDTGFDASKPFLFADDFHPTPEAHHIVSQYTVSVLNAPYRVMLLTNAN
NVPVKGALASLDGRLQQLRNVDNEQGKLGVFGGYSGNHSHTLTLGSDYQIMDNILLGGMI
SRYQDNSSPADNFHYDGRGYVFTAYGLWRYYDKGWISGDLHYLDMKYEDITRGIVLNDWL
RKENASTSGHQWGGRITAGWDIPLTSAVTTSPIIQYAWDKSYVKGYRESGNNSTAMHFGE
QRYDSQVGTLGWRLDTNFGYFNPYAEVRFNHQFGDKRYQIRSAINSTQTSFVSESQKQDT
HWREYTIGMNAVITKDWGAFASISRNDGDVQNHTYSFSLGVNASF
>Q9RBS2
MPRDTPQTVPGHSPFWPLFFFTHNNKREARHDLARLSLTLMPILPRLFHRTSRTSSADTQ
RDARTPPNASPLHGEPGRTPRSRGELGRNLRLRSNAQTSGTPGTPARPQIRASASRTAPS
TPQHPQGTEGTRTVPNSPLHNDARVFRERADHTGLSAWRTEMLTRFIEHSRKHGLANDFE
QVRVYDRLSRAVDHLKSVLRMSGDSVQLKSLPVPELPDVTFEIAHLKNLETVDCDLHALP
ATLENLFLLETLSLKGAKNFKALPDAVWRLPALQELKLSETGLKSLPPVGGGSALQRLTI
EDSPLEQLPAGFADLDQLASLSLSNTKLEKLSSGIGQLPALKSLSLQDNPKLERLPKSLG
QVEELTLIGGRIHALPSASGMSSLQKLTVDNSSLAKLPADFGALGNLAHVSLSNTKLRDL
PASIGNLFTLKTLSLQDNPKLGSLPASFGQLSGLQELTLNGNRIHELPSMGGASSLQTLT
VDDTALAGLPADFGALRNLAHLSLSNTQLRELPANTGNLHALKTLSLQGNQQLATLPSSL
GYLSGLEELTLKNSSVSELPPMGPGSALKTLTVENSPLTSIPADIGIQCERLTQLSLSNT
QLRALPSSIGKLSNLKGLTLKNNARLELLSESGVRKLESVRKIDLSGCVRLTGLPSSIGK
LPKLRTLDLSGCTGLSMASLPRSLVLPRDGLNVIFPEHLKTDVGNARIQQNPRARLLEGH
LERQNEAMNHAMFGDDESVGSMTSVPDNEAGVVSMAFHAKHAYKRLERLRQEAGSSMAAP
MRNNDAESMRRALAYAFRSMSDLPTFKKLDNAARSLSYEVQEQLADLVAAPVGQKLVKAI
AEGAYGAGRDARAIEQMLPELATRIANHPEIQQLREQAKRYPSSLPPEKLAAKLTPLIQP
LWDGTRAVAPMPGQLRQALQDLLVTAEGRQLASEIDRAAHSHRAGDEGKALRGLLPVLAA
KIGSDPRVVRVREFVSRHTFGSPQQRAESLAQTLTPVVQELWAQTQAEVAKQGQTPAASP
SRQR
>P04127
MIKSVIAGAVAMAVVSFGVNNAAPTIPQGQGKVTFNGTVVDAPCSISQKSADQSIDFGQL
SKSFLEAGGVSKPMDLDIELVNCDITAFKGGNGAKKGTVKLAFTGPIVNGHSDELDTNGG
TGTAIVVQGAGKNVVFDGSEGDANTLKDGENVLHYTAVVKKSSAVGAAVTEGAFSAVANF
NLTYQ
>P04977
MRCTRAIRQTARTGWLTWLAILAVTAPVTSPAWADDPPATVYRYDSRPPEDVFQNGFTAW
GNNDNVLDHLTGRSCQVGSSNSAFVSTSSSRRYTEVYLEHRMQEAVEAERAGRGTGHFIG
YIYEVRADNNFYGAASSYFEYVDTYGDNAGRILAGALATYQSEYLAHRRIPPENIRRVTR
VYHNGITGETTTTEYSNARYVSQQTRANPNPYTSRRSVASIVGTLVRMAPVIGACMARQA
ESSEAMAAWSERAGEAMVLVYYESIAYSF
>P07111
MRLRFSVPLFFFGCVFVHGVFAGPFPPPGMSLPEYWGEEHVWWDGRAAFHGEVVRPACTL
AMEDAWQIIDMGETPVRDLQNGFSGPERKFSLRLRNCEFNSQGGNLFSDSRIRVTFDGVR

GETPDKFNLSGQAKGINLQIADVRGNIARAGKVMPAIPLTGNEEALDYTLRIVRNGKKLE
AGNYFAVLGFRVDYE
>P23024
MQLLKQLFKKKFVKEEHDKKTGQEGMTLLEVIIVLGIMGVVSAGVVTLAQRAIDSQIMTK
AAQSLNSIQVALTQTYRGLGNYPATADATAASKLTSGLVSLGKISSDEAKNPFNGTNMNI
FSFPRNAAANKAFAISVDGLTQAQCKTLITSVGDMFPYIAIKAGGAVALADLGDFENSAA
AAETGVGVIKSIAPASKNLDLTNITHVEKLCKGTAPFGVAFGNS
>Q8Z289
MMTMLRGWITMIVMLTAINAQAACSWPAWEQFKKDYISQQGRVIDPGDARKITTSEGQSY
AMFFALAANDRPAFAQLFNWTQNNLAQGSLREHLPAWLWGQKDPDTWSVLDSNSASDGDI
WMAWSLLEAGRLWKETRYTEVGTALLKRIAREEVVNVPGLGSMLLPGKIGFAEANSWRFN
PSYLPPQLAQYFSRFGAPWSTLRETNLRLLLETSPKGFSPDWVRYESKQGWQLKAEKTLI
SSYDAIRVYLWTGMMHDGDPQKARLLARFKPMATLTMKNGVPPEKVDVVSGNAQGTGPVG
FSAALLPFLQNRGAQAVQRQRVADHFPGSDAYYNYVLTLFGQGWDQHRFRFTVKGELLPD
WGQECVSSR
>P15922
MKVITFSRRSALASIVATCLMSTPALAATAQAPQKLQIPTLSYDDHSVMLVWDTPEDTSN
ITDYQIYQNGQLIGLASQNNDKNSPAKPYISAFYKSDAANFHHRIVLQNAKVDGLKAGTD
YQFTVRTVYADGTTSNDSNTVTTTTTAVPKVINITQYGAKGDGTTLNTSAIQKAIDACPT
GCRIDVPAGVFKTGALWLKSDMTLNLLQGATLLGSDNAADYPDAYKIYSYVSQVRPASLL
NAIDKNSSAVGTFKNIRIVGKGIIDGNGWKRSADAKDELGNTLPQYVKSDNSKVSKDGIL
AKNQVAAAVATGMDTKTAYSQRRSSLVTLRGVQNAYIADVTIRNPANHGIMFLESENVVE
NSVIHQTFNANNGDGVEFGNSQNIMVFNSVFDTGDDSINFAAGMGQDAQKQEPSQNAWLF
NNFFRHGHGAVVLGSHTGAGIVDVLAENNVITQNDVGLRAKSAPAIGGGAHGIVFRNSAM
KNLAKQAVIVTLSYADNNGTIDYTPAKVPARFYDFTVKNVTVQDSTGSNPAIEITGDSSK
DIWHSQFIFSNMKLSGVSPTSISDLSDSQFNNLTFSNLRSGSSPWKFGTVKNVTVDGKTV
TP
>Q47499
MEKVLIWFCGTGTTKQDFLANVEISGFSAIVAIDGIGTAAMLTKTQALAKRANWGGSFVD
MSETLGVLYDQVNGYDDRAGVVTLDSLFPLVDYLKTLKEYQLVVGGHSRGAAVGLTEFLA
ELYHLAVQNQAPGVWANAKTIRLVVVDPVQGQQDADKDTNAFNAILKDKTLAQILAELET
KWFGGREVFDTLVYSARYDARSSFAFDSRWYRFITEQMGKQAGPAKRAKLVMAGFRHSAP
VSKEDEISALYQGKGVAPIAFLQQLVSFDPNWEQSARLLSQIENGYLDQLAAGAKTDLIS
QLDKQTSLLSTALPALSAANRCKKRCRRITRKNRNTAGSTAISTGRRRFNASYQVTSD
>P16326
MRISTQMMYEQNMSGITNSQAEWMKLGEQMSTGKRVTNPSDDPIAASQAVVLSQAQAQNS
QYALARTFATQKVSLEESVLSQVTTAIQTAQEKIVYAGNGTLSDDDRASLATDLQGIRDQ
LMNLANSTDGNGRYIFAGYKTEAAPFDQATGGYHGGEKSVTQQVDSARTMVIGHTGAQIF
NSITSNAVPEPDGSDSEKNLFVMLDTAIAALKTPVEGNNVEKEKAAAAIDKTNRGLKNSL
NNVLTVRAELGTQLSELSTLDSLGSDRALGQKLQMSNLVDVDWNSVISSYVMQQAALQAS
YKTFTDMQGMSLFQLNR
>Q8ZMM8
MERSLDSLAGMAKSAFGAGTSAAMRQATSPKTILEYIINFFTCGGIRRRNETQYQELIET
MAETLKSTMPDRGAPLPENIILDDMDGCRVEFNLPGENNEAGQVIVRVSKGDHSETREIP

LASFEKICRALLFRCEFSLPQDSVILTAQGGMNLKGAVLTGANLTSENLCDADLSGANLE
GAVLFMADCEGANFKGANLSGTSLGDSNFKNACLEDSIMCGATLDHANLTGANLQHASLL
GCSMIECNCSGANMDHTNLSGATLIRADMSGATLQGATIMAAIMEGAVLTRANLRKASFI
STNLDGADLAEANLNNTCFKDCTLTDLRTEDATMSTSTQTLFNEFYSENI
>Q9I747
MAVDMFIKIGDVKGESKDKTHAEEIDVLAWSWGMSQSGSMHMGGGGGAGKVNVQDLSFTK
YIDKSTPNLMMACSSGKHYPQAKLTIRKAGGENQVEYLIITLKEVLVSSVSTGGSGGEDR
LTENVTLNFAQVQVDYQPQKADGAKDGGPVKYGWNIRQNVQA
>P55704
MDINSTSPLNASPQPDSPPPANASAFAHQLSGFQYSPPHAADSLLPQVEADSPYLDTRHP
YSQYLDSAYPYPSPCEWQHDLYTRTRERSPHPSEQRPHARVLQGAPEHDQDQHLEAAGPR
EGSWQVGPSRSGPSQAGLSPSATPLNPSPPPHATDLETKHPYSQYLDWANPSLLDWQQDL
HTRATASPAPLTAERGRSPQPSEQQPHARALQVPEYDQDLIWQRVDAAGPQAGPWQVGPS
HSGPSQARPSHAWPSSSAGAEPAELSDFVMDSGVRAWDHWFLAPHMASEDQMSMLRATGL
MPTAEVPTTTFLMMGMRHVAEFRGEGVIRIRPSVDFDI
>P0C556
MIRAYEQNPQHFIEDLEKVRVEQLTGHGSSVLEELVQLVKDKNIDISIKYDPRKDSEVFA
NRVITDDIELLKKILAYFLPEDAILKGGHYDNQLQNGIKRVKEFLESSPNTQWELRAFMA
VMHFSLTADRIDDDILKVIVDSMNHHGDARSKLREELAELTAELKIYSVIQAEINKHLSS
SGTINIHDKSINLMDKNLYGYTDEEIFKASAEYKILEKMPQTTIQVDGSEKKIVSIKDFL
GSENKRTGALGNLKNSYSYNKDNNELSHFATTCSDKSRPLNDLVSQKTTQLSDITSRFNS
AIEALNRFIQKYDSVMQRLLDDTSGK
>P08408
MIRLSLFISLLLTSVAVLADVQINIRGNVYIPPCTINNGQNIVVDFGNINPEHVDNSRGE
VTKTISISCPYKSGSLWIKVTGNTMGGGQNNVLATNITHFGIALYQGKGMSTPLILGNGS
GNGYGVTAGLDTARSTFTFTSVPFRNGSGILNGGDFQTTASMSMIYN
>P21158
MRAFATNVCTGPVDVLINNAGVSGLWCALGDVDYADMARTFTINALGPLRVTSAMLPGLR
QGALRRVAHVTSRMGSLAANTDGGAYAYRMSKAALNMAVRSMSTDLRPEGFVTVLLHPGW
VQTDMGGPDATLPAPDSVRGMLRVIDGLNPEHSGRFFDYQGTEVPW
>P22963
MSHILRAAVLAAVLLPFPALADQAGKSPAGVRYHGGDEIILQGFHWNVVREAPNDWYNIL
RQQASTIAADGFSAIWMPVPWRDFSSWTDGGKSGGGEGYFWHDFNKNGRYGSDAQLRQAA
GALGGAGVKVLYDVVPNHMNRGYPDKEINLPAGQGFWRNDCADPGNYPNDCDDGDRFIGG
ESDLNTGHPQIYGMFRDELANLRSGYGAGGFRFDFVRGYAPERVDSWMSDSADSSFCVGE
LWKGPSEYPSWDWRNTASWQQIIKDWSDRAKCPVFDFALKERMQNGSVADWKHGLNGNPD
PRWREVAVTFVDNHDTGYSPGQNGGQHHWALQDGLIRQAYAYILTSPGTPVVYWSHMYDW
GYGDFIRQLIQVRRTAGVRADSAISFHSGYSGLVATVSGSQQTLVVALNSDLANPGQVAS
GSFSEAVNASNGQVRVWRSGSGDGGGNDGGEGGLVNVNFRCDNGVTQMGDSVYAVGNVSQ
LGNWSPASAVRLTDTSSYPTWKGSIALPDGQNVEWKCLIRNEADATLVRQWQSGGNNQVQ
AAAGASTSGSF
>P31498
MIGPISQINISGGLSEKETSSLISNEELKNIITQLETDISDGSWFHKNYSRMDVEVMPAL
VIQANNKYPEMNLNLVTSPLDLSIEIKNVIENGVRSSRFIINMGEGGIHFSVIDYKHING

KTSLILFEPANFNSMGPAMLAIRTKTAIERYQLPDCHFSMVEMDIQRSSSECGIFSFALA
KKLYIERDSLLKIHEDNIKGILSDGENPLPHDKLDPYLPVTFYKHTQGKKRLNEYLNTNP
QGVGTVVNKKNETIVNRFDNNKSIVDGKELSVSVHKKRIAEYKTLLKV
>Q8RJP2
MHMNKPLQAWRTPLLTLIFVLPLTATGAVKLTLDGMNSTLDNGLLKVRFGADGSAKEVWK
GGTNLISRLSGAARDPDKNRSFYLDYYSGGVNEFVPERLEVIKQTPDQVHLAYIDDQNGK
LRLEYHLIMTRDVSGLYSYVVAANTGSAPVTVSELRNVYRFDATRLDTLFNSIRRGTPLL
YDELEQLPKVQDETWRLPDGSVYSKYDFAGYQRESRYWGVMGNGYGAWMVPASGEYYSGD
ALKQELLVHQDAIILNYLTGSHFGTPDMVAQPGFEKLYGPWLLYINQGNDRELVADVSRR
AEHERASWPYRWLDDARYPRQRATVSGRLRTEAPHATVVLNSSAENFDIQTTGYLFSART
NRDGRFSLSNVPPGEYRLSAYADGGTQIGLLAQQTVRVEGKKTRLGQIDARQPAPLAWAI
GQADRRADEFRFGDKPRQYRWQTEVPADLTFEIGKSRERKDWYYAQTQPGSWHILFNTRT
PEQPYTLNIAIAAASNNGMTTPASSPQLAVKLNGQLLTTLKYDNDKSIYRGAMQSGRYHE
AHIPLPAGALQQGGNRITLELLGGMVMYDAITLTETPQ
>P07965
MKKSILFIFLSVLSFSPFAQDAKPVESSKEKITLESKKCNIAKKSNKSGPESMNSSNYCC
ELCCNPACTGCY
>P02969
MALSVNTNQPALIALQNLNRTNDDMQAVQTRINTGEAISTAKDTAAVWSHRPGAGDMSGL
AREDEPGSGDIDRGRGPRAGESVSDLLKLMREKVVAAKDTSLTTTSRQALNADFQGLIKN
LNQVLRSATFDGANLLDGSQAADMSFLADADAGQAITLTLQNLSLGGTINTLTATDDILD
PVNAAGVLTRLDATLSAVNQAVGNIGTQAKQIDAHNTFVAKLNDVLETGVGNLVDADLAK
ESARLQALQVKQPLGAQALSIANGAPQIILSLFKGG
>Q9HWK6
MHKRTYLNACLVLALAAGASQALAAPGASEMAGDVAVLQASPASTGHARFANPNAAISAA
GIHFAAPPARRVARAAPLAPKPGTPLQVGVGLKTATPEIDLTTLEWIDTPDGRHTARFPI
SAAGAASLRAAIRLETHSGSLPDDVLLHFAGAGKEIFEASGKDLSVNRPYWSPVIEGDTL
TVELVLPANLQPGDLRLSVPQVSYFADSLYKAGYRDGFGASGSCEVDAVCATQSGTRAYD
NATAAVAKMVFTSSADGGSYICTGTLLNNGNSPKRQLFWSAAHCIEDQATAATLQTIWFY
NTTQCYGDASTINQSVTVLTGGANILHRDAKRDTLLLELKRTPPAGVFYQGWSATPIANG
SLGHDIHHPRGDAKKYSQGNVSAVGVTYDGHTALTRVDWPSAVVEGGSSGSGLLTVAGDG
SYQLRGGLYGGPSYCGAPTSQRNDYFSDFSGVYSQISRYFAP
>P35482
MFKRSLIAASLSVAALVSAQAMAVTGGGASLPAELYKGSADSILPANFSYAVTGSGTGKN
AFLTNNSSLFGTTGTVHYAGSDSVLSGSELTTYNSNYNGTYGPLIQIPSVATSVTVPYRK
DGNTTLNLTSAQLCDAFSGAKTTWGQLLGTTDSTPIRIVYRTGSSGTTELFTRHLNSICP
TRFATNSTFTNARLPAGGTLPSNWVGVAATSTVVSTVKATNGSLGYVSPDAVNINSNAEV
SRVNGNLPTQANVSTALGSVAPPANAADRADPSKWVPVFTNPSAGYSIVGYTNFVFGQCY
KDASVSTDVRAFINKHYGGTTTNAAVAAHGFIPLTPAWKSAIVSAFYTGTSENLAIGNTN
VCNTKGRP
>P58330
MTSILTNVAAMAALQTLRGIDSNMEETQARVSSGLRVGTASDNAAYWSIATTMRSDNMAL
SAVQDALGLGAAKVDTAYAGMENAVEVVKEIRAKLVAATEDGVDKAKIQEEIEQLKQQLT
SIATAASFSGENWLQADITTPVTKSVVGSFVRDSSGVVSVKTIDYVLDGNSVLFDTVGDA

GILDKIYNVSQASVTLPVNVNGTTTEYTVAAYAVDELIAAGATFDGDSANVTGYTVPAGG
IDYNGNFVKVEGTWVRAIDVAATGQEVVYDDGTTKWGVDTTVAGAPAINVVAPASIENID
ITNAAQAANLDALIRGVDEALEDLISATSALGSISMRIGMQEEFVSKLTDSIDSGIGRLV
DADMNEESTRLKALQTQQQLAIQSLSIANTNSENILQLFRQ
>P22630
MKNTAGILAIAGMLIAPLAHADVILHAFNWKYSEVTAKADLIKGAGYKQVLISPPLKSSG
NEWWARYQPQDLRLVDSPLGNKQDLEQLIAAMQARGIAVYADVVLNHMANESWKRNDLNY
PGTELLGQYAANPDYYSRQRLFGDLGQNLLSASDFHPEGCITDWSDPGHVQYWRLCGGAG
DKGLPDLDPNNWVVSQQQAYLKALKGMGIKGFRVDAVKHMSDYQINAVFTPEIKQGMHVF
GEVITTGGAGSTDYERFLKPYLDNSGQGAYDFPLFASLRGALGYGGSMNQLADPGAYGQA
LPGNRAVTFAITHDIPTNDGFRYQILNQTDEKLAYAYLLGRDGGSPLVYSDHGETQDKDG
LRWQDYYLRSDLKGMIRFHNAVQGQPMQLIGSGDCFVLFKRGKQGLVGVNKCDYEQEYWL
DTAKFELNWYRNYKDVLDQSAVINVQSQWVRVAMPARRPPLAAE
>O33680
MSKTVLNAVGTPLYYSGSSTAWFSATGSGPTLHGTAGNDSMWGDSSVNVTMIGGRGDDIY
YLYSSINRAYEAAGEGVDTISTWMSYTLPANFENLTVTGSGRFAFGNEADNIIKGGSGTQ
TIDGRGGNDVLIGAGGADTFVFARGNGSDLITDFNYDDIVRLDGYGFTSFEQILSNVAQE
GADLRLHLADGESLVFANTTADELQAHQFRLSLDRSVLSQTFSDEFNTLQLRNGTSGVWD
AKFWWAPEKGATLSSNGEQQWYINPSYEPTASVNPFSVNNGVLTITAAPASEAIQAEING
YDYTSGMLTTYSSFAQTYGYFEMRADMPDDQGVWPAFWLLPADGSWPPELDVVEMRGQDS
NTVIATVHSNETGSRTSIENSVKVADASGFHTYGVLWTEEEIVWYFDDAAIARADTPSDM
HDPMYMLVNLAVGGIAGTPRDGLADGSEMKIDYIKAYSLDADWQI
>Q6LAD6
MKIAPVAINHSPLSREVPSHAAPTQAKQTNLQSEAGDLDARKSSASSPETRALLATKTVL
GRHKIEVPAFGGWFKKKSSKHETGGSSANADSSSVASDSTEKPLFRLTHVPYVSQGNERM
GCWYACARMVGHSVEAGPRLGLPELYEGREGPAGLQDFSDVERFIHNEGLTRVDLPDNER
FTHEELGALLYKHGPIIFGWKTPNDSWHMSVLTGVDKETSSITFHDPRQGPDLAMPLDYF
NQRLAWQVPHAMLYR
>P04737
MNAVLSVQGASAPVKKKSFFSKFTRLNMLRLARAVIPAAVLMMFFPQLAMAAGSSGQDLM
ASGNTTVKATFGKDSSVVKWVVLAEVLVGAVMYMMTKNVKFLAGFAIISVFIAVGMAVVG
L
>Q48258
MEIQQTHRKINRPLVSLALVGALVSITPQQSHAAFFTTVIIPAIVGGIATGAAVGTVSGL
LGWGLKQAEEANKTPDKPDKVWRIQAGKGFNEFPNKEYDLYKSLLSSKIDGGWDWGNAAR
HYWVKDGQWNKLEVDMQNAVGTYNLSGLINFTGGDLDVNMQKATLRLGQFNGNSFTSYKD
SADRTTRVDFNAKNILIDNFLEINNRVGSGAGRKASSTVLTLQASEGITSRENAEISLYD
GATLNLASNSVKLMGNVWMGRLQYVGAYLAPSYSTINTSKVTGEVNFNHLTVGDHNAAQA
GIIASNKTHIGTLDLWQSAGLNIIAPPEGGYKDKPNDKPSNTTQNNAKNDKQESSQNNSN
TQVINPPNSAQKTEIQPTQVIDGPFAGGKNTVVNINRINTNADGTIRVGGFKASLTTNAA
HLHIGKGGINLSNQASGRSLLVENLTGNITVDGPLRVNNQVGGYALAGSSANFEFKAGTD
TKNGTATFNNDISLGRFVNLKVDAHTANFKGIDTGNGGFNTLDFSGVTNKVNINKLITAS
TNVAVKNFNINELVVKTNGVSVGEYTHFSEDIGSQSRINTVRLETGTRSIYSGGVKFKGG
EKLVINDFYYAPWNYFDARNIKNVEITNKLAFGPQGSPWGTAKLMFNNLTLGQNAVMDYS

QFSNLTIQGDFVNNQGTINYLVRGGQVATLNVGNAAAMFFSNNVDSATGFYQPLMKINSA
QDLIKNKEHVLLKAKIIGYGNVSAGTDSIANVNLIEQFKERLALYNNNNRMDICVVRNTD
DIKACGTAIGNQSMVNNPENYKYLEGKAWKNIGISKTANGSKISVHYLGNSTPTENGGNT
TNLPTNTTNKVRFASYALIKNAPFARYSATPNLVAINQHDFGTIESVFELANRSNDIDTL
YANSGAQGRDLLQTLLIDSHDAGYARTMIDATSANEITKQLNTATTTLNNIASLEHKTSG
LQTLSLSNAMILNSRLVNLSRRHTNHIDSFAKRLQALKDQRFASLESAAEVLYQFAPKYE
KPTNVWANAIGGTSLNSGGNASLYGTSAGVDAYLNGEVEAIVGGFGSYGYSSFSNQANSL
NSGANNTNFGVYSRIFANQHEFDFEAQGALGSDQSSLNFKSALLRDLNQSYNYLAYSAAT
RASYGYDFAFFRNALVLKPSVGVSYNHLGSTNFKSNSNQKVALKNGASSQHLFNASANVE
ARYYYGDTSYFYMNAGVLQEFANFGSSNAVSLNTFKVNATRNPLNTHARVMMGGELKLAK
EVFLNLGFVYLHNLISNIGHFASNLGMRYSF
>Q54450
MAFSVNYDSSFGGYSIHDYLGQWASTFGDVNHTNGNVTDANSGGFYGGSLSGSQYAISST
ANQVTAFVAGGNLTYTLFNEPAHTLYGQLDSLSFGDGLSGGDTSPYSIQVPDVSFGGLNL
SSLQAQGHDGVVHQVVYGLMSGDTGALETALNGILDDYGLSVNSTFDQVAAATAVGVQHA
DSPELLAA
>P44415
MNSMDKNQQSSQNELDLGLNQEPITPKKTIQPSSSILGKAKGLFAKKNHVQTNFQQRKEP
TFGDSSTQENDPLIPSENLKKVQKPVLQTSSTEENISAVDEEISAENNADEPVEKAEKPI
LAQPEKWKILQVLPAKHRRLFMAIFVLVILLIIFFALKPSSDTVESFTQSNSNEVPVQFQ
SLDQSQPLETTILDNPPAQNQMAVEQANQSEFAPKAEEAANNTTAQNPLVENAPMQQNVV
QSPSQMPNEMAAASVAPMQPAQAEQPKATVPVQPMKKAVEPQVAHKDTVKKEVKVAEKAQ
APAKATEQNVAKTAGNAPIVEAKPVQAKKEKKVQIVDAKPVSKSTASRLSAKTLTVPKGV
SLMQLFRDNQLNISDVNAMSKATGAGNVLSSFKSGDKVTVSVNNQGRVNEMRLSNGARFV
RQSDGSYQYKK
>Q9X6X9
MKTIQGKSPGRWYSRGMLLAAMAASGVIGLAACGGGNDGNSAGNNGNAGGNGNNNGNNNG
NTVSNTKPSFVGTVTVRRFDGVSDDLLTAGLGASGLASATAPAVANAVAPTAAELRRLTI
YNNYRALIDTSAKGGYGTLYGPNVDADGNVTSGNGMVAGAEYVAYPDDGSGQQNVVLLVQ
IPDAFDAAHPCIITATSSGSRGIYGAISTGEWGLKRKCAVAYTDKGTGAGPHDLATDTVP
LQDGTRTTRTLAGNTAQFAAPLAASRLAAFNVATPNRLAFKHAHSQRNPEKDWGLFTLQA
VQFAFWAINDKLGISSGQTVSQLPVRPGNTIVIASSVSNGGGAAIAAAEQDTGNLIDGVA
VGEPALSLPSSINVQVKRGGASLPINGKPLFDYVSYANEFRLCAALSASVASAPTQAYFG
AALGWPASVQANRCAALHAKGLLSSTTTAAQADEALQKMRDYGWEPESDLLHASMAYFEI
DPSVATTFGNALARASVFDNLCDLSFAAVDGSFHPATMNATVLAQLAATGNGVPPTTGVQ
LINNIAQGGAAQSRQSIDSSGTQAANLDGALCLRNLLSGSDAASQALQLGLSQTLRSGNL
RGKPALIVQGRNDALLPVNHGARPYLGLNAQVDGSSKLSYIEVTNAQHFDGFIDLLPGYD
SLFVPLAVYEQRALDAVYANLRSGTPLPPSQVVRTTPRGGAAGAAPPITAANVPNFTMTP
AAGDRIQVSVSGGVATVSVPN
>Q60106
MKIEKTALTVAIALAMSSLSAHAEDAWVSTHTQAAMSPPASTQVLAASSTSATTTGNAYT
LNMTGSPRIDGAAVTALEADHPLHVEVALKLRNPDALQTFLAGVTTPGSALFGKFLTPSQ
FTERFGPTQSQVDAVVAHLQQAGFTNIEVAPNRLLISADGTAGAATNGFRTSIKRFSANG
REFFANDAPALVPASLGDSVNAVLGLQNVSVKHTLHHVYHPEDVTVPGPNVGTQAAAAVA

AHHPQDFAAIYGGSSLPAATNTAVGIITWGSITQTVTDLNSFTSGAGLATVNSTITKVGS
GTFANDPDSNGEWSLDSQDIVGIAGGVKQLIFYTSANGDSSSSGITDAGITASYNRAVTD
NIAKLINVSLGEDETAAQQSGTQAADDAIFQQAVAQGQTFSIASGDAGVYQWSTDPTSGS
PGYVANSAGTVKIDLTHYSVSEPASSPYVIQVGGTTLSTSGTTWSGETVWNEGLSAIAPS
QGDNNQRLWATGGGVSLYEAAPSWQSSVSSSTKRVGPDLAFDAASSSGALIVVNGSTEQV
GGTSLASPLFVGAFARIESAANNAIGFPASKFYQAFPTQTSLLHDVTSGNNGYQSHGYTA
ATGFDEATGFGSFDIGKLNTYAQANWVTGGGGGSTNAPPVANFSVATTGLVATFTDSSTD
SDGSIASHAWTFGDGSTSTATSPSHTYSAAGTYSVAETVTDNAGATSTKTSSVTVSSSGG
TGGGTVLQNGVAATGLSAAKNGQLKYTVAIPSGAKSLKIAISGGTGDADLYVKFGSAPTT
SSYDCRPYVTGNTESCSFASPQTGTYYVLLNGYAAFSGVSLKATWTN
>Q9EZE7
MNKIYALKYCHATGGLIAVSELASRVMKKAARGSLLALFNLSLYGAFLSASQAAQLNIDN
VWARDYLDLAQNKGVFKAGATNVSIQLKNGQTFNFPNVPIPDFSPASNKGATTSIGGAYS
VTATHNGTTHHAISTQNWGQSSYKYIDRMTNGDFAVTRLDKFVVETTGVKNSVDFSLNSH
DALERYGVEINGEKKIIGFRVGAGTTYTVQNGNTYSTGQVYNPLLLSASMFQLNWDNKRP
YNNTTPFYNETTGGDSGSGFYLYDNVKKEWVMLGTLFGIASSGADVWSILNQYDENTVNG
LKNKFTQKVQLNNNTMSLNSDSFTLAGNNTAVEKNNNNYKDLSFSGGGSINFDNDVNIGS
GGLIFDAGHHYTVTGNNKTFKGAGLDIGDNTTVDWNVKGVVGDNLHKIGAGTLNVNVSQG
NNLKTGDGLVVLNSANAFDNIYMASGHGVVKINHSAALNQNNDYRGIFFTENGGTLDLNG
YDQSFNKIAATDIGALITNSAVQKAVLSVNNQSNYMYHGSVSGNTEINHQFDTQKNNSRL
ILDGNVDITNDINIKNSQLTMQGHATSHAVFREGGVTCMLPGVICEKDYVSGIQQQENSA
NKNNNTDYKTNNQVSSFEQPDWENRLFKFKTLNLINSDFIVGRNAIVVGDISANNSTLSL
SGKDTKVHIDMYDGKNITGDGFGFRQDIKDGVSVSPESSSYFGNVTLNNHSLLDIGNKFT
GGIEAYDSSVSVTSQNAVFDRVGSFVNSSLTLEKGAKLTAQGGIFSTGAVDVKENASLIL
TGTPSAQKQEYYSPVISTTEGINLGDKASLSVKNMGYLSSDIHAGTTAATINLGDGDAET
DSPLFSSLMKGYNAVLSGNITGEQSTVNMNNALWYSDGNSTIGTLKSTGGRVELGGGKDF
ATLRVKELNANNATFLMHTNNSQADQLNVTNKLLGSNNTVLVDFLNKPASEMNVTLITAP
KGSDEKTFTAGTQQIGFSNVTPVISTEKTDDATKWMLTGYQTVSDAGASKTATDFMASGY
KSFLTEVNNLNKRMGDLRDTQGDAGVWARIMNGTGSADGGYSDNYTHVQIGADRKHELDG
VDLFTGALLTYTDSNASSHAFSGKTKSVGGGLYASALFDSGAYFDLIGKYLHHDNQYTAS
FASLGTKDYSSHSWYAGAEVGYRYHLSEESWVEPQMELVYGSVSGKSFSWEDRGMALSMK
DKDYNPLIGRTGVDVGRTFSGDDWKITARAGLGYQFDLLANGETVLRDASGEKRFEGEKD
SRMLMNVGMNAEIKDNMRFGLELEKSAFGKYNVDNAINADFRYSF
>P21347
MHPNYYLSPLAVAIALGIASPVKAADPIPLQKSSFSEVTQKFQLTLPGVMKGAVVSTNSL
QFIRQHTDGNKVTHVRMQQQYAGFPVFGGYAILHSKNATPSLATAKSDEKMNGVIYDGLQ
AELGQPKPSFVKNASMALQQFKDKYANKQVSEDQVTPMIYIDEKHQAHWAYKVSVLVIHD
DRIPERPTAIIDAETNKPFVQWDDVKTEKVQAKGMGFGGNRKIGEYQFGKDLPLLEITRD
SSVEMCFMENTDVKVVDMGHKYYSNNKPMQFTCKETPDTQSTKTYYTGYSADGYDRDNGA
ASPTNDALYAGYVIKHMYHDWYGVEALTKSDGSPMQLVMRVHYGQGYENAYWDGKQMTFG
DGDTMMYPLVSLGVGGHEVSHGFTEQHSGLEYFGQSGGMNESFSDMAAQAAEYYSVGKNS
WQIGPEIMKEDSGYDALRYMDKPSRDGMSIDVADDYYGGLDVHYSSGVYNHLFYILANQP
NWNLRMAFDVMVKANMDYWTPYSTFDEGGCGMLSAAKDLGYNLDDIKKSLSEVTINYQSC
YVD

>A5F7A4
MRFKNVKKTALMLAMFGMATSSNAALFDYNATGDTEFDSPAKQGWMQDNTNNGSGVLTNA
DGMPAWLVQGIGGRAQWTYSLSTNQHAQASSFGWRMTTEMKVLSGGMITNYYANGTQRVL
PIISLDSSGNLVVEFEGQTGRTVLATGTAATEYHKFELVFLPGSNPSASFYFDGKLIRDN
IQPTASKQNMIVWGNGSSNTDGVAAYRDIKFEIQGDVIFRGPDRIPSIVASSVTPGVVTA
FAEKRVGGGDPGALSNTNDIITRTSRDGGITWDTELNLTEQINVSDEFDFSDPRPIYDPS
SNTVLVSYARWPTDAAQNGDRIKPWMPNGIFYSVYDVASGNWQAPIDVTDQVKERSFQIA
GWGGSELYRRNTSLNSQQDWQSNAKIRIVDGAANQIQVADGSRKYVVTLSIDESGGLVAN
LNGVSAPIILQSEHAKVHSFHDYELQYSALNHTTTLFVDGQQITTWAGEVSQENNIQFGN
ADAQIDGRLHVQKIVLTQQGHNLVEFDAFYLAQQTPEVEKDLEKLGWTKIKTGNTMSLYG
NASVNPGPGHGITLTRQQNISGSQNGRLIYPAIVLDRFFLNVMSIYSDDGGSNWQTGSTL
PIPFRWKSSSILETLEPSEADMVELQNGDLLLTARLDFNQIVNGVNYSPRQQFLSKDGGI
TWSLLEANNANVFSNISTGTVDASITRFEQSDGSHFLLFTNPQGNPAGTNGRQNLGLWFS
FDEGVTWKGPIQLVNGASAYSDIYQLDSENAIVIVETDNSNMRILRMPITLLKQKLTLSQ
N
>P09545
MPKLNRCAIAIFTILSAISSPTLLANINEPSGEAADIISQVADSHAIKYYNAADWQAEDN
ALPSLAELRDLVINQQKRVLVDFSQISDAEGQAEMQAQFRKAYGVGFANQFIVITEHKGE
LLFTPFDQAEEVDPQLLEAPRTARLLARSGFASPAPANSETNTLPHVAFYISVNRAISDE
ECTFNNSWLWKNEKGSRPFCKDANISLIYRVNLERSLQYGIVGSATPDAKIVRISLDDDS
TGAGIHLNDQLGYRQFGASYTTLDAYFREWSTDAIAQDYRFVFNASNNKAQILKTFPVDN
INEKFERKEVSGFELGVTGGVEVSGDGPKAKLEARASYTQSRWLTYNTQDYRIERNAKNA
QAVSFTWNRQQYATAESLLNRSTDALWVNTYPVDVNRISPLSYASFVPKMDVIYKASATE
TGSTDFIIDSSVNIRPIYNGAYKHYYVVGAHQSYHGFEDTPRRRITKSASFTVDWDHPVF
TGGRPVNLQLASFNNRCIQVDAQGRLAANTCDSQQSAQSFIYDQLGRYVSASNTKLCLDG
EALDALQPCNQNLTQRWEWRKGTDELTNVYSGESLGHDKQTGELGLYASSNDAVSLRTIT
AYTDVFNAQESSPILGYTQGKMNQQRVGQDHRLYVRAGAAIDALGSASDLLVGGNGGSLS
SVDLSGVKSITATSGDFQYGGQQLVALTFTYQDGRQQTVGSKAYVTNAHEDRFDLPAAAK
ITQLKIWSDDWLVKGVQFDLN
>P0A4M3
MRNLFIALMLLFSSIAFSQTVENNKKTVQQPQQIESKVNIKKLSENEECPFIKQVDENGN
LIDCCEICCNPACFGCLN
>Q5SF96
MIVTYGTVGCPVSRGGSPGCGRRIAEELRLAEDARLRLALLGRCIVKGSPAQARGELRAE
LKAIDATIELRKELDAIDAEWAPKIELSAELRAIDAEWRPAIRLRSAYRAIIGRIELRKE
LDAIDAEWAPKIELSAELKAIDAEWRPAIRLRSAYRAIIGRWELSKELKAIDAEWRPAIA
RESLRKELDAIDAEWQHAITFWHISRAIIGSIELSKELKAIDAKWKYVAIYERQKAQRRR
EERAAKAREELRKELNDIDAKWKSASAIKLRKDLRSTSEGVDHTEFALELRATDKSGNME
LVLKLKATDTKNQHDAIVKAIEDGFVGYAAECGAATRELNACGGMSTTSAPSTDLISTVV
SAVTTGTGQQQSAGSESQRPTECGGGGTLLSSLFALILPSSNWYCNAVLYGGFLGCLGS
>P08062
MDPKAEGNGENITETAAGNVETSDFVNLKRQKREGVNSTGMSEIDMTGSQETPEHNMHGS
PTHTDDLGPRLDADMLDSQSSHVSSSAQGNRSEVENELSNLFAKMALPGHDRRTDEYILV
RQTGQDKFAGTTKCNLDHLPTKAEFNASCRLYRDGVGNYYPPPLAFERIDIPEQLAAQLH

NLEPREQSKQCFQYKLEVWNRAHAEMGITGTDIFYQTDKNIKLDRNYKLRPEDRYIQTEK
YGRREIQKRYEHQFQAGSLLPDILIKTPQNDIHFSYRFAGDAYANKRFEEFERAIKTKYG
SDTEIKLKSKSGIMHDSKYLESWERGSADIRFAEFAGENRAHNKQFPAATVNMGRQPDGQ
GGMTRDRHVSVDYLLQNLPNSPWTQALKEGKLWDRVQVLARDGNRYMSPSRLEYSDPEHF
TQLMDQVGLPVSMGRQSHANSVKFEQFDRQAAVIVADGPNLREVPDLSPEKLQQLSQKDV
LIADRNEKGQRTGTYTNVVEYERLMMKLPSDAAQLLAEPSDRYSRAFVRPEPALPPISDS
RRTYESRPRGPTVNSL
>P13734
MLKVIPWLLVTSSLVAIPTYIHATTEVVVNLNVKHSVEGKSEFERKNHIKLHSTLNDNDW
QGEEDKLKYMMEELDVYFGRDNGGTVWNFNQAIEDPANIGYADPQNIIARGQAQRETNWG
QNKSALHQYDGRGDLMIGGQPRAHYLGNTSPCCGGSAWQAKGGDAVGDFLGQYVNEFFRS
AGDPVTKGHLAPVYFEVLNEPLYQVTDAPHELGLEQPIPPIDIFTFHNDVADAFRQHNTH
IKIGGFTVAFPIFEQREFARWEERMKLFIDTSGSHMDVYSTHFYDLEDDNRFKGSRLEAT
LDMIDQYSLLALGETKPHVISEYGGRNRPMENAPWSALRDWWFLKTASPMLMQFLSRPDS
VLTSIPFVPIKALWGTAADGTPYNWRLLRQQKEAPNETGENWVFTEMVKFYQLWSDVKGT
RVDTFSTNSDFLIDSYVQNDKAYVLISNLTEQAEKIVVHKYGAPASSQPTTRIKHLYLKG
AAPRLMKQVMRQISKKSRLLLKRLW
>Q01099
MSLNTSGLGASTMQISIGGAGGNNGLLGTSRQNAGLGGNSALGLGGGNQNDTVNQLAGLL
TGMMMMMSMMGGGGLMGGGLGGGLGNGLGGSGGLGEGLSNALNDMLGGSLNTLGSKGGNN
TTSTTNSPLDQALGINSTSQNDDSTSGTDSTSDSSDPMQQLLKMFSEIMQSLFGDGQDGT
QGSSSGGKQPTEGEQNAYKKGVTDALSGLMGNGLSQLLGNGGLGGGQGGNAGTGLDGSSL
GGKGLQNLSGPVDYQQLGNAVGTGIGMKAGIQALNDIGTHSDSSTRSFVNKGDRAMAKEI
GQFMDQYPEVFGKPQYQKGPGQEVKTDDKSWAKALSKPDDDGMTPASMEQFNKAKGMIKS
AMAGDTGNGNLQARGAGGSSLGIDAMMAGDAINNMALGKLGAA
>P29484
MNIKLSFISIAFLSLSFNVAANEFEKSQEHYKSVTDLKNKIEILELEKKITELSGEIRNA
RMPKIDKSAPVLSPQPVVKSSEELQKSIEHIEEELKVELAYLVNNGQQKKYTFNLNGKLI
TLVNGDFVNGWKFIEDQNKIQFSKGNKVIDVN
>P40600
MKKKLIYAAVVSALLAGCGGSDDNKGDTSSYLDYLLTGSNAVGPSALAARAWDGTLKFST
ETADLSNPVSAMSTLDGWSTTQAIQIVPVTSSGITVQAPTTAEFGASVAPLYLLEVTFDS
TALRPSGVKKVLTYGVDFVVAASAWQAEPGSAQAVEPLPCLANDSGHRTAERQSRRCLKA
GSDYGNYKNNAGSNAQEQTINGLIALQEGLFKAATGIATDHVIFSDWFGTQSGADVLVAV
KGAAASVLKADPVTLDAAKLWKQDAWEHQPARHLYPGRDRPTCLPDPAGCRAVPAAEQKD
AIATAFGPVLRSTRLLKRPRSIPVPSSCLTSSPHRRPQVPGARPRPSPGTVPSQPVRHRQ
CAEGVTRSDRRAGGGGRGSGPAGDADCRSDPPERAAGRGEQADWGDAHLRRQAAGRRAEH
WSLQPAADAGRGAIRADACLRQGCPQHHHGCHHLSARRDLGQRERLRPGAGPDLEDLCRH
AGGQEGGAGGDRSSAARRAWLRLSGSMDTVTTSDNPTPYLNLSYLTVARDNLKQSVAICW
ACVWRLAWPTPRAIGTAGSLKVHFLGHSLGASRVPTCCGRQPDHRQRASGCPVQVRYRWP
GHAGSHSAAAAELADFGPTIKMGVLTSGSAELKAGFTAYAPNCTDGGAYLLRQRVPAEPG
RGHSATAATRCRVQLCGPVGAGFG
>P0C1A4
MNNSRMSSVSTQKTTGRSALGTKSALAAIIATTMMVSVASAASLQTTKATEAASTGWATQ

SGGTTGGAKASSSKIYAVKSISEFKAALNGTDSSPKIIQVTGAIDISGGKAYTSFDDQKA
RSQISIPSNTTIIGIGNKGKFTNGSLVVKGVSNVILRNLYIETPVDVAPHYEEGDGWNAE
WDAVVIDSTDHVWVDHVTISDGSFTDDKYTTKNGEKYVQHDGSLDIKRGSDYVTVSNSRF
ELHDKTILIGHSDNNGSQDAGKLRVTFHNNLFDRVGERTPRVRFGSVHAYNNVYVGDVNH
KAYRYQYSFGIGTSGSLLSESNAFTIDNMKKISGRDKECSVVKAFNGKIFSDKGSIINGA
SYNLNGCGFGFSAYSAKIPYKYSAQTITTSLAGSISSNAGYGKL
>Q03475
MSSIDPATFAAQFAQIEIQPFKQRYQLQTNTYQSQLSALGKVESAMREFRTALNEMNSST
NSIIKNSTSISQEGYFTANADAKALSGSYQIFVEQVATSHQVSTGMPADLDATTEIPKTG
NLEFTINGKTMTIDLSTVDTDGDGVTTVSDLTKAINNNSDNPGVNATLVRSNGQTHFMLS
STETGVANQINVSATGTGQAWFEDAFTNLSQISAPQDAVIWLGAEKTGLKLTNSSNTFEG
VIDGVDITVTKAQTSGETAIGLGIGADDEATKEQLNKFVDAYNTLISTIDEHTQIGSEDK
KRGVLASDPTMRSIESQLSSLVRGEHGGMRLSEIGVTLDRHGKLKVDQEKFAEAQKNNSA
GLEAMFNGDGALLDSMDAMAEPFLKFSSGAFKSRKEALQANLDRLSDKQTTLERKYDMSY
KRYLKQFTQMNTLMTQMNQTMSMFG
>Q59478
MLKSGVMVASLCLFSVPSRAAVPAPGDKFELSGWSLSVPVDSDNDGKADQIKEKTLAAGY
RNSDFFTLSDAGGMVFKAPISGAKTSKNTTYTRSELREMLRKGDTSIATQGVSRNNWVLS
SAPLSEQKKAGGVDGTLEATLSVDHVTTTGVNWQVGRVIIGQIHANNDEPIRLYYRKLPH
HQKGSVYFAHEPRKGFGDEQWYEMIGTLQPSHGNQTAAPTEPEAGIALGETFSYRIDATG
NKLTVTLMREGRPDVVKTVDMSKSGYSEAGQYLYFKAGVYNQNKTGKPDDYVQATFYRLK
ATHGAQR
>P74873
MLKYEERKLNNLTLSSFSKVGVSNDARLYIAKENTDKAYVAPEKFSSKVLTWLGKMPLFK
NTEVVQKHTENIRVQDQKILQTFLHALTEKYGETAVNDALLMSRINMNKPLTQRLAVQIT
ECVKAADEGFINLIKSKDNVGVRNAALVIKGGDTKVAEKNNDVGAESKQPLLDIALKGLK
RTLPQLEQMDGNSLRENFQEMASGNGPLRSLMTNLQNLNKIPEAKQLNDYVTTLTNIQVG
VARFSQWGTCGGEVERWVDKASTHELTQAVKKIHVIAKELKNVTAELEKIEAGAPMPQTM
SGPTLGLARFAVSSIPINQQTQVKLSDGMPVPVNTLTFDGKPVALAGSYPKNTPDALEAH
MKMLLEKECSCLVVLTSEDQMQAKQLPPYFRGSYTFGEVHTNSQKVSSASQGEAIDQYNM
QLSCGEKRYTIPVLHVKNWPDHQPLPSTDQLEYLADRVKNSNQNGAPGRSSSDKHLPMIH
CLGGVGRTGTMAAALVLKDNPHSNLEQVRADFRDSRNNRMLEDASQFVQLKAMQAQLLMT
TAS
>P52090
MLAKQIKKANSRSTLLRKSLLFAAPIILAVSSSSVYALTQVSNFGTNPGNLQMFKHVPSG
MPANAPLVVALHGCTQTAAAYEASGWSALGNTHKFYVVYPQQQSGNNSNKCFNWFEPGDI
TRGQGEALSIKQMVDNMKANHSIDPSRVYVTGLSAGAFMTTVMAATYPDVFAGAAPIAGG
PYKCATSMTSAFTCMSPGVDKTPAAWGDLARGGYSGYNGPKPKISIWHGSSDYTVAPANQ
NETVEQFTNYHGIDQTPDVSDTVGGFPHKVYKSANGTPLVETYTITGMGHGTPVDPGTGA
NQCGTAGAYILDVNVCSSYYIGQFFGIIGGGGTTTTTTSGNVTTTTAATTTTTTATQGYT
QTTSATVTNHYVAGRINVTQYNVLGARYGYVTTIPLYYCPSLSGWTDKANCSPI
>P96747
MMISDATMMQQNYYLNNAQKASDKALENIAAVRAISGVDSANLAIADSLRSQSSTIDQGV
ANAYDAIGVLQIADASLTNISQSADRLNELSVKMNNAALNDSQKGMLRTEATRIQESIND

SFNNATYNGKNVFQTMNFVVGSGTETTNLNPLATDGLSIDSQDSITNFMDQLGSLRSEIG
SGINAITSNINASVQNSINSKAAENNLLNNDMAKNVNDFNANYLKENAAAFVAAQSNMQL
QSKIANLLQ
>Q887C6
MQALNSISSLQTSASLFPVSLNSDVSANTSTSSKELKAVIDQLVQALTQSGQLDETSPLG
KMLAKAMAADGKSANSIDDITASLDKLIHEKLGDNFGASAGIGAGGGGGGIGGAGSGSGV
GGGLSSDAGAGQSDLMSQVLNGLGKAVLDDLLTPSGEGGTTFSSDDMPTLEKVAQFMDDN
KAQFPTRDGGSWMNELKEDNGLDAQETAQFRSALDVIGQQLGQQQGDASGVTSGGGLGSP
VSDSSLGNPAIDANTGPAANGNASVDVGQLIGQLIDRGLQSVSSGGGLGTPVDNSTQPTG
GTPAANPTGNVSNQDLGQLLSGLLQRGLEATLQDAGNTGADLQSSAAQVAAQLINALLQG
TNNQTNQAVA
>Q2QCI9
MAGINGAGPSGAYFVGHTDPEPASGGAHGSSSGASSSNSPRLPAPPDAPASQARDRREML
LRARPLSRQTREWVAQGMPPTAEAGVPIRPQESAEAAAPQARAEERHTPEADAAASHVRT
EGGRTPQALAGTSPRHTGAVPHANRIVQQLVDAGADLAGINTMIDNAMRRHAIALPSRTV
QSILIEHFPHLLAGELISGSELATAFRAALRREVRQQEASAPPRTAARSSVRTPERSTVP
PTSTESSSGSNQRTLLGRFAGLMTPNQRRPSSASNASASQRPVDRSPPRVNQVPTGANRV
VMRNHGNNEADAALQGLAQQGVDMEDLRAALERHILHRRPIPMDIAYALQGVGIAPSIDT
GESLMENPLMNLSVALHRALGPRPARAQAPRPAVPVAPATVSRRPDSARATRLQVIPARE
DYENNVAYGVRLLSLNPGAGVRETVAAFVNNRYERQAVVADIRAALNLSKQFNKLRTVSK
ADAASNKPGFKDLADHPDDATQCLFGEELSLTSSVQQVIGLAGKATDMSESYSREANKDL
VFMDMKKLAQFLAGKPEHPMTRETLNAENIAKYAFRIVP
>P96786
MAIGSLSSLGLGSKVLNYDVIDKLKDADEKALIAPLDKKMEQNVEKQKALVEIKTLLSAL
KGPVKTLSDYSTYISRKSNVTGDALSASVGVGVPIQDIKVDVQNLAQGDINELGAKFSSR
DDIFSQVDTTLKFYTQNKDYAVNIKAGMTLGDVAQSITDATNGEVMGIVMKTGGNDPYQL
MVNTKNTGEDNRVYFGSHLQSTLTNKNALSLGVDGSGKSEVSLNLKGADGNMHEVPIMLE
LPESASIKQKNTAIQKAMEQALENDPNFKNLIANGDISIDTLHGGESLIINDRRGGNIEV
KGSKAKELGFLQTTTQESDLLKSSRTIKEGKLEGVVSLNGQKLDLSALTKESNTSEENTD
AIIQAINAKEGLSAFKNAEGKLVINSKTGMLTIKGEDALGKASLKDLGLNAGMVQSYEAS
QNTLFMSKNLQKASDSAFTYNGVSITRPTNEVNDVISGVNITLEQTTEPNKPAIISVSRD
NQAIIDSLTEFVKAYNELIPKLDEDTRYDADTKIAGIFNGVGDIRAIRSSLNNVFSYSVH
TDNGVESLMKYGLSLDDKGVMSLDEAKLSSALNSNPKATQDFFYGSDSKDMGGREIHQEG
IFSKFNQVIANLIDGGNAKLKIYEDSLDRDAKSLTKDKENAQELLKTRYNIMAERFAAYD
SQISKANQKFNSVQMMIDQAAAKKN
>Q56019
MVNDASSISRSGYTQNPRLAEAAFEGVRKNTDFLKAADKAFKDVVATKAGDLKAGTKSGE
SAINTVGLKPPTDAAREKLSSEGQLTLLLGKLMTLLGDVSLSQLESRLAVWQAMIESQKE
MGIQVSKEFQTALGEAQEATDLYEASIKKTDTAKSVYDAATKKLTQAQNKLQSLDPADPG
YAQAEAAVEQAGKEATEAKEALDKATDATVKAGTDAKAKAEKADNILTKFQGTANAASQN
QVSQGEQDNLSNVARLTMLMAMFIEIVGKNTEESLQNDLALFNALQEGRQAEMEKKSAEF
QEETRKAEETNRIMGCIGKVLGALLTIVSVVAAVFTGGASLALAAVGLAVMVADEIVKAA
TGVSFIQQALNPIMEHVLKPLMELIGKAITKALEGLGVDKKTAEMAGSIVGAIVAAIAMV
AVIVVVAVVGKGAAAKLGNALSKMMGETIKKLVPNVLKQLAQNGSKLFTQGMQRITSGLG

NVGSKMGLQTNALSKELVGNTLNKVALGMEVTNTAAQSAGGVAEGVFIKNASEALADFML
ARFAMDQIQQWLKQSVEIFGENQKVTAELQKAMSSAVQQNADASRFILRQSRA
>Q51718
MPLRTLLCGLLLAVCLGQHALAASRCSERPRTLLRPAEVSCSYQSTWLDSGLVGQRKIIY
QTPLGTPPAGGWPVVLIYQGSFFPLNDFSYHSNLPFGGYYEGKLVQNLLDHGYAVIAPSA
PADLFWQTNIPGLAQAYELSTDYDFLGNVLAAIASGHFGPLNAQRQYATGISSGGYNTSR
MAVSFPGKFRALAVQSGSYATCSGPLCVVPDQLPADHPPTLFLHGFVDAVVPWWSMDLYY
DRLLHQGIETARYTEPLGGHEWFAASPGKVLAWFNAHP
>P08715
MTTITTAQIKSTLQSAKQSAANKLHSAGQSTKDALKKAAEQTRNAGNRLILLIPKDYKGQ
GSSLNDLVRTADELGIEVQYDEKNGTAITKQVFGTAEKLIGLTERGVTIFAPQLDKLLQK
YQKAGNILGGGAENIGDNLGKAGGILSTFQNFLGTALSSMKIDELIKKQKSGGNVSSSEL
AKASIELINQLVDTVASLNNNVNSFSQQLNTLGSVLSNTKHLNGVGNKLQNLPNLDNIGA
GLDTVSGILSAISASFILSNADADTRTKAAAGVELTTKVLGNVGKGISQYIIAQRAAQGL
STSAAAAGLIASAVTLAISPLSFLSIADKFKRANKIEEYSQRFKKLGYDGDSLLAAFHKE
TGAIDASLTTISTVLASVSSGISAAATTSLVGAPVSALVGAVTGIISGILEASKQAMFEH
VASKMADVIAEWEKKHGKNYFENGYDARHAAFLEDNFKILSQYNKEYSVERSVLITQQHW
DTLIGELAGVTRNGDKTLSGKSYIDYYEEGKRLEKKXDEFQKQVFDPLKGNIDLSDSKSS
TLLKFVTPLLTPGEEIRERRQSGKYEYITELLVKGVDKWTVKGVQDKGAVYDYSNLIQHA
SVGNNQYREIRIESHLGDGDDKVFLSAGSANIYAGKGHDVVYYDKTDTGYLTIDGTKATE
AGNYTVTRVLGGDVKVLQEVVKEQEVSVGKRTEKTQYRSYEFTHINGKNLTETDNLYSVE
ELIGTTRADKFFGSKFTDIFHGADGDDLIEGNDGNDRLYGDKGNDTLSGGNGDDQLYGGD
GNDKLIGVAGNNYLNGGDGDDEFQVQGNSLAKNVLFGGKGNDKLYGSEGADLLDGGEGDD
LLKGGYGNDIYRYLSGYGHHIIDDDGGKEDKLSLADIDFRDVAFKREGNDLIMYKGEGNV
LSIGHKNGITFRNWFEKESGDISNHEIEQIFDKSGRIITPDSLKKALEYQQRNNKASYVY
GNDALAYGSQGDLNPLINEISKIISAAGSFDVKEERTAASLLQLSGNASDFSYGRNSITL
TTSA
>P55127
MNEGEVVLTPEQIQTLRGYASRGDTYGGWRYLANLGDRYADNAAAIVGKDTNLNGLNLWM
KKGVENLWDDTVGKKTRLEKFDRVALQHFSQYVDLINKNNGRLPNTSEIERSYYKAVTYH
GVSSSAAIDLVINRSLPDMADGYWALGLGIEAERIHNEQAVNNPNGSERDNRKQLISALD
KGFDGSFKEKHFTFLQSVMMDLTKLGVEYTIDGWQKIGGWGNGIINDLYKSVVKREWTGI
FEIVNNNIKQGNEAFKNEINSLVHDMKAAGKEFGDDLNTQWNNLTQAAEIIYNDIVDNTS
QGIEKGVKAIKELSEKMKNAASDLADGSAEKAKQVVEDLAQAAKEAYENAKSTAEKAAQA
AREFFKGLPSFKDLAEKFRDLFPNPEGWIDDGHQCFAPWVKETKKRNGKYHVYDPLALDL
DGDGIETVATKGFSGSLFDHNRDGIRTATGWVAADDGLLVRDLNGNGIIDNGAELFGDNT
KLADGSFAKHGYAALAELDSNGDNIINAADAAFQTLRVWQDLNQDGISQANELRTLEELG
IQSLDLAYKDVNKNLGNGNTLAQQGSYTKTDGTTAKMGDLLLAADNLHSRFKDKVELTAE
QAKAANLAGIGRLRDLREAAALSGDLANMLKAYSAAETKEAQLALLDNLIHKWAETDSNW
GKKSPMRLSTDWTQTANEGIALTPSQVAQLKKNALVSLSDKAKAAIDAARDRIAVLDAYT
GQDSSTLYYMSEEDALNIVKVTNDTYDHLAKNIYQNLLFQTRLQPYLNQISFKMENDTFT
LDFSGLVQAFNHVKETNPQKAFVDLAEMLAYGELRSWYEGRRLMADYVEEAKKAGKFEDY
QKVLGQETVALLAKTSGTQADDILQNVGFGHNKNVSLYGNDGNDTLIGGAGNDYLEGGSG
SDTYVFGKGFGQDTVYNYDYATGRKDIIRFTDGITADMLTFTREGNHLLIKAKDDSGQVT

VQSYFQNDGSGAYRIDEIHFDNGKVLDVATVKELVQQSTDGSDRLYAYQSGNTLNGGLGD
DYLYGADGDDLLNGDAGNDSIYSGNGNDTLNGGEGNDALYGYNGNDALNGGEGNDHLNGE
DGNDTLIGGAGNDYLEGGSGSDTYVFGKGFGQDTVYNYDYATGRKDIIRFTDGITADMLT
FTREGNHLLIKAKDGSGQVTVQSYFQNDGSGAYRIDEIHFDNGKVLDVATVKELVQQSTD
GSDRLYAYQSGNTLNGGLGDDYLYGADGDDLLNGDAGNDSIYSGNGNDTLDGGEGNDALY
GYNGNDALNGGEGNDHLNGEDGNDTLIGGAGNDYLEGGSGSDTYVFGKGFGQDTVYNYDY
ATGRKDIIRFTDGITADMLTFTREGNHLLIKAKDDSGQVTVQSYFQNDGSGAYRIDEIHF
DNGKVLDVATVKELVQQSTDGSDRLYAYQSGSTLNGGLGDDYLYGADGDDLLNGDAGNDS
IYSGNGNDTLDGGEGNDALYGYNGNDALNGGEGNDHLNGEDGNDTLIGGAGNDYLEGGSG
SDTYVFGKGFGQDTVYNYDYATGRKDIIRFTDGITADMLTFTREGNHLLIKAKDGSGQVT
VQSYFQNDGSGAYRIDEIHFDNGKVLDVATVKKLVQQSTDGSDRLYAYQSGNTLNGGLGD
DYLYGADGDDLLNGDAGNDSIYSGNGNDTLNGGEGNDALYGYNGNDVLNGGEGNDHLNGE
DGNDTLIGGAGNDYLEGGSGSDTYVFGKGFGQDTVYNYHVDKNSDTMHFKGFKAADVHFI
RSGSDLVLSASEQDNVRISGFFYGENHRVDTFVFDDAAISNPDFAKYINAGNNLVQSMSV
FGSNTAATGGNVDANTQSVQQPLLVTPSA

>Q8ZNG2

MNICVNSLYRLSIPQFHSLYTEEVSDEALTLLFSAVENGDQNCIDLLCNLALRNDDLGHR
VEKFLFDLFSGKRTGSSDIDKKINQACLVLHQIANNDITKDNTEWKKLHAPSRLLYMAGS
ATTDLSKKIGIAHKIMGDQFAQTDQEQVGVENLWCGARMLSSDELAAATQGLVQESPLLS
VNYPIGLIHPTTKENILSTQLLEKIAQSGLSHNEVFLVNTGDHWLLCLFYKLAEKIKCLI
FNTYYDLNENTKQEIIEAAKIAGISESDEVNFIEMNLQNNVPNGCGLFCYHTIQLLSNAG
QNDPATTLREFAENFLTLSVEEQALFNTQTRRQIYEYSLQ

>P33219

MKKRGAFLGLLLVSACASVFAANNETSKSVTFPKCEDLDAAGIAASVKRDYQQNRVARWA
DDQKIVGQADPVAWVSLQDIQGKDDKWSVPLTVRGKSADIHYQVSVDCKAGMAEYQRR

>Q08860

MAQVINTNSLSLITQNNINKNQSALSSSIERLSSGLRINSAKDDAAGQAIANRFTSNIKG
LTQAARNANDGISVAQTTEGALSEINNNLQRIRELTVQASTGTNSDSDLDSIQDEIKSRL
DEIDRVSGQTQFNGVNVLAKDGSMKIQVGANDGQTITIDLKKIDSDTLGLNGFNVNGGGA
VANTAASKADLVAANATVVGNKYTVSAGYDAAKASDLLAGVSDGDTVQATINNGFGTAAS
ATNYKYDSASKSYSFDTTTASAADVQKYLTPGVGDTAKGTITIDGSAQDVQISSDGKITA
SNGDKLYIDTTGRLTKNGSGASLTEASLSTLAANNTKATTIDIGGTSISFTGNSTTPDTI
TYSVTGAKVDQAAFDKAVSTSGNNVDFTTAGYSVNGTTGAVTKGVDSVYVDNNEALTTSD
TVDFYLQDDGSVTNGSGKAVYKDADGKLTTDAETKAATTADPLKALDEAISSIDKFRSSL
GAVQNRLDSAVTNLNNTTTNLSEAQSRIQDADYATEVSNMSKAQIIQQAGNSVLAKANQV
PQQVLSLLQG

>P09489

MILNKRLKLAYCVFLGCYGLSIHSSLAAYQDPGRLGAPDSWKTAEFNRQWGLEAISAEFA
YARGYTGKGITIGVIDNAILSHSEFSGKLTRLDNGSYNFSYDKQDNMSFGDHGTHVAGIA
AAKRDGAGMHGVAFDADIIGTKLNDYGNRNGREELIQSAARVINNSWGIAPDIRRDAKGD
IIWLPNGRPDYVAFVKSEVIAEMMRSKSSVEWGSEQPVPTGGHSAMSTLLRAARHGKLIV
FSAGNYNNYNIPEAQKSLPYAFPDVLNNYLIVTNLSDENQLSVSSTSCGQTASYCVSAPG
SDIYSTVGRLESNTGGAVNREAYNKGELSLNPGYGNKSGTSMAAPHVTGVAAVLMQRFPY
MSADQISAVIKTTATDLGVAGIDNLFGWGRVNLRDAINGPKMFITKEDIPQEYYVPGSYS

EKQFVVNIPGLGNIVEPGTPVERRCTSSECSFDSWSNDISGHGGLTKTGAGTLALLGNNT
YRGDTWVKQGVLAIDGSVASNVYIENSGTLSGEGTVGAFRAARSGSVAPGNGIGTLHVLH
DAIFDRGSQYNVEVADNGRSDKIAARRAFLNGGSVNVSLERSQNLLSQNEAQSLLGNKYT
ILTTTDGVTGRFENANPSYPFVKVALDYRGNDVGLGITRTDASFDSLASTENEKAVARAV
ETLNATEPVTETAKRSVAIPAAEEANLLQSDGGEAQAVNEEASIVAGHPIYESFLGFTSA
RELQQATRQLSGQIHADMASAQINESRYLRDTATERLRQAEGRRTATDIKADDNGAWAKL
LGSWGHASGNDNATGYQTSTYGVLLGLDSELFGDGRLGMMTGYTRTSLDGGYQSDAHSDN
YHLGLYGDKRFGALALRAGGTYTWHRIDTSRSVNYGAQSDREKAKYNARTGQLFIESGYD
WTSDAVNLEPFANLAYTHYRNEEINEQGGAAALRGDKQSQSATASTLGLRADTEWQTDSV
AIALRGELGWQHQYGKLERKTQLMFKRTDAAFDVNSVPVSRDGAILKAGVDVSINKNAVL
SLGYGGQLSSNHQDNSVNAGLTWRF

(5) $\mathbb{S}_5$: 32 fimbrium proteins

>P11900
MKKTLIALAVAASAAVSGSVMAADWTEGQPGDIIIGGEITSPSVKWLWKTGEGLSSFSNT
TNEIVKRKLNISVPTDELFLAAKMSDGIKGVFVGNTLIPKIEMASYDGSVITPSFTSNTA
MDIAVKVKNSGDNTELGTLSVPLSFGAAVATIFDGDTTDSAVAHIIGGSAGTVFEGLVNP
GRFTDQNIAYKWNGLSKAEMAGYVEKLMPGQSASTSYSGFHNWDDLSHSNYTSANKASYL
SYGSGVSAGSTLVMNLNKDVAGRLEWVAPVTITVIYS
>P53508
MKKTIGLILILASFGSHARTEIATKNFPVSTTISKSFFAPEPQIQPSFGKNVGKEGGLLF
SVSLTVPENVSQVTVYPVYDEDYGLGRLVNTADDSQSIIYQIVDDKGRKMLKDHGAEVTP
NQQITFRALNYTSGDKEIPPGIYNDQVMVGYYVN
>P20862
MIKKVPVLLFFMASISITHASQTATKSLGVSITLSKAQCKINNRAGISGSFVLPMISTSG
QIISSKKFTTVPIIIDCTAGGNVNQLEITFGDNSSKKIDSTTWYTTNKDLGLRFSWTKDK
TQGFNLGVAHNINKSIWLEGNKKFNASVDVSPVVIRNTVQGGQYTSALPVTVTFI
>P08180
MKKLAIIGATSVMMMTGTAQANFTSSGTNGKVDLTITEECRVTVESKSESFLRSGLVANR
HITNLGIQSTGCGTGQRVALKLGAGSYDDTNGAHMTHENGTDKLLVSMGSATGDGTQDGG
VYYINRDGNWNGQMVFIVRNDQQHLPTGKYTLNLEGGFWTK
>P07111
MRLRFSVPLFFFGCVFVHGVFAGPFPPPGMSLPEYWGEEHVWWDGRAAFHGEVVRPACTL
AMEDAWQIIDMGETPVRDLQNGFSGPERKFSLRLRNCEFNSQGGNLFSDSRIRVTFDGVR
GETPDKFNLSGQAKGINLQIADVRGNIARAGKVMPAIPLTGNEEALDYTLRIVRNGKKLE
AGNYFAVLGFRVDYE
>P62607
MIKSVIAGAVAMAVVSFGAYAAPTIPQGQGKVTFNGTVVDAPCGIDAQSADQSIDFGQVS
KLFLENDGESQPKSFDIKLINCDITNFKKAAGGGGAKTGTVSLTFSGVPSGPQSDMLQTV
GATNTAIVVTDPHGKRVKFDGATATGVSYLVDGDNTIHFTAAVRKDGSGNPVTEGAFSAV
ANFNLTYQ
>P22595
MKKVLLPLAALVLSATASNAMAANGTVKFTGEIKQSTCQVTSDTQNKEVYLGTYPTSAFP
TVGSKSASKAFQISLEKCDAGDYSLRFDGNTVAGNPDLLSVSNVGGTGAAATGVGIEITD

NNGKPFAIGDGSNINDDVAKVTIAADGKATFNLQARYRSFDSNVTAGLANATSPFTIEYK

>P33781
MKKNLLITSVLAMATVSGSVLAAVTNGQLTFNWQGVVPSAPVTQSSWAFVNGLDIPFTPG
TEQLNITLDSNKDITARSVKPYDFFIVPVSGNVTPGAPVTRDTSANINSVNAFLSSVPVS
NGFVGNKQLTLSTAVEAAKGEVAITLNGQALKVGSASPTVVTVASNKKESHISIDMNAKA
AAADVAEGAAINFVAPVTFAVDI
>P02970
MKKTLIALAIAASAASGMAHAWMTGDFNGSVDIGGSITADDYRQKWEWKVGTGLNGFGNV
LNDLTNGGTKLTITVTGNKPILLGRTKEAFATPVSGGVDGIPQIAFTDYEGASVKLRNTD
GETNKGLAYFVLPMKNAEGTKVGSVKVNASYAGVFGKGGVTSADGELFSLFADGLRAIFY
GGLTTTVSGAALTSGSAAAARTELFGSLSRNDILGQIQRVNANITSLVDVAGSYREDMEY
TDGTVVSAAYALGIANGQTIEATFNQAVTTSTQWSAPLNVAITYY
>P08191
MKRVITLFAVLLMGWSVNAWSFACKTANGTAIPIGGGSANVYVNLAPVVNVGQNLVVDLS
TQIFCHNDYPETITDYVTLQRGSAYGGVLSNFSGTVKYSGSSYPFPTTSETPRVVYNSRT
DKPWPVALYLTPVSSAGGVAIKAGSLIAVLILRQTNNYNSDDFQFVWNIYANNDVVVPTG
GCDVSARDVTVTLPDYPGSVPIPLTVYCAKSQNLGYYLSGTTADAGNSIFTNTASFSPAQ
GVGVQLTRNGTIIPANNTVSLGAVGTSAVSLGLTANYARTGGQVTAGNVQSIIGVTFVYQ

>P25448
MKNKYNLLFFLFLLCYGDVALAACTGKLKISPGYSGHTYSFDSSIPNNSNIARYLVEISE
KIVCDADQSGWDGKRYAQLHLYSSGALCESVSGDGITFRSNVSGLSWRFPNGIPYHCAAG
QINLGGIKYADRNGKVTWNPGELRHEIFLRVDNRFDFSKSRTFSVNTISGRGGLGGDSSV
VIPLIGSSFNYSYSNIATCTLTGPSEVNFNTVTTSDVLKGTTHRDLNLRAECRNRGASLG
LNFKFEPQYKDVSANKQGVFYAKNTSGSLTYKLTKKADASAIPLNEFVKLIVEDKVNIHT
GNTIPLLLTLQKGDGKIATGKIETFLNVTMEHM
>P13429
MVKDIIKTVTFSCMLAGSMFVTCHVCAAGSVVNITGNVQDNTCDVDINSRNFDVSLGSYD
SRQFTAAGDTTPASVFHVGLTSCGSAVRAVKLTFTGTPDNQEAGLIQINSINGARGVGIQ
LLDKDKHELKINVPTTIALMPGTQTIAFYARLKATYLPVKAGNVDAVVNFVLDYQ
>P0ABW7
MKLKKTIGAMALATLFATMGASAVEKTISVTASVDPTVDLLQSDGSALPNSVALTYSPAV
NNFEAHTINTVVHTNDSDKGVVVKLSADPVLSNVLNPTLQIPVSVNFAGKPLSTTGITID
SNDLNFASSGVNKVSSTQKLSIHADATRVTGGALTAGQYQGLVSIILTKST
>P21648
MKKLTLFIGLMALGTTSAWASCWQSNSAYEINMAMGRVVVSPDLPVGSVIATKTWTMPDN
NTIYVTCDRNTTLKSDAKVVAAGLVQGANKVYSTAIPGIGLRFSRKGAISMIYPDSYTTT
GSSFRLVGSTFTLDIIKTSTTTGSGTLASGPYTEYGPGFTILKTSLNADAITIVSPSCTI
LGGKNMNVDIGTIKRADLKGVGTWAGGTPFDIKLECSGGVSVSGYANINTSFSGTLATNT
SANQGVLLNEKTGNSAAKGVGVQVIKDNTPLEFNKKHNIGTLQSQETRYITLPLHARFYQ
YAPTTSTGEVESHLVFNLTYD
>P18103
MKKTLLAIILGGMAFATTNASANTGTINFNGKITSATCTIDPEVNGNRTSTIDLGQAAIS

GHGTVVDFKLKPAPGSNDCLAKTNARIDWSGSMNSLGFNNTASGNTAAKGYHMTLRATNV
GNGSGGANINTSFTTAEYTHTSAIQSFNYSAQLKKDDRAPSNGGYKAGVFTTSASFLVTY
M
>Q52473
MVAFAGLTSKLTNLGNSAVGGVGGALQGVNTVASNATLQKNILLGTGDSLSVDAQAKASK
ESDANGAKLIAMQAQETMKKQTMDVLNAIQAGKEDSTNKKISATATNAKGISY
>Q47953
MRSKTITFPVLKLTGQSQALTNDMHKNADHTVPGLTVATGHLIAEALQMRLQGLNELALI
LKHAHWNVVGPQFIAVHEMLDSQVDEVRDFIDEIAERMATLGVAPNGLSGNLVETRQSPE
YPLGRATAQDHLKLIDLYYSHNIEAHRVVLEHNGHLDPISEDLLVAQTRSLEKLQWFIRA
HLDNGNGNI
>P12061
MRKSASAVAVLALIACGSAHAAGFVGNKAVVQAAVTIAAQNTTSANWSQDPGFTGPAVAA
GQKVGTLSITATGPHNSVSIAGKGASVSGGVATVPFVDGQGQPVFRGRIQGANINDQANT
GIDGLAGWRVASSQETLNVPVTTFGKSTLPAGTFTATFYVQQYQN
>Q93U24
MKLLKVAAIAAIVFSGSALAGVVPQYGGGGGNHGGGGNNSGPNSELNIYQYGGGNSALAL
QADARNSDLTITQHGGGNGADVGQGSDDSSIDLTQRGFGNSATLDQWNGKDSHMTVKQFG
GGNGAAVDQTASNSTVNVTQVGFGNNATAHQY
>Q03846
MKKTLLGSLILLAFAGNVQAAANADTKGTVTFFGKVVENTCQVKTDHKNRSVVLNDVGKN
SLKDKGNTAMPTPFTITLQNCNLTAANSSTNKANKVGLYFYSWENADKENNFTLKNKTST
SNDFATMVNIQLMESDGTKEIKVVGKETEDFVHKNATGAGVALTQTHPDNDHISGSTQLT
GVTGDLPLHFIAQYYSLGSTTAGKVQSSVDFQIAYE
>P33782
MKITHHYKSLLSAIISVALFYSAAPHADILDGGEIQFNGFVTDDAPKWTWQISSPDQTWA
VDTADARTENGQLVFDLSDKGPLPFLEGYLYEVAERGGPGFTPFITFSSNGRPFAVKEGS
DTSVQRFRASVPVRDPETGNVSGQLSFTLNQGMAVSTGKQEEGASTPSGMSLVSGQSVTD
VQSGSLPQGLKNRLSALLLMNKGFGNGMSAVDNGQVITQGVLADGRVMNLAAAYASAVSD
FELRLPAEGTPARWQAGLNVTVTVQ
>P08190
MKWCKRGYVLAAILALASATIQAADVTITVNGKVVAKPCTVSTTNATVDLGDLYSFSLMS
AGAASAWHDVALELTNCPVGTSRVTASFSGAADSTGYYKNQGTAQNIQLELQDDSGNTLN
TGATKTVQVDDSSQSAHFPLQVRALTVNGGATQGTIQAVISITYTYS
>P33784
MLNIIHRLKSGMFPALFFLTSASVLAHPLTIPPGHWLEGMAVGVTELSGTLYVRDVSWQW
QPRAVRMSSPDAVQAGLAAGKGGMVSESRRGQDFYILGGHTTSLTTARSGLQPSVTLLQV
APSSPRIAARGELARGQVRYGEITFTLRHLLAWQDNITGGQGWSVVSGEVTPEAEKQVKR
QLWQVNGYEWTPDYAGLTARPDAFISGAESLLSQENGSQHIAGAWVTSLSDVRVNFPGAE
EPVKRWQGNLTPVVVYF
>P25447
MKKTMMAAALVLSALSIQSALAAEYSEKTQYLGVVNGQVVGNSVVKVTRTPTDPVLYRSG
SNSPLPAELIIRHAESRPASGGLANITVKEALPDNGEARITLKTSLMVDGKRVALSARQQ
GEDVVITVPEAQQQIELRTDAPAELEVPVSYRGNLQIALQVED

>P04738
MKKAFLLACVFFLTGGGVSHAAVQKTIFSADVVASVCHVVVDADSTGNSGRLTFGTYRKS
TGASVPPRDFTVRLYESGATVQGCSAFLAGQVATLDFGNPGQLDAAGVVTRGAGDGIRVD
VRAVDAQADYRGRLTQDNHSVKYPVDFAAKGQFRFRAQPVFPADVKAGEYSGALTFVVTY
Q
>P20861
MKKLYKAITVICILMSNLQSAQGATKSVQVPIRTEVKIPTCQLEIDSSIDFSFVKIEDII
SSRATSKEANLNFRCDAHVDNVRIMFVPGSNRTSSDKRVMHSGTTGLGYSLQWSRASSGY
SDIGFNTQYQWSDSDAYQNLLSGKLRLKPVSFPGESLSKEGKVSSTINIEVTYD
>P15488
MLKIKYLLIGLSLSAMSSYSLAAAGPTLTKELALNVLSPAALDATWAPQDNLTLSNTGVS
NTLVGVLTLSNTSIDTVSIASTNVSDTSKNGTVTFAHETNNSASFATTISTDNANITLDK
NAGNTIVKTTNGSQLPTNLPLKFITTEGNEHLVSGNYRANITITSTIK
>P19369
MPNFFRNGCIALVGSVAAMGAAHAEGGIAEAAGKALDSAQSDVTITAPKVMMVVATVVGV
GILINMMRKA
>P12267
MKKVLLSAAMATAFFGMAAANAADTNVGGGQVNFFGKVTDVSCTVSVNGQGSDANVYLSP
VTLTEVKAAAADTYLKPKSFTIDVSDCQAADGTKQDDVSKLGVNWTGGNLLAGATAKQQG
YLANTEAAGAQNIQLVLSTDNATALTNKIIPGDSTQPKAAGDASAVQDGARFTYYVGYAT
STPTTVTTGVVNSYATYEITYQ
>P53522
MILNKKNIHSKSVMLFCAGIVSLMPLHAIAYLQGEVRTNGGPNIFYAVLDHTTFPNNKAG
ELATVNFSLPDRYDGTVYCPNSRIYDRALTYFKATTDLPPVGNNFYQLNEYVDIKINFEI
WGPNPLPTVPFSDIPNNRNNQQGCRVPSSPKPHISSGSSGQLTFRLRKPIINGVSLNGQS
LAQMYAMVSHSGAPKTYGSEPISKLVITSGIITTKDKCIFNNGSPITFDFGNVGNTSDYL
NGQNYKITRNIPIKCEGGSFTDPNSRIMFKVQTGSSGIASFDSNYLGTTGSVDRSNLGIV
LRDKSGTIIPPNQYFSVGKLNNFNGNWEVSAAPIAKAGSKITEGEFSAHATLIAEFM
>P05788
MQIPFQRALRLCLRAALAAIASAAHADDGTIVITGTITDTTCVIEDPSGPNHTKVVQLPK
ISKNALKANGDQAGRTPFIIKLKDCPSSLGNGVKAYFEPGPTTDYSTGDLRAYKMVYATN
PQTQLSNITAATEAQGVQVRISNLNDSKITMGANEATQQAAGFDPEVQTGGTSRTVTMRY
LASYVKKNGDVEASAITTYVGFSVVYP
>P33406
MNMKKFVKKPLAIAVLMLASGGMVNMVHAEPTVINSKDISATKTVKEGGSFSVEFKATEN
EIVSGKLDADTPAFHLVMSDSGEHKGWNVRPTGASEGGQMVSADGTRVDLHTNELSWDND
HWWIDDGSERVEATFFLAAGDEVKAGEYQFTGRVEEYVE

(6) $\mathbb{S}_6$: 12 flagellum proteins

>P96786
MAIGSLSSLGLGSKVLNYDVIDKLKDADEKALIAPLDKKMEQNVEKQKALVEIKTLLSAL
KGPVKTLSDYSTYISRKSNVTGDALSASVGVGVPIQDIKVDVQNLAQGDINELGAKFSSR
DDIFSQVDTTLKFYTQNKDYAVNIKAGMTLGDVAQSITDATNGEVMGIVMKTGGNDPYQL
MVNTKNTGEDNRVYFGSHLQSTLTNKNALSLGVDGSGKSEVSLNLKGADGNMHEVPIMLE

LPESASIKQKNTAIQKAMEQALENDPNFKNLIANGDISIDTLHGGESLIINDRRGGNIEV
KGSKAKELGFLQTTTQESDLLKSSRTIKEGKLEGVVSLNGQKLDLSALTKESNTSEENTD
AIIQAINAKEGLSAFKNAEGKLVINSKTGMLTIKGEDALGKASLKDLGLNAGMVQSYEAS
QNTLFMSKNLQKASDSAFTYNGVSITRPTNEVNDVISGVNITLEQTTEPNKPAIISVSRD
NQAIIDSLTEFVKAYNELIPKLDEDTRYDADTKIAGIFNGVGDIRAIRSSLNNVFSYSVH
TDNGVESLMKYGLSLDDKGVMSLDEAKLSSALNSNPKATQDFFYGSDSKDMGGREIHQEG
IFSKFNQVIANLIDGGNAKLKIYEDSLDRDAKSLTKDKENAQELLKTRYNIMAERFAAYD
SQISKANQKFNSVQMMIDQAAAKKN
>Q9PHW6
MAFGSLSSLGFGSGVLTQDTIDKLKEAEQKARIDPYTKKIEENTTKQKDLTEIKTKLLSF
QTAVSSLADATVFAKRKVVGSISDNPPASLTVNSGVALQSMNINVTQLAQKDVYQSKGLA
NDGGFVNAQLNGTADLTFFSNGKEYTVTVDKNTTYRDLADKINEASGGEIVAKIVNTGEK
GTPYRLTLTSKETGEDSAISFYAGKKDSNGKYQKDINAEKIFDDLGWGLDVSASIDPDKD
KKGYGIKDASLHIQTAQNAEFTLDGIKMFRSSNTVTDLGVGMTLTLNKTGEINFDVQQDF
EGVTKAMQDLVDAYNDLVTNLNAATDYNSETGTKGTLQGISEVNSIRSSILADLFDSQVV
DGTTEDANGNKVNTKVMLSMQDFGLSLNDAGTLSFDSSKFEQKVKEDPDSTESFFSNITK
YEDINHTGEVIKTGSLSKYLNSNGGNTNGLEFKPGDFTIVFNNQTYDLSKNSDGTNFKLT
GKTEEELLQNLANHINSKGIEGLKVKVESYNQNNVTGFRLNFSGDGSSDFSIKGDANILK
ELGLSDVNITSKPIEGKGIFSKLKATLQEMTGKDGSITKYDESLTNDIKSLNTSKDSTQA
MIDTRYDTMANQWLQYESILNKLNQQLNTVTNMINAANNSNN
>Q03475
MSSIDPATFAAQFAQIEIQPFKQRYQLQTNTYQSQLSALGKVESAMREFRTALNEMNSST
NSIIKNSTSISQEGYFTANADAKALSGSYQIFVEQVATSHQVSTGMPADLDATTEIPKTG
NLEFTINGKTMTIDLSTVDTDGDGVTTVSDLTKAINNNSDNPGVNATLVRSNGQTHFMLS
STETGVANQINVSATGTGQAWFEDAFTNLSQISAPQDAVIWLGAEKTGLKLTNSSNTFEG
VIDGVDITVTKAQTSGETAIGLGIGADDEATKEQLNKFVDAYNTLISTIDEHTQIGSEDK
KRGVLASDPTMRSIESQLSSLVRGEHGGMRLSEIGVTLDRHGKLKVDQEKFAEAQKNNSA
GLEAMFNGDGALLDSMDAMAEPFLKFSSGAFKSRKEALQANLDRLSDKQTTLERKYDMSY
KRYLKQFTQMNTLMTQMNQTMSMFG
>P58330
MTSILTNVAAMAALQTLRGIDSNMEETQARVSSGLRVGTASDNAAYWSIATTMRSDNMAL
SAVQDALGLGAAKVDTAYAGMENAVEVVKEIRAKLVAATEDGVDKAKIQEEIEQLKQQLT
SIATAASFSGENWLQADITTPVTKSVVGSFVRDSSGVVSVKTIDYVLDGNSVLFDTVGDA
GILDKIYNVSQASVTLPVNVNGTTTEYTVAAYAVDELIAAGATFDGDSANVTGYTVPAGG
IDYNGNFVKVEGTWVRAIDVAATGQEVVYDDGTTKWGVDTTVAGAPAINVVAPASIENID
ITNAAQAANLDALIRGVDEALEDLISATSALGSISMRIGMQEEFVSKLTDSIDSGIGRLV
DADMNEESTRLKALQTQQQLAIQSLSIANTNSENILQLFRQ
>P22251
MGFRINTNVAALNAKANSDLNAKSLDASLSRLSSGLRINSAADDASGMAIADSLRSQANT
LGQAISNGNDALGILQTADKAMDEQLKILDTIKTKATQAAQDGQSLKTRTMLQADINKLM
EELDNIANTTSFNGKQLLSGNFTNQEFQIGASSNQTVKATIGATQSSKIGVTRFETGAQS
FTSGVVGLTIKNYNGIEDFKFDNVVISTSVGTGLGALAEEINKSADKTGVRATYDVKTTG
VYAIKEGTTSQDFAINGVTIGKIEYKDGDGNGSLISAINAVKDTTGVQASKDENGKLVLT
SADGRGIKITGDIGVGSGILANQKENYGRLSLVKNDGRDINISGTNLSAIGMGTTDMISQ

SSVSLRESKGQISATNADAMGFNSYKGGGKFVFTQNVSSISAFMSAQGSGFSRGSGFSVG
SGKNLSVGLSQGIQIISSAASMSNTYVVSAGSGFSSGSGNSQFAALKTTAANTTDETAGV
TTLKGAMAVMDIAETAITNLDQIRADIGSIQNQVTSTINNITVTQVNVKAAESQIRDVDF
ASESANYSKANILAQSGSYAMAQANSSQQNVLRLLQ
>Q56827
MASISSLGAGSGMDLGSLLDKLQAAEKKRLEPLAQQQTSYKAKLTGFGTLKGSLEKLKSA
SEELKKFDKLNTTKTNGDHKTFTPSTDSKASPGNYEIEVQQLAKAQSLQSTEVSGVKKLL
GEQGKGTRTIIITQPGEKEPMKISLKDDETSLVEIRDAINKKEGNVNASIIKADENGTEE
EGKSYLILTSKKAGTRSIMTIKVEGDDELGKLLNYTSDGKGGGSGAMTQKVGAANAKLTV
NGIPIERQTNEIKDAPEGIILNLKKVSETEEVIVKVNGEDKKIPRPKTEILVVSRDIEPM
KEAIKKWVDSYNELQTTFDSLAKFKPVGKGEAASKDNGALLGDGTLKGIQSQLRHQLFAA
QDVADIATLNKLGIKQKLDGTLEISDEKLEKNLKEKSADVKAFFMGDGAKPGSTQTYNLL
KETLDGHEGTIATATEGINKRLKTLERQVEQTNRNIDATMERYKRQFTELDKLVNSLNNT
SSSLFQLLR
>Q56705
MSLGPVGMSGGMDINSMVSKIVDAERVPKQQRIDNDRTTINASISAYGRLRESLDTMKNL
MANFRQEKAFAVRTVETTDDNIVSATATTDAIAGKYAIDVLQLAQSHKVASDVLPEDAKF
GPGKLQISLGDDRFNIEVRSRSKLIDVVRGINGAKDNPGVRASVINDVEGPRLILASNLS
GKDHQIKVSVEAERGNPLKYFEYQTLEDRVNALEEARAAAEEVLGPLQAPQQPDQPEILD
ENGNPLPPEAQKAADNAQDDAQDDASQEPISAAGAEAAKAGQEAIDKANQRSSLRPEERI
PGWTETASGTLLDSYEEPELELDEKAIEKAPDVPGWNNAASGTLTDSYVTTKEAKQLLEQ
EKAEIEQKIADEKQELDAKVERGELSEEQAKQIHRAKLDPQERERLEKIDEAEAKIAKAQ
SSFEEYLGMTEVQAGQDSEVLLDGVAKLSSHNNVIEDAIEGVDLTLKGKSEPNKPPAEIG
VEYDRQSVRSDIENFVSAYNSFYQTSQALSSVDPTTGQKGPLAGDSTVRSADSRLKAVFS
SRIDQAPENLKSLTEFGITTTRQGTLEINYDMLDRQLNNNFNELEKFFGGNTGFAKRIED
AIHGITGITGSIRTREKSLTEQNYRLNDDQAALDRRMEGLEKRTHAKFTAMQDATGKMQG
QLGALMSALG
>P96747
MMISDATMMQQNYYLNNAQKASDKALENIAAVRAISGVDSANLAIADSLRSQSSTIDQGV
ANAYDAIGVLQIADASLTNISQSADRLNELSVKMNNAALNDSQKGMLRTEATRIQESIND
SFNNATYNGKNVFQTMNFVVGSGTETTNLNPLATDGLSIDSQDSITNFMDQLGSLRSEIG
SGINAITSNINASVQNSINSKAAENNLLNNDMAKNVNDFNANYLKENAAAFVAAQSNMQL
QSKIANLLQ
>Q08860
MAQVINTNSLSLITQNNINKNQSALSSSIERLSSGLRINSAKDDAAGQAIANRFTSNIKG
LTQAARNANDGISVAQTTEGALSEINNNLQRIRELTVQASTGTNSDSDLDSIQDEIKSRL
DEIDRVSGQTQFNGVNVLAKDGSMKIQVGANDGQTITIDLKKIDSDTLGLNGFNVNGGGA
VANTAASKADLVAANATVVGNKYTVSAGYDAAKASDLLAGVSDGDTVQATINNGFGTAAS
ATNYKYDSASKSYSFDTTTASAADVQKYLTPGVGDTAKGTITIDGSAQDVQISSDGKITA
SNGDKLYIDTTGRLTKNGSGASLTEASLSTLAANNTKATTIDIGGTSISFTGNSTTPDTI
TYSVTGAKVDQAAFDKAVSTSGNNVDFTTAGYSVNGTTGAVTKGVDSVYVDNNEALTTSD
TVDFYLQDDGSVTNGSGKAVYKDADGKLTTDAETKAATTADPLKALDEAISSIDKFRSSL
GAVQNRLDSAVTNLNNTTTNLSEAQSRIQDADYATEVSNMSKAQIIQQAGNSVLAKANQV
PQQVLSLLQG

>P02969

MALSVNTNQPALIALQNLNRTNDDMQAVQTRINTGEAISTAKDTAAVWSHRPGAGDMSGL

AREDEPGSGDIDRGRGPRAGESVSDLLKLMREKVVAAKDTSLTTTSRQALNADFQGLIKN

LNQVLRSATFDGANLLDGSQAADMSFLADADAGQAITLTLQNLSLGGTINTLTATDDILD

PVNAAGVLTRLDATLSAVNQAVGNIGTQAKQIDAHNTFVAKLNDVLETGVGNLVDADLAK

ESARLQALQVKQPLGAQALSIANGAPQIILSLFKGG

>P16326

MRISTQMMYEQNMSGITNSQAEWMKLGEQMSTGKRVTNPSDDPIAASQAVVLSQAQAQNS

QYALARTFATQKVSLEESVLSQVTTAIQTAQEKIVYAGNGTLSDDDRASLATDLQGIRDQ

LMNLANSTDGNGRYIFAGYKTEAAPFDQATGGYHGGEKSVTQQVDSARTMVIGHTGAQIF

NSITSNAVPEPDGSDSEKNLFVMLDTAIAALKTPVEGNNVEKEKAAAAIDKTNRGLKNSL

NNVLTVRAELGTQLSELSTLDSLGSDRALGQKLQMSNLVDVDWNSVISSYVMQQAALQAS

YKTFTDMQGMSLFQLNR

>Q9R9R6

MAITSAGAGSGIDLESVISASVSAKKAQLQQPIITKQNSTQITLSGIGQLKSSISAFTDI

LDKLSAPGAFNKRAINITQSKDDPILKVEGKSGASNGQYNIIVNKLAETSRQEGIFDSST

TPLATQDGQLTFKAGDKTFKVDVKAGDTLQDIRKSINSNGDNFGLSVNIVNTADGKAKLV

IDSGISGDGKDLTITGDNAELGVFEAGGGVMSQTRAASSAEINVDGNVLKSDTNTFDDSI

QDLKVTVLRVSDKDSAGDLKANKVDITTDKTSIQELVQQFVDGYNTLQDKMNSLGKRNTF

VGGVKQDDGGALAGDSTTRAIESFMSNLLVSPSQNSGTYSTVFEIGIKMDNKGKLSLDKT

KFGEAVDKNFDQVVALFGGEKGLASTLNSGLKEYTKSGGMLAQREDVLNSDLRALTQKTA

TANAQLTKYEAALRAQYGSLDALLVKMNSSASALATLQTSYQKS

(7) $\mathbb{S}_7$: 8 nucleoid proteins

>P0ABT2

MSTAKLVKSKATNLLYTRNDVSDSEKKATVELLNRQVIQFIDLSLITKQAHWNMRGANFI

AVHEMLDGFRTALIDHLDTMAERAVQLGGVALGTTQVINSKTPLKSYPLDIHNVQDHLKE

LADRYAIVANDVRKAIGEAKDDDTADILTAASRDLDKFLWFIESNIE

>P33920

MSLDINQIALHQLIKRDEQNLELVLRDSLLEPTETVVEMVAELHRVYSAKNKAYGLFSEE

SELAQTLRLQRQGEEDFLAFSRAATGRLRDELAKYPFADGGFVLFCHYRYLAVEYLLVAV

LSNLSSMRVNENLDINPTHYLDINHADIVARIDLTEWETNPESTRYLTFLKGRVGRKVAD

FFMDFLGASEGLNAKAQNRGLLQAVDDFTAEAQLDKAERQNVRQQVYSYCNEQLQAGEEI

ELKSLSKELAGVSEVSFTEFAAEKGYELEESFPADRSTLRQLTKFAGSGGGLTINFDAML

LGERIFWDPATDTLTIKGTPPNLRDQLQRRTSGGN

>P22523

MIERGKFRSLTLINWNGFFARTFDLDELVTTLSGGNGAGKSTTMAAFVTALIPDLTLLHF

RNTTEAGATSGSRDKGLHGKLKAGVCYSMLDTINSRHQRVVVGVRLQQVAGRDRKVDIKP

FAIQGLPMSVQPTQLVTETLNERQARVLPLNELKDKLEAMEGVQFKQFNSITDYHSLMFD

LGIIARRLRSASDRSKFYRLIEASLYGGISSAITRSLRDYLLPENSGVRKAFQDMEAALR

ENRMTLEAIRVTQSDRDLFKHLISEATNYVAADYMRHANERRVHLDKALEFRRELHTSRQ

QLAAEQYKHVDMARELAEHNGAEGDLEADYQAASDHLNLVQTALRQQEKIERYEADLDEL

QIRLEEQNEVVAEAIERQQENEARAEAAELEVDELKSQLADYQQALDVQQTRAIQYNQAI

AALNRAKELCHLPDLTADCAAEWLETFQAKELEATEKMLSLEQKMSMAQTAHSQFEQAYQ

LVVAINGPLARNEAWDVARELLREGVDQRHLAEQVQPLRMRLSELEQRLREQQEAERLLA
DFCKRQGKNFDIDELEALHQELEARIASLSDSVSNAREERMALRQEQEQLQSRIQSLMQR
APVWLAAQNSLNQLSEQCGEEFTSSQDVTEYLQQLLEREREAIVERDEVGARKNAVDEEI
ERLSQPGGSEDQRLNALAERFGGVLLSEIYDDVSLEDAPYFSALYGPSRHAIVVPDLSQV
TEHLEGLTDCPEDLYLIEGDPQSFDDSVFSVDELEKAVVVKIADRQWRYSRFPEVPLFGR
AARESRIESLHAEREVLSERFATLSFDVQKTQRLHQAFSRFIGSHLAVAFESDPEAEIRQ
LNSRRVELERALSNHENDNQQQRIQFEQAKEGVTALNRILPRLNLLADDSLADRVDEIRE
RLDEAQEAARFVQQFGNQLAKLEPIVSVLQSDPEQFEQLKEDYAYSQQMQRDARQQAFAL
TEVVQRRAHFSYSDSAEMLSGNSDLNEKLRERLEQAEAERTRAREALRGHAAQLSQYNQV
LASLKSSYDTKKELLNDLQRELQDIGVRADSGAEERARIRRDELHAQLSNNRSRRNQLEK
ALTFCEAEMDNLTRKLRKLERDYFEMREQVVTAKAGWCAVMRMVKDNGVERRLHRRELAY
LSADDLRSMSDKALGALRLAVADNEHLRDVLRMSEDPKRPERKIQFFVAVYQHLRERIRQ
DIIRTDDPVEAIEQMEIELSRLTEELTSREQKLAISSRSVANIIRKTIQREQNRIRMLNQ
GLQNVSFGQVNSVRLNVNVRETHAMLLDVLSEQHEQHQDLFNSNRLTFSEALAKLYQRLN
PQIDMGQRTPQTIGEELLDYRNYLEMEVEVNRGSDGWLRAESGALSTGEAIGTGMSILVM
VVQSWEDESRRLRGKDISPCRLLFLDEAARLDARSIATLFELCERLQMQLIIAAPENISP
EKGTTYKLVRKVFQNTEHVHVVGLRGFAPQLPETLPGTDEAPSQAS
>P36767
MLWFKNLMVYRLSREISLRAEEMEKQLASMAFTPCGSQDMAKMGWVPPMGSHSDALTHVA
NGQIVICARKEEKILPSPVIKQALEAKIAKLEAEQARKLKKTEKDSLKDEVLHSLLPRAF
SRFSQTMMWIDTVNGLIMVDCASAKKAEDTLALLRKSLGSLPVVPLSMENPIELTLTEWV
RSGSAAQGFQLLDEAELKSLLEDGGVIRAKKQDLTSEEITNHIEAGKVVTKLALDWQQRI
QFVMCDDGSLKRLKFCDELRDQNEDIDREDFAQRFDADFILMTGELAALIQNLIEGLGGE
AQR
>P60293
MSEFSQTVPELVAWARKNDFSISLPVDRLSFLLAVATLNGERLDGEMSEGELVDAFRHVS
DAFEQTSETIGVRANNAINDMVRQRLLNRFTSEQAEGNAIYRLTPLGIGITDYYIRQREF
STLRLSMQLSIVAGELKRAADAAEEGGDEFHWHRNVYAPLKYSVAEIFDSIDLTQRLMDE
QQQQVKDDIAQLLNKDWRAAISSCELLLSETSGTLRELQDTLEAAGDKLQANLLRIQDAT
MTHDDLHFVDRLVFDLQSKLDRIISWGQQSIDLWIGYDRHVHKFIRTAIDMDKNRVFAQR
LRQSVQTYFDEPWALTYANADRLLDMRDEEMALRDEEVTGELPEDLEYEEFNEIREQLAA
IIEEQLAVYKTRQVPLDLGLVVREYLSQYPRARHFDVARIVIDQAVRLGVAQADFTGLPA
KWQPINDYGAKVQAHVIDKY
>P36659
MELKDYYAIMGVKPTDDLKTIKTAYRRLARKYHPDVSKEPDAEARFKEVAEAWEVLSDEQ
RRAEYDQMWQHRNDPQFNRQFHHGDGQSFNAEDFDDIFSSIFGQHARQSRQRPATRGHDI
EIEVAVFLEETLTEHKRTISYNLPVYNAFGMIEQEIPKTLNVKIPAGVGNGQRIRLKGQG
TPGENGGPNGDLWLVIHIAPHPLFDIVGQDLEIVVPVSPWEAALGAKVTVPTLKESILLT
IPPGSQAGQRLRVKGKGLVSKKQTGDLYAVLKIVMPPKPDENTAALWQQLADAQSSFDPR
KDWGKA
>P0C093
MAEKQTAKRNRREEILQSLALMLESSDGSQRITTAKLAASVGVSEAALYRHFPSKTRMFD
SLIEFIEDSLITRINLILKDEKDTTARLRLIVLLLLGFGERNPGLTRILTGHALMFEQDR
LQGRINQLFERIEAQLRQVLREKRMREGEGYTTDETLLASQILAFCEGMLSRFVRSEFKY

RPTDDFDARWPLIAAQLQ
>P22524
MPVKLAQALANPLFPALDSALRSGRHIGLDELDNHAFLMDFQEYLEEFYARYNVELIRAP
EGFFYLRPRSTTLIPRSVLSELDMMVGKILCYLYLSPERLANEGIFTQQELYDELLTLAD
EAKLLKLVNNRSTGSDVDRQKLQEKVRSSLNRLRRLGMVWFMGHDSSKFRITESVFRFGA
DVRAGDDPREAQRRLIRDGEAMPIENHLQLNDETEENQPDSGEEE

(8) $\mathbb{S}_8$: 180 periplasm proteins

>P25718
MKLAACFLTLLPGFAVAASWTSPGFPAFSEQGTGTFVSHAQLPKGTRPLTLNFDQQCWQP
ADAIKLNQMLSLQPCSNTPPQWRLFRDGEYTLQIDTRSGTPTLMISIQNAAEPVASLVRE
CPKWDGLPLTVDVSATFPEGAAVRDYYSQQIAIVKNGQIMLQPAATSNGLLLLERAETDT
SAPFDWHNATVYFVLTDRFENGDPSNDQSYGRHKDGMAEIGTFHGGDLRGLTNKLDYLQQ
LGVNALWISAPFEQIHGWVGGGTKGDFPHYAYHGYYTQDWTNLDANMGNEADLRTLVDSA
HQRGIRILFDVVMNHTGYATLADMQEYQFGALYLSGDEVKKSLGERWSDWKPAAGQTWHS
FNDYINFSDKTGWDKWWGKNWIRTDIGDYDNPGFDDLTMSLAFLPDIKTESTTASGLPVF
YKNKMDTHAKAIDGYTPRDYLTHWLSQWVRDYGIDGFRVDTAKHVELPAWQQLKTEASAA
LREWKKANPDKALDDKPFWMTGEAWGHGVMQSDYYRHGFDAMINFDYQEQAAKAVDCLAQ
MDTTWQQMAEKLQGFNVLSYLSSHDTRLFREGGDKAAELLLLAPGAVQIFYGDESSRPFG
PTGSDPLQGTRSDMNWQDVSGKSAASVAHWQKISQFRARHPAIGAGKQTTLLLKQGYGFV
REHGDDKVLVVWAGQQ
>P39185
MKISRRDFIKQTAITATASVAGVTLPAGAANFVTDSEVTKLKWSKAPCRFCGTGCGVTVA
VKDNKVVATQGDPQAEVNKGLNCVKGYFLSKIMYGQDRLTRPLMRMKNGKYDKNGDFAPV
TWDQAFDEMERQFKRVLKEKGPTAVGMFGSGQWTVWEGYAAAKLYKAGFRSNNIDPNARH
CMASAAAGFMRTFGMDEPMGCYDDFEAADAFVLWGSNMAEMHPILWTRVTDRRLSHPKTR
VVVLSTFTHRCFDLADIGIIFKPQTDLAMLNYIANYIIRNNKVNKDFVNKHTVFKEGVTD
IGYGLRPDHPLQKAAKNASDPGAAKVITFDEFAKFVSKYDADYVSKLSAVPKAKLDQLAE
LYADPNIKVMSLWTMGFNQHTRGTWANNMVYNLHLLTGKIATPGNSPFSLTGQPSACGTA
REVGTFSHRLPADMVVTNPKHREEAERIWKLPPGTIPDKPGYDAVLQNRMLKDGKLNAYW
VQVNNNMQAAANLMEEGLPGYRNPANFIVVSDAYPTVTALAADLVLPSAMWVEKEGAYGN
AERRTQFWHQLVDAPGEARSDLWQLVEFAKRFKVEEVWPPELIAKKPEYKGKTLYDVLYR
NGQVDKFPLKDVNAEYHNAEAKAFGFYLQKGLFEEYATFGRGHGHDLAPFDAYHEARGLR
WPVVNGKETRWRYREGSDPYVKAGTGFQFYGNPDGKAVIFALPYEPPAESPDKEYPYWLV
TGRVLEHWHSGSMTRRVPELYRSFPNAVVFMHPEDAKALGLRRGVEVEVVSRRGRMRSRI
ETRGRDAPPRGLVFVPWFDASQLINKVTLDATCPISLQTDFKKCAVKIVKV
>P77214
MKKALQVAMFSLFTVIGFNAQANEHHHETMSEAQPQVISATGVVKGIDLESKKITIHHDP
IAAVNWPEMTMRFTITPQTKMSEIKTGDKVAFNFVQQGNLSLLQDIKVSQ
>P53052
MFKRPLTLSLLASLIALTTSTAQAATVDLRVLETTDLHSNMMDFDYYKDKPTEKFGLVRT
ASLIEAARQQATNSVLVDNGDLIQGSPLGDYMAAKGLKAGEIHPVYKAMNTLDYAVGNIG
NHEFNYGLDYLKKSLAGAKFPYVNANVIDVKTGKPLFQPYLIVDTPVKDRDGKNHNLRIG
YIGFGPPQVMIWDKANLTGKVTVDDITETAKKWVPEMRKQGANLVVAIPHSGLSSDPYKT

MAENSVYYLSQVPGIDAIMFGHAHAVFPSKDFATIKGADIAQGTLNGIPAVMPGQWGDHL
GVVDFVLNNDQGQWQVTQAKAEARPIFDKATQKSLAAENANLMKVLAADHQGTRDFVSQP
IGTASDNMYSYLSLIQDDPTVQIVNNAQRAYTEHFIQGDPDLADLPVLSAAAPFKAGGRK
NDPASFVEVEKGELTFRNAADLYLYPNTLVVVKASGADVKQWLECSAAQFNQIDVNSSKP
QSLINWDSFRTYNFDVIDGVNYEIDVSQPARYDGECALINDKAERIKNLTFNGKPIDPQA
TFLIGTNNYRAYSGKFAGTGDSHIAFASPDENRAVLSAYISAETKKHGQVTPQADNNWRL
ATLNSQQPLDIRFETSPSTKAAEFIKQKAQYPMKAMGTDEIGFAVFKIDLQK
>P35483
MTPGYPLALSLAVSMAVLGSALPAQARQDDPSLFNRQARGELSEYGGARRVEQDLTQALK
QSLSKKKAKNVILLIGDGMGDSEITVARNYARGAGGYFKGIDALPLTGQYTHYSLHKDSG
LPDYVTDSAASATAWSTGVKSYNGAIGVDIHEQPHRNLLELAKLNGKATGNVSTAELQDA
TPAALLAHVTARKCYGPEATSKQCPSNALENGGAGSITEQWLKTRPDVVLGGGAATFAET
AKAGRYAGKTLRAQAEARGYRIVENLDELKAVRRANQKQPLIGLFAPGNMPVRWLGPTAT
YHGNLNQPAVSCEANPKRTADIPTLAQMTSKAIELLKDNPNGFFLQVEGASIDKQDHAAN
PCGQIGETVDLDEAVQKALAFAKADGETLVIVTADHAHSSQIIPPETAAPGLTQLLTTKD
GAPLAISYGNSEESSQEHTGTQLRIAAYGPQAANVTGLTDQTDLFFTIRRALNLRD
>P0A862
MSQTVHFQGNPVTVANSIPQAGSKAQTFTLVAKDLSDVTLGQFAGKRKVLNIFPSIDTGV
CAASVRKFNQLATEIDNTVVLCISADLPFAQSRFCGAEGLNNVITLSTFRNAEFLQAYGV
AIADGPLKGLAARAVVVIDENDNVIFSQLVDEITTEPDYEAALAVLKA
>P11278
MKKRALLLSMSVLAMLYIPAGQAAEIDRLTVVKQYVDNVLNKASDTYHGDKPSPLLADGV
DPRTGQQMEWIFPDGRRAVLSNFSAQQNLMRVMSGLSELSGDPQYQKRAEDIVRYHFQNY
QDNSGLLYWGGHRFVDLKTLQPEGPSEKEKVHELKNAYPYYDLMFSVDSDATTRFIRGFW
NAHVYDWRILETSRHGEYGKPMGALWESTFEQQPPFFATKGLSFLNAGNDLIYSASLLYK
YQQDQGALVWAKRLADQYVLPRDAKTGLGVYQFTQALKREEPTDDADTHSKFGDRAQRQF
GPEFGPTALEGNMMLKGRTSTLYSENALMQLQLGKDLGGQGDDLLKWTVDGLKAFAKYGY
NEQDNTFRPMIANGQDLSNYTLPRDGYYGKKGSVLKPYKAGNEFLISYARAYAVDNDPLL
WKVARGIASDQGLGDIGSAPGKEMKVKLDTTNSDPYALFALLDLYNASQVAEYRSLAEKV
ADNIIKTRYIDGFFMASPDRQYADVDAIEPYALLALEASLRNKPQAVAPFLNGAGFTEGA
YLMADGSARISTRDNELFLLNVGETLQPNGRK
>P31133
MTALNKKWLSGLVAGALMAVSVGTLAAEQKTLHIYNWSDYIAPDTVANFEKETGIKVVYD
VFDSNEVLEGKLMAGSTGFDLVVPSASFLERQLTAGVFQPLDKSKLPEWKNLDPELLKLV
AKHDPDNKFAMPYMWATTGIGYNVDKVKAVLGENAPVDSWDLILKPENLEKLKSCGVSFL
DAPEEVFATVLNYLGKDPNSTKADDYTGPATDLLLKLRPNIRYFHSSQYINDLANGDICV
AIGWAGDVWQASNRAKEAKNGVNVSFSIPKEGAMAFFDVFAMPADAKNKDEAYQFLNYLL
RPDVVAHISDHVFYANANKAATPLVSAEVRENPGIYPPADVRAKLFTLKVQDPKIDRVRT
RAWTKVKSGK
>P72181
MRQRTPFARPGLLASAALALVLGPLAASAQEQVAPPKDPAAALEDHKTRTDNRYEPSLDN
LAQQDVAAPGAPEGVSALSDAQYNEANKIYFERCAGCHGVLRKGATGKALTPDLTRDLGF
DYLQSFITYGSPAGMPNWGTSGELSAEQVDLMANYLLLDPAAPPEFGMKEMRESWKVHVA
PEDRPTQQENDWDLENLFSVTLRDAGQIALIDGATYEIKSVLDTGYAVHISRLSASGRYL

FVIGRDGKVNMIDLWMKEPTTVAEIKIGSEARSIETSKMEGWEDKYAIAGAYWPPQYVIM
DGETLEPKKIQSTRGMTYDEQEYHPEPRVAAILASHYRPEFIVNVKETGKILLVDYTDLD
NLKTTEISAERFLHDGGLDGSHRYFITAANARNKLVVIDTKEGKLVAIEDTGGQTPHPGR
GANFVHPTFGPVWATSHMGDDSVALIGTDPEGHPDNAWKILDSFPALGGGSLFIKTHPNS
QYLYVDATLNPEAEISGSVAVFDIKAMTGDGSDPEFKTLPIAEWAGITEGQPRVVQGEFN
KDGTEVWFSVWNGKDQESALVVVDDKTLELKHVIKDERLVTPTGKFNVYNTMTDTY
>P05465
MNQPTSRSGLTTFTVIIIGLLALFLLIGGIWLATLGGSIYYIIAGVLLLIVAWQLYKRAS
TALWFYAALMLGTIIWSVWEVGTDFWALAPRLDILGILGLWLLVPAVTRGINNLGSSKVA
LSSTLAIAIVLMVYSIFNDPQEINGEIKTPQPETAQAVPGVAESDWPAYGRTQAGVRYSP
LKQINDQNVKDLKVAWTLRTGDLKTDNDSGETTNQVTPIKIGNNMFICTAHQQLIAIDPA
TGKEKWRFDPKLKTDKSFQHLTCRGVMYYDANNTTEFATSLQSKKSSSTQCPRKVFVPVN
DGRLVAVNADTGKACTDFGQNGQVNLQEFMPYAYPGGYNPTSPGIVTGSTVVIAGSVTDN
YSNKEPSGVIRGYDVNTGKLLWVFDTGAADPNAMPGEGTTFVHNSPNAWAPLAYDAKLDI
VYVPTGVGTPDIWGGDRTELKERYANSMLAINASTGKLVWNFQTTHHDLWDMDVPSQPSL
ADIKNKAGQTVPAIYVLTKTGNAFVLDRRNGQPIVPVTEKPVPQTVKRGPQTKGEFYSKT
QPFSDLNLAPQDKLTDKDMWGATMLDQLMCRVSFKRLNYDGIYTPPSENGTLVFPGNLGV
FEWGGMSVNPDRQVAVMNPIGLPFVSRLIPADPNRAQTAKGAGTEQGVQPMYGVPYGVEI
SAFLSPLGLPCKQPAWGYVAGVDLKTHEVVWKKRIGTIRDSLPNLFQLPAVKIGVPGLGG
SISTAGNVMFVGATQDNYLRAFNVTNGKKLWEARLPAGGQATPMTYEINGKQYVVIMAGG
HGSFGTKMGDYLVAYALPDNK
>Q03315
MTATLRAFGWLAAFALTVTFAQGAAAEEQQKGKVGAKPVETGVVIRGVTLAGPVGNPGTS
TGKTCDFSGEPVDPSGRLEGASVNCRPNGNQANTTPGLPARFNAYCMINAPVKSARLIQA
ARPENANHCDLSGITPKDATGQFGGAVWR
>P72298
MRLKSIMCAALFVVAGQAAAQEKSITIATEGAYAPWNFSGPNGKLDGFEIDLAKVLCERM
KVKCQIVAQNWDGIIPSLVAKKYDVIMAAMSVTPKRQEVISFSTPYGAHMNGFAVMKDSK
LADMPGSGEVYSLNTQADAAKKRIDDVNAFLDGTTVGVQGSTTGSQFLENYFKNSVDIKE
YKTVEEFNIDLMSGRVDAVFASATVLTAAFEQPDMKDAKVVGPLFSGDELGKVAVGLRKD
DAALKAEFRLGAVGLRKEDAALKADFDSSIKAVADEGTIKTLSSKWFKVDVTPH
>O68897
MKSALKTFVPGALALLLLFPVAAQAKEVETKTKLANVVILATGGTIAGAGASAANSATYQ
AAKVGIEQLIAGVPELSQIANVRGEQVMQIASESINNENLLQLGRRVAELADSKDVDGIV
ITHGTDTLEETAYFLNLVEKTDKPIIVVGSMRPGTAMSADGMLNLYNAVAVAGSKDARGK
GVLVTMNDEIQSGRDVSKMINIKTEAFKSPWGPLGMVVEGKSYWFRLPAKRHTMDSEFDI
KTIKSLPDVEIAYGYGNVSDTAVKALAQAGAKAIIHAGTGNGSVSSKVVPALQELRKQGV
QIIRSSHVNAGGFVLRNAEQPDDKYDWVVAHDLNPQKARILAMVALTKTQDSKELQRMFW
EY
>Q57142
MKIMIACGLVAAALFTLTSGQSLAADAPFEGRKKCSSCHKAQAQSWKDTAHAKAMESLKP
NVKKEAKQKAKLDPAKDYTQDKDCVGCHVDGFGQKGGYTIESPKPMLTGVGCESCHGPGR
NFRGDHRKSGQAFEKSGKKTPRKDLAKKGQDFHFEERCSACHLNYEGSPWKGAKAPYTPF
TPEVDAKYTFKFDEMVKEVKAMHEHYKLEGVFEGEPKFKFHDEFQASAKPAKKGK

>P21852
MSGCRAQNAPGGIPVTPKSSYSGPIVVDPVTRIEGHLRIEVEVENGKVKNAYSSSTLFRG
LEIILKGRDPRDAQHFTQRTCGVCTYTHALASTRCVDNAVGVHIPKNATYIRNLVLGAQY
LHDHIVHFYHLHALDFVDVTAALKADPAKAAKVASSISPRKTTAADLKAVQDKLKTFVET
GQLGPFTNAYFLGGHPAYYLDPETNLIATAHYLEALRLQVKAARAMAVFGAKNPHTQFTV
VGGVTCYDALTPQRIAEFEALWKETKAFVDEVYIPDLLVVAAAYKDWTQYGGTDNFITFG
EFPKDEYDLNSRFFKPGVVFKRDFKNIKPFDKMQIEEHVRHSWYEGAEARHPWKGQTQPK
YTDLHGDDRYSWMKAPRYMGEPMETGPLAQVLIAYSQGHPKVKAVTDAVLAKLGVGPEAL
FSTLGRTAARGIETAVIAEYVGVMLQEYKDNIAKGDNVICAPWEMPKQAEGVGFVNAPRG
GLSHWIRIEDGKIGNFQLVVPSTWTLGPRCDKNNVSPVEASLIGTPVADAKRPVEILRTV
HSFDPCIACGVHVIDGHTNEVHKFRIL
>P18278
MTRPASAKRRSLLGILAAGTICAAALPYAAVPARADGQGNTGEAIIHADDHPENWLSYGR
TYSEQRYSPLDQINRSNVGDLKLLGYYTLDTNRGQEATPLVVDGIMYATTNWSKMEALDA
ATGKLLWQYDPKVPGNIADKGCCDTVNRGAGYWNGKVFWGTFDGRLVAADAKTGKKVWAV
NTIPADASLGKQRSYTVDGAVRVAKGLVLIGNGGAEFGARGFVSAFDAETGKLKWRFYTV
PNNKNEPDHAASDNILMNKAYKTWGPKGAWVRQGGGGTVWDSLVYDPVSDLIYLAVGNGS
PWNYKYRSEGIGSNLFLGSIVALKPETGEYVWHFQATPMDQWDYTSVQQIMTLDMPVKGE
MRHVIVHAPKNGFFYVLDAKTGEFLSGKNYVYQNWANGLDPLTGRPMYNPDGLYTLNGKF
WYGIPGPLGAHNFMAMAYSPKTHLVYIPAHQIPFGYKNQVGGFKPHADSWNVGLDMTKNG
LPDTPEARTAYIKDLHGWLLAWDPVKMETVWKIDHKGPWNGGILATGGDLLFQGLANGEF
HAYDATNGSDLYKFDAQSGIIAPPMTYSVNGKQYVAVEVGWGGIYPISMGGVGRTSGWTV
NHSYIAAFSLDGKAKLPALNNRGFLPVKPPAQYDQKVVDNGYFQYQTYCQTCHGDNGEGA
GMLPDLRWAGAIRHQDAFYNVVGRGALTAYGMDRFDTSMTPDEIEAIRQYLIKRANDTYQ
REVDARKNDKNIPENPTLGINP
>P12376
MLLNRTSFVTLFAAGMLVSALAQAHPKLVSSTPAEGSEGAAPAKIELHFSENLVTQFSGA
KLVMTAMPGMEHSPMAVKAAVSGGGDPKTMVITPASPLTAGTYKVDWRAVSSDTHPITGS
VTFKVK
>P0AAA9
MKRNTKIALVMMALSAMAMGSTSAFAHGGHGMWQQNAAPLTSEQQTAWQKIHNDFYAQSS
ALQQQLVTKRYEYNALLAANPPDSSKINAVAKEMENLRQSLDELRVKRDIAMAEAGIPRG
AGMGMGYGGCGGGGHMGMGHW
>P39186
MKPSRSWASLLAVCAVLLAALAMQAIFFPAPARAQGLVDAMRGPTAIANEPRAPLLYPTE
NKDIRRTRNYTMQPPTIPHKIDGYQLDKDFNRCMFCHARTRTEETQAIPVSITHYMDRDN
NVLADVSPRRYFCTQCHVPQADTKPLIGNNFVDVDTILKRRPGAKGAAK
>P05458
MPRSTWFKALLLLVALWAPLSQAETGWQPIQETIRKSDKDNRQYQAIRLDNGMVVLLVSD
PQAVKSLSALVVPVGSLEDPEAYQGLAHYLEHMSLMGSKKYPQADSLAEYLKMHGGSHNA
STAPYRTAFYLEVENDALPGAVDRLADAIAEPLLDKKYAERERNAVNAELTMARTRDGMR
MAQVSAETINPAHPGSKFSGGNLETLSDKPGNPVQQALKDFHEKYYSANLMKAVIYSNKP
LPELAKMAADTFGRVPNKESKKPEITVPVVTDAQKGIIIHYVPALPRKVLRVEFRIDNNS
AKFRSKTDELITYLIGNRSPGTLSDWLQKQGLVEGISANSDPIVNGNSGVLAISASLTDK

GLANRDQVVAAIFSYLNLLREKGIDKQYFDELANVLDIDFRYPSITRDMDYVEWLADTMI
RVPVEHTLDAVNIADRYDAKAVKERLAMMTPQNARIWYISPKEPHNKTAYFVDAPYQVDK
ISAQTFADWQKKAADIALSLPELNPYIPDDFSLIKSEKKYDHPELIVDESNLRVVYAPSR
YFASEPKADVSLILRNPKAMDSARNQVMFALNDYLAGLALDQLSNQASVGGISFSTNANN
GLMVNANGYTQRLPQLFQALLEGYFSYTATEDQLEQAKSWYNQMMDSAEKGKAFEQAIMP
AQMLSQVPYFSRDERRKILPSITLKEVLAYRDALKSGARPEFMVIGNMTEAQATTLARDV
QKQLGADGSEWCRNKDVVVDKKQSVIFEKAGNSTDSALAAVFVPTGYDEYTSSAYSSLLG
QIVQPWFYNQLRTEEQLGYAVFAFPMSVGRQWGMGFLLQSNDKQPSFLWERYKAFFPTAE
AKLRAMKPDEFAQIQQAVITQMLQAPQTLGEEASKLSKDFDRGNMRFDSRDKIVAQIKLL
TPQKLADFFHQAVVEPQGMAILSQISGSQNGKAEYVHPEGWKVWENVSALQQTMPLMSEK
NE
>Q01269
MPKSFRHLVQALACLALLASASLQAQESRLDRILESGVLRVATTGDYKPFSYRTEEGGYA
GFDVDMAQRLAESLGAKLVVVPTSWPNLMRDFADDRFDIAMSGISINLERQRQAYFSIPY
LRDGKTPITLCSEEARFQTLEQIDQPGVTAIVNPGGTNEKFARANLKKARILVHPDNVTI
FQQIVDGKADLMMTDAIEARLQSRLHPELCAVHPQQPFDFAEKAYLLPRDEAFKRYVDQW
LHIAEQSGLLRQRMEHWLEYRWPTAHGK
>P04032
MKRVLLLSSLCAALSFGLAVSGVAADGAALYKSCIGCHGADGSKAAMGSAKPVKGQGAEE
LYKKMKGYADGSYGGERKAMMTNAVKKYSDEELKALADYMSKL
>P02924
MHKFTKALAAIGLAAVMSQSAMAENLKLGFLVKQPEEPWFQTEWKFADKAGKDLGFEVIK
IAVPDGEKTLNAIDSLAASGAKGFVICTPDPKLGSAIVAKARGYDMKVIAVDDQFVNAKG
KPMDTVPLVMMAATKIGERQGQELYKEMQKRGWDVKESAVMAITANELDTARRRTTGSMD
ALKAAGFPEKQIYQVPTKSNDIPGAFDAANSMLVQHPEVKHWLIVGMNDSTVLGGVRATE
GQGFKAADIIGIGINGVDAVSELSKAQATGFYGSLLPSPDVHGYKSSEMLYNWVAKDVEP
PKFTEVTDVVLITRDNFKEELEKKGLGGK
>Q06530
MTLNRRDFIKTSGAAVAAVGILGFPHLAFGAGRKVVVVGGGTGGATAAKYIKLADPSIEV
TLIEPNTDYYTCYLSNEVIGGDRKLESIKHGYDGLRAHGIQVVHDSATGIDPDKKLVKTA
GGAEFGYDRCVVAPGIELIYDKIEGYSEEAAAKLPHAWKAGEQTAILRKQLEDMADGGTV
VIAPPAAPFRCPPGPYERASQVAYYLKAHKPKSKVIILDSSQTFSKQSQFSKGWERLYGF
GTENAMIEWHPGPDSAVVKVDGGEMMVETAFGDEFKADVINLIPPQRAGKIAQIAGLTND
AGWCPVDIKTFESSIHKGIHVIGDACIANPMPKSGYSANSQGKVAAAAVVALLKGEEPGT
PSYLNTCYSILAPAYGISVAAIYRPNADGSAIESVPDSGGVTPVDAPDWVLEREVQYAYS
WYNNIVHDTFG
>P42790
MKSSAAKQTVLCLNRYAVVALPLAIASFAAFGASPASTLWAPTDTKAFVTPAQVEARSAA
PLLELAAGETAHIVVSLKLRDEAQLKQLAQAVNQPGNAQFGKFLKRRQFLSQFAPTEAQV
QAVVAHLRKNGFVNIHVVPNRLLISADGSAGAVKAAFNTPLVRYQLNGKAGYANTAPAQV
PQDLGEIVGSVLGLQNVTRAHPMLKVGERSAAKTLAAGTAKGHNPTEFPTIYDASSAPTA
ANTTVGIITIGGVSQTLQDLQQFTSANGLASVNTQTIQTGSSNGDYSDDQQGQGEWDLDS
QSIVGSAGGAVQQLLFYMADQSASGNTGLTQAFNQAVSDNVAKVINVSLGWCEADANADG
TLQAEDRIFATAAAQGQTFSVSSGDEGVYECNNRGYPDGSTYSVSWPASSPNVIAVGGTT

LYTTSAGAYSNETVWNEGLDSNGKLWATGGGYSVYESKPSWQSVVSGTPGRRLLPDISFD
AAQGTGALIYNYGQLQQIGGTSLASPIFVGLWARLQSANSNSLGFPAASFYSAISSTPSL
VHDVKSGNNGYGGYGYNAGTGWDYPTGWGSLDIAKLSAYIRSNGFGH
>P37735
MLTRRILGALVGATALSLALSVPALAEPIVIKFSHVVAPDTPKGKGAAKFEELAEKYTNG
AVDVEVYPNSQLYKDKEELEALQLGAVQMLAPSLAKFGPLGVQDFEVFDLPYIFKDYEAL
HKVTQGEAGKMLLSKLEAKGITGLAFWDNGFKIMSANTPLTMPDDFLGLKMRIQSSKVLE
AEMNALGAVPQVMAFSEVYQALQTGVVDGTENPPSNMFTQKMNEVQKHATVSNHGYLGYA
VIVNKQFWDGLPADVRTGLEKAMAESTDYANGIAKEENEKALQAMKDAGTTEFHELTAEE
RAAWEEVLTPVHDEMAERIGAETIAAVKAATAE
>P06875
MKNRNRMIVNCVTASLMYYWSLPALAEQSSSEIKIVRDEYGMPHIYANDTWHLFYGYGYV
VAQDRLFQMEMARRSTQGTVAEVLGKDFVKFDKDIRRNYWPDAIRAQIAALSPEDMSILQ
GYADGMNAWIDKVNTNPETLLPKQFNTFGFTPKRWEPFDVAMIFVGTMANRFSDSTSEID
NLALLTALKDKYGVSQGMAVFNQLKWLVNPSAPTTIAVQESNYPLKFNQQNSQTAALLPR
YDLPAPMLDRPAKGADGALLALTAGKNRETIAAQFAQGGANGLAGYPTTSNMWVIGKSKA
QDAKAIMVNGPQFGWYAPAYTYGIGLHGAGYDVTGNTPFAYPGLVFGHNGVISWGSTAGF
GDDVDIFAERLSAEKPGYYLHNGKWVKMLSREETITVKNGQAETFTVWRTVHGNILQTDQ
TTQTAYAKSRAWDGKEVASLLAWTHQMKAKNWQEWTQQAAKQALTINWYYADVNGNIGYV
HTGAYPDRQSGHDPRLPVPGTGKWDWKGLLPFEMNPKVYNPQSGYIANWNNSPQKDYPAS
DLFAFLWGGADRVTEIDRLLEQKPRLTADQAWDVIRQTSRQDLNLRLFLPTLQAATSGLT
QSDPRRQLVETLTRWDGINLLNDDGKTWQQPGSAILNVWLTSMLKRTVVAAVPMPFDKWY
SASGYETTQDGPTGSLNISVGAKILYEAVQGDKSPIPQAVDLFAGKPQQEVVLAALEDTW
ETLSKRYGNNVSNWKTPAMALTFRANNFFGVPQAAAEETRHQAEYQNRGTENDMIVFSPT
TSDRPVLAWDVVAPGQSGFIAPDGTVDKHYEDQLKMYENFGRKSLWLTKQDVEAHKESQE
VLHVQR
>P31545
MQYKDENGVNEPSRRRLLKVIGALALAGSCPVAHAQKTQSAPGTLSPDARNEKQPFYGEH
QAGILTPQQAAMMLVAFDVLASDKADLERLFRLLTQRFAFLTQGGAAPETPNPRLPPLDS
GILGGYIAPDNLTITLSVGHSLFDERFGLAPQMPKKLQKMTRFPNDSLDAALCHGDVLLQ
ICANTQDTVIHALRDIIKHTPDLLSVRWKREGFISDHAARSKGKETPINLLGFKDGTANP
DSQNDKLMQKVVWVTADQQEPAWTIGGSYQAVRLIQFRVEFWDRTPLKEQQTIFGRDKQT
GAPLGMQHEHDVPDYASDPEGKVIALDSHIRLANPRTAESESSLMLRRGYSYSLGVTNSG
QLDMGLLFVCYQHDLEKGFLTVQKRLNGEALEEYVKPIGGGYFFALPGVKDANDYFGSAL
LRV
>P37734
MSFSLSRLVVALGAGLLACAAQAAEVQVAVAANFTAPMKDIASQFEKDTGHKVITSFGPT
GGFYSQIQNGAPFEVFLAADDTTPEKLEKEGGTVAGSRFTYAVGKLVLWSAKPGYVDDQG
AVLKKNAFKHLSIANPKTAPYGAAAVQVLAKLGLTEATKSKLVEGASIAQAHQFVATGNA
ELGFVALSQVYKDGKLTGGSGWNVPGDLYEPIRQDAVILTKGKDNPAAQALVDYLKGPKA
TEVIKAYGYGLQ
>P03841
MKMNKSLIVLCLSAGLLASAPGISLADVNYVPQNTSDAPAIPSAALQQLTWTPVDQSKTQ
TTQLATGGQQLNVPGISGPVAAYSVPANIGELTLTLTSEVNKQTSVFAPNVLILDQNMTP

SAFFPSSYFTYQEPGVMSADRLEGVMRLTPALGQQKLYVLVFTTEKDLQQTTQLLDPAKA
YAKGVGNSIPDIPDPVARHTTDGLLKLKVKTNSSSSVLVGPLFGSSAPAPVTVGNTAAPA
VAAPAPAPVKKSEPMLNDTESYFNTAIKNAVAKGDVDKALKLLDEAERLGSTSARSTFIS
SVKGKG
>P37902
MQLRKPATAILALALSAGLAQADDAAPAAGSTLDKIAKNGVIVVGHRESSVPFSYYDNQQ
KVVGYSQDYSNAIVEAVKKKLNKPDLQVKLIPITSQNRIPLLQNGTFDFECGSTTNNVER
QKQAAFSDTIFVVGTRLLTKKGGDIKDFANLKDKAVVVTSGTTSEVLLNKLNEEQKMNMR
IISAKDHGDSFRTLESGRAVAFMMDDALLAGERAKAKKPDNWEIVGKPQSQEAYGCMLRK
DDPQFKKLMDDTIAQVQTSGEAEKWFDKWFKNPIPPKNLNMNFELSDEMKALFKEPNDKA
LN
>P10488
MYDRWFSQQELQVLPFADEDEQRNQTWLELVGEAQQLMANAARQMSRGRLRWQPAGWSSW
SRIHRHAEFLTRLNEMHAAEPQMREQTGVTPEMIDFITRAFAESKLAIWARYLNAEELAF
TRQHYFDRLMEWPALVADLHRACREKRDRPPRKVSSWRSAAWRCSSLTRVKMRRRSRISL
CHAQVPALMKGTWMTSEVLSWLQQAIGVMMRQAQGPRE
>Q8RNT4
MKRRSVLLSGVALSGTALANDSIFFSPLKYLGAEQQRSIDASRSLLDNLIPPSLPQYDNL
AGKLARRAVLTSKKLAYVWTENFANVKGVPMARSVPLGELPNVDWLLKTAGVIVELIVNS
FASLPASAAAQFERIPAGLNGDLEAARQVHEALLEEAKNDPAAAGSLLLRFTELQTRVIA
LLTRVGLLVDDILKSASNLVTQGGQGDGLNRFRAVFGTLRLPEVADSFRDDEAFAYWRVA
GPNPLLIRRVDALPANFPLGEEQFRRVMGADDSLLEAAASRRLYLLDYAELGKLAPSGAV
DKLLTGTGFAYAPIALFALGKDRARLLPVAIQCGQDPATHPMFVRPAESESDLYWGWQMA
KTVVLVAEENYHEMFVHLAQTHLVSEAFCLATQRTLAPSHPLHVLLAPHFEGTLFVNEGA
ARILLPSAGFIDVMFAAPIQDTQATAGGNRLGFDFYRGMLPESLKARNVDDPAALPDYPY
RDDGLLVWNAIRQWAADYVAVYYASDGDVTADVELAAWVGEVIGSGKVAGFRPITGRSQL
VEVLTMVIFTASAQHAAVNFPQPSMMTYAPAICAMSAAPAPDSPSGKSEADWLKMMPPAL
VALEKVNIYHLLGSVYHGRLGDYRQTGFPYAPVFSDRRVTASGGPLERFQARLKEVEATI
RTRNQARRKPYEYLLPSRIPASTNI
>P38554
ETFEIPESVTMSPKQFEGYTPKKGDVTFNHASHMDIACQQCHHTVPDTYTIESCMTEGCH
DNIKERTEISSVYRTFHTTKDSEKSCVGCHRELKRQGPSDAPLACNSCHVQ
>P23006
MASARESTPRYLTLIGATLACSALALGAAQAQTEPAEPEAPAETAAADAAGQTEGQRGAA
EAAAALAAGEADEPVILEAPAPDARRVYIQDPAHFAAITQQFVIDGSTGRILGMTDGGFL
PHPVAAEDGSFFAQASTVFERIARGKRTDYVEVFDPVTFLPIADIELPDAPRFLVGTYQW
MNALTPDNKNLLFYQFSPAPAVGVVDLEGKTFDRMLDVPDCYHIFPASPTVFYMNCRDGS
LARVDFADGETKVTNTEVFHTEDELLINHPAFSLRSGRLVWPTYTGKIFQADLTAEGATF
RAPIEALTEAERADDWRPGGWQQTAYHRQSDRIYLLVDQRDEWKHKAASRFVVVLNAETG
ERINKIELGHEIDSINVSQDAEPLLYALSAGTQTLHIYDAATGEELRSVDQLGRGPQIIT
THDMDS
>P00811
MFKTTLCALLITASCSTFAAPQQINDIVHRTITPLIEQQKIPGMAVAVIYQGKPYYFTWG
YADIAKKQPVTQQTLFELGSVSKTFTGVLGGDAIARGEIKLSDPTTKYWPELTAKQWNGI

TLLHLATYTAGGLPLQVPDEVKSSSDLLRFYQNWQPAWAPGTQRLYANSSIGLFGALAVK
PSGLSFEQAMQTRVFQPLKLNHTWINVPPAEEKNYAWGYREGKAVHVSPGALDAEAYGVK
STIEDMARWVQSNLKPLDINEKTLQQGIQLAQSRYWQTGDMYQGLGWEMLDWPVNPDSII
NGSDNKIALAARPVKAITPPTPAVRASWVHKTGATGGFGSYVAFIPEKELGIVMLANKNY
PNPARVDAAWQILNALQ
>Q8VUS8
MNALVGCTTSFDPGWEVDAFGAVSNLCQPMEADLYGCADPCWWPAQVADTLNTYPNWSAG
ADDVMQDWRKLQSVFPETKGSS
>Q52812
MKNKLLSAAIGAAVLAVGASAASATTLSDVKAKGFVQCGVNTGLTGFAAPDASGNWAGFD
VDFCKAVASAVFGDPTKVKYTPTNAKERFTALQSGEIDVLSRNTTWTINRDTALGFNFRP
VTYYDGQGFMVRKGLNVKSALELSGAAICVQSGTTTELNLADYFKTNNLQYNPVVFENLP
EVNAAYDAGRCDVYTTDQSGLYSLRLTLKNPDEHIILPEIISKEPLGPAVRQGDDQWFDI
VSWTAYALINAEEFGITQANVDEMKNSPNPDIKRFLGSETDTKIGTDLGLTNDWAANVIK
GVGNYGEIFERNIGQGSPLKIARGLNALWNKGGIQYAPPVR
>P0AFI5
MPKFRVSLFSLALMLAVPFAPQAVAKTAAATTASQPEIASGSAMIVDLNTNKVIYSNHPD
LVRPIASISKLMTAMVVLDARLPLDEKLKVDISQTPEMKGVYSRVRLNSEISRKDMLLLA
LMSSENRAAASLAHHYPGGYKAFIKAMNAKAKSLGMNNTRFVEPTGLSVHNVSTARDLTK
LLIASKQYPLIGQLSTTREDMATFSNPTYTLPFRNTNHLVYRDNWNIQLTKTGFTNAAGH
CLVMRTVINNKPVALVVMDAFGKYTHFADASRLRTWIETGKVMPVPAAALSYKKQKAAQM
AAAGQTAQND
>P18035
MSLTKSLLFTLLLSAAAVQASTRDEIERLWNPQGMATQPAQPAAGTSARTAKPAPRWFRL
SNGRQVNLADWKVVLFMQGHCPYCHQFDPVLKQLAQQYGFSVFSYTLDGQGDTAFPEALP
VPPDVMQTFFPNIPVATPTTFLVNVNTLEALPLLQGATDAAGFMARVDTVLQMYGGKKGA
K
>P22619
MLGNFRFDDMVEKLSRRVAGQTSRRSVIGKLGTAMLGIGLVPLLPVDRRGRVSRANAADA
PAGTDPRAKWVPQDNDIQACDYWRHCSIDGNICDCSGGSLTNCPPGTKLATASWVASCYN
PTDGQSYLIAYRDCCGYNVSGRCPCLNTEGELPVYRPEFANDIIWCFGAEDDAMTYHCTI
SPIVGKAS
>P40120
MDRRRFIKGSMAMAAVCGTSGIASLFSQAAFAADSDIADGQTQRFDFSILQSMAHDLAQT
AWRGAPRPLPDTLATMTPQAYNSIQYDAEKSLWHNVENRQLDAQFFHMGMGFRRRVRMFS
VDPATHLAREIHFRPELFKYNDAGVDTKQLEGQSDLGFAGFRVFKAPELARRDVVSFLGA
SYFRAVDDTYQYGLSARGLAIDTYTDSKEEFPDFTAFWFDTVKPGATTFTVYALLDSASI
TGAYKFTIHCEKSQVIMDVENHLYARKDIKQLGIAPMTSMFSCGTNERRMCDTIHPQIHD
SDRLSMWRGNGEWICRPLNNPQKLQFNAYTDNNPKGFGLLQLDRDFSHYQDIMGWYNKRP
SLWVEPRNKWGKGTIGLMEIPTTGETLDNIVCFWQPEKAVKAGDEFAFQYRLYWSAQPPV
HCPLARVMATRTGMGGFSEGWAPGEHYPEKWARRFAVDFVGGDLKAAAPKGIEPVITLSS
GEAKQIEILYIEPIDGYRIQFDWYPTSDSTDPVDMRMYLRCQGDAISETWLYQYFPPAPD
KRQYVDDRVMS
>Q59452

MKLTKVALFSLGLFGFSSMALAHGDHMHNHDTKMDTMSKDMMSMEKIVVPVQQLDPQNGN
KDVGTVEITESAYGLVFTPKLHDLAHGLHGFHIHEKPSCEPKEKDGKLVAGLGAGGHWDP
KQTQKHGYPWSDDAHMGDLPALFVMHDGSATTPVLAPRLKKLAEVKGHSLMIHAGGDNHS
DHPAPLGGGGPRMACGVIK
>Q06529
MTQSTPRLMLAASVLALGLASNAGAEPTAEMLTNNCAGCHGTHGNSVGPASPSIAQMDPM
VFVEVMEGFKSGEIASTIMGRIAKGYSTADFEKMAGYFKQQTYQPAKQSFDTALADTGAK
LHDKYCEKCHVEGGKPLADEEDYHILAGQWTPYLQYAMSDFREERRPMEKKMASKLRELL
KAEGDAGLDALFAFYASQQ
>Q05433
MSKRNAVTTFFTNRVTKALGMTLALMMTCQSAMASLAADQTRYIFRGDKDALTITVTNND
KERTFGGQAWVDNIVEKDTRPTFVVTPSFFKVKPNGQQTLRIIMASDHLPKDKESVYWLN
LQDIPPALEGSGIAVAVRTKLKLFYRPKALIEGRKGAEEGISLQSRPDGRTMLVNTTPYI
FAIGSLLDGNGKRIATDNETAQKLLMFMPGDEVQVKGNVVKVDSLNDYGELQTWTINQKK
TPTSSGQKASDSLVNPSDKADKK
>Q9X4Y1
MKTHRLNMTASLLIGISAFAVQAFASEPTVVPEQPPFPAQGKITYVSRDSILEFKALREY
REPEWVTEKFVKAGKLPPVAERLPKEPMVFKAGNMPDGMGVYGDVMRHVIGGRPEGWNYS
AGQTQGWGGIDIGMFECLTRTAPLFQVEADDMEPLPNLAKSWDWSEDGRKLTMHLIEGAK
WSDGDPFDADDVMFYWEDNVLDSSVSPLNGATPETFGEGTTLKKIDQYTVEWTFKEAFPR
QHLFAMAYGTFCPGPSHILKTKHPKYAGTTYNEYKNGFPAEYMNLPVMGAWVPVAYRPDD
IIVLRRNPYYWKVDEAGNQLPYLNELHYKLSTWADRDVQAIAGSGDISNLEQPENFVESL
KRAANESAPARLAFGPRVIGYNMHMNFSGNGWGDPDERAKAVRELNRNLDFRKAVTMAVD
RKKLGEALVKGPFTAIYPGGLSSGTSFYDRNSTIYYPHDLEGAKVLLEKVGLKDTDGNGF
VNFPAGKLGGRDVEIVLLVNSDYSTDRNLAEGMVGQMEKLGLRVVLNALDGKQRDAANYA
GRFDWMIHRNTAEFASVVQNTPQLAPTGPRTSWHHRAPEGGEVDVMPHEQELVDIVNKFI
ASNDNDERTELMKQYQKVATTNVDTVGLTEYPGALIINKRFSNIPPGAPIFMFNWAEDTI
IRERVFVAADKQGDYELYPEQLPGKPGESGPIN
>P61316
MKKIAITCALLSSLVASSVWADAASDLKSRLDKVSSFHASFTQKVTDGSGAAVQEGQGDL
WVKRPNLFNWHMTQPDESILVSDGKTLWFYNPFVEQATATWLKDATGNTPFMLIARNQSS
DWQQYNIKQNGDDFVLTPKASNGNLKQFTINVGRDGTIHQFSAVEQDDQRSSYQLKSQQN
GAVDAAKFTFTPPQGVTVDDQRK
>P18472
MRCRGLIALLIWGQSVAAADLGTWGDLWPVKEPDMLTVIMQRLTALEQSGEMGRKMDAFK
ERVIRNSLRPPAVPGIGRTEKYGSRLFDPSVRLAADIRDNEGRVFARQGEVMNPLQYVPF
NQTLYFINGDDPAQVAWMKRQTPPTLESKIILVQGSIPEMQKSLDSRVYFDQNGVLCQRL
GIDQVPARVSAVPGDRFLKVEFIPAEEGRK
>P21170
MSDDMSMGLPSSAGEHGVLRSMQEVAMSSQEASKMLRTYNIAWWGNNYYDVNELGHISVC
PDPDVPEARVDLAQLVKTREAQGQRLPALFCFPQILQHRLRSINAAFKRARESYGYNGDY
FLVYPIKVNQHRRVIESLIHSGEPLGLEAGSKAELMAVLAHAGMTRSVIVCNGYKDREYI
RLALIGEKMGHKVYLVIEKMSEIAIVLDEAERLNVVPRLGVRARLASQGSGKWQSSGGEK
SKFGLAATQVLQLVETLREAGRLDSLQLLHFHLGSQMANIRDIATGVRESARFYVELHKL

GVNIQCFDVGGGLGVDYEGTRSQSDCSVNYGLNEYANNIIWAIGDACEENGLPHPTVITE
SGRAVTAHHTVLVSNIIGVERNEYTVPTAPAEDAPRALQSMWETWQEMHEPGTRRSLREW
LHDSQMDLHDIHIGYSSGIFSLQERAWAEQLYLSMCHEVQKQLDPQNRAHRPIIDELQER
MADKMYVNFSLFQSMPDAWGIDQLFPVLPLEGLDQVPERRAVLLDITCDSDGAIDHYIDG
DGIATTMPMPEYDPENPPMLGFFMVGAYQEILGNMHNLFGDTEAVDVFVFPDGSVEVELS
DEGDTVADMLQYVQLDPKTLLTQFRDQVKKTDLDAELQQQFLEEFEAGLYGYTYLEDE
>Q50925
MRIGEWMRGLLLCAGLMMCGVVHADISTVPDETYDALKLDRGKATPKETYEALVKRYKDP
AHGAGKGTMGDYWEPIAISIYMDPNTFYKPPVSPKEVAERKDCVECHSDETPVWVRAWKR
STHANLDKIRNLKSDDPLYYKKGKLEEVENNLRSMGKLGEKETLKEVGCIDCHVDVNKKD
KADHTKDIRMPTADTCGTCHLREFAERESERDTMVWPNGQWPAGRPSHALDYTANIETTV
WAAMPQREVAEGCTMCHTNQNKCDNCHTRHEFSAAESRKPEACATCHSGVDHNNWEAYTM
SKHGKLAEMNRDKWNWEVRLKDAFSKGGQNAPTCAACHMEYEGEYTHNITRKTRWANYPF
VPGIAENITSDWSEARLDSWVLTCTQCHSERFARSYLDLMDKGTLEGLAKYQEANAIVHK
MYEDGTLTGQKTNRPNPPEPEKPGFGIFTQLFWSKGNNPASLELKVLEMAENNLAKMHVG
LAHVNPGGWTYTEGWGPMNRAYVEIQDEYTKMQELSALQARVNKLEGKQTSLLDLKGTGE
KISLGGLGGGMLLAGALALIGWRKRKQTRA
>P24930
MYTQNTMKKNWYVTVGAAAALAATVGMGTAMAGTLDTTWKEATLPQVKAMLEKDTGKVSG
DTVTYSGKTVHVVAAAVLPGFPFPSFEVHDKKNPTLEIPAGATVDVTFINTNKGFGHSFD
ITKKGPPYAVMPVIDPIVAGTGFSPVPKDGKFGYTDFTWHPTAGTYYYVCQIPGHAATGM
FGKIVVK
>P33363
MKWLCSVGIAVSLALQPALADDLFGNHPLTPEARDAFVTELLKKMTVDEKIGQLRLISVG
PDNPKEAIREMIKDGQVGAIFNTVTRQDIRAMQDQVMELSRLKIPLFFAYDVLHGQRTVF
PISLGLASSFNLDAVKTVGRVSAYEAADDGLNMTWAPMVDVSRDPRWGRASEGFGEDTYL
TSTMGKTMVEAMQGKSPADRYSVMTSVKHFAAYGAVEGGKEYNTVDMSPQRLFNDYMPPY
KAGLDAGSGAVMVALNSLNGTPATSDSWLLKDVLRDQWGFKGITVSDHGAIKELIKHGTA
ADPEDAVRVALKSGINMSMSDEYYSKYLPGLIKSGKVTMAELDDAARHVLNVKYDMGLFN
DPYSHLGPKESDPVDTNAESRLHRKEAREVARESLVLLKNRLETLPLKKSATIAVVGPLA
DSKRDVMGSWSAAGVADQSVTVLTGIKNAVGENGKVLYAKGANVTSDKGIIDFLNQYEEA
VKVDPRSPQEMIDEAVQTAKQSDVVVAVVGEAQGMAHEASSRTDITIPQSQRDLIAALKA
TGKPLVLVLMNGRPLALVKEDQQADAILETWFAGTEGGNAIADVLFGDYNPSGKLPMSFP
RSVGQIPVYYSHLNTGRPYNADKPNKYTSRYFDEANGALYPFGYGLSYTTFTVSDVKLSA
PTMKRDGKVTASVQVTNTGKREGATVVQMYLQDVTASMSRPVKQLKGFEKITLKPGETQT
VSFPIDIEALKFWNQQMKYDAEPGKFNVFIGTDSARVKKGEFELL
>P07024
MKLLQRGVALALLTTFTLASETALAYEQDKTYKITVLHTNDHHGHFWRNEYGEYGLAAQK
TLVDGIRKEVAAEGGSVLLLSGGDINTGVPESDLQDAEPDFRGMNLVGYDAMAIGNHEFD
NPLTVLRQQEKWAKFPLLSANIYQKSTGERLFKPWALFKRQDLKIAVIGLTTDDTAKIGN
PEYFTDIEFRKPADEAKLVIQELQQTEKPDIIIAATHMGHYDNGEHGSNAPGDVEMARAL
PAGSLAMIVGGHSQDPVCMAAENKKQVDYVPGTPCKPDQQNGIWIVQAHEWGKYVGRADF
EFRNGEMKMVNYQLIPVNLKKKVTWEDGKSERVLYTPEIAENQQMISLLSPFQNKGKAQL
EVKIGETNGRLEGDRDKVRFVQTNMGRLILAAQMDRTGADFAVMSGGGIRDSIEAGDISY

KNVLKVQPFGNVVVYADMTGKEVIDYLTAVAQMKPDSGAYPQFANVSFVAKDGKLNDLKI
KGEPVDPAKTYRMATLNFNATGGDGYPRLDNKPGYVNTGFIDAEVLKAYIQKSSPLDVSV
YEPKGEVSWQ
>Q01537
MSEQFRLTRRSMLAGAAVAGALAPVVTSVAHAEGGGIKTNSAATAANIATLERVKVELVK
PPFVHAHTQKAEGEPKVVEFKMTIQEKKIVVDDKGTEVHAMTFDGSVPGPMMIVHQDDYV
ELTLVNPDTNELQHNIDFHSATGALGGGALTVVNPGDTAVLRFKATKAGVFVYHCAPAGM
VPWHVTSGMNGAIMVLPRDGLKDHKGHELVYDKVYYVGEQDFYVPKDENGKFKKYESAGE
AYPDVLEAMKTLTPTHVVFNGAVGALTGDNALQAKVGDRVLILHSQANRDTRPHLIGGHG
DYVWATGKFANPPELDQETWFIPGGAAGAAYYTFQQPGIYAYVNHNLIEAFELGAAGHFK
VTGDWNDDLMTAVVSPTSG
>P07598
MSRTVMERIEYEMHTPDPKADPDKLHFVQIDEAKCIGCDTCSQYCPTAAIFGEMGEPHSI
PHIEACINCGQCLTHCPENAIYEAQSWVPEVEKKLKDGKVKCIAMPAPAVRYALGDAFGM
PVGSVTTGKMLAALQKLGFAHCWDTEFTADVTIWEEGSEFVERLTKKSDMPLPQFTSCCP
GWQKYAETYYPELLPHFSTCKSPIGMNGALAKTYGAERMKYDPKQVYTVSIMPCIAKKYE
GLRPELKSSGMRDIDATLTTRELAYMIKKAGIDFAKLPDGKRDSLMGESTGGATIFGVTG
GVMEAALRFAYEAVTGKKPDSWDFKAVRGLDGIKEATVNVGGTDVKVAVVHGAKRFKQVC
DDVKAGKSPYHFIEYMACPGGCVCGGGQPVMPGVLEAMDRTTTRLYAGLKKRLAMASANK
A
>P33407
MKYKFSHNFISYNLFLFVFMSLILLPYSHASSMGFNTSQHKFSVRTGETRIIYPLSSVKG
VSLSVTNPQDYPILVQTQVKGEDKHSPAPFMATPPLFRLDAGMRGRVRVTRTGGNFPEDR
ESLQWLCITGVPPKEGDVWDNSQHDKKNNMQDVNLNILLSVGTCMKLLVRPDQLRQKPEE
MAGKLIWHRNGQQLQVNNPTPFYMNFKSVSLGNKNIKLSSAGNENYVAPFAERSFSLPVD
MAERPAEINWQIINDLGSESQVFKANI
>P21338
MKAFWRNAALLAVSLLPFSSANALALQAKQYGDFDRYVLALSWQTGFCQSQHDRNRNERD
ECRLQTETTNKADFLTVHGLWPGLPKSVAARGVDERRWMRFGCATRPIPNLPEARASRMC
SSPETGLSLETAAKLSEVMPGAGGRSCLERYEYAKHGACFGFDPDAYFGTMVRLNQEIKE
SEAGKFLADNYGKTVSRRDFDAAFAKSWGKENVKAVKLTCQGNPAYLTEIQISIKADAIN
APLSANSFLPQPHPGNCGKTFVIDKAGY
>O08368
MSIRRLACSLLLSSLALPVLAAECPAMLQGELPKLRAKGENIELCQYAGKPLVVVNTASF
CGFTPQFKGLEALYQRYKDQELEVLGVPSDDFRQESADSKETATVCYVNYGVTFAMTEPQ
PVSGANAIPLFKGLAEQSRQPRWNFFKYVVDRQGKVVASFSSLTKPDDPELIAARREGDR
LATLIPSRFTALHPSGRWALSVCLPRLARRRTQFTRRALQEQ
>P14924
MIKVPRFICMIALTSGILASGLSQSVSAHTEKSEPSSTYHFHSDPLLYLAPPPTSGSPLQ
AHDDQTFNSTRQLKGSTRWALATQDADLHLASVLKDYACAAGMNLDIAQLPHLANLIKRA
LRTEYDDIGRAKNNWNRKRPFVDTDQPICTEKDREGLGKQGSYPSGHTTIGWSVALILAE
LIPDHAANILQRGQIFGTSRIVCGAHWFSDVQAGYIMASGEIAALHGDADFRRDMELARK
ELEKARTSAHTPDDLLCKIEQSAR
>P14171

MRFTATVLSRVATGLALGLSMATASLAETPVEALSETVARIEEQLGARVGLSLMETGTGW
SWSHREDELFLMNSTVKVPVCGAILARWDAGRLSLSDALPVRKADLVPYAPVTETRVGGN
MTLDELCLAAIDMSDNVAANILIGHLGGPEAVTQFFRSVGDPTSRLDRIEPKLNDFASGD
ERDTTSPAAMSETLRALLLGDVLSPEARGKLAEWMRHGGVTGALLRAEAEDAWLILDKSG
SGSHTRNLVAVIQPEGGAPWIATMFISDTDAEFEVRNEALKDLGRAVVAVVRE
>P39325
MWKRLLIVSAVSAAMSSMALAAPLTVGFSQVGSESGWRAAETNVAKSEAEKRGITLKIAD
GQQKQENQIKAVRSFVAQGVDAIFIAPVVATGWEPVLKEAKDAEIPVFLLDRSIDVKDKS
LYMTTVTADNILEGKLIGDWLVKEVNGKPCNVVELQGTVGASVAIDRKKGFAEAIKNAPN
IKIIRSQSGDFTRSKGKEVMESFIKAENNGKNICMVYAHNDDMVIGAIQAIKEAGLKPGK
DILTGSIDGVPDIYKAMMDGEANASVELTPNMAGPAFDALEKYKKDGTMPEKLTLTKSTL
YLPDTAKEELEKKKNMGY
>Q9HXB1
MNGVSRLLSLALLGAALHWAPAQAEEQPRLFELLGQPGYKATWHAMFKGESDVPKWVSDA
SGPSSPSTSLSLEGQPYVLANSCKPHDCGNNRLLVAFRGDKSAAYGLQVSLPDEPAEVMQ
TPSKYATYRWYGEPSRQVRELLMKQLESDPNWK
>Q3J164
MKFQVKALAAIAAFAALPALAQEGDPEAGAKAFNQCQTCHVIVDDSGTTIAGRNAKTGPN
LYGVVGRTAGTQADFKGYGEGMKEAGAKGLAWDEEHFVQYVQDPTKFLKEYTGDAKAKGK
MTFKLKKEADAHNIWAYLQQVAVRP
>P31810
MKKVLLALGLGVSTLMSVNSFAADLQEGKQYVQVSQQASQQKEVIEFFSFYCPHCYAFEM
EYKIPQQVVDALPKDVKFKQYHVNFLGHQSENLTRAWALAMALGAESKVKSPLFEAAQKD
ALKSMDDIRAIFLSNGITAEQFDGGINSFAVNGLVNKQVNAAEQFKVRGVPDFYVNGKFR
VNPEGLNYDDFVKDYVQTVKGLLQK
>P0ADA1
MMNFNNVFRWHLPFLFLVLLTFRAAAADTLLILGDSLSAGYRMSASAAWPALLNDKWQSK
TSVVNASISGDTSQQGLARLPALLKQHQPRWVLVELGGNDGLRGFQPQQTEQTLRQILQD
VKAANAEPLLMQIRLPANYGRRYNEAFSAIYPKLAKEFDVPLLPFFMEEVYLKPQWMQDD
GIHPNRDAQPFIADWMAKQLQPLVNHDS
>P51698
MSLGAKPFGEKKFIEIKGRRMAYIDEGTGDPILFQHGNPTSSYLWRNIMPHCAGLGRLIA
CDLIGMGDSDKLDPSGPERYAYAEHRDYLDALWEALDLGDRVVLVVHDWGSALGFDWARR
HRERVQGIAYMEAIAMPIEWADFPEQDRDLFQAFRSQAGEELVLQDNVFVEQVLPGLILR
PLSEAEMAAYREPFLAAGEARRPTLSWPRQIPIAGTPADVVAIARDYAGWLSESPIPKLF
INAEPGALTTGRMRDFCRTWPNQTEITVAGAHFIQEDSPDEIGAAIAAFVRRLRPA
>Q47084
MNEYLVSRRRLLRLSLSLLPLGLGRPALAQSLFMPQRVITLFQGATDTAVALGITPAGVV
DSWSEKPMYRYLRQALAGVPHVGLETQPSLEDIVLLKPDAIVASRFRHQRLEPLLSQIAP
VVMLDEIYQFKKTVQVMGQALQRQAVADQLLQNWQLRVNGLREQLQRKFGGDWPPTVSIL
DIREDHIRSYLPGSFPGSVLSELGFGWSDASRAQPGVSLKLTNKESIPVVDADIFFIFLR
SESPSVQRNYESLIRHPLWQQLRAPRRNQVWVVNGVTWSLSGGILGANMMLDDIARVTGI
AGGVS
>P0AEQ3

MKSVLKVSLAALTLAFAVSSHAADKKLVVATDTAFVPFEFKQGDKYVGFDVDLWAAIAKE
LKLDYELKPMDFSGIIPALQTKNVDLALAGITITDERKKAIDFSDGYYKSGLLVMVKANN
NDVKSVKDLDGKVVAVKSGTGSVDYAKANIKTKDLRQFPNIDNAYMELGTNRADAVLHDT
PNILYFIKTAGNGQFKAVGDSLEAQQYGIAFPKGSDELRDKVNGALKTLRENGTYNEIYK
KWFGTEPK
>P0AEX9
MKIKTGARILALSALTTMMFSASALAKIEEGKLVIWINGDKGYNGLAEVGKKFEKDTGIK
VTVEHPDKLEEKFPQVAATGDGPDIIFWAHDRFGGYAQSGLLAEITPDKAFQDKLYPFTW
DAVRYNGKLIAYPIAVEALSLIYNKDLLPNPPKTWEEIPALDKELKAKGKSALMFNLQEP
YFTWPLIAADGGYAFKYENGKYDIKDVGVDNAGAKAGLTFLVDLIKNKHMNADTDYSIAE
AAFNKGETAMTINGPWAWSNIDTSKVNYGVTVLPTFKGQPSKPFVGVLSAGINAASPNKE
LAKEFLENYLLTDEGLEAVNKDKPLGAVALKSYEEELAKDPRIAATMENAQKGEIMPNIP
QMSAFWYAVRTAVINAASGRQTVDEALKDAQTRITK
>Q52963
MLKRLCTILAASALAAPLALGIARANGTEELTPYKMIRSLQYVQDSVVLGDHSAIEMQRF
MLGAIDERLRAADHSAFRDPRNVDAALVYVMSGGNPATLDLLADRDIEGNFDSRVTDALR
QYLNGKGPLIVENLTKAAPEYKNSRIGPYLFLILGNAMSQQDPIEAMKHYDWARLTAPGT
IIEEAALRRSVSLAAQAGLPEKGFRYALNYARRYLTSPFASQFADVFVELAVAHFDEAAD
GRVSEILSFMDSARQREVYLRVARRAAIAGNQALARLASRRAEELAGDDSSRSQVLASFY
EGLAAVPSADVFSAAEALEAIPDEKLSPRDRALREAAKAVADAVVRPPLGESPAQAPAPI
AERPAGEQSELAAEESGSGMSPFGQPVEASPGRPSEMTAEADVAASDDPALDGFLASGRS
KIDEIDALLKGEGQ
>P29822
MDYSRLLKRSVSAALTAAALLCSTAAFAGEVTIWCWDPNFNVAIMKEAAERYTAKHPDTT
FNIVDFAKADVEQKLQTGLASGMTDTLPDIVLIEDYGAQKYLQSFPGSFAALTDKIDFSG
FAKYKVDLMTLEGQVYGVPFDSGVTGLYYRTDYLEQAGFKPEDMQNLTWDRFIEIGKEVK
AKTGHEMMALDANDGGLIRIMMQSGGQWYFNEDGSLNITGNAALKAALETQARIVNERVA
KPTSGSNDGIRALTSGDVASVLRGVWITGTVKSQPDQAGKWALTAIPKLNIEGATAASNL
GGSSWYVLEASAEKDEAIDFLNEIYAKDLDFYQKILTERGAVGSLLAARTGEAYQKPDDF
FGGQTVWQNFADWLVQVPAVNYGIFTNELDTAVTANFPALVKGTPVDEVLKAIEDQAAGQ
IQ
>P62395
MSGILTRWRQFGKRYFWPHLLLGMVAASLGLPALSNAAEPNAPAKATTRNHEPSAKVNFG
QLALLEANTRRPNSNYSVDYWHQHAIRTVIRHLSFAMAPQTLPVAEESLPLQAQHLALLD
TLSALLTQEGTPSEKGYRIDYAHFTPQAKFSTPVWISQAQGIRAGPQRLT
>P74917
MTTYLSQDRLRNKENDTMTYQHSKMYQSRTFLLFSALLLVAGQASAAVGSADAPAPYRVS
SDCMVCHGMTGRDTLYPIVPRLAGQHKSYMEAQLKAYKDHSRADQNGEIYMWPVAQALDS
AKITALADYFNAQKPPMQSSGIKHAGAKEGKAIFNQGVTNEQIPACMECHGSDGQGAGPF
PRLAGQRYGYIIQQLTYFHNGTRVNTLMNQIAKNITVAQMKDVAAYLSSL
>P31550
MLKKCLPLLLLCTAPVFAKPVLTVYTYDSFAADWGPGPVVKKAFEADCNCELKLVALEDG
VSLLNRLRMEGKNSKADVVLGLDNNLLDAASKTGLFAKSGVAADAVNVPGGWNNDTFVPF
DYGYFAFVYDKNKLKNPPQSLKELVESDQNWRVIYQDPRTSTPGLGLLLWMQKVYGDDAP

QAWQKLAKKTVTVTKGWSEAYGLFLKGESDLVLSYTTSPAYHILEEKKDNYAAANFSEGH
YLQVEVAARTAASKQPELAQKFLQFMVSPAFQNAIPTGNWMYPVANVTLPAGFEKLTKPA
TTLEFTPAEVAAQRQAWISEWQRAVSR
>Q9X759
MNKKAMAAAVSMILAGGAHAAQQERPNVIVIIADDMGYSDISPFGGEIPTPNLQAMAEQG
MRMSQYYTSPMSAPARSMLLTGNSNQQAGMGGMWWYDSTIGKEGYELRLTDRVTTMAERF
KDAGYNTLMAGKWHLGFVPGATPKERGFNHAFAFMGGGTSHFNDAIPLGTVEAFHTYYTR
DGERVSLPDDFYSSEAYARQMNSWIKATPKEQPVFAWLAFTAPHDPLQAPDEWIKRFKGQ
YEQGYAEVYRQRIARLKALGIIHDDTPLPHLELDKEWEALTPEQQKYTAKVMQVYAAMIA
NMDAQIGTLMETLKQTGRDKNTLLVFLTDNGANPAQGFYYESTPEFWKQFDNSYDNVGRK
GSFVSYGPHWANVSNAPYANYHKTTSAQGGINTDFMISGPGITRHGKIDASTMAVYDVAP
TLYEFAGIDPNKSLAKKPVLPMIGVSFKRYLTGEVQEPPRGNYGVELHHQAAWVDGEWKL
RRLVPRGLTAGDAPWQLFNLHDDPLETHDVAAEHPDRVKAMSEAYEAFAKRTMVTKAQGK
MIDYVGIDSKTGRYLAVDPATMKPVPAPQAIPVSEIH
>P77335
MTEIVADKTVEVVKNAIETADGALDLYNKYLDQVIPWQTFDETIKELSRFKQEYSQAASV
LVGDIKTLLMDSQDKYFEATQTVYEWCGVATQLLAAYILLFDEYNEKKASAQKDILIKVL
DDGITKLNEAQKSLLVSSQSFNNASGKLLALDSQLTNDFSEKSSYFQSQVDKIRKEAYAG
AAAGVVAGPFGLIISYSIAAGVVEGKLIPELKNKLKSVQNFFTTLSNTVKQANKDIDAAK
LKLTTEIAAIGEIKTETETTRFYVDYDDLMLSLLKEAAKKMINTCNEYQKRHGKKTLFEV
PEV
>P16700
MAVNLLKKNSLALVASLLLAGHVQATELLNSSYDVSRELFAALNPPFEQQWAKDNGGDKL
TIKQSHAGSSKQALAILQGLKADVVTYNQVTDVQILHDKGKLIPADWQSRLPNNSSPFYS
TMGFLVRKGNPKNIHDWNDLVRSDVKLIFPNPKTSGNARYTYLAAWGAADKADGGDKGKT
EQFMTQFLKNVEVFDTGGRGATTTFAERGLGDVLISFESEVNNIRKQYEAQGFEVVIPKT
NILAEFPVAWVDKNVQANGTEKAAKAYLNWLYSPQAQTIITDYYYRVNNPEVMDKLKDKF
PQTELFRVEDKFGSWPEVMKTHFTSGGELDKLLAAGRN
>P46883
MGSPSLYSARKTTLALAVALSFAWQAPVFAHGGEAHMVPMDKTLKEFGADVQWDDYAQLF
TLIKDGAYVKVKPGAQTAIVNGQPLALQVPVVMKDNKAWVSDTFINDVFQSGLDQTFQVE
KRPHPLNALTADEIKQAVEIVKASADFKPNTRFTEISLLPPDKEAVWAFALENKPVDQPR
KADVIMLDGKHIIEAVVDLQNNKLLSWQPIKDAHGMVLLDDFASVQNIINNSEEFAAAVK
KRGITDAKKVITTPLTVGYFDGKDGLKQDARLLKVISYLDVGDGNYWAHPIENLVAVVDL
EQKKIVKIEEGPVVPVPMTARPFDGRDRVAPAVKPMQIIEPEGKNYTITGDMIHWRNWDF
HLSMNSRVGPMISTVTYNDNGTKRKVMYEGSLGGMIVPYGDPDIGWYFKAYLDSGDYGMG
TLTSPIARGKDAPSNAVLLNETIADYTGVPMEIPRAIAVFERYAGPEYKHQEMGQPNVST
ERRELVVRWISTVGNYDYIFDWIFHENGTIGIDAGATGIEAVKGVKAKTMHDETAKDDTR
YGTLIDHNIVGTTHQHIYNFRLDLDVDGENNSLVAMDPVVKPNTAGGPRTSTMQVNQYNI
GNEQDAAQKFDPGTIRLLSNPNKENRMGNPVSYQIIPYAGGTHPVAKGAQFAPDEWIYHR
LSFMDKQLWVTRYHPGERFPEGKYPNRSTHDTGLGQYSKDNESLDNTDAVVWMTTGTTHV
ARAEEWPIMPTEWVHTLLKPWNFFDETPTLGALKKDK
>P24183
MDVSRRQFFKICAGGMAGTTVAALGFAPKQALAQARNYKLLRAKEIRNTCTYCSVGCGLL

MYSLGDGAKNAREAIYHIEGDPDHPVSRGALCPKGAGLLDYVNSENRLRYPEYRAPGSDK
WQRISWEEAFSRIAKLMKADRDANFIEKNEQGVTVNRWLSTGMLCASGASNETGMLTQKF
ARSLGMLAVDNQARVUHGPTVASLAPTFGRGAMTNHWVDIKNANVVMVMGGNAAEAHPVG
FRWAMEAKNNNDATLIVVDPRFTRTASVADIYAPIRSGTDITFLSGVLRYLIENNKINAE
YVKHYTNASLLVRDDFAFEDGLFSGYDAEKRQYDKSSWNYQLDENGYAKRDETLTHPRCV
WNLLKEHVSRYTPDVVENICGTPKADFLKVCEVLASTSAPDRTTTFLYALGWTQHTVGAQ
NIRTMAMIQLLLGNMGMAGGGVNALRGHSNIQGLTDLGLLSTSLPGYLTLPSEKQVDLQS
YLEANTPKATLADQVNYWSNYPKFFVSLMKSFYGDAAQKENNWGYDWLPKWDQTYDVIKY
FNMMDEGKVTGYFCQGFNPVASFPDKNKVVSCLSKLKYMVVIDPLVTETSTFWQNHGESN
DVDPASIQTEVFRLPSTCFAEEDGSIANSGRWLQWHWKGQDAPGEARNDGEILAGIYHHL
RELYQSEGGKGVEPLMKMSWNYKQPHEPQSDEVAKENNGYALEDLYDANGVLIAKKGQLL
SSFAHLRDDGTTASSCWIYTGSWTEQGNQMANRDNSDPSGLGNTLGWAWAWPLNRRVLYN
RASADINGKPWDPKRMLIQWNGSKWTGNDIPDFGNAAPGTPTGPFIMQPEGMGRLFAINK
MAEGPFPEHYEPIETPLGTNPLHPNVVSNPVVRLYEQDALRMGKKEQFPYVGTTYRLTEH
FHTWTKHALLNAIAQPEQFVEISETLAAAKGINNGDRVTVSSKRGFIRAVAVVTRRLKPL
NVNGQQVETVGIPIHWGFEGVARKGYIANTLTPNVGDANSQTPEYKAFLVNIEKA
>P35077
MTDATNRFRPGLVGRALVRAGLLFAVAACAQAQLLPGARDLNRIDDRQRKEQLQRDIERA
LTRPPVELNPQSEAAAPARKPDATSGHTVTVHAVDLDFGVEGRLFDPAPLVQDYLNRPLD
NEQLFLLVKALSAALYDRGYATSIVTFVPPGVVDGVLKLKVEWGRIKGWLIDGKPLEGTR
DRMMVFSAMPGWQDKVLNVFDIDQAIYNINNGGKTGNITIVPADEYGYSYLDLQLQRRAL
PRVSLGMDNSGPGTPENGRYKYNASVTANDLLGLNDTLGLYIGNRYYRDAGHDAERNYDL
MYSVPLGRTRLDLQTGYSTYRNLLKTRYGQYQSAGNSRSFGLKATRLLYRDTRSQFSVYG
GLKLRQNKNYLAGTRLDVSSKHYSDVTVGMQYSTQRGANAYFGDLSFTRGVGVNNGKYAA
YDERGPQGNVSRFNGSLAWTRYMALAGQPIQWASQLGFQYSRQQLLNSYQITVGDEYTVR
GYNLRTSQSGDSGVYLSNTLTVPVQFSLLGKQASVAPFVGADVGALKSNHPDARTIRMAG
LAAGVRFDLPYARMSFTYSKPVGAQPGGAPRAPVWLYINAGLSF
>P18126
MNLLSGWVRPLMLGCGLLGAALSAGSIQAAVCEYRVTNEWGSGFTASIRITNNGSSTING
WSVSWNYTDGSRVTSSWNAGLSGANPYSATPVGWNTSIPIGSSVEFGVQGNNGSSRAQVP
AVTGAICGGQGSSAPSSVASSSSSSSVVSSTPRSSSSSVSSSVPGTSSSSSSSVLTGAQA
CNWYGTLTPLCNNTSNGWGYEDGRSCVARTTCSAQPAPYGIVSTSSSTPLSSSSSSRSSV
ASSSSLSSATSSSASSVSSVPPIDGGCNGYATRYWDCCKPHCGWSANVPSLVSPLQSCSA
NNTRLSDVSVGSSCDGGGGYMCWDKIPFAVSPTLAYGYAATSSGDVCGRCYQLQFTGSSY
NAPGDPGSAALAGKTMIVQATNIGYDVSGGQFDILVPGGGVGAFNACSAQWGVSNAELGA
QYGGFLAACKQQLGYNASLSQYKSCVLNRCDSVFGSRGLTQLQQGCTWFAEWFEAADNPS
LKYKEVPCPAELTTRSGMNRSILNDIRNTCP
>Q9XDP1
MSAKWINSIFKSVVLTAALALPFTASAFTEGTDYMVLEKPIPDADKTLIKVFSYACPFCY
KYDKAVTGPVADKVADLVTFVPFHLETKGEYGKQASELFAVTMAKDKAAGVSLFDEKSQF
KKAKFAWYAAYHDKKERWSDGKDPAAFLKTGLDAAGMSQAEFEAALKEPAVQQTLQKWKA
AYEVAKIQGVPAYVVNGKYLIYTKNIKSIDSMAQLVRELATK
>P38683
MRVLLFLLLSLFMLPAFSADNLLRWHDAQHFTVQASTPLKAKRAWKLCALYPSLKDSYWL

SLNYGMQEAARRYGVDLKVLEAGGYSQLATQQAQIDQCKQWGAEAILLGSSTTSFPDLQK
QVASLPVIELVNAIDAPQVKSRVGVPWFQMGYQPGRYLVQWAHGKPLNVLLMPGPDNAGG
SKEMVEGFRAAIAGSPVRIVDIALGDNDIEIQRNLLQEMLERHPEIDVVAGTAIAAEAAM
GEGRNLKTPLTVVSFYLSHQVYRGLKRGRVIMAASDQMVWQGELAVEQAIRQLQGQSVSD
NVSPPILVLTPKNADREHIRRSLSPGGFRPVYFYQHTSAAKK
>P23847
MRISLKKSGMLKLGLSLVAMTVAASVQAKTLVYCSEGSPEGFNPQLFTSGTTYDASSVPL
YNRLVEFKIGTTEVIPGLAEKWEVSEDGKTYTFHLRKGVKWHDNKEFKPTRELNADDVVF
SFDRQKNAQNPYHKVSGGSYEYFEGMGLPELISEVKKVDDNTVQFVLTRPEAPFLADLAM
DFASILSKEYADAMMKAGTPEKLDLNPIGTGPFQLQQYQKDSRIRYKAFDGYWGTKPQID
TLVFSITPDASVRYAKLQKNECQVMPYPNPADIARMKQDKSINLMEMPGLNVGYLSYNVQ
KKPLDDVKVRQALTYAVNKDAIIKAVYQGAGVSAKNLIPPTMWGYNDDVQDYTYDPEKAK
ALLKEAGLEKGFSIDLWAMPVQRPYNPNARRMAEMIQADWAKVGVQAKIVTYEWGEYLKR
AKDGEHQTVMMGWTGDNGDPDNFFATLFSCAASEQGSNYSKWCYKPFEDLIQPARATDDH
NKRVELYKQAQVVMHDQAPALIIAHSTVFEPVRKEVKGYVVDPLGKHHFENVSIE
>P07662
MLRVLHRAASALVMATVIGLAPAVAFALAEPTSTPQAPIAAYKPRSNEILWDGYGVPHIY
GVDAPSAFYGYGWAQARSHGDNILRLYGEARGKGAEYWGPDYEQTTVWLLTNGVPERAQQ
WYAQQSPDFRANLDAFAAGINAYAQQNPDDISPEVRQVLPVSGADVVAHAHRLMNFLYVA
SPGRTLGEGDPPDLADQGSNSWAVAPGKTANGNALLLQNPHLSWTTDYFTYYEAHLVTPD
FEIYGATQIGLPVIRFAFNQRMGITNTVNGMVGATNYRLTLQDGGYLYDGQVRPFERPQA
SYRLRQADGTTVDKPLEIRSSVHGPVFERADGTAVAVRVAGLDRPGMLEQYFDMITADSF
DDYEAALARMQVPTFNIVYADREGTINYSFNGVAPKRAEGDIAFWQGLVPGDSSRYLWTE
THPLDDLPRVTNPPGGFVQNSNDPPWTPTWPVTYTPKDFPSYLAPQTPHSLRAQQSVRLM
SENDDLTLERFMALQLSHRAVMADRTLPDLIPAALIDPDPEVQAAARLLAAWDREFTSDS
RAALLFEEWARLFAGQNFAGQAGFATPWSLDKPVSTPYGVRDPKAAVDQLRTAIANTKRK
YGAIDRPFGDASRMILNDVNVPGAAGYGNLGSFRVFTWSDPDENGVRTPVHGETWVAMIE
FSTPVRAYGLMSYGNSRQPGTTHYSDQIERVSRADFRELLLRREQVEAAVQERTPFNFKP

>P42213
MKLFKSILLIAACHAAQASATIDINADPNLTGAAPLTGILNGQKSDTQNMSGFDNTPPPA
PPVVMSRMFGAQLFNGTSADSGATVGFNPDYILNPGDSIQVRLWGAFTFDGALQVDPKGN
IFLPNVGPVKIAGVSNSQLNALVTSKVKEVYQSNVNVYASLLQAQPVKVYVTGFVRNPGL
YGGVTSDSLLNYLIKAGGVDPERGSYVDIVVKRGNRVRSNVNLYDFLLNGKLGLSQFADG
DTIIVGPRQHTFSVQGDVFNSYDFEFRESSIPVTEALSWARPKPGATHITIMRKQGLQKR
SEYYPISSAPGRMLQNGDTLIVSTDRYAGTIQVRVEGAHSGEHAMVLPYGSTMRAVLEKV
RPNSMSQMNAVQLYRPSVAQRQKEMLNLSLQKLEEASLSAQSSTKEEASLRMQEAQLISR
FVAKARTVVPKGEVILNESNIDSVLLEDGDVINIPEKTSLVMVHGEVLFPNAVSWQKGMT
TEDYIEKCGGLTQKSGNARIIVIRQNGARVNAEDVDSLKPGDEIMVLPKYESKNIEVTRG
ISTILYQLAVGAKVILSL
>Q51393
MTQSISRPLQYAYIAAFGGLLLGLAGWSLKSVPGFSAAADTPLLNGKLAHAFEAHYDKEF
PIKRLGTNLWAALDYTLFHEGRPGVVIGKDGWLFTDEEFKPAPSGQQLEDNWALVRGVQR
ELNRRGVKLVLAVIPAKARLYPEHIGREQPAALHDSLYQDFLARARAAGIDSPDLLGSLR

QAKDNGAVFLRTDTHWSPLGAETVAQRLGAEIRETHLLDVPAQNFVTRVGEERTHKGDLL
SFLPLDPLFDELLPRPEQLQQRTTEAAPALPGGQQSGAGDDLFGDSQQPRLALVGTSYSA
NPRWNFEGALKQALSADLINYAKEGKGPLEPMLELLQDEGFRKDPPQLLVWEFPERYLPM
ASDLSQFDADWVAQLKASGGRDERLAASRND
>P00099
MKPYALLSLLATGTLLAQGAWAEDPEVLFKNKGCVACHAIDTKMVGPAYKDVAAKFAGQA
GAEAELAQRIKNGSQGVWGPIPMPPNAVSDDEAQTLAKWVLSQK
>P0AG82
MKVMRTTVATVVAATLSMSAFSVFAEASLTGAGATFPAPVYAKWADTYQKETGNKVNYQG
IGSSGGVKQIIANTVDFGASDAPLSDEKLAQEGLFQFPTVIGGVVLAVNIPGLKSGELVL
DGKTLGDIYLGKIKKWDDEAIAKLNPGLKLPSQNIAVVRRADGSGTSFVFTSYLAKVNEE
WKNNVGTGSTVKWPIGLGGKGNDGIAAFVQRLPGAIGYVEYAYAKQNNLAYTKLISADGK
PVSPTEENFANAAKGADWSKTFAQDLTNQKGEDAWPITSTTFILIHKDQKKPEQGTEVLK
FFDWAYKTGAKQANDLDYASLPDSVVEQVRAAWKTNIKDSSGKPLY
>P37994
MLRNLVIFAVLGAGLTTLAAAGQDINNFTQAKAAAAKIHQDAPGTFYCGCKINWQGKKGT
PDLASCGYQVRKDANRASRIEWEHVVPAWQFGHQRQCWQDGGRKNCTKDDVYRQIETDLH
NLQPAIGEVNGDRGNFMYSQWNGGERQYGQCEMKIDFKSQLAEPPERARGAIARTYFYMR
DRYNLNLSRQQTQLFDAWNKQYPATTWECTREKRIAAVQGNHNPYVQQACSPDAAPYYNG
LSLIMIAAVATVAARWLTPAGHLPSD
>P0ABE7
MRKSLLAILAVSSLVFSSASFAADLEDNMETLNDNLKVIEKADNAAQVKDALTKMRAAAL
DAQKATPPKLEDKSPDSPEMKDFRHGFDILVGQIDDALKLANEGKVKEAQAAAEQLKTTR
NAYHQKYR
>P18473
MKLSMKSLAALLMMLNGAVMASENVNTPENRQFLKQQENLSRQLREKPDHQLKAWAEKQV
LENPLQRSDNHFLDELVRKQQASQDGKPRQGALYFVSFSIPEEGLKRMLGETRHFGIPAT
LRGMVNNDLKTTAEAVLSLVKDGATDGVQIDPTLFSQYGIRTVPALVVFCSQGYDIIRGN
LRVGQALEKVAATGDCRQVAHDLLAGKGDSGK
>P0AB24
MTINFRRNALQLSVAALFSSAFMANAADVPQVKVTVTDKQCEPMTITVNAGKTQFIIQNH
SQKALEWEILKGVMVVEERENIAPGFSQKMTANLQPGEYDMTCGLLTNPKGKLIVKGEAT
ADAAQSDALLSLGGAITAYKAYVMAETTQLVTDTKAFTDAIKAGDIEKAKALYAPTRQHY
ERIEPIAELFSDLDGSIDAREDDYEQKAADPKFTGFHRLEKALFGDNTTKGMDQYAEQLY
TDVVDLQKRISELAFPPSKVVGGAAGLIEEVAASKISGEEDRYSHTDLWDFQANVEGSQK
IVDLLRPQLQKANPELLAKVDANFKKVDTILAKYRTKDGFETYDKLTDADRNALKGPITA
LAEDLAQLRGVLGLD
>Q46684
MEKSATRQKALLIALPLLFSPLASAVQQAVLDTRGAPLITVNGLTFKDLNRDGKLNPYED
WRLPAAERAADLVSRMTLAEKAGVMMHGSAPTAGSVTGAGTQYDLNAAKTMIADRYVNSF
ITRLSGDNPAQMAEENNKLQQLAEATRLGIPLTISTDPRSSFQSLVGVSVSVGKFSKWPE
TLGLAAIGDEELVRRFADIVRQEYRAVGITEALSPQADLATEPRWPRIDGTFGEDPDLTK
KMVRGYVTGMQNGKNGLNAQSVISIVKHWVGYGAAKDGWDSHNVYGKYAQFRQNNLQWHI
DPFTGAFEAHAAGIMPTYSILRNASWHGKPIEQVGAGFNRFLLTDLLRGQYGFDGVILSD

WLITNDCKGDCLTGVKPGEKPVPRGMPWGVEKLTPAERFVKAVNAGVDQFGGVTDSALLV
QAVQDGKLTEARLDTSVNRILKQKFQTGLFERPYVNATQANDIVGRADWQQLADDTQARS
LVLLQNNNLLPLRKGSRVWLHGIAANAAQEVGFIVVNTPEQADVALIRTHTPYEQPHKNF
FFGSRHHEGSLAFRNDNPDYQAIVRASAKVPTLVTVYMERPAILTNVVDKTRAVVANFGV
SDSVLLNRLMSGAAYTAKLPFELPSSMSAVRNQQPDLPYDSAKPLFPFGYGLPH
>Q51705
MESKQEKGLSRRALLGATAGGAAVAGAFGGRLALGPAALGLGTAGVATVAGSGAALAASG
DGSVAPGQLDDYYGFWSSGQSGEMRILGIPSMRELMRVPVFNRCSATGWGQTNESVRIHE
RTMSERTKKFLAANGKRIHDNGDLHHVHMSFTEGKYDGRFLFMNDKANTRVARVRCDVMK
CDAILEIPNAKGIHGLRPQKWPRSNYVFCNGEDETPLVNDGTNMEDVANYVNVFTAVDAD
KWEVAWQVLVSGNLDNCDADYEGKWAFSTSYNSEKGMTLPEMTAAEMDHIVVFNIAEIEK
AIAAGDYQELNGVKVVDGRKEASSLFTRYIPIANNPHGCNMAPDKKHLCVAGKLSPTATV
LDVTRFDAVFYENADPRSAVVAEPELGLGPLHTAFDGRGNAYTSLFLDSQVVKWNIEDAI
RAYAGEKVDPIKDKLDVHYQPGHLKTVMGETLDATNDWLVCLSKFSKDRFLNVGPLKPEN
DQLIDISGDKMVLVHDGPTFAEPHDAIAVHPSILSDIKSVWDRNDPMWAETRAQAEADGV
DIDNWTEEVIRDGNKVRVYMSSVAPSFSIESFTVKEGDEVTVIVTNLDEIDDLTHGFTMG
NYGVAMEIGPQMTSSVTFVAANPGVYWYYCQWFCHALHMEMRGRMLVEPKEA
>P07822
MSGLPLISRRRLLTAMALSPLLWQMNTAHAAAIDPNRIVALEWLPVELLLALGIVPYGVA
DTINYRLWVSEPPLPDSVIDVGLRTEPNLELLTEMKPSFMVWSAGYGPSPEMLARIAPGR
GFNFSDGKQPLAMARKSLTEMADLLNLQSAAETHLAQYEDFIRSMKPRFVKRGARPLLLT
TLIDPRHMLVFGPNSLFQEILDEYGIPNAWQGETNFWGSTAVSIDRLAAYKDVDVLCFDH
DNSKDMDALMATPLWQAMPFVRAGRFQRVPAVWFYGATLSAMHFVRVLDNAIGGKA
>P15069
MMPRIKPLLVLCAALLTVTPAASADVNSDMNQFFNKLGFASNTTQPGVWQGQAAGYAYGG
SLYARTQVKNVQLISMTLPDINAGCGGIDAYLGSFSFINGEQLQRFVKQIMSNAAGYFFD
LALQTTVPEIKTAKDFLQKMASDINSMNLSSCQAAQGIIGGLFPRTQVSQQKVCQDIAGE
SNIFADWAASRQGCTVGGKSDSVRDKASDKDKERVTKNINIMWNALSKNRMFDGNKELKE
FVMTLTGSLVFGPNGEITPLSARTTDRSIIRAMMEGGTAKISHCNDSDKCLKVVADTPVT
ISRDNALKSQITKLLASIQNKAVSDTPLDDKEKGFISSTTIPVFKYLVDPQMLGVSNSMI
YQLTDYIGYDILLQYIQELIQQARAMVATGNYDEAVIGHINDNMNDATRQIAAFQSQVQV
QQDALLVVDRQMSYMRQQLSARMLSRYQNNYHFGGSTL
>P00260
MSDKPISKSRRDAVKVMLGTAAAIPMINLVGFGTARASAPANAVAADDATAIALKYNQDA
TKSERVAAARPGLPPEEQHCANCQFMQADAAGATDEWKGCQLFPGKLINVNGWCASWTLK
AG
>Q8GPG1
MPGFRFLLAATAAFLATSPALPLSADSLNAGNIRLVDPEETVPVIKIPDGIYLRTPNDPD
DIIWARVPEFRVEMVMAPPVHPSVGLRYRDEYPEQDLVVQLARTSERFYVRLRWVDPTRD
MSTLRDRFRDGAAIEFSESDDSVSYMMGTDAESPVNIWYWHPDGDRVESLAAGSPGSLTR
LDRQPVTGASEYRTGHGPDDSQWIVVMSRPLASEGDHQVSFERDTIPVAFALWQGADAQR
DGLKLVSLNWIFARMTPDAAPAPGN
>P17543
MVVNKTTAVLYLIALSLSGFIHTFLRAEERGIYDDVFTADALRHYRINERGGRTGSLTCS

GALLSSPCTLVSNEVPLSLRPENHSAAAGAPLMLRLAGCGDGGALQPGKRGVAMTVSGSL
VTGPGSGSALLPDRKLSGCDHLVIHDGDTFLLCRPDRRQEEMLAAWRKRATQEGEYSDAR
SNPAMLRLSIKYE
>Q9L7P2
MQTPKLIRPTLLSMAILSSMAWATGASAALVPPKGYDAPIEKMKTGDHNFSCEAIPKPYT
DKLVFRSKYEGSDKARATLNAVSEEAFRDATKDITTLERGVSKVVMQYMRDGRPEQLDCA
LNMMTTWAKADALESREFNHTGKSMRKWALGSMSSAYLRLKFSESHPLANRQQDAKIIET
WFSKLADQVVSDWSNLPLEKINNHSYWAAWSVMATAVATNRQDLFDWAVKEYKVAANQVD
KDGFLPNEMKRRQRALSYHNYALPPLAMIASFAQANGVDLRPENNGALKRLGDRVLAGVK
DPSIFAEHNGEKQDMTDLKKDPKFAWLEPYCSLYTCSPDVLEEKHEKQPFKTFRLGGDLT
KVYDPTHEKGDKGDNDGS
>P0AFH8
MTMTRLKISKTLLAVMLTSAVATGSAYAENNAQTTNESAGQKVDSSMNKVGNFMDDSAIT
AKVKAALVDHDNIKSTDISVKTDQKVVTLSGFVESQAQAEEAVKVAKGVEGVTSVSDKLH
VRDAKEGSVKGYAGDTATTSEIKAKLLADDIVPSRHVKVETTDGVVQLSGTVDSQAQSDR
AESIAKAVDGVKSVKNDLKTK
>P0AEL6
MRLAPLYRNALLLTGLLLSGIAAVQAADWPRQITDSRGTHTLESQPQRIVSTSVTLTGSL
LAIDAPVIASGATTPNNRVADDQGFLRQWSKVAKERKLQRLYIGEPSAEAVAAQMPDLIL
ISATGGDSALALYDQLSTIAPTLIINYDDKSWQSLLTQLGEITGHEKQAAERIAQFDKQL
AAAKEQIKLPPQPVTAIVYTAAAHSANLWTPESAQGQMLEQLGFTLAKLPAGLNASQSQG
KRHDIIQLGGENLAAGLNGESLFLFAGDQKDADAIYANPLLAHLPAVQNKQVYALGTETF
RLDYYSAMQVLDRLKALF
>P14497
MNKALLPLLLCCFIFPASGKDAGWQWYNEKINPKEKENKPVPAAPRQEPDIMQKLAALQT
ATKRALYEAILYPGVDNFVKYFRLQNYWAQQAGLFTMSARKAMLAHPELDYNLQYSHYNG
TVRNQLAADQAQQRQAIAKLAEHYGIMFFYRGQDPIDGQLAQVINGFRDTYGLSVIPVSV
DGVINPLLPDSRTDQGQAQRLGVKYFPAMMLVDPKQGSVRPLSYGFISQDDLAKQFLNVS
EDFKPNF
>P0AD59
MGRISSGGMMFKAITTVAALVIATSAMAQDDLTISSLAKGETTKAAFNQMVQGHKLPAWV
MKGGTYTPAQTVTLGDETYQVMSACKPHDCGSQRIAVMWSEKSNQMTGLFSTIDEKTSQE
KLTWLNVNDALSIDGKTVLFAALTGSLENHPDGFNFK
>P37975
MMKSRTRRLSLSTLFGALLGVSVAAAWLYYSHRNEAGHGDLHEILHEAVPLDANEREILE
LKEDAFAQRRREIETRLRAANGKLADAIAKNPAWSPEVEAATQEVERAAGDLQRATLVHV
FEMRAGLKPEHRPAYDRVLIDALRRGSQ
>P15931
MIGDGKLLASAAWDAQSLNELKAKAGQDPAANIRPVARQVEGMFVQMMLKSMREALPKDG
LFSSDQTRLYTSMYDQQIAQQMTAGKGLGLADMMVKQMTSGQTMPADDAPQVPLKFSLET
VNSYQNQALTQLVRKAIPKTPDSSDAPLSGDSKDFLARLSLPARLASEQSGVPHHLILAQ
AALESGWGQRQILRENGEPSYNVFGVKATASWKGPVTEITTTEYENGEAKKVKAKFRVYS
SYLEALSDYVALLTRNPRYAAVTTAATAEQGAVALQNAGYATDPNYARKLTSMIQQLKAM
SEKVSKTYSANLDNLF

>Q47459
MKKILVSFVAIMAVASSAMAAETMNMHDQVNNAQAPAHQMQSSAEKSAVQGDSMTMMDMS
SHDQAAMSHDMMQNGNSAAHQDMAEMHKKMMKSKPAASNETAKSFSEMNEHEKSAVVHEK
ANNGQSSVIHQQQAEKHRSQITQN
>P21853
MKISIGLGKEGVEERLAERGVSRRDFLKFCTAIAVTMGMGPAFAPEVARALMGPRRPSVV
YLHNAECTGCSESVLRAFEPYIDTLILDTLSLDYHETIMAAAGDAAEAALEQAVNSPHGF
IAVVEGGIPTAANGIYGKVANHTMLDICSRILPKAQAVIAYGTCATFGGVQAAKPNPTGA
KGVNDALKHLGVKAINIAGCPPNPYNLVGTIVYYLKNKAAPELDSLNRPTMFFGQTVHEQ
CPRLPHFDAGEFAPSFESEEARKGWCLYELGCKGPVTMNNCPKIKFNQTNWPVDAGHPCI
GCSEPDFWDAMTPFYQN
>A2TJI4
MKKYILGVILAMGSLSAIAGGGNSERPPSVAAGECVTFNSKLGEIGGYSWKYSNDACNET
VAKGYAIGVAMHRTVNYEGGYSIQSSGIVKPGSDFIMKGGKTYKGHKKVSAGGDTPYWYK

>P42517
MTHFVAIFFSSLFMCSNVFAGSVSSVSLGSLSSALNERMQVMKAVAGYKALHHLPIEDLP
REQVVLDHMLQNAQQAGLEPHSVEPFVHALMNASKTIQYRYRADWLSSPDSAVPVRDLTE
TRQQIQQLDTQLLTAISQRLMTGAFSQEDKEFLMSHLTAPHLSESDKNSLFASLSRIQRQ
H
>P0A326
MTDRPIMTTSAGAPIPDNQNSLTAGERGPILMQDYQLIEKLSHQNRERIPERAVHAKGWG
AYGTLTITGDISRYTKAKVLQPGAQTPMLARFSTVAGELGAADAERDVRGFALKFYTQEG
NWDLVGNNTPVFFVRDPLKFPDFIHTQKRHPRTHLRSATAMWDFWSLSPESLHQVTILMS
DRGLPTDVRHINGYGSHTYSFWNDAGERYWVKFHFKTMQGHKHWTNAEAEQVIGRTREST
QEDLFSAIENGEFPKWKVQVQIMPELDADKTPYNPFDLTKVWPHADYPPIDIGVMELNRN
PENYFTEVENAAFSPSNIVPGIGFSPDKMLQARIFSYADAHRHRLGTHYESIPVNQPKCP
VHHYHRDGQMNVYGGIKTGNPDAYYEPNSFNGPVEQPSAKEPPLCISGNADRYNHRIGND
DYSQPRALFNLFDAAQKQRLFSNIAAAMKGVPGFIVERQLGHFKLIHPEYEAGVRKALKD
AHGYDANTIALNEKITAAE
>P0AEE5
MNKKVLTLSAVMASMLFGAAAHAADTRIGVTIYKYDDNFMSVVRKAIEQDAKAAPDVQLL
MNDSQNDQSKQNDQIDVLLAKGVKALAINLVDPAAAGTVIEKARGQNVPVVFFNKEPSRK
ALDSYDKAYYVGTDSKESGIIQGDLIAKHWAANQGWDLNKDGQIQFVLLKGEPGHPDAEA
RTTYVIKELNDKGIKTEQLQLDTAMWDTAQAKDKMDAWLSGPNANKIEVVIANNDAMAMG
AVEALKAHNKSSIPVFGVDALPEALALVKSGALAGTVLNDANNQAKATFDLAKNLADGKG
AADGTNWKIDNKVVRVPYVGVDKDNLAEFSKK
>Q3J2P2
MRPIPALALTFSLVAMPALAQDARQIERMIEGRHGLMTLMAHELGKLGGMAKEETPYDAE
VAGKAASNLSALASVISPELFPKGSAVGEAEDSEALPAIWEKPDDFAQKISGMEEAAAKM
QAAAGTDLASLQGAMRDLGAACGSCHETYRQKD
>P07102
MKAILIPFLSLLIPLTPQSAFAQSEPELKLESVVIVSRHGVRAPTKATQLMQDVTPDAWP
TWPVKLGWLTPRGGELIAYLGHYQRQRLVADGLLAKKGCPQSGQVAIIADVDERTRKTGE

AFAAGLAPDCAITVHTQADTSSPDPLFNPLKTGVCQLDNANVTDAILSRAGGSIADFTGH
RQTAFRELERVLNFPQSNLCLKREKQDESCSLTQALPSELKVSADNVSLTGAVSLASMLT
EIFLLQQAQGMPEPGWGRITDSHQWNTLLSLHNAQFYLLQRTPEVARSRATPLLDLIKTA
LTPHPPQKQAYGVTLPTSVLFIAGHDTNLANLGGALELNWTLPGQPDNTPPGGELVFERW
RRLSDNSQWIQVSLVFQTLQQMRDKTPLSLNTPPGEVKLTLAGCEERNAQGMCSLAGFTQ
IVNEARIPACSL
>P0AFM2
MRHSVLFATAFATLISTQTFAADLPGKGITVNPVQSTITEETFQTLLVSRALEKLGYTVN
KPSEVDYNVGYTSLASGDATFTAVNWTPLHDNMYEAAGGDKKFYREGVFVNGAAQGYLID
KKTADQYKITNIAQLKDPKIAKLFDTNGDGKADLTGCNPGWGCEGAINHQLAAYELTNTV
THNQGNYAAMMADTISRYKEGKPVFYYTWTPYWVSNELKPGKDVVWLQVPFSALPGDKNA
DTKLPNGANYGFPVSTMHIVANKAWAEKNPAAAKLFAIMQLPVADINAQNAIMHDGKASE
GDIQGHVDGWIKAHQQQFDGWVNEALAAQK
>P24037
MKKTLMASAVGAVIAFGTHGAMAAAPADWSSVAATDVTLFYPGVSPVEWITKGTEHGGAR
ALKKGETCAGCHSEEASDMGEKMASGKKLEPSPIAGKAPFINAKVQAANDGENLYLRFTW
KQPAASGAAPMDADNPVKIAYMLEGGSKVELAEAGGCWGSCHGDARTMPGAADTKTKYVK
DGSLANGVYYDLNQWRSGENKAFDGYVATERVMEGGQALVDAQGKLDGDTWTVVFTRKFA
GGEGDVTLAPGNLYNFGFAIHDDSATGRYHHVSLGYSLGIDAQGDITAAKQ
>O53021
MSKRILAPVVTVLSLTAFSPAFAATTSTHVLLTTSAGNIELALDDQKAPVFVKNFVDYVN
SGFYNGTIFHRVIPGFMVQGGGFSSDMKQKATNPPVKNEADNGLRNLRGTISMARTSEKD
SATSQFFINVADNAFLDHGQRDFGYAVFGKVVKGMEVADKISQVQTENVGPYQNVPSKPI
VIQSAKIIKK
>P33225
MNNNDLFQASRRRFLAQLGGLTVAGMLGPSLLTPRRATAAQAATDAVISKEGILTGSHWG
AIRATVKDGRFVAAKPFELDKYPSKMIAGLPDHVHNAARIRYPMVRVDWLRKRHLSDTSQ
RGDNRFVRVSWDEALDMFYEELERVQKTHGPSALLTASGWQSTGMFHNASGMLAKAIALH
GNSVGTGGDYSTGAAQVILPRVVGSMEVYEQQTSWPLVLQNSKTIVLWGSDLLKNQQANW
WCPDHDVYEYYAQLKAKVAAGEIEVISIDPVVTSTHEYLGREHVKHIAVNPQTDVPLQLA
LAHTLYSENLYDKNFLANYCVGFEQFLPYLLGEKDGQPKDAAWAEKLTGIDAETIRGLAR
QMAANRTQIIAGWCVQRMQHGEQWAWMIVVLAAMLGQIGLPGGGFGFGWHYNGAGTPGRK
GVILSGFSGSTSIPPVHDNSDYKGYSSTIPIARFIDAILEPGKVINWNGKSVKLPPLKMC
IFAGTNPFHRHQQINRIIEGLRKLETVIAIDNQWTSTCRFADIVLPATTQFERNDLDQYG
NHSNRGIIAMKQVVPPQFEARNDFDIFRELCRRFNREEAFTEGLDEMGWLKRIWQEGVQQ
GKGRGVHLPAFDDFWNNKEYVEFDHPQMFVRHQAFREDPDLEPLGTPSGLIEIYSKTIAD
MNYDDCQGHPMWFEKIERSHGGPGSQKYPLHLQSVHPDFRLHSQLCESETLRQQYTVAGK
EPVFINPQDASARGIRNGDVVRVFNARGQVLAGAVVSDRYAPGVARIHEGAWYDPDKGGE
PGALCKYGNPNVLTIDIGTSQLAQATSAHTTLVEIEKYNGTVEQVTAFNGPVEMVAQCEY
VPASQVKS
>Q82S91
MKTTLIKVIAASVTALFLSMQVYASGHTAHVDEAVKHAEEAVAHGKEGHTDQLLEHAKES
LTHAKAASEAGGNTHVGHGIKHLEDAIKHGEEGHVGVATKHAQEAIEHLRASEHKSH
>P13656

MKLNIFTKSMIGMGLVCSALPALAMEAWNNQQGGNKYQVIFDGKIYENAWWVSSTNCPGK
AKANDATNPWRLKRTATAAEISQFGNTLSCEKSGSSSSSNSNTPASNTPANGGSATPAQG
TVPSNSSVVAWNKQQGGQTWYVVFNGAVYKNAWWVASSNCPGDAKSNDASNPWRYVRAAT
ATEISETSNPQSCTSAPQPSPDVKPAPDVKPAPDVQPAPADKSNDNYAVVAWKGQEGSST
WYVIYNGGIYKNAWWVGAANCPGDAKENDASNPWRYVRAATATEISQYGNPGSCSVKPDN
NGGAVTPVDPTPETPVTPTPDNSEPSTPADSVNDYSLQAWSGQEGSEIYHVIFNGNVYKN
AWWVGSKDCPRGTSAENSNNPWRLERTATAAELSQYGNPTTCEIDNGGVIVADGFQASKA
YSADSIVDYNDAHYKTSVDQDAWGFVPGGDNPWKKYEPAKAWSASTVYVKGDRVVVDGQA
YEALFWTQSDNPALVANQNATGSNSRPWKPLGKAQSYSNEELNNAPQFNPETLYASDTLI
RFNGVNYISQSKVQKVSPSDSNPWRVFVDWTGTKERVGTPKKAWPKHVYAPYVDFTLNTI
PDLAALAKNHNVNHFTLAFVVSKDANTCLPTWGTAYGMQNYAQYSKIKALREAGGDVMLS
IGGANNAPLAASCKNVDDLMQHYYDIVDNLNLKVLDFDIEGTWVADQASIERRNLAVKKV
QDKWKSEGKDIAIWYTLPILPTGLTPEGMNVLSDAKAKGVELAGVNVMTMDYGNAICQSA
NTEGQNIHGKCATSAIANLHSQLKGLHPNKSDAEIDAMMGTTPMVGVNDVQGEVFYLSDA
RLVMQDAQKRNLGMVGIWSIARDLPGGTNLSPEFHGLTKEQAPKYAFSEIFAPFTKQ
>Q9Z4P0
MKLKYLVSAMALVVLSSGTAMAKTPDMGSFHADMGSCQSCHAKPIKVTDSETHENAQCKS
CHGEYAELANDKLQFDPHNSHLGDINCTSCHKGHEEPKFYCNECHSFDIKPMPFSDAKKK
KSWDDGWDQDKIQKAIAAGPSETTQVLVVGAGSAGFNASLAAKKAGANVILVDKAPFSGG
NSMISAGGMNAVGTKQQTAHGVEDKVEWFIEDAMKGGRQQNDIKLVTILAEQSADGVQWL
ESLGANLDDLKRSGGARVDRTHRPHGGKSSGPEIIDTLRKAAKEQGIDTRLNSRVVKLVV
NDDHSVVGAVVHGKHTGYYMIGAKSVVLATGGYGMNKEMIAYYRPTMKDMTSSNNITATG
DGVLMAKEIGASMTDIDWVQAHPTVGKDSRILISETVRGVGAVMVNKDGNRFISELTTRD
KASDAILKQPGQFAWIIFDNQLYKKAKMVRGYDHLEMLYKGDTVEQLAKSTGMKVADLAK
TVSDYNGYVASGKDTAFGRADMPLNMTQSPYYAVKVAPGIHHTMGGVAINTTASVLDLQS
KPIDGLFAAGEVTGGVHGYNRLGGNAIADTVVFGRIAGDNAAKHALDK
>P39265
MNKYLKYFSGTLVGLMLSTSAFAAAEYAVVLKTLSNPFWVDMKKGIEDEAKTLGVSVDIF
ASPSEGDFQSQLQLFEDLSNKNYKGIAFAPLSSVNLVMPVARAWKKGIYLVNLDEKIDMD
NLKKAGGNVEAFVTTDNVAVGAKGASFIIDKLGAEGGEVAIIEGKAGNASGEARRNGATE
AFKKASQIKLVASQPADWDRIKALDVATNVLQRNPNIKAIYCANDTMAMGVAQAVANAGK
TGKVLVVGTDGIPEARKMVEAGQMTATVAQNPADIGATGLKLMVDAEKSGKVIPLDKAPE
FKLVDSILVTQ
>P0AG80
MKPLHYTASALALGLALMGNAQAVTTIPFWHSMEGELGKEVDSLAQRFNAENPDYKIVPT
YKGNYEQNLSAGIAAFRTGNAPAILQVYEVGTATMMASKAIKPVYDVFKEAGIQFDESQF
VPTVSGYYSDSKTGHLLSQPFNSSTPVLYYNKDAFKKAGLDPEQPPKTWQDLADYAAKLK
ASGMKCGYASGWQGWIQLENFSAWNGLPFASKNNGFDGTDAVLEFNKPEQVKHIAMLEEM
NKKGDFSYVGRKDESTEKFYNGDCAMTTASSGSLANIREYAKFNYGVGMMPYDADAKDAP
QNAIIGGASLWVMQGKDKETYTGVAKFLDFLAKPENAAEWHQKTGYLPITKAAYDLTREQ
GFYEKNPGADTATRQMLNKPPLPFTKGLRLGNMPQIRVIVDEELESVWTGKKTPQQALDT
AVERGNQLLRRFEKSTKS
>P18471
MKRRLWLLMLFLFAGHVPAASADSACEGRFVNPITDICWSCIFPLSLGSIKVSQGKVPDT

ANPSMPIQICPAPPPLFRRIGLAIGYWEPMALTDVTRSPGCMVNLGFSLPAFGKTAQGTA
KKDEKQVNGAFYHVHWYKYPLTYWLNIITSLGCLEGGDMDIAYLSEIDPTWTDSSLTTIL
NPEAVIFANPIAQGACAADAIASAFNMPLDVLFWCAGSQGSMYPFNGWVSNESSPLQSSL
LVSERMAFKLHRQGMIMETIGKNNAVCNEYPSPILPKERWRYQMVNMYPDSGQCHPFGRS
VTRWETGKNPPNTKKNFGYLMWRKRNCVFL
>P39691
MKKRVVLFSLLTLALSGVARADDAAIKQTLNRLGLQSAEVKDSPIGGMKTVLTENGVLYI
TEDGKHLLQGPLYDVSGKTPVNVTNHILNERLDALKDQMIVYKAPQEKHVITVFTDITCG
YCHKLHEQMKDYNALGITVRYLAYPRQGMNSQAAKDMQSIWCVADRNKAFDAAMKGDDVS
PATCKTDIGAHYQLGVLFGVQGTPAIVLDDGTVVPGYQPPKEMMAMLDAHKASLKSGG
>Q9Z4P4
MKFKLLLAGSLVAVGAMALLASNINEKEKQRVELAKAPSEAGIAGKEKSEEWAKYYPRQF
DSWKKTKEYDSFTDMLAKDPALVIAWSGYAFSKDYNSPRGHYYALQDNVNSLRTGAPVDA
KTGPLPTACWTCKSPDVPRLIEEDGELEYFTGKWAKYGSQIVNVIGCANCHDDKTAELKV
RVPHLNRGLQAAGLKTFEESTHQDKRTLVCAQCHVEYYFKKTEWKDAKGADKTAMVVTLP
WANGVGKDGNAGVEGMIKYYDEINFSDWTHNISKTPMLKAQHPGFEFWKSGIHGQKGVSC
ADCHMPYTQEGSVKYSDHQVKENPLDSMDQSCMNCHRESESKLRGIVHQKYERKEFLNKV
AFDNIGKAHLETGKAIEAGASDEELKEVRKLIRHGQFKADMAIAAHGNYFHAPEETLRLL
AAGSDDAQKARLLLVKILAKHGVMDYIAPDFDTKDKAQKLAKVDIAALAAEKMKFKQTLE
QEWKKEAKAKGRANPELYKDVDTINDGKSSWNKK
>P35482
MFKRSLIAASLSVAALVSAQAMAVTGGGASLPAELYKGSADSILPANFSYAVTGSGTGKN
AFLTNNSSLFGTTGTVHYAGSDSVLSGSELTTYNSNYNGTYGPLIQIPSVATSVTVPYRK
DGNTTLNLTSAQLCDAFSGAKTTWGQLLGTTDSTPIRIVYRTGSSGTTELFTRHLNSICP
TRFATNSTFTNARLPAGGTLPSNWVGVAATSTVVSTVKATNGSLGYVSPDAVNINSNAEV
SRVNGNLPTQANVSTALGSVAPPANAADRADPSKWVPVFTNPSAGYSIVGYTNFVFGQCY
KDASVSTDVRAFINKHYGGTTTNAAVAAHGFIPLTPAWKSAIVSAFYTGTSENLAIGNTN
VCNTKGRP
>P18956
MIKPTFLRRVAIAALLSGSCFSAAAAPPAPPVSYGVEEDVFHPVRAKQGMVASVDATATQ
VGVDILKEGGNAVDAAVAVGYALAVTHPQAGNLGGGGFMLIRSKNGNTTAIDFREMAPAK
ATRDMFLDDQGNPDSKKSLTSHLASGTPGTVAGFSLALDKYGTMPLNKVVQPAFKLARDG
FIVNDALADDLKTYGSEVLPNHENSKAIFWKEGEPLKKGDTLVQANLAKSLEMIAENGPD
EFYKGTIAEQIAQEMQKNGGLITKEDLAAYKAVERTPISGDYRGYQVYSMPPPSSGGIHI
VQILNILENFDMKKYGFGSADAMQIMAEAEKYAYADRSEYLGDPDFVKVPWQALTNKAYA
KSIADQIDINKAKPSSEIRPGKLAPYESNQTTHYSVVDKDGNAVAVTYTLNTTFGTGIVA
GESGILLNNQMDDFSAKPGVPNVYGLVGGDANAVGPNKRPLSSMSPTIVVKDGKTWLVTG
SPGGSRIITTVLQMVVNSIDYGLNVAEATNAPRFHHQWLPDELRVEKGFSPDTLKLLEAK
GQKVALKEAMGSTQSIMVGPDGELYGASDPRSVDDLTAGY
>P23485
MNPLLTDSRRQALRSASHWYAVLSGERVSPQQEARWQQWYEQDQDNQWAWQQVENLRNQL
GGVPGDVASRALHDTRLTRRHVMKGLLLLLGAGGGWQLWQSETGEGLRADYRTAKGTVSR
QQLEDGSLLTLNTQSAADVRFDAHQRTVRLWYGEIAITTAKDALQRPFRVLTRQGQLTAL
GTEFTVRQQDNFTQLDVQQHAVEVLLASAPAQKRIVNAGESLQFSASEFGAVKPLDDEST

SWTKDILSFSDKPLGEVIATLTRYRNGVLRCDPAVAGLRLSGTFPLKNTDAILNVIAQTL
PVKIQSITRYWINISPL
>Q44642
MNTRASNFLAASFSTIMLVGAFSLPAFAQENQMTTQPARIAVTGEGMMTPSPDMAILNLS
VLRQAKTAREAMTANNNEAMTANNEAMRKVLDAMKKAGIEDRDLQTGGIDIQPIYVYPDD
KNNLKEPTITGYSVSTSLTVRVRELANVGKILHESVTLGVNQGGDFNLVNDNPSAVITRG
KRAVANAIAKPKTLADAAGVGLGRVVEISELSRPPMPMPMPIARGQFRTMLAAAPDNSVP
IAAGENSYNVSVNVVFEIK
>P0AEE1
MRNLVKYVGIGLLVMGLAACDDKDTNATAQGSVAESNATGNPVNLLDGKLSFSLPADMTD
QSGKLGTQANNMHVWSDATGQKAVIVIMGDDPKEDLAVLAKRLEDQQRSRDPQLQVVTNK
AIELKGHKMQQLDSIISAKGQTAYSSVILGNVGNQLLTMQITLPADDQQKAQTTAENIIN
TLVIQ
>P40695
MRPTLTWTLLALLLCGTAIGAVLLFYPSEPAPVAPFASPPQATPAAKPSIPSRAPEMNTA
TAPDNLEQQLGEFGRNAGQMSEIERKQAAEGLIEQLKREVAVGADPRQTFEEIQRLTPYV
EADARRREALDFEIWMALKDNASVQQQAPTPGEEEQLREYAQESDKVIAEVLASVDDEEQ
RHAAIDERLKALRKQIFGEENPRLLQR
>P19567
MEKTMLNAIKSGFGIAIAAMLVAAPAAAADFEVHMLNKGKDGAMVFEPASLKVAPGDTVT
FIPTDKGHNVETIKGMIPDGAEAFKSKINENYKVTFTAPGVYGVKCTPHYGMGMVGVVQV
GDAPANLEAVKGAKNPKKAQERLDAALAALGN
>P19843
MFKAQATFSRYSAAVSLLLLFSGAAQAAPQSITTLPLQPDGENRWRLPAGEYQGQFTIEQ
PMQLRCEPGAVIQSQGQGSSLLISAPDVLVEGCTLYEWGSDLTAMDSAVFILPAAERAQI
SNNRMRGPGFGVFVDGTRDVQVIGNEIDGDAGVRSQDRGNGIHLFAVSGARVLHNHVRNA
RDGIYIDTSNGNHLEGNVIEDVRYGVHYMFANENSLIDNVTRRTRTGYALMQSRKLTVTG
NRSEQDQNYGILMNYITYSTITGNFVSDVQRGDTGGDSMISGGEGKALFIYNSLFNTIEN
NHFEKSSLGIHLTAGSEDNRISGNAFVGNQQQVKYVASRTQEWSVDGRGNYWSDYLGWDR
NNDGLGDIAYEPNDNVDRLLWLYPQVRLLMNSPSIEVLRWVQRAFPVIKSPGVQDSHPLM
KLPTEKLLTEKQEPTS
>P60069
MSQRQVAYVFDLNKCIGCHTCTMACKQLWTNRDGREYMYWNNVETRPGKGYPKNWEGKGG
GFDQEGKLKTNGIIPIMADYGGRIGDFNLNEVLLEGKADQVVPHEKATWGPNWDEDEGKG
EFPNNHSFYLPRICNHCSNPACLAACPTKAIYKRPEDGIVVVDQTRCRGYRYCVKACPYG
KMYFNLQKGKSEKCIGCYPRVEKGEAPACVKQCSGRIRFWGYRDDKNGPIYKLVEQWKVA
LPLHAEYGTEPNVFYVPPMNTTPPPFEEDGRLGDKPRIPIEDLEALFGPGVKQALATLGG
EMAKRRKAQASELTDILIGFTNKDRYGV
>Q56952
MIERLNSPFLRAAALFTIVAFSSLISTAALAENNPSDTAKKFKVVTTFTIIQDIAQNIAG
DVAVVESITKPGAEIHDYQPTPRDIVKAQSADLILWNGMNLERWFEKFFESIKDVPSAVV
TAGITPLPIREGPYSGIANPHAWMSPSNALIYIENIRKALVEHDPAHAETYNRNAQAYAE
KIKALDAPLRERLSRIPAEQRWLVTSEGAFSYLAKDYGFKEVYLWPINAEQQGIPQQVRH
VIDIIRENKIPVVFSESTISDKPAKQVSKETGAQYGGVLYVDSLSGEKGPVPTYISLINM

TVDTIAKGFGQ
>P0AGC3
MEKAKQVTWRLLAAGVCLLTVSSVARADSLDEQRSRYAQIKQAWDNRQMDVVEQMMPGLK
DYPLYPYLEYRQITDDLMNQPAVTVTNFVRANPTLPPARTLQSRFVNELARREDWRGLLA
FSPEKPGTTEAQCNYYYAKWNTGQSEEAWQGAKELWLTGKSQPNACDKLFSVWRASGKQD
PLAYLERIRLAMKAGNTGLVTVLAGQMPADYQTIASAIISLANNPNTVLTFARTTGATDF
TRQMAAVAFASVARQDAENARLMIPSLAQAQQLNEDQIQELRDIVAWRLMGNDVTDEQAK
WRDDAIMRSQSTSLIERRVRMALGTGDRRGLNTWLARLPMEAKEKDEWRYWQADLLLERG
REAEAKEILHQLMQQRGFYPMVAAQRIGEEYELKIDKAPQNVDSALTQGPEMARVRELMY
WNLDNTARSEWANLVKSKSKTEQAQLARYAFNNQWWDLSVQATIAGKLWDHLEERFPLAY
NDLFKRYTSGKEIPQSYAMAIARQESAWNPKVKSPVGASGLMQIMPGTATHTVKMFSIPG
YSSPGQLLDPETNINIGTSYLQYVYQQFGNNRIFSSAAYNAGPGRVRTWLGNSAGRIDAV
AFVESIPFSETRGYVKNVLAYDAYYRYFMGDKPTLMSATEWGRRY
>Q07982
MTNKISSSDNLSNAVSATDDNASRTPNLTRRALVGGGVGLAAAGALASGLQAATLPAGAS
QVPTTPAGRPMPYAIRPMPEDRRFGYAIVGLGKYALNQILPGFAGCQHSRIEALVSGNAE
KAKIVAAEYGVDPRKIYDYSNFDKIAKDPKIDAVYIILPNSLHAEFAIRAFKAGKHVMCE
KPMATSVADCQRMIDAAKAANKKLMIGYRCHYDPMNRAAVKLIRENQLGKLGMVTTDNSD
VMDQNDPAQQWRLRRELAGGGSLMDIGIYGLNGTRYLLGEEPIEVRAYTYSDPNDERFVE
VEDRIIWQMRFRSGALSHGASSYSTTTTSRFSVQGDKAVLLMDPATGYYQNLISVQTPGH
ANQSMMPQFIMPANNQFSAQLDHLAEAVINNKPVRSPGEEGMQDVRLIQAIYEAARTGRP
VNTDWGYVRQGGY
>P0AE85
MRIVTAAVMASTLAVSSLSHAAEVGSGDNWHPGEELTQRSTQSHMFDGISLTEHQRQQMR
DLMQQARHEQPPVNVSELETMHRLVTAENFDENAVRAQAEKMANEQIARQVEMAKVRNQM
YRLLTPEQQAVLNEKHQQRMEQLRDVTQWQKSSSLKLLSSSNSRSQ
>P13482
MKSPAPSRPQKMALIPACIFLCFAALSVQAEETPVTPQPPDILLGPLFNDVQNAKLFPDQ
KTFADAVPNSDPLMILADYRMQQNQSGFDLRHFVNVNFTLPKEGEKYVPPEGQSLREHID
GLWPVLTRSTENTEKWDSLLPLPEPYVVPGGRFREVYYWDSYFTMLGLAESGHWDKVADM
VANFAHEIDTYGHIPNGNRSYYLSRSQPPFFALMVELLAQHEGDAALKQYLPQMQKEYAY
WMDGVENLQAGQQEKRVVKLQDGTLLNRYWDDRDTPRPESWVEDIATAKSNPNRPATEIY
RDLRSAAASGWDFSSRWMDNPQQLNTLRTTSIVPVDLNSLMFKMEKILARASKAAGDNAM
ANQYETLANARQKGIEKYLWNDQQGWYADYDLKSHKVRNQLTAAALFPLYVNAAAKDRAN
KMATATKTHLLQPGGLNTTSVKSGQQWDAPNGWAPLQWVATEGLQNYGQKEVAMDISWHF
LTNVQHTYDREKKLVEKYDVSTTGTGGGGGEYPLQDGFGWTNGVTLKMLDLICPKEQPCD
NVPATRPTVKSATTQPSTKEAQPTP
>P25548
MKSIISLMAACAIGAASFAAPAFAQDKGSVGIAMPTKSSARWIDDGNNIVKQLQEAGYKT
DLQYADDDIPNQLSQIENMVTKGVKVLVIASIDGTTLSDVLKQAGEQGIKVIAYDRLIRN
SGDVSYYATFDNFQVGVLQATSITDKLGLKDGKGPFNIELFGGSPDDNNAFFFYDGAMSV
LKPYIDSGKLVVKSGQMGMDKVGTLRWDPATAQARMDNLLSAYYTDAKVDAVLSPYDGLS
IGIISSLKGVGYGTKDQPLPVVSGQDAEVPSVKSIIAGEQYSTIFKDTRELAKVTVNMVN
AVMEGKEPEVNDTKTYENGVKVVPSYLLKPVAVTKENYKQVLVDGGYYKEDQLK

>Q03026
MSASAKLSRMVCLLCGFFSTGISMASSLILLSASDLAGQWTLQQDEAPAICHLELRDSEV
AEASGYDLGGDTACLTRWLPSEPRAWRPTPAGIALLERGGLTLMLLGRQGEGDYRVQKGD
GGQLVLRRATP
>P26648
MSLSRRQFIQASGIALCAGAVPLKASAAGQQQPLPVPPLLESRRGQPLFMTVQRAHWSFT
PGTRASVWGINGRYLGPTIRVWKGDDVKLIYSNRLTENVSMTVAGLQVPGPLMGGPARMM
SPNADWAPVLPIRQNAATLWYHANTPNRTAQQVYNGLAGMWLVEDEVSKSLPIPNHYGVD
DFPVIIQDKRLDNFGTPEYNEPGSGGFVGDTLLVNGVQSPYVEVSRGWVRLRLLNASNSR
RYQLQMNDGRPLHVISGDQGFLPAPVSVKQLSLAPGERREILVDMSNGDEVSITCGEAAS
IVDRIRGFFEPSSILVSTLVLTLRPTGLLPLVTDSLPMRLLPTEIMAGSPIRSRDISLGD
DPGINGQLWDVNRIDVTAQQGTWERWTVRADEPQAFHIEGVMFQIRNVNGAMPFPEDRGW
KDTVWVDGQVELLVYFGQPSWAHFPFYFNSQTLEMADRGSIGQLLVNPVP
>P29898
MKRILTLTVAALALGTPALAYDGTNCKAPGNCWEPKPDYPAKVEGSKYDPQHDPAELSKQ
GESLAVMDARNEWRVWNMKKTGKFEYDVKKIDGYDETKAPPAE
>P0ABZ6
MKNWKTLLLGIAMIANTSFAAPQVVDKVAAVVNNGVVLESDVDGLMQSVKLNAAQARQQL
PDDATLRHQIMERLIMDQIILQMGQKMGVKISDEQLDQAIANIAKQNNMTLDQMRSRLAY
DGLNYNTYRNQIRKEMIISEVRNNEVRRRITILPQEVESLAQQVGNQNDASTELNLSHIL
IPLPENPTSDQVNEAESQARAIVDQARNGADFGKLAIAHSADQQALNGGQMGWGRIQELP
GIFAQALSTAKKGDIVGPIRSGVGFHILKVNDLRGESKNISVTEVHARHILLKPSPIMTD
EQARVKLEQIAADIKSGKTTFAAAAKEFSQDPGSANQGGDLGWATPDIFDPAFRDALTRL
NKGQMSAPVHSSFGWHLIELLDTRNVDKTDAAQKDRAYRMLMNRKFSEEAASWMQEQRAS
AYVKILSN
>P77202
MLKKILLLALLPAIAFAEELPAPVKAIEKQGITIIKTFDAPGGMKGYLGKYQDMGVTIYL
TPDGKHAISGYMYNEKGENLSNTLIEKEIYAPAGREMWQRMEQSHWLLDGKKDAPVIVYV
FADPFCPYCKQFWQQARPWVDSGKVQLRTLLVGVIKPESPATAAAILASKDPAKTWQQYE
ASGGKLKLNVPANVSTEQMKVLSDNEKLMDDLGANVTPAIYYMSKENTLQQAVGLPDQKT
LNIIMGNK
>P04816
MKRNAKTIIAGMIALAISHTAMADDIKVAVVGAMSGPIAQWGDMEFNGARQAIKDINAKG
GIKGDKLVGVEYDDACDPKQAVAVANKIVNDGIKYVIGHLCSSSTQPASDIYEDEGILMI
SPGATNPELTQRGYQHIMRTAGLDSSQGPTAAKYILETVKPQRIAIIHDKQQYGEGLARS
VQDGLKAANANVVFFDGITAGEKDFSALIARLKKENIDFVYYGGYYPEMGQMLRQARSVG
LKTQFMGPEGVGNASLSNIAGDAAEGMLVTMPKRYDQDPANQGIVDALKADKKDPSGPYV
WITYAAVQSLATALERTGSDEPLALVKDLKANGANTVIGPLNWDEKGDLKGFDFGVFQWH
ADGSSTAAK
>P05149
MKKLAILGVTVYSFAQLANAATLNVKSYGTTQNGQKVDLYTMSNNNGVSVSFISFGGVIT
QILTPDAQGKQNNIVLGFDDLKGYEVTDTKEGIHFGGLIGRYANRIGNAKFSLDGKTYNL
EKNNGPNSLHSGNPGFDKRVWQVKPLVSKGETVKASLKLTSPNGDQGFPGKLDVEVIYSL
SDQNEFKIEYKAKTDQPTVVNLTNHSYFNLSGAGNNPYGVLDHVVQLNAGRILVTDQNSL

PTGEIASVAGTPFDFRMPKAIVKDIRANNQQLAYGYGYDQTWVINQKSQGKLNLAAIVVD
PKSKRTMQVLTTEPSVQMYTADHLLGNIVGANGVLYRQADALALETQHFPDSPNQPTFPS
TRLNPNQTYNSVTVFKFGVQK
>Q9S1H0
MRKVMNSPDDGNGRRRFLQFSMAALASAAAPSSVWAFSKIQPIEDPLKSYPYRDWEDLYR
KEWTWDSTGFITHSNGCVAGCAWRVFVKNGVPMREEQVSEYPQLPGVPDMNPRGCQKGAV
YCSWSKQPDFLKYPLKRVGERGERKWKRISWDEAFTEIADKIIDTTVKRGPGNVCMPKRP
FAVITSAGYSRLANLIGAIKPDVSSMTGDLYPGIQTVRMPARTVSTFDDWFTSDLILMWH
KNPIVTRIPDAHFLTEARYNGARLVNISPDYNPSSVHADLHLPVTTGTDSHLAAAIVNVL
IADKKYKADYLKEQTDLPFLVRTDNGKFLREKDFNKDGSDEVFYIWDSKSGKAVLAPGSM
GSKDKTLKLGAVEPALEGTFDANGIEVTTVFARLKAEIAPYTPEATHKTTGIHPSVVRQL
AGWIGDCKALRILDGYNNQKHFDGFQCGRLKILILTLIGHHGTTGSIDTTYEGWVLEGNK
ALGGVKGRPGRSVSMVLAQWVWGEQYRRSKAYFDDTELREQIGFGVDEMEALRKESEANG
WMPNWQSIKDPVVYINAGINTFATSTGYQHLRENFLKRCELYVVVDFRLNSGAMYADIVL
PAATNLEKLDIRETSSTRFIHAFGQPIKPMYDRRTDWQISVGLARKIQERARARGITRVD
DPEIKSFIDFDKVYDEFTMNGAVEKDEDALRFVMEKSKALGPGSYEEVLKRGFVGVGPSA
GKTGPVPADKPYRPFTVNVSEKVPYKTLTGRLQFYIDHDWYQRFGATVPKPQYGGGVLGP
KKYPFVYNTPHTRWGVHSFARTDQWMLRHQRGEPDVRLNPAAMARKGIKDGDQVRIFNSS
GEFFAMAKAWPGLPENMLFSEHGWEQYLYKNMTHYNSVNAELINPLELVGGYGHVKFAAG
GFNPNRIFHETTVDVEKA
>Q51658
MLRLACLAPLAILIPAAGTAEQARPADDALAALGAQLFVDPALSRNATQSCATCHDPARA
FTDPREGKAGLAVSVGDDGQSHGDRNTPTLGYAALVPAFHRDANGKYKGGQFWDGRADDL
KQQAGQPMLNPVEMAMPDRAAVAARLRDDPAYRTGFEALFGKGVLDDPERAFDAAAEALA
AYQATGEFSPFDSKYDRVMRGEEKFTPLEEFGYTVFITWNCRLCHMQRKQGVAERETFTN
FEYHNIGLPVNETAREASGLGADHVDHGLLARPGIEDPAQSGRFKVPSLRNVAVTGPYMH
NGVFTDLRTAILFYNKYTSRRPEAKINPETGAPWGEPEVARNLSLAELQSGLMLDDGRVD
ALVAFLETLTDRRYEPLLEESRAAQKD
>P33979
MPRSLFRTVLTALVAASCLIAAPALAKSRIKDIVSFEGVRENQLIGYGIVVGLNGTGDSL
RNAPMTKQSLEAMLERQGVNVRDNNLNTKNTAAVMVTANLPPFSASGSKVDVTVSTLGDA
KSLLGGTLLVTSLQGADGQTYAVAQGTVQTGSVSAGGASGSSVTKGVPTAGRIAGGGVIE
RETGFQMVNMDIMRLTLRNPDFTTARRVADAINAKFPGCAQAQNPTIIATRPPPGMDMIS
FMTNIENLMVEPDGPAKVVIDEVAGVIVMGDDVRISQVAIAQGNLTITVQENPAVSQPAP
FSQGQTAVVPQSTVNVEEEKGKQLLTLGGAPSLKGLIGGLNALGVTPRDMISILQAVKAA
GALQADIEVM
>P0AAJ8
MNRRNFIKAASCGALLTGALPSVSHAAAENRPPIPGSLGMLYDSTLCVGCQACVTKCQDI
NFPERNPQGEQTWSNNDKLSPYTNNIIQVWTSGTGVNKDQEENGYAYIKKQCMHCVDPNC
VSVCPVSALKKDPKTGIVHYDKDVCTGCRYCMVACPYNVPKYDYNNPFGALHKCELCNQK
GVERLDKGGLPGCVEVCPAGAVIFGTREELMAEAKKRLALKPGSEYHYPRQTLKSGDTYL
HTVPKYYPHLYGEKEGGGTQVLVLTGVPYENLDLPKLDDLSTGARSENIQHTLYKGMMLP
LAVLAGLTVLVRRNTKNDHHDGGDDHES
>P51697

MSDLDRLASRAAIQDLYSDKLIAVDKRQEGRLASIWWDDAEWTIEGIGTYKGPEGALDLA
NNVLWPMFHECIHYGTNLRLEFVSADKVNGIGDVLLLGNLVEGNQSILIAAVFTDEYERR
DGVWKFSKRNACTNYFTPLAGIHFAPPGIHFAPSGA
>P0A855
MKQALRVAFGFLILWASVLHAEVRIVIDSGVDSGRPIGVVPFQWAGPGAAPEDIGGIVAA
DLRNSGKFNPLDRARLPQQPGSAQEVQPAAWSALGIDAVVVGQVTPNPDGSYNVAYQLVD
TGGAPGTVLAQNSYKVNKQWLRYAGHTASDEVFEKLTGIKGAFRTRIAYVVQTNGGQFPY
ELRVSDYDGYNQFVVHRSPQPLMSPAWSPDGSKLAYVTFESGRSALVIQTLANGAVRQVA
SFPRHNGAPAFSPDGSKLAFALSKTGSLNLYVMDLASGQIRQVTDGRSNNTEPTWFPDSQ
NLAFTSDQAGRPQVYKVNINGGAPQRITWEGSQNQDADVSSDGKFMVMVSSNGGQQHIAK
QDLATGGVQVLSSTFLDETPSLAPNGTMVIYSSSQGMGSVLNLVSTDGRFKARLPATDGQ
VKFPAWSPYL
>P77368
MKTLIVSTVLAFITFSAQAAAFQVTSNEIKTGEQLTTSHVFSGFGCEGGNTSPSLTWSGV
PEGTKSFAVTVYDPDAPTGSGWWHWTVVNIPATVTYLPVDAGRRDGTKLPTGAVQGRNDF
GYAGFGGACPPKGDKPHHYQFKVWALKTEKIPVDSNSSGALVGYMLNANKIATAEITPVY
EIK
>Q54468
MNAFKLSALARLTATMGFLGGMGSAMADQQLVDQLSQLKLNVKMLDNRAGENGVDCAALG
ADWASCNRVLFTLSNDGQAIDGKDWVIYFHSPRQTLRVDNDQFKIAHLTGDLYKLEPTAK
FSGFPAGKAVEIPVVAEYWQLFRNDFLPRWYATSGDAKPKMLANTDTENLDQFVAPFTGD
QWKRTKDDKNILMTPASRFVSNADLQTLPAGALRGKIVPTPMQVKVHAQDADLRKGVALD
LSTLVKPAADVVSQRFALLGVPVQTNGYPIKTDIQPGKFKGAMAVSGAYELKIGKKEAQV
IGFDQAGVFYGLQSILSLVPSDGSGKIATLDASDAPRFPYRGIFLDVARNFHKKDAVLRL
LDQMAAYKLNKFHFHLSDDEGWRIEIPGLPELTEVGGQRCHDLSETTCLLPQYGQGPDVY
GGFFSRQDYIDIIKYAQARQIEVIPEIDMPAHARAAVVSMEARYKKLHAAGKEQEANEFR
LVDPTDTSNTTSVQFFNRQSYLNPCLDSSQRFVDKVIGEIAQMHKEAGQPIKTWHFGGDE
AKNIRLGAGYTDKAKPEPGKGIIDQSNEDKPWAKSQVCQTMIKEGKVADMEHLPSYFGQE
VSKLVKAHGIDRMQAWQDGLKDAESSKAFATSRVGVNFWDTLYWGGFDSVNDWANKGYEV
VVSNPDYVYMDFPYEVNPDERGYYWGTRFSDERKVFSFAPDNMPQNAETSVDRDGNHFNA
KSDKPWPGAYGLSAQLWSETQRTDPQMEYMIFPRALSVAERSWHRAGWEQDYRAGREYKG
GETHFVDTQALEKDWLRFANILGQRELAKLDKGGVAYRLPVPGARVAAGKLEANIALPGL
GIEYSTDGGKQWQRYDAKAKPAVSGEVQVRSVSPDGKRYSRAEKV
>P52236
MARFSPMMLLPVAIFAGFAGLSGWALLRDDPDALPSAMIGREAPSVGEATLPGKVQLTDE
MLRQPGVKLVNFWASWCPPCRAEHPTLTELSARLPVYGVDLKDPEGAALGFLSEHGDPFH
ALAADPRGRVAIDWGVTAPPETFIIDGSGRILHRHAGPLVREDYTNRFLPELEKALAAE
>P43478
MKPISIVAFPIPAISMLLLSAVSQAASMQPPIAKPGETWILQAKRSDEFNVKDATKWNFQ
TENYGVWSWKNENATVSNGKLKLTTKRESHQRTFWDGCNQQQVANYPLYYTSGVAKSRAT
GNYGYYEARIKGASTFPGVSPAFWMYSTIDRSLTKEGDVQYSEIDVVELTQKSAVRESDH
DLHNIVVKNGKPTWMRPGSFPQTNHNGYHLPFDPRNDFHTYGVNVTKDKITWYVDGEIVG
EKDNLYWHRQMNLTLSQGLRAPHTQWKCNQFYPSANKSAEGFPTSMEVDYVRTWVKVGNN
NSAPGEGQSCPNTFVAVNSVQLSAAKQTLRKGQSTTLESTVLPNCATNKKVIYSSSNKNV

ATVNSAGVVKAKNKGTATITVKTKNKGKIDKLTIAVN
>Q06062
MNPMTRRHTWTRLACALSLGVAAFAAQADEGALYGPQAPKGSAFVRAYNAGNSELDVSVG
STSLNDVAPLGSSDFKFLPPGSYTAQVGQQSLPVKLDPDSYYTLVSQPGGKPQLVAEPPF
KNKQKALVRVQNLSGSKLTLKTADGKTDVVKDVGPQSHGDREINPVKVNLALFDGSKKVS
DLKPVTLARGEVVCLYVTGSGGKLAPVWVKRPVKAD
>Q05097
MAWKGEVLANNEAGQVTSIIYNPGDVITIVAAGWASYGPTQKWGPQGDREHPDQGLICHD
AFCGALVMKIGNSGTIPVNTGLFRWVAPNNVQGAITLIYNDVPGTYGNNSGSFSVNIGKD
QS
>P29484
MNIKLSFISIAFLSLSFNVAANEFEKSQEHYKSVTDLKNKIEILELEKKITELSGEIRNA
RMPKIDKSAPVLSPQPVVKSSEELQKSIEHIEEELKVELAYLVNNGQQKKYTFNLNGKLI
TLVNGDFVNGWKFIEDQNKIQFSKGNKVIDVN
>P12374
MESRTSRRTFVKGLAAAGVLGGLGLWRSPSWAASGSPALSVLSGTEFDLSIGEMPVNITG
RRRTAMAINGGLPGPLLRWKEGDTVTLRVRNRLDAATSIHWHGIILPPNMDGVPGLSFAG
IEPGGVYVYQFKVQQNGTYWYHSHSGFQEQVGVYGPLVIEAKEPEPFKYDSEHVVMLTDW
TDEDPVSLMRTLKKQSDYYNFHKRTVGDFVNDVADKGWAATVADRKMWAEMKMNPTDLAD
VSGATYTYLLNGQAPNMNWTGLFRPGEKLRLRFINGSAMTYFDIRIPGLKMTVVASDGQF
VNPVEVDELRIAVAETFDVIVEPTAEAYTVFAQSMDRTGYARGTLAVREGLVAQVPPLDP
RPLVTMDDMGMGGMDHGSMDGMSGMDSGADDGMQTMSSMGGDSMPAMDHSKMSTMQGMDH
GAMSGMDHGAMGGMVMQSHPASENDNPLVDMQAMSPTAKLNDPGLGLRNNGRKVLTYADL
KSTFEDPDGREPSRTIELHLTGHMEKFAWSFDGIKFADAQPLILKYGERVRIVLVNDTMM
THPIHLHGMWSDLEDEDGNFRVRKHTIDMPPGSKRSYRVTADALGRWAYHCHLLYHMEMG
MFREVRVEE
>P0A0Y3
MKTSIRYALLAAALTAATPALADITVYNGQHKEAAQAVADAFTRATGIKVKLNSAKGDQL
AGQIKEEGSRSPADVFYSEQIPALATLSAANLLEPLPASTINETRGKGVPVAAKKDWVAL
SGRSRVVVYDTRKLSEKDLEKSVLNYATPKWKNRIGYAPTSGAFLEQVVAIVKLKGEAAA
LKWLKGLKEYGKPYAKNSVALQAVENGEIDAALINNYYWHAFAREKGVQNVHTRLNFVRH
RDPGALITYSGAAVLKSSQNKDEAKKFVAFLASKEGQRALTAVRAEYPLNPHVVSTFNLE
PIAKLEAPQVSATTVSEKEHATRLLEQAGMK
>P39172
MLHKKTLLFAALSAALWGGATQAADAAVVASLKPVGFIASAIADGVTETEVLLPDGASEH
DYSLRPSDVKRLQNADLVVWVGPEMEAFMQKPVSKLPGAKQVTIAQLEDVKPLLMKSIHG
DDDDHDHAEKSDEDHHHGDFNMHLWLSPEIARATAVAIHGKLVELMPQSRAKLDANLKDF
EAQLASTETQVGNELAPLKGKGYFVFHDAYGYFEKQFGLTPLGHFTVNPEIQPGAQRLHE
IRTQLVEQKATCVFAEPQFRPAVVESVARGTSVRMGTLDPLGTNIKLGKTSYSEFLSQLA
NQYASCLKGD
>P24059
MHLHLRGICLVLAVASSSSSALAADAGHGADLAKRWCASCHVVANGQAVASADVPSFASV
ARRPDFSSEKLAFFLLDPHPKMPSFPLSRTEAGDIAAYIGSLRP
>P24092

MRNGRTLLRWAGVLAATAIIGVGGFWSQGTTKALPEGPGEKRADLIEIGAMERFGKLDLP
KVAFRHDQHTTAVTGMGKDCAACHKSKDGKMSLKFMRLDDNSAAELKEIYHANCIGCHTD
LAKAGKKTGPQDGECRSCHNPKPSAASSWKEIGFDKSLHYRHVASKAIKPVGDPQKNCGA
CHHVYDEASKKLVWGKNKEDSCRACHGEKPVDKRPALDTAAHTACISCHMDVAKTKAETG
PVNCAGCHAPEAQAKFKVVREVPRLDRGQPDAALILPVPGKDAPREMKGTMKPVAFDHKA
HEAKANDCRTCHHVRIDTCTACHTVNGTADSKFVQLEKAMHQPDSMRSCVGCHNTRVQQP
TCAGCHGFIKPTKSDAQCGVCHVAAPGFDAKQVEAGALLNLKAEQRSQVAASMLSARPQP
KGTFDLNDIPEKVVIGSIAKEYQPSEFPHRKIVKTLIAGIGEDKLAATFHIEKGTLCQGC
HHNSPASLTPPKCASCHGKPFDADRGDRPGLKAAYHQQCMGCHDRMKIEKPANTACVDCH
KERAK
>P45523
MKSLFKVTLLATTMAVALHAPITFAAEAAKPATAADSKAAFKNDDQKSAYALGASLGRYM
ENSLKEQEKLGIKLDKDQLIAGVQDAFADKSKLSDQEIEQTLQAFEARVKSSAQAKMEKD
AADNEAKGKEYREKFAKEKGVKTSSTGLVYQVVEAGKGEAPKDSDTVVVNYKGTLIDGKE
FDNSYTRGEPLSFRLDGVIPGWTEGLKNIKKGGKIKLVIPPELAYGKAGVPGIPPNSTLV
FDVELLDVKPAPKADAKPEADAKAADSAKK
>P50024
MFKKLIGLLFLMPMTALATQFIEGKDYQTVASAQLSTNKDKTPLITEFFSYGCPWCYKID
APLNDWATRMGKGAHLERVPVVFKPNWDLYAKAYYTAKTLAMSDKMNPILFKAIQEDKNP
LATKQSMVDFFVAHGVDREIAKSAFENSPTIDMRVNSGMSLMAHYQINAVPAFVVNNKYK
TDLQMAGSEERLFEILNYLVRKSA
>P36560
MKKVLALVVAAAMGLSSAAFAAETTTTPAPTATTTKAAPAKTTHHKKQHKAAPAQKAQAA
KKHHKNTKAEQKAPEQKAQAAKKHAKKHSHQQPAKPAAQPAA
>P26982
MKKTTLAMSALALSLGLALSPLSATAAETSSSAMTAQQMPSLAPMLEKVMPSVVSINVEG
STTVNTPRMPRNFQQFFGDDSPFCQDGSPFQNSPFCQGGGNGGNGGQQQKFMALGSGVII
DAAKGYVVTNNHVVDNASVIKVQLSDGRKFDAKVVGKDPRSDIALIQIQNPKNLTAIKLA
DSDALRVGDYTVAIGNPFGLGETVTSGIVSALGRSGLNVENYENFIQTDAAINRGNSGGA
LVNLNGELIGINTAILAPDGGNIGIGFAIPSNMVKNLTSQMVEYGQVKRGELGIMGTELN
SELAKAMKVDAQRGAFVSQVMPNSSAAKAGIKAGDVITSLNGKPISSFAALRAQVGTMPV
GSKISLGLLREGKAITVNLELQQSSQSQVDSSTIFSGIEGAEMSNKGQDKGVVVSSVKAN
SPAAQIGLKKGDVIIGANQQPVKNIAELRKILDSKPSVLALNIQRGDSSIYLLMQ
>P09394
MKLTLKNLSMAIMMSTIVMGSSAMAADSNEKIVIAHRGASGYLPEHTLPAKAMAYAQGAD
YLEQDLVMTKDDNLVVLHDHYLDRVTDVADRFPDRARKDGRYYAIDFTLDEIKSLKFTEG
FDIENGKKVQTYPGRFPMGKSDFRVHTFEEEIEFVQGLNHSTGKNIGIYPEIKAPWFHHQ
EGKDIAAKTLEVLKKYGYTGKDDKVYLQCFDADELKRIKNELEPKMGMELNLVQLIAYTD
WNETQQKQPDGSWVNYNYDWMFKPGAMKQVAEYADGIGPDYHMLIEETSQPGNIKLTGMV
QDAQQNKLVVHPYTVRSDKLPEYTPDVNQLYDALYNKAGVNGLFTDFPDKAVKFLNKE
>P0AEU7
MKKWLLAAGLGLALATSAQAADKIAIVNMGSLFQQVAQKTGVSNTLENEFKGRASELQRM
ETDLQAKMKKLQSMKAGSDRTKLEKDVMAQRQTFAQKAQAFEQDRARRSNEERGKLVTRI
QTAVKSVANSQDIDLVVDANAVAYNSSDVKDITADVLKQVK

>P23827
MKTILPAVLFAAFATTSAWAAESVQPLEKIAPYPQAEKGMKRQVIQLTPQEDESTLKVEL
LIGQTLEVDCNLHRLGGKLENKTLEGWGYDYYVFDKVSSPVSTMMACPDGKKEKKFVTAY
LGDAGMLRYNSKLPIVVYTPDNVDVKYRVWKAEEKIDNAVVR
>P06202
MSNITKKSLIAAGILTALIAASAATAADVPAGVQLADKQTLVRNNGSEVQSLDPHKIEGV
PESNVSRDLFEGLLISDVEGHPSPGVAEKWENKDFKVWTFHLRENAKWSDGTPVTAHDFV
YSWQRLADPNTASPYASYLQYGHIANIDDIIAGKKPATDLGVKALDDHTFEVTLSEPVPY
FYKLLVHPSVSPVPKSAVEKFGDKWTQPANIVTNGAYKLKNWVVNERIVLERNPQYWDNA
KTVINQVTYLPISSEVTDVNRYRSGEIDMTYNNMPIELFQKLKKEIPNEVRVDPYLCTYY
YEINNQKAPFNDVRVRTALKLALDRDIIVNKVKNQGDLPAYSYTPPYTDGAKLVEPEWFK
WSQQKRNEEAKKLLAEAGFTADKPLTFDLLYNTSDLHKKLAIAVASIWKKNLGVNVNLEN
QEWKTFLDTRHQGTFDVARAGWCADYNEPTSFLNTMLSDSSNNTAHYKSPAFDKLIADTL
KVADDTQRSELYAKAEQQLDKDSAIVPVYYYVNARLVKPWVGGYTGKDPLDNIYVKNLYI
IKH
>P0A1W4
MKNIRNFSIIAHIDHGKSTLSDRIIQICGGLSDREMEAQVLDSMDLERERGITIKAQSVT
LDFKASDGETYQLNFIDTPGHVDFSYEVSRSLAACEGALLVVDAGQGVEAQTLANCYTAM
EMDLEVVPVLNKIDLPAADPERVAEEIEDIVGIDATDAVRCSAKTGVGVTDVLERLVRDI
PPPQGDPDGPLQALIIDSWFDNYLGVVSLVRIKNGTMRKGDKIKVMSTGQTYNADRLGIF
TPKQVDRTELKCGEVGWLVCAIKDILGAPVGDTLTSARNPAEKALPGFKKVKPQVYAGLF
PVSSDDYESFRDALGKLSLNDASLFYEPESSSALGFGFRCGFLGLLHMEIIQERLEREYD
LDLITTAPTVVYEVETTAKETIYVDSPSKLPPLNNIYELREPIAECHMLLPQAYLGNVIT
LCIEKRGVQTNMVYHGNQVALTYEIPMAEVVLDFFDRLKSTSRGYASLDYNFKRFQASDM
VRVDVLINNERVDALALITHRDNSQSRGRELVEKMKDLIPRQQFDIAIQAAIGTHIIARS
TVKQLRKNVLAKCYGGDISRKKKLLQKQKEGKKRMKQIGNVELPQEAFLAILHVGKDNK
>P0A321
MFKQVLGGMALMAAFSAPVLAAECSVDIAGTDQMQFDKKAIEVSKSCKQFTVNLKHTGKL
PRNVMGHNWVLTKTADMQAVEKDGIAAGLDNQYLKAGDTRVLAHTKVLGGGESDSVTFDV
AKLAAGDDYTFFCSFPGHGALMKGTLKLVD
>P13628
MQIVNLTRRGFLKAACVVTGGALISIRMTGKAVAAAKQLKDYMMDRINGVYGADAKFPVR
ASQDNVQVQKLYADFLEKPMSHKAEQLLHTHWVDRSKAIERMKAQGAYPNPRAKEFEGNT
YPYE
>Q56686
MFKNKLAVLFTCLSVFSFSAQSGSFDTVTLGSKGGIQDGNLTAFLIKSEADSNFVMLDAG
SVVNGLIVSEQKGAFKDITVPDSSPYTKVGYLLKDRIKGYFISHAHLDHVAGLIISSPDD
SKKPIYGLAATNKDLMKNYFNWSAWPNFGNKGEGFKLNKYNYVDLQPGVWSPVAETTMSV
VSLPLSHSGGQSTVFILKDSEGDVFAYFGDTGPDEVEKSSAMRTAWSVLAPFVKQGKLKG
IIIEVSFTNETPDKSLFGHLTPNWLVKELSVLEDMNGKGSLKDLNVAISHIKYSLKNSED
PKVIIKKQLVEVNDLGVNFIFPEQGDSLQF
>P0C0T5
MNKTAIALLALLASSASLAATPWQKITQPVPGSAQSIGSFSNGCIVGADTLPIQSEHYQV
MRTDQRRYFGHPDLVMFIQRLSSQVSNLGMGTVLIGDMGMPAGGRFNGGHASHQTGLDVD

IFLQLPKTRWTSAQLLRPQALDLVSRDGKHVVSTLWKPEIFSLIKLAAQDKDVTRIFVNP
AIKQQLCLDAGTDRDWLRKVRPWFQHRAHMHVRLRCPADSLECEDQPLPPSGDGCGAELQ
SWFEPPKPGTTKPEKKTPPPLPPSCQALLDEHVI
